# Optimization of a Deep-Learning Method Based on the Classification of Images Generated by Parameterized Deep Snap a Novel Molecular-Image-Input Technique for Quantitative Structure–Activity Relationship (QSAR) Analysis

**DOI:** 10.3389/fbioe.2019.00065

**Published:** 2019-03-28

**Authors:** Yasunari Matsuzaka, Yoshihiro Uesawa

**Affiliations:** Department of Medical Molecular Informatics, Meiji Pharmaceutical University, Tokyo, Japan

**Keywords:** chemical structure, constitutive androgen receptor, Deep Snap, Tox21, deep learning, QSAR, molecular image

## Abstract

Numerous chemical compounds are distributed around the world and may affect the homeostasis of the endocrine system by disrupting the normal functions of hormone receptors. Although the risks associated with these compounds have been evaluated by acute toxicity testing in mammalian models, the chronic toxicity of many chemicals remains due to high cost of the compounds and the testing, etc. However, computational approaches may be promising alternatives and reduce these evaluations. Recently, deep learning (DL) has been shown to be promising prediction models with high accuracy for recognition of images, speech, signals, and videos since it greatly benefits from large datasets. Recently, a novel DL-based technique called DeepSnap was developed to conduct QSAR analysis using three-dimensional images of chemical structures. It can be used to predict the potential toxicity of many different chemicals to various receptors without extraction of descriptors. DeepSnap has been shown to have a very high capacity in tests using Tox21 quantitative qHTP datasets. Numerous parameters must be adjusted to use the DeepSnap method but they have not been optimized. In this study, the effects of these parameters on the performance of the DL prediction model were evaluated in terms of the loss in validation as an indicator for evaluating the performance of the DL using the toxicity information in the Tox21 qHTP database. The relations of the parameters of DeepSnap such as (1) number of molecules per SDF split into (2) zoom factor percentage, (3) atom size for van der waals percentage, (4) bond radius, (5) minimum bond distance, and (6) bond tolerance, with the validation loss following quadratic function curves, which suggests that optimal thresholds exist to attain the best performance with these prediction models. Using the parameter values set with the best performance, the prediction model of chemical compounds for CAR agonist was built using 64 images, at 105° angle, with AUC of 0.791. Thus, based on these parameters, the proposed DeepSnap-DL approach will be highly reliable and beneficial to establish models to assess the risk associated with various chemicals.

## Introduction

The traditional human-safety assessment of chemical compounds involves repetitive-dosage subacute toxicity testing *in vivo* using animal models. However, the risk remains that such compounds could pose major public health concerns to humans by potentially disrupting normal endocrine functions with various hormone receptors upon long-term exposure (Genuis and Kyrillos, 2017; Heindel et al., 2017; Manibusan and Touart, 2017; Sifakis et al., 2017; Tapia-Orozco et al., 2017; Heindel, 2018; Marty et al., 2018). However, since some molecular mechanisms differ between species and depend on environmental factors, it is often difficult to apply the outcomes of animal testing to predict the effects on human health (Brockmeier et al., 2017; Leist et al., 2017; Fay et al., 2018). Moreover, a large number of chemical substances need to be studied to identify the adverse effects on development, metabolic homeostasis, reproduction, cytotoxicity, etc. (Zhu et al., 2014; Bell et al., 2017; Insel et al., 2017; Juberg et al., 2017; Clark and Steger-Hartmann, 2018; Mortensen et al., 2018). Thus, high-throughput (HTP) assays and economical methods are required (Tollefsen et al., 2014; Chen et al., 2015; Wang et al., 2015; Richard et al., 2016). Alternative computational prediction methods based on *in-silico* experiments are essential for conducting safety evaluations of high-risk chemical substances (Malloy et al., 2017; Lo et al., 2018; Luechtefeld et al., 2018; Zhang et al., 2018). Among these, quantitative structure–activity relationship (QSAR) analysis can predict physiological activity, toxicity, enzymatic reactions, receptor agonist/antagonist activity, environmental fate, etc. (Bloomingdale et al., 2017; Polishchuk, 2017; Halder et al., 2018; Khan and Roy, 2018; Simões et al., 2018). This analysis is conducted based on a formulation of established rules for the relationship between the chemical structure of a compound and its activity and relies on the structural, quantum chemical, and physicochemical features, which are represented as various numerical molecular descriptors (Dougall, 2001; Fang et al., 2003; Roy and Das, 2014; Silva and Trossini, 2014). However, there are limited programs that can precisely evaluate the response patterns of cellular signaling molecules due to various chemical compounds.

These days, machine learning has been applied in extensive toxicological fields, and it is highly effective for risk assessment (Ambe et al., 2018; Banerjee et al., 2018; Luechtefeld et al., 2018; Cipullo et al., 2019). More recently, deep learning (DL), a machine-learning method designed to extract and recognize discriminative information patterns and rules, has been proposed to identify features by several flexible fully-connected layers of a neural network (NN) (Li S. et al., 2017; Qiu et al., 2017; Hu et al., 2018; Li H. et al., 2018; Luechtefeld et al., 2018; Mayr et al., 2018). Until today, support vector machine, random forest, and artificial NN were needed to select a reasonable combination of features (corresponding to chemical structure descriptors in QSAR analysis) manually when learning (feature selection techniques). In many cases, it is extremely difficult to find the optimal solutions, since myriad (Manallack et al., 2010; Talevi et al., 2012; Guimarães et al., 2016; Fang et al., 2017). Therefore, various approximation methods have been developed to obtain an optimal combination for an approximate solution (Yap et al., 2007; Kulkarni et al., 2009). However, since there is no completely trustworthy approximation method, complicated craftsmanship procedures are required to extract effective features in conventional machine learning.

On the other hand, a convolutional neural network (CNN) that constitutes DL has a function of feature expression learning that makes it automatically extract features and unnecessary to manually extract features (Fernandez et al., 2018; Lumini and Nanni, 2018). Unlike the conventional method, which is essential for extraction of a molecular structure descriptor, it is able to identify the most informative features required automatically, which is useful for prediction from the input information of the entire molecule “without supervision” by hierarchically decomposing an image so that the CNN learns to recognize higher-quality features while maintaining their spatial relationships (Ma et al., 2015; Ragoza et al., 2017; Xu et al., 2017; Ghasemi et al., 2018; Liu R. et al., 2018; Peng et al., 2018). These layer structures of the DL consist of input, hidden intermediate, and output layers of a NN, which is an algorithm designed for pattern recognition where information flows and is referred to as a deep neural network (DNN) (LeCun et al., 2015; Mallat, 2016; Suárez-Paniagua and Segura-Bedmar, 2018; Voulodimos et al., 2018). In this DNN, it is possible to directly learn feature quantity contained in a large amount of input data without human intervention at each layer (Azimi et al., 2018). Moreover, it poses a capacity to improve the prediction accuracy for very complicated image recognition by increasing the information transmission and processing ability using a large number of hidden layers and some techniques such as dropout, data augmentation, Rectified Linear Units (ReLUs), and multiple graphics processing units (GPUs) (Rawat and Wang, 2017; Gawehn et al., 2018; Ha et al., 2018; Hussain et al., 2018; Poernomo and Kang, 2018; Qiao et al., 2018; Saha et al., 2018; Sato et al., 2018; Shen et al., 2018; Steven and Han, 2018; Tustison et al., 2018; Vakli et al., 2018; Wang S. H. et al., 2018). Therefore, it is also possible to cope with the deviation and the deformation of the position of input image data for detecting on the edge region (Krizhevsky et al., 2012). However, since the result depends on the size of the filter, the moving width, and settings such as padding (the process of filling that allocates the end of region with 0 to pad out the number of convolutions of the edge region of the image) (Szegedy et al., 2014; Johnson and Zhang, 2015). In addition, CNNs appropriate combinations of extracted constituent elements and data orderly to the next layer, so it is possible to efficiently learn feature quantities (Szegedy et al., 2014; Cagli et al., 2017).

Studies have reported very high prediction accuracy DL with highly non-linear hierarchical patterns based on large-scale data, especially in the fields of imaging and toxicology (LeCun et al., 2015; Ma et al., 2015; Mayr et al., 2016; Pastur-Romay et al., 2016; Zhang et al., 2017). In addition, some studies have demonstrated the use of DL in QSAR analysis to calculate feature values from molecular structures without human intervention that three steps: (1) model building from labeled data inputs, (2) evaluation and tuning of the model, and (3) training the final model to perform prediction (Bengio et al., 2013; LeCun et al., 2015; Ma et al., 2015; Mayr et al., 2016; Pastur-Romay et al., 2016; Pham et al., 2017; Zhang et al., 2017). However, since for delivering information on the whole molecule sufficiently established most of the cases where DL is applied to QSAR on conventional descriptor calculation at present. Therefore, further work is required to increase prediction accuracy for applications DL for QSAR analysis. First, a systematic and suitable input is required for complicated data such as the three-dimensional (3D) structures of chemical compounds. Moreover, as a result of the insufficient amount of chemical compounds, there is a lack of training data. To address these issues, a novel QSAR model using DL based on 3D molecular images of chemical compounds was previously developed (Uesawa, 2018).

**Figure 1:**
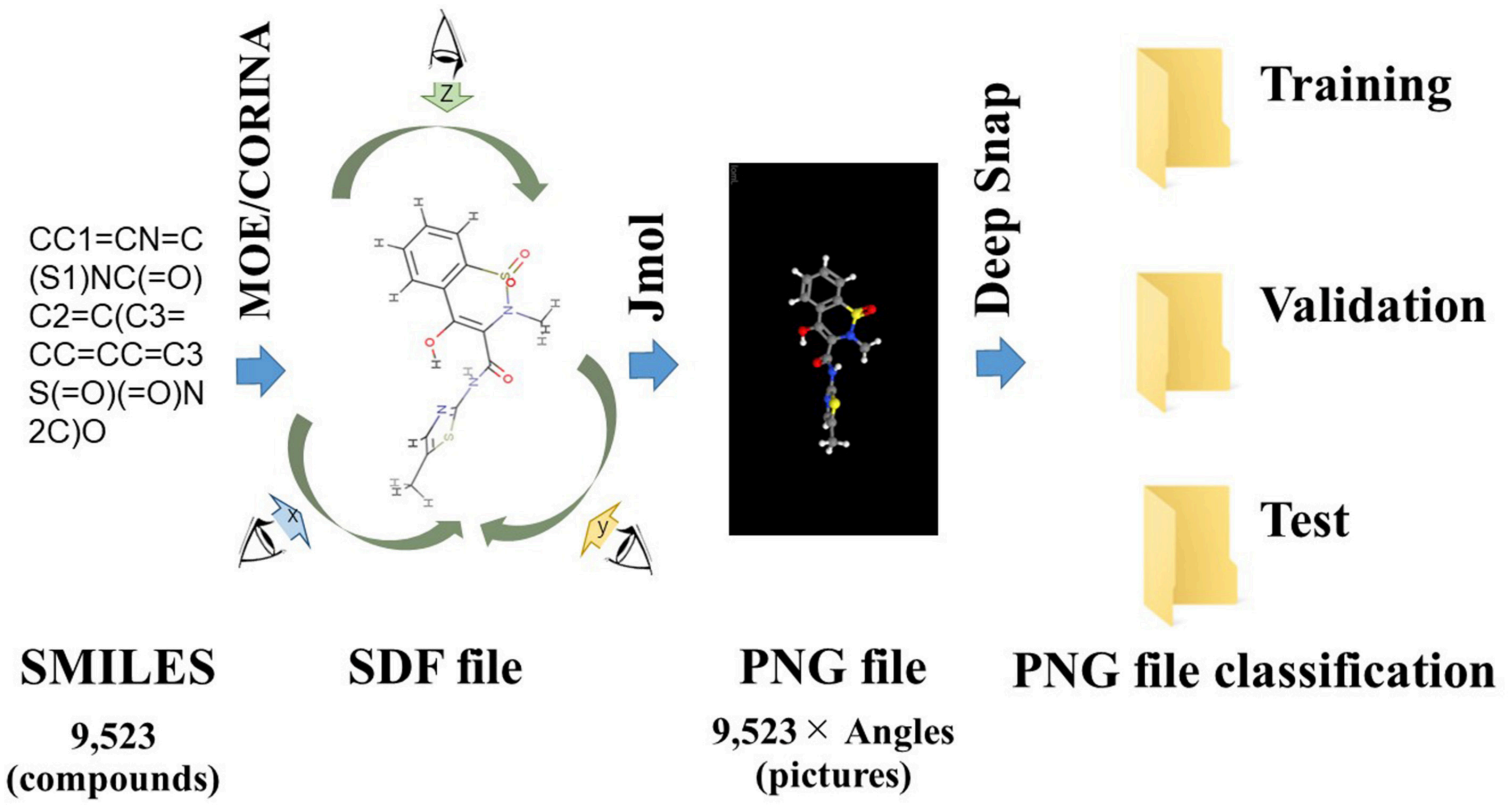
Schematic of the Deep Snap procedure. 9,523 SMILES 3D structures by CORINA Classic software after washing by MOE application, and into SDF file format, and then photograph an arbitrary angle on the x-, y-, and z-axes by Jmol-Deep Snap. The resulted images are saved as PNG files in three datasets (training, validation, and test) in order to input DL.

Deep Snap is a procedure of generating an omnidirectional snapshot portraying 3D structures of chemical compounds using a drawing software (Jmol; Hanson, 2016) based on the Structure Data File (SDF) format (Figure 1). The 3D information is input into the DL model without calculating structural descriptors. For example, when the 3D molecular structure is rotated in 45° increments on the x-, y-, and z-axes and photographed, a total of 512 images are captured for each molecule and saved in the portable network graphics (PNG) format. This allows for combining digital information regarding the 2D plane location of the atoms with pixel-level data representing the three primary colors (RGB) (Figure 1; Uesawa, 2018). Then, these images are used in inputs of the DL model after a resolution of 256 × 256 pixels images of the 3D molecular structure are represented as a ball-and-stick model for each atomic composition with different colors representing different atoms (Uesawa, 2018). We refer to this omnidirectional snapshot capturing procedure for 3D structures of compounds as “Deep Snap.”

In the Tox21 data challenge in 2014, a crowd-sourced QSAR competition for chemical risk assessment held by the National Institutes of Health (NIH) in the United States (Tox21 Data Challenge., 2014), approximately 7,000–9,000 different chemical structures depending on the target type. This data was split evenly into training and validation datasets (a 50% of training and a 50% of validation) that were created for the purpose of developing high-performance prediction models for various adverse-outcome pathways (Attene-Ramos et al., 2013; Tox21 Data Challenge., 2014. Recently, using a set of these chemicals (containing a total of 7,320 different molecules with 3,660 reserved for training and 3,660 reserved for validation), the Deep Snap procedure was applied to successfully predict which chemical compounds disrupt the potential of the mitochondrial membrane (MMP), which play pivotal roles in apoptosis, oxidative phosphorylation, calcium homeostasis, and cellular metabolism such as heme, fatty acid, and steroid synthesis (Midzak et al., 2011; Hua et al., 2012; Bolisetty et al., 2013; Shaughnessy et al., 2014; Li A. X. et al., 2017; Liu et al., 2017; Yun et al., 2017; Wang C. et al., 2018). Individual compounds well-known inhibitors for complex between uncouplers (e.g., Carbonyl cyanide-p-trifluoromethoxyphenylhydrazone: FCCP) and particular protein/complex in the transporter chain (rotenone and antimycin A) have been detected in 76 structurally related clusters from the Tox21 10K library (Attene-Ramos et al., 2015; Xia et al., 2018). As potential mitochondrial toxicants, these compounds were found to cause significant reduction of the MMP using an MMP assay in HepG2 cells and rat hepatocytes (Attene-Ramos et al., 2015; Xia et al., 2018). Using transfer learning techniques and an unmodified version of the AlexNet network, the prediction model developed by the Deep Snap-DL method showed area under the ROC curve (AUC) value of 0.921 in the external validation, which included only 647 of the chemical structures employed previously by the Tox 21 Data Challenge 2014 (Uesawa, 2018). At the Tox 21 Data Challenge 2014 competition, the best AUC = 0.95 (Abdelaziz et al., 2016). The prediction performance (AUC = 0.921) by the Deep Snap-DL method is equal to top 10th in the Tox 21 Data Challenge 2014 competition (Tox21 Data Challenge., 2014; Uesawa, 2018. The result suggests that the DL approach based on Deep Snap is suitable for modeling to support toxicological assessments. However, further improvements are required for speed, automation, optimization, and efficiency. Despite the requirement for these improvements, herein, we examine the parameters for Deep Snap and DL to characterize how they affect the DNNs.

## Materials and Methods

### Data

Chemical substance profiles for cellular toxicity were collected from the publicly available Tox21 10K chemical library, 12,500 chemical substances, including pesticides, industrial, food-use, and drugs, procured from commercial sources screened by the Toxicology in the 21st Century (Tox21) program, a multi-agency collaboration between the U.S. Environmental Protection Agency, the National Institute of Environmental Health Sciences, National Toxicology Program, NIH Chemical Genomics Center, National Center for Advancing Translational Sciences, and the US Food and Drug Administration (1) incorporate advances in molecular systems by identifying patterns of chemical compounds-induced biological response, (2) prioritize compounds for more extensive toxicological evaluation, and (3) develop predictive models for biological response in human (NRC., 2007 Collins et al., 2008; Kavlock et al., 2009; Huang et al., 2011, 2014, 2016; Attene-Ramos et al., 2013; Tice et al., 2013; Chen et al., 2015; Hsieh et al., 2015, 2017; Merrick et al., 2015; Huang and Xia, 2017; Sipes et al., 2017). Their structures and the corresponding activities were used to determine agonist of a constitutive androstane receptor (CAR; NR1l3), which is a member of the ligand-activated superfamily of nuclear receptors transcriptionally activated genes predominantly expressed in the liver such as *CYP2B6* and *CYP3A4* involved in not only all phases of drug metabolism, transport, detoxification, and disposition about 50% of the drug metabolization potential in the body but also energy metabolism, tumor progression, cholesterol homeostasis, and glucose metabolism (Qatanani and Moore, 2005; Kobayashi et al., 2015; McMahon et al., 2018).

### Deep Snap Procedure: Creation of Molecular Image Files

**Figure 2:**
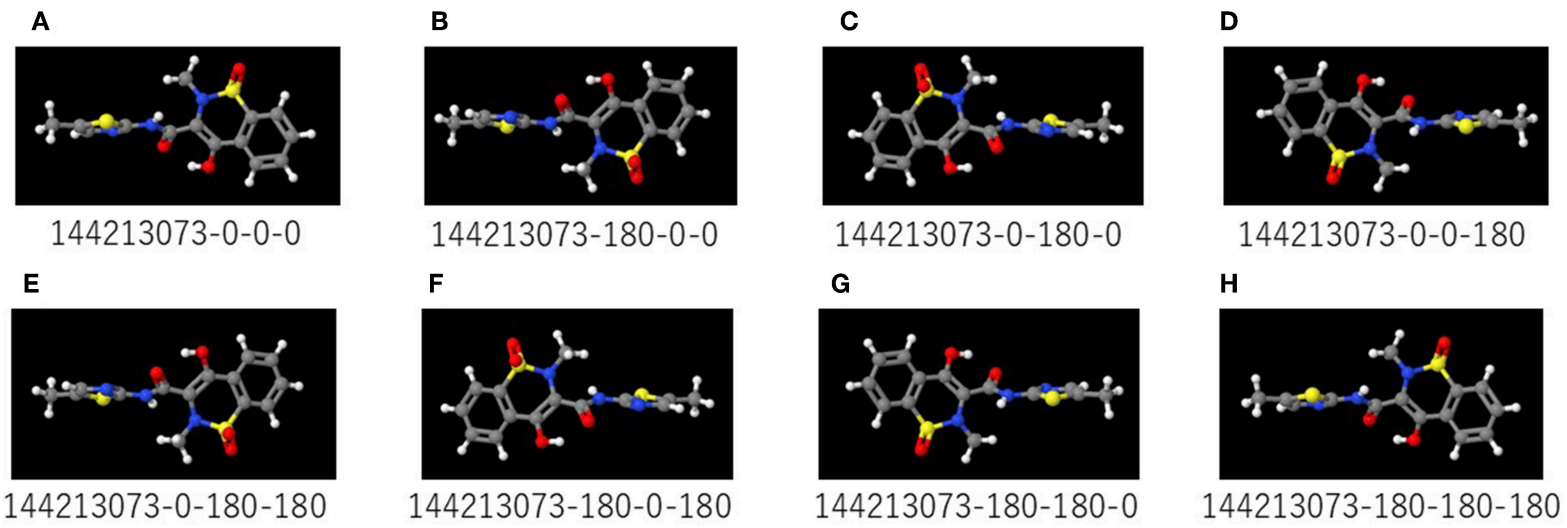
**(A–H)** are representative images captured by rotating the 3D structure in 180° increments on Deep Snap. The numbers below the images are the substance identification numbers (SID) provided in the PubChem database and increments of the viewing direction on the x-, y-, and z-axes. Red, yellow, blue, white, and gray colors in the molecular structures indicate the oxygen, sulfur, nitrogen, hydrogen, and carbon atoms, respectively.

A total of 9,667 of the chemical structures and the corresponding labeled activity scores were downloaded in the SMILES (Simplified molecular input line entry system) format (Weininger, 1988; Putz and Dudaş, 2013; Achary, 2014; Kumar and Chauhan, 2018) from the PubChem database (AID 1224892) derived from Tox21 10k library, the activity scores defined as the Pubchem_activity_scores (zero and scores between 1 and 100 were represented as inactive and active compounds, respectively, by cell viability and agonist activity screenings of the CAR signaling pathway). Then, by eliminating non-organic compounds, a total of 9,523 of the chemical compounds were selected (Table 1; Supplementary Table 1). After structure cleaning and standardization (removing salts, counterions, and fragments) by conformational import that is a high-throughput conformer generation method for large numbers of molecules using the MOE application software program (but no treatment of protonation states) (Chen and Foloppe, 2008; Molecular Operating Environment, Chemical Computing Group, Canada) (Supplementary Table 1), one 3D chemical structure per compound which have “rotatable torsions” was curated and optimized to generate a single low energy conformation using CORINA Classic software (Molecular Networks GmbH, Nürnberg, Germany, https://www.mn-am.com/products/corina) has been licensed in the past to predict 3D structures for some of the molecules in the main large public databases of small molecules such as PubChem a data-based commercial 3D molecular model builder with high accuracy and high speed for the 3D-structures of organic and metal-organic (also known as organometallic) molecules high coverage for nearly all organics but approximately half of the organometallics (Sadowski et al., 1994; Reitz et al., 2004; Tetko et al., 2005; Renner et al., 2006; Wang et al., 2009; Schwab, 2010; Andronico et al., 2011; Sayers et al., 2018; 3D Structure Generator CORINA Classic., 2019). Finally, these chemical structures were converted to the SDF file format. During the Deep Snap process, when the number of molecules described in the SDF file is large, the power required for the describing. Therefore, in order to improve the depiction speed, it is possible to multiple processes to be executed simultaneously by partitioning of the input data. The size of PNG file is different depending on the number of per SDF file. Moreover, the csv file including annotation data numbers, activity score, and dataset types that was divided randomly into training (4,761 chemicals), validation (2,381 chemicals), and testing (2,381 chemicals) datasets (Table 1; Supplementary Table 1) was used as the source for labeling each sample. Since the 3D-chemical structures can rotate 360° on each snapshots were captured at a range of fixed increments based on the SDF molecular structure file and the using a novel technique to capture generated images by their description function without human intervention saved as 256 × 256 (pixels resolution) PNG files (RGB) organized by their annotation data numbers (Figure 1). In this study, the 3D structure data was preliminarily portrayed as ball-and-stick structures in four types of increments on the x-, y-, and z-axes: first was (0,0,0), second was (0,0,0), (0,90,0), and (0,0,90), third was (0,0,0), (180,0,0), (0,180,0), and (0,0,180), fourth was (0,0,0), (180,0,0), (0,180,0), (0,0,180), (0,180,180), (180,0,180), (180,180,0), and (180,180,180) included 4 overlapped images automatically and manually obtained from the Deep Snap process, respectively to assess the systematic and suitable input of the 3D structures of chemical compounds and optimization Deep Snap (Figures 2A–H). The 3D ball-and-stick model with different colors to different atoms represented by which uses a unique algorithm to calculate surfaces (Jmol, Herráez, 2006; Cammer, 2007; Hanson, 2016; Scalfani et al., 2016; Hanson and Lu, 2017). More detailed technical information is available at the Jmol website^1^ As for the depiction process in Deep Snap, it is possible to design a setting cfg file that can specify arbitrary of the Jmol script such as image pixel size, image format (png or jpg), number of molecules per sdf file to split into (MPS), zoom factor (ZF, %), atom size for van der waals (AT, %), bond radius (BR) (mÅ), minimum bond distance (MBD), bond tolerance (BT), etc. Finally, using 64 pictures 105° angle and (MPS:100, ZF:100, AT:23, MBD:0.4, BT:0.8) as permutation test to assess non-specific activity score, they were randomly reassigned based on the activity scores without changing training, validation, and test datasets. Using a total of 10 different datasets, the prediction models were constructed by Deep Snap-DL method with the parameter values for the best performance optimized in this study eight pictures at 180° angle.

**Table 1:** Number of chemical compounds in train, validation, and test datasets used in optimization of parameter of Deep Snap.

**Activity score**	**Training**	**Validation**	**Test**	**Sum**
0: Non-toxic	3,651	1,858	1,878	7,387
1: Toxic	1,110	523	503	2,136
Sum	4,761	2,381	2,381	9,523

### Machine-Learning Models Based on DL

**Figure 3:**
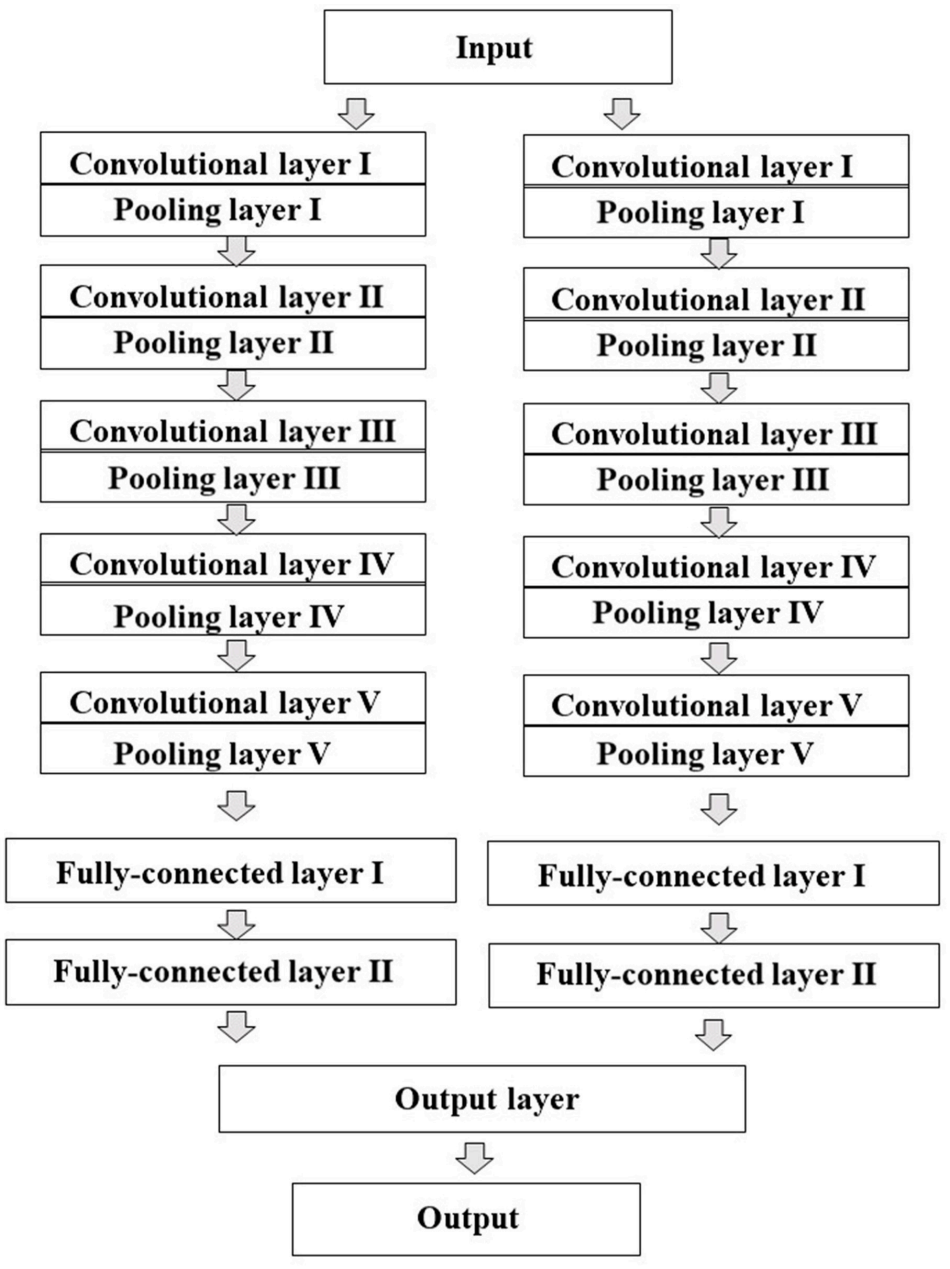
Schematic representation of the architecture of the convolutional neural network (CNN) model. AlexNet was used as transfer learning. The CNN contains total eight pre-learned layers five convolutional and pooling layers automatically extracted features from input pixel data and three fully-connected layers. The two juxtaposed convolutional and pooling layers are finally combined to the third fully-connected layers.

All the two-dimensional (2D) images contained digitized information data about plane configuration and the corresponded to the type of atom for the chemical structure produced by Deep Snap were resized by DIGITS version 4.0.0 software to a fixed resolution of 256 × 256 pixels and input into DL model to build the prediction models, which were trained based on the activity scores of chemical compounds and the corresponding 2D chemical-structure images. In this study, the total number of training epochs was 30, snapshot interval in epochs 1, validation interval in epochs 1, random seed 1, solver type stochastic gradient descent, base learning rate 0.01. Training, testing, and validation were performed using the dataset described in Table 1 and Supplementary Table 2. Finally, the performance of the prediction model was evaluated using one test dataset not used for validation. For the DL, a pre-trained implemented the open-source DL framework was used to build and train the DL models transfer learning (Jia et al., 2014). AlexNet is a convolutional neural network constructed by the University of Toronto (Krizhevsky et al., 2012). The fundamental architecture of this CNN constituted eight pre-trained layers, including five convolutional/pooling that convolution of feature volume and reduces layers by compressing images using max pooling compresses by selecting the maximum value in each region as a representative value convolutional/pooling layer I converts the previous volume (224 × 224 × 3) to (11 × 11 × 3) convolutional/pooling layer II converts the result of layer I to (5 × 5 × 48) convolutional/pooling layer III converts the result of layer II to (3 × 3 × 256) convolutional/pooling layer IV converts the result of layer III to (3 × 3 × 192) convolutional/pooling layer V converts the result of layer IV to (3 × 3 × 192) fully- connected layers that make final connections between feature values and force to zero to suppress overfitting (dropout) total 4,096 neurons. Since AlexNet has 60 million parameters, their optimization was essential to avoid overfitting (Figure 3; Krizhevsky et al., 2012; Szegedy et al., 2014; Cagli et al., 2017; Rawat and Wang, 2017; Aggarwal et al., 2018; Ha et al., 2018; Vakli et al., 2018). The non-saturating nonlinearity f (x) = max (0, x) as the function instead of such as sigmoid function f (x) = (1+e^−x^)^−1^ or f (x) = tanh (x) because the training time with gradient descent ReLUs much faster than that associated with if the input is negative, there is no contribution to other units (Nair and Hinton, 2010; Krizhevsky et al., 2012; Elfwing et al., 2018; Saha et al., 2018; Wang S. H. et al., 2018). Furthermore, adding a layer of local response normalization (LRN) between the pooling layer and the convolutional layer increases accuracy. The LRN is capable of handling a large number of CNNs with a large learning capacity that can be controlled by varying their assumptions about the nature of images that (1) the locality of pixel dependencies and (2) the stationarity of statistics.

The loss, which is a summation (not a percentage) of the errors in each dataset as shown below cross entropy error (CEE) with respect to the model's parameters by changing the weight vector values, in construction of the prediction models is calculated on training and validation datasets, where pi and yi correspond to the accuracy label (ground truth vector) and output of softmax (estimate values taken direct from the last layer output) for class *i*, respectively.

CEE= -Σ(pi)log(yi)

The loss value implies how well or poorly a certain model behaves after each iteration of optimization. Loss is indicative of unless the model has over-fitted with respect to the training data. The accuracy of the model is usually determined after the validation samples are fed to the model and the number of mistakes (zero-one loss) that the model makes recorded. The percentage of misclassification is calculated (Martinez and Stiefelhagen, 2018; Nguyen et al., 2018; Zhang and Sabuncu, 2018; Khened et al., 2019).

### Evaluation of the Predictive Models

In this method, it is possible to calculate the prediction result for each of a plurality of images prepared from the x-, y-, and z-axis directions with respect to one molecule. Therefore, the median of all these predicted values generated per molecule was used as a representative predicted value for each molecule. The metric was calculated on the basis of the predicted and the experimentally determined (true) labels, and the auroc (area under receiver operating characteristic) was calculated using JMP pro 14, statistical discovery software (SAS Institute Inc. NC) to evaluate the predictive models using 3D chemical structures including training (38,088 pictures), validation (19,048 pictures), and testing (19,048 pictures) datasets captured from eight increments on the x-, y-, and z-axes: (0,0,0), (180,0,0), (0,180,0), (0,0,180), (0,180,180), (180,0,180), (180,180,0), and (180,180,180) (Supplementary Table 2) (Linden, 2006). Sensitivity describes the true positive rate i.e., the proportion of actual positive samples that were correctly identified as positive for all positive samples including true and false positives.

Sensitivity = Σ True Positives / (Σ True Positives+Σ False Positives)

Specificity is the true negative rate i.e., the proportion of actual negative samples that were correctly identified as negative for all negative samples including true and false negatives.

$$\begin{array}{l} \text{Specificity }=\;\Sigma \text{ True Negatives/}(\Sigma \text{ True Negatives}\\ \;+\Sigma \text{FalsePositives}) \end{array}$$

### Random Forest

The file, including chemical structures as indicated by SMILES, chemical annotation numbers, activity scores, dataset classes divided into training and validation. Based on this information, the 3D chemical structures were built, descriptors were calculated using the MOE chemical calculation system. Using these descriptors, the prediction model was constructed using JMP pro 14.

## Results and Discussion

**Figure 4:**
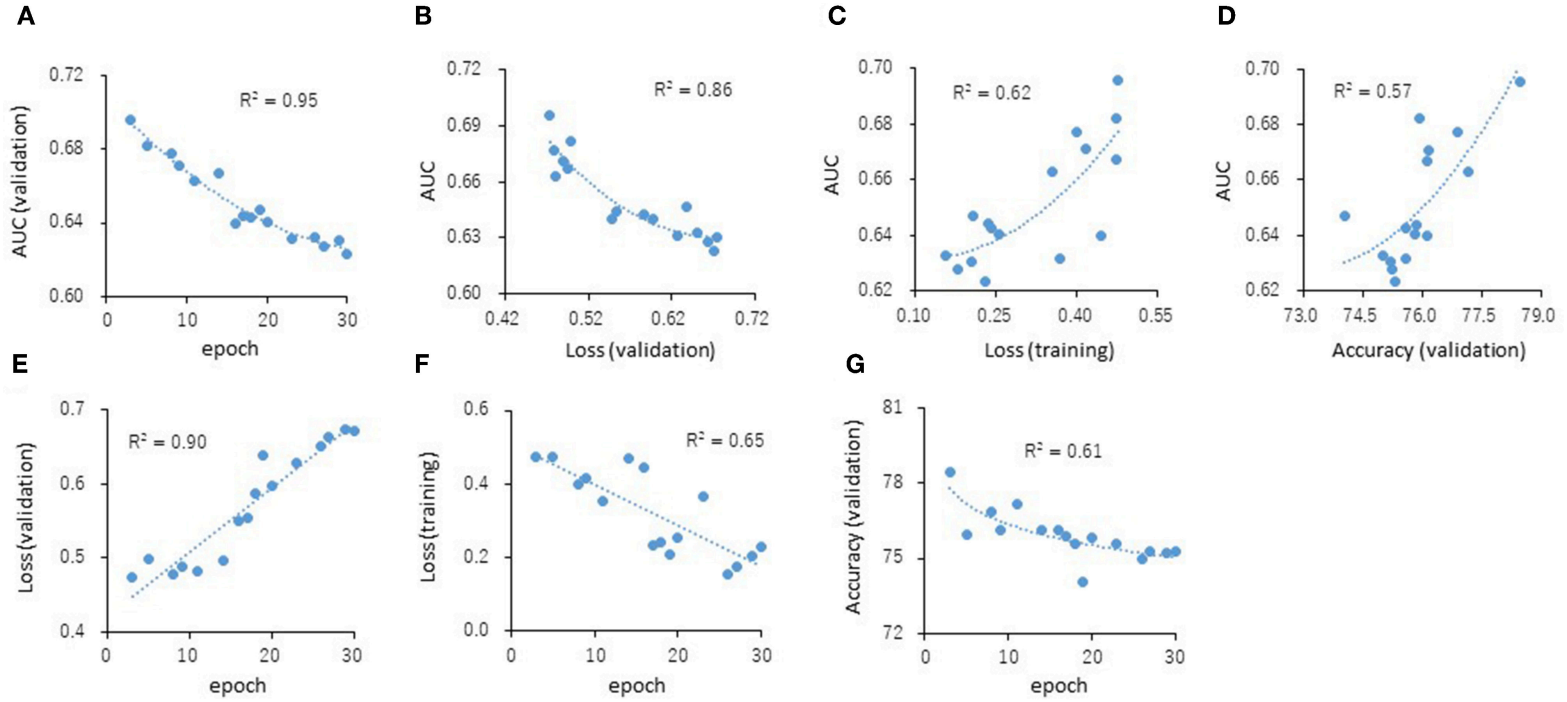
Correlations of the epochs **(A)**, validation loss **(B)**, training loss **(C)**, and validation accuracy **(D)** with the AUCs and the validation loss **(E)**, training loss **(F)**, and validation accuracy **(G)** with the epochs. The R^2^ values represent the correlation coefficients with two-dimensional equation representing the approximate fitted curve.

The predictive models for the presence or absence of activity as a CAR agonist and cell viability were built using the open-source Caffe in combination with the Deep Snap approach were applied to the training (38,088 pictures) and validation (19,048 pictures) datasets 180° angle (Supplementary Table 2). The testing dataset (19,048 pictures) was used to measure the performance of each prediction model (Supplementary Table 2). The AUC was calculated. The correlations (R^2^ values) of the AUC with each epoch were 0.95 (Figure 4A). The correlations (R^2^ values) were calculated from the testing datasets with validation loss (VL), training loss (TL), and validation accuracy (VA). VL is an error summation not a percentage obtained from how well the model is doing for. TL is an error summation which by attempting to determine good values for all the weights and biases (an empirical risk minimization). VA is the percentage of correct answers based on the results obtained from. As results, these R^2^ values with AUCs were 0.86 (VL), 0.62 (TL), and 0.57 (VA), respectively (Figures 4B–D). Moreover, the R^2^ values of the VL, TL, and VA each epochs were 0.90, 0.65, and 0.61, respectively (Figures 4E–G). These findings suggest that VL is the most important parameter of those considered here for evaluating the performance of a DL model.

**Figure 5:**
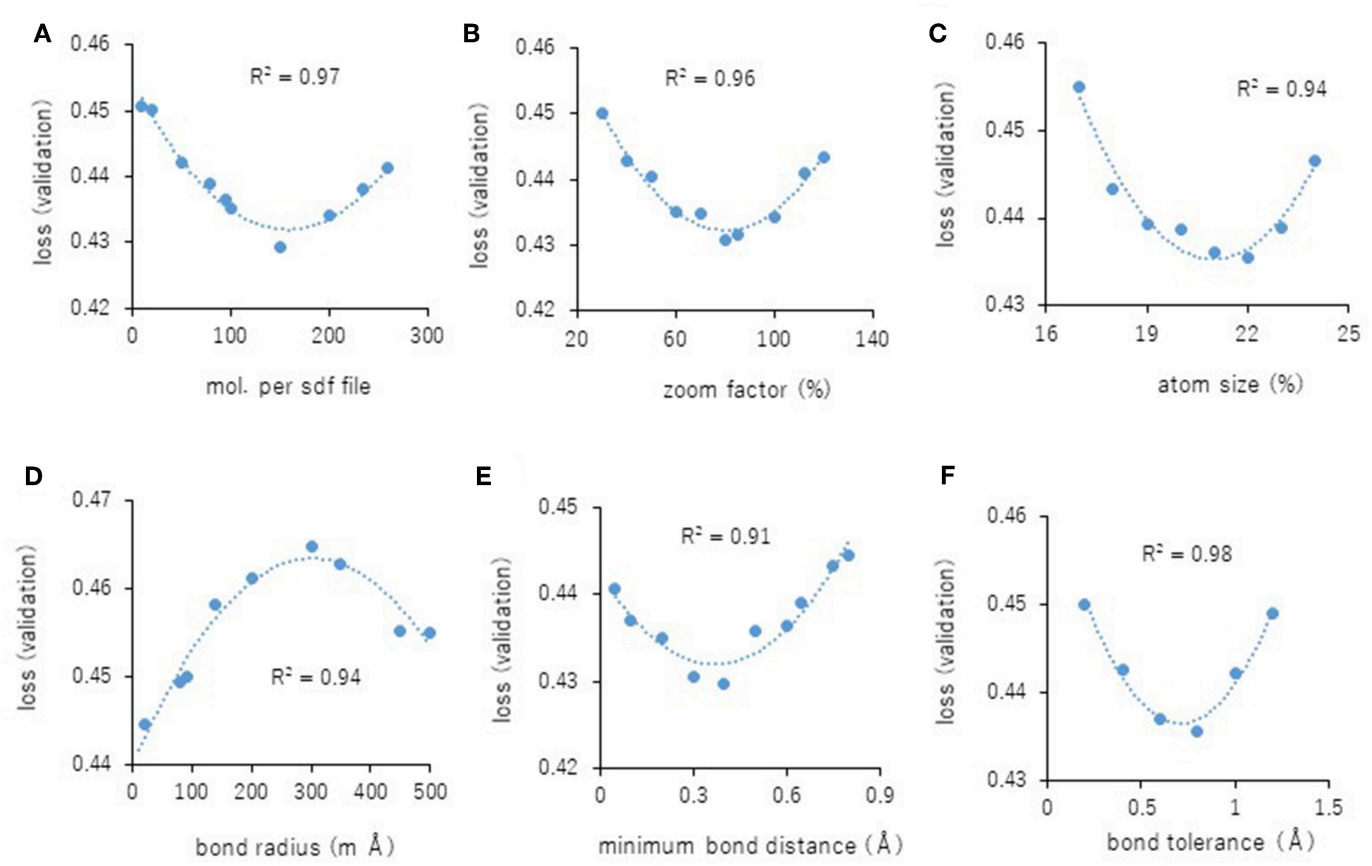
**(A–F)** displays parameterization of performance on Deep Snap. Correlation between the minimum VL of each epoch and the parameter values **(A)**: MPS, **(B)**: ZF, **(C)**: AS, **(D)**: BR, **(E)**: MBD, and **(F)**: BT for four images based on the 180° angle.

**Figure 6:**
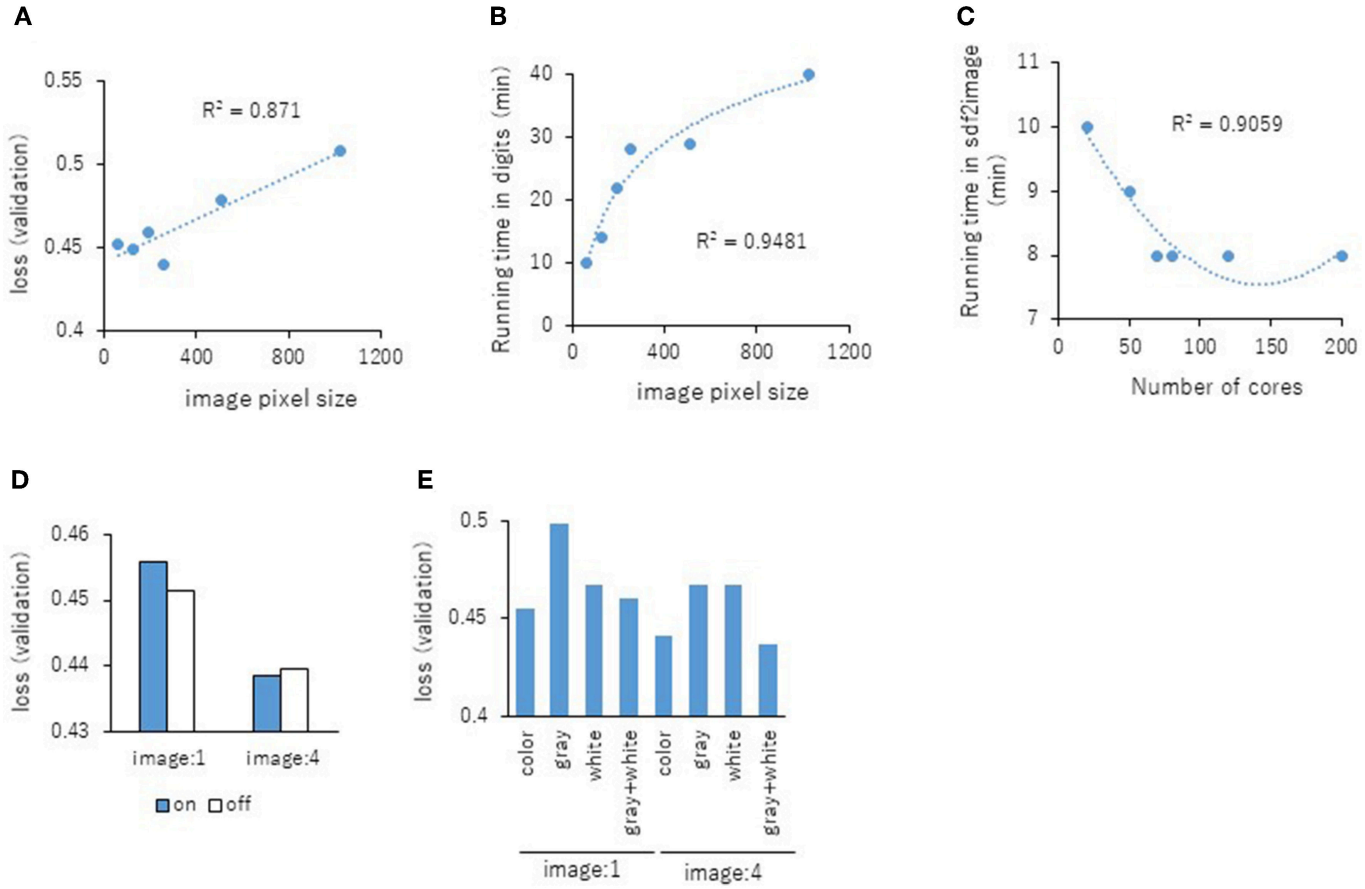
Relationship between the IPS and the minimum VL of each epochs **(A)** or RT in DL **(B)** using three pictures on the angle of 90° with R^2^ values between the IPS and the minimum VL or RT. **(C)** Influence of RT in three images with the number **(D)** The minimum VLs of space-filling (on; blue bar) and normal spacing (off; white bar) using one or four angles images. **(E)** The minimum VLs of multi-color, monotone-color (gray and white), and two-color (gray + white) using one or four angles images.

Next, the parameters for capturing Jmol-generated images on Deep Snap were optimized by assessing the DL models using the same procedure based on the VL using four pictures on the x-, y-, and z-axes: (0,0,0), (180,0,0), (0,180,0), and (0,0,180) in the training (19,044 pictures), validation (9,524 pictures), and test (9,524 pictures) datasets (Figures 2A–D and Supplementary Table 2). The following parameters were considered: (1) the number of molecules per SDF file: MPS, (2) the zoom factor: ZF, (3) the atom size for Van der Waals interactions: AT, (4) the bond radius: BR, (5) the minimum bond distance: MBD, and (6) the bond tolerance: BT. The parameter values (and corresponding minimum VL values) for the best model are as follows: (1) MPS: 150 (0.430), (2) ZF: 80% (0.431), (3) AT: 22% (0.435), (4) BR: 20 mÃ (0.425), (5) MBD: 0.4 Ã (0.430), and (6) BT: 0.8 Ã (0.436) (Figures 5A–F). In addition, the R^2^ values between these parameters and VLs were more than 0.90, and each of these relations followed quadratic function curves. Also, the R^2^ values of the running time (RT) in DL with the above six parameters showed that the RTs were moderately associated with AT (R^2^ = 0.48), BR (R^2^ = 0.47), and BT (R^2^ = 0.43) (Supplementary Figures 1C,D,F). However, MPS, ZF, and MBD showed no associations (Supplementary Figures 1A,B,E). Similarly, the image pixel size (IPS) was examined in the same way as the VL and RT in DL using three pictures on the x-, y-, and z-axes: (0,0,0), (0,90,0), and (0,0,90) in the training (14,283 pictures, 4,761 compounds), validation (7,143 pictures, 2,381 compounds), and test (7,143 pictures, 2,381 compounds) datasets (Supplementary Table 2). The IPSs (256×256) and (64×64) exhibited minimum VL (0.440) (Figure 6A) and minimum RT (10 min) (Figure 6B), respectively. Moreover, the number of cores in the multi-core CPU architecture showed the minimum RT (8 min) in the Jmol-generated images with 70 (Figure 6C). Also, we explored the effects of the minimum VL with space-filling, where the atoms are represented by spheres whose radii and center-to-center distances are proportional to the radii of the atoms and the distances between the atomic nuclei using one (0,0,0) or four (0,0,0), (180,0,0), (0,180,0), (0,0,180) image angles (Figures 2A–D) on the optimized parameters. When using one image, space-filling chemical structures into the image slightly increased the minimum VL (0.456) compared with that of normal spacing (0.452) (Figure 6D, left). However, there were no minimum VL changes between space-filling and normal spacing when using four image angles (Figure 6D, right). Furthermore, we compared the influence of the image color types of chemical structures with the minimum VL by using one or four image angles the optimized parameters, similarly. When the atomic colors of all the structures were changed to monotone (gray or white), these minimum VLs (0.468 or 0.467 for gray and white, respectively) increased to more than that of normal multi-color structures (0.442) using four image angles (Figure 6E, right). However, in the structures where the color of all atoms was changed to gray except for hydrogen (two-color: gray + white), the minimum VL (0.437) was decreased slightly compared with that of normal multi-color structures (0.442) using the four images (Figure 6E, right). When one angle image was used similarly, increased minimum VL of gray (0.499), white (0.468), or gray + white (0.460) was observed compared with that of normal multi-color (0.455) (Figure 6E, left). These findings suggest that optimal thresholds exist to attain the best performance with the prediction model. Finally, using the parameter values for the best performance model, AUCs were calculated using eight images of chemical structures captured at 180° increments on the x-, y-, and z-axes. As a result of optimization, the AUC exhibited 0.764 with minimum VL of 0.432. Furthermore, using 64 images at 105° angle and with default parameter values other than BR 15mÃ, the AUC increased into 0.791.

To assess (1) the suitableness of input as supervised data, (2) sufficient amount of images for training, and (3) adequate training for input dataset of pictures of chemical structure into the DL, the activity scores of the datasets, including training, validation, and test, were randomly assigned keeping the numbers of the three datasets unchanged as permutation test. The calculation of the performed each parameterized values of Deep Snap with each best performance model to capture chemical structures eight pictures at 180° angle using a total of ten different datasets with assignments of various activity scores. As result, the average AUCs were 0.553 (±0.007) with the average minimum VL of 0.522 (±0.014), indicated almost random guessing. These results suggest that the prediction models in this study extracted the CAR agonist activity-specific structural features from chemical compounds. Also, we calculated the AUC random forest as another method the same datasets for the above Deep Snap for CAR agonist and 206 of descriptors to build the prediction model in ROC-AUC value 0.749. Previously, we found that the prediction for the performance of compounds inducing MMP disruption was better 45° angles using 512 pictures for one molecule, with AUCs of 0.921 (Uesawa, 2018). Moreover, using 90° angle which 64 pictures for each, the performance of the prediction model indicated that the ROC-AUC value was 0.898 (Uesawa, 2018). In this study, we have used only 64 pictures based on 105° angle to avoid high computational cost. These results suggested that the prediction performance in the Deep Snap-DL method could be improved by input images due to more information about chemical structures. Also, as for the score activity of the CAR, the chemicals with scores other than 0 were defined as positive in order to secure enough input data in this study. However, in Tox21 program, the obvious activity for the CAR agonist is defined for chemicals with score of more than 40 (PubChem; https://pubchem.ncbi.nlm.nih.gov/#, AID 1224892). Therefore, it is necessary to optimize various types of assignments for the activity scores and/or other datasets in detail to further increase the prediction performance. In addition, a comparison of the performances between this state-of-the-art Deep Snap and 1,024 of extended-connectivity fingerprint (ECFG) of descriptors calculated from Dragon 7.0 (Kode srl., Pisa, Italy, Rogers and Hahn, 2010; Nikolic et al., 2012; Concu and Cordeiro, 2018; Uesawa, 2018). The prediction model constructed by DL in an H2O 3.2 package, where hidden layers, epochs, and best epochs were 200, 10, and 5, respectively (H20 ai, CA, USA, Chow, 2014) with ECFP showed that the ROC-AUC was 0.888 (Uesawa, 2018). In addition, the random forest in JMP pro 14, in which number of terms and maximum splits per tree were 500 and 256 for fingerprint, and 500 and 29 for 3D descriptors, respectively, predicted the models using the above ECFP descriptors or 3D descriptors with AUC of 0.901 or 0.907 (Uesawa, 2018). Until today, to improve the performance of prediction model, the selection of structural descriptors carried out using the skills and knowledge. Because it is difficult to perfectly preserve the original data, many of these descriptors are irreversible conversions. However, in the DL method using task-specific automatically extracted image information for molecular structures that do not require such high craftsmanship input data, it may demonstrate equal to or better than the above method using descriptors hand-engineered without prior knowledge or assumptions about the features.

When considering applying DL to a compound, whose molecular structure is a variable data format that can have branches and loops, there are problems with how to handle that input or output. To address this issue, graphic-based convolution, which has the ability to handle graph structures, simple encoding of the molecules (atoms, bonds, distances, etc.) represented by edge-connected nodes introducing convolution operations on each nodes non-Euclidean structure was proposed as modifications of DL architectures specialized for molecular fingerprints and models in the terms of structural features, physical properties, and activity (Duvenaud et al., 2015; Gilmer et al., 2017; Zhou and Li, 2017; Fernandez et al., 2018; Li C. et al., 2018). Since a chemical compound can also be represented as an undirected graphs of atoms when an atom is defined as a vertex (node) and a bond is defined as a side (edge), it is possible to construct a highly accurate prediction model by applying a convolution operation to the graph including their physical and chemical properties and extracting meaningful features from the large scale datasets of graph structure (Defferrard et al., 2016; Kipf and Welling, 2016). However, unlike image data, there drawback that a connection relation of peripheral nodes around the attention node of the graph is indefinite for each target node. To solve this difficulty with a heuristic or theoretical approach, graph convolution can be applied to graph Fourier transformation considering the adjacency of nodes by parameterizing weighted and undirected graphs without loops and multiple edges. Fourier conversion decomposes a waveform signal component by frequency component, but graph Fourier conversion decomposed a signal defined on a graph into “gentle signal” or “steep signal.” As for chemical structure, the graph signal converts into a graph spectral region assigning feature vectors to each atom in a chemical substance and their interaction between atoms. Thus, it is very well-adapted to prediction of local molecular structure-dependent physiological activity. In the case of definitions derived from the graph Fourier transform, for technical reasons, it needs to undirected and weighted graph without loops and multiple edges. On the other hand, by defining graph convolution more directly from only the connection relationship of nodes and edges, it is possible to introduce a more complicated structure such as a directed graph, multiple edges, and loops to graph convolution (Schlichtkrull et al., 2017). That is, for each node, its adjacent nodes are classified according to how they are connected, and then the sum (or average) of the signals of the neighboring nodes is taken for each neighborhood according to the manner of connection and according to how it is connected. However, since this method relied on edge and/or node information, the graph structures from the 3D conformational flexibility and the diversity of many features on the edge and/or node, such as shape, electrostatics, quantum effects, and other properties emerged from the molecular graph essential to clearly represent the biological systems and their relationship for the molecular activity and to consistently outperform other models (Kearnes et al., 2016). Additionally, since this graph structured format is heterogeneous among molecules, many learning algorithms how to process the complex graph effectively, except homogeneous input features. Therefore, to resolve issues, data transformings for the graph structure of the molecules to fix data size and format (Duvenaud et al., 2015; Liu K. et al., 2018). In addition, representations by the SMILES (Weininger, 1988; Putz and Dudaş, 2013; Achary, 2014; Jastrzebski et al., 2018; Kumar and Chauhan, 2018) do not encode bond lengths and mutual orientation of atom in space, meaning that they lack information for the molecular conformations, such as 3D atomic arrangements and some molecule stereoisomers.

Also, 3D-CNN, convolutional layers extended to 3D filter that move 3-directions (x, y, z) extract spatiotemporal features from moving objects proposed as a method applied to motion image recognition (Ji et al., 2013; Blendowski and Heinrich, 2018; Lu et al., 2018). It has been successfully used to extract against the temporal change of the spatial structure data as a feature expression of 3D volume space such as cuboid output using the node locally connected to all the images within a certain time width (Ji et al., 2013; Maturana and Scherer, 2015). In this method, although the temporal change such as event detection in videos, 3D images etc. is considered in the extracted feature, it depends on the size in the time direction of the filter. Therefore, when recognizing an operation longer than the filter size, selection and combination processing of those features must be performed. As for chemical compounds, the 3D-CNN has been successfully shown to able to handle the data with spatial structure such as 3D-structures, on the choice of the data representation (Ji et al., 2013; Maturana and Scherer, 2015; Blendowski and Heinrich, 2018; Kuzminykh et al., 2018). If a suitable representation used, the most critical information efficiently captured. In addition, the chemical compounds induced conformational changes target interactions is possible to a number of conformations or orientations (Tuffery and Derreumaux, 2017; Salmaso and Moro, 2018). Furthermore, the conformational changes of target proteins by ligands and protein-ligands interactions have been studied computational (Yang et al., 2016; Hollingsworth and Dror, 2018; Nusrat and Khan, 2018). Therefore, the 3D-CNN could be a very useful method for extracting structural features based on molecular dynamics, which the dynamic behavior of molecular system as a function of time. However, since a data in non-euclidean spaces, such as spherical data is difficult to trivially apply for direct 3D representation, the suitable conditions such as scaling and required number of input samples have not been cleared completely, which leads to poor performance by sparsity and redundancy in the data and increased complexity in the convolution process (Ji et al., 2013; Maturana and Scherer, 2015; Blendowski and Heinrich, 2018; Kuzminykh et al., 2018). In additions, 3D-CNNs requires more 3D matrix and more calculations than 2D. Thus, the scaling for the CNNs to 3D representations is not straightforward due to the sparsity in input data and the complexity in the convolution operations (Ji et al., 2013; Maturana and Scherer, 2015; Blendowski and Heinrich, 2018; Kuzminykh et al., 2018). Therefore, even now, 3D-CNN need shape descriptors by hand, such as light field descriptors (Pu and Ramani, 2006), mesh DOG (Zaharescu et al., 2009), spin images (Johnson and Hebert, 1999), heat kernel signatures (Xiang et al., 2014), and spherical harmonics high performance (Kazhdan et al., 2003). To alleviate this problem, although Gaussian blur representation was proposed to reduce the sparsity and the redundancy of input, convolving with the Gaussian kernel leads to information loss (Kuzminykh et al., 2018).

Previously, it was ascertained that the Deep Snap-DL method yields the corresponding predicted values for different physiological activities between optical R/S isomers (Uesawa, 2018). This report indicated that Deep Snap-DL accurately extract physiological activities depending on molecular conformation-specificity optimization for various conformations is necessary to maintain high performance of the prediction model. In this research, to define the steric conformation of the molecular structure, CORINA Classic software was used. However, if more suitable definition of 3D steric structures of chemical compounds directly or indirectly related to biological activity, mechanisms, and molecular pathways such as determination of 3D structure for a protein receptor with apparent ligand affinity pocket were established based on the molecular dynamics stimulation, the Deep Snap-DL procedure would be outperformed.

On the other hand, there are some problems that need to be improved so far in this Deep Snap-DL method. At first, in principle, this strategy to capture more detail and greater amount of information chemical structures using more molecular images from 3D-rotation (Uesawa, 2018). In supervised learning, output data corresponding to input data can be obtained, but learning is performed for the purpose of minimizing the error by comparing the output to new data. Therefore, the correction of misclassification for a large amount of labeled input data is difficult. If the classification criteria within image data could be clarified using proposed visual explanations technique (Simonyan et al., 2013; Mahendran and Vedaldi, 2014; Selvaraju et al., 2016; Smilkov et al., 2017; Zhen et al., 2017; Philbrick et al., 2018), it may be useful for estimation of 3D structure important for physiological activity of the compound and would more reduction of calculation cost by reducing the number of images used. Furthermore, by parameters for Deep Snap in this study, the calculation time was reduced the relatively high performance of the prediction model for the CAR agonist activity. In agreement with previous report although DL able to accurately predict for a molecule with just close neighbors in the training dataset, a hitherto unexamined chemical was predicted close to the average of all training molecule activities, which the lack of ability to learn beyond the training dataset (Liu R. et al., 2018). Deep Snap-DL method indicated the performances of prediction models depending on input datasets produced by various conditions including bonds, spacing, angles, colors, atom size, etc. Moreover, the AUCs were reduced by random permutation of the activity scores of datasets consisting training, validations, and test as non-endpoint activity. These findings suggested that the task-specific improvement of Deep Snap-DL technique by adjustments of input data with the representations of chemical structure such as bonds, space, atom size etc. could be more available approach than conventional methods. Taken together, by combining the Deep Snap strategy with parts of graph-CNN or 3D-CNN functions. Overall, the novel approach Deep Snap not only would fill a gap between chemical structure and toxicological prediction, but also may be useful for constructing an *in silico* prediction model of appropriate chemical risk assessment replace.

In summary, the relations of the parameters of Deep Snap such as (1) number of molecules per SDF files split into (2) zoom factor percentage, (3) atom size for van der waals percentage, (4) bond radius, (5) minimum bond distance, and (6) bond tolerance with the VLs as indicator for evaluating the performance of the DL following quadratic function curves, suggesting that optimal thresholds exist to attain the best performance with these prediction models. Using the parameter values the best performance with the prediction model, the prediction model for CAR agonist was built using 64 images at 105° angle AUCs of 0.791. The results of this study feature the possible power of novel DL-based QSAR approach for prediction of potential toxicity of large datasets of any chemical compounds.

## Author Contributions

YU initiated and supervised the work, designed the experiments, collected the information about chemical compounds, and edited the manuscript. YM drafted the manuscript. YU and YM read and approved the final manuscript.

### Conflict of Interest Statement

The authors declare that the research was conducted in the absence of any commercial or financial relationships that could be construed as a potential conflict of interest.

## Footnotes

^1^Jmol: An Open-Source Java Viewer for Chemical Structures in 3D. Available online at: http://www.jmol.org/
