# Supplementary material for: Optimization of a Deep-Learning Method Based on the Classification of Images Generated by Parameterized Deep Snap a Novel Molecular-Image-Input Technique for Quantitative Structure–Activity Relationship (QSAR) Analysis
Source: Front Bioeng Biotechnol. 2019 Mar 28;7:65. doi: 10.3389/fbioe.2019.00065 (PMC6447703; doi:10.3389/fbioe.2019.00065)
Supplement: Supplementary file 1 [file Data_Sheet_1.PDF]

| Supplementary table 1. Structures and activities of chemical compounds measured by a CAR agonist-mode assay from the Tox21 10k library | | | |
|---|---|---|---|
| SID | chemical structures | activity score | dataset class |
| 251919990 | C1=CC=C(C=C1)COC2=CC=C(C=C2)S(=O)(=O)C3=CC=CC=C3O | 0 | train |
| 251919989 | CC(C)(C1=CC(=C(C=C1)O)C2=CC=CC=C2)C3=CC(=C(C=C3)O)C4=CC=CC=C4 | 1 | validation |
| 251919988 | CCCC(C(CO)O)O | 0 | test |
| 251919987 | CCC1=C(C(=CC=C1)CC)N(COC)C(=O)CS(=O)(=O)[O-].[Na+] | 0 | train |
| 251919986 | C[As+](C)(C)CC(=O)[O-] | 0 | train |
| 251919985 | C1=C(C(=CC(=C1Cl)Cl)Cl)C2=C(C(=CC(=C2Cl)Cl)Cl)Cl | 0 | validation |
| 251919984 | CCC1=C(C(=CC=C1)CC)NC(=O)C(=O)[O-].[Na+] | 0 | test |
| 251919983 | CC(C)(C)C1=NNC(=NC1=O)SC | 0 | train |
| 251919982 | C1=CC=C(C=C1)C(CBr)Br | 0 | train |
| 251919981 | CC1=C(C(=O)OC2=C1C=CC(=C2)O)Cl | 1 | validation |
| 251919980 | CC1=CC2=C(C=C1)SC3=CC=CC(=C32)C | 1 | test |
| 251919979 | CC1=CC[C@H](CC1)C(C)(C)O | 0 | train |
| 251919978 | C1=COC(=C1)CS | 1 | train |
| 251919977 | CC(C)C(=O)OCCC(C)CCC=C(C)C | 0 | validation |
| 251919976 | CN1C=CC=C1 | 0 | test |
| 251919975 | C1=CC=C(C(=C1)O)Br | 0 | train |
| 251919974 | CC(C=O)C1=CC=CC=C1 | 0 | train |
| 251919973 | CC=CC1=CC(=C(C=C1)OC)OC | 0 | validation |
| 251919972 | COC1=CC(=C(C=C1)OC)C=O | 0 | test |
| 251919971 | CC(=O)C1=CC=C(C=C1)C2=CC=CC=C2 | 1 | train |
| 251919970 | CCN(CC)C1=CC=CC(=C1)C | 0 | train |
| 251919969 | COC1=C(C(=CC=C1)OC)O | 0 | validation |
| 251919968 | C1=CC(=C(C=C1Cl)Cl)C(=O)Cl | 0 | test |
| 251919967 | C1=CC(=C(C=C1Cl)[N+](=O)[O-])Cl | 0 | train |
| 251919966 | CCN(CC)C(=O)Cl | 0 | train |
| 251919965 | CCOC(=O)[C@@H]([C@H](C(=O)OCC)O)O | 0 | validation |
| 251919964 | COC1=CC=C(C=C1)C#N | 0 | test |
| 251919963 | CC1=CC=C(C=C1)C(=O)Cl | 0 | train |
| 251919962 | C1C2=CC=CC=C2C(=O)O1 | 0 | train |
| 251919961 | C1=CC=C(C(=C1)C#N)Cl | 0 | validation |
| 251919960 | CCOC=C(C(=O)OCC)C(=O)OCC | 0 | test |
| 251919959 | C1=CC2=C(C=CN=C2C=C1Cl)Cl | 0 | train |
| 251919958 | CCC(C)C(=O)OC | 0 | train |
| 251919957 | CCN1C2=CC=CC=C2C3=CC=CC=C31 | 1 | validation |
| 251919956 | C#CC1=C(C(=O)C(=C(C1=O)Cl)Cl)C#C | 0 | test |
| 251919955 | C1=CC2=C(C=CC=C2O)C(=C1)O | 1 | train |
| 251919954 | C1=C(C=C(C(=C1Br)N)Br)[N+](=O)[O-] | 0 | train |
| 251919953 | C1=CC=C2C(=C1)C(=O)C3=C(C2=O)C(=CC=C3)Cl | 1 | validation |
| 251919952 | C(CCl)NCCCl.Cl | 0 | test |
| 251919951 | C1=CC2=C3C(=CC=C4C3=C1C(=O)OC4=O)C(=O)OC2=O | 0 | train |
| 251919950 | CC1=CC=C(C=C1)S(=O)(=O)OC | 0 | train |
| 251919949 | CC(C)C(=O)Cl | 0 | validation |
| 251919948 | CC(C)CBr | 0 | test |
| 251919947 | CCO[Si](C)(C)OCC | 0 | train |
| 251919946 | CCCCOP(=O)(CCCC)OCCCC | 1 | train |
| 251919945 | COC1=C(C=CC(=C1)C=C)O | 0 | validation |
| 251919944 | C1=CC=C(C=C1)C(C2=CC=CC=C2)(C3=CC=CC=C3)Cl | 1 | test |
| 251919943 | C1=CC(=CC=C1C#N)O | 0 | train |
| 251919942 | CC(=C)CCO | 0 | train |
| 251919941 | CCCCC(=C)C | 0 | validation |
| 251919940 | CC1=CC(=C(C(=C1)C)C(=O)P(=O)(C2=CC=CC=C2)C3=CC=CC=C3)C | 1 | test |
| 251919939 | C1=CC=C(C=C1)C(=O)C2=C(C=CC(=C2)Cl)N | 1 | train |
| 251919938 | C1=CC=C(C=C1)C2=NC3=CC=CC=C3N2 | 1 | train |
| 251919937 | CC1=CP(=O)(CC1)C2=CC=CC=C2 | 0 | validation |
| 251919936 | C1=CC=C(C=C1)OC2=CC=C(C=C2)Cl | 0 | test |
| 251919935 | CC1=CNC(=O)NC1=O | 0 | train |
| 251919934 | CC(C)C1=CC=CC=C1N | 0 | train |
| 251919933 | C1=CC(=C(C=C1N)[N+](=O)[O-])Cl | 0 | validation |
| 251919932 | CCCCOCC | 0 | test |
| 251919931 | C1=CNC=CC1=O | 0 | train |

| | | | |
|---|---|---|---|
| 251919930 | C1=CC(=CN=C1)Cl | 0 | train |
| 251919929 | CC1=CC(=O)NC(=O)N1 | 0 | validation |
| 251919928 | C1=C(C=C(C=C1Br)Br)Br | 0 | test |
| 251919927 | CCC=C(C)C=O | 0 | train |
| 251919926 | CCOC(=O)CN.Cl | 0 | train |
| 251919925 | CC(=O)OC1CCCCC1 | 0 | validation |
| 251919924 | C1=CC(=CC=C1CO)[N+](=O)[O-] | 0 | test |
| 251919923 | C1=COC(=C1)CN | 0 | train |
| 251919922 | CCOC(C(=O)C1=CC=CC=C1)OCC | 0 | train |
| 251919921 | CN1C=CN=C1 | 0 | validation |
| 251919920 | C1=CC=C2C(=C1)N=C(S2)Cl | 0 | test |
| 251919919 | C1C(=O)CC2=CC=CC=C21 | 1 | train |
| 251919918 | C1=CC=C(C(=C1)CCl)CCl | 1 | train |
| 251919917 | CC=CCO | 0 | validation |
| 251919916 | CN1C=NC2=C1C(=O)N(C(=O)N2)C | 0 | test |
| 251919915 | C1=CC=C(C(=C1)C(=O)N)Cl | 0 | train |
| 251919914 | C1=CC=C(C=C1)[Sb](C2=CC=CC=C2)C3=CC=CC=C3 | 1 | train |
| 251919913 | CCCC(=O)OC(OC(=O)CCC)OC(=O)CCC | 0 | validation |
| 251919912 | CC1=CC(=CN=C1)C | 0 | test |
| 251919911 | C[C@@]12CC[C@@H](C1(C)C)C[C@H]2OC(=O)C=C | 0 | train |
| 251919910 | CC(=O)O[C@@H]1C[C@@H]2CC[C@]1(C2(C)C)C | 0 | train |
| 251919909 | CC(=O)C1=CC=CC=C1N | 0 | validation |
| 251919908 | CC(C)CCOCCC(C)C | 0 | test |
| 251919907 | C1=CC(=CC=C1O)I | 0 | train |
| 251919906 | C1CC2=CC=CC=C2C1 | 0 | train |
| 251919905 | CC1=CC(=C(C(=C1)C)C=O)C | 0 | validation |
| 251919904 | [B].C1COCCN1 | 0 | test |
| 251919903 | CCOCOCC | 0 | train |
| 251919902 | CN(C)CCCl.Cl | 0 | train |
| 251919901 | C1=CC(=C(N=C1)N)N | 1 | validation |
| 251919900 | CC(CC(C)(C)C)CC(C)(C)CC(C)(C)C | 0 | test |
| 251919899 | COCCC(=O)OC | 0 | train |
| 251919898 | C1COCCN1CCCl.Cl | 0 | train |
| 251919897 | C1=CC(=C(C=C1N)[N+](=O)[O-])F | 0 | validation |
| 251919896 | C[Si-](C)[O+]=[Si](C)C | 0 | test |
| 251919895 | CCN(CCC#N)C1=CC=C(C=C1)N=NC2=CC=C(C=C2)[N+](=O)[O-] | 1 | train |
| 251919894 | CC1=C(C=CC(=C1)O)SC | 0 | train |
| 251919893 | CN(C)C(=O)C=C | 0 | validation |
| 251919892 | C1=CC=C(C=C1)C(=O)C(Cl)Cl | 1 | test |
| 251919891 | C1=CC(=CC(=C1)OC2=CC=C(C=C2)N)OC3=CC=C(C=C3)N | 1 | train |
| 251919890 | CC(C)CC1=NC=CN=C1OC | 0 | train |
| 251919889 | CCN(CC)CCOC(=O)C=C | 0 | validation |
| 251919888 | C1=CC(=NC(=C1)Cl)Cl | 0 | test |
| 251919887 | C/C=C/C(=C)C1=C(CCCC1(C)C)C | 0 | train |
| 251919886 | C/C(=C/C(=[OH+])C)/[O-].C/C(=C/C(=[OH+])C)/[O-].C/C(=C/C(=[OH+])C)/[O-].[Cr] | 0 | train |
| 251919885 | C=CCOC(=O)N | 0 | validation |
| 251919884 | O.O.O.O.O.O.Cl[Sc](Cl)Cl | 0 | test |
| 251919883 | C(CO)CS | 0 | train |
| 251919882 | CC(C)(C)C1=CC=C(C=C1)OC2CCCCC2O | 1 | train |
| 251919881 | C1=CC=C(C(=C1)C#N)N | 0 | validation |
| 251919880 | CN(C)C1=CC=C(C=C1)C(=C2C=CC(=[N+](C)C)C=C2)C3=CC=CC=C3.C(=O)(C(=O)[O-])O | 1 | test |
| 251919879 | C1=CNC(=O)C(=C1)O | 0 | train |
| 251919878 | CCC1CCCCC1 | 0 | train |
| 251919877 | C[C@]12CC[C@@H](C[C@H]1CC[C@@H]3[C@@H]2C[C@H]([C@]4([C@@]3(CC[C@@H]4C5=CC(=O)OC5)O)C)O)O | 0 | validation |
| 251919876 | CC1CCOC(C1)C=C(C)C | 0 | test |
| 251919875 | CCOC(=O)OC(=O)OCC | 0 | train |
| 251919874 | CC(CCC=C(C)C)O | 0 | train |
| 251919873 | O.O.O.O.O.O.Cl[Dy](Cl)Cl | 0 | validation |
| 251919872 | C1CC1C=O | 0 | test |
| 251919871 | C1CC2=C(C1)C=C(C=C2)O | 0 | train |
| 251919870 | C1=CC(=CC=C1N)S(=O)(=O)[N-]C2=NC=CS2.[Na+] | 0 | train |

| | | | |
|---|---|---|---|
| 251919869 | C1=CC(=O)NC=C1 | 0 | validation |
| 251919868 | C1=CC(=CC=C1CC#N)Cl | 0 | test |
| 251919867 | C/C(=C/C(=[OH+])C)/[O-].C/C(=C/C(=[OH+])C)/[O-].C/C(=C/C(=[OH+])C)/[O-].[Fe] | 0 | train |
| 251919866 | C/C(=C/C(=[OH+])C)/[O-].C/C(=C/C(=[OH+])C)/[O-].C/C(=C/C(=[OH+])C)/[O-].[Al] | 0 | train |
| 251919865 | O.O.O.O.O.O.Cl[Eu](Cl)Cl | 0 | validation |
| 251919864 | COC1=CC=CC=C1C=O | 0 | test |
| 251919863 | C1=CC=C(C=C1)C(=O)C2=CC=C(C=C2)Cl | 1 | train |
| 251919862 | CN1C(=S)N=NN1 | 0 | train |
| 251919861 | CC(C)(C1=CC=C(C=C1)OC2=CC=C(C=C2)N)C3=CC=C(C=C3)OC4=CC=C(C=C4)N | 1 | validation |
| 251919860 | C(COC(=O)Cl)OC(=O)Cl | 0 | test |
| 251919859 | C1CC(=O)NC1=O | 0 | train |
| 251919858 | C1=CC(=CC=C1C(=O)Cl)Cl | 0 | train |
| 251919857 | CC1=CC(=CC=C1)N(C)C | 0 | validation |
| 251919856 | C[C@@]12CC[C@@H]3[C@H](CC3(C)C)C(=C)CC[C@H]1O2 | 0 | test |
| 251919855 | CCCCCCCCCCBr | 0 | train |
| 251919854 | CC1=NC=C(C=C1)O | 0 | train |
| 251919853 | CCCCOC=C | 0 | validation |
| 251919852 | CCCCCCS | 0 | test |
| 251919851 | C=COCCCl | 0 | train |
| 251919850 | C1=CC=C(C=C1)C[P+](C2=CC=CC=C2)(C3=CC=CC=C3)C4=CC=CC=C4.[Cl-] | 0 | train |
| 251919849 | C1=CC=NC(=C1)Br | 0 | validation |
| 251919848 | CN1CN(CN(C1)C)C | 0 | test |
| 251919847 | CC1=NC(=CC=C1)C | 0 | train |
| 251919846 | CN1CCN(CC1)C | 0 | train |
| 251919845 | CCCCCCCC(=O)OCC | 0 | validation |
| 251919844 | CCC(=O)OC/C=C(¥C)/CCC=C(C)C | 0 | test |
| 251919843 | CCCC(C#C)O | 0 | train |
| 251919842 | CC1=CC=C(C=C1)CCl | 0 | train |
| 251919841 | CCCCCCCCCCC1=CC=CC=C1 | 0 | validation |
| 251919840 | CC(=N[Si](C)(C)C)O[Si](C)(C)C | 0 | test |
| 251919839 | CCCCOP(OCCCC)OCCCC | 1 | train |
| 251919838 | C=CC1=CC=NC=C1 | 0 | train |
| 251919837 | COC1=C(C=C2C(=C1)C(=NC=N2)NC3=CC(=CC=C3)Cl)OC | 0 | validation |
| 251919836 | C1=CC(=NC=C1C(=O)N)N | 1 | test |
| 251919835 | CC(C1=CC=CC=C1)(C2=CC=C(C=C2)O)C3=CC=C(C=C3)O | 1 | train |
| 251919834 | CC1=CC=C(C=C1)C2OC[C@H]3[C@@H](O2)[C@H](OC(O3)C4=CC=C(C=C4)C)[C@@H](CO)O | 0 | train |
| 251919833 | CCC1=CC=CC(=C1N(C(C)COC)C(=O)C(=O)O)C | 0 | validation |
| 251919832 | C1CCC(CC1)C2=C(C=CC(=C2)C3(CCCCC3)C4=CC(=C(C=C4)O)C5CCCCC5)O | 1 | test |
| 251919831 | C1=CC=C(C=C1)C2=CC=CC=C2C3=CC=CC=C3 | 1 | train |
| 251919830 | CCC1=CC=CC=C1C | 0 | train |
| 251919829 | C1CN(CCN1)C2=C(C=C3C(=C2)N(C=C(C3=O)C(=O)O)C4=CC=C(C=C4)F)F | 0 | validation |
| 251919828 | COC(=O)C=CC1=CC=CC=C1 | 0 | test |
| 251919827 | CC(C1=CC=C(C=C1)O)C2=CC=C(C=C2)O | 1 | train |
| 251919826 | CC1=C(C(=CC=C1)C)C | 0 | train |
| 251919825 | C1CCSC1 | 0 | validation |
| 251919824 | CC1CCCCC1 | 0 | test |
| 251919823 | CCOC1=C(C=C(C=C1)C=CC)O | 0 | train |
| 251919822 | CC1=CCC(=O)O1 | 0 | train |
| 251919821 | CC=CC(=O)OCC(C)C | 0 | validation |
| 251919820 | CC1=NC=CN=C1 | 0 | test |
| 251919819 | CCC(=O)OCC=CC1=CC=CC=C1 | 0 | train |
| 251919818 | CC(C)C(=O)OCC1=CC=CC=C1 | 0 | train |
| 251919817 | CCCCCC=CC=CC=C | 0 | validation |
| 251919816 | CCCCCCCC(OC)OC | 0 | test |
| 251919815 | CCCCN(C)C | 0 | train |
| 251919814 | CC(C)(C=C)O | 0 | train |
| 251919813 | CC1=C(C=C(C=C1)C(C)C)OC | 0 | validation |
| 251919812 | CC(=CCCC(=CCCC(=CCOC(=O)C)C)C)C | 0 | test |
| 251919811 | CC(=CCC/C(=C/CCC(=C)C=C)/C)C | 0 | train |
| 251919810 | CCC(C)CC(=O)CC | 0 | train |

| | | | |
|---|---|---|---|
| 251919809 | CC(C)C1=C(C(=C(C(=N1)C(C)C)/C=C/[C@H](C[C@H](CC(=O)[O-])O)O)C2=CC=C(C=C2)F)COC.[Na+] | 0 | validation |
| 251919808 | C1CC1C2=NC3=CC=CC=C3C(=C2/C=C/[C@H](C[C@H](CC(=O)[O-])O)O)C4=CC=C(C=C4)F.C1CC1C2=NC3=CC=CC=C3C(=C2/C=C/C(C[C@H](CC(=O)[O-])O)O)C4=CC=C(C=C4)F.[Ca+2] | 0 | test |
| 251919807 | C=CC(=O)OCCOC1C2CCC1C3C2CC=C3 | 0 | train |
| 251919806 | CN(C)CCN1CCOCC1 | 0 | train |
| 251919805 | CC(C)(C)C1=CC(=O)C(=CC1=O)C(C)(C)C | 1 | validation |
| 251919804 | CCC(C)(OOC(C)(C)C)OOC(C)(C)C | 0 | test |
| 251919803 | CO[Si](C1=CC=CC=C1)(C2=CC=CC=C2)OC | 0 | train |
| 251919802 | C[N+](C)(C)CCCCCC[N+](C)(C)C.[Cl-].[Cl-] | 0 | train |
| 251919801 | CCC(C)C1=C(C(=CC(=C1)C(C)(C)C)N2N=C3C=CC=CC3=N2)O | 1 | validation |
| 251919800 | C1=CC2=C(C=CC(=C2S(=O)(=O)[O-])N)C(=C1)S(=O)(=O)O.[Na+] | 0 | test |
| 251919799 | CCCCNC1=C2C(=C(C=C1)NCCCC)C(=O)C3=CC=CC=C3C2=O | 0 | train |
| 251919798 | CC[Hg]N1C(=O)C2C(C1=O)C3(C(=C(C2(C3(Cl)Cl)Cl)Cl)Cl)Cl | 1 | train |
| 251919797 | CCCCCCCCCCCCS(=O)(=O)Cl | 0 | validation |
| 251919796 | CC(=NNC(=S)N)C | 1 | test |
| 251919795 | C[C@@H](CCC=C(C)C)CC=O | 0 | train |
| 251919794 | CC1CCC(C(C1)O)C(=C)C | 0 | train |
| 251919793 | CO[Si](CCCNCCNCCN)(OC)OC | 0 | validation |
| 251919792 | C(CO)C(CO)O | 0 | test |
| 251919791 | C1=CC(=CC(=C1)N2C(=O)C=CC2=O)N3C(=O)C=CC3=O | 1 | train |
| 251919790 | C1=CC=C(C=C1)OC(=O)Cl | 0 | train |
| 251919789 | C1=CC=C(C=C1)/C=C/C#N | 0 | validation |
| 251919788 | CC(C)OP(OC(C)C)OC(C)C | 0 | test |
| 251919787 | CCCCCCCCCCCC(=O)Cl | 0 | train |
| 251919786 | C1CC1(C(CC2=CC=CC=C2Cl)(CN3C=NC=N3)O)Cl | 1 | train |
| 251919785 | CC(C)(C)C1=CC(=CC(=C1O)C(C)(C)C)CCC(=O)NCCCCCCNC(=O)CCC2=CC(=C(C(=C2)C(C)(C)C)O)C(C)(C)C | 1 | validation |
| 251919784 | CCCCCCCCSCC1=CC(=C(C(=C1)CSCCCCCCCC)O)C | 0 | test |
| 251919783 | CCC[C@@H](C(=O)OCC)N[C@@H](C)C(=O)N1[C@H]2CCCC[C@H]2C[C@H]1C(=O)O.CC(C)(C)N | 0 | train |
| 251919782 | CCCCCC1=CC=C(C=C1)O | 1 | train |
| 251919781 | C(COCCC#N)C#N | 0 | validation |
| 251919780 | CS(=O)(=O)C | 0 | test |
| 251919779 | C([C@H]([C@H]([C@H](C=O)O)O)O)O | 0 | train |
| 251919778 | CO[Si](CCC1CCC2C(C1)O2)(OC)OC | 0 | train |
| 251919777 | C1=CC=C2C=C(C(=CC2=C1)O)O | 0 | validation |
| 251919776 | C1=C(C=C(C(=C1Cl)O)N)[N+](=O)[O-] | 0 | test |
| 251919775 | CCNC1=NC(=NC(=N1)OC)NC(C)(C)C | 1 | train |
| 251919774 | CCOP(=S)(OCC)SCS(=O)(=O)C(C)(C)C | 0 | train |
| 251919773 | C[Si](C)(C=C)Cl | 0 | validation |
| 251919772 | CCOC(=O)[C@H](C)O | 0 | test |
| 251919771 | CCOP(=S)(OCC)SCS(=O)(=O)CC | 0 | train |
| 251919770 | CCOP(=S)(OCC)SCCS(=O)(=O)CC | 1 | train |
| 251919769 | CC1CCC(CC1)C(C)(C)O | 0 | validation |
| 251919768 | CCN(CC)C1=CC=C(C=C1)N=NC2=CC=CC=C2 | 1 | test |
| 251919767 | CCN(CC)C1=CC2=C(C=C1)C3(C4=C(O2)C=C(C=C4)N(CC)CC)C5=CC=CC=C5C(=O)O3 | 0 | train |
| 251919766 | B(OCC)(OCC)OCC | 0 | train |
| 251919765 | C(C(=O)N)Cl | 0 | validation |
| 251919764 | CC/C=C¥C/C=C¥C/C=C¥CCCCCCCC(=O)OC | 0 | test |
| 251919763 | CC1=CC(=C(C(=C1)C(C)(C)C)O)C | 0 | train |
| 251919762 | CC1=CC=C(C=C1)C=O | 0 | train |
| 251919761 | C1=CC(=CC=C1C2=C(C(=C(N2)C(F)(F)F)Br)C#N)Cl | 1 | validation |
| 251919760 | CCCC(C)CCC(=O)C | 0 | test |
| 251919759 | C/C=C/C(=O)C1=C(C=CCC1(C)C)C | 0 | train |
| 251919758 | C1=CC2=C3C(=C(C=C2S(=O)(=O)[O-])S(=O)(=O)[O-])C=CC4=C(C=C(C1=C43)S(=O)(=O)[O-])S(=O)(=O)[O-].[Na+].[Na+].[Na+].[Na+] | 0 | train |
| 251919757 | COC(=O)C12CC3=C(C1=NN(CO2)C(=O)N(C4=CC=C(C=C4)OC(F)(F)F)C(=O)OC)C=CC(=C3)Cl | 0 | validation |
| 251919756 | C/C(=C¥C1=CC=C(C=C1)C(=O)O)/C2=CC3=C(C=C2)C(CCC3(C)C)(C)C | 0 | test |
| 251919755 | C1=CC=C(C=C1)[Sn](C2=CC=CC=C2)(C3=CC=CC=C3)Cl | 1 | train |

| | | | |
|---|---|---|---|
| 251919754 | CC1=CC(=C(C=C1)N/N=C/2¥C(=O)C=CC3=CC=CC=C32)C | 1 | train |
| 251919753 | C1=CC=C2C(=O)C=CC(=O)C2=C1 | 1 | validation |
| 251919752 | CCC(CO)O | 0 | test |
| 251919751 | C1=CC(=CC(=C1)C(F)(F)F)/C(=N/NC(=O)NC2=CC=C(C=C2)OC(F)(F)F)/CC3=CC=C(C=C3)C#N | 0 | train |
| 251919750 | C1=CC=C(C=C1)C2=CC=C(C=C2)Cl | 0 | train |
| 251919749 | C1(=C(C(=C(C(=C1Cl)Cl)Cl)Cl)Cl)[O-].[Na+] | 1 | validation |
| 251919748 | COP(=O)(CCC(=O)NCO)OC | 0 | test |
| 251919747 | CC(=O)CC(=O)N | 0 | train |
| 251919746 | C1=CC(=CC(=C1)CN=C=O)CN=C=O | 0 | train |
| 251919745 | CC(C1=C(C(=CC(=C1)C(C)(C)C)C(C)(C)C)O)C2=C(C(=CC(=C2)C(C)(C)C)C(C)(C)C)O | 0 | validation |
| 251919744 | C1=CC(=C(C=C1[N+](=O)[O-])C#N)N | 1 | test |
| 251919743 | C1=CC(=C(C=C1[N+](=O)[O-])N)Cl | 0 | train |
| 251919742 | CC(C)C(=O)C(C)C | 0 | train |
| 251919741 | CC1=CC2=NNN=C2C=C1 | 0 | validation |
| 251919740 | C1[C@H]2[C@@H]([C@H]([C@@H]1Cl)Cl)[C@@]3(C(=C([C@]2(C3(Cl)Cl)Cl)Cl)Cl)Cl | 0 | test |
| 251919739 | CC1([C@H]([C@H]1C(=O)OCC2=CC(=CC=C2)OC3=CC=CC=C3)C=C(Cl)Cl)C | 0 | train |
| 251919738 | C1[C@H]2[C@@H]([C@H]([C@H]1Cl)Cl)[C@@]3(C(=C([C@]2(C3(Cl)Cl)Cl)Cl)Cl)Cl | 0 | train |
| 251919737 | CC(C)(C)C1=NNC(=O)N(C1=O)N | 0 | validation |
| 251919736 | CCC1=CC=CC(=C1N(C(C)COC)C(=O)CS(=O)(=O)O)C | 0 | test |
| 251919735 | CCOC(=O)C1=NN(C(C1)(C)C(=O)OCC)C2=C(C=C(C=C2)Cl)Cl | 1 | train |
| 251919734 | CC(C)(C)C(C(N1C=NC=N1)OC2=CC=C(C=C2)C3=CC=CC=C3)O | 1 | train |
| 251919733 | CCC1=CC=CC(=C1N(COCC)C(=O)CS(=O)(=O)O)C | 0 | validation |
| 251919732 | C1=CC=C(C=C1)[Si](C2=CC=CC=C2)(C3=CC=CC=C3)O | 1 | test |
| 251919731 | C[C@]12CC[C@H](C[C@@H]1CC[C@@H]3[C@@H]2CC[C@]4([C@H]3CCC4=O)C)O | 0 | train |
| 251919730 | CCC1(C(=O)N(C(=O)N1Cl)Cl)C | 0 | train |
| 251919729 | COC1=C(C=C(C=C1)[N+](=O)[O-])[O-].[Na+] | 0 | validation |
| 251919728 | CC1(C(=O)N(C(=O)N1CO)CO)C | 0 | test |
| 251919727 | OS(=O)[O-].[Na+] | 0 | train |
| 251919726 | C1CC1(C(CC2=CC=CC=C2Cl)(CN3C(=S)N=CN3)O)Cl | 0 | train |
| 251919725 | C[C@H](C(=O)O)OC1=C(C=C(C=C1)Cl)Cl | 0 | validation |
| 251919724 | CCC1=CC(=CC(=C1C2=C(N3CCOCCN3C2=O)OC(=O)C(C)(C)C)CC)C | 0 | test |
| 251919723 | CN(C)C(=O)C1=CC=CC=C1NS(=O)(=O)NC(=O)NC2=NC(=CC(=N2)OC)OC | 0 | train |
| 251919722 | CC1=CC(=NC(=O)N1)C.C1=CC(=CC=C1NC(=O)NC2=CC=C(C=C2)[N+](=O)[O-])[N+](=O)[O-] | 0 | train |
| 251919721 | C1=CC(=C(C(=C1)Cl)C(=O)NCC2=C(C=C(C=N2)C(F)(F)F)Cl)Cl | 0 | validation |
| 251919720 | CC1=CSC(=C1N([C@@H](C)COC)C(=O)CCl)C | 0 | test |
| 251919719 | C/C(=N¥NC(=O)NC1=CC(=CC(=C1)F)F)/C2=C(C=CC=N2)C(=O)O | 0 | train |
| 251919718 | CCC1=C(C(=O)C=NN1C2=CC=C(C=C2)Cl)C(=O)[O-].[K+] | 0 | train |
| 251919717 | CCC(=C1C(=O)CC(CC1=O)CC(C)SCC)NOC/C=C/Cl | 0 | validation |
| 251919716 | CCOC1=NC(=CC2=NC(=NN21)S(=O)(=O)NC3=C(C=CC=C3Cl)C(=O)OC)F | 0 | test |
| 251919715 | CN1C(=O)C=CS1 | 0 | train |
| 251919714 | CC(C)N.C1=CC(=C(C=C1Cl)Cl)OCC(=O)O | 0 | train |
| 251919713 | CC1=C(C(=CC=C1)C)N([C@H](C)C(=O)OC)C(=O)COC | 0 | validation |
| 251919712 | CC1=C(C=CC(=C1)Cl)O[C@H](C)C(=O)O | 0 | test |
| 251919711 | CCS(=O)(=O)C1=C(N=CC=C1)S(=O)(=O)NC(=O)NC2=NC(=CC(=N2)OC)OC | 1 | train |
| 251919710 | CC1=CC(=CC(=C1SC)C)OC(=O)NC | 1 | train |
| 251919709 | CC1([C@H]([C@H]1C(=O)O[C@H](C#N)C2=CC(=CC=C2)OC3=CC=CC=C3)/C=C(/C(F)(F)F)¥Cl)C | 0 | validation |
| 251919708 | CC1=CC=C(C=C1)S(=O)(=O)C(I)I | 1 | test |
| 251919707 | CC1=CC2=C(C=C1)N=C3C(=N2)SC(=O)S3 | 0 | train |
| 251919706 | C(S(=O)(=O)C(Cl)(Cl)Cl)(Cl)(Cl)Cl | 1 | train |
| 251919705 | CC1(COCN1)C | 0 | validation |
| 251919704 | C(/C=C¥Cl)Cl | 0 | test |
| 251919703 | C1C(O1)C(=O)N | 0 | train |
| 251919702 | C(N(CP(=O)([O-])[O-])CP(=O)([O-])[O-])P(=O)(O)[O-].[Na+].[Na+].[Na+].[Na+].[Na+] | 0 | train |
| 251919701 | C(CN(CC(=O)O)CC(=O)O)CN(CC(=O)O)CC(=O)O | 0 | validation |
| 251919700 | C1CC=CC1 | 0 | test |
| 251919699 | CCCCCCCCCCCCCC1=CC=CC=C1 | 0 | train |
| 251919698 | CCCCCCCOC(=O)C1=CC=CC=C1C(=O)OCCCCCCC | 1 | train |
| 251919697 | CCC1=C(C(=C(C=C1C)N)CC)N | 1 | validation |
| 251919696 | CCCCCCCCCCCCCC[N+](C)(C)CC1=CC=CC=C1.[Cl-] | 0 | test |

| | | | |
|---|---|---|---|
| 251919695 | CCCCCCCCCCCCCCCCCO | 0 | train |
| 251919694 | CC(C)(C)C1=CC(=CC(=C1O)C(C)(C)C)CCC(=O)OCCSCCOC(=O)CCC2=CC(=C(C(=C2)C(C)(C)C)O)C(C)(C)C | 0 | train |
| 251919693 | COP(=S)(N)OC | 0 | validation |
| 251919692 | C/C=C/C/C=C/C/C=C/CC=C | 0 | test |
| 251919691 | CC1=CC(=CC=C1)OP(=O)(OC2=CC=CC(=C2)C)OC3=CC=CC(=C3)C | 1 | train |
| 251919690 | CCC(=O)O | 0 | train |
| 251919689 | CC(C)(COCC1CO1)COCC2CO2 | 0 | validation |
| 251919688 | CC1=CC=CC2=C1C=CC3=CC=CC=C32 | 1 | test |
| 251919687 | CC(C=C)C#N | 0 | train |
| 251919686 | CN(C)C1=CC=C(C=C1)C(=C2C=CC(=[N+](C)C)C=C2)C3=CC=CC=C3.[Cl-] | 1 | train |
| 251919685 | C(CO)C#N | 0 | validation |
| 251919684 | CC(C#N)O | 0 | test |
| 251919683 | C1N(CN(CN1CCO)CCO)CCO | 0 | train |
| 251919682 | CCOC(=O)CC(C)O | 0 | train |
| 251919681 | C1=CC=C(C=C1)OC2=C(C3=C(C(=C2)O)C(=O)C4=CC=CC=C4C3=O)N | 1 | validation |
| 251919680 | CCCCCCCCC1=CC=C(C=C1)NC2=CC=C(C=C2)CCCCCCCC | 0 | test |
| 251919679 | C=CC(=O)OCC(CBr)Br | 0 | train |
| 251919678 | C1=CC=C2C(=C1)C(=O)OC23C4=C(C(=C(C=C4)O)Br)OC5=C3C=CC(=C5Br)O | 0 | train |
| 251919677 | COC1=CC(=C(C=C1)OC)N/N=C/2¥C(=O)C=CC3=CC=CC=C32 | 1 | validation |
| 251919676 | CCOC(/C=C(¥C)/CCC=C(C)C)OCC | 0 | test |
| 251919675 | C(CO)CCl | 0 | train |
| 251919674 | CC(CN(C)C)Cl.Cl | 0 | train |
| 251919673 | C1=CC=C2C(=C1)C3=C4C2=CC5=CC=CC6=C5C4=C(C=C6)C=C3 | 1 | validation |
| 251919672 | C1(C(C(C(C(C1Cl)Cl)Cl)Cl)Cl)Cl | 0 | test |
| 251919671 | CCOC(=O)C1=CC=CC=C1S(=O)(=O)NC(=O)NC2=NC(=CC(=N2)Cl)OC | 0 | train |
| 251919670 | CC(C)NCC(COC1=CC=C(C=C1)CCOC)O | 0 | train |
| 251919669 | C1=CC=C(C=C1)C2=CC(=CC=C2)O | 1 | validation |
| 251919668 | CCC(C)C1=CC=CC=C1 | 0 | test |
| 251919667 | C1=CC=C(C(=C1)C(=C(Cl)Cl)C2=CC=C(C=C2)Cl)Cl | 1 | train |
| 251919666 | CCOP(=O)(OCC)SCCSCC | 0 | train |
| 251919665 | CCN(CC)C1=CC(=C(C=C1)C=O)O | 1 | validation |
| 251919664 | C1=CC(=CN=C1)CCCO | 0 | test |
| 251919663 | CCCCCCCC(=O)C | 0 | train |
| 251919662 | CCCCCCCCC(=O)C | 0 | train |
| 251919661 | COC(=O)C1=CC=C(C=C1)[N+](=O)[O-] | 0 | validation |
| 251919660 | C1=C(C=C(C(=C1I)O)I)I | 0 | test |
| 251919659 | C[C@]12CC[C@H](C1(C)C)CC2=O | 0 | train |
| 251919658 | COC1=CC=CC(=C1)O | 0 | train |
| 251919657 | CCC1=CC(=CC=C1)CC | 0 | validation |
| 251919656 | CC1CNCC(O1)C | 0 | test |
| 251919655 | CCCC(C)C=O | 0 | train |
| 251919654 | C1=CC(=C(C=C1[N+](=O)[O-])Cl)N | 1 | train |
| 251919653 | CCN(CC)C1=CC=C(C=C1)C=O | 1 | validation |
| 251919652 | C1=CNC=C1 | 0 | test |
| 251919651 | CCC1=CN=C(C=C1)C | 0 | train |
| 251919650 | C1=CC(=CC=C1[N+](=O)[O-])[N+](=O)[O-] | 1 | train |
| 251919649 | CCC1=C(C(=C(C(=C1Br)Br)Br)Br)Br | 1 | validation |
| 251919648 | CC[N+](CC)(CC)CC1=CC=CC=C1.[Cl-] | 0 | test |
| 251919647 | CC(=O)C=O | 0 | train |
| 251919646 | C(#N)Br | 0 | train |
| 251919645 | CC1=C/C(=N/NC2=CC=C(C=C2)NC(=O)C)/C(=O)C=C1 | 1 | validation |
| 251919644 | CC1=CC=CC(=C1)C=C | 0 | test |
| 251919643 | C1=CC(=CC=C1N)N.Cl.Cl | 0 | train |
| 251919642 | CN(CCCC(=O)C1=CN=CC=C1)N=O | 0 | train |
| 251919641 | CN(CCCC(C1=CN=CC=C1)O)N=O | 0 | validation |
| 251919640 | C1(=C(C(=C(C(=C1Br)Br)Br)Br)Br)OC2=C(C(=C(C(=C2Br)Br)Br)Br)Br | 0 | test |
| 170466899 | CCCCCCCC(=O)[O-].CCCCCCCC(=O)[O-].[Zn+2] | 0 | train |
| 170466898 | CN1C=NC2=C1C=C(C(=C2F)NC3=C(C=C(C=C3)Br)Cl)C(=O)NOCCO | 0 | train |
| 170466897 | CC(C)N1C2=C(/C(=C/3¥C=C4C=C(C=CC4=N3)O)/N1)C(=NC=N2)N | 1 | validation |
| 170466896 | C1CC(C1)(C2=CC=C(C=C2)C3=C(C=C4C(=N3)C=CN5C4=NNC5=O)C6=CC=CC=C6)N | 1 | test |
| 170466895 | CN1CCC(CC1)NC2=NC=C3C(=N2)C(=NC=N3)NC4=CC(=C(C=C4)F)Cl.Cl.Cl | 1 | train |

| | | | |
|---|---|---|---|
| 170466894 | CCCCC/C=C¥C/C=C¥C/C=C¥C/C=C¥CCCC(=O)NCC(=O)O | 0 | train |
| 170466893 | CC(=O)C1=CC=C(C=C1)C2=CC=C(C=C2)C(=O)NCCCCN3CCN(CC3)C4=CC=CC=C4OC | 0 | validation |
| 170466892 | CN1C=CC2=CC3=C(CCN3C(=O)NC4=CN=CC=C4)C=C21.Cl | 1 | test |
| 170466891 | CC1(CO[C@H](CN1)CC(=O)O)C | 0 | train |
| 170466889 | CN(C)CC[C@@H](C1=CC=C(C=C1)Cl)C2=CC=CC=N2.Cl | 0 | validation |
| 170466888 | CC1=NN=C(O1)C(=O)NC(C)(C)C2=NC(=C(C(=O)N2C)O)C(=O)NCC3=CC=C(C=C3)F | 0 | test |
| 170466887 | CCCCCN=C(N)NN/C=C/1¥C=NC2=C1C=C(C=C2)OC | 1 | train |
| 170466886 | C1=CC=C2C(=C1)C=CC=C2NCCN.Cl.Cl | 0 | train |
| 170466885 | CC1=CC=C(C=C1)OC(=O)C | 0 | validation |
| 170466884 | CCC(C)(C)C1=CC=C(C=C1)O | 0 | test |
| 170466883 | C(/C=C/C(=O)O)N | 0 | train |
| 170466882 | C[C@H]1CNCCC2=C1C=C(C=C2)Cl | 0 | train |
| 170466881 | CC1=CC=C(C=C1)S(=O)(=O)N[C@@H](CCCCN)C(=O)CCl.Cl | 0 | validation |
| 170466880 | CCN(CC)C(=O)C1=CC=C(C=C1)[C@H](C2=CC(=CC=C2)OC)N3C[C@H](N(C[C@@H]3C)CC=C)C | 0 | test |
| 170466879 | CC1=CC(=C(C=C1)Cl)O | 0 | train |
| 170466878 | CC(C)C[C@@H](C(=O)[C@]1(CO1)C)NC(=O)[C@H](CC2=CC=CC=C2)NC(=O)[C@H](CC(C)C)NC(=O)[C@H](CCC3=CC=CC=C3)NC(=O)CN4CCOCC4 | 1 | train |
| 170466877 | C1=CN=CC=C1C(=O)NN | 0 | validation |
| 170466876 | C1=CC(=C(C=C1C[C@@H](C(=O)O)N)I)OC2=CC(=C(C(=C2)I)O)I | 0 | test |
| 170466875 | C1=CC(=CC=C1N)O | 0 | train |
| 170466874 | CN1C2=C(C(=O)N(C1=O)C)NC=N2 | 0 | train |
| 170466873 | C1CCN(CC1)CC2=CC(=CC=C2)OCCCNC3=NC4=CC=CC=C4S3.C(=C¥C(=O)O)¥C(=O)O.C(=C¥C(=O)O)¥C(=O)O | 0 | validation |
| 170466872 | C[C@@H]1C[C@H]2[C@@H]3CCC4=CC(=O)C=C[C@@]4([C@]3([C@H](C[C@@]2([C@]1(C(=O)COC(=O)C)O)C)O)F)C | 0 | test |
| 170466871 | C1=CC=C(C=C1)CCCO | 0 | train |
| 170466870 | C1=C(C=C(C(=C1Br)O)Br)Br | 0 | train |
| 170466869 | C1[C@H]([C@@H]([C@@H](C[C@]1(C(=O)O)OC(=O)C=CC2=CC(=C(C=C2)O)O)OC(=O)C=CC3=CC(=C(C=C3)O)O)O)O | 0 | validation |
| 170466868 | C1=CC=C(C(=C1)C(F)(F)F)N2C=CN=C2 | 0 | test |
| 170466867 | COC(=O)[C@H](CCCN=C(N)N[N+](=O)[O-])N | 0 | train |
| 170466866 | CN([C@H]1CC[C@@]2(CCCO2)C[C@@H]1N3CCCC3)C(=O)CC4=CC=CC=C4 | 0 | train |
| 170466865 | CC1=NC=C(C(=C1O)C=O)CO | 0 | validation |
| 170466864 | C1CCC(CC1)NC2CCCCC2 | 0 | test |
| 170466863 | CCOC(=O)C1=CN=CC=C1 | 0 | train |
| 170466862 | C1=CC=C(C(=C1)C2=NC(=NO2)C3=CC(=CC=C3)C(=O)O)F | 1 | train |
| 170466861 | C1=CC=C(C=C1)NC(=O)CS | 0 | validation |
| 170466860 | C1=CC(=C(C=C1C=C(C#N)C#N)O)O | 0 | test |
| 170466859 | CCN(CC1=CN=C(C=C1)Cl)/C(=C/[N+](=O)[O-])/NC | 0 | train |
| 170466858 | C1=CC(=CC=C1N)N | 1 | train |
| 170466857 | CN(C)CCOC(C1=CC=CC=C1)C2=CC=CC=C2 | 0 | validation |
| 170466856 | CCOC(=O)[C@](C)(CC1=CC(=C(C=C1)O)O)N.Cl | 0 | test |
| 170466855 | CCCCCCC(=O)CCCCCC/C=C/C[C@H]([C@@H]([C@@](CO)(C(=O)O)N)O)O | 0 | train |
| 170466854 | CC(CN1CCN(CCN(CCN(CC1)CC(=O)[O-])CC(=O)[O-])CC(=O)[O-])O.[Gd+3] | 0 | train |
| 170466853 | C1=CC(=CC=C1C[C@@H](C(=O)O)N)O | 0 | validation |
| 170466852 | CC(C(=O)O)O | 0 | test |
| 170466851 | C[C@@H]1C[C@H]2[C@@H]3CCC4=CC(=O)C=C[C@@]4([C@]3([C@H](C[C@@]2([C@]1(C(=O)COC(=O)C5=CC=NC=C5)O)C)O)F)C | 0 | train |
| 170466850 | C1[C@H]([C@@H]([C@@H]([C@@](O1)(CO)O)O)O)O | 0 | train |
| 170466849 | CC1=CC(=CC=C1)OC(=O)C | 0 | validation |
| 170466848 | C1=CC(=CC=C1C2=COC3=CC(=CC(=C3C2=O)O)O)O | 1 | test |
| 170466847 | C(/C(=C¥C(=O)O)/C(=O)O)C(=O)O | 0 | train |
| 170466846 | C1=CN=CC(=C1N)N | 0 | train |
| 170466845 | C[C@]12CCC(=O)C=C1CC[C@@H]3[C@@H]2[C@H](C[C@]4([C@H]3CC[C@@H]4C(=O)CO)C)O | 0 | validation |
| 170466844 | CCOC(=O)C1=CC=CC=C1O | 0 | test |
| 170466843 | CCCCCCCCCCCC(=O)O[Sn](CCCC)(CCCC)OC(=O)CCCCCCCCCCC | 0 | train |
| 170466842 | CCCCCC(=CC1=CC=CC=C1)C=O | 0 | train |
| 170466841 | CCC(=O)C1=CC2=C(C=C1)SC3=CC=CC=C3N2CCCN(C)C.Cl | 0 | validation |
| 170466840 | CN(C)S(=O)(=O)C1=CC=C(C=C1)OP(=S)(OC)OC | 0 | test |
| 170466839 | C1[C@@H]2[C@H]([C@H]([C@@H](O2)N3C=NC4=C3N=CN=C4N)O)OP(=O)(O1)O | 0 | train |

| | | | |
|---|---|---|---|
| 170466838 | C1=CC(=CC=C1/C=C/C2=CC(=CC(=C2)O)O)O | 1 | train |
| 170466837 | C/C=C/1¥[C@@H]2CC3=C([C@]1(CC(=C2)C)N)C=CC(=O)N3 | 0 | validation |
| 170466836 | C1CC1C(=O)NC2=CC(=C(C=C2)Cl)Cl | 1 | test |
| 170466835 | C1CC1CONC(=O)C2=C(C(=C(C=C2)F)F)NC3=C(C=C(C=C3)I)Cl | 0 | train |
| 170466834 | C1=CC=C(C=C1)NN.Cl | 1 | train |
| 170466833 | C1=CC=C2C(=C1)C(=O)C3=CC=CC=C3C2=O | 1 | validation |
| 170466832 | C1=CC=C(C(=C1)C2=C3C=C(C(=O)C(=C3OC4=C(C(=C(C=C24)Br)[O-])Br)Br)Br)C(=O)[O-].[Na+].[Na+] | 0 | test |
| 170466831 | C(C(=O)O)N | 0 | train |
| 170466830 | C(C(CS)O)O | 0 | train |
| 170466829 | CC1CC(=O)NN=C1C2=CC=C(C=C2)NC(=NC)NC#N | 0 | validation |
| 170466828 | CC1=NC=C(C(=C1O)CN)CO.Cl.Cl | 0 | test |
| 170466827 | CCOC1=C(C=CC(=C1)NC(=O)C)C(=O)OC | 0 | train |
| 170466826 | CC(C)OC1=CC=C(C=C1)NC(=O)N2CCN(CC2)C3=NC=NC4=CC(=C(C=C43)OC)OCCCN5CCCCC5 | 0 | train |
| 170466825 | C[C@@H]1CN(CCN1[C@H](COC)C2=CC=C(C=C2)C(F)(F)F)C3(CCN(CC3)C(=O)C4=C(N=CN=C4C)C)C.C(=C¥C(=O)O)¥C(=O)O | 0 | validation |
| 170466824 | CCCC(=O)O[C@@]1(CC[C@@H]2[C@@]1(C[C@@H]([C@H]3[C@H]2CCC4=CC(=O)CC[C@]34C)O)C)C(=O)CC | 0 | test |
| 170466823 | C([C@@H]1[C@@H]([C@@H]([C@H]([C@H](O1)O[C@]2([C@H]([C@@H]([C@H](O2)CCl)O)O)CCl)O)O)Cl)O | 0 | train |
| 170466822 | C1=CC=NC(=C1)C(=O)O | 0 | train |
| 170466821 | CCS(=O)(=O)C(C)(C)S(=O)(=O)CC | 0 | validation |
| 170466820 | CN(C)CCC1=CC=C(C=C1)O | 0 | test |
| 170466819 | CC1=C(C=CC(=C1)OCCOCC[N+](C)(C)CC2=CC=CC=C2)C(C)(C)CC(C)(C)C.[Cl-] | 0 | train |
| 170466818 | C1=C(C=C(C=C1[N+](=O)[O-])[N+](=O)[O-])C(=O)N | 0 | train |
| 170466817 | C1=CC(=C(C=C1C2=C(C(=O)C3=C(C=C(C=C3O2)O)O)O)O)O.O.O | 0 | validation |
| 170466816 | CC(=C)[C@H]1CC2=C(O1)C=CC3=C2O[C@@H]4COC5=CC(=C(C=C5[C@@H]4C3=O)OC)OC | 0 | test |
| 170466815 | C1=CC(=CC=C1CCN)O | 0 | train |
| 170466814 | C1=C(OC=C(C1=O)O)CO | 0 | train |
| 170466813 | C[C@H]1C(=O)O[C@@H]2CCN3[C@@H]2C(=CC3)COC(=O)[C@]([C@]1(C)O)(C)O | 0 | validation |
| 170466812 | CC[C@H]1C(=O)N(CC(=O)N([C@H](C(=O)N[C@H](C(=O)N([C@H](C(=O)N[C@H](C(=O)N[C@@H](C(=O)N([C@H](C(=O)N([C@H](C(=O)N([C@H](C(=O)N([C@H](C(=O)N1)[C@@H]([C@H](C)C/C=C/C)O)C)C(C)C)C)CC(C)C)C)CC(C)C)C)C)CC(C)C)C)C(C)C)CC(C)C)C)C | 0 | test |
| 170466811 | CC(C)OC1=CC2=C(C=C1)C(=O)C(=CO2)C3=CC=CC=C3 | 1 | train |
| 170466810 | COP(=S)(OC)SCN1C(=O)C2=CC=CC=C2C1=O | 0 | train |
| 170466809 | C=CCOC(CN1C=CN=C1)C2=C(C=C(C=C2)Cl)Cl.OS(=O)(=O)O | 1 | validation |
| 170466808 | CC1=C(C(=CC=C1)C)N(C(C)C(=O)OC)C(=O)COC | 0 | test |
| 170466807 | CC1=C(C=CC(=C1)OP(=S)(OC)OC)SC | 1 | train |
| 170466806 | CCNC1=NC(=NC(=N1)Cl)NCC | 0 | train |
| 170466805 | C(C(=O)O)(Cl)Cl | 0 | validation |
| 170466804 | C1=CNC(=S)NC1=O | 0 | test |
| 170466803 | C1=CC(=CN=C1)CO | 0 | train |
| 170466802 | CN(C)C(=O)NC1=CC(=C(C=C1)Cl)Cl | 1 | train |
| 170466801 | CC1=C2C(=CC(=C1C(=O)O)O)C(=O)C3=C(C2=O)C(=C(C(=C3O)O)[C@H]4[C@@H]([C@H]([C@@H]([C@H](O4)CO)O)O)O)O | 0 | validation |
| 170466800 | CC1=C(C(=C2C(=C1)C(=C(C(=C2C=O)O)O)C(C)C)O)C3=C(C=C4C(=C3O)C(=C(C(=C4C(C)C)O)O)C=O)C | 0 | test |
| 170466799 | COC1=C(C2=C(CC3C4=CC5=C(C=C4CCN3C2)OCO5)C=C1)OC | 1 | train |
| 170466798 | CC(=O)OC1=CC=C(C=C1)C2(C3=CC=CC=C3NC2=O)C4=CC=C(C=C4)OC(=O)C | 0 | train |
| 170466797 | CC(=O)NC1=CC=C(C=C1)O | 0 | validation |
| 170466796 | CC(=O)OCC(=O)[C@]1(CCC2[C@@]1(C[C@@H](C3C2CCC4=CC(=O)CC[C@]34C)O)C)O | 0 | test |
| 170466795 | C(CS)N.Cl | 0 | train |
| 170466794 | C1=C(C=C(C(=C1I)O)I)C[C@@H](C(=O)O)N | 0 | train |
| 170466793 | CC1=C(N(C2=C1C=C(C=C2)O)CC3=CC=C(C=C3)OCCN4CCCCCC4)C5=CC=C(C=C5)O.CC(=O)O | 1 | validation |
| 170466792 | CC1(C(N2C(S1(=O)=O)CC2=O)C(=O)OCOC(=O)C(C)(C)C)C | 0 | test |
| 170466791 | CCCCCCCCCCCCOCCOCCOCCOCCOCCOCCOCCOCCOCCO | 0 | train |
| 170466790 | CC(C)C1=C(C=C2C(=C1)CC[C@@H]3[C@@]2(CCC[C@@]3(C)C(=O)[O-])C)S(=O)(=O)O.[Na+] | 0 | train |
| 170466789 | CS(=O)(=O)O.C1CCC2(C1)CC(=O)N(C(=O)C2)CCNCC3COC4=CC=CC=C4O3 | 0 | validation |

| | | | |
|---|---|---|---|
| 170466788 | CN1CCN(CC1)CCCOC2=C(C=C3C(=C2)N=CC(=C3NC4=CC(=C(C=C4Cl)Cl)OC)C#N)OC.CO | 0 | test |
| 170466787 | C1=CC=C(C=C1)CCO | 0 | train |
| 170466786 | COC1=C(C=CC(=C1)C=O)O | 0 | train |
| 170466785 | CC1=CC(=C(C(=C1)C(C)(C)C)O)C(C)(C)C | 0 | validation |
| 170466784 | COC(=O)/C=C¥C(=O)OC | 0 | test |
| 170466783 | CN(C1=CC=C(C=C1)OC(=O)C2=CC=CO2)C(=O)C(Cl)Cl | 0 | train |
| 170466782 | C1=CC=C(C=C1)[C@H](CNCCOC2=CC=C(C=C2)CC(=O)O)O.Cl | 0 | train |
| 170466781 | C1=CC=C(C(=C1)CN)SC2=CC=CC=C2CO | 0 | validation |
| 170466780 | CCCCOC(=O)CCCCCCCCC(=O)OCCCC | 0 | test |
| 170466779 | CC(=C)C(=O)OCC(CO)O | 0 | train |
| 170466778 | C1=C(OC(=C1)[N+](=O)[O-])C=CC(=NN=C(N)N)C=CC2=CC=C(O2)[N+](=O)[O-] | 1 | train |
| 170466777 | CN1CC(=O)N=C1N | 0 | validation |
| 170466776 | CC1=C(C(=NC(=N1)N)N)C2=CC(=C(C=C2)Cl)Cl | 0 | test |
| 170466775 | C1=CC(=C(C(=C1C=O)O)O)O | 0 | train |
| 170466774 | C1CNCCC1(C2=CC=CC=C2)O | 0 | train |
| 170466773 | C1=CC(=O)NC=C1C(=O)O | 0 | validation |
| 170466772 | COC1=C(C=C2C(=C1)CCC2=O)OC | 0 | test |
| 170466771 | CC(=O)NC1=C(C=C(C(=C1)OC)C(=O)OC)Cl | 0 | train |
| 170466770 | C1=CC(=CC(=C1)Cl)CCl | 0 | train |
| 170466769 | CC1=CC=C(C=C1)C2=CC=CC=C2C#N | 0 | validation |
| 170466768 | CC(=O)OC[C@@H]1[C@H]([C@H]([C@@H](O1)OC(=O)C)OC(=O)C)OC(=O)C | 0 | test |
| 170466767 | CC(C)OP(=O)(CP(=O)(OC(C)C)OC(C)C)OC(C)C | 0 | train |
| 170466766 | C[C@@H]1CC[C@@]2([C@H]([C@H]3[C@@H](O2)C[C@@H]4[C@@]3(C(=O)C[C@H]5[C@H]4CC[C@@H]6[C@@]5(CC[C@@H](C6)O)C)C)C)OC1 | 0 | train |
| 170466765 | C1=CC(=C(C=C1N)C(F)(F)F)[N+](=O)[O-] | 0 | validation |
| 170466764 | COC1=CC=C(C=C1)[C@H]2[C@H](C(=O)NC3=CC=CC=C3S2)O | 0 | test |
| 170466763 | COC(=O)C1=C(N=C(C(=N1)Cl)Cl)N | 1 | train |
| 170466762 | COC1=C(C=C(C=C1)S(=O)(=O)N)C(=O)OC | 0 | train |
| 170466761 | CNCC1=CC=CC=C1.Cl | 0 | validation |
| 170466760 | C1CN(CCN1)C2=CC(=CC=C2)Cl | 0 | test |
| 170466759 | CC1=NC2=C(C=C1)C=CC(=C2)Cl | 0 | train |
| 170466758 | CC1=C(C=CS1)Br | 0 | train |
| 170466757 | CC(=O)C1=CC2=CC=CC=C2S1 | 0 | validation |
| 170466756 | CCOC(=O)C(=O)C1=CSC(=N1)NC=O | 0 | test |
| 170466755 | CC1=C(N2[C@@H]([C@@H](C2=O)N)SC1)C(=O)O | 0 | train |
| 170466754 | C1=CC=C(C(=C1)S)Cl | 0 | train |
| 170466753 | C1=CC(=CC=C1S(=O)(=O)NC(=O)N)Cl | 0 | validation |
| 170466752 | COC1=CC2=C(C=C1)C(=O)CCC2 | 0 | test |
| 170466751 | C1CC(CCC1=O)(C#N)C2=CC=C(C=C2)F | 0 | train |
| 170466750 | COC1=CC2=C(C=C1)C=C(C=C2)C=O | 0 | train |
| 170466749 | CCOC(=O)C(=O)CCC1=CC=CC=C1 | 0 | validation |
| 170466748 | CC(=O)C1=CC(=C(C=C1)O)C(=O)N | 0 | test |
| 170466747 | C1=CC=C(C=C1)[P+](CCCCC(=O)O)(C2=CC=CC=C2)C3=CC=CC=C3.[Br-] | 0 | train |
| 170466746 | C1=CC=C(C(=C1)C(=O)C2=C(C=CC(=C2)Cl)NC(=O)CBr)F | 1 | train |
| 170466745 | CN1CCN(CC1)N | 0 | validation |
| 170466744 | C1CN(CCC1N)C2=NC(=CC=C2)Cl.Cl | 0 | test |
| 170466743 | C1CCN(C1)CCCl | 0 | train |
| 170466742 | CC1=C(C(=NO1)C2=C(C=CC=C2Cl)Cl)C(=O)Cl | 0 | train |
| 170466741 | C1CN(CCN1)C2=CC=CC=C2 | 0 | validation |
| 170466740 | C1=C/C(=C/2¥NN=CO2)/C(=O)C=C1 | 0 | test |
| 170466739 | C1=NC2=C(N1)C(=O)N=CN2 | 0 | train |
| 170466738 | COC1=C(C=C2C(=C1)C(=NC(=N2)Cl)N)OC | 0 | train |
| 170466737 | COCCC1=CC=C(C=C1)OCC2CO2 | 0 | validation |
| 170466736 | C1CN(CCN1)C(C2=CC=C(C=C2)F)C3=CC=C(C=C3)F | 0 | test |
| 170466735 | C[C@]12CC[C@H]3[C@H]([C@@H]1CC[C@@H]2C(=O)O)CCC4=CC(=O)CC[C@]34C | 0 | train |
| 170466734 | COC1=C(C=C2C(=C1)C(=O)NC(=O)N2)OC | 0 | train |
| 170466733 | CC1=CC(=C(C(=C1O)C)C)O | 0 | validation |
| 170466732 | CC(C)(C)C1=CC=C(C=C1)C(=O)CCCCl | 1 | test |
| 170466731 | CC(=O)OCC1=C(N2[C@@H]([C@@H](C2=O)N)SC1)C(=O)O | 0 | train |
| 170466730 | C([C@@H]1[C@@H]([C@@H]([C@H]([C@@H](O1)O[C@H]([C@@H](CO)O)[C@@H]([C@H](C(=O)O)O)O)O)O)O)O | 0 | train |
| 170466729 | COC1=C(C=C(C=C1)Cl)C(=O)NCCC2=CC=C(C=C2)S(=O)(=O)N | 0 | validation |

| | | | |
|---|---|---|---|
| 170466728 | C1=CC=C2C(=C1)N=C(N2CC3=CC=C(C=C3)F)N | 1 | test |
| 170466727 | C1=CC(=C(C=C1Cl)Cl)C(=O)CCl | 1 | train |
| 170466726 | C1CN(CCN1CCCCl)C2=CC(=CC=C2)Cl.Cl | 0 | train |
| 170466725 | C1CC2=CC=CC=C2NC3=CC=CC=C31 | 1 | validation |
| 170466724 | CC¥1=C(C2=C(/C1=C¥C3=CC=C(C=C3)SC)C=CC(=C2)F)CC(=O)O | 0 | test |
| 170466723 | C1CN(CCN1)C2=CC=CC=C2Cl | 0 | train |
| 170466722 | CCOC(=O)CN1CCCC1=O | 0 | train |
| 170466721 | CCCC(=O)NC1=CC(=C(C=C1)O)C(=O)C | 0 | validation |
| 170466720 | CC1=C(C(=NO1)C2=CC=CC=C2)C(=O)Cl | 0 | test |
| 170466719 | CC(=O)C1=CC[C@@H]2[C@@]1(CC[C@H]3[C@H]2CC=C4[C@@]3(CC[C@@H](C4)OC(=O)C)C)C | 0 | train |
| 170466718 | COC1=C(C=C(C=C1)Cl)C(=O)OC | 0 | train |
| 170466717 | CCOC(=O)N1CCC(CC1)NC2=C(C=C(C=C2)Cl)N | 0 | validation |
| 170466716 | C1CC1N2C=C(C(=O)C3=CC(=C(C=C32)Cl)F)C(=O)O | 0 | test |
| 170466715 | C1CNCCC1N2C3=CC=CC=C3NC2=O | 0 | train |
| 170466714 | CC(=CC(=O)OC)N | 0 | train |
| 170466713 | CCOC(=O)C(=O)CCCCCCl | 0 | validation |
| 170466712 | CC(C)C(=O)NC1=CC=CC(=C1)C(F)(F)F | 0 | test |
| 170466711 | C1=CC(=C(C=C1Br)F)CBr | 0 | train |
| 170466710 | C1CCN(CC1)CCOC2=CC=C(C=C2)C(=O)O.Cl | 0 | train |
| 170466709 | CCOC(=O)N1CCC(CC1)N2C3=C(C=C(C=C3)Cl)NC2=O | 0 | validation |
| 170466708 | C/C=C(/C(=C/C)/C1=CC=C(C=C1)OC(=O)C)¥C2=CC=C(C=C2)OC(=O)C | 0 | test |
| 170466707 | C1=CC=C(C(=C1)C(=O)C2=C(C=CC(=C2)Cl)N)F | 1 | train |
| 170466706 | C1=CC(=C(C=C1C(F)(F)F)N)S.Cl | 0 | train |
| 170466705 | C1C(CC(=O)NC1=O)C2=CC=C(C=C2)Cl | 0 | validation |
| 170466704 | C1=CC=C2C(=C1)C(=O)N(C(=O)N2)CCCl | 0 | test |
| 170466703 | CCOC(=O)N1CCC(=O)CC1 | 0 | train |
| 170466702 | CCN(CC)CCN | 0 | train |
| 170466701 | CCC1=CC=CC2=C1NC=C2CCO | 0 | validation |
| 170466700 | CC(C)(C)C1=CC(=CC(=C1O)C(C)(C)C)C=O | 0 | test |
| 170466699 | C1CCC2(C1)CC(=O)NC(=O)C2 | 0 | train |
| 170466698 | C1=NN=NN1CC(=O)O | 0 | train |
| 170466697 | C1CC2=C(C=CC=C2O)C(=O)C1 | 0 | validation |
| 170466696 | COC(=O)C(C(=O)OC)Cl | 0 | test |
| 170466695 | CCOC(=O)C1=CC(=C(C=C1)O)O | 1 | train |
| 170466694 | CCN1CCCC1CN | 0 | train |
| 170466693 | C1=CC=C2C(=C1)NC3=C(S2)C=CC(=C3)Cl | 1 | validation |
| 170466692 | CC(C)NC(=O)C1=CC=C(C=C1)C=O | 0 | test |
| 170466691 | C1CC2=CC=CC=C2C(=O)C3=CC=CC=C31 | 1 | train |
| 170466690 | CC(=O)CCC1=CC=C(C=C1)OC | 0 | train |
| 170466689 | CO/N=C(/C1=CSC(=N1)NC(=O)CCl)¥C(=O)O | 0 | validation |
| 170466688 | C1C2=C(C=CC(=C2)Cl)NC1=O | 1 | test |
| 170466687 | C1CNCCC1(C2=CC=C(C=C2)Cl)O | 0 | train |
| 170466686 | CCOC(=O)C(C1=CC=CC=C1)O | 0 | train |
| 170466685 | CCCC(CCC)(C(=O)OCC)C(=O)OCC | 0 | validation |
| 170466684 | CC1=C(C=CC=C1N)C(F)(F)F | 0 | test |
| 170466683 | C1CN2CCC1CC2 | 0 | train |
| 170466682 | CC(=CCOC1=C2C(=CC3=C1OC=C3)C=CC(=O)O2)C | 1 | train |
| 170466681 | CNCC(C1=CC=C(C=C1)O)O | 0 | validation |
| 170466680 | C1=CC2=C(C=CN2)C(=C1)O | 0 | test |
| 170466679 | COC1=CC(=CC(=C1OC)OC)CC#N | 0 | train |
| 170466678 | CC(=O)NC1=CC(=C(C=C1)C(=O)OC)OC | 0 | train |
| 170466677 | C1=CC=C2C(=C1)C(=O)NC3=C(O2)C=CC(=C3)Cl | 1 | validation |
| 170466676 | CCC(=O)N(C1CCNCC1)C2=CC=CC=C2 | 0 | test |
| 170466675 | C1CN(CCN1)C(=O)C2=CC=CO2 | 0 | train |
| 170466674 | C1=C(NC(=NC1=O)N)N | 0 | train |
| 170466673 | C1=CC(=CC=C1C(=O)CCCCl)F | 1 | validation |
| 170466672 | COCCOCC(OC)OC | 0 | test |
| 170466671 | COC1=C(C(=CC(=C1)S(=O)(=O)N)C(=O)O)OC | 0 | train |
| 170466670 | C1CNCCC1N2C3=C(C=C(C=C3)Cl)NC2=O | 0 | train |
| 170466669 | CCOC1=NC=C(C(=O)N1)F | 0 | validation |
| 170466668 | CCS(=O)(=O)C1=CC(=C(C=C1)OC)C(=O)OC | 0 | test |

| | | | |
|---|---|---|---|
| 170466667 | C[C@H]1C[C@@H](C2=C(S1(=O)=O)SC=C2)O | 0 | train |
| 170466666 | CC(=O)C1CCOC1=O | 0 | train |
| 170466665 | C1CC(C1)C(=O)O | 0 | validation |
| 170466664 | CC(=O)N1CCN(CC1)C2=CC=C(C=C2)O | 0 | test |
| 170466663 | COC1=CC=CC=C1N2CCNCC2.Cl | 0 | train |
| 170466662 | CCC(C)(CN(C)C)OC(=O)C1=CC=CC=C1.Cl | 0 | train |
| 170466661 | CC1=C(C(=O)N(N1C)C2=CC=CC=C2)N | 0 | validation |
| 170466660 | C1=CC(=CC=C1C(=O)O)N | 0 | test |
| 170466659 | CN1CCC=C(C1)C(=O)OC | 0 | train |
| 170466658 | CN1C2=C(C(=O)N(C1=O)C)N(C=N2)CC(=O)O | 0 | train |
| 170466657 | CC1(C(C1C(=O)OC(C#N)C2=CC(=CC=C2)OC3=CC=CC=C3)C=C(Cl)Cl)C | 1 | validation |
| 170466656 | CNC(=O)C1=CC=CC=C1SC2=CC3=C(C=C2)C(=NN3)/C=C/C4=CC=CC=N4 | 1 | test |
| 170466655 | C1C2=C(C(=CC=C2)O)C(=O)C3=C1C=CC=C3O | 0 | train |
| 170466654 | C1CN(CCN1CC(CO)O)C2=CC=CC=C2 | 0 | train |
| 170466653 | CC(=O)SC[C@@H](CC1=CC=CC=C1)C(=O)NCC(=O)OCC2=CC=CC=C2 | 0 | validation |
| 170466652 | C(=NN)(N)N.C(=NN)(N)N.OS(=O)(=O)O | 0 | test |
| 170466651 | CCOC(=O)N | 0 | train |
| 170466650 | C1=CC2=C(C=C1Cl)N(C(=O)N(C2=O)CC3=C(C=C(C=C3)Br)F)CC(=O)O | 0 | train |
| 170466649 | C1=C2C3=C(C(=C1O)O)OC(=O)C4=CC(=C(C(=C43)OC2=O)O)O | 0 | validation |
| 170466648 | C1=CC(=C[N+](=C1)[O-])CO | 0 | test |
| 170466647 | C1=CC=C2C(=C1)C(=NN2CC3=C(C=C(C=C3)Cl)Cl)C(=O)O | 0 | train |
| 170466646 | C1=CC(=CC=C1C(=O)O)OCCCOC2=CC=C(C=C2)C(=O)O | 0 | train |
| 170466645 | C1CN(CCC1C(=O)C2=CC=C(C=C2)F)CCN3C(=O)C4=CC=CC=C4NC3=O | 0 | validation |
| 170466644 | COC1=C(C=CC(=C1)C2=NNC(=O)C=C2)OC(F)F | 0 | test |
| 170466643 | COC1=C(C=C(C=C1)S(=O)(=O)N2[C@@H]([C@@](C3=C2C=CC(=C3)Cl)(C4=CC=CC=C4Cl)O)C(=O)N5CCC[C@@H]5C(=O)N)OC | 0 | train |
| 170466642 | CCNC(=O)[C@@H]1CCCN1C(=O)[C@H](CCCN=C(N)N)NC(=O)[C@H](CC(C)C)NC(=O)CNC(=O)[C@H](CC2=CC=C(C=C2)O)NC(=O)[C@H](CO)NC(=O)[C@H](CC3=CNC4=CC=CC=C43)NC(=O)[C@H](CC5=CN=CN5)NC(=O)[C@@H]6CCC(=O)N6 | 0 | train |
| 170466641 | CC1=NC=C(C=C1)CCN2C3=C(CN(CC3)C)C4=C2C=CC(=C4)Cl | 0 | validation |
| 170466640 | CCN(CC)CCOC1=CC=C(C=C1)C(CC2=CC=C(C=C2)Cl)(C3=CC=C(C=C3)C)O | 1 | test |
| 170466639 | CC1=C2C(=CS1)C(=O)NC3=CC=CC=C3N2C(=O)CN4CCN(CC4)C | 0 | train |
| 170466638 | CC1=C(N(C2=C1C=C(C=C2)C(=O)OCCN(C)C)CC3=CC=CC=C3)C | 1 | train |
| 170466637 | CN1C[C@@H]2C[C@H]1CN2C3=C(C=C4C(=C3)N(C=C(C4=O)C(=O)O)C5CC5)F | 0 | validation |
| 170466636 | C1=CC(=C(C=C1Cl)Cl)/C(=C/N2C=NC=N2)/Cl.Cl | 0 | test |
| 170466635 | CCC1=C(C(=CC=C1)CC)NC(=O)CN(CC(=O)O)CC(=O)O | 0 | train |
| 170466634 | CCCN(CCC)C(=O)CC1=C(N=C2N1C=C(C=C2)Cl)C3=CC=C(C=C3)Cl | 1 | train |
| 170466633 | CN1CC2=C(C=CC(=C2)C(=O)N3CCC(CC3)C4CCNCC4)N[C@H](C1=O)CC(=O)O | 0 | validation |
| 170466632 | CN1CC2=C(C=CC(=C2)C(=O)N3CCC(CC3)C4CCNCC4)N[C@H](C1=O)CC(=O)O | 0 | test |
| 170466631 | CN(CCCN1CCC2=CC(=C(C=C2CC1=O)OC)OC)CCC3=CC(=C(C=C3)OC)OC.Cl | 0 | train |
| 170466630 | CC1=NC=C(N1CC(C)O)[N+](=O)[O-] | 0 | train |
| 170466629 | CC1=C(C2=CC=CC=C2N1CCC(=O)O)CN3C=CN=C3 | 0 | validation |
| 170466628 | CC1=CC(=C(C=C1NC(=O)C2=CC(=CC(=C2O)I)I)Cl)C(C#N)C3=CC=C(C=C3)Cl | 0 | test |
| 170466627 | CCCCCCCCCCOCC(CN)O.Cl | 1 | train |
| 170466626 | CC(=O)N[C@@H](CO)C(=O)N[C@@H](CC(=O)O)C(=O)N[C@@H](CCCCN)C(=O)N1CCC[C@H]1C(=O)O | 0 | train |
| 170466625 | CC(=O)NC1CCSC1=O | 0 | validation |
| 170466624 | CC1=CN=C(N=C1)NS(=O)(=O)C2=CC=C(C=C2)N | 0 | test |
| 170466623 | CC1=C(C(=CC=C1)C)NC(=O)C2=CC(=C(C=C2O)Cl)S(=O)(=O)N | 0 | train |
| 170466622 | CC(C)OC(=O)C1=CC2=C(N1)C=CC=C2OCC(CNC(C)(C)C)O | 0 | train |
| 170466621 | CC1=C(C(=CC=C1)C)NC(=O)CN2CCN(CC2)CCCC(C3=CC=C(C=C3)F)C4=CC=C(C=C4)F | 0 | validation |
| 170466620 | CC(C)NCC(C1=CC(=C(C=C1)N)C#N)O | 0 | test |
| 170466619 | CC(C)NCC(COC1=CC=C(C=C1)NC(=O)C)O | 0 | train |
| 170466618 | CC1=CC=C(C=C1)N2C(=CC(=N2)C(C)(C)C)NC(=O)NC3=CC=C(C4=CC=CC=C43)OCCN5CCOCC5 | 1 | train |
| 170466617 | CC(C)CN1C=NC2=C1C(=O)N(C(=O)N2C)C | 0 | validation |
| 170466616 | CC1(C(=O)C=C(O1)C(=O)O)C2=CC=CC=C2 | 0 | test |
| 170466615 | CC(C)(C)CC(C)(C)C1=CC(=C(C=C1)O)CC2=C(C=C(C=C2)Cl)Cl | 1 | train |
| 170466614 | CC[N+](CC)(CC)CCOC1=CC=C(C=C1)/C=C/C2=CC=CC=C2.[I-] | 0 | train |
| 170466613 | CC(=O)N/N=C/C1=CC=C(O1)[N+](=O)[O-] | 0 | validation |
| 170466612 | COCCC1=CC=CC=N1 | 0 | test |
| 170466611 | C1COC2=C([C@]13C(=O)NC(=O)N3)C=C(C=C2)F | 0 | train |

| | | | |
|---|---|---|---|
| 170466610 | CC1=N[C@@]2([C@H](O1)C[C@@H]3[C@@]2(C[C@@H]([C@H]4[C@H]3CCC5=CC(=O)C=C[C@]45C)O)C)C(=O)COC(=O)C | 0 | train |
| 170466609 | C1=CC=C(C=C1)C(C2=CC=CC=C2)C(=O)C3C(=O)C4=CC=CC=C4C3=O | 0 | validation |
| 170466608 | C1=CC(=CC(=C1)S(=O)(=O)NCCNS(=O)(=O)C2=CC=CC(=C2)[N+](=O)[O-])[N+](=O)[O-] | 0 | test |
| 170466607 | C1CN(CCN1)C2=C3C(=CC=C2)OCCO3.Cl | 0 | train |
| 170466606 | CC(C)NCC(C1=CC2=CC=CC=C2C=C1)O | 0 | train |
| 170466605 | CC(=O)N[C@@H](CCC(=O)O)C(=O)O | 0 | validation |
| 170466604 | C(CSCCO)O | 0 | test |
| 170466603 | C1=CN(C(=N1)[N+](=O)[O-])CC(=O)NCCO | 0 | train |
| 170466602 | C1=CC=C(C=C1)/C=C/C(=O)NCCO | 1 | train |
| 170466601 | C1=CC(=CC=C1OC2=C(C=C(C=C2)NC(=O)C3=CC(=CC(=C3O)I)I)Cl)Cl | 0 | validation |
| 170466600 | C1=CC=C(C=C1)C(CC2=CC(=C(C(=C2)I)O)I)C(=O)O | 0 | test |
| 170466599 | C=CCN1C2=C(NC(=NC2=O)N)N(C1=O)[C@H]3[C@@H]([C@@H]([C@H](O3)CO)O)O | 0 | train |
| 170466598 | CC1=C(C=CC=C1NC2=C(C=CC=N2)C(=O)O)C(F)(F)F.CNC[C@@H]([C@H]([C@H]([C@@H](CO)O)O)O)O | 1 | train |
| 170466597 | COC1=C(C=CC(=C1)C=C2CCCC(=CC3=CC(=C(C=C3)O)OC)C2=O)O | 0 | validation |
| 170466596 | CN1C(=CN=C1COC(=O)N)[N+](=O)[O-] | 0 | test |
| 170466595 | C1CN=C(N1)C2COC3=CC=CC=C3O2 | 1 | train |
| 170466594 | CCN(CC)CCNC(=O)COC1=CC=C(C=C1)OC.Cl | 0 | train |
| 170466593 | C1CNCCC1C(C2=CC=CC=C2)(C3=CC=CC=C3)O | 0 | validation |
| 170466592 | CCOP(=S)(OCC)O/N=C(¥C#N)/C1=CC=CC=C1 | 1 | test |
| 170466591 | CCC1=CC(=C(C(=C1O)C(=O)NC[C@@H]2CCCN2CC)OC)Cl.Cl | 0 | train |
| 170466590 | CN(C)CCN1CCN(C1=O)C2=CC(=CC=C2)Cl | 0 | train |
| 170466589 | CC1=C(C(=O)N(N1C)C2=CC=CC=C2)NC(C)C | 0 | validation |
| 170466588 | CCN1C=CN=C1CC2COC3=CC=CC=C3O2 | 0 | test |
| 170466587 | CCCCCCCCCCCCOCCO | 0 | train |
| 170466586 | CC1=CC=CC=C1OCC(CO)O | 0 | train |
| 170466585 | CC1=CC=C(C=C1)S(=O)(=O)NC(=O)NC2CCCCCCC2 | 0 | validation |
| 170466584 | C1=CC(=C(C=C1N)S(=O)(=O)O)C=CC2=C(C=C(C=C2)N)S(=O)(=O)O | 0 | test |
| 170466583 | CC(C(=O)OC(C)(C)CC1=CC=C(C=C1)Cl)N | 0 | train |
| 170466582 | CC(C(=O)C1=CC=CC=C1)N(C)C | 0 | train |
| 170466581 | C([C@@H](C(=O)O)N)OP(=O)(O)O | 0 | validation |
| 170466580 | CC(C)(C)CC(C)(C)C1=CC(=C(C=C1)O)N2N=C3C=CC=CC3=N2 | 1 | test |
| 170466579 | CCCCCCCC/C=C¥CCCCCCCCN | 0 | train |
| 170466578 | C1COCCN1CCN2C=NC=C2[N+](=O)[O-] | 0 | train |
| 170466577 | C1[C@H](NCS1)C(=O)O | 0 | validation |
| 170466576 | CC1=CC=CC(=C1O)C(=O)O | 0 | test |
| 170466575 | C1=CSC(=C1)/C(=C¥2/C3=C(C=CC(=C3)Cl)N(C2=O)C(=O)N)/O | 1 | train |
| 170466574 | C1=CC(=CC=C1N)S(=O)(=O)NC2=NN=C(C=C2)Cl | 0 | train |
| 170466573 | CC(C)CCCC(C)N | 0 | validation |
| 170466572 | C[C@]12CCC(=O)C=C1CC[C@@H]3[C@@H]2CC[C@]4([C@H]3CC[C@@]4(C(=O)CO)O)C | 0 | test |
| 170466571 | CN1C[C@@H](C[C@H]2[C@H]1CC3=CN(C4=CC=CC2=C34)C)CNC(=O)OCC5=CC=CC=C5 | 0 | train |
| 170466570 | CC(C)(C)OC(=O)C1=C2[C@@H]3CCCN3C(=O)C4=C(N2C=N1)C=CC=C4Br | 0 | train |
| 170466569 | CCC(C1=CC=CC=C1)C(=O)OCCN(CC)CC | 0 | validation |
| 170466568 | CCN(CC)CCOC(=O)C(C1=CC=CC=C1)(C2=CC=CC=C2)O | 0 | test |
| 170466567 | CC1(O[C@@H]2[C@H]([C@H](O[C@@H]2O1)C(CO)O)OCCCN(C)C)C.Cl | 0 | train |
| 170466566 | CCCCCCCCCCCCCCCCCCN(C)C | 0 | train |
| 170466565 | C1=CC(=CC=C1C2=NC(=CS2)CC(=O)O)Cl | 0 | validation |
| 170466564 | C1=CC=C(C=C1)C2=NC3=CC=CC=C3C(=C2O)C(=O)O | 1 | test |
| 170466563 | C1=CC=C2C(=C1)NC3=CC=CC=C3S2 | 1 | train |
| 170466562 | C1=C(OC(=C1)[N+](=O)[O-])C=NNC(=O)C(=O)N | 0 | train |
| 170466561 | CC1=C(C(C(=C(N1)C)C(=O)OC(C)C)C2=CC(=CC=C2)[N+](=O)[O-])C(=O)OCCOC | 1 | validation |
| 170466560 | COC1=CC(=CC(=C1OC)OC)C(=S)N2CCOCC2 | 0 | test |
| 170466559 | COC1=CC=C(C=C1)C(=O)N2CCN(CC2)CC(=O)N3CCOCC3 | 0 | train |
| 170466558 | CCN1CCCC1CNC(=O)C(C2=CC=CC=C2)(C3=CC=CC=C3)O | 0 | train |
| 170466557 | CC1=NC2=C(N1)C(=O)N(C(=O)N2CC3=CC=CO3)C | 0 | validation |
| 170466556 | CN1C2=CC=CC=C2C(=N[C@@H](C1=O)NC(=O)C3=CC4=CC=CC=C4N3)C5=CC=CC=C5 | 0 | test |
| 170466555 | CN(C)[C@@H](CCOC1=CC=CC2=CC=CC=C21)C3=CC=CC=C3 | 0 | train |
| 170466554 | CC1=C(C(=O)N2C=CSC2=N1)CCN3CCC(=C(C4=CC=C(C=C4)F)C5=CC=C(C=C5)F)CC3 | 0 | train |
| 170466553 | CC1(CCCCC1)COC2=CC=C(C=C2)CC3C(=O)NC(=O)S3 | 1 | validation |
| 170466552 | CC[N+]1(CCCC(C1)OC(=O)C(C2=CC=CC=C2)(C3=CC=CC=C3)O)C.[Br-] | 0 | test |

| | | | |
|---|---|---|---|
| 170466551 | CCCCCCCCCCCCCCNCCO.CC(C(=O)O)O | 1 | train |
| 170466550 | CC(C)COC(=O)C1=CC=C(C=C1)N | 1 | train |
| 170466549 | CC1=CC=CC=C1N2CCN(CC2)CCC3=NN=C4N3CCCC4.Cl | 0 | validation |
| 170466548 | CCCCCCC[N+](CC)(CC)CCCCC1=CC=C(C=C1)Cl.CC1=CC=C(C=C1)S(=O)(=O)[O-] | 0 | test |
| 170466547 | CC1=CC(=C(C(=C1)N2N=C3C=CC(=CC3=N2)Cl)O)C(C)(C)C | 0 | train |
| 170466546 | CC(CC1=CC(=CC=C1)C(F)(F)F)NCCOC(=O)C2=CC=CC=C2.Cl | 0 | train |
| 170466545 | CC1=C([N+](=O)C2=CC=CC=C2N1[O-])C(=O)NCCO | 0 | validation |
| 170466544 | C(COCCOCCN(CC(=O)O)CC(=O)O)N(CC(=O)O)CC(=O)O | 0 | test |
| 170466543 | C[C@@H](C(=O)N[C@@H](CC(C)C)[C@H](CC(=O)O)O)NC(=O)C[C@@H]([C@H](CC(C)C)NC(=O)[C@H](C(C)C)NC(=O)[C@H](C(C)C)NC(=O)CC(C)C)O | 0 | train |
| 170466542 | CC[N+]1(CCC(C1)OC(=O)C(C2=CC=CC=C2)(C3=CC=CC=C3)O)CC.[Br-] | 0 | train |
| 170466541 | COC(=O)C1=CC=CC=C1C(=O)C2=CC=C(C=C2)OCCN3CCCCC3.Cl | 0 | validation |
| 170466540 | C1C[C@@H]([C@@H]([C@@H]2[C@@H]1CN3CCC4=C([C@@H]3C2)NC5=CC=CC=C45)C(=O)O)O.O | 0 | test |
| 170466539 | C1=CC=C(C=C1)CNC(=O)CN2C=CN=C2[N+](=O)[O-] | 0 | train |
| 170466538 | CC(CC1=CC=CC=C1)(C2=CC=CC=C2)NC(=O)CN.Cl | 0 | train |
| 170466537 | C([C@@H](C(=O)O)N)O/C(=C/[N+]#N)/[O-] | 0 | validation |
| 170466536 | C1=CC(=CN=C1)/C=N/NC(=S)N | 0 | test |
| 170466535 | CCN(CC)CCNC1=C2C(=C(C=C1)CO)SC3=CC=CC=C3C2=O | 1 | train |
| 170466534 | CC1=NC(=C(C(=N1)Cl)NC2=NCCN2)OC | 0 | train |
| 170466533 | COC1=C(C(=CC(=C1)CC=C)C(=O)NCCO)O | 0 | validation |
| 170466532 | C1=CC=C(C=C1)CCNC(=O)CCCl | 0 | test |
| 170466531 | C1C(C2(C(=C(C1(C2(Cl)Cl)Cl)Cl)Cl)Cl)CBr | 1 | train |
| 170466530 | C1=CC(=CC=C1[C@@H](C(=O)O)N)O | 0 | train |
| 170466529 | C1CN(CC1C2=CC=CC=C2)CCC(=O)C3=CC4=C(C=C3)OCCO4 | 0 | validation |
| 170466528 | CS(=O)(=O)C1=CC=C(C=C1)C2=CN3C=CC=CC3=N2 | 1 | test |
| 170466527 | CN1CCN(CC1)C2=C(C=C3C4=C2SCCN4C=C(C3=O)C(=O)O)F.Cl | 0 | train |
| 170466526 | C1=CN=CC=C1N | 0 | train |
| 170466525 | CCNC(=O)N1CCN(CC1)CCCC(C2=CC=C(C=C2)F)C3=CC=C(C=C3)F.Cl | 1 | validation |
| 170466524 | CC1CC(CC(N1C)(C)C)OC(=O)C(C2=CC=CC=C2)O.Cl | 0 | test |
| 170466523 | C1=CC(=CC2=NC3=C(C=CC(=C3)N)C=C21)N.C1=CC(=CC2=NC3=C(C=CC(=C3)N)C=C21)N.OS(=O)(=O)O | 0 | train |
| 170466522 | CCN(CC)CCC1=C(C2=C(C(=C(C=C2)OCC(=O)OCC)Cl)OC1=O)C.Cl | 0 | train |
| 170466521 | CCCCCCCCNC(C)C(C1=CC=C(C=C1)SC(C)C)O | 1 | validation |
| 170466520 | CCN(CC)CCOC(=O)C(C1=CC=CC=C1)C2=CC=CC=C2.Cl | 0 | test |
| 170466519 | CCN(CC)CCSC(=O)C(C1=CC=CC=C1)C2=CC=CC=C2 | 0 | train |
| 170466518 | CCCCCCCCCCCCCCC[C@@H]([C@H](CO)N)O | 0 | train |
| 170466517 | CC1=CN(C(=O)NC1=O)[C@H]2[C@H]([C@@H]([C@H](O2)CO)O)F | 0 | validation |
| 170466516 | CC1=C(SC=N1)CCCl | 0 | test |
| 170466515 | CC1=C(C(=CC=C1)OCC(CNC(C)(C)C)O)C | 0 | train |
| 170466514 | CC(C)(C)NCC(COC1=CC=C(C=C1)NC(=O)NC2CCCCC2)O | 1 | train |
| 170466513 | CC(=CC1C(C1(C)C)C(=O)OCN2C(=O)C3=C(C2=O)CCCC3)C | 0 | validation |
| 170466512 | C1CNCC2=C1C(=O)NO2 | 0 | test |
| 170466511 | C1=CC2=C(C(=C(C=C2S(=O)(=O)O)I)O)N=C1 | 0 | train |
| 170466510 | CCCCCC(C)N.CCCCCC(C)N.OS(=O)(=O)O | 0 | train |
| 170466509 | C1COCCN1CC2CN(C(=O)O2)N=CC3=CC=C(O3)[N+](=O)[O-].Cl | 1 | validation |
| 170466508 | CC1=CC(=O)NC(=S)N1 | 0 | test |
| 170466507 | C1=CC(=CC=C1NC(=O)N)[As](=O)(O)O | 0 | train |
| 170466506 | C[C@@H]1CCC[C@@H](N1)C.Cl | 0 | train |
| 170466505 | C([C@@H]1[C@H]([C@@H]([C@H](C(O1)O)N)O)O)O.Cl | 0 | validation |
| 170466504 | CC(CCCC(C)(C)O)N.Cl | 0 | test |
| 170466503 | CC(=O)NC1=C(C=CC(=C1)[As](=O)(O)O)O | 0 | train |
| 170466502 | C1CCC(CC1)NS(=O)(=O)O | 0 | train |
| 170466501 | CCCN1CCC[C@H]2[C@H]1CC3=C(C2)NN=C3.Cl | 0 | validation |
| 170466500 | CCN¥1C2=CC=CC=C2S/C1=C/C=C/C3=[N+](C4=CC=CC=C4S3)CC.[I-] | 0 | test |
| 170466499 | CC1=NC2=CC=CC=C2N1CC3=CC=C(C=C3)Cl | 1 | train |
| 170466498 | C1CC1NC2=NC(=NC(=N2)N)N | 0 | train |
| 170466497 | C1=C(C(=NC(=O)N1[C@H]2[C@H]([C@@H]([C@H](O2)CO)O)F)N)I | 0 | validation |
| 170466496 | C1C2CN(CC1CN(C2)C(=O)C3=CC=C(C=C3)N)CC4=CC=CC=C4 | 0 | test |
| 170466495 | C1=CC(=CC=C1C(=O)NP(=O)(N)N)F | 0 | train |
| 170466494 | C1COCCN1CNC(=O)C2=NC=CN=C2 | 0 | train |
| 170466493 | CC1=CC(=O)C2=C(C3=C(C(=C2O1)OC)OC=C3)OC | 1 | validation |

| | | | |
|---|---|---|---|
| 170466492 | COC(=O)NC1=NC2=C(N1)C=C(C=C2)C(=O)C3CC3 | 0 | test |
| 170466491 | COC1=CC=CC=C1OCC2CNC(=O)O2 | 0 | train |
| 170466490 | C[C@@H](C(=O)N1CC2=CC(=C(C=C2C[C@H]1C(=O)O)OC)OC)N[C@@H](CCC3=CC=CC=C3)C(=O)O | 0 | train |
| 170466489 | CCOC(=O)C(=C(C1=CC=CC=C1)C2=CC=CC=C2)C#N | 1 | validation |
| 170466488 | CC1=C(N(C2=C1C=C(C=C2)C(=O)OCCN(C)C)CCN(C)C)C | 1 | test |
| 170466487 | CN=C(NCCSCC1=CSC(=N1)N=C(N)N)NC#N | 0 | train |
| 170466486 | C1=CC=C(C=C1)SC2=CC=C(C=C2)COC(CN3C=CN=C3)C4=C(C=C(C=C4)Cl)Cl | 0 | train |
| 170466485 | CCCCCN(CCCCC)C(=O)C(CCC(=O)[O-])NC(=O)C1=CC(=C(C=C1)Cl)Cl.[Na+] | 0 | validation |
| 170466484 | CCCOC(=O)C1=CN=CN1C(C)C2=CC=CC=C2 | 1 | test |
| 170466483 | CNCCC(C1=CC=CC=C1)OC2=CC=CC=C2OC | 0 | train |
| 170466482 | C1CCN(CC1)CCC2OC(=O)C(O2)(C3=CC=CC=C3)C4=CC=CC=C4.Cl | 0 | train |
| 170466481 | CN(C)CCCSC1=CC=CC=C1NC(=O)/C=C/C2=CC=CC=C2 | 0 | validation |
| 170466480 | CCN1CCCC1CNC(=O)C2=CC(=C(C=C2OC)N)S(=O)(=O)CC | 0 | test |
| 170466479 | CC1([C@H]([C@@H](C2=C(O1)C=CC(=C2)C#N)N3CCCC3=O)O)C | 0 | train |
| 170466478 | CC1CCC2=C3N1C=C(C(=O)C3=CC(=C2C)F)C(=O)O | 0 | train |
| 170466477 | CCC(C)(C#C)OC(=O)C1=CC=CC=C1C(=O)O | 0 | validation |
| 170466476 | CCCCCCCCC(=O)NCC1=CC(=C(C=C1)O)OC | 0 | test |
| 170466475 | CC(=O)OC1=CC=CC2=C1C(=O)C3=C(C=C(C=C3C2=O)C(=O)O)OC(=O)C | 1 | train |
| 170466474 | CC(=O)NC(CC1=CC=CC=C1)C(=O)O | 0 | train |
| 170466473 | CC1=NC2=C(C=C1)C(=CC(=C2O)Br)Br | 1 | validation |
| 170466472 | CC(=CCNC1=NC=NC2=C1N=CN2[C@H]3[C@@H]([C@@H]([C@H](O3)CO)O)O)C | 1 | test |
| 170466471 | CC1=C(C(=O)C(=C(C1=O)C)CCCCC#CCCCC#CCO)C | 1 | train |
| 170466470 | C1=CC2=C3C(=C1)C(=O)N(C(=O)C3=CC=C2)CC(=O)O | 0 | train |
| 170466469 | COC1=C(C=CC(=C1)[C@@H]2[C@H](OC3=C(O2)C=C(C=C3)[C@@H]4[C@H](C(=O)C5=C(C=C(C=C5O4)O)O)O)CO)O | 0 | validation |
| 170466468 | C[C@]12CC[C@H]3[C@H]([C@@H]1CC[C@@H]2C(=O)CO)CCC4=CC(=O)CC[C@]34C | 0 | test |
| 170466467 | CN(C)CCOC(=O)C(C1=CC=CC=C1)(C2=CC=CC=C2)OCC#C.Cl | 1 | train |
| 170466466 | C1=CSC(=C1)C(=O)NC2=NC=C(S2)[N+](=O)[O-] | 1 | train |
| 170466465 | CCOC(C1=CC=CC=C1)(C2=CC=CC=C2)C(=O)OCCN(C)C | 1 | validation |
| 170466464 | CCN(CC)CCC1=NC(=NO1)C2=CC=CC=C2.C(C(=O)O)C(CC(=O)O)(C(=O)O)O | 1 | test |
| 170466463 | C1=CC(=CC(=C1)NC2=C(C=CC=N2)C(=O)O)C(F)(F)F | 1 | train |
| 170466462 | CN1CCC2=CC(=C(C=C2[C@@H]3[C@@H]1CCC4=CC=CC=C34)O)Cl | 0 | train |
| 170466461 | C1CN(CCN1CC2=CC3=C(C=C2)OCO3)C(=O)COC4=CC=C(C=C4)Cl.Cl | 0 | validation |
| 170466460 | CC1(C(=O)NC(=O)O1)C | 0 | test |
| 170466459 | COC1=CC(=C(C(=C1)OC)C(=O)CCCN2CCCC2)OC.Cl | 0 | train |
| 170466458 | C1=C/C(=C¥NNC(=O)C2=CC=NC=C2)/C(=O)C=C1 | 1 | train |
| 170466457 | C1C2=C(C=CC(=C2)N)N=C3N1C(=O)C4=CC=CC=C43 | 1 | validation |
| 170466456 | C1=CC(=CC=C1C(=O)OCC(CO)O)N | 0 | test |
| 170466455 | C1CN(CCC1C(=O)C2=CC=C(C=C2)F)CCN3C(=O)C4=CC=CC=C4NC3=S.Cl | 0 | train |
| 170466454 | C1CN(CCC12C(=O)NCN2C3=CC=CC=C3)CCCOC4=CC=C(C=C4)F | 0 | train |
| 170466453 | COC1=CC2=C(C=C1)N=C(S2)NC(=O)NC3=CC=CC=C3 | 1 | validation |
| 170466452 | COC1=CC(=CC(=C1OC)OC)C(=O)N2CCCCCCC2 | 0 | test |
| 170466451 | COC1=CC=C(C=C1)C=CC(=O)C2=C(C=C(C=C2)OC)OC | 1 | train |
| 170466450 | CN1CC(C2=C(C1)C(=CC=C2)N)C3=CC=CC=C3.C(=C¥C(=O)O)¥C(=O)O | 0 | train |
| 170466449 | CC1=C(C(=CC=C1)C)NC(=O)CN2CCCC2 | 0 | validation |
| 170466448 | C1CCC2=C(C1)C(=C3CCCC3=N2)N.O.Cl | 0 | test |
| 170466447 | C1=CC(=CN=C1)CN | 0 | train |
| 170466446 | CCCOC1=CC=CC=C1C2=NC(=O)C3=NNNC3=N2 | 0 | train |
| 170466445 | CCN1C2=NC=C(C(=C2C=N1)NN=C(C)C)C(=O)OCC.Cl | 0 | validation |
| 170466444 | CCCCN(CCCC)C(C1=CC=C(C=C1)OC)C(=O)N | 1 | test |
| 170466443 | CCC1=CC(=CC=C1)N(C)C(=NC2=CC=CC3=CC=CC=C32)N.Cl | 0 | train |
| 170466442 | CCC1(C(=O)C=CNC1=O)CC | 0 | train |
| 170466441 | CCC1(C(=O)N(C(=O)N(C1=O)COC)COC)C2=CC=CC=C2 | 1 | validation |
| 170466440 | CC1=C2C=CC=NC2=C(C=C1)O | 1 | test |
| 170466439 | CC(CC1C2=CC=CC=C2CCC3=CC=CC=C13)CN(C)C | 0 | train |
| 170466438 | CCC1(CC2=CC=CC=C2O1)C3=NCCN3 | 0 | train |
| 170466437 | CC(C)(CC1=CC=CC=C1Cl)N | 0 | validation |
| 170466436 | CC(=O)NC1=CC=C(C=C1)/C=N/NC(=S)N | 0 | test |
| 170466435 | C1CCC(CC1)C(CC2CCCCN2)C3CCCCC3.C(=C¥C(=O)O)¥C(=O)O | 1 | train |
| 170466434 | C1=CC=C(C=C1)N2C=C(C(=N2)C3=CC=C(C=C3)Cl)CC(=O)O | 0 | train |
| 170466433 | C1(=C(C(=O)C(=C(C1=O)O)O)O)O | 0 | validation |

| | | | |
|---|---|---|---|
| 170466432 | CN1[C@@H]2CC[C@H]1CC(C2)OC(=O)C3=CNC4=CC=CC=C43 | 0 | test |
| 170466431 | C1C(=C(N2[C@H](S1)[C@@H](C2=O)NC(=O)CC3=CC=CS3)C(=O)[O-])C[N+]4=CC=C(C=C4)C(=O)N | 0 | train |
| 170466430 | CC[C@@]12CCCN3[C@@H]1C4=C(CC3)C5=CC=CC=C5N4[C@](C2)(C(=O)OC)O | 1 | train |
| 170466429 | CC(=O)[C@]1(CC[C@@H]2[C@@]1(CC[C@H]3[C@H]2CCC4=CC(=O)CC[C@]34C)C)O | 0 | validation |
| 170466428 | CC(=O)[C@H]1CC[C@@H]2[C@@]1(CC(=O)[C@H]3[C@H]2CC[C@@H]4[C@@]3(CC[C@H](C4)O)C)C | 0 | test |
| 170466427 | CC(C)(C(=O)O)OC1=CC=C(C=C1)Cl | 0 | train |
| 170466426 | C1CN(CCC1(C2=CC(=CC=C2)C(F)(F)F)O)CCCC(=O)C3=CC=C(C=C3)F.Cl | 0 | train |
| 170466425 | CCN1CCC[C@H]1CNC(=O)C2=C(C=CC(=C2OC)Br)OC | 0 | validation |
| 170466424 | CC(=O)NCCCCCC(=O)O | 0 | test |
| 170466423 | CCCCCCCCCCCCCC[N+](C)(C)CC1=CC=CC=C1.O.O.[Cl-] | 0 | train |
| 170466422 | C1=C(OC(=C1)[N+](=O)[O-])/C=N/NC(=O)C2=CC(=CC(=C2O)[N+](=O)[O-])[N+](=O)[O-] | 1 | train |
| 170466421 | C1=CC(=C(C=C1Cl)SC2=C(C=CC(=C2)Cl)O)O | 0 | validation |
| 170466420 | C1=CC=C2C(=C1)C(OC2=O)OC(=O)C3=C(N=CC=C3)NC4=CC=CC(=C4)C(F)(F)F | 0 | test |
| 170466419 | CC1=C(NC(=O)N1)C(=O)C2=CC=C(C=C2)SC | 0 | train |
| 170466418 | COC1=CC(=C(C=C1C(=O)NC2CN3CCC2CC3)Cl)N.Cl | 0 | train |
| 170466417 | CC(=O)NCCC1=CC=CC2=C1C=C(C=C2)OC | 1 | validation |
| 170466416 | COC1=CC(=NC(=N1)O[C@H](C(=O)O)C(C2=CC=CC=C2)(C3=CC=CC=C3)OC)OC | 0 | test |
| 170466415 | COP(=S)(OC)OC1=CC(=C(C=C1Cl)Cl)Cl | 1 | train |
| 170466414 | CN1CC[C@@H]([C@@H](C1)O)C2=C(C=C(C3=C2OC(=CC3=O)C4=CC=CC=C4Cl)O)O.Cl | 0 | train |
| 170466413 | CN1CCN(CC1)C2=NC3=C(CC4=CC=CC=C42)C=CC(=C3)F | 1 | validation |
| 170466412 | CN1CCN(CC1)C2=NN=C3C(=C2)N(C4=CC=CC=C4O3)C | 1 | test |
| 170466411 | CCCCN1CCCC1C(=O)NC2=C(C=C(C=C2C)C)C | 1 | train |
| 170466410 | CC1=C(C(=CC=C1)C)NC(=O)NC(=NC)N | 0 | train |
| 170466409 | C1CN(CCC1N2C3=CC=CC=C3NC2=O)CCCC(=O)C4=CC=C(C=C4)F | 0 | validation |
| 170466408 | C1=CC=C(C=C1)CCNCCCCCCNCCC2=CC(=C(C=C2)O)O | 0 | test |
| 170466407 | C1CC2CN(CCN2C1)CCC(=O)N3C4=CC=CC=C4SC5=C3C=C(C=C5)Cl | 0 | train |
| 170466406 | CCNC(=O)NCC1=CC(=CC=C1)Cl | 0 | train |
| 170466405 | CC1=CC(=CC(=C1OCCCC2=CC(=NO2)C)C)C3=NOC(=N3)C(F)(F)F | 1 | validation |
| 170466404 | CCC(C1=CC=CC=C1)C(=O)OCCOCCN(CC)CC.C(C(=O)O)C(CC(=O)O)(C(=O)O)O | 0 | test |
| 170466403 | C[C@]12CC[C@H]3[C@H]([C@@H]1CC[C@@H]2O)CCC4=C3C=CC(=C4)OC(=O)N(CCCl)CCCl | 0 | train |
| 170466402 | CCCCC1=CC2=C(C=C1)N=C(N2)NC(=O)OC | 1 | train |
| 170466401 | CCCCCCCC/C=C¥CCCCCCCC(=O)NCC1=CC(=C(C=C1)O)OC | 0 | validation |
| 170466400 | CC(=O)NC(=O)C1=CC=CC=C1O | 0 | test |
| 170466399 | CC1=NC2=C(C=C(C=C2)NC(=O)NC3=CC4=C(C=C3)N=C(C=C4N)C)C(=C1)N.Cl.Cl | 0 | train |
| 170466398 | C1C[C@@H](CN(C1)CCC2=CC3=C(C=C2)OCO3)OC(C4=CC=CC=C4)C5=CC=CC=C5.C(=C/C(=O)O)¥C(=O)O | 0 | train |
| 170466397 | CC1=C(C(=CC=C1)NC2=CC(=NC(=N2)SCC(=O)O)Cl)C | 0 | validation |
| 170466396 | CC1=CC(=C(N1)C=C2C3=CC=CC=C3NC2=O)C | 1 | test |
| 170466395 | CC1=CC(=C2C3=C4C(=CC(=C13)O)C(C(=O)C5=C(C=C(C(=C45)C2=O)O)O)(C)C)O | 1 | train |
| 170466394 | COC1=C(C=C(C=C1)CC2=CN=C(N=C2N)N)OC | 0 | train |
| 170466393 | C1=CC=C2C(=C1)C(C(=O)N2)(C3=CC=C(C=C3)O)C4=CC=C(C=C4)O | 0 | validation |
| 170466392 | C1=CC=C2C(=C1)C(=O)C(C2=O)C3=CC=C(C=C3)Br | 1 | test |
| 170466391 | C1=CC2=C(C=C1Cl)SNC2=O | 0 | train |
| 170466390 | CC1=CC2=C(C=C1N)SC3=CC(=[N+](C)C)C=CC3=N2.[Cl-] | 1 | train |
| 170466389 | CC(C)C(=O)NC1=CC=CC(=C1)C2CN3CCSC3=N2 | 0 | validation |
| 170466388 | CC1(C2=CC=CN2C3=CC=CC=C3CO1)CN4CCC(CC4)N5C6=CC=CC=C6NC5=O | 0 | test |
| 170466387 | C1=CC=C2C(=C1)C(=NN=C2NN)NN | 1 | train |
| 170466386 | CCOC1=NC2=CC=CC(=C2N1CC3=CC=C(C=C3)C4=CC=CC=C4C5=NNN=N5)C(=O)O | 0 | train |
| 170466385 | C1=CC=C(C=C1)NNC(=O)N | 0 | validation |
| 170466384 | C1=CC(=CC=C1C(=O)O)S(=O)(=O)N(Cl)Cl | 0 | test |
| 170466383 | C1=CC=C2C(=C1)C(=O)C=C(O2)C(=O)O | 0 | train |
| 170466382 | C1=CC(=CC=C1C(=O)NN=CC2=CC=C(O2)[N+](=O)[O-])O | 1 | train |
| 170466381 | C1=CC=C2C(=C1)NC(=N2)CCC(=O)O | 0 | validation |
| 170466380 | C1CN(CCN1CCCCN2C(=O)C3=CC=CC=C3S2(=O)=O)C4=NC=CC=N4 | 0 | test |
| 170466379 | C1=CC=C(C=C1)P(=O)(CC(=O)NN)C2=CC=CC=C2 | 0 | train |
| 170466378 | C1=CC=C(C=C1)NS(=O)(=O)C2=CC=C(C=C2)N | 0 | train |
| 170466377 | C12(C(C(C(C(C1(F)F)(F)F)(F)F)(F)F)(C(C(C(C2(F)F)(F)F)(F)F)(F)F)F)F | 0 | validation |

| | | | |
|---|---|---|---|
| 170466376 | C([C@@H]1[C@@H]2[C@@H]([C@H]([C@H](O1)O[C@@H]3[C@H](O[C@@H]([C@@H]([C@H]3O)O)O[C@@H]4[C@H](O[C@@H]([C@@H]([C@H]4O)O)O[C@@H]5[C@H](O[C@@H]([C@@H]([C@H]5O)O)O[C@@H]6[C@H](O[C@@H]([C@@H]([C@H]6O)O)O[C@@H]7[C@H](O[C@@H]([C@@H]([C@H]7O)O)O[C@@H]8[C@H](O[C@H](O2)[C@@H]([C@H]8O)O)CO)CO)CO)CO)CO)CO)O)O)O)O | 0 | test |
| 170466375 | COP(=S)(OC)OC1=CC=C(C=C1)SC2=CC=C(C=C2)OP(=S)(OC)OC | 0 | train |
| 170466374 | COC1=NN=C(C=C1)NS(=O)(=O)C2=CC=C(C=C2)N | 0 | train |
| 170466373 | COP(=O)(C(C1=CC=C(C=C1)Cl)OP(=O)(OC)OC)OC | 0 | validation |
| 170466372 | COC1=CC(=CC(=C1)O)O | 0 | test |
| 170466371 | C1=CC=C(C=C1)CNC(=O)CCCl | 0 | train |
| 170466370 | CC(C)(C)NCC(C1=CC(=CC(=C1)OC(=O)N(C)C)OC(=O)N(C)C)O.Cl | 0 | train |
| 170466369 | CC1=NC=C(N1C)[N+](=O)[O-] | 0 | validation |
| 170466368 | CN1CCC(C(C1)C(=O)C2=CC=C(C=C2)F)(C3=CC=C(C=C3)F)O | 0 | test |
| 170466367 | CN1CCN2C3=C(C=C(C=C3)OC)C4=C2C1=NCC4 | 0 | train |
| 170466366 | CN(C)C/C=C(/C1=CC=C(C=C1)Br)¥C2=CN=CC=C2.O.Cl.Cl | 0 | train |
| 170466365 | CCCCCCN1C(=O)C2=C(N=CN2C)N(C1=O)C | 1 | validation |
| 170466364 | CC[C@]12C=CC3=C4CCC(=O)C=C4CC[C@H]3[C@@H]1CC[C@]2(C#C)O | 0 | test |
| 170466363 | CCC1(CCCCN(C1)C)C2=CC(=CC=C2)O.Cl | 0 | train |
| 170466362 | CC1=C(C(=CC=C1)C)NC(=O)C2=CC=C(C=C2)N | 0 | train |
| 170466361 | CCC(CC)(CC(=O)NC1=CC=CC(=C1)/C=C/C2=NC(=CS2)C3CCC3)C(=O)O | 1 | validation |
| 170466360 | CC1=CC(=C(N1C2=CC=C(C=C2)Cl)C)CC(=O)O | 0 | test |
| 170466359 | CC1=C(C(CCC1)(C)C)/C=C/C(=C/C=C/C(=C/C(=O)NC2=CC=C(C=C2)O)/C)/C | 1 | train |
| 170466358 | CC(CN(CCN(CC(C)O)CC(C)O)CC(C)O)O | 0 | train |
| 170466357 | CC(C)NNC(=O)C1=CC=NC=C1.OS(=O)(=O)O | 0 | validation |
| 170466356 | CC(C)(C(=O)O)OC1=CC=C(C=C1)C2CC2(Cl)Cl | 0 | test |
| 170466355 | CC(=O)N1C[C@@H](C[C@H]1C(=O)O)O | 0 | train |
| 170466354 | CC(C)(C)C(=O)NCCCC(=O)O | 0 | train |
| 170466353 | C[N+]1=C2C(=C3C=CC4=C(C3=C1)OCO4)C=CC5=CC6=C(C=C52)OCO6 | 1 | validation |
| 170466352 | CC(CC1=CC=CC=C1)N(C)CC2=CC=CO2 | 0 | test |
| 170466351 | C/C(=C¥C(=O)O)/C1=C(C=C(C=C1)OC)OC | 0 | train |
| 170466350 | C[C@@H]1[C@@H]2[C@H](C(=O)N2C(=C1S[C@H]3C[C@H](NC3)C(=O)N(C)C)C(=O)O)[C@@H](C)O | 0 | train |
| 170466349 | C[C@H]1C[C@@H](C(=O)[C@@H](C1)[C@@H](CC2CC(=O)NC(=O)C2)O)C | 0 | validation |
| 170466348 | C[C@]12CC[C@H]3[C@H]([C@@H]1CC[C@@H]2O)CCC4=CC(=O)CC[C@H]34 | 0 | test |
| 170466347 | C1CC(=O)N[C@@H]1C(=O)O | 0 | train |
| 170466346 | CCOC1=C(C=C(C=C1)CC2=NC=CC3=CC(=C(C=C32)OCC)OCC)OCC.Cl | 0 | train |
| 170466345 | C1=CC=C2C(=C1)C=C(N2CC(=O)O)C(=O)NC3=NC(=CS3)C4=CC=CC=C4Cl | 1 | validation |
| 170466344 | C1=CC=NC(=C1)CO | 0 | test |
| 170466343 | C1=CC=C2C(=C1)C(=CN2)CCNC(=O)C3=CN=CC=C3 | 1 | train |
| 170466342 | C1=CC=C(C=C1)N2C3=CC=CC=C3C(C2=O)(CC4=CC=NC=C4)CC5=CC=NC=C5 | 0 | train |
| 170466341 | C1=CC(=C(C(=C1)Cl)CN2C=NC3=C2N=CN=C3N)F | 0 | validation |
| 170466340 | C1COCCN1S(=O)(=O)C2=CC=C(C=C2)NC(=O)C3=C(C=CC(=C3)Cl)NS(=O)(=O)C4=CC=C(S4)Cl | 0 | test |
| 170466339 | CN1CCN(CC1)C2CC3=CC=CC=C3SC4=C2C=C(C=C4)SC | 0 | train |
| 170466338 | COC1=C(C(=C(C=C1)CCN(CCC2=C(C(=C(C=C2)OC)OC)OC)C(=O)C(C3=CC=CC=C3)C4=NCC5=CC(=C(C=C54)OC)OC)OC)OC.Cl | 0 | train |
| 170466337 | COC1=C(C(=NC=C1)CS(=O)C2=NC3=C(N2)C=C(C=C3)OC(F)F)OC | 0 | validation |
| 170466336 | CN1CCN(CC1)C(=O)C2CCCCC2 | 0 | test |
| 170466335 | CCN(CC)S(=O)(=O)C1=CC=C(C=C1)C(=O)O | 0 | train |
| 170466334 | CC1=CC(=NO1)NC(=O)C2=C(C3=CC=CC=C3S(=O)(=O)N2C)O | 0 | train |
| 170466333 | CCN1CCC2=C(CC1)OC(=N2)N.Cl.Cl | 0 | validation |
| 170466332 | CCN(CC)CC1CCCCN1CC(=O)N2C3=CC=CC=C3C(=O)NC4=C2N=CC=C4 | 0 | test |
| 170466331 | C1COCCN1CCCNC(=O)C2=CC=C(C=C2)Cl | 0 | train |
| 170466330 | CCC(=O)O.CCC(=O)O.C1CN=C(N1)C2=CC(=CC=C2)NC(=O)NC3=CC=CC(=C3)C4=NCCN4 | 0 | train |
| 170466329 | CCOCCN1C2=CC=CC=C2N=C1C3CCN(CC3)CCC4=CC=C(C=C4)C(C)(C)C(=O)O | 0 | validation |
| 170466328 | CC1=C(C(=O)OC2=C1C=CC(=C2)OP(=O)(OCCCl)OCCCl)Cl | 0 | test |
| 170466327 | CCCCCCCCOC1=CC(=C(C=C1)C(=O)C2=CC=CC=C2)O | 0 | train |
| 170466326 | CC(C)(C)C1CCC(CC1)CC2=C(C3=CC=CC=C3C(=O)C2=O)O | 0 | train |
| 170466325 | CC1=C(C(=CC=C1)C)NC(=O)CN(CC(=O)O)CC(=O)O | 0 | validation |
| 170466324 | CC1=CN(C(=O)NC1=O)[C@H]2C[C@@H]([C@H](O2)CO)F | 0 | test |
| 170466323 | CC=CC=NNC(=O)C1=CC=NC=C1 | 0 | train |
| 170466322 | CC1=CC=C(C=C1)S(=O)(=O)NC(=O)NC2CCCCC2 | 0 | train |

| | | | |
|---|---|---|---|
| 170466321 | CC¥1=C(C2=C(/C1=C¥C3=CC=C(C=C3)S(=O)(=O)C)C=CC(=C2)F)CC(=O)O | 0 | validation |
| 170466320 | CC(C)(CC1=CNC2=CC=CC=C21)NCC(COC3=CC=CC=C3C#N)O | 0 | test |
| 170466319 | C1COCCN1CCCC2=CC=C(C=C2)COC3=CC=CC=C3 | 1 | train |
| 170466318 | C1=CC=C(C=C1)C2=NC3=CC=CC=C3C(=C2)C(=O)O | 1 | train |
| 170466317 | CC(=O)[C@H]1CC[C@@H]2[C@@]1(CC[C@H]3[C@H]2CC[C@@H]4[C@@]3(CC[C@@](C4)(C)O)C)C | 0 | validation |
| 170466316 | C[C@]12CCC(=O)C=C1CC[C@@H]3[C@@H]2C(=O)C[C@]4([C@H]3CC[C@@]4(C(=O)CO)O)C | 0 | test |
| 170466315 | CCCOC1=CC2=C(C=C1)[C@H]3CC[C@]4([C@H]([C@@H]3CC2)CCC4OC)C | 0 | train |
| 170466314 | C1CC1C2=CC=CC=C2OCC3=NCCN3 | 0 | train |
| 170466313 | C1CN(CCN1CCC(=O)N2C3=CC=CC=C3SC4=C2C=C(C=C4)C(F)(F)F)CCO | 0 | validation |
| 170466312 | CN1C2=C(C(=O)N(C1=O)C)N(C=N2)CC3OCCO3 | 0 | test |
| 170466311 | CN1CCC(=C2C3=C(CCC4=CC=CC=C42)SC=C3)CC1 | 0 | train |
| 170466310 | C1=CC(=CC=C1CC(C(=O)O)N)Cl | 0 | train |
| 170466309 | C1CC(C2=C(C3=CC=CC=C3N=C2C1)N)O.C(=C¥C(=O)O)¥C(=O)O | 0 | validation |
| 170466308 | C1=CC2=C(C=C1O)C(=CN2)C[C@@H](C(=O)O)N | 0 | test |
| 170466307 | C1CN(CCC12C(=O)NCN2C3=CC=CC=C3)CCCC(C4=CC=C(C=C4)F)C5=CC=C(C=C5)F | 1 | train |
| 170466306 | CC(C)OC(=O)/C=C(¥C)/C=C/CC(C)CCCC(C)(C)OC | 0 | train |
| 170466305 | CN(C)CCN(CC1=CC=C(C=C1)Cl)C2=CC=CC=N2.Cl | 0 | validation |
| 170466304 | CC1=CC=CC=C1C(=O)NC2=CC=C(C=C2)C(=O)N3CCCC(C4=CC=CC=C43)N(C)C | 0 | test |
| 170466303 | CN1CCC(CC1)N(CC2=CC=CC=C2)C3=CC=CC=C3 | 0 | train |
| 170466302 | CCOP(=S)(OCC)OC1=CC2=C(C=C1)C(=C(C(=O)O2)Cl)C | 1 | train |
| 170466301 | C1CNCCC1SC2=NC(=CC=C2)Cl | 0 | validation |
| 170466300 | CCOC1=CC=CC=C1OCC2CNCCO2.Cl | 0 | test |
| 170466299 | CCN(CC)CCNC(=O)C1=CC(=C(C=C1OC)N)Br | 0 | train |
| 170466298 | CCC1=CC2=CC(=C(C=C2C(=N1)CC3=CC=CC=C3)OC)OC | 1 | train |
| 170466297 | CN(CCCOC1=C(C=C(C=C1)Cl)Cl)CC#C.Cl | 1 | validation |
| 170466296 | CCCN1CCC[C@H]2[C@H]1CC3=CN=C(N=C3C2)N | 1 | test |
| 170466295 | CCN(CC)C(=O)C1=CC(=C(C=C1)O)OC | 0 | train |
| 170466294 | CC(=C[C@@H]1[C@H](C1(C)C)C(=O)OCC2=COC(=C2)CC3=CC=CC=C3)C | 0 | train |
| 170466293 | CCOC(=O)NC(C(Cl)(Cl)Cl)O | 0 | validation |
| 170466292 | CCN(CC)CCNC(=O)COC1=CC=C(C=C1)Cl | 0 | test |
| 170466291 | CCCS(=O)C1=CC2=C(C=C1)N=C(N2)NC(=O)OC | 1 | train |
| 170466290 | CCN1C=C(C(=O)C2=CC(=C(C=C21)N3CCN(CC3)C)F)C(=O)O.CS(=O)(=O)O | 0 | train |
| 170466289 | CCN(CC)CCOC1=CC=C(C=C1)CC2=CC=CC=C2 | 0 | validation |
| 170466288 | CC1CCN(CC1)CCCC(=O)C2=CC=C(C=C2)F | 0 | test |
| 170466287 | CCC1=CC=C(C=C1)C2CCNCC2 | 0 | train |
| 170466286 | CC1=C(C(=CC=C1)C)NC(=O)CN2CCCC2=O | 0 | train |
| 170466285 | CC1=C(OC2=C(C1=O)C=CC=C2C(=O)OCCN3CCCCC3)C4=CC=CC=C4.Cl | 0 | validation |
| 170466284 | CCC1=NN=C(S1)NS(=O)(=O)C2=CC=C(C=C2)N | 0 | test |
| 170466283 | CC(CC(C1=CC=CC=C1)(C2=CC=CC=C2)C(=O)N)N(C)C.OS(=O)(=O)O | 0 | train |
| 170466282 | CC1(C2=C(C=CC(=C2)Br)NC(=O)O1)C | 0 | train |
| 170466281 | CC1=C(C(=O)N2C=CC=CC2=N1)CCN3CCC(CC3)C(=O)C4=CC=C(C=C4)F | 0 | validation |
| 170466280 | CC(C)CCCC(C)NCCC(C)C | 0 | test |
| 170466279 | CC(=O)NC1=CC(=CC=C1)O | 0 | train |
| 170466278 | C1CC1C(C2CC2)NC3=NCCO3 | 0 | train |
| 170466277 | C1=CC(=CC=C1N)S(=O)(=O)NC(=O)N | 0 | validation |
| 170466276 | CCCCCCCCCCCCCC[N+](C)(C)C.[Br-] | 0 | test |
| 170466275 | CC(C1=NCCN1)OC2=C(C=CC=C2Cl)Cl | 0 | train |
| 170466274 | C(C(=O)O)P(=O)(O)O | 0 | train |
| 170466273 | CCN1C=C(C(=O)C2=CC3=C(C=C21)OCO3)C(=O)O | 0 | validation |
| 170466272 | CN1CC(=O)N=C1NC(=O)NC2=CC(=CC=C2)Cl | 1 | test |
| 170466271 | C[C@H]([C@@H]1[C@@H]2N(C1=O)C(=C(S2)[C@H]3CCCO3)C(=O)O)O | 0 | train |
| 170466270 | CCC(C1=CC=C(C=C1)N2CC3=CC=CC=C3C2=O)C(=O)O | 1 | train |
| 170466269 | C1=CC=C(C=C1)NC2=NC=NC(=N2)N | 1 | validation |
| 170466268 | C1=C(C=C(C(=C1Cl)O)S(=O)C2=CC(=CC(=C2O)Cl)Cl)Cl | 0 | test |
| 170466267 | C1=CC=C(C(=C1)C(=C2C=C(C(=O)C(=C2)I)I)C3=CC(=C(C(=C3)I)[O-])I)C(=O)[O-].[Na+].[Na+] | 0 | train |
| 170466266 | C1=CC=C(C(=C1)C(=O)OCCOCCO)NC2=CC=CC(=C2)C(F)(F)F | 0 | train |
| 170466265 | CN(C(=O)CCC1=NN(C(=C1)C2=CC=C(C=C2)Cl)C3=CC=C(C=C3)OC)O | 1 | validation |
| 170466264 | C1CN(CCC12C(=O)NCN2C3=CC=CC=C3)CC4COC5=CC=CC=C5O4 | 0 | test |
| 170466263 | C1=CC=C(C=C1)CNS(=O)(=O)C2=CC=C(C=C2)N | 0 | train |

| | | | |
|---|---|---|---|
| 170466262 | CC1=C(C(C(=C(N1)C)C(=O)OC)C2=CC=CC=C2OC(F)F)C(=O)OC | 1 | train |
| 170466261 | COC(=O)N=N/C=C/1¥C=[N+](C2=CC=CC=C2N1O)[O-] | 0 | validation |
| 170466260 | CC(C)CC1=NN=C(S1)NS(=O)(=O)C2=CC=C(C=C2)OC | 0 | test |
| 170466259 | CC1=C(N=C(O1)C2=CC=CC=C2)CCOC3=CC=C(C=C3)CN(CC(=O)O)C(=O)OC4=CC=C(C=C4)OC | 0 | train |
| 170466258 | C[C@H]1[C@@H]([C@H]([C@H]([C@@H](O1)OC[C@@H]2[C@H]([C@@H]([C@H]([C@@H](O2)OC3=CC(=C4C(=C3)OC(=CC4=O)C5=CC(=C(C=C5)OC)O)O)O)O)O)O)O)O | 0 | train |
| 170466257 | COC1=CC(=CC(=C1OC)OC)C(=O)N2CCOCC2 | 0 | validation |
| 170466256 | CN1C2=NC=NC3=C2C(=CN3[C@H]4[C@@H]([C@@H]([C@H](O4)CO)O)O)C(=N1)N | 1 | test |
| 170466255 | CN([C@H]1CC[C@@]2(CCCO2)C[C@@H]1N3CCCC3)C(=O)CC4=CC(=C(C=C4)Cl)Cl | 0 | train |
| 170466254 | CN1C2=CC=CC=C2C(=C(C1=O)C(=O)N(C)C3=CC=CC=C3)O | 0 | train |
| 170466253 | COC(=O)[C@H](CC1=CC(=C(C=C1)O)O)N.Cl | 0 | validation |
| 170466252 | CNC(=O)NC(C(Cl)(Cl)Cl)O | 0 | test |
| 170466251 | CN1CCN(CC1)C2CC3=CC=CC=C3SC4=C2C=C(C=C4)Cl.C(=C¥C(=O)O)¥C(=O)O | 0 | train |
| 170466250 | CCCC1=C(C=CC(=C1O)C(=O)C)OCCCCC2=NNN=N2 | 0 | train |
| 170466249 | CCCCNC(=O)NS(=O)(=O)C1=CC=C(C=C1)N | 0 | validation |
| 170466248 | CCN(CCCC1=CC=CC=C1)CCCC2=CC=CC=C2.C(C(=O)O)C(CC(=O)O)(C(=O)O)O | 0 | test |
| 170466247 | CNCC(=O)C1=CC(=C(C=C1)O)O | 0 | train |
| 170466246 | CCOC(=O)NC1=C(N=C(C=C1)NCC2=CC=C(C=C2)F)N.C(=C¥C(=O)O)¥C(=O)O | 0 | train |
| 170466245 | CCC(=O)NC1=CC=C(C=C1)O | 0 | validation |
| 170466244 | CCCCCCCCCCOC1=C(C=C2C(=C1)C(=O)C(=CN2)C(=O)OCC)OCC | 0 | test |
| 170466243 | CC1=NC=C(N1CC(CCl)O)[N+](=O)[O-] | 0 | train |
| 170466242 | C1CN(CCN1CC2=CC3=C(C=C2)OCO3)C4=NC=CC=N4 | 0 | train |
| 170466241 | C[C@@H]1C[C@H]2[C@@H]3C[C@@H](C4=CC(=O)C(=C[C@@]4([C@]3([C@H](C[C@@]2([C@]1(C(=O)CO)O)C)O)F)C)Cl)F.O | 0 | validation |
| 170466240 | CCCCC(CC)CN=C(N)NC(=NCCCCCCN=C(N)NC(=NCC(CC)CCCC)N)N.Cl.Cl | 1 | test |
| 170466239 | C1=C(C(=CC(=C1O)O)O)CCN.Cl | 0 | train |
| 170466238 | C1CN(CC2=CC=CC=C21)C(=N)N.C1CN(CC2=CC=CC=C21)C(=N)N.OS(=O)(=O)O | 0 | train |
| 170466237 | CC(=O)NC1=CC=C(C=C1)OC(=O)C2=CC=CC=C2OC(=O)C | 0 | validation |
| 170466236 | C1=CC=C(C=C1)CCNC2=CC=CC=C2C(=O)O | 1 | test |
| 170466235 | C1=CC=C(C=C1)CN/C=C/2¥C=CC=CC2=O | 0 | train |
| 170466234 | C1=CC(=C(C(=C1C(=O)C2=CC(=C(C(=C2)O)O)O)O)O)O | 0 | train |
| 170466233 | C1CN(CCC1NC(=O)C2=CC=CC=C2)CCC3=CNC4=CC=CC=C43.Cl | 0 | validation |
| 170466232 | C1=CC(=CC=C1C(=O)O)S(=O)(=O)N | 0 | test |
| 170466231 | C(C#N)C(=O)NN | 0 | train |
| 170466230 | C1=CC=C(C=C1)CCN=C(N)N=C(N)N.Cl | 0 | train |
| 170466229 | C1=C(C(=CC(=C1N)S(=O)(=O)N)S(=O)(=O)N)C(=C(Cl)Cl)Cl | 0 | validation |
| 170466228 | C[C@H]1C[C@H]([C@@](O[C@@H]1[C@H]2C[C@@H]([C@@H](O2)[C@@]3(CC[C@@H](O3)[C@@]4(CC[C@@]5(O4)C[C@@H]([C@H]([C@H](O5)[C@@H](C)[C@H]6[C@@H]([C@H]([C@@H]([C@](O6)(CC(=O)[O-])O)C)OC)OC)C)O)C)C)OC7C[C@@H]([C@H]([C@@H](O7)C)OC)OC)(C)O)C.[NH4+] | 1 | test |
| 170466227 | C#CC1=CC=C(C=C1)C2=CC=CC=C2F | 0 | train |
| 170466226 | COP(=O)(OC)OC=C(Cl)Cl | 0 | train |
| 170466225 | COC1=CC(=CC(=C1OC)OC)C(=O)NCCCCCC(=O)O | 0 | validation |
| 170466224 | COC1=C(C=C(C=C1)/C=N/NC(=O)C2=CC=NC=C2)OC | 0 | test |
| 170466223 | COC(=O)NC1=NC2=C(N1)C=C(C=C2)S(=O)C3=CC=CC=C3 | 1 | train |
| 170466222 | CN1CCN(CC1)CCC(=O)N2C3=CC=CC=C3SC4=C2C=C(C=C4)C(F)(F)F | 0 | train |
| 170466221 | COC1=CC=CC=C1N2CCN(CC2)CC(COC3=CC(=C(C(=C3)OC)OC)OC)O | 0 | validation |
| 170466220 | CN(C1CCCCC1)C(=O)CCCOC2=CC3=C(C=C2)NC(=O)C=C3 | 0 | test |
| 170466219 | C1CN(CCC12CNC(=O)O2)CCC3=CC=CC=C3.Cl | 0 | train |
| 170466218 | CCN1CCC[C@H]1CNC(=O)C2=C(C(=CC(=C2OC)Cl)Cl)O | 0 | train |
| 170466217 | CCOC(=O)C1=C(N(C2=C1C=C(C=C2)O)C)C | 0 | validation |
| 170466216 | C[C@@H]1CN(C[C@@H](N1)C)CCCN2C3=CC=CC=C3C4=CC=CC=C42.Cl.Cl | 1 | test |
| 170466215 | CCN(CC)CC(=O)OC1=CC=C(C=C1)NC(=O)C | 0 | train |
| 170466214 | CCN(CC)CC(=O)NC1=C(C=C(C=C1C)C)C.Cl | 1 | train |
| 170466213 | CCCCCC(=O)N | 0 | validation |
| 170466212 | CC1=COC2=CC=CC=C2C1=O | 0 | test |
| 170466211 | CC1=C(N=CN1)C2=CC=C(C=C2)Cl | 1 | train |
| 170466210 | CC(C)NCC(C1=CC=C(C=C1)[N+](=O)[O-])O | 0 | train |
| 170466209 | CC(C)C[C@@H](C(=O)O)NC(=O)C | 0 | validation |
| 170466208 | CC1=CC[C@@H](CC1)[C@@](C)(CCC=C(C)C)O | 0 | test |
| 170466207 | CC(=O)NC1=CC=C(C=C1)C(=O)O | 0 | train |

| | | | |
|---|---|---|---|
| 170466206 | C[C@]12CCC(=O)C=C1C=C[C@@H]3[C@@H]2CC[C@]4([C@H]3CC[C@@]45CCC(=O)O5)C | 0 | train |
| 170466205 | CC[C@@]12CCCN3[C@@H]1C4=C(CC3)C5=CC=CC=C5N4C(=O)C2 | 1 | validation |
| 170466204 | C[C@@H]1CC[C@H]2[C@H](C(=O)O[C@H]3C24[C@H]1CCC(O3)(OO4)C)C | 1 | test |
| 170466203 | C[C@]12CCC(=O)C(=C1CC[C@@H]3[C@@H]2CC[C@]4([C@H]3CCC4=O)C)O | 0 | train |
| 170466202 | CC1=CC2=C(C=C1)NC3C2CN(CC3)C | 0 | train |
| 170466201 | C1=CC=C(C=C1)C(=O)NC2=CC(=C(C=C2)C(=O)O)O | 0 | validation |
| 170466200 | CC[N+](CC)(CC)CC.[Cl-] | 0 | test |
| 170466199 | C1=CC(=CC=C1CCNS(=O)(=O)C2=CC=C(C=C2)Cl)CC(=O)O | 0 | train |
| 170466198 | C1CCN(C1)CC#CC(C2=CC=CC=C2)(C3=CC=CC=C3)O | 1 | train |
| 170466197 | C1CN(CCC1(C2=CC(=C(C=C2)Cl)C(F)(F)F)O)CCCC(C3=CC=C(C=C3)F)C4=CC=C(C=C4)F | 1 | validation |
| 170466196 | C1CN(CC=C1C2=CC=CC=C2)CCCCC3=CNC4=C3C=C(C=C4)C(=O)O | 0 | test |
| 170466195 | C1CCC(CC1)NC(=O)CN2CCNCC2 | 0 | train |
| 170466194 | C1CN(CCN1CC2=CC3=C(C=C2)OCO3)CC(=O)N4C5=CC=CC=C5SC6=CC=CC=C64 | 1 | train |
| 170466193 | C1=CC=C(C=C1)N2C(=NN=N2)N | 0 | validation |
| 170466192 | C1=CC=C(C(=C1)N)NC(=O)C2=CC=C(C=C2)N | 0 | test |
| 170466191 | C1=CC(=C(C=C1[N+](=O)[O-])Cl)C(=O)N | 0 | train |
| 170466190 | CC(=O)N[C@H](COC)C(=O)NCC1=CC=CC=C1 | 0 | train |
| 170466189 | CC1=CN=C(C(=C1OC)C)CSC2=NC3=C(N2)C=C(C=C3)OC | 0 | validation |
| 170466188 | COCCCN1CCC(CC1)NC(=O)C2=CC(=C(C3=C2OCC3)N)Cl | 0 | test |
| 170466187 | CCC1(CC2=CC=CC=C2C1)C3=CN=CN3 | 0 | train |
| 170466186 | CCCOC(=O)C1=CC=C(C=C1)N | 1 | train |
| 170466185 | C1CN(CCN1)C2=C(C=C3C(=C2)N(C=C(C3=O)C(=O)O)C4=CC=C(C=C4)F)F.Cl | 0 | validation |
| 170466184 | C1COCCN1CCOC(=O)C2=C(N=CC=C2)NC3=CC=CC(=C3)C(F)(F)F | 0 | test |
| 170466183 | CCCC1=NC=C(S1)C(=O)O | 0 | train |
| 170466182 | C[C@]12CCC(=O)C=C1CC[C@@H]3[C@@H]2CC[C@]4([C@H]3CC[C@]4(C#C)O)C | 0 | train |
| 170466181 | CC(=O)NC1=CC=C(C=C1)OCCOCCOC2=CC=C(C=C2)NC(=O)C | 0 | validation |
| 170466180 | CC(=O)NC1=NC=C(S1)[N+](=O)[O-] | 0 | test |
| 170466179 | CC1=CC(=O)OC2=C1C=CC(=C2)O[C@H]3[C@@H]([C@H]([C@@H](CS3)O)O)O | 0 | train |
| 170466178 | C1=CC=C(C(=C1)C(=O)NC2=CC=C(C=C2)S(=O)(=O)NC3=NC=CS3)C(=O)O | 0 | train |
| 170466177 | CC1=C(N=C(O1)C2=CC=CC=C2)CCC(=O)C3=CC=C(C=C3)CC4C(=O)NC(=O)S4 | 0 | validation |
| 170466176 | C1=CC=C(C=C1)CCNC2=NC(=CS2)C3=CC=CC=C3 | 1 | test |
| 170466175 | CC(C1=CC=CC=C1)NCCC(C2=CC=CC=C2)C3=CC=CC=C3.Cl | 1 | train |
| 170466174 | CN1C[C@H](C[C@H]2[C@H]1CC3=CN(C4=CC=CC2=C34)C)NS(=O)(=O)N(C)C | 1 | train |
| 170466173 | CN(C)[C@H]1[C@@H]2C[C@@H]3CC4=C(C(=CC=C4)O)C(=C3C(=O)[C@@]2(C(=C(C1=O)C(=O)N)O)O)O | 0 | validation |
| 170466172 | CC1=CC(=NN=C1NCCN2CCOCC2)C3=CC=CC=C3.Cl.Cl | 0 | test |
| 170466171 | CS(=O)(=O)O.CS(=O)(=O)O.C=CCNC1=NC(=NC(=N1)N2CCN(CC2)C(C3=CC=C(C=C3)F)C4=CC=C(C=C4)F)NCC=C | 0 | train |
| 170466170 | CC1=CC=C(C=C1)S(=O)(=O)NC(=O)NC2CCCC2 | 0 | train |
| 170466169 | CN1CCC(=C2C3=CC=CC=C3SC4=CC=CC=C42)CC1.C(=C¥C(=O)O)¥C(=O)O | 0 | validation |
| 170466168 | CCN(CCCCOC(=O)C1=CC(=C(C=C1)OC)OC)C(C)CC2=CC=C(C=C2)OC.Cl | 0 | test |
| 170466167 | C1=CC2=C(C(=C(C=C2Br)Br)O)N=C1 | 0 | train |
| 170466166 | C1C2=CC=CC=C2C(=O)C3=C(O1)C=CC(=C3)CC(=O)O | 0 | train |
| 170466165 | C=CCSCC1NC2=CC(=C(C=C2S(=O)(=O)N1)S(=O)(=O)N)Cl | 0 | validation |
| 170466164 | C(CC(=O)O)[C@@H](C(=O)O)NC(=O)N | 0 | test |
| 170466163 | COC1=CC(=CC(=C1OC)OC)/C=C/C(=O)N | 0 | train |
| 170466162 | CC1=C(C(C(=C(N1)C)C(=O)OCCCN2CCC(CC2)(C3=CC=CC=C3)C4=CC=CC=C4)C5=CC(=CC=C5)[N+](=O)[O-])C(=O)OC.Cl | 0 | train |
| 170466161 | COC1=C/C(=C(¥N2CCOCC2)/S)/C=CC1=O | 0 | validation |
| 170466160 | CN1[C@@H](CCC1=O)C2=CN=CC=C2 | 0 | test |
| 170466159 | CC(C1=CC=CC=C1)N2C=NC=C2C(=O)OC.Cl | 0 | train |
| 170466158 | CN1C(=O)COC2=C1C=C(C=C2C(=O)NC3CN4CCC3CC4)Cl | 0 | train |
| 170466157 | CC1=CC2=C(C=C1)N3CCNC4C3=C2CCC4.CS(=O)(=O)O | 1 | validation |
| 170466156 | CN1C2=C(C(=O)N(C1=O)C)N(C=N2)CCO | 0 | test |
| 170466155 | C1CN(CCC1CC2=CC=C(C=C2)F)CC(C3=CC=C(C=C3)Cl)O | 0 | train |
| 170466154 | C1COCCN1C(=N)N=C(N)N.Cl | 0 | train |
| 170466153 | CCOP(=S)(OCC)OC1=NC(=NC(=C1)C)C(C)C | 1 | validation |
| 170466152 | CCOC(=O)C(C1=C(C2=CC=CC=C2OC1=O)O)C3=C(C4=CC=CC=C4OC3=O)O | 0 | test |
| 170466151 | CCN(CC)CCNC(=O)C1=CC=C(C=C1)NC(=O)C.Cl | 0 | train |
| 170466150 | CCN(CC)CCOC1=CC2=C(C=C1)C3=C(C2=O)C=C(C=C3)OCCN(CC)CC | 1 | train |
| 170466149 | CCCC(C1=CC=CC=C1)(C2=CC=CC=C2)C(=O)OCCN(CC)CC.Cl | 1 | validation |
| 170466148 | CC1CN(CCN1)C2=C(C(=C3C(=C2)N(C=C(C3=O)C(=O)O)C4CC4)C)F.Cl | 0 | test |

| | | | |
|---|---|---|---|
| 170466147 | CC1=C(C(=CC=C1)C)NC2=NCCCS2 | 0 | train |
| 170466146 | CC1N2C3=CC(=C(C=C3C(=O)C(=C2S1)C(=O)O)F)N4CCNCC4 | 0 | train |
| 170466145 | CC1CCC2=C3N1C=C(C(=O)C3=CC(=C2)F)C(=O)O | 0 | validation |
| 170466144 | CC1=CC(=C(C=C1)O)N2N=C3C=CC=CC3=N2 | 1 | test |
| 170466143 | CC(CCCCN1C(=O)C2=C(N=CN2C)N(C1=O)C)O | 0 | train |
| 170466142 | CC(C)NCC(C1=CC(=CC(=C1)O)O)O.CC(C)NCC(C1=CC(=CC(=C1)O)O)O.OS(=O)(=O)O | 0 | train |
| 170466141 | CC(=O)NC1=CC=C(C=C1)S(=O)(=O)NC2=CC=C(C=C2)[N+](=O)[O-] | 0 | validation |
| 170466140 | CCCCCCCCCCCCCCCCN | 0 | test |
| 170466139 | C[C@H]1[C@H]2CC[C@@H]3[C@@]2(CC[C@H]4[C@H]3CC=C5[C@@]4(CC[C@@H](C5)N(C)C)C)CN1C | 0 | train |
| 170466138 | C1COCCN1[N+]2=NOC(=C2)N.[Cl-] | 0 | train |
| 170466137 | C1[C@@H]([C@H]([C@@H]([C@H]([C@@H]1N)O[C@@H]2[C@@H]([C@H]([C@@H]([C@H](O2)CO)O)N)O)O)O[C@@H]3[C@@H]([C@H]([C@@H]([C@H](O3)CN)O)O)N)N.OS(=O)(=O)O | 0 | validation |
| 170466136 | C[N+]1(CCCCC1)CCC(C2=CC=CC=C2)(C3=CC=CC=C3)C(=O)N.[Br-] | 0 | test |
| 170466135 | C1=CC(=CC=C1NC(=O)CCC(=O)O)S(=O)(=O)N | 0 | train |
| 170466134 | CC1=C(C=CC(=C1)O)Cl | 0 | train |
| 170466133 | CC1(OC[C@H]2[C@@H](O1)[C@H]3[C@@](O2)(OC(O3)(C)C)C(=O)[O-])C.[Na+] | 0 | validation |
| 170466132 | C1=NC2=C(C(=N1)N)N=CN2[C@H]3[C@@H]([C@@H]([C@H](O3)COP(=O)(O)O)O)O | 0 | test |
| 170466131 | CC[C@]12CC[C@H]3[C@H]([C@@H]1C=C[C@]2(C#C)O)CCC4=CC(=O)CC[C@H]34 | 0 | train |
| 170466130 | C1=CC(=C[N+](=C1)[O-])C(=O)O | 0 | train |
| 170466129 | C[C@@H]1CC(=O)NN=C1C2=CC=C(C=C2)NN=C(C#N)C#N | 1 | validation |
| 170466128 | C[C@@H]1CC(=O)NN=C1C2=CC=C(C=C2)NN=C(C#N)C#N | 0 | test |
| 170466127 | CC(C)[N+](C)(C)CC(COC1=CC=CC2=CC=CC=C21)O.[Cl-] | 0 | train |
| 170466126 | CCCCCCCCCCCC[N+](C)(C)CC1=CC=CC=C1.[Br-] | 0 | train |
| 170466125 | C(C(Cl)(Cl)Cl)(NC(=O)NC(C(Cl)(Cl)Cl)O)O | 0 | validation |
| 170466124 | C1=CC2=C(C=CC(=C2N=C1)O)[N+](=O)[O-] | 1 | test |
| 170466123 | C1COCCN1C2=CC3=C(C=C2C(F)(F)F)NC(=O)C(=O)N3CP(=O)(O)O | 0 | train |
| 170466122 | C1=CC2=C(C=C1Cl)N=C(O2)N | 0 | train |
| 170466121 | C1COCC(=O)N1C2=CC=C(C=C2)N3CC(OC3=O)CNC(=O)C4=CC=C(S4)Cl | 0 | validation |
| 170466120 | C1CN(CCN1CCCN2C3=CC=CC4=C3C(=CC=C4)S2(=O)=O)C5=CC=C(C=C5)F | 1 | test |
| 170466119 | C1CN(CC=C1C2=CC=CC=C2)CCCCC3=CNC4=C3C=C(C=C4)O | 0 | train |
| 170466118 | CC1=CC(=C(C(=C1)C)N=C2C=C3C4=CC(=C(C=C4CCN3C(=O)N2C)OC)OC)C.Cl | 1 | train |
| 170466117 | CC1=C(C2=CC=CC=C2N1CCN3CCOCC3)C(=O)C4=CC=C(C=C4)OC | 0 | validation |
| 170466116 | CCN1C2=C(C(=O)N(C1=O)CC)N(C(=N2)/C=C/C3=CC(=C(C=C3)OC)OC)C | 0 | test |
| 170466115 | CCNC(=O)/C=C/C1=CC(=CC=C1)Br | 1 | train |
| 170466114 | CN1[C@@H]2CC[C@H]1CC(C2)OC(=O)C3=CC(=CC(=C3)Cl)Cl | 0 | train |
| 170466113 | C1=CC=C2C(=C1)C(=NN=C2NC3=CC=C(C=C3)Cl)CC4=CC=NC=C4 | 1 | validation |
| 170466112 | C1=CC=C(C(=C1)C2=NC(=NO2)C3=CC=NC=C3)Cl | 1 | test |
| 170466111 | CCCCNC1=C2C=NN(C2=NC(=C1C(=O)OCC)C)CC.Cl | 1 | train |
| 170466110 | CC(C)[C@H]1C2=C(CC[C@@]1(CCN(C)CCCC3=NC4=CC=CC=C4N3)OC(=O)COC)C=C(C=C2)F.Cl.Cl | 1 | train |
| 170466109 | COC(=O)[C@H](CC1=CC=CC=C1)NC(=O)[C@H](CC(=O)O)N | 0 | validation |
| 170466108 | CC1=C(C(=O)N2CCSC2=N1)CCN3CCC(CC3)C(=O)C4=CC=C(C=C4)F | 0 | test |
| 170466107 | CCCCCCOC(=O)/N=C(¥C1=CC=C(C=C1)NCC2=NC3=C(N2C)C=CC(=C3)C(=O)N(CCC(=O)OCC)C4=CC=CC=N4)/N | 0 | train |
| 170466106 | CC1=CC2=C(N1)C=CC=C2OCC(CNC(C)(C)C)OC(=O)C3=CC=CC=C3 | 0 | train |
| 170466105 | CC1=C(C(=CC=C1)C)NC(=O)CN2CCCCCC2 | 0 | validation |
| 170466104 | CC(C)CCOC(=O)C1=CC=C(C=C1)N(C)C | 1 | test |
| 170466103 | CC(C)(C)C1=NN=C(S1)NS(=O)(=O)C2=CC=C(C=C2)N | 0 | train |
| 170466102 | CCCCCCCCCCCCCCCC(=O)NCCO | 0 | train |
| 170466101 | CC1=C(C=C(C=C1)C(=O)NC2=C3C(=CC(=CC3=C(C=C2)S(=O)(=O)O)S(=O)(=O)O)S(=O)(=O)O)NC(=O)C4=CC(=CC=C4)NC(=O)NC5=CC=CC(=C5)C(=O)NC6=C(C=CC(=C6)C(=O)NC7=C8C(=CC(=CC8=C(C=C7)S(=O)(=O)O)S(=O)(=O)O)S(=O)(=O)O)C | 0 | validation |
| 170466100 | CC(C(=O)N1C(CN(C1=O)C)C(=O)O)NC(CCC2=CC=CC=C2)C(=O)O | 0 | test |
| 170466099 | C[N+]1(CC2=C(C1)C(=C(C(=C2Cl)Cl)Cl)Cl)CC[N+](C)(C)C.[Cl-].[Cl-] | 0 | train |
| 170466098 | CCCCCCCCOC1=CC=CC=C1C(=O)NC2=CC=C(C=C2)C(=O)OCC[N+](C)(CC)CC.[Br-] | 0 | train |
| 170466097 | C1=CC=C(C=C1)CNC(=O)CCNNC(=O)C2=CC=NC=C2 | 0 | validation |
| 170466096 | C1CC[C@H]2[C@@H](C1)C(=O)N(C2=O)CCCCN3CCN(CC3)C4=NSC5=CC=CC=C54.Cl | 0 | test |
| 170466095 | C[C@@H]1CC2=CC(=O)CC[C@@H]2[C@@H]3[C@@H]1[C@@H]4CC[C@]([C@]4(CC3)C)(C)O | 0 | train |

| | | | |
|---|---|---|---|
| 170466094 | CC(=O)O[C@H]1[C@H]([C@@H]2[C@]([C@H](CCC2(C)C)O)([C@@]3([C@@]1(O[C@@](CC3=O)(C)C=C)C)O)C)O | 0 | train |
| 170466093 | C1CN(CCN1)C2=NC3=CC=CC=C3C=C2.C(=C¥C(=O)O)¥C(=O)O | 0 | validation |
| 170466092 | C1=CC=C(C(=C1)C2=CC=C(C=C2)C(F)(F)F)C(=O)O | 0 | test |
| 170466091 | C1=CC=C(C(=C1)C(=O)O)OP(=O)(O)O | 0 | train |
| 170466090 | C1=CC=C2C(=C1)C(=O)NC(=O)O2 | 0 | train |
| 170466089 | C1=C(OC(=C1)[N+](=O)[O-])C=NO | 1 | validation |
| 170466088 | C1CN(CCN1CCCC2=CC=CC=C2)CCOC(C3=CC=C(C=C3)F)C4=CC=C(C=C4)F.Cl.Cl | 1 | test |
| 170466087 | COC(=O)NC1=NC2=C(N1)C=C(C=C2)C(=O)C3=CC=CS3 | 1 | train |
| 170466086 | CN1C(=O)CCC(=N1)C(=O)N | 0 | train |
| 170466085 | CCN(CC)CCCC(C)NC1=C2C=C(C=CC2=NC3=C1C=CC(=C3)Cl)OC.O.O.Cl.Cl | 1 | validation |
| 170466084 | C1=CC=C(C=C1)C2=C(N=C(S2)N)N | 0 | test |
| 170466083 | C(CCl)N(CCCl)CCCl | 0 | train |
| 170466082 | CCN(CC)CCOC(=O)C(C1CCCCC1)C2=CC=CC=C2.Cl | 0 | train |
| 170466081 | CCN(CC)CCOC(=O)C(C)(C1=CC=CC=C1)C2=CC=CC=C2 | 1 | validation |
| 170466080 | CCN(CC)C(=O)C1=CC(=CC=C1)C | 0 | test |
| 170466079 | CCN(CCN1C(=NC2=C1C(=O)N(C(=O)N2C)C)CC3=CC=CC=C3)CCO.Cl | 0 | train |
| 170466078 | CCC(C)(C)C1=CC=C(C=C1)CC(C)CN2C[C@H](O[C@H](C2)C)C | 0 | train |
| 170466077 | CC1=C(C(=CC=C1)C)NC(=O)C2=CC=CC=C2C(=O)O | 0 | validation |
| 170466076 | CC(C)C1=C(N(N(C1=O)C2=CC=CC=C2)C)CN(C)C(C)CC3=CC=CC=C3 | 1 | test |
| 170466075 | CC(=O)OC[C@@H]1[C@H]([C@H]([C@@H](O1)N2C(=O)NC(=O)C=N2)OC(=O)C)OC(=O)C | 0 | train |
| 170466074 | CCN1/C(=C¥C=C¥C2=[N+](C3=CC=CC=C3C=C2)CC)/C=CC4=CC=CC=C41.[I-] | 1 | train |
| 170466073 | CC1=C(N(C(=C1)CC(=O)[O-])C)C(=O)C2=CC=C(C=C2)Cl.[Na+] | 0 | validation |
| 170466072 | CC1=C(OC(=N1)C2=CC=C(C=C2)Cl)COC(C)(C)C(=O)O | 0 | test |
| 170466071 | C1=CC=C2C(=C1)C(=O)C3=CC=CC=C3N2CC(=O)O | 0 | train |
| 170466070 | CNC1=C2CCCCC2=NN1C | 0 | train |
| 170466069 | C[C@]12CC[C@H]3[C@H]([C@@H]1CC[C@]2(C)O)CCC4=CC(=O)C=C[C@]34C | 0 | validation |
| 170466068 | CCO[C@@H]1[C@@H]([C@@H]2CC[C@H]([C@H]3[C@]24[C@H](O1)OC(CC3)(OO4)C)C)C | 1 | test |
| 170466067 | CCCCCCCCCCCCCCCC[N+](C)(C)CC.[Br-] | 0 | train |
| 170466066 | CCCC(=O)OCOC(=O)C1=C(NC(=C(C1C2=C(C(=CC=C2)Cl)Cl)C(=O)OC)C)C | 0 | train |
| 170466065 | CC1([C@@H](N2[C@H](S1)[C@@H](C2=O)NC(=O)[C@@H](C3=CC=CC=C3)N)C(=O)OCOC(=O)C(C)(C)C)C | 0 | validation |
| 170466064 | C[N+](C)(C)CCOC(=O)C(C1=CC=CC=C1)(C2=CC=CC=C2)O.[I-] | 0 | test |
| 170466063 | C[C@H]1C[C@@H]2[C@H](CC[C@]3([C@H]2CC[C@@]3(C(=O)C)O)C)[C@@]4(C1=CC(=O)CC4)C | 1 | train |
| 170466062 | CC[N+]1=C2C=C(C=CC2=C3C=CC(=CC3=C1C4=CC=CC=C4)N)N.[Br-] | 0 | train |
| 170466061 | CC1=CC=C(C=C1)C(C)OC(=O)C2(CCC(C2(C)C)C(=O)O)C.C(CO)NCCO | 0 | validation |
| 170466060 | C1=CC(=CC(=C1)NC(=O)C(=O)O)C2=NNN=N2 | 0 | test |
| 170466059 | C1CN(CCN1CCCN2C3=CC=CC=C3NC2=O)C(C4=CC=CC=C4)C5=CC=CC=C5 | 0 | train |
| 170466058 | C1=CC(=C(C=C1S(=O)(=O)N)S(=O)(=O)N)Cl | 0 | train |
| 170466057 | CN(C1=NC(=CC=C1)OC)C(=S)OC2=CC3=C(CCCC3)C=C2 | 1 | validation |
| 170466056 | CN1CCN(CC1)C2=C(C=C3C(=C2F)N(C=C(C3=O)C(=O)O)CCF)F | 0 | test |
| 170466055 | CC1(CCC2CC(CC1N2C)OC(=O)C(C3=CC=CS3)(C4=CC=CS4)O)C | 0 | train |
| 170466054 | C1=CC(=CC=C1CN2C=CN=C2)/C=C/C(=O)O.Cl | 0 | train |
| 170466053 | CC1=CC=C(C=C1)S(=O)(=O)O.C1CN(CC1N)C2=C(C=C3C(=O)C(=CN(C3=N2)C4=C(C=C(C=C4)F)F)C(=O)O)F | 0 | validation |
| 170466052 | CC1=NN=C2N1C3=C(C=C(S3)CCC(=O)N4CCOCC4)C(=NC2)C5=CC=CC=C5Cl | 0 | test |
| 170466051 | CC(CN(C)C)C(C)(CC1=CC=C(C=C1)Cl)O.Cl | 0 | train |
| 170466050 | CC1=C(C(=O)N(N1C)C2=CC=CC=C2)NC(=O)C3=CN=CC=C3 | 0 | train |
| 170466049 | CC(C)C1=NN2C=CC=CC2=C1C(=O)C(C)C | 1 | validation |
| 170466048 | C1=C(C(=CNC1=O)Cl)O | 0 | test |
| 170466047 | C1CC2C[C@H]1[C@H]3[C@@H]2CN(C3)NC(=O)C4=CC(=C(C=C4)Cl)S(=O)(=O)N | 0 | train |
| 170466046 | C1CCC(C1)CC2NC3=CC(=C(C=C3S(=O)(=O)N2)S(=O)(=O)N)Cl | 0 | train |
| 170466045 | CC(=CCC/C(=C/CC/C(=C/C(=O)OCC(=O)[C@]1(CC[C@@H]2[C@@]1(C[C@@H]([C@H]3[C@H]2CCC4=CC(=O)C=C[C@]34C)O)C)O)/C)/C)C | 0 | validation |
| 170466044 | CC(C)C(=O)OCC1=CC(=CC=C1)OC(=O)[C@H]2C(S[C@H]3N2C(=O)[C@H]3NC(=O)CC4=CC=CC=C4)(C)C | 0 | test |
| 170466043 | CC1=C(C2=C(N1C(=O)C3=CC=C(C=C3)Cl)C=CC(=C2)OC)CC(=O)OCC(=O)O | 0 | train |
| 170466042 | CCN1C=C(C(=O)C2=CN=C(N=C21)N3CCCC3)C(=O)O | 0 | train |
| 170466041 | C1=C(C(=O)NC(=O)N1[C@@H]2[C@H]([C@@H]([C@H](O2)CO)O)O)/C=C¥Br | 0 | validation |
| 170466040 | C1=CC=C(C=C1)C(=O)NCCC(=O)O | 0 | test |
| 170466039 | C1CCC(CC1)N2CC3=CC(=C(C=C3C2=O)S(=O)(=O)N)Cl | 0 | train |

| | | | |
|---|---|---|---|
| 170466038 | C1=CC(=NC=C1Cl)C(=O)NCCN.Cl | 0 | train |
| 170466037 | C1CN(CCN1C/C=C/C2=CC=CC=C2)C(C3=CC=C(C=C3)F)C4=CC=C(C=C4)F | 0 | validation |
| 170466036 | CCCOC(C1=CC=CC=C1)(C2=CC=CC=C2)C(=O)OC3CCN(CC3)C.Cl | 0 | test |
| 170466035 | CCCCCOC1=CC=CC=C1/C(=C¥SC)/N2C=CN=C2 | 1 | train |
| 170466034 | CCCCC(CC)CNC(=O)CC(C)OC(=O)CCC(=O)O | 0 | train |
| 170466033 | CC1=NC(=C(O1)C2=CC(=C(C=C2)S(=O)(=O)N)F)C3CCCCC3 | 1 | validation |
| 170466032 | CC(C)NCC(C1=CC=CC=C1Cl)O.Cl | 0 | test |
| 170466031 | CC[C@H](C1=CC=C(C=C1)O)[C@@H](CC)C2=CC=C(C=C2)O | 1 | train |
| 170466030 | C1=CC=C2C(=C1)C=CC=C2CC(=O)O | 0 | train |
| 170466029 | CC1=NC=C(C(=N1)N)CNC(=O)N(CCCl)N=O | 0 | validation |
| 170466028 | CC1=NC=C(C(=C1O)C=O)COP(=O)(O)O.O | 0 | test |
| 170466027 | C1=NC(=C(N1[C@H]2[C@@H]([C@@H]([C@H](O2)CO)O)O)O)C(=O)N | 0 | train |
| 170466026 | COC1=CC=CC=C1N2CCN(CC2)CC(COC3=CC=CC4=CC=CC=C43)O | 0 | train |
| 170466025 | CCO[C@H](CC1=CC=C(C=C1)OCCC2=CC=C(C=C2)OS(=O)(=O)C)C(=O)O | 0 | validation |
| 170466024 | CCOC(=O)[C@H](CCC1=CC=CC=C1)N[C@@H](C)C(=O)N2[C@@H](CN(C2=O)C)C(=O)O.Cl | 0 | test |
| 170466023 | CCCC1=CC(=C(C=C1)OCC(=O)N2CCN(CC2)C(=O)COC3=C(C=C(C=C3)CCC)OC)OC | 1 | train |
| 170466022 | CCN(CC)CC1=NC=CN1C2=C(C=C(C=C2)[N+](=O)[O-])C(=O)C3=CC=CC=C3Cl | 0 | train |
| 170466021 | CCN(CC(C)O)C1=NN=C(C=C1)NNC(=O)OCC | 0 | validation |
| 170466020 | CCOC(=O)OC(C)OC1=C(N(S(=O)(=O)C2=CC=CC=C21)C)C(=O)NC3=CC=CC=N3 | 0 | test |
| 170466019 | CC1=NC=C(C(=N1)N)CN(/C(=C(¥SS/C(=C(¥N(C=O)CC2=CN=C(N=C2N)C)/C)/CCOC(=O)C(C)C)/CCOC(=O)C(C)C)/C)C=O | 0 | train |
| 170466018 | CC(C)(C(=O)OCCCOC(=O)C(C)(C)OC1=CC=C(C=C1)Cl)OC2=CC=C(C=C2)Cl | 0 | train |
| 170466017 | CC(=CCOC1=CC=C(C=C1)/C=C/C(=O)C2=C(C=C(C=C2)OCC=C(C)C)OCC(=O)O)C | 0 | validation |
| 170466016 | C1=CC=C(C(=C1)C=O)C=O | 0 | test |
| 170466015 | C=CCN1CCC2=C(CC1)SC(=N2)N | 0 | train |
| 170466014 | CC1=C(C2=C(N1C(=O)C3=CC=C(C=C3)Cl)C=CC(=C2)OC)CC(=O)OC/C=C(¥C)/CC/C=C(¥C)/CCC=C(C)C | 0 | train |
| 170466013 | CN1C(=CC(=O)N(C1=O)C)NCCCN2CCN(CC2)C3=CC=CC=C3OC.Cl | 0 | validation |
| 170466012 | C1=CC(=C(C(=C1)Cl)COC(CN2C=CN=C2)C3=C(C=C(C=C3)Cl)Cl)Cl | 1 | test |
| 170466011 | C1CNCCN(C1)S(=O)(=O)C2=CC=CC3=C2C=CN=C3.Cl | 0 | train |
| 170466010 | COC1=C(C(=C(C=C1)CN2CCN(CC2)C(C3=CC=C(C=C3)F)C4=CC=C(C=C4)F)OC)OC.Cl.Cl | 0 | train |
| 170466009 | CCCNC(C)(C)COC(=O)C1=CC=CC=C1.Cl | 0 | validation |
| 170466008 | CCOC1=CC(=C(C=C1C(=O)NCC2CN(CCO2)CC3=CC=C(C=C3)F)Cl)N.C(C(=O)O)C(CC(=O)O)(C(=O)O)O | 1 | test |
| 170466007 | CCCCCCCCC/C=C¥C=C/C=C¥C=C/C=C¥C(=O)OCC | 0 | train |
| 170466006 | CCCCC/C=C¥C/C=C¥CCCCCCCC(=O)OCC | 0 | train |
| 170466005 | CC1=CC(=NO1)NS(=O)(=O)C2=CC=C(C=C2)NC(=O)C | 0 | validation |
| 170466004 | C[C@@H]1CCC[C@@H](N1CCCC(C2=CC=CC=C2)(C3=CC=CC=N3)O)C | 0 | test |
| 170466003 | C1=CC=C(C=C1)C2=NC(=C(S2)CC(=O)O)C3=CC=C(C=C3)Cl | 0 | train |
| 170466002 | CC(C)NC(C)C | 0 | train |
| 170466001 | CN1C2=CC=CC=C2C(=O)N(C3=CC=CC=C31)CCN(C)C.Cl | 1 | validation |
| 170466000 | CCN(CC)CCOC1=CC=CC=C1C(=O)CCC2=CC=CC=C2 | 1 | test |
| 170465999 | CCO[C@H]1[C@@H]([C@H]([C@H](O1)C(COCC2=CC=CC=C2)OCC3=CC=CC=C3)OCC4=CC=CC=C4)O | 0 | train |
| 170465998 | CC(=O)O[C@@H]1CC2C3CCC4=C(C3CCC2([C@H]1OC(=O)C)C)C=CC(=C4)OC(=O)C5=CC=CC=C5 | 0 | train |
| 170465997 | CC(C)OC1=CC2=C(C=C1)C(=O)C(=CO2)C3=CC=CC=C3 | 1 | validation |
| 170465996 | CC(C)(C)C(=O)OC1=CC=C(C=C1)S(=O)(=O)NC2=CC=CC=C2C(=O)NCC(=O)[O-].O.O.O.O.[Na+] | 0 | test |
| 170465995 | CC(C)(C)C1=CC=C(C=C1)C(=O)CCCN2CCC(CC2)OC(C3=CC=CC=C3)C4=CC=CC=C4 | 1 | train |
| 170465994 | CN1CCC2=CC3=C(C4=C2[C@@H]1CC5=CC=C(C=C5)OC6=C(C=CC(=C6)C[C@@H]7C8=CC(=C(C=C8CCN7C)OC)O4)OC)OCO3 | 1 | train |
| 170465993 | C1[C@@H]([C@@H]([C@H]([C@@H]([C@]1(CO)O)O)O)O)NC(CO)CO | 0 | validation |
| 170465992 | C1=C(C=C(C(=C1Cl)N2C(=C(C(=N2)C#N)S(=O)C(F)(F)F)N)Cl)C(F)(F)F | 0 | test |
| 170465991 | CC(C)C(CCCN(C)CCC1=CC(=C(C=C1)OC)OC)(C#N)C2=CC(=C(C(=C2)OC)OC)OC.Cl | 0 | train |
| 170465990 | CCCCCCOC1=CC=CC=C1C(=O)N | 1 | train |
| 170465989 | CCCCOCCOC(=O)C1=CN=CC=C1 | 0 | validation |
| 170465988 | CC1=C(C(=CC=C1)C)NC(=O)CC23CCCN2CCC3 | 0 | test |
| 170465987 | CC1=NC=C(C(=N1)N)CN(C=O)C(=C(CCOP(=O)(O)O)SC(=O)C2=CC=CC=C2)C | 0 | train |
| 170465986 | CC1=CC(=NC(=N1)C)NS(=O)(=O)C2=CC=C(C=C2)N | 1 | train |
| 170465985 | C[C@H]1COC2=C3N1C=C(C(=O)C3=CC(=C2C4(CC4)N)F)C(=O)O | 0 | validation |

| | | | |
|---|---|---|---|
| 170465984 | C[C@]12CC[C@H]3[C@H]([C@@H]1CC[C@@]2(C#C)O)CCC4=CCCC[C@H]34 | 0 | test |
| 170465983 | C(=C¥C(=O)O)¥C(=O)O | 0 | train |
| 170465982 | CCCCN=C(N)N=C(N)N | 0 | train |
| 170465981 | COC(=O)C1=C(C=CC(=C1)Cl)NS(=O)(=O)C(F)(F)F | 0 | validation |
| 170465980 | C1CC2=C(C[C@@H]1NS(=O)(=O)C3=CC=C(C=C3)F)C4=CC=CC=C4N2CCC(=O)O | 0 | test |
| 170465979 | C1=CC=C(C(=C1)CC(=O)OCC(=O)O)NC2=C(C=CC=C2Cl)Cl | 0 | train |
| 170465978 | C1CCN(CC1)CC2=CC(=NC=C2)OC/C=C¥CNC(=O)CS(=O)CC3=CC=CO3 | 0 | train |
| 170465977 | CC1=C(C(C(=C(N1)C)C(=O)OCC(=O)C)C2=CC=CC=C2[N+](=O)[O-])C(=O)OC | 0 | validation |
| 170465976 | CC1=NC=C(C(=N1)N)CN(C=O)/C(=C(¥CCO)/SSCCC(CCCCC(=O)OC)SC(=O)C)/C | 0 | test |
| 170465975 | CCOC(=O)C1=CC(=O)C2=C(O1)C=CC=C2OCC(COC3=CC=CC4=C3C(=O)C=C(O4)C(=O)OCC)OC(=O)[C@H](CCCCN)N | 0 | train |
| 170465974 | CN(C)CCOC1=CC=C(C=C1)CNC(=O)C2=CC(=C(C=C2)OC)OC.Cl | 0 | train |
| 170465973 | CN(C)CC(COC1=CC=CC=C1CCC2=CC(=CC=C2)OC)OC(=O)CCC(=O)O.Cl | 0 | validation |
| 170465972 | CCCCC1(C(=O)N(N(C1=O)C2=CC=CC=C2)C3=CC=CC=C3)COC(=O)CCC(=O)O | 0 | test |
| 170465971 | CCCCCN(CCCOC)C(=O)C(CCC(=O)O)NC(=O)C1=CC(=C(C=C1)Cl)Cl | 0 | train |
| 170465970 | CC(=O)OC1=CC=C(C=C1)C(=C2CCCCC2)C3=CC=C(C=C3)OC(=O)C | 1 | train |
| 170465969 | CC(=CCC1C(=O)N(N(C1=O)C2=CC=CC=C2)C3=CC=CC=C3)C | 0 | validation |
| 170465968 | CC(COC1=CC=CC=C1)NC(C)C(C2=CC=C(C=C2)O)O.Cl | 0 | test |
| 170465967 | CC[N+]1(CCCCC1)CCOC(=O)C(C2=CC=CC=C2)(C3=CC=CC=C3)O.[Br-] | 0 | train |
| 170465966 | CCN(CC)C(=O)N[C@H]1CN([C@H]2CC3=CNC4=CC=CC(=C34)C2=C1)C | 0 | train |
| 170465965 | CNC(=O)C1=C(C(=C(C(=C1I)NC(=O)CCCCC(=O)NC2=C(C(=C(C(=C2I)C(=O)O)I)C(=O)NC)I)I)C(=O)O)I | 0 | validation |
| 170465964 | C1CN(CCN1CC/C=C¥2/C3=CC=CC=C3SC4=C2C=C(C=C4)C(F)(F)F)CCO | 1 | test |
| 170465963 | COC1=CC(=CC(=C1)OC)OC | 0 | train |
| 170465962 | COC1=C(C=C(C=C1)C2CC(=O)NC2)OC3CCCC3 | 0 | train |
| 170465961 | CC1=C(C(C(=C(N1)C)C(=O)OCCN2CCN(CC2)C(C3=CC=CC=C3)C4=CC=CC=C4)C5=CC(=CC=C5)[N+](=O)[O-])C(=O)OC.Cl.Cl | 0 | validation |
| 170465960 | CN1C(=CC(=O)N(C1=O)C)NCCN(CCCC2=CC=C(C=C2)[N+](=O)[O-])CCO.Cl | 0 | test |
| 170465959 | C1=CC(=CC=C1C(CN)O)O | 0 | train |
| 170465958 | CCN(CC)C(=O)NC1=CC(=C(C=C1)OCC(CNC(C)(C)C)O)C(=O)C.Cl | 0 | train |
| 170465957 | CCCCC(/C=C(¥C)/C=C/C=C/C(=O)N1CCCC1=O)O | 0 | validation |
| 170465956 | C[C@H]1[C@@H]([C@H]([C@H]([C@@H](O1)O[C@H]2CC[C@@]3([C@H]4CC[C@@]5([C@H](CC[C@@]5([C@@H]4CCC3=C2)O)C6=COC(=O)C=C6)C)C)O)O)O | 1 | test |
| 170465955 | CC(C)(C)NCC(C1=CC(=C(C(=C1)Cl)N)Cl)O | 0 | train |
| 170465954 | CC(=O)NC1=CC=C(C=C1)CC(=O)O | 0 | train |
| 170465953 | CC(CN1C=NC2=C1C(=O)N(C(=O)N2C)C)O | 0 | validation |
| 170465952 | C1=CC=C(C=C1)CN2C3=CC=CC=C3C(=N2)OCC(=O)[O-].[Na+] | 0 | test |
| 170465951 | C[C@]12CC[C@](C[C@H]1C3=CC(=O)[C@@H]4[C@]5(CC[C@@H](C([C@@H]5CC[C@]4([C@@]3(CC2)C)C)(C)C)OC(=O)CCC(=O)[O-])C)(C)C(=O)[O-].[Na+].[Na+] | 0 | train |
| 170465950 | C1=NC2=C(N1)C(=NC=N2)N | 0 | train |
| 170465949 | C1CCN(CC1)C2(CCN(CC2)CCCC(=O)C3=CC=C(C=C3)F)C(=O)N.Cl.Cl | 0 | validation |
| 170465948 | C1=CC=C(C=C1)N(CCC(=O)O)C(=O)C2=C(C(=C(C=C2I)I)N)I | 0 | test |
| 170465947 | C1=CC(=C(C=C1Cl)C2=NC(=NC(=N2)N)N)Cl.C(=C¥C(=O)O)¥C(=O)O | 0 | train |
| 170465946 | C1=CC=C(C=C1)C2=C(N=CN2)C3=CC=CC=C3 | 0 | train |
| 170465945 | CCN(CC)CC(=O)NC1=C(C=CC=C1C)C(=O)OC | 1 | validation |
| 170465944 | CCOC(=O)[C@H](CCC1=CC=CC=C1)N[C@H]2CS[C@@H](CN(C2=O)CC(=O)O)C3=CC=CS3.Cl | 0 | test |
| 170465943 | C1=CC=C(C(=C1)C(=O)OCC(CO)O)NC2=C3C=CC(=CC3=NC=C2)Cl.Cl | 0 | train |
| 170465942 | C1CN(CCN1CCCN2C3=CC=CC=C3C=CC4=CC=CC=C42)CCO.Cl.Cl | 0 | train |
| 170465941 | CC(=O)OCC(=O)NCCCOC1=CC=CC(=C1)CN2CCCCC2.Cl | 0 | validation |
| 170465940 | CC1=C(C(=O)C(=C(C1=O)C)C(CCCCCC(=O)O)C2=CC=CC=C2)C | 0 | test |
| 170465939 | COCCOC1=CC(=CC=C1)S(=O)(=O)[N-]C2=NC=CC=N2.[Na+] | 0 | train |
| 170465938 | CN1CCN(CC1)C2=NC3=CC=CC=C3SC4=C2C=C(C=C4)Cl | 1 | train |
| 170465937 | C[C@@H]1C2=C(CN1)C=C(C=C2)C3=C(C4=C(C=C3)C(=O)C(=CN4C5CC5)C(=O)O)OC(F)F.CS(=O)(=O)O.O | 0 | validation |
| 170465936 | CCN(CC)CCNC(C1=CC=CC=C1)C(=O)OCCC(C)C | 0 | test |
| 170465935 | CC1=NN(C(=O)C1)C2=CC=CC=C2 | 0 | train |
| 170465934 | CN(C)CCOC1=CC2=CC=CC=C2SC3=C1C=C(C=C3)Cl | 0 | train |
| 170465933 | C1=CSC(=C1)CNC2=CC(=C(C=C2C3=NNN=N3)S(=O)(=O)N)Cl | 0 | validation |
| 170465932 | C1[C@H]([C@H](OC2=CC(=CC(=C21)O)O)C3=CC(=C(C=C3)O)O)O | 0 | test |
| 170465931 | CC1=CN=C(C(=C1OC)C)CS(=O)C2=NC3=C(N2)C=CC(=N3)OC | 1 | train |
| 170465930 | COC1=NC=CN=C1NS(=O)(=O)C2=CC=C(C=C2)N | 0 | train |

| | | | |
|---|---|---|---|
| 170465929 | C[C@@H]([C@@H](C1=CC=CC=C1)O)NCCC(=O)C2=CC(=CC=C2)OC.Cl | 0 | validation |
| 170465928 | CN1C=C(C2=CC=CC=C21)C(=O)[C@@H]3CCC4=C(C3)NC=N4.Cl | 0 | test |
| 170465927 | CCN1CCCC(C1)OC(=O)C(C2=CC=CC=C2)C3=CC=CC=C3.Cl | 0 | train |
| 170465926 | CN(CCCN1C2=CC=CC=C2CCC3=CC=CC=C31)CC(=O)C4=CC=C(C=C4)Cl.Cl | 1 | train |
| 170465925 | CCC1NC2=CC(=C(C=C2S(=O)(=O)N1)S(=O)(=O)N)Cl | 0 | validation |
| 170465924 | C1CCN(C1)CC2=NC3=CC=CC=C3N2CC4=CC=C(C=C4)Cl.Cl | 1 | test |
| 170465923 | CC1=C(C2=CC3=C(C(=C(N3)C=C4C(=C(C(=N4)C=C5C(=C(C(=N5)C=C1N2)C)CCC(=O)[O-])CCC(=O)[O-])C)C=C)C)C=C.[Na+].[Na+] | 0 | train |
| 170465922 | CC(C)CC1=CC=C(C=C1)CC(=O)O | 0 | train |
| 170465921 | CC(C)(C)NCC(C1=CC(=C(C(=C1)Cl)N)C(F)(F)F)O.Cl | 0 | validation |
| 170465920 | C1CN(CCN1C/C=C/C2=CC=CC=C2)C(C3=CC=CC=C3)C4=CC=CC=C4 | 0 | test |
| 170465919 | C[C@@]1(C2C[C@H]3[C@@H](C(=O)C(=C([C@]3(C(=O)C2=C(C4=C1C=CC=C4O)O)O)O)C(=O)NCN5CCCC5)N(C)C)O | 0 | train |
| 170465918 | C[C@]12CC[C@H]3[C@H]([C@@H]1CC[C@@H]2OC(=O)CCC4=CC=CO4)CCC5=CC(=O)CC[C@H]35 | 0 | train |
| 170465917 | C([C@@H]1[C@@H]([C@@H]([C@H]([C@@H](O1)O[C@H]([C@@H](CO)O)[C@@H]([C@H](CO)O)O)O)O)O)O | 0 | validation |
| 170465916 | C1=CC(=CC=C1O)S(=O)(=O)O | 0 | test |
| 170465915 | C1=CNC(=O)NC1=O | 0 | train |
| 170465914 | CC#C[C@@]1(CC[C@@H]2[C@@]1(CC[C@H]3[C@H]2C[C@@H](C4=CC(=O)CC[C@]34C)C)C)O | 0 | train |
| 170465913 | C1=CC=C(C=C1)C2=C(C(=O)N=C(N2)N)Br | 0 | validation |
| 170465912 | CN1C(=O)CN=C(C2=C1C=CC(=C2)[N+](=O)[O-])C3=CC=CC=C3 | 0 | test |
| 170465911 | CNC1CCCN(C1)C2=C(C=C3C(=C2OC)N(C=C(C3=O)C(=O)O)C4CC4)F | 0 | train |
| 170465910 | CC1(OC[C@H](O1)COC(=O)CCC2=CC=C(C=C2)OC[C@H](CNCCNC(=O)N3CCOCC3)O)C.Cl | 0 | train |
| 170465909 | CCC(=O)O[C@H]1CCC2C3CCC4([C@H](CCC4C3CCC2=C1)OC(=O)CC)C | 0 | validation |
| 170465908 | CC(=CCC/C(=C/CC/C(=C/CCC(=O)OC/C=C(¥C)/CCC=C(C)C)/C)/C)C | 0 | test |
| 170465907 | C[C@]12CCC(=O)C=C1CC[C@@H]3[C@@H]2CC[C@]4([C@H]3CCC4=O)C | 0 | train |
| 170465906 | COC1=C2CCCCC(C2=NC=C1)S(=O)C3=NC4=CC=CC=C4N3 | 1 | train |
| 170465905 | C[C@]12CC[C@H]3[C@H]([C@@H]1CC[C@@H]2OC(=O)CCC4CCCC4)CCC5=CC(=O)CC[C@H]35 | 0 | validation |
| 170465904 | C[N+]1([C@@H]2CC[C@H]1CC(C2)OC(=O)C(C3=CC=CC=C3)(C4=CC=CC=C4)O)CCF.[Br-].[Br-] | 0 | test |
| 170465903 | C1=NC(=S)C2=C(N1)N(C=N2)[C@H]3[C@@H]([C@@H]([C@H](O3)CO)O)O | 1 | train |
| 170465902 | C1=CC=C(C=C1)CCCOC(=O)N | 0 | train |
| 170465901 | C1CC(=O)N(C1)CC(=O)N | 0 | validation |
| 170465900 | CNCCCCOC1=CC=CC=C1CC2=CC=CC=C2 | 1 | test |
| 170465899 | CCC(=C(C1=CC=CC=C1)C2=CC=CC=C2)CN.Cl | 0 | train |
| 170465898 | CC1=C(C=C(C(=O)N1)C#N)C2=CN3C=CN=C3C=C2.Cl | 0 | train |
| 170465897 | CCOC(=O)C1=C(NC(=C(C1C2=CC(=CC=C2)[N+](=O)[O-])C(=O)OC)C)C | 0 | validation |
| 170465896 | CCC(=O)C1=CC=C(C=C1)O | 0 | test |
| 170465895 | CC(C)CC1=CC=C(C=C1)C(C)C(=O)OCC2=CC=CC=N2 | 0 | train |
| 170465894 | C[N+](CCCl)(CCCl)[O-] | 0 | train |
| 170465893 | C[N+]1([C@@H]2CC[C@H]1CC(C2)OC(=O)C(CO)C3=CC=CC=C3)C.[N+](=O)([O-])[O-] | 0 | validation |
| 170465892 | C1C(CC2=CC=CC=C2C1C3=C(SC4=CC=CC=C4C3=O)O)C5=CC=C(C=C5)C6=CC=C(C=C6)Br | 1 | test |
| 170465891 | C1=CC=C2C(=C1)C(=CC(=O)N2)CC(C(=O)O)NC(=O)C3=CC=C(C=C3)Cl | 0 | train |
| 170465890 | C1=CC(=C(C=C1[C@@H]([C@@H](C(=O)O)N)O)O)O | 0 | train |
| 170465889 | COC1=CC(=CC(=C1OC)OC)C(=O)NC2CCCNC2 | 0 | validation |
| 170465888 | COC1=CC=C(C=C1)CCN2CCC(CC2)NC3=NC4=CC=CC=C4N3CC5=CC=C(C=C5)F | 1 | test |
| 170465887 | CC(=O)N(CC(=O)N(C)C)C1=CC=CC=C1 | 0 | train |
| 170465886 | C1C(S/C(=C(/C#N)¥N2C=CN=C2)/S1)C3=CC=CC=C3Cl | 1 | train |
| 170465885 | CCOC1=CC=C(C=C1)NC(=O)CC(C)O | 0 | validation |
| 170465884 | CCCSSC(=C(C)N(CC1=CN=C(N=C1N)C)C=O)CCO | 0 | test |
| 170465883 | CC1(CCC(C2=C1C=CC(=C2)NC(=O)C3=CC=C(C=C3)C(=O)O)(C)C)C | 0 | train |
| 170465882 | CC1CCC2=C3N1C=C(C(=O)C3=CC(=C2N4CCC(CC4)O)F)C(=O)O | 0 | train |
| 170465881 | CCOCC(COC1=CC=C(C=C1)NC(=O)CC[S+](C)C)O.CC1=CC=C(C=C1)S(=O)(=O)[O-] | 0 | validation |
| 170465880 | C[C@H](C1=CC=CC=N1)C2=C(CC3=CC=CC=C32)CCN(C)C.C(=C¥C(=O)O)¥C(=O)O | 0 | test |
| 170465879 | CC(C)OC(=O)C(=C1SC=CS1)C(=O)OC(C)C | 1 | train |
| 170465878 | CN1C[C@@H](C[C@]2([C@H]1CC3=CN(C4=CC=CC2=C34)C)OC)COC(=O)C5=CC(=CN=C5)Br | 0 | train |
| 170465877 | CCN(CC)CCNC(=O)C1=C(C=CC(=C1)S(=O)(=O)C)OC.Cl | 0 | validation |

| | | | |
|---|---|---|---|
| 170465876 | CCC(=O)O[C@@H]1C[C@H]2[C@@H]3CCC4=C([C@H]3CC[C@@]2([C@H]1OC(=O)CC)C)C=CC(=C4)OC(=O)CC | 0 | test |
| 170465875 | CO[C@H]1[C@@H](C[C@@H]2CN3CCC4=C([C@H]3C[C@@H]2[C@@H]1C(=O)OC)NC5=C4C=CC(=C5)OC)OC(=O)/C=C/C6=CC(=C(C(=C6)O)OC | 0 | train |
| 170465874 | C[C@@H]1CC2=C(CCC(=O)C2)[C@@H]3[C@@H]1[C@@H]4CC[C@]([C@]4(CC3)C)(C#C)O | 0 | train |
| 170465873 | C1CN(CCC1(C2=CC=C(C=C2)Br)O)CCCC(=O)C3=CC=C(C=C3)F | 0 | validation |
| 170465872 | CCO/C(=N/C1=C[N+](=NO1)N2CCOCC2)/[O-] | 0 | test |
| 170465871 | CN1CCN(CC1)CCCN2C3=CC=CC=C3SC4=C2C=C(C=C4)S(=O)(=O)N(C)C.CS(=O)(=O)O.CS(=O)(=O)O | 1 | train |
| 170465870 | C1[C@@H]([C@H](O[C@H]1N2C=C(C(=O)NC2=O)Br)CO)O | 1 | train |
| 170465869 | C1CCN(C1)C2=C(C(=CC(=C2)C(=O)O)S(=O)(=O)N)OC3=CC=CC=C3 | 0 | validation |
| 170465868 | C1CSSC1CCCCC(=O)N | 0 | test |
| 170465867 | CC1=C(C(C(=C(N1)C)C(=O)OC/C=C/C2=CC=CC=C2)C3=CC(=CC=C3)[N+](=O)[O-])C(=O)OCCOC | 1 | train |
| 170465866 | CC1=CC(=CC=C1)N(C)C(=S)OC2=CC3=C(C=C2)C4CCC3C4 | 1 | train |
| 170465865 | C1CCN(CC1)CCC(C2=CC=CC=C2)(C3=CC=CC=C3)O | 0 | validation |
| 170465864 | CCOC(=O)C1=C(SC2=C1CCN(C2)CC3=CC=CC=C3)N | 0 | test |
| 170465863 | CC1=C(C(=O)N(N1C)C2=CC=CC=C2)N(C)C | 0 | train |
| 170465862 | CC1=C2C=CC(=C2C=C(C=C1)C(C)C)C | 1 | train |
| 170465861 | C[C@@H]1[C@H]([C@H]([C@@H](O1)N2C=C(C(=O)NC2=O)F)O)O | 0 | validation |
| 170465860 | CC1(C(=O)NC(=O)N(C1=O)C)C2=CCCCC2 | 0 | test |
| 170465859 | CC[C@@]12CCCN3[C@@H]1C4=C(CC3)C5=CC=CC=C5N4C(=C2)C(=O)OCC | 1 | train |
| 170465858 | C[C@]12CC[C@H]3[C@H]([C@@H]1CC[C@@H]2O)CC=C4[C@@]3(CC[C@@H](C4)O)C | 0 | train |
| 170465857 | C1=CC=C(C(=C1)C(=O)O)NC2=CC=CC(=C2)C(F)(F)F | 0 | validation |
| 170465856 | C1CN(CCC12C(=O)NCN2C3=CC=CC=C3)CCCC(=O)C4=CC=C(C=C4)F | 0 | test |
| 170465855 | CS(=O)(=O)O.C1=CC(=CC=C1C(=O)OC2=CC3=C(C=C2)C=C(C=C3)C(=N)N)N=C(N)N | 0 | train |
| 170465854 | COC1=CC=C(C=C1)C(=O)N2CCCC2=O | 0 | train |
| 170465853 | COC1=CC(=C(C=C1C(=O)NC2CCN(CC2)CC3=CC=CC=C3)Cl)N.C(=C¥C(=O)O)¥C(=O)O | 1 | validation |
| 170465852 | CCC1=C(C2=CC=CC=C2O1)C(=O)C3=CC(=C(C(=C3)I)O)I | 0 | test |
| 170465851 | CCOC(CN1CCN(CC1)CC(C)C(=O)C2=CC=CC=C2)C3=CC=CC=C3.Cl.Cl | 0 | train |
| 170465850 | CCOC(=O)OC1=C(C=C(C=C1)CCNC(=O)[C@H](CCSC)NC(=O)C)OC(=O)OCC | 0 | train |
| 170465849 | CC1N2C3=CC(=C(C=C3C(=O)C(=C2S1)C(=O)O)F)N4CCN(CC4)CC5=C(OC(=O)O5)C | 0 | validation |
| 170465848 | CC1=NC=C(C(=N1)N)CN(C=O)/C(=C¥2/CCOC(=O)S2)/C | 0 | test |
| 170465847 | C/C(=C(¥C1=C(C=C(C=C1)Cl)Cl)/OCCOC2=CC=C(C=C2)Cl)/N3C=CN=C3 | 0 | train |
| 170465846 | CC(C)C(CC(C)N(C)C)(C#N)C1=CC=CC=C1 | 0 | train |
| 170465845 | CS(=O)(=O)C1=CC=C(C=C1)[C@H]([C@@H](CO)NC(=O)C(Cl)Cl)O | 0 | validation |
| 170465844 | CC1=C(C(C(=C(N1)C)C(=O)OCCC2=CC=C(C=C2)N3CCN(CC3)C(C4=CC=CC=C4)C5=CC=CC=C5)C6=CC(=CC=C6)[N+](=O)[O-])C(=O)OC | 0 | test |
| 170465843 | CCOC1=C(C=NN(C1=O)C)N2CCOCC2 | 0 | train |
| 170465842 | CCN(CC)CCCN(C1CC2=CC=CC=C2C1)C3=CC=CC=C3.Cl | 0 | train |
| 170465841 | CCCCCCCC(=O)OCC(COC(=O)CCCCCCC)OC(=O)CCCCCCC | 0 | validation |
| 170465840 | CC1COC2(N1CC(=O)NC3=C2C=C(C=C3)Cl)C4=CC=CC=C4Cl | 0 | test |
| 170465839 | CC1=NC=C(C(=C1O)CO)CSSCC2=CN=C(C(=C2CO)O)C.Cl.Cl | 0 | train |
| 170465838 | CC(C1=CC2=C(C=C1)SC3=CC=CC=C3C(=O)C2)C(=O)O | 0 | train |
| 170465837 | C[N+](C)(C)CCCCCC[N+](C)(C)C | 0 | validation |
| 170465836 | C[C@]12CC[C@H]3[C@H]([C@@H]1CC[C@@H]2O)CC[C@]45[C@@]3(CC(=C([C@H]4O5)O)C#N)C | 0 | test |
| 170465835 | C1=CC=C(C=C1)C2=CC=C(C=C2)CC(=O)O | 0 | train |
| 170465834 | CCN1CCC[C@H]1CNC(=O)C2=C(C=CC(=C2)S(=O)(=O)N)OC | 0 | train |
| 170465833 | CCCCCCNC(=O)N1C=C(C(=O)NC1=O)F | 0 | validation |
| 170465832 | C1CCN(CC1)CCOC(C2=CC=CC=C2)C3=CC=C(C=C3)Cl.Cl | 0 | test |
| 170465831 | COC1=CC(=CC(=C1OC)OC)C(=O)OCCCN2CCCN(CC2)CCCOC(=O)C3=CC(=C(C(=C3)OC)OC)OC | 0 | train |
| 170465830 | CCOC(=O)C1=CC=C(C=C1)OC(=O)CCCCCN=C(N)N.CS(=O)(=O)O | 0 | train |
| 170465829 | CCC1(C(=O)NC(=O)NC1=O)CC | 0 | validation |
| 170465828 | CC(C)C(C(=O)NC(=O)N)Br | 0 | test |
| 170465827 | CC1=CC(=NC(=N1)N2C(=CC(=N2)C)OC)OC | 0 | train |
| 170465826 | C1=CC=C(C=C1)C2=CC=C(C=C2)C(C3=CC=CC=C3)N4C=CN=C4 | 1 | train |
| 170465825 | CC(C)(C)NCC(COC1=CC=CC=C1OCC2CCCO2)O | 0 | validation |
| 170465824 | C[N+]1(CCCCC1)CC2COC(O2)(C3CCCCC3)C4=CC=CC=C4.[I-] | 0 | test |
| 170465823 | C[N+]1(CC(CC(=C(C2=CC=CS2)C3=CC=CS3)C1)OC)C.[Br-] | 0 | train |

| | | | |
|---|---|---|---|
| 170465822 | C[C@H]1[C@H]([C@H](C[C@@H](O1)O[C@H]2C[C@@](CC3=C(C4=C(C(=C23)O)C(=O)C5=C(C4=O)C=CC=C5OC)O)(C(=O)CO)O)N)O[C@@H]6CCCCO6 | 1 | train |
| 170465821 | CC1=C(C(=O)N(N1C)C2=CC=CC=C2)C(C)C | 1 | validation |
| 170465820 | CC[C@@H]([C@@H](C1=C2C=CC(=O)NC2=C(C=C1)O)O)NC(C)C | 0 | test |
| 170465819 | CC1=CC(=O)OC2=C1C=CC(=C2)O | 0 | train |
| 170465818 | CC(=C)[C@H]1CN[C@@H]([C@H]1CC(=O)O)C(=O)O | 0 | train |
| 170465817 | CCC(=O)C1=C(C=C(C=C1O)O)O | 0 | validation |
| 170465816 | C1=CC(=C(C=C1C2(C3=C(C(=C(C(=C3Br)Br)Br)Br)C(=O)O2)C4=CC(=C(C=C4)O)S(=O)(=O)[O-])S(=O)(=O)[O-])O.[Na+].[Na+] | 0 | test |
| 170465815 | CN(C)CCOC(=O)COC1=CC=C(C=C1)Cl.Cl | 0 | train |
| 170465814 | C1[C@@H]([C@H]([C@@H](C([C@@H]1N)O)O[C@H]2[C@@H]([C@@H]([C@H](O2)CO)O)O)O[C@@H]3[C@@H]([C@H]([C@@H]([C@H](O3)CN)O)O)N)N.OS(=O)(=O)O | 0 | train |
| 170465813 | CCCCOC1=CC=C(C=C1)CC(=O)NO | 0 | validation |
| 170465812 | CC(C)NCC(COC1=CC=CC=C1CC=C)O | 0 | test |
| 170465811 | CC(C1=CC2=C(C=C1)OC3=C(C2)C=CC=N3)C(=O)O | 0 | train |
| 170465810 | CC(=O)NC(CCC(=O)N)C(=O)O | 0 | train |
| 170465809 | C1CC(OC1)N2C=C(C(=O)NC2=O)F | 0 | validation |
| 170465808 | CC(C1=CC=C(C=C1)CC2CCCC2=O)C(=O)O | 0 | test |
| 170465807 | C[C@]12CC[C@H]3[C@H]([C@@H]1C[C@H]([C@@H]2O)O)CCC4=C3C=CC(=C4)O | 0 | train |
| 170465806 | CN1CCC2=CC3=C(C(=C2C1)OC)OCO3.Br | 1 | train |
| 170465805 | C1=CC=C2C(=C1)C(OS2(=O)=O)(C3=CC=C(C=C3)O)C4=CC=C(C=C4)O | 0 | validation |
| 170465804 | C1=CC(=C(C(=C1CNNC(=O)C(CO)N)O)O)O.Cl | 0 | test |
| 170465803 | CN(CC1=CC(=CC(=C1N)Br)Br)C2CCCCC2.Cl | 0 | train |
| 170465802 | CC1=CC(=C(C=C1)Cl)OCC(CNC(C)(C)C)O | 0 | train |
| 170465801 | CC1=C(C=C2C(=C1)CCCS2(=O)=O)S(=O)(=O)N | 0 | validation |
| 170465800 | C(C(C(=O)O)N)SCC(=O)O | 0 | test |
| 170465799 | C1=CC=C(C=C1)C2=CC=C(C=C2)C(=O)CCC(=O)O | 0 | train |
| 170465798 | CCOC1=CC2=C(C3=C(C=C(C=C3)N)N=C2C=C1)N.CC(C(=O)O)O.O | 0 | train |
| 170465797 | COC1=C(C(=C(C=C1)CN2CCNCC2)OC)OC.Cl.Cl | 0 | validation |
| 170465796 | CCC1=CC=C(C=C1)C(=O)C(C)CN2CCCCC2.Cl | 0 | test |
| 170465795 | CCCC1=NC=CC(=C1)C(=S)N | 0 | train |
| 170465794 | C1CN2CCC1C(C2)CN3C4=CC=CC=C4SC5=CC=CC=C53 | 1 | train |
| 170465793 | CN1CCN(CC1)CC(=O)N2C3=CC=CC=C3C(=O)NC4=C2N=CC=C4 | 0 | validation |
| 170465792 | CCC1=C(C2=CC=CC=C2O1)C(=O)C3=CC(=C(C(=C3)Br)O)Br | 0 | test |
| 170465791 | CC(C(C1=CC=C(C=C1)O)O)N2CCC(CC2)CC3=CC=CC=C3 | 0 | train |
| 170465790 | C1CC(C(C(C1)(COC(=O)C2=CN=CC=C2)COC(=O)C3=CN=CC=C3)O)(COC(=O)C4=CN=CC=C4)COC(=O)C5=CN=CC=C5 | 0 | train |
| 170465789 | C1=CC(=CN=C1)C(=O)OCC(COC(=O)C2=CN=CC=C2)(COC(=O)C3=CN=CC=C3)COC(=O)C4=CN=CC=C4 | 0 | validation |
| 170465788 | C1CCS(=O)(=O)N(C1)C2=CC=C(C=C2)S(=O)(=O)N | 0 | test |
| 170465787 | C1COC2(N1CC(=O)NC3=C2C=C(C=C3)Cl)C4=CC=CC=C4Cl | 1 | train |
| 170465786 | CN1CCCN(CC1)C(C2=CC=CC=C2)C3=CC=C(C=C3)Cl | 1 | train |
| 170465785 | C1CCN(CC1)C2(CCN(CC2)CCCN3C4=CC=CC=C4CCC5=C3C=C(C=C5)Cl)C(=O)N | 1 | validation |
| 170465784 | CN(C)C(=O)COC(=O)CC1=CC=C(C=C1)OC(=O)C2=CC=C(C=C2)N=C(N)N.CS(=O)(=O)O | 0 | test |
| 170465783 | C1CC(CCC1CN)C(=O)OC2=CC=C(C=C2)CCC(=O)O.Cl | 0 | train |
| 170465782 | CCOC(=O)NNC1=NN=CC2=CC=CC=C21.Cl | 0 | train |
| 170465781 | CC(C)(C)NCC(C1=CC=CC=C1Cl)O.Cl | 0 | validation |
| 170465780 | CC1=C2C(=C(C(=C1C)OC(=O)C3=CN=CC=C3)C)CC[C@@](O2)(C)CCC[C@H](C)CCC[C@H](C)CCCC(C)C | 0 | test |
| 170465779 | CC1(CO[C@@H]([C@@H]([C@H]1NC)O)O[C@H]2[C@@H](C[C@@H]([C@H]([C@H]2O)O[C@@H]3[C@@H](CC=C(O3)CN)N)N)N)O | 0 | train |
| 170465778 | C=CCN1CC[C@]23CCCC[C@H]2[C@H]1CC4=C3C=C(C=C4)O | 0 | train |
| 170465777 | CC1=C(OC(=O)O1)COC(=O)[C@H]2C(S[C@H]3N2C(=O)[C@H]3NC(=O)[C@@H](C4=CC=CC=C4)N)(C)C.Cl | 0 | validation |
| 170465776 | CC(=O)[C@]1(CC[C@@H]2[C@@]1(CC[C@H]3[C@H]2C=C(C4=CC(=O)CC[C@]34C)Cl)C)OC(=O)C | 0 | test |
| 170465775 | CCOC1=CC=CC=C1C(=O)N | 0 | train |
| 170465774 | CCCN(CCC)C(=O)C(CCC(=O)O)NC(=O)C1=CC=CC=C1 | 0 | train |
| 170465773 | CCCCN(CCN(CCCC)C(=O)N1CCOCC1)C(=O)N2CCOCC2 | 0 | validation |
| 170465772 | CCOC1=CC(=C(C=C1C(=O)CCC(=O)O)OCC)OCC | 0 | test |
| 170465771 | CCC(C)(C(=O)O)OC1=CC=C(C=C1)C2(CCCCC2)C3=CC=C(C=C3)OC(C)(CC)C(=O)O | 0 | train |
| 170465770 | CCN(CC)C1=CC(=NC2=NC=NN12)C | 0 | train |

| | | | |
|---|---|---|---|
| 170465769 | CC1=CC=C(C=C1)C(=O)C(C)CN2CCCCC2.Cl | 0 | validation |
| 170465768 | COC1=NC=NC(=C1)NS(=O)(=O)C2=CC=C(C=C2)N | 0 | test |
| 170465767 | CCN1C=C(C(=O)C2=CN=C(N=C21)N3CCNCC3)C(=O)O | 0 | train |
| 170465766 | C1=CC=C2C(=C1)C(=O)NS2(=O)=O | 0 | train |
| 170465765 | CN(C)C(=O)OC1=CC=CC(=C1)[N+](C)(C)C.[Br-] | 0 | validation |
| 170465764 | C(CO)CSC[C@@H](C(=O)O)N | 0 | test |
| 170465763 | C[C@H]1[C@@H]([C@H]([C@H]([C@@H](O1)O[C@H]2C[C@H]([C@@]3([C@@H]4[C@@H](CC[C@@]3(C2)O)[C@]5(CC[C@@H]([C@]5(C[C@H]4O)C)C6=CC(=O)OC6)O)CO)O)O)O)O | 1 | train |
| 170465761 | C1=C(NC(=O)NC1=O)C(=O)O | 0 | validation |
| 170465760 | CN(C)CCCOC1(CCCCCC1)CC2=CC=CC=C2 | 0 | test |
| 170465759 | CC1=CC(=C(C=C1OC(=O)C)C(C)C)OCCN(C)C.Cl | 0 | train |
| 170465758 | C/C(=N/NC(=O)C1=CC=NC=C1)/C(=O)O | 0 | train |
| 170465757 | C[C@@]12CCN([C@@H]1N(C3=C2C=C(C=C3)OC(=O)NC)C)C | 0 | validation |
| 170465756 | CC1=C(C(C(=C(N1)N)C(=O)OC2CN(C2)C(C3=CC=CC=C3)C4=CC=CC=C4)C5=CC(=CC=C5)[N+](=O)[O-])C(=O)OC(C)C | 1 | test |
| 170465755 | C1=CC(=C(C=C1C(CNCCCCCCNCC(C2=CC(=C(C=C2)O)O)O)O)O)O.OS(=O)(=O)O | 0 | train |
| 170465754 | CC(C)C[C@@H]1[C@@H](OC(=O)N1CCCN2CCCCCC2)C3=CC=CC=C3.Cl | 0 | train |
| 170465753 | CCC(=O)O[C@@]1([C@H](C[C@@H]2[C@@]1(C[C@@H](C3([C@H]2CCC4=CC(=O)C=C[C@@]43C)F)O)C)C)C(=O)COC | 0 | validation |
| 170465752 | COC1=C(C2=C[N+]3=C(C=C2C=C1)C4=CC5=C(C=C4CC3)OCO5)OC.[Cl-] | 1 | test |
| 170465751 | C[C@@]12CC[C@H]3[C@]([C@@]14[C@H](O4)C(=O)O[C@H]2C5=COC=C5)(C(=O)C[C@@H]6[C@@]37COC(=O)C[C@@H]7OC6(C)C)C | 0 | train |
| 170465750 | C[C@@H]1C[C@H]2[C@@H]3C[C@@H](C4=CC(=O)C=C[C@@]4([C@]3([C@H](C[C@@]2([C@]1(C(=O)CO)O)C)O)F)C)F | 0 | train |
| 170465749 | C1=CC=C(C=C1)C(CC(C2=CC=C(C=C2)C3=CC=C(C=C3)Br)O)C4=C(C5=CC=CC=C5OC4=O)O | 0 | validation |
| 170465748 | C(C[C@@H](C(=O)O)N)CN=C(N)N | 0 | test |
| 170465747 | CC1=CNC2=CC=CC=C12 | 0 | train |
| 170465746 | C1=CC=C2C(=C1)C(=O)/C(=C¥3/C(=O)C4=CC=CC=C4N3)/N2 | 1 | train |
| 170465745 | CC1CC(=O)NN=C1C2=CC3=C(C=C2)N=C(N3)C4=CC=C(C=C4)OC.Cl | 1 | validation |
| 170465744 | C1=CC(=CC=C1NC(=O)NC2=CC=C(C=C2)[N+](=O)[O-])[N+](=O)[O-] | 0 | test |
| 170465743 | CC(C)(C(=O)O)OC1=CC=C(C=C1)CCNC(=O)C2=CC=C(C=C2)Cl | 0 | train |
| 170465742 | CC(=O)N[C@@H](CC1=CC=C(C=C1)O)C(=O)O | 0 | train |
| 170465741 | C1CN(C(=N1)N[N+](=O)[O-])CC2=CN=C(C=C2)Cl | 0 | validation |
| 170465740 | C1CCN2C[C@@H]3C[C@H]([C@H]2C1)CN4[C@H]3CCCC4 | 0 | test |
| 170465739 | C[C@@H]1CC(=O)C=C([C@]12C(=O)C3=C(O2)C(=C(C=C3OC)OC)Cl)OC | 0 | train |
| 170465738 | C(CC(=O)O)C(=O)O | 0 | train |
| 170465737 | C1CCC(CC1)C(=O)N2CC3C4=CC=CC=C4CCCN3C(=O)C2 | 0 | validation |
| 170465736 | C1=CC(=C(C(=C1)F)C(=O)NC(=O)NC2=CC(=C(C=C2Cl)OC(C(C(F)(F)F)F)(F)F)Cl)F | 0 | test |
| 170465735 | CCCCOC(=O)C1=CC=C(C=C1)N | 1 | train |
| 170465734 | CCC(COC(=O)C1=CC(=C(C(=C1)OC)OC)OC)(C2=CC=CC=C2)N(C)C.C(=C¥C(=O)O)¥C(=O)O | 1 | train |
| 170465733 | C1=CC=C2C(=C1)C=CN2 | 0 | validation |
| 170465732 | CN1CCN(CC1)C2=C(C=C3C4=C2OCN(N4C=C(C3=O)C(=O)O)C)F | 0 | test |
| 170465731 | CC(=O)C1=CC2=C(C=C1)SC3=CC=CC=C3N2CCCN(C)C.C(=C¥C(=O)O)¥C(=O)O | 0 | train |
| 170465730 | CCCCCCCCCCCCCCCCCCOCC(CO)O | 0 | train |
| 170465729 | CCCCC(CC)CN1CC(CN(C1)CC(CC)CCCC)(C)N | 0 | validation |
| 170465728 | C[C@H]1[C@@H]2CC[C@]3(C=CC(=O)C(=C3[C@H]2OC1=O)C)C | 1 | test |
| 170465727 | CC1=C(C=C(C=C1C(=O)N)[N+](=O)[O-])[N+](=O)[O-] | 0 | train |
| 170465726 | C(CS(=O)(=O)O)N | 0 | train |
| 170465725 | C1=CC=C(C=C1)CC2=C(C=CC(=C2)Cl)O | 1 | validation |
| 170465724 | C1=CC=C(C=C1)C2=CC=CC=C2[O-].O.O.O.O.[Na+] | 0 | test |
| 170465723 | C1=CC=C2C(=C1)N=C(S2)SSC3=NC4=CC=CC=C4S3 | 0 | train |
| 170465722 | C1=CC(=CC=C1O)O[C@H]2[C@@H]([C@H]([C@@H]([C@H](O2)CO)O)O)O | 0 | train |
| 170465721 | C1=CC(=CC=C1NC(=O)CCC(=O)O)S(=O)(=O)NC2=NC=CS2 | 0 | validation |
| 170465720 | CC1=CC2=C(C=C1CC(=O)C3=C(C=CS3)S(=O)(=O)NC4=C(C(=NO4)C)Cl)OCO2 | 0 | test |
| 170465719 | C1=CC=C2C(=C1)C(=O)OC23C4=CC(=C(C(=C4OC5=C(C(=C(C=C35)I)O)I)I)O)I | 0 | train |
| 170465718 | CC1=CC(=C(C=C1CC2=CN=C(N=C2N)N)OC)OC | 0 | train |
| 170465717 | CCOC(=O)C1=CC(=CC=C1)N.CS(=O)(=O)O | 0 | validation |
| 170465716 | C1=CC(=CC=C1[N+](=O)[O-])[As](=O)(O)O | 0 | test |
| 170465715 | CCOC(=O)C1=CC=C(C=C1)O | 0 | train |
| 170465714 | CCCCCCCCCCCCCCCC[N+](C)(C)C.[Br-] | 0 | train |

| | | | |
|---|---|---|---|
| 170465713 | C1CSSC1CCCCC(=O)O | 0 | validation |
| 170465712 | CN(C)C1=CC=C(C=C1)C(=C2C=CC(=[N+](C)C)C=C2)C3=CC=CC=C3.CN(C)C1=CC=C(C=C1)C(=C2C=CC(=[N+](C)C)C=C2)C3=CC=CC=C3.C(=O)(C(=O)O)O.C(=O)(C(=O)[O-])O.C(=O)(C(=O)[O-])O | 1 | test |
| 170465711 | C1=CC=C(C=C1)COC(=O)C2=CC=C(C=C2)O | 1 | train |
| 170465710 | CC1=CC=CC=C1N=NC2=CC(=C(C=C2)N/N=C/3¥C(=O)C=CC4=CC=CC=C43)C | 1 | train |
| 170465709 | C1CCN(CC1)C(=S)SSC(=S)N2CCCCC2 | 1 | validation |
| 170465708 | C1=CC(=CC=C1N)S(=O)(=O)N=C(N)N | 0 | test |
| 170465707 | COP(=O)(C(C(Cl)(Cl)Cl)O)OC | 0 | train |
| 170465706 | CN1CCC2=CC3=C(C=C2[C@H]1[C@H]4C5=C(C(=C(C=C5)OC)OC)C(=O)O4)OCO3 | 0 | train |
| 170465705 | CC[C@H]1CN2CCC3=CC(=C(C=C3[C@@H]2C[C@@H]1C[C@@H]4C5=CC(=C(C=C5CCN4)OC)OC)OC)OC | 1 | validation |
| 170465704 | C[C@]12C3CCC([C@]1(C(=O)OC2=O)C)O3 | 0 | test |
| 170465703 | CC1=CC(=C(C=C1)N=CN(C)C=NC2=C(C=C(C=C2)C)C)C | 0 | train |
| 170465702 | CC1=C[C@@H]2[C@H](CC[C@]3([C@H]2CC(=C)[C@@]3(C(=O)C)OC(=O)C)C)[C@@]4(C1=CC(=O)CC4)C | 0 | train |
| 170465701 | CC1=NC2=C(C=C1)C(=CC(=C2O)Cl)Cl | 0 | validation |
| 170465700 | CCCCC(=O)OCC(=O)[C@H]1[C@@H](C[C@@H]2[C@@]1(C[C@@H]([C@]3([C@H]2C[C@@H](C4=CC(=O)C=C[C@@]43C)F)F)O)C)C | 0 | test |
| 170465699 | C1=CC=[N+](C(=C1)SSC2=CC=CC=[N+]2[O-])[O-] | 1 | train |
| 170465698 | CC1=CC=C(C=C1)S(=O)(=O)NC(=O)NN2CC3CCCC3C2 | 0 | train |
| 170465697 | C1CN(CCN1CCCC(=O)C2=CC=C(C=C2)F)C3=CC=CC=N3 | 0 | validation |
| 170465696 | CC1=C(SC=C1)/C=C/C2=NCCCN2C.[C@@H]([C@@H](C(=O)O)O)(C(=O)O)O | 0 | test |
| 170465695 | CNC(=O)OC1=CC=CC2=CC=CC=C21 | 1 | train |
| 170465694 | CNCCC1=CC=CC=N1.CS(=O)(=O)O | 0 | train |
| 170465693 | CCCC1CCCCN1 | 0 | validation |
| 170465692 | CC(=CCC/C(=C/CO)/C)C | 0 | test |
| 170465691 | C[C@H]1[C@@H]([C@H]([C@H]([C@@H](O1)OC[C@@H]2[C@H]([C@@H]([C@H]([C@@H](O2)OC3=C(OC4=CC(=CC(=C4C3=O)O)O)C5=CC(=C(C=C5)O)O)O)O)O)O)O)O | 0 | train |
| 170465690 | COC1=NC(=NC(=C1)NS(=O)(=O)C2=CC=C(C=C2)N)OC | 0 | train |
| 170465689 | CC(C)(C)C1=CC=C(C=C1)O | 0 | validation |
| 170465688 | CCCCOC(=O)C1=CC=C(C=C1)O | 0 | test |
| 170465687 | COC1=C(C=C(C=C1)CC2=NC=CC3=CC(=C(C=C32)OC)OC)OC.Cl | 1 | train |
| 170465686 | C12=C(NC(=O)NC1=O)[N-]C(=O)N2.[Na+] | 0 | train |
| 170465685 | CCCC[N+]1([C@@H]2CC(C[C@H]1[C@H]3[C@@H]2O3)OC(=O)[C@H](CO)C4=CC=CC=C4)C.[Br-] | 0 | validation |
| 170465684 | COC1=CC=CC=C1OC(=O)OC2=CC=CC=C2OC | 0 | test |
| 170465683 | CCCCCCCCCCCCCC(=O)OC(C)C | 0 | train |
| 170465682 | CC(=O)[C@]1(CC[C@@H]2[C@@]1(CC[C@H]3[C@H]2C=C(C4=CC(=O)[C@@H]5C[C@@H]5[C@]34C)Cl)C)OC(=O)C | 0 | train |
| 170465681 | C1=CC=C(C(=C1)C(=O)OCC(CO)O)NC2=C3C=CC=C(C3=NC=C2)C(F)(F)F | 0 | validation |
| 170465680 | C=CCNC(=S)N | 0 | test |
| 170465679 | COC1=C(C=C(C=C1)C2=CC(=NN2C3=CC=C(C=C3)S(=O)(=O)N)C(F)F)F | 0 | train |
| 170465678 | CC=CC1=CC=C(C=C1)OC | 0 | train |
| 170465677 | CN1C(=C(C2=C(S1(=O)=O)C=CS2)O)C(=O)NC3=CC=CC=N3 | 0 | validation |
| 170465676 | CN1CCCN=C1/C=C/C2=CC=CS2.[C@@H]([C@H](C(=O)O)O)(C(=O)O)O | 0 | test |
| 170465675 | CCCC1=NC=C(C(=N1)N)C[N+]2=CC=CC=C2C.Cl.[Cl-] | 0 | train |
| 170465674 | CCC(=O)C(CCN(C)C)(C1=CC=CC=C1)C2=CC=CC=C2 | 1 | train |
| 170465673 | C1=CC(=CC=C1O)Cl | 0 | validation |
| 170465672 | CC1=C(C(=O)N(N1C)C2=CC=CC=C2)N(C)CS(=O)(=O)[O-].[Na+] | 0 | test |
| 170465671 | C1=CC=C(C=C1)OCCO | 0 | train |
| 170465670 | C[N+]1=C2C=C(C=CC2=CC3=C1C=C(C=C3)N)N.C1=CC(=CC2=NC3=C(C=CC(=C3)N)C=C21)N.[Cl-] | 0 | train |
| 170465669 | COC1=CC=C(C=C1)C2=CC(=S)SS2 | 1 | validation |
| 170465668 | CC1=C(C(=O)C(=C(N1)C)Cl)Cl | 0 | test |
| 170465667 | C1C(=O)NC2=C(C=C(C=C2)[N+](=O)[O-])C(=N1)C3=CC=CC=C3 | 0 | train |
| 170465666 | C1=CC(=C(C=C1[As](=O)(O)O)[N+](=O)[O-])O | 0 | train |
| 170465665 | C1=CC=C2C(=C1)C=CC(=O)O2 | 0 | validation |
| 170465664 | CN(C)C(=S)SSC(=S)N(C)C | 1 | test |
| 170465663 | C1COCCN1CCNC(=O)C2=CC=C(C=C2)Cl | 0 | train |
| 170465662 | CCC(CC)(CNC(=O)CCCO)C1=CC(=CC=C1)OC | 0 | train |
| 170465661 | CCCOC1=CC2=C(C=C1)N=C(N2)NC(=O)OC | 0 | validation |

| | | | |
|---|---|---|---|
| 170465660 | CC1OC(OC(O1)C)C | 0 | test |
| 170465659 | CC1=CC(=C2C(=C1)C=C3C=CC=C(C3=C2O)O)O | 1 | train |
| 170465658 | CC(C)CCCC(C)CCCC(C)CCCCC(C)CCCC(C)CCCC(C)C | 0 | train |
| 170465657 | CC(CC1=CC=C(C=C1)O)NCC(C2=CC(=CC(=C2)O)O)O.Br | 0 | validation |
| 170465656 | C1=CC=C(C=C1)NC(=NC2=CC=CC=C2)N | 0 | test |
| 170465655 | C1=NC(=O)C2=C(N1)N(C=N2)[C@H]3[C@@H]([C@@H]([C@H](O3)CO)O)O | 0 | train |
| 170465654 | CC=CC1=CC(=C(C=C1)O)OC | 0 | train |
| 170465653 | C1=CC=C2C(=C1)N=CC(=N2)NS(=O)(=O)C3=CC=C(C=C3)N | 0 | validation |
| 170465652 | CCCCCCCCCCCCCCCCC(C(=O)O)C(CC(=O)O)(C(=O)O)O | 0 | test |
| 170465651 | CC(=O)CCC(=O)[O-].CC(=O)CCC(=O)[O-].O.O.[Ca+2] | 0 | train |
| 170465650 | C1=CC=C(C=C1)[NH3+].[N+](=O)(O)[O-] | 0 | train |
| 170465649 | C1=CC=C(C=C1)C(=O)C2=CC=CC=C2 | 0 | validation |
| 170465648 | CS(=O)(=O)C1=CC=C(C=C1)[C@H]([C@@H](CF)NC(=O)C(Cl)Cl)O | 0 | test |
| 170465647 | COC(=O)NC1=NC2=C(N1)C=C(C=C2)SC3=CC=CC=C3 | 1 | train |
| 170465646 | C[C@]12C=CC3=C4CCC(=O)C=C4CC[C@H]3[C@@H]1CC[C@]2(CC=C)O | 0 | train |
| 170465645 | CC(COC1=CC=CC=C1)O | 0 | validation |
| 170465644 | C[C@@H]1CN(C[C@@H](N1)C)C2=C(C3=C(C(=C2F)F)C(=O)C(=CN3C4CC4)C(=O)O)F | 0 | test |
| 170465643 | CC(C)(CC1=CC=CC=C1)N(C)C(=O)CN(CCO)CC(=O)N(C)C(C)(C)CC2=CC=CC=C2 | 0 | train |
| 170465642 | CN1[C@@H]2CC[C@H]1CC(C2)OC(=O)C(C3=CC=CC=C3)O.Br | 0 | train |
| 170465641 | C1=CC=C2C(=C1)C(=O)C3=C(C2=O)C(=C(C=C3)O)O | 0 | validation |
| 170465640 | C1=CC2=C(C(=C(C=C2I)I)O)N=C1 | 1 | test |
| 170465639 | CC1=C(C(=CC=C1)CC2=CN=CN2)C.Cl | 0 | train |
| 170465638 | CC1=C(C=CC=C1Cl)NC2=CC=CC=C2C(=O)O | 0 | train |
| 170465637 | CC(CCC1=CC=CC=C1)NC(C)C(C2=CC=C(C=C2)O)O | 0 | validation |
| 170465636 | CC(=O)CC(C1=CC=C(C=C1)[N+](=O)[O-])C2=C(C3=CC=CC=C3OC2=O)O | 0 | test |
| 170465635 | C1=CC(=CC=C1N)[As](=O)(O)O | 0 | train |
| 170465634 | C1(=O)N(C(=O)N(C(=O)N1Cl)Cl)Cl | 0 | train |
| 170465633 | C1=C(C=C(C(=C1O)O)O)C(=O)O | 0 | validation |
| 170465632 | C(C(C(=O)[O-])O)C(=O)[O-].[Na+].[Na+] | 0 | test |
| 170465631 | CCN(CC)C(=O)N1CCN(CC1)C.C(C(=O)O)C(CC(=O)O)(C(=O)O)O | 0 | train |
| 170465630 | CCCCCCCCCCCCCCCCCC(=O)O | 0 | train |
| 170465629 | C1=CC(=CC=C1C=NN/C(=N/N=CC2=CC=C(C=C2)Cl)/N)Cl | 0 | validation |
| 170465628 | CC(=O)OCC(=O)[C@]1(CC[C@@H]2[C@@]1(C[C@@H]([C@]3([C@H]2CCC4=CC(=O)C=C[C@@]43C)F)O)C)O | 0 | test |
| 170465627 | C[C@H]([C@H](C1=CC=CC=C1)O)N.Cl | 0 | train |
| 170465626 | CCN[C@@H](C)CC1=CC(=CC=C1)C(F)(F)F.Cl | 0 | train |
| 170465625 | CC1=C(C=C(C=C1)NC2=NC=CC(=N2)N(C)C3=CC4=NN(C(=C4C=C3)C)C)S(=O)(=O)N | 1 | validation |
| 170465624 | CCCN1C=NC2=C1C(=O)N(C(=O)N2C)CCCCC(=O)C | 0 | test |
| 170465623 | CN1CCN(CC1)C2=C(C=C3C(=C2)N(C=C(C3=O)C(=O)O)C4=CC=C(C=C4)F)F.Cl | 0 | train |
| 170465622 | CCCCCCCCCCCCCCCCOP(=O)([O-])OCC[N+](C)(C)C | 0 | train |
| 170465621 | CC1=NC=C(C=C1)C2=NC=C(C=C2C3=CC=C(C=C3)S(=O)(=O)C)Cl | 0 | validation |
| 170465620 | CCOC1=CC=CC=C1O[C@@H]([C@H]2CNCCO2)C3=CC=CC=C3.CS(=O)(=O)O | 0 | test |
| 170465619 | CC(CC1=CC=CC=C1)NCC2=CC=CC=C2Cl.Cl | 1 | train |
| 170465618 | CCC(C1=CC=CC=C1)C(=O)OCCN2CCOC(C2C)C3=CC=CC=C3 | 1 | train |
| 170465617 | CN1CCC(CC1)COC2=C(C=C3C(=C2)N=CN=C3NC4=C(C=C(C=C4)Br)F)OC | 0 | validation |
| 170465616 | CCN(CC)CCOC(=O)C(CC1CCCO1)CC2=CC=CC3=CC=CC=C32.C(=O)(C(=O)O)O | 0 | test |
| 170465615 | CC[C@@]1(C2=C(COC1=O)C(=O)N3CC4=C(C5=CC=CC=C5N=C4C3=C2)/C=N/OC(C)(C)C)O | 0 | train |
| 170465614 | C[C@]12CC[C@H]3[C@H]([C@@H]1CCC2=O)CC=C4[C@@]3(CC[C@@H](C4)O)C | 0 | train |
| 170465613 | C1CN(CCC1C2=CN(C3=C2C=C(C=C3)Cl)C4=CC=C(C=C4)F)CCN5CCNC5=O | 1 | validation |
| 170465612 | CS(=O)(=O)NC1=C(C=C(C=C1)[N+](=O)[O-])OC2=CC=CC=C2 | 0 | test |
| 170465611 | CC1=C(C(=O)C(=C(C1=O)OC)OC)CCCCCCCCCCO | 0 | train |
| 170465610 | C1=CC(=CC(=C1)Cl)C(C2=CC3=C(C=C2)N=CN3)N4C=CN=C4.Cl | 1 | train |
| 170465609 | CN1C=C(C2=CC=CC=C21)C3=C(C(=O)NC3=O)C4=CN(C5=CC=CC=C54)C6CCN(CC6)CC7=CC=CC=N7 | 0 | validation |
| 170465608 | C1=NC(=C(N1[C@H]2[C@@H]([C@@H]([C@H](O2)CO)O)O)N)C(=O)N | 0 | test |
| 170465607 | CCCC1=NC(=C2N1NC(=NC2=O)C3=C(C=CC(=C3)S(=O)(=O)N4CCN(CC4)CC)OCC)C.C(C(=O)O)C(CC(=O)O)(C(=O)O)O | 0 | train |
| 170465606 | CC1=CN(C(=O)C=C1)C2=CC=CC=C2 | 0 | train |
| 170465605 | C[C@@H](CSC(=O)C1=CC=CC=C1)C(=O)N2C[C@H](C[C@H]2C(=O)O)SC3=CC=CC=C3 | 0 | validation |
| 170465604 | CN(C1CCN(CC1)C2=NC3=CC=CC=C3N2CC4=CC=C(C=C4)F)C5=NC=CC(=O)N5 | 0 | test |
| 170465603 | C1CN(CC=C1C2=CC(=CC=C2)C(F)(F)F)CCC3=CC4=CC=CC=C4C=C3.Cl | 0 | train |

| | | | |
|---|---|---|---|
| 170465602 | C1CCN(CC1)C[C@H](CO/N=C(/C2=C[N+](=CC=C2)[O-])¥Cl)O | 0 | train |
| 170465601 | CC(CC1=CC=CC=C1)NCCCCl | 0 | validation |
| 170465600 | C(CC(=O)N[C@@H](CS)C(=O)NCC(=O)O)[C@@H](C(=O)O)N | 0 | test |
| 170465599 | CC1=C(N(N=C1C(=O)NN2CCCCC2)C3=C(C=C(C=C3)Cl)Cl)C4=CC=C(C=C4)Cl | 0 | train |
| 170465598 | CC(C)(C)C1=CC=C(C=C1)C(CCCN2CCC(CC2)C(C3=CC=CC=C3)(C4=CC=CC=C4)O)O | 1 | train |
| 170465597 | CC1=CC(=C(C=C1)NC2=C(C=CC=C2Cl)F)CC(=O)O | 0 | validation |
| 170465596 | CN1CCN2C(C1)C3=CC=CC=C3CC4=CC=CC=C42 | 1 | test |
| 170465595 | C1CN(CCN1C[C@H](COC2=CC=CC3=C2C=CC=N3)O)C4C5=CC=CC=C5[C@H]6[C@H](C6(F)F)C7=CC=CC=C47.Cl.Cl.Cl | 0 | train |
| 170465594 | C1CN(CCC1N2C3=C(C=C(C=C3)Cl)NC2=O)CCCN4C5=CC=CC=C5NC4=O | 0 | train |
| 170465593 | C1=CC=C(C=C1)C2=C(C2=O)C3=CC=CC=C3 | 1 | validation |
| 170465592 | CC1CN(CCN1)C2=C(C=C3C(=C2OC)N(C=C(C3=O)C(=O)O)C4CC4)F | 0 | test |
| 170465591 | CC1=C(C2=C(C=C1)NC(=NC2=O)N)SC3=CC=NC=C3.Cl.Cl | 0 | train |
| 170465590 | CC1C(C(CC(O1)OC2C[C@@](CC3=C(C4=C(C(=C23)O)C(=O)C5=C(C4=O)C=CC=C5OC)O)(C(=O)CO)O)N6CCOC(C6)OC)O | 0 | train |
| 170465589 | CC(=O)NCCC1=CNC2=C1C=C(C=C2)OC | 0 | validation |
| 170465588 | CCCCCCCCCCCCCCCC(=O)O | 0 | test |
| 170465587 | CC1=C(C(C(=C(N1)C)C(=O)OC(C)(C)CN(C)CCC(C2=CC=CC=C2)C3=CC=CC=C3)C4=CC(=CC=C4)[N+](=O)[O-])C(=O)OC.Cl | 0 | train |
| 170465586 | C1CSC(=O)C1NC(=O)CSCC(=O)O | 0 | train |
| 170465585 | CC(=CC1C(C1(C)C)C(=O)OCC2=CC(=CC=C2)OC3=CC=CC=C3)C | 1 | validation |
| 170465584 | C1=CC=C(C=C1)COC(=O)C=CC2=CC=CC=C2 | 1 | test |
| 170465583 | CCOC(=O)N1C=CN(C1=S)C | 0 | train |
| 170465582 | C1=CC(=CN=C1)C(=O)NCCO[N+](=O)[O-] | 0 | train |
| 170465581 | CC1(C2=C(C=C(C=C2)OC)C(=O)N(C1=O)CCC3=CC=C(C=C3)S(=O)(=O)NC(=O)NC4CCCCC4)C | 0 | validation |
| 170465580 | CCOC(=O)C1=C(NC(=C(C1C2=CC=CC=C2/C=C/C(=O)OC(C)(C)C)C(=O)OCC)C)C | 1 | test |
| 170465579 | CN1CCOC(C2=CC=CC=C2C1)C3=CC=CC=C3.Cl | 0 | train |
| 170465578 | C[C@@H]1CC[C@H](C(=O)C1)C(C)C | 0 | train |
| 170465577 | CC(C1=CC=C(S1)C(=O)C2=CC=CC=C2)C(=O)O | 1 | validation |
| 170465576 | CN(C)CCCOC1=NN(C2=CC=CC=C21)CC3=CC=CC=C3.Cl | 0 | test |
| 170465575 | CCCC(=O)O[C@@]1([C@H](C[C@@H]2[C@@]1(CC(=O)[C@]3([C@H]2CCC4=CC(=O)C=C[C@@]43C)F)C)C)C(=O)CCl | 0 | train |
| 170465574 | C1=CC(=C(C=C1Cl)Cl)CO | 0 | train |
| 170465573 | C1=CC=C(C=C1)[C@H](C(=O)O)O | 0 | validation |
| 170465572 | CCN1CCN(CC1)C2=C(C=C3C(=C2)N(C=C(C3=O)C(=O)O)C4CC4)F | 0 | test |
| 170465571 | C1=CC=C(C(=C1)C(=O)O)S | 0 | train |
| 170465570 | C[C@@H](NCC(CN[C@@H](/C(=N/O)/C)C)(C)C)/C(=N/O)/C | 0 | train |
| 170465569 | CN1C[C@@H]2[C@@H](C1)C3=C(C=CC(=C3)Cl)OC4=CC=CC=C24 | 0 | validation |
| 170465568 | C1COCCN1SC2=NC3=CC=CC=C3S2 | 1 | test |
| 170465567 | CN(C)C(=S)SC(=S)N(C)C | 1 | train |
| 170465566 | C([C@H]([C@H]([C@@H]([C@@H](CO)O)O)O)O)O | 0 | train |
| 170465565 | C([C@H]([C@@H]([C@@H]([C@H](CO)O)O)O)O)O | 0 | validation |
| 170465564 | C([C@H]([C@@H]([C@@H]([C@H](CO)O)O)O)O)O | 0 | test |
| 170465563 | CCCOC(=O)C1=CC=CC=C1 | 0 | train |
| 170465562 | CCN(CC)CCOCCOC(=O)C1(CCCC1)C2=CC=CC=C2 | 0 | train |
| 170465561 | CCCCCCCCCCCCSCCOCCO | 0 | validation |
| 170465560 | CC(=O)/C=C/C=C¥1/C2CCC(C2)C1(C)C | 0 | test |
| 170465559 | C1=CC=C2C(=C1)C(=O)OC2(C3=CC=C(C=C3)O)C4=CC=C(C=C4)O | 1 | train |
| 170465558 | CC(=O)[C@H]1CC[C@@H]2[C@@]1(CC[C@H]3[C@H]2CC=C4[C@@]3(CC[C@@H](C4)O)C)C | 0 | train |
| 170465557 | CC(C)(C)C(=O)C(N1C=CN=C1)OC2=CC=C(C=C2)Cl | 0 | validation |
| 170465556 | CC1=C(C(=O)N(N1C)C2=CC=CC=C2)I | 0 | test |
| 170465555 | CCOCCOC(=O)C=CC1=CC=C(C=C1)OC | 1 | train |
| 170465554 | CCOC1=CC=C(C=C1)NC(=O)C | 0 | train |
| 170465553 | CCCCCCCC/C=C/C(=O)[O-].CCCCCCCC/C=C/C(=O)[O-].[Ca+2] | 0 | validation |
| 170465552 | C1=CC=C(C(=C1)C(=O)O)N | 0 | test |
| 170465551 | C1=CC=C(C(=C1)C(=O)OCC(CO)O)N | 0 | train |
| 170465550 | C1=CC(=CC=C1NC(=O)NC2=CC(=C(C=C2)Cl)C(F)(F)F)Cl | 0 | train |
| 170465549 | CC1=C(C=C(C=C1)C(=O)C(=O)[O-])C.[Na+] | 0 | validation |
| 170465548 | C([C@@H]([C@H]([C@@H](C(=O)CO)O)O)O)O | 0 | test |
| 170465547 | C1=CC=C(C(=C1)CO)O | 0 | train |

| | | | |
|---|---|---|---|
| 170465546 | CC1=CC(=C(C=C1Cl)C(C)C)O | 0 | train |
| 170465545 | C1=CC=C(C(=C1)C(=O)OC2=CC=CC=C2C(=O)O)O | 0 | validation |
| 170465544 | CCCCCCCCCCCCCCCC(=O)OC(C)C | 0 | test |
| 170465543 | CC(=O)OCC(COC(=O)C)OC(=O)C | 0 | train |
| 170465542 | C1=CC(=CC=C1NC(=O)C2=CC(=CC(=C2O)Br)Br)Br | 0 | train |
| 170465541 | C1(C(C(C(C(C1O)O)O)O)O)O | 0 | validation |
| 170465540 | COC1=CC(=C(C=C1)C(=O)C2=CC=CC=C2O)O | 0 | test |
| 170465539 | CN1CCC2=CC3=C(C(=C2[C@@H]1[C@@H]4C5=C(C(=C(C=C5)OC)OC)C(=O)O4)OC)OCO3 | 0 | train |
| 170465538 | CCN(CC)CCOC(=O)C1=CC=C(C=C1)OCC | 0 | train |
| 170465537 | C1=CC=C(C=C1)OCC(=O)O | 0 | validation |
| 170465536 | C1CCN(CC1)CC(COC(=O)NC2=CC=CC=C2)OC(=O)NC3=CC=CC=C3.Cl | 0 | test |
| 170465535 | CC(C(C1=CC=CC=C1)O)N(C)C/C=C/C2=CC=CC=C2 | 0 | train |
| 170465534 | C1=CC=C(C=C1)C(=O)OC2=CC=CC3=C2N=CC=C3 | 1 | train |
| 170465533 | CCCCCCCCCCC(CCCCCCCC)CO | 0 | validation |
| 170465532 | C1=CC2=C(C(=C1)O)C(=O)C3=C(C2=O)C=CC=C3O | 0 | test |
| 170465531 | CN(C)CCN(CC1=CC=CS1)C2=CC=CC=N2.Cl | 0 | train |
| 170465530 | CC(CC1=CC=CC=C1OC)NC | 0 | train |
| 170465529 | C1=CC(=CC=C1C(C2=CC=C(C=C2)Cl)C(Cl)(Cl)Cl)Cl | 1 | validation |
| 170465528 | CC1CCCCN1CCCOC(=O)C2=CC=C(C=C2)OC3CCCCC3 | 0 | test |
| 170465527 | C=CCCCCCCCCC(=O)O | 0 | train |
| 170465526 | CCCCCCCCCCCCCCCCCCO | 0 | train |
| 170465525 | CC(=O)OC[C@@H]1[C@H]([C@@H]([C@H]([C@H](O1)O[C@]2([C@H]([C@@H]([C@H](O2)CO<br>C(=O)C)OC(=O)C)OC(=O)C)COC(=O)C)OC(=O)C)OC(=O)C)OC(=O)C | 0 | validation |
| 170465524 | CC(=O)O[Hg]C1=CC=CC=C1 | 1 | test |
| 170465523 | C1=CC2=C(C=CC(=C2N=C1)O)Cl | 1 | train |
| 170465522 | CCCCCCCCCCCCCCCCCC(=O)OCC(CO)O | 0 | train |
| 170465521 | CCCC(C(CC)CO)O | 0 | validation |
| 170465520 | CN(C)CCN(CC1=CSC=C1)C2=CC=CC=N2 | 0 | test |
| 170465519 | CN(C)CCN(CC1=CC=C(C=C1)OC)C2=NC=CC=N2.Cl | 0 | train |
| 170465518 | CCN(CC)CCOC(=O)C1(CCCC1)C2=CC=CC=C2.CCN(CC)CCOC(=O)C1(CCCC1)C2=CC=CC=C<br>2.C(CS(=O)(=O)O)S(=O)(=O)O | 0 | train |
| 170465517 | C(C(=O)[O-])SC#N.[Na+] | 0 | validation |
| 170465516 | CCCCC1=CC2=CC=CC=C2C(=N1)OCCN(C)C.Cl | 0 | test |
| 170465515 | C1=C(C=C(C(=C1Cl)O)SC2=CC(=CC(=C2O)Cl)Cl)Cl | 0 | train |
| 170465514 | CC1=C2C(=C(C=C1)[N+](=O)[O-])[Hg]O2 | 1 | train |
| 170465513 | CC(=O)OC1=CC=CC(=C1)O | 0 | validation |
| 170465512 | CC1=C(C(CCC1)(C)C)/C=C/C(=C/C=C/C(=C/COC(=O)C)/C)/C | 0 | test |
| 170465511 | C1=CC=C2C(=C1)C3=CC=CC=C3S2 | 0 | train |
| 170465510 | CC1=CC(=O)N(N1C)C2=CC=CC=C2 | 0 | train |
| 170465509 | C[C@@H]([C@@H](C1=CC=CC=C1)O)NC.Cl | 0 | validation |
| 170465508 | CC1=CC(=CC=C1)N(C)C(=S)OC2=CC3=CC=CC=C3C=C2 | 1 | test |
| 170465507 | C1=CC=C(C=C1)C(C(=O)C2=CC=CC=C2)O | 1 | train |
| 170465506 | COC(=O)C1=CN=CC=C1 | 0 | train |
| 170465505 | CCC(C)C(C(=O)O)N | 0 | validation |
| 170465504 | CC1=CC(=C(C=C1)C(C)C)O | 0 | test |
| 170465503 | CC1=CC(=CC(=C1Cl)C)O | 0 | train |
| 170465502 | C[C@H](CCC(=O)O)[C@H]1CC[C@@H]2[C@@]1(C(=O)C[C@H]3[C@H]2C(=O)C[C@H]4[C@@<br>]3(CCC(=O)C4)C)C | 0 | train |
| 170465501 | C1=CC=C(C=C1)OC(=O)C2=CC=CC=C2O | 0 | validation |
| 170465500 | CCCCCCCCCCCCCCCC[N+]1=CC=CC=C1.[Br-] | 0 | test |
| 170465499 | CCCCC(CC)COC(=O)C1=CC=C(C=C1)N(C)C | 0 | train |
| 170465498 | CCCCC(CC)COC(=O)C1=CC=CC=C1O | 0 | train |
| 170465497 | C[C@@]12CC[C@@H](C1(C)C)CC2=O | 0 | validation |
| 170465496 | C1=CC=C(C(=C1)C(=O)N)O | 0 | test |
| 170465495 | CC1=CC=C(C=C1)O | 0 | train |
| 170465494 | CCCCCCCCCCCC[N+](C)(C)CCOC1=CC=CC=C1.[Br-] | 0 | train |
| 170465493 | CC(=O)NC1=CC=CC=C1 | 0 | validation |
| 170465492 | CC(C)C[C@H](C(=O)O)N | 0 | test |
| 170465491 | C1(C(=O)NC(=N1)O[Al])NC(=O)N.O.O | 0 | train |
| 170465490 | C1=CC=C(C=C1)C2=NC3=C(N2)C=C(C=C3)S(=O)(=O)O | 0 | train |
| 170465489 | COC1=C(C=C(C(=C1)O)C(=O)C2=CC=CC=C2)S(=O)(=O)O | 0 | validation |
| 170465488 | CCN1CCCC1=O | 0 | test |

| | | | |
|---|---|---|---|
| 170465487 | CN1C=NC2=C1C(=O)NC(=O)N2C | 0 | train |
| 170465486 | C1=CC=C(C=C1)O | 0 | train |
| 170465485 | C1=CC=C2C(=C1)C(=CN2)C[C@H](C(=O)O)N | 0 | validation |
| 170465484 | C(C(Cl)(Cl)Cl)(O)O | 0 | test |
| 170465483 | CC1(C2CCC(O1)(CC2)C)C | 0 | train |
| 170465482 | C1=CC=C(C(=C1)C(=O)O)O.C1=CC2=C(C(=C1)O)N=CC=C2 | 0 | train |
| 170465481 | C1=CC(=CC=C1NC(=O)NC2=CC(=C(C=C2)Cl)Cl)Cl | 1 | validation |
| 170465480 | COC(=O)C1=CC=C(C=C1)O | 0 | test |
| 170465479 | CN1C2=C(C(=O)N(C1=O)C)NC(=N2)Br | 0 | train |
| 170465478 | CC1CC(CC(C1)(C)C)OC(=O)C2=CC=CC=C2O | 0 | train |
| 170465477 | CN(C)CCOC1=CC=CC=C1CC2=CC=CC=C2.C(C(=O)O)C(CC(=O)O)(C(=O)O)O | 0 | validation |
| 170465476 | C[C@H]([C@@H](C(=O)O)N)O | 0 | test |
| 170465475 | C1=CC(=C(C=C1Cl)CC2=C(C=CC(=C2)Cl)O)O | 0 | train |
| 170465474 | CCCCCCCCCCCCOS(=O)(=O)[O-].[Na+] | 0 | train |
| 170465473 | CC(C)(C)CC(C)(C)C1=CC=C(C=C1)OCCOCC[N+](C)(C)CC2=CC=CC=C2.[Cl-] | 0 | validation |
| 170465472 | C1=CC(=CC=C1CCO[C@H]2[C@@H]([C@H]([C@@H]([C@H](O2)CO)O)O)O)O | 0 | test |
| 170465471 | CCCCCCC1=C(C=C(C=C1)O)O | 0 | train |
| 170465470 | CC1=CC(=CC(=C1CC2=NCCN2)C)C(C)(C)C.Cl | 0 | train |
| 170465469 | CC[Hg]SC1=CC=CC=C1C(=O)[O-].[Na+] | 1 | validation |
| 170465468 | C1N2CN3CN1CN(C2)C3 | 0 | test |
| 170465467 | COC(=O)[C@H]1[C@H](CC[C@@H]2[C@@H]1C[C@H]3C4=C(CCN3C2)C5=CC=CC=C5N4)O | 0 | train |
| 170465466 | CC1=CC(=C(C=C1C2=CC(=C(C=C2C)O)I)C(C)C)C(C)C)OI | 1 | train |
| 170465465 | CCCCCCCCCCCCCCCC[N+](C)(C)CC1=CC=CC=C1.[Cl-] | 0 | validation |
| 170465464 | COC1=C(C=CC(=C1)CC=C)O | 0 | test |
| 170465463 | CCOC(=O)C1=CC=C(C=C1)N | 0 | train |
| 170465462 | C1=CC(=CC(=C1)O)O | 0 | train |
| 170465461 | CN(C)C1=CC2=C(C=C1)N=C3C=CC(=[N+](C)C)C=C3S2.[Cl-] | 1 | validation |
| 170465460 | C1(C(=O)NC(=O)N1)NC(=O)N | 0 | test |
| 170465459 | CCCCN(CCCC)CCCOC(=O)C1=CC=C(C=C1)N | 1 | train |
| 170465458 | CC(C)(C(Cl)(Cl)Cl)O | 0 | train |
| 170465457 | C([C@@H](C(=O)O)N)SSC[C@@H](C(=O)O)N | 0 | validation |
| 170465456 | CCCC(=O)O[C@@]1(CC[C@@H]2[C@@]1(C[C@@H]([C@H]3[C@H]2CCC4=CC(=O)CC[C@]34C)O)C)C(=O)CO | 0 | test |
| 170465455 | CC1=C(C2=CC=CC=C2C(=C1)O)O | 1 | train |
| 170465454 | C1=CC(=CC=C1N)S(=O)(=O)NC2=NC=CS2 | 0 | train |
| 170465453 | CN(C)CCC[C@@]1(C2=C(CO1)C=C(C=C2)C#N)C3=CC=C(C=C3)F.C(=O)(C(=O)O)O | 0 | validation |
| 170465452 | CCCCN1CCCC[C@H]1C(=O)NC2=C(C=CC=C2C)C.Cl | 0 | test |
| 170465451 | C[C@H]1[C@H]([C@H](C[C@@H](O1)O[C@H]2C[C@@](CC3=C(C4=C(C(=C23)O)C(=O)C5=C(C4=O)C=CC=C5OC)O)(C(=O)CO)O)N)O.Cl | 0 | train |
| 170465450 | C1=C(C=C(C(=C1I)OC2=CC(=C(C(=C2)I)O)I)I)C[C@@H](C(=O)O)N | 0 | train |
| 170465449 | CC(C1=CC=CC(=C1)C(=O)C2=CC=CC=C2)C(=O)O | 0 | validation |
| 170465448 | CC(C)NCC(COC1=CC=C(C=C1)CCOCC2CC2)O | 0 | test |
| 170465447 | C1=NC2=C(C(=N1)N)N=CN2[C@H]3[C@@H]([C@@H]([C@H](O3)CO)O)O | 0 | train |
| 170465446 | CCCC(CCC)C(=O)O | 0 | train |
| 170465445 | CN1CCN(CC1)C(=O)O[C@H]2C3=NC=CN=C3C(=O)N2C4=NC=C(C=C4)Cl | 0 | validation |
| 170465444 | CC1=C2C(=C(C(=C1C)OC(=O)C)C)CC[C@@](O2)(C)CCC[C@H](C)CCC[C@H](C)CCCC(C)C | 0 | test |
| 170465443 | CC1=CN=C(C(=C1OC)C)CS(=O)C2=NC3=C(N2)C=C(C=C3)OC | 0 | train |
| 170465442 | C1CN(CCN1CCOCC(=O)O)C(C2=CC=CC=C2)C3=CC=C(C=C3)Cl.Cl.Cl | 0 | train |
| 170465441 | CC(=O)OC1=CC2=C(S1)CCN(C2)C(C3=CC=CC=C3F)C(=O)C4CC4.Cl | 0 | validation |
| 170465440 | COC1=CC=CC=C1OCCNCC(COC2=CC=CC3=C2C4=CC=CC=C4N3)O.C(C(C(=O)O)O)(C(=O)O)O | 0 | test |
| 170465439 | CC(=O)NO | 0 | train |
| 170465438 | C(=N)(N)N.C(=N)(N)N.C(=O)(O)O | 0 | train |
| 170465437 | C1=CC=C2C(=C1)C(=NO2)CS(=O)(=O)N | 0 | validation |
| 170465436 | CCCC(C)(COC(=O)N)COC(=O)NC(C)C | 0 | test |
| 170465435 | CC1=C(C(=CC=C1)C)NC(=O)CN2CCN(CC2)CC(COC3=CC=CC=C3OC)O | 0 | train |
| 170465434 | COCCOC1=C(C=C2C(=C1)C(=NC=N2)NC3=CC=CC(=C3)C#C)OCCOC | 1 | train |
| 170465433 | CC[C@@H]1[C@@]([C@@H]([C@H](C(=O)[C@@H](C[C@@]([C@@H]([C@H]([C@@H]([C@H](C(=O)O1)C)O[C@H]2C[C@@]([C@H]([C@@H](O2)C)O)(C)OC)C)O[C@H]3[C@@H]([C@H](C[C@H](O3)C)N(C)C)OC(=O)CC)(C)O)C)C)O)(C)O | 0 | validation |

| | | | |
|---|---|---|---|
| 170465432 | C1=C(C(=C(C(=C1I)NC(=O)CCOCCOCCOCCOCCC(=O)NC2=C(C=C(C(=C2I)C(=O)O)I)I)I)C(=O)O)I | 0 | test |
| 170465431 | CC1=CC=CN2C1=NC=C(C2=O)C3=NN=N[N-]3.[K+] | 0 | train |
| 170465430 | C[C@]12C[C@@H]([C@H]3[C@H]([C@@H]1CC[C@@]2(C(=O)COC(=O)CC(C)(C)C)O)CCC4=CC(=O)C=C[C@]34C)O | 0 | train |
| 170465429 | C1=CC(=CC=C1[C@H]([C@@H](COC(=O)CCC(=O)[O-])NC(=O)C(Cl)Cl)O)[N+](=O)[O-].[Na+] | 0 | validation |
| 170465428 | COC1=CN=C(N=C1)NS(=O)(=O)C2=CC=C(C=C2)N | 0 | test |
| 170465427 | C[N+]1(CCC(C1)OC(=O)C(C2CCCC2)(C3=CC=CC=C3)O)C.[Br-] | 0 | train |
| 170465426 | CCC(=C)C(=O)C1=C(C(=C(C=C1)OCC(=O)O)Cl)Cl | 1 | train |
| 170465425 | CC/C(=C(¥C1=CC=CC=C1)/C2=CC=C(C=C2)OCCN(C)C)/C3=CC=CC=C3.C(C(=O)O)C(CC(=O)O)(C(=O)O)O | 0 | validation |
| 170465424 | CNCCCN1C2=CC=CC=C2CCC3=CC=CC=C31.Cl | 0 | test |
| 170465423 | CNS(=O)(=O)CCC1=CC2=C(C=C1)NC=C2C3CCN(CC3)C.Cl | 0 | train |
| 170465422 | C1=CC(=CC=C1COC(CN2C=CN=C2)C3=C(C=C(C=C3)Cl)Cl)Cl.[N+](=O)(O)[O-] | 0 | train |
| 170465421 | C1=CC=C(C=C1)C2C(=O)N=C(O2)N | 0 | validation |
| 170465420 | C1CCC2=NC3=CC=CC=C3C(=C2C1)N | 0 | test |
| 170465419 | C1CN=C(N1)NC2=C(C=CC3=NSN=C32)Cl | 0 | train |
| 170465418 | CN(CCC1=CC=C(C=C1)NS(=O)(=O)C)CCOC2=CC=C(C=C2)NS(=O)(=O)C | 0 | train |
| 170465417 | CN(CCOC1=CC=C(C=C1)CC2C(=O)NC(=O)S2)C3=CC=CC=N3 | 0 | validation |
| 170465416 | CC1=C(N=CN1)CN2CCC3=C(C2=O)C4=CC=CC=C4N3C.Cl | 0 | test |
| 170465415 | COC1=C(C=C2C(=C1)C(=NC(=N2)N3CCN(CC3)C(=O)C4=CC=CO4)N)OC | 0 | train |
| 170465414 | CCN1C2=CC3=C(C=C2C(=O)C(=N1)C(=O)O)OCO3 | 0 | train |
| 170465413 | CN(C)CC/C=C/1¥C2=CC=CC=C2COC3=CC=CC=C31.Cl | 0 | validation |
| 170465412 | CC(C1=CC=C(C=C1)C(=O)C2=CC=CS2)C(=O)O | 0 | test |
| 170465411 | CC(=O)C1=CC=C(C=C1)S(=O)(=O)NC(=O)NC2CCCCC2 | 0 | train |
| 170465410 | CS(=O)(=O)O.C#CCN[C@@H]1CCC2=CC=CC=C12 | 0 | train |
| 170465409 | CC(C)N1C2=CC=CC=C2C(=C1/C=C/[C@H](C[C@H](CC(=O)[O-])O)O)C3=CC=C(C=C3)F.[Na+] | 0 | validation |
| 170465408 | CCC(C)C1(C(=O)NC(=O)NC1=O)CC=C | 0 | test |
| 170465407 | C1=NC(=O)NC(=C1F)N | 0 | train |
| 170465406 | CC1=C[C@@H]2[C@H](CC[C@]3([C@H]2CC[C@@]3(C(=O)C)OC(=O)C)C)[C@@]4(C1=CC(=O)CC4)C | 0 | train |
| 170465405 | CO[C@H]1[C@@H](C[C@@H]2CN3CCC4=C([C@H]3C[C@@H]2[C@@H]1C(=O)OC)NC5=C4C=CC(=C5)OC)OC(=O)C6=CC(=C(C(=C6)OC)OC)OC | 0 | validation |
| 170465404 | CC1=C(C(=NO1)C2=CC=CC=C2Cl)C(=O)N[C@H]3[C@@H]4N(C3=O)[C@H](C(S4)(C)C)C(=O)[O-].[Na+] | 0 | test |
| 170465403 | C[C@]12CC[C@H]3[C@H]([C@@H]1CC[C@]2(C)O)CC[C@@H]4[C@@]3(C/C(=C/O)/C(=O)C4)C | 0 | train |
| 170465402 | CC(C)NC(=O)C1=CC=C(C=C1)CNNC.Cl | 0 | train |
| 170465401 | C[C@H]1C(=O)N[C@H](C(=O)N/C(=C¥NC(=O)N)/C(=O)N[C@H](C(=O)NC[C@@H](C(=O)N1)N)[C@@H]2CCN=C(N2)N)CNC(=O)C[C@H](CCCN)N | 0 | validation |
| 170465400 | CCCOC1=C(C=C(C=C1)C(=O)OCCN(CC)CC)N.Cl | 0 | test |
| 170465399 | CC1=C(C=C(C=C1)N2C(=O)C(=C(N2)C)N/N=C¥3/C=CC=C(C3=O)C4=CC(=CC=C4)C(=O)O)C | 0 | train |
| 170465398 | CC[N+](CC)(CC)CCOC1=C(C(=CC=C1)OCC[N+](CC)(CC)CC)OCC[N+](CC)(CC)CC.[I-].[I-].[I-] | 0 | train |
| 170465397 | C1=CC=C(C=C1)CCCCOCCCCCCNCC(C2=CC(=C(C=C2)O)CO)O.C1=CC=C2C(=C1)C=CC(=C2O)C(=O)O | 0 | validation |
| 170465396 | CC(C)(C)OC(=O)NCCC(=O)N[C@@H](CC1=CNC2=CC=CC=C21)C(=O)N[C@@H](CCSC)C(=O)N[C@@H](CC(=O)O)C(=O)N[C@@H](CC3=CC=CC=C3)C(=O)N | 0 | test |
| 170465395 | COC1=C(C=C2C(=C1)C(=NC(=N2)N3CCN(CC3)C(=O)C4COC5=CC=CC=C5O4)N)OC.CS(=O)(=O)O | 0 | train |
| 170465394 | COC1=CC=C(C=C1)C(=C(C2=CC=C(C=C2)OC)Cl)C3=CC=C(C=C3)OC | 0 | train |
| 170465393 | CC1=C(C=C(C=C1)NC(=O)C2=CC=C(C=C2)CN3CCN(CC3)C)NC4=NC=CC(=N4)C5=CN=CC=C5.CS(=O)(=O)O | 1 | validation |
| 170465392 | C1CC(=O)NC2=C1C=CC(=C2)OCCCCN3CCN(CC3)C4=C(C(=CC=C4)Cl)Cl | 0 | test |
| 170465391 | CCCCNC1=CC=C(C=C1)C(=O)OCCOCCOCCOCCOCCOCCOCCOCCOCCOC | 0 | train |
| 170465390 | CC1=C(C(=CC=C1)Cl)NC(=O)C2=CN=C(S2)NC3=NC(=NC(=C3)N4CCN(CC4)CCO)C | 0 | train |
| 170465389 | C[C@]12CCC(=O)C=C1CC[C@@H]3[C@@]2([C@H](C[C@]4([C@H]3C[C@@H]5[C@]4(OC(O5)(C)C)C(=O)CCl)C)O)F | 0 | validation |

| | | | |
|---|---|---|---|
| 170465388 | CCCCN(CCCC)CC(C1=C2C3=C(C=C(C=C3)Cl)/C(=C/C4=CC=C(C=C4)Cl)/C2=CC(=C1)Cl)O | 1 | test |
| 170465387 | CN1C(=NC(=O)C(=N1)[O-])SCC2=C(N3[C@@H]([C@@H](C3=O)NC(=O)/C(=N¥OC)/C4=CSC(=N4)N)SC2)C(=O)[O-].O.O.O.[Na+].[Na+] | 0 | train |
| 170465386 | CC[N+](CC)(CCNC(=O)C(=O)NCC[N+](CC)(CC)CC1=CC=CC=C1Cl)CC2=CC=CC=C2Cl.[Cl-].[Cl-] | 0 | train |
| 170465385 | CCC(=O)O[C@@]1([C@H](C[C@@H]2[C@@]1(C[C@@H]([C@]3([C@H]2CCC4=CC(=O)C=C[C@@]43C)F)O)C)C)C(=O)CCl | 0 | validation |
| 170465384 | CC(=O)CCCCN1C(=O)C2=C(N=CN2C)N(C1=O)C | 0 | test |
| 170465383 | CC[C@@H](CO)NC(=O)[C@H]1CN([C@@H]2CC3=CNC4=CC=CC(=C34)C2=C1)C | 0 | train |
| 170465382 | C1=CC=C(C=C1)COC(=O)C2=CC=CC=C2 | 0 | train |
| 170465381 | C1=CC(=C(C(=C1)Cl)CC(=O)N=C(N)N)Cl.Cl | 0 | validation |
| 170465380 | CCN1CCN(C(=O)C1=O)C(=O)N[C@H](C2=CC=CC=C2)C(=O)N[C@H]3[C@@H]4N(C3=O)[C@@H](C(S4)(C)C)C(=O)[O-].[Na+] | 0 | test |
| 170465379 | COC1=CC=C(C=C1)C2C(=O)C3=CC=CC=C3C2=O | 1 | train |
| 170465378 | CN1CCC(=C2C3=C(C(=O)CC4=CC=CC=C42)SC=C3)CC1 | 0 | train |
| 170465377 | CNS(=O)(=O)CC1=CC2=C(C=C1)NC=C2CCN(C)C | 0 | validation |
| 170465376 | C1CN(CCN1)C2=NC3=CC=CC=C3OC4=C2C=C(C=C4)Cl | 1 | test |
| 170465375 | C1CN(CCN1CCCN2C3=CC=CC=C3SC4=C2C=C(C=C4)C(F)(F)F)CCO | 1 | train |
| 170465374 | CNCCC(C1=CC=CC=C1)OC2=CC=C(C=C2)C(F)(F)F | 1 | train |
| 170465373 | CC(C)[C@@H](C(=O)OCCOCN1C=NC2=C1NC(=NC2=O)N)N.Cl | 0 | validation |
| 170465372 | CCOC(=O)/C=C(¥C)/C=C/C=C(¥C)/C=C/C1=C(C(=C(C=C1C)OC)C)C | 0 | test |
| 170465371 | CN(C)CCCN1C2=CC=CC=C2SC3=C1C=C(C=C3)Cl | 1 | train |
| 170465370 | C[C@@H]1CN(C[C@@H](N1)C)C2=C(C3=C(C(=C2F)N)C(=O)C(=CN3C4CC4)C(=O)O)F | 0 | train |
| 170465369 | CC1=NN=C(S1)NS(=O)(=O)C2=CC=C(C=C2)N | 0 | validation |
| 170465368 | CC[C@@]1(C(=O)N(C(=O)N1)C)C2=CC=CC=C2 | 0 | test |
| 170465367 | C1=CN=CC=C1C2=CNC(=O)C(=C2)N | 0 | train |
| 170465366 | CC1CC2=CC=CC=C2N1NC(=O)C3=CC(=C(C=C3)Cl)S(=O)(=O)N | 0 | train |
| 170465365 | CC(C)C1=CC2=C(C=C1)OC3=NC(=C(C=C3C2=O)C(=O)O)N | 1 | validation |
| 170465364 | C1=CC(=C(C(=C1)Cl)C=NN=C(N)N)Cl | 0 | test |
| 170465363 | CC(=O)C1=CC2=C(C=C1)SC3=CC=CC=C3N2CCCN4CCC(CC4)CCO | 0 | train |
| 170465362 | C/C=C(/C(=C/C)/C1=CC=C(C=C1)O)¥C2=CC=C(C=C2)O | 0 | train |
| 170465361 | C1[C@H]([C@@H]1N)C2=CC=CC=C2.Cl | 0 | validation |
| 170465360 | CC1=C(C=CC2=C1OC(=O)C(=C2[O-])NC(=O)C3=CC(=C(C=C3)O)CC=C(C)C)O[C@H]4[C@@H]([C@@H]([C@H](C(O4)(C)C)OC)OC(=O)N)O.[Na+] | 0 | test |
| 170465359 | CNC[C@@H]([C@H]([C@@H]([C@@H](CO)O)O)O)O | 0 | train |
| 170465358 | C(CCCC(=O)O)CCCC(=O)O | 0 | train |
| 170465357 | C[C@]12CC[C@H]3[C@H]([C@@H]1CC[C@]2(C#C)O)CCC4=C3C=CC(=C4)OC | 1 | validation |
| 170465356 | CNC[C@@H](C1=CC(=CC=C1)O)O.Cl | 0 | test |
| 170465355 | CC1=C(N2[C@@H]([C@@H](C2=O)NC(=O)[C@@H](C3=CCC=CC3)N)SC1)C(=O)O | 0 | train |
| 170465354 | CC1([C@@H](N2[C@H](S1)[C@@H](C2=O)N=CN3CCCCCC3)C(=O)O)C | 0 | train |
| 170465353 | CN1C(=NN=N1)SCC2=C(N3[C@@H]([C@@](C3=O)(NC(=O)C(C4=CC=C(C=C4)O)C(=O)[O-])OC)OC2)C(=O)[O-].[Na+].[Na+] | 0 | validation |
| 170465352 | C(CCC(=O)O)CC(=O)O | 0 | test |
| 170465351 | CC1=C(C=CC=C1O)C(=O)N[C@@H](CSC2=CC=CC=C2)[C@@H](CN3C[C@H]4CCCC[C@H]4C[C@H]3C(=O)NC(C)(C)C)O.CS(=O)(=O)O | 1 | train |
| 170465350 | CC[N+](C)(CC)CCOC(=O)C1C2=CC=CC=C2OC3=CC=CC=C13.[Br-] | 0 | train |
| 170465349 | C[N+](C)(C)CCCCCCCCCC[N+](C)(C)C.[Br-].[Br-] | 0 | validation |
| 170465348 | CCCCCCCCCCCCCCCCO | 0 | test |
| 170465347 | C1CCC(CC1)N2C(=NN=N2)CCCCOC3=CC4=C(C=C3)NC(=O)CC4 | 0 | train |
| 170465346 | C1=C(C=C(C(=C1NC(=O)C(=O)O)Cl)NC(=O)C(=O)O)C#N | 0 | train |
| 170465345 | C1[C@@H]([C@H]([C@@H]([C@H]([C@@H]1NC(=O)[C@H](CCN)O)O[C@@H]2[C@@H]([C@H]([C@@H]([C@H](O2)CO)O)N)O)O)O[C@@H]3[C@@H]([C@H]([C@@H]([C@H](O3)CN)O)O)O)N | 0 | validation |
| 170465344 | C1=CC(=CC=C1CSC(CN2C=CN=C2)C3=C(C=C(C=C3)Cl)Cl)Cl.[N+](=O)(O)[O-] | 1 | test |
| 170465343 | C1=CC(=C(C=C1Cl)Cl)C(CN2C=CN=C2)OCC3=C(SC=C3)Cl | 1 | train |
| 170465342 | C1CN(CCN1CCC2=C(C=C3C(=C2)CC(=O)N3)Cl)C4=NSC5=CC=CC=C54 | 0 | train |
| 170465341 | C1CCN(CC1)CCOC2=CC=C(C=C2)C(=O)C3=C(SC4=C3C=CC(=C4)O)C5=CC=C(C=C5)O.Cl | 0 | validation |
| 170465340 | CN1C2=C(C=C(C=C2)N(CCCl)CCCl)N=C1CCCC(=O)O | 0 | test |
| 170465339 | CC1=CC(=O)OC2=C1C=C3C=C(OC3=C2C)C | 1 | train |

| | | | |
|---|---|---|---|
| 170465338 | C[C@H](CN1C=NC2=C1N=CN=C2N)OCP(=O)(OCOC(=O)OC(C)C)OCOC(=O)OC(C)C.C(=CC(=O)O)C(=O)O | 0 | train |
| 170465337 | CC1=CC(=C(C=C1)O)[C@H](CCN(C(C)C)C(C)C)C2=CC=CC=C2 | 0 | validation |
| 170465336 | C[C@H](C1=CC=CC2=CC=CC=C21)NCCCC3=CC(=CC=C3)C(F)(F)F.Cl | 1 | test |
| 170465335 | CCCCCCCCCCCCCCCC[N+](C)(C)CCN(CC1=CC=C(C=C1)OC)C2=NC=CC=N2.[Br-] | 0 | train |
| 170465334 | CC(=O)OCC(=O)[C@H]1CC[C@@H]2[C@@]1(CC[C@H]3[C@H]2CCC4=CC(=O)CC[C@]34C)C | 0 | train |
| 170465333 | CO/N=C/1¥CN(CC1CN)C2=C(C=C3C(=O)C(=CN(C3=N2)C4CC4)C(=O)O)F.CS(=O)(=O)O | 0 | validation |
| 170465332 | CCOC(=O)C1(CCN(CC1)CCC(C#N)(C2=CC=CC=C2)C3=CC=CC=C3)C4=CC=CC=C4.Cl | 1 | test |
| 170465331 | C[C@]12C[C@@H]([C@H]3[C@H]([C@@H]1C[C@@H]4[C@]2(OC(O4)(C)C)C(=O)CO)C[C@@H](C5=CC(=O)C=C[C@]35C)F)O | 0 | train |
| 170465330 | CCC1=C[C@H]2C[C@@](C3=C(CN(C2)C1)C4=CC=CC=C4N3)(C5=C(C=C6C(=C5)[C@]78CCN9[C@H]7[C@@](C=CC9)([C@H]([C@@]([C@@H]8N6C)(C(=O)OC)O)OC(=O)C)CC)OC)C(=O)O C.C(C(C(=O)O)O)(C(=O)O)O.C(C(C(=O)O)O)(C(=O)O)O | 1 | train |
| 170465329 | CCOC(=O)C(C)(C)OC1=CC=C(C=C1)Cl | 0 | validation |
| 170465328 | CC1=NS(=O)(=O)C2=C(N1)C=CC(=C2)Cl | 0 | test |
| 170465327 | C([C@@H]1[C@H]([C@@H]([C@H](C(=O)O1)O)O)O)O | 0 | train |
| 170465326 | C1[C@@H]([C@H]([C@@H]([C@H](N1CCO)CO)O)O)O | 0 | train |
| 170465325 | CN(C)CCCN1C2=CC=CC=C2SC3=C1C=C(C=C3)C(F)(F)F.Cl | 0 | validation |
| 170465324 | C1CCN(CC1)CCC(C2CC3CC2C=C3)(C4=CC=CC=C4)O.Cl | 0 | test |
| 170465323 | C1CC(=O)NC(=O)C1N2C(=O)C3=CC=CC=C3C2=O | 0 | train |
| 170465322 | C1=CC=C(C=C1)CC(=O)NC(=O)N | 0 | train |
| 170465321 | C1CNCC(C2=CC(=C(C(=C21)Cl)O)O)C3=CC=C(C=C3)O | 0 | validation |
| 170465320 | CC1=C(C(C(=C(N1)C)C(=O)OCC(C)C)C2=CC=CC=C2[N+](=O)[O-])C(=O)OC | 1 | test |
| 170465319 | CNCC[C@@H](C1=CC=CS1)OC2=CC=CC3=CC=CC=C32.Cl | 1 | train |
| 170465318 | C[C@@](C1=CC=CC=C1)(C2=CC=C(C=C2)Cl)OCC[C@H]3CCCN3C.C(=C/C(=O)O)¥C(=O)O | 0 | train |
| 170465317 | CN1CCC[C@@H]1CC2=CNC3=C2C=C(C=C3)CCS(=O)(=O)C4=CC=CC=C4 | 0 | validation |
| 170465316 | CN1C2=C(C(=O)N(C1=O)C)N(C=N2)CC(CO)O | 0 | test |
| 170465315 | CN1C(=O)CC(C1=O)C2=CC=CC=C2 | 0 | train |
| 170465314 | CCN(CC)CC(=O)OCC(=O)[C@]1(CC[C@@H]2[C@@]1(C[C@@H]([C@H]3[C@H]2CCC4=CC(=O)CC[C@]34C)O)C)O | 0 | train |
| 170465313 | CCN1CC(C(C1=O)(C2=CC=CC=C2)C3=CC=CC=C3)CCN4CCOCC4.Cl | 0 | validation |
| 170465312 | CCOC(=O)[C@H](CCC1=CC=CC=C1)N[C@@H](C)C(=O)N2CC3=CC=CC=C3C[C@H]2C(=O)O.Cl | 0 | test |
| 170465311 | CCOC(=O)CC(C(=O)OCC)SP(=S)(OC)OC | 1 | train |
| 170465310 | CCCCC1C(=O)N(N(C1=O)C2=CC=C(C=C2)O)C3=CC=CC=C3 | 1 | train |
| 170465309 | CCCC(=O)NC1=CC(=C(C=C1)OCC(CNC(C)C)O)C(=O)C.Cl | 0 | validation |
| 170465308 | CC1=CC2=C(C=C1C(=C)C3=CC=C(C=C3)C(=O)O)C(CCC2(C)C)(C)C | 0 | test |
| 170465307 | CC(C)(CC1=CC=C(C=C1)Cl)N.Cl | 0 | train |
| 170465306 | CC(=O)OCC1=C(N2[C@@H]([C@@H](C2=O)NC(=O)CSC3=CC=NC=C3)SC1)C(=O)[O-].[Na+] | 0 | train |
| 170465305 | C1=CC(=CC=C1C(=O)NCCC(=O)O)N/N=C¥2/C=CC(=O)C(=C2)C(=O)O | 0 | validation |
| 170465304 | C(CC(=O)N)[C@H](C(=O)O)N | 0 | test |
| 170465303 | C1=C2C(=CC(=C1Cl)S(=O)(=O)N)S(=O)(=O)N=CN2 | 0 | train |
| 170465302 | C1=CC=C(C(=C1)C(=O)NCC(=O)O)I | 0 | train |
| 170465301 | C[C@@H](C1=CC2=C(C=C1)C=C(C=C2)OC)C(=O)[O-].[Na+] | 0 | validation |
| 170465300 | C1C[C@@H]2CN(C(=O)C3=C2C(=CC=C3)C1)[C@@H]4CN5CCC4CC5.Cl | 0 | test |
| 170465299 | C[C@@H](C1=CC(=CC=C1)OC2=CC=CC=C2)C(=O)[O-].C[C@@H](C1=CC(=CC=C1)OC2=CC=CC=C2)C(=O)[O-].O.O.[Ca+2] | 0 | train |
| 170465298 | C[C@]12CCC(=O)C=C1[C@H](C[C@@H]3[C@@H]2[C@H](C[C@]4([C@H]3C[C@@H]5[C@]4(OC(O5)(C)C)C(=O)CO)C)O)F | 0 | train |
| 170465297 | CN1CCC2=CC=CC3=C2[C@H]1CC4=C3C(=C(C=C4)O)O | 1 | validation |
| 170465296 | CC1=C(C(=NO1)C2=C(C=CC=C2Cl)Cl)C(=O)N[C@H]3[C@@H]4N(C3=O)[C@H](C(S4)(C)C)C(=O)[O-].O.[Na+] | 0 | test |
| 170465295 | CC(=O)O[C@H]1C[C@@H]2CC[C@@H]3[C@@H]([C@]2(C[C@@H]1N4CCCCC4)C)CC[C@]5([C@H]3C[C@@H]([C@@H]5OC(=O)C)[N+]6(CCCCC6)C)C.[Br-] | 0 | train |
| 170465294 | C[C@H]1C[C@H]2[C@@H]3C[C@@H](C4=CC(=O)C=C[C@@]4([C@]3([C@H](C[C@@]2([C@]1(C(=O)COC(=O)C)OC(=O)C)C)O)F)C)F | 0 | train |
| 170465293 | C1CSC2=N[C@H](CN21)C3=CC=CC=C3.Cl | 0 | validation |
| 170465292 | CN(C)CCOC(C1=CC=CC=C1)C2=CC=C(C=C2)Br | 0 | test |
| 170465291 | CC(C)[N+](C)(CCC(C1=CC=CC=C1)(C2=CC=CC=C2)C(=O)N)C(C)C | 0 | train |

| | | | |
|---|---|---|---|
| 170465290 | CC(C)(C)[C@@H](C(=O)N[C@@H](CC1=CC=CC=C1)[C@H](CN(CC2=CC=C(C=C2)C3=CC=CC=N3)NC(=O)[C@H](C(C)(C)C)NC(=O)OC)O)NC(=O)OC | 0 | train |
| 170465289 | CCN(CC)CCNC(=O)C1C(=C(N=C1C)/C=C¥2/C3=C(C=CC(=C3)F)NC2=O)C.C(C(C(=O)O)O)C(=O)O | 1 | validation |
| 170465288 | CN(C)CCC1=CNC2=C1C=C(C=C2)CS(=O)(=O)N3CCCC3 | 0 | test |
| 170465287 | CN1CCCC(C1)CC2C3=CC=CC=C3SC4=CC=CC=C24.Cl | 1 | train |
| 170465286 | CCN1C=CC(=NC1=O)NS(=O)(=O)C2=CC=C(C=C2)N | 0 | train |
| 170465285 | CCCC1=NN(C2=C1NC(=NC2=O)C3=C(C=CC(=C3)S(=O)(=O)N4CCN(CC4)C)OCC)C.C(C(=O)O)C(CC(=O)O)(C(=O)O)O | 0 | validation |
| 170465284 | C1C2C3=CC=CC=C3CC4=CC=CC=C4N2C(=N1)N.Cl | 0 | test |
| 170465283 | C[C@]12CCC(=O)C=C1C[C@H]([C@@H]3[C@]24[C@H](O4)C[C@]5([C@H]3CC[C@@]56CCC(=O)O6)C)C(=O)OC | 0 | train |
| 170465282 | CC[N+](C)(CC)CCOC(=O)C(C1CCCCC1)(C2=CC=CC=C2)O.[Br-] | 0 | train |
| 170465281 | C=C1CC[C@]2([C@H]3CC4=C5[C@]2([C@H]1OC5=C(C=C4)O)CCN3CC6CC6)O | 0 | validation |
| 170465280 | C1CCN(CC1)CCCC(C2=CC=CC=C2)(C3=CC=CC=C3)O | 0 | test |
| 170465279 | CC1=CC(=CC=C1)NC2=C(C=NC=C2)S(=O)(=O)NC(=O)NC(C)C | 0 | train |
| 170465278 | C[C@H](CN1C2=CC=CC=C2SC3=CC=CC=C31)CN(C)C.C[C@H](CN1C2=CC=CC=C2SC3=CC=CC=C31)CN(C)C.[C@@H]([C@@H](C(=O)O)O)(C(=O)O)O | 0 | train |
| 170465277 | CC(C)NCC(COC1=CC=C(C=C1)CCC(=O)OC)O.Cl | 0 | validation |
| 170465276 | CN1CCCN=C1COC(=O)C(C2CCCCC2)(C3=CC=CC=C3)O.Cl | 0 | test |
| 170465275 | C1=CC=C2C(=C1)C(=C(C(=O)O2)CC3=C(C4=CC=CC=C4OC3=O)O)O | 1 | train |
| 170465274 | C1C2CC(C1C=C2)C3NC4=CC(=C(C=C4S(=O)(=O)N3)S(=O)(=O)N)Cl | 0 | train |
| 170465273 | C1=CC=C(C=C1)OC(=O)C2=C(C=C(C=C2)N)O | 1 | validation |
| 170465272 | CN1CCCCC1CCN2C3=CC=CC=C3SC4=C2C=C(C=C4)S(=O)C | 0 | test |
| 170465271 | CC(C)(CC1=CC=CC=C1)NC.CC(C)(CC1=CC=CC=C1)NC.OS(=O)(=O)O | 0 | train |
| 170465270 | CN1CCN(CC1)C(C2=CC=CC=C2)C3=CC=CC=C3.Cl.Cl | 0 | train |
| 170465269 | C1C2=C(C=CC(=C2Cl)Cl)NC3=NC(=CN31)O.Cl | 0 | validation |
| 170465268 | CC(C)(C1=CN=CC=C1)C(=O)C2=CN=CC=C2 | 0 | test |
| 170465267 | C1=CC=C(C=C1)N=NC2=C(N=C(C=C2)N)N.Cl | 1 | train |
| 170465266 | CCOC(=O)C1(CCN(CC1)CCC2=CC=C(C=C2)N)C3=CC=CC=C3 | 0 | train |
| 170465265 | CN1CCN(CC1)CCCN2C3=CC=CC=C3SC4=C2C=C(C=C4)C(F)(F)F.Cl.Cl | 1 | validation |
| 170465264 | CCCN[C@H]1CCC2=C(C1)SC(=N2)N | 0 | test |
| 170465263 | CCN1C=C(C(=O)C2=CC(=C(N=C21)N3CCNCC3)F)C(=O)O | 0 | train |
| 170465262 | CCN1C=C(C(=O)C2=CC(=C(C(=C21)F)N3CCNC(C3)C)F)C(=O)O.Cl | 0 | train |
| 170465261 | CC(C(C)(C)C)N=C(NC#N)NC1=CC=NC=C1.O | 0 | validation |
| 170465260 | CC(CN1C2=CC=CC=C2CCC3=CC=CC=C31)CN(C)C.C(=C¥C(=O)O)¥C(=O)O | 0 | test |
| 170465259 | CCC1(CCC(=O)NC1=O)C2=CC=C(C=C2)N | 0 | train |
| 170465258 | CC(=O)OC1=CC2=C(C=C1)[C@H]3CC[C@]4([C@H]([C@@H]3CC2)CC[C@@H]4O)C | 1 | train |
| 170465257 | C(CNCCNCCN)N | 0 | validation |
| 170465256 | C[C@@H]([C@@H](C1=CC(=CC=C1)O)O)N.[C@@H]([C@@H](C(=O)O)O)(C(=O)O)O | 0 | test |
| 170465255 | [C@H]([C@@H](C(=O)O)S)(C(=O)O)S | 0 | train |
| 170465254 | CC1=NN=C(S1)SCC2=C(N3[C@@H]([C@@H](C3=O)NC(=O)CN4C=NN=N4)SC2)C(=O)O | 0 | train |
| 170465253 | CCOC1=C(C2=CC=CC=C2C=C1)C(=O)N[C@H]3[C@@H]4N(C3=O)[C@H](C(S4)(C)C)C(=O)[O-].O.[Na+] | 0 | validation |
| 170465252 | CCC[C@@H]1C[C@H](N(C1)C)C(=O)N[C@@H]([C@@H]2[C@@H]([C@@H]([C@H]([C@H](O2)SC)O)O)O)[C@@H](C)O.Cl | 0 | test |
| 170465251 | C[C@]12CC[C@H]3[C@H]([C@@H]1CC[C@]2(C)O)CC[C@@H]4[C@@]3(CC5=C(C4)NN=C5)C | 0 | train |
| 170465250 | CCCN1C[C@@H](C[C@H]2[C@H]1CC3=CNC4=CC=CC2=C34)CSC.CS(=O)(=O)O | 0 | train |
| 170465249 | C1[C@H](C(=O)NO1)N | 0 | validation |
| 170465248 | CC1([C@@H](N2[C@H](S1(=O)=O)CC2=O)C(=O)O)C | 0 | test |
| 170465247 | C1[C@@H]([C@H](O[C@H]1N2C=C(C(=O)NC2=O)F)CO)O | 0 | train |
| 170465246 | C1=CC=C(C=C1)CSCC2=NS(=O)(=O)C3=CC(=C(C=C3N2)Cl)S(=O)(=O)N | 0 | train |
| 170465245 | CC(C)(C)C1=CC=C(C=C1)S(=O)(=O)NC2=C(C(=NC(=N2)C3=NC=CC=N3)OCCO)OC4=CC=CC=C4OC | 0 | validation |
| 170465244 | CNC(=O)C1=CN(N=C1)C2=NC3=C(C(=N2)N)N=CN3[C@H]4[C@@H]([C@@H]([C@H](O4)CO)O)O | 0 | test |
| 170465243 | CN1C(NC2=CC(=C(C=C2S1(=O)=O)S(=O)(=O)N)Cl)CCl | 0 | train |
| 170465242 | CC1=CC=C(C=C1)C2=C(N3C=C(C=CC3=N2)C)CC(=O)N(C)C | 1 | train |
| 170465241 | CC1=CC(=CC(=C1)OCC2CNC(=O)O2)C | 0 | validation |
| 170465240 | CC(C)COCC(CN(CC1=CC=CC=C1)C2=CC=CC=C2)N3CCCC3 | 1 | test |
| 170465239 | CC1=CC=C(C=C1)C(=O)OC2=C(C=C(C=C2)C(CNC(C)(C)C)O)OC(=O)C3=CC=C(C=C3)C | 0 | train |

| | | | |
|---|---|---|---|
| 170465238 | CCCCNC(=O)NS(=O)(=O)C1=CC=C(C=C1)C | 0 | train |
| 170465237 | CN(C)CCOC(C1=CC=C(C=C1)Cl)C2=CC=CC=N2.C(=C¥C(=O)O)¥C(=O)O | 0 | validation |
| 170465236 | C[C@@H]1[C@H]([C@H](C[C@@H](O1)O[C@H]2CC[C@]3([C@@H](C2)CC[C@@H]4[C@@H]3C[C@H]([C@]5([C@@]4(CC[C@@H]5C6=CC(=O)OC6)O)C)O)C)O)O[C@H]7C[C@@H]([C@@H]([C@H](O7)C)O[C@H]8C[C@@H]([C@@H]([C@H](O8)C)O[C@H]9C[C@@H]([C@H]([C@@H]([C@H](O9)CO)O)O)O)O)O | 1 | test |
| 170465235 | CCN1CCN(C(=O)C1=O)C(=O)N[C@H](C2=CC=C(C=C2)O)C(=O)N[C@H]3[C@@H]4N(C3=O)C(=C(CS4)CSC5=NN=NN5C)C(=O)[O-].[Na+] | 0 | train |
| 170465234 | C1CN(CCN1CCCN2C(=O)N3C=CC=CC3=N2)C4=CC(=CC=C4)Cl.Cl | 0 | train |
| 170465233 | CCC(C)C(C)(COC(=O)N)COC(=O)N | 0 | validation |
| 170465232 | CS(=O)(=O)OCCCCOS(=O)(=O)C | 0 | test |
| 170465231 | COC(=O)[C@H](C1=CC=CC=C1Cl)N2CCC3=C(C2)C=CS3.OS(=O)(=O)O | 1 | train |
| 170465230 | CC(C)(C1=CC=CC=C1CC[C@H](C2=CC=CC(=C2)/C=C/C3=NC4=C(C=CC(=C4)Cl)C=C3)SCC5(CC5)CC(=O)[O-])O.[Na+] | 0 | train |
| 170465229 | C1=CC=C(C=C1)CCNN.OS(=O)(=O)O | 1 | validation |
| 170465228 | CC(C)(C)NC(=O)[C@@H]1CN(CCN1C[C@H](C[C@@H](CC2=CC=CC=C2)C(=O)N[C@@H]3[C@@H](CC4=CC=CC=C34)O)O)CC5=CN=CC=C5.OS(=O)(=O)O | 0 | test |
| 170465227 | C1=CC(=C(C=C1[C@H](CN)O)O)O.C(C(C(=O)O)O)(C(=O)O)O.C(C(C(=O)O)O)(C(=O)O)O | 0 | train |
| 170465226 | C1=CC(=C(C=C1Cl)Cl)CO/N=C(¥CN2C=CN=C2)/C3=C(C=C(C=C3)Cl)Cl.[N+](=O)(O)[O-] | 1 | train |
| 170465225 | CC1(C2CCC(C2)C1(C)NC)C | 0 | validation |
| 170465224 | CN(C)[C@H]1[C@@H]2[C@H]([C@@H]3C(=C)C4=C(C(=CC=C4)O)C(=C3C(=O)[C@@]2(C(=C(C1=O)C(=O)N)O)O)O)O.Cl | 0 | test |
| 170465223 | CCC1(CCC(=O)NC1=O)C2=CC=CC=C2 | 1 | train |
| 170465222 | CC(C1=CC2=CC=CC=C2S1)N(C(=O)N)O | 0 | train |
| 170465221 | CC1=C(C=C(C(=O)N1)C#N)C2=CC=NC=C2 | 0 | validation |
| 170465220 | CC1=C(SC(=N1)C2=CC(=C(C=C2)OCC(C)C)C#N)C(=O)O | 1 | test |
| 170465219 | C1CN=C(N1)CC2=CC=CC=C2.Cl | 0 | train |
| 170465218 | C1C2CNCC1C3=CC4=NC=CN=C4C=C23.Cl | 0 | train |
| 170465217 | CC(C(=O)NCC(=O)O)S | 0 | validation |
| 170465216 | CC1=CC=C(C=C1)C(=O)C2=CC=C(N2C)CC(=O)[O-].O.O.[Na+] | 0 | test |
| 170465215 | CCN1C=C(C(=O)C2=C1N=C(C=C2)C)C(=O)O | 0 | train |
| 170465214 | C[N+]1(CCCC(C1)OC(=O)C(C2=CC=CC=C2)(C3=CC=CC=C3)O)C.[Br-] | 0 | train |
| 170465213 | CC(=O)NCC1=C(C(=C(C(=C1I)C(=O)O)I)NC(=O)C)I | 0 | validation |
| 170465212 | CCCCCCCC(=O)OCC(CO)O | 0 | test |
| 170465211 | CC[C@@]1(CC[C@@H]2[C@@]1(CC[C@H]3[C@H]2CCC4=CCCC[C@H]34)C)O | 0 | train |
| 170465210 | CCCCCC(=O)O[C@@]1(CC[C@@H]2[C@@]1(CC[C@H]3[C@H]2CCC4=CC(=O)CC[C@]34C)C)C(=O)C | 0 | train |
| 170465209 | COC1=C2C(=CC(=C1N3C[C@@H]4CCCN[C@@H]4C3)F)C(=O)C(=CN2C5CC5)C(=O)O.Cl | 0 | validation |
| 170465208 | C1CC2=C(C=CC(=C2)F)O[C@H]1[C@H](CNC[C@@H]([C@@H]3CCC4=C(O3)C=CC(=C4)F)O)O.Cl | 1 | test |
| 170465207 | C1CC1CN2CC[C@]34[C@@H]5C(=O)CC[C@]3([C@H]2CC6=C4C(=C(C=C6)O)O5)O | 0 | train |
| 170465206 | C1=CC(=C(C=C1N)O)C(=O)[O-].[Na+] | 0 | train |
| 170465205 | CN(C)[C@H]1[C@@H]2C[C@@H]3[C@@H](C4=C(C=CC(=C4C(=C3C(=O)[C@@]2(C(=C(C1=O)C(=O)N)O)O)O)O)Cl)O.Cl | 0 | validation |
| 170465204 | CCCN(CCC1=CC=CS1)[C@H]2CCC3=C(C2)C=CC=C3O | 0 | test |
| 170465203 | C1=CC=C(C=C1)C(=O)C2=C(C(=CC=C2)CC(=O)N)N | 0 | train |
| 170465202 | C1=CC(=C(C(=C1)F)CN2C=C(N=N2)C(=O)N)F | 0 | train |
| 170465201 | C1CC(=O)NC(=O)C1N2CC3=C(C2=O)C=CC=C3N | 0 | validation |
| 170465200 | COC1CN(CCC1NC(=O)C2=CC(=C(C=C2OC)N)Cl)CCCOC3=CC=C(C=C3)F | 0 | test |
| 170465199 | CC(C)[C@@H](CC1=CC(=C(C=C1)OC)OCCCOC)C[C@@H]([C@H](C[C@@H](C(C)C)C(=O)NCC(C)(C)C(=O)N)O)N.Cl | 0 | train |
| 170465198 | CC(=O)C1=CC(=C(C=C1)OCCCN2CCC(CC2)C3=NOC4=C3C=CC(=C4)F)OC | 0 | train |
| 170465197 | CCOCCN1C(=NC2=CC=CC=C12)N3CCN(CCC3)C.C(=C/C(=O)O)¥C(=O)O.C(=C/C(=O)O)¥C(=O)O | 0 | validation |
| 170465196 | CCS(=O)(=O)CCN1C(=NC=C1[N+](=O)[O-])C | 0 | test |
| 170465195 | CCOC(=O)O[C@@]1(CC[C@@H]2[C@@]1(C[C@@H]([C@H]3[C@H]2CCC4=CC(=O)C=C[C@]34C)O)C)C(=O)OCCl | 0 | train |
| 170465194 | C[C@H]1C[C@H]2[C@@H]3CC[C@@H]([C@]3(C[C@@H]([C@@H]2[C@@]4(C1=CC(=O)CC4)C)O)C)C(=O)C | 1 | train |
| 170465193 | C[C@]12CC[C@H]3[C@H]([C@@H]1CC[C@@H]2OC(=O)CCC4=CC=CC=C4)CCC5=CC(=O)CC[C@H]35 | 0 | validation |
| 170465192 | CC(C)NCCCC1(C2=CC=CC=C2C3=CC=CC=C31)C(=O)N | 0 | test |

| | | | |
|---|---|---|---|
| 170465191 | CCCCC(=O)O[C@@]1([C@@H](C[C@@H]2[C@@]1(C[C@@H]([C@]3([C@H]2CCC4=CC(=O)C=C[C@@]43C)F)O)C)C)C(=O)CO | 0 | train |
| 170465190 | CCCCNC1=CC=C(C=C1)C(=O)OCCN(C)C.Cl | 1 | train |
| 170465189 | C1=CC(=C(C(=C1)CC(=O)[O-])N)C(=O)C2=CC=C(C=C2)Br.C1=CC(=C(C(=C1)CC(=O)[O-])N)C(=O)C2=CC=C(C=C2)Br.O.O.O.[Na+].[Na+] | 0 | validation |
| 170465188 | C[C@]12C[C@@H]([C@]3([C@H]([C@@H]1C[C@H]([C@@]2(C(=O)CO)O)O)CCC4=CC(=O)C=C[C@@]43C)F)O | 0 | test |
| 170465187 | C1[C@H]([C@@H]2[C@H](O1)[C@H](CO2)O)O | 0 | train |
| 170465186 | CC[C@@]1(C[C@@H]2C[C@@](C3=C(CCN(C2)C1)C4=CC=CC=C4N3)(C5=C(C=C6C(=C5)[C@]78CCN9[C@H]7[C@@](C=CC9)([C@H]([C@@]([C@@H]8N6C=O)(C(=O)OC)O)OC(=O)C)CC)OC)C(=O)OC)O | 1 | train |
| 170465185 | C1=CC=C(C=C1)N2C(=CC=N2)NS(=O)(=O)C3=CC=C(C=C3)N | 0 | validation |
| 170465184 | C1=CC(=C(C=C1[N+](=O)[O-])Cl)NC(=O)C2=C(C=CC(=C2)Cl)O | 1 | test |
| 170465183 | C1=CC(=C2C(=C1NCCNCCO)C(=O)C3=C(C=CC(=C3C2=O)O)O)NCCNCCO.Cl.Cl | 1 | train |
| 170465182 | CC1=C(C(=C(C2=C1COC2=O)O)C/C=C(¥C)/CCC(=O)O)OC | 0 | train |
| 170465181 | CN(C)CC/C=C¥1/C2=CC=CC=C2COC3=C1C=C(C=C3)CC(=O)O.Cl | 0 | validation |
| 170465180 | C1=CC=C(C=C1)NC(=O)CCCCCCC(=O)NO | 1 | test |
| 170465179 | C(CN)CNCCSP(=O)(O)O | 0 | train |
| 170465178 | C1=CC=C(C=C1)C(COC(=O)N)COC(=O)N | 0 | train |
| 170465177 | CC(=O)O.C1C[C@H](N(C1)C(=O)[C@@H]2CSSCCC(=O)N[C@H](C(=O)N[C@H](C(=O)N[C@H](C(=O)N[C@H](C(=O)N2)CC(=O)N)CCC(=O)N)CC3=CC=CC=C3)CC4=CC=C(C=C4)O)C(=O)N[C@@H](CCCN=C(N)N)C(=O)NCC(=O)N | 0 | validation |
| 170465176 | CC1=C(C=CC2=C1C(=NC(=N2)N)N)CNC3=CC(=C(C(=C3)OC)OC)OC.Cl.Cl.Cl | 0 | test |
| 170465175 | CC1=CC=C(C=C1)S(=O)(=O)O.CNC(=O)C1=NC=CC(=C1)OC2=CC=C(C=C2)NC(=O)NC3=CC(=C(C=C3)Cl)C(F)(F)F | 0 | train |
| 170465174 | CC(C)(C)C#C/C=C/CN(C)CC1=CC=CC2=CC=CC=C21 | 1 | train |
| 170465173 | CC1=C(C(C(=C(N1)C)C(=O)OCCN(C)CC2=CC=CC=C2)C3=CC(=CC=C3)[N+](=O)[O-])C(=O)OC | 0 | validation |
| 170465172 | CN1CCCC(CC1)N2C(=O)C3=CC=CC=C3C(=N2)CC4=CC=C(C=C4)Cl.Cl | 0 | test |
| 170465171 | CC(C1=CC=CC=C1)(C2=CC=CC=N2)OCCN(C)C.C(CC(=O)O)C(=O)O | 0 | train |
| 170465170 | CCOC(=O)OC(C)OC(=O)[C@H]1C(S[C@H]2N1C(=O)[C@H]2NC(=O)[C@@H](C3=CC=CC=C3)N)(C)C.Cl | 0 | train |
| 170465169 | CCC1=NC=CC(=C1)C(=S)N | 0 | validation |
| 170465168 | CCN(C1=CC=CC(=C1)C2=CC=NC3=C(C=NN23)C#N)C(=O)C | 0 | test |
| 170465167 | CCC1NC2=CC(=C(C=C2C(=O)N1)S(=O)(=O)N)Cl | 0 | train |
| 170465166 | CC1C(OCCN1)C2=CC=CC=C2.Cl | 0 | train |
| 170465165 | C[C@H]1C[C@H]2[C@@H]3CCC4=CC(=O)C=C[C@@]4([C@H]3C(=O)C[C@@]2([C@]1(C(=O)CO)O)C)C | 0 | validation |
| 170465164 | CC(C12CC3CC(C1)CC(C3)C2)N.Cl | 0 | test |
| 170465163 | CC1=C(C(=NO1)C2=CC=CC=C2)C3=CC=C(C=C3)S(=O)(=O)N | 0 | train |
| 170465162 | CC(C)CC(C1(CCC1)C2=CC=C(C=C2)Cl)N(C)C.Cl | 0 | train |
| 170465161 | CN1C(S(=O)(=O)CCC1=O)C2=CC=C(C=C2)Cl | 0 | validation |
| 170465160 | CC(=O)N(CC(CO)O)C1=C(C(=C(C(=C1I)C(=O)NCC(CO)O)I)C(=O)NCCO)I | 0 | test |
| 170465159 | CC1=C(ON=C1C)N(C(=O)C)S(=O)(=O)C2=CC=C(C=C2)N | 0 | train |
| 170465158 | C1CN(CCN1C(=O)CCBr)C(=O)CCBr | 0 | train |
| 170465157 | CC(C)(C)CC(C)(C)C1=CC=C(C=C1)O | 0 | validation |
| 170465156 | C1=CC=C(C=C1)CC2NC3=C(C=C(C(=C3)C(F)(F)F)S(=O)(=O)N)S(=O)(=O)N2 | 0 | test |
| 170465155 | C1=CC(=CC=C1C(=O)NCC(=O)O)N | 0 | train |
| 170465154 | CC(=O)NC1=C(C=C(C(=C1I)C(=O)O)I)I | 0 | train |
| 170465153 | CC1=C(SC=C1)C(=CCCN2CCC[C@H](C2)C(=O)O)C3=C(C=CS3)C.Cl | 0 | validation |
| 170465152 | CC1([C@@H](N2[C@H](S1)[C@@H](C2=O)NC(=O)C3=C(C=CC=C3OC)OC)C(=O)[O-])C.O.[Na+] | 0 | test |
| 170465151 | CC#C[C@@]1(CC[C@@H]2[C@@]1(C[C@@H](C3=C4CCC(=O)C=C4CC[C@@H]23)C5=CC=C(C=C5)N(C)C)C)O | 0 | train |
| 170465150 | C(CN(CC(=O)O)CC(=O)O)N(CCN(CC(=O)O)CC(=O)O)CC(=O)O | 0 | train |
| 170465149 | C[C@@H]1CC(=O)[C@]2([C@@H](O1)O[C@@H]3[C@H]([C@@H]([C@@H]([C@@H]([C@H]3O2)NC)O)NC)O)O.Cl.Cl | 0 | validation |
| 170465148 | C[C@@]1([C@@H](N2[C@H](S1(=O)=O)CC2=O)C(=O)[O-])CN3C=CN=N3.[Na+] | 0 | test |
| 170465147 | CC(COC1=CC=CC=C1)N(CCCl)CC2=CC=CC=C2 | 1 | train |
| 170465146 | C1CN(CCN1CCOCCO)C2=NC3=CC=CC=C3SC4=CC=CC=C42 | 0 | train |
| 170465145 | C1=NC2=C(N1[C@H]3[C@H]([C@@H]([C@H](O3)CO)O)F)N=C(N=C2N)Cl | 0 | validation |
| 170465144 | C(C(C(C(C(F)(F)Br)(F)F)(F)F)(F)F)(C(C(C(F)(F)F)(F)F)(F)F)(F)F | 0 | test |

| | | | |
|---|---|---|---|
| 170465143 | CC1=CC(=NC(=N1)O[C@H](C(=O)O)C(C2=CC=CC=C2)(C3=CC=CC=C3)OC)C | 0 | train |
| 170465142 | CCN[C@@H]1C[C@@H]([C@H]([C@@H]([C@H]1O[C@@H]2[C@@H]([C@H]([C@@](CO2)(C)O)NC)O)O)OC3C(CC=C(O3)CN)N)N.CCN[C@@H]1C[C@@H]([C@H]([C@@H]([C@H]1O[C@@H]2[C@@H]([C@H]([C@@](CO2)(C)O)NC)O)O)OC3C(CC=C(O3)CN)N)N.OS(=O)(=O)O.OS(=O)(=O)O.OS(=O)(=O)O.OS(=O)(=O)O.OS(=O)(=O)O | 0 | train |
| 170465141 | CCOC(=O)NC1=CC2=C(C=C1)SC3=CC=CC=C3N2C(=O)CCN4CCOCC4.Cl | 0 | validation |
| 170465140 | CCCCN(CCCC)CCC(C1=C2C=CC(=CC2=C3C=C(C=C(C3=C1)Cl)Cl)C(F)(F)F)O.Cl | 0 | test |
| 170465139 | CCCN(CCC)CCC1=C2CC(=O)NC2=CC=C1 | 0 | train |
| 170465138 | CCN(CC)C(=O)C1CN2CCC3=CC(=C(C=C3C2CC1OC(=O)C)OC)OC | 0 | train |
| 170465137 | CC[C@@]1(C2=C(COC1=O)C(=O)N3CC4=C(C3=C2)N=C5C=CC(=C(C5=C4)CN(C)C)O)O.Cl | 0 | validation |
| 170465136 | CC1=CC=C(C=C1)S(=O)(=O)NC(=O)NN2CCCCCC2 | 0 | test |
| 170465135 | CCCCC1=NC(=C(N1CC2=CC=C(C=C2)C3=CC=CC=C3C4=NNN=N4)CO)Cl | 0 | train |
| 170465134 | CN/C(=C¥[N+](=O)[O-])/NCCSCC1=CC=C(O1)CN(C)C | 0 | train |
| 170465133 | C1=CC=C(C=C1)C(=O)NS(=O)(=O)C2=CC=C(C=C2)N | 0 | validation |
| 170465132 | CN(C)CCOC1=CC=C(C=C1)CNC(=O)C2=CC(=C(C(=C2)OC)OC)OC.Cl | 0 | test |
| 170465131 | CCOC(=O)[C@H](CCC1=CC=CC=C1)N[C@@H](C)C(=O)N2CC3=CC(=C(C=C3C[C@H]2C(=O)O)OC)OC.Cl | 0 | train |
| 170465130 | C1=CC=C(C=C1)C2C(=O)C3=CC=CC=C3C2=O | 0 | train |
| 170465129 | C1=CN(C(=O)N=C1N)[C@H]2C([C@@H]([C@H](O2)CO)O)(F)F.Cl | 0 | validation |
| 170465128 | C1C[C@@H](O[C@@H]1CO)N2C=CC(=NC2=O)N | 0 | test |
| 170465127 | CN1CCC(=C2C3=CC=CC=C3C=CC4=CC=CC=C42)CC1 | 0 | train |
| 170465126 | C1[C@@H]([C@H](O[C@H]1N2C=NC(=NC2=O)N)CO)O | 0 | train |
| 170465125 | CS(=O)(=O)CCNCC1=CC=C(O1)C2=CC3=C(C=C2)N=CN=C3NC4=CC(=C(C=C4)OCC5=CC(=CC=C5)F)Cl | 0 | validation |
| 170465124 | C1(=C(N=C(C(=N1)Cl)N)N)C(=O)N=C(N)N | 0 | test |
| 170465123 | CN(C)CC(C1=CC=C(C=C1)OC)C2(CCCCC2)O | 0 | train |
| 170465122 | CC(C)(C)NCC(COC1=CC=CC2=C1CCC(=O)N2)O.Cl | 0 | train |
| 170465121 | CN[C@H]1CC[C@H](C2=CC=CC=C12)C3=CC(=C(C=C3)Cl)Cl.Cl | 0 | validation |
| 170465120 | CCCN1CCCC[C@H]1C(=O)NC2=C(C=CC=C2C)C.Cl | 0 | test |
| 170465119 | CCN[C@H]1C[C@@H](S(=O)(=O)C2=C1C=C(S2)S(=O)(=O)N)C.Cl | 0 | train |
| 170465118 | CN(C)CCN(CC1=CC=CC=C1)C2=CC=CC=N2.C(C(=O)O)C(CC(=O)O)(C(=O)O)O | 0 | train |
| 170465117 | CCOC(=O)C1=CN=CN1C(C)C2=CC=CC=C2 | 1 | validation |
| 170465116 | CCC1=CN=C(C=C1)CCOC2=CC=C(C=C2)CC3C(=O)NC(=O)S3.Cl | 0 | test |
| 170465115 | CC[C@@H](CO)NCCN[C@@H](CC)CO.Cl.Cl | 0 | train |
| 170465114 | CC1=C(C(=CC=C1)C)NC(=O)C(C)N.Cl | 0 | train |
| 170465113 | CC(C)C1=C(C(=C(N1CC[C@H](C[C@H](CC(=O)[O-])O)O)C2=CC=C(C=C2)F)C3=CC=CC=C3)C(=O)NC4=CC=CC=C4.CC(C)C1=C(C(=C(N1CC[C@H](C[C@H](CC(=O)[O-])O)O)C2=CC=C(C=C2)F)C3=CC=CC=C3)C(=O)NC4=CC=CC=C4.[Ca+2] | 0 | validation |
| 170465112 | C[C@](CC1=CC=C(C=C1)O)(C(=O)O)N | 0 | test |
| 170465111 | C[N+]1(CCC2=CC(=C3C=C2[C@@H]1CC4=CC=C(C=C4)OC5=C6[C@@H](CC7=CC(=C(C=C7)OC)O3)[N+](CCC6=CC(=C5OC)OC)(C)C)OC)C.[I-].[I-] | 0 | train |
| 170465110 | C[C@H]1[C@@H](C(=O)N1S(=O)(=O)O)NC(=O)C(=NOC(C)(C)C(=O)O)C2=CSC(=N2)N | 0 | train |
| 170465109 | CC(C)C1=C(C(=C(C(=N1)C(C)C)/C=C/[C@H](C[C@H](CC(=O)[O-])O)O)C2=CC=C(C=C2)F)COC.[Na+] | 0 | validation |
| 170465108 | C1=CC=C(C=C1)CCCC(=O)[O-].[Na+] | 0 | test |
| 170465107 | CC(CN1C2=CC=CC=C2SC3=CC=CC=C31)N(C)C | 0 | train |
| 170465106 | CC1([C@@H](N2[C@H](S1)[C@@H](C2=O)NC(=O)C(C3=CC=CC=C3)C(=O)[O-])C(=O)[O-])C.[Na+].[Na+] | 0 | train |
| 170465105 | CO[C@@]1([C@@H]2N(C1=O)C(=C(CS2)COC(=O)N)C(=O)O)NC(=O)CC3=CC=CS3 | 0 | validation |
| 170465104 | CC(=O)[C@H]1CC[C@@H]2[C@@]1(CC[C@@H]3[C@H]2CC=C4C=C(=O)CC[C@@]34C)C | 0 | test |
| 170465103 | CN(C)CCN1C(=NN=N1)SCC2=C(N3[C@@H]([C@@H](C3=O)NC(=O)CC4=CSC(=N4)N)SC2)C(=O)O.Cl | 0 | train |
| 170465102 | C1=CC=C(C=C1)CC(=O)O | 0 | train |
| 170465101 | CC(=O)OCC1=C(N2[C@@H]([C@@H](C2=O)NC(=O)CC3=CC=CS3)SC1)C(=O)O | 0 | validation |
| 170465100 | C1CCN[C@H](C1)[C@H](C2=CC(=NC3=C2C=CC=C3C(F)(F)F)C(F)(F)F)O.Cl | 1 | test |
| 170465099 | C1=CC(=CC=C1C(CC(=O)O)CN)Cl | 0 | train |
| 170465098 | C(CC(O)(P(=O)(O)O)P(=O)(O)[O-])CN.O.O.O.[Na+] | 0 | train |
| 170465097 | C[C@@H]1[C@H]2[C@@H]([C@H]3[C@@H](C(=O)C(=C([C@]3(C(=O)C2=C(C4=C1C=CC=C4O)O)O)O)O)C(=O)N)N(C)C)O.Cl | 0 | validation |

| | | | |
|---|---|---|---|
| 170465096 | CC1([C@@H](N2[C@H](S1)[C@@H](C2=O)NC(=O)CC3=CC=CC=C3)C(=O)O)C.CC1([C@@H](N2[C@H](S1)[C@@H](C2=O)NC(=O)CC3=CC=CC=C3)C(=O)O)C.C1=CC=C(C=C1)CNCCNCC2=CC=CC=C2 | 0 | test |
| 170465095 | C[C@@H](C(=O)N[C@@H](C)C(=O)NC1([C@H]2[C@@H]1CN(C2)C3=C(C=C4C(=O)C(=CN(C4=N3)C5=C(C=C(C=C5)F)F)C(=O)O)F)C)N.CS(=O)(=O)O | 1 | train |
| 170465094 | CN1CCN(CC1)C2=NC3=CC=CC=C3OC4=C2C=C(C=C4)Cl.C(CC(=O)O)C(=O)O | 1 | train |
| 170465093 | CC(C)(C)C1=CC=C(C=C1)CN(C)CC2=CC=CC3=CC=CC=C32 | 0 | validation |
| 170465092 | CCCCS(=O)(=O)N[C@@H](CC1=CC=C(C=C1)OCCCCC2CCNCC2)C(=O)O.O.Cl | 0 | test |
| 170465091 | CC1=C(C(=C2CCC(OC2=C1C)(C)COC3=CC=C(C=C3)CC4C(=O)NC(=O)S4)C)O | 0 | train |
| 170465090 | CC(=O)OC1=CC=CC=C1C(=O)NC2=NC=C(S2)[N+](=O)[O-] | 1 | train |
| 170465089 | CC1=NC=C(N=C1)C(=O)NCCC2=CC=C(C=C2)S(=O)(=O)NC(=O)NC3CCCCC3 | 0 | validation |
| 170465088 | CC(CNC1CCCCC1)OC(=O)C2=CC=CC=C2.Cl | 0 | test |
| 170465087 | B([C@H](CC(C)C)NC(=O)[C@H](CC1=CC=CC=C1)NC(=O)C2=NC=CN=C2)(O)O | 1 | train |
| 170465086 | C[C@]12C[C@@H]([C@H]3[C@H]([C@@H]1CC[C@@]2(C(=O)CO)O)C[C@@H](C4=CC(=O)C=C[C@]34C)F)O | 0 | train |
| 170465085 | C[C@@H]1[C@@H]2[C@H](C(=O)N2C(=C1S[C@H]3C[C@H](NC3)CNS(=O)(=O)N)C(=O)O)[C@@H](C)O | 0 | validation |
| 170465084 | C1=CC2=C(C(=C(C=C2Cl)Cl)O)N=C1 | 1 | test |
| 170465083 | CCOC1=CC2=C(C=C1)N=C(S2)S(=O)(=O)N | 1 | train |
| 170465082 | C1NC2=C(C=C(C(=C2)C(F)(F)F)S(=O)(=O)N)S(=O)(=O)N1 | 0 | train |
| 170465081 | CC1=CC=C(C=C1)C(=O)C2=CC(=C(C(=C2)O)O)[N+](=O)[O-] | 1 | validation |
| 170465080 | CN1CCCCC1CCN2C3=CC=CC=C3SC4=C2C=C(C=C4)SC | 1 | test |
| 170465079 | C1CN(C[C@@H]1C(C2=CC=CC=C2)(C3=CC=CC=C3)C(=O)N)CCC4=CC5=C(C=C4)OCC5.Br | 0 | train |
| 170465078 | CCC1=NN(C(=O)N1CCOC2=CC=CC=C2)CCCN3CCN(CC3)C4=CC(=CC=C4)Cl.Cl | 0 | train |
| 170465077 | CNCCCC1C2=CC=CC=C2C=CC3=CC=CC=C13.Cl | 1 | validation |
| 170465076 | C1=NC(=NC(=O)N1[C@H]2[C@@H]([C@@H]([C@H](O2)CO)O)O)N | 0 | test |
| 170465075 | C1CNC[C@H]([C@@H]1C2=CC=C(C=C2)F)COC3=CC4=C(C=C3)OCO4 | 0 | train |
| 170465074 | CS(=O)(=O)O.C1[C@@H]2[C@@H](C2N)CN1C3=C(C=C4C(=O)C(=CN(C4=N3)C5=C(C=C(C=C5)F)F)C(=O)O)F | 1 | train |
| 170465073 | COC1=C(C=C2C(=C1)C(=NC(=N2)N3CCN(CC3)C(=O)C4CCCO4)N)OC.Cl | 0 | validation |
| 170465072 | CC(CCCN)NC1=C2C(=CC(=C1)OC)C=CC=N2.OP(=O)(O)O.OP(=O)(O)O | 0 | test |
| 170465071 | CCCCC1=NC=C(N1CC2=CC=C(C=C2)C(=O)O)/C=C(¥CC3=CC=CS3)/C(=O)O.CS(=O)(=O)O | 0 | train |
| 170465070 | CC1=C(C(C(=C(N1)C)C(=O)OC(C)C)C2=CC=CC3=NON=C32)C(=O)OC | 1 | train |
| 170465069 | CN(C)CC/C=C/1¥C2=CC=CC=C2SC3=C1C=C(C=C3)Cl | 0 | validation |
| 170465068 | CN1C(NC2=CC(=C(C=C2S1(=O)=O)S(=O)(=O)N)Cl)CSCC(F)(F)F | 0 | test |
| 170465067 | CC1=CC=CC=C1O[C@H](CCNC)C2=CC=CC=C2.Cl | 0 | train |
| 170465066 | CN(CC#C)CC1=CC=CC=C1.Cl | 0 | train |
| 170465065 | CCN(CC)C(C)CN1C2=CC=CC=C2SC3=CC=CC=C31.Cl | 0 | validation |
| 170465064 | CCCCC1=C(C2=C(O1)C=CC(=C2)NS(=O)(=O)C)C(=O)C3=CC=C(C=C3)OCCCN(CCCC)CCCC | 1 | test |
| 170465063 | CC1=CC2=C(C=C1C)N(C3=NC(=O)NC(=O)C3=N2)C[C@@H]([C@@H]([C@@H](CO)O)O)O | 0 | train |
| 170465062 | CC(C)(C)NC[C@@H](COC1=NSN=C1N2CCOCC2)O | 0 | train |
| 170465061 | C[C@@H]1CN(CC[C@@]1(C2=CC=CC=C2)C(=O)O)C3CCC(CC3)(C#N)C4=CC=C(C=C4)F.Cl | 0 | validation |
| 170465060 | C(=O)([O-])P(=O)([O-])[O-].[Na+].[Na+].[Na+] | 0 | test |
| 170465059 | CN¥1C=CC=C/C1=C¥[NH+]=O.[Cl-] | 0 | train |
| 170465058 | CC(=O)NC1=C(C(=C(C(=C1I)C(=O)[O-])I)C(=O)NC)I.[Na+] | 0 | train |
| 170465057 | C(CCC(=O)O)CCN | 0 | validation |
| 170465056 | CCC(CC1=C(C(=C(C=C1I)I)N)I)C(=O)O | 1 | test |
| 170465055 | C1=CC(=CC=C1CN)S(=O)(=O)N.Cl | 0 | train |
| 170465054 | C[C@]12CC[C@H]3[C@H]([C@@H]1CC[C@]2(C#C)O)CCC4=C3C=CC(=C4)OC5CCCC5 | 0 | train |
| 170465053 | C[C@]12CCC(=O)C=C1CC[C@@H]3[C@@H]2[C@H](C[C@]4([C@H]3CC[C@@]4(C(=O)COC(=O)CCC(=O)[O-])O)C)O.[Na+] | 0 | validation |
| 170465052 | CN1C(=NN=N1)SCC2=C(N3[C@@H]([C@@](C3=O)(NC(=O)CSCC#N)OC)SC2)C(=O)[O-].[Na+] | 0 | test |
| 170465051 | C[C@H]1C[C@H]2[C@@H]3CC[C@@]([C@]3(C[C@@H]([C@@H]2[C@@]4(C1=CC(=O)C=C4)C)O)C)(C(=O)CO)O | 0 | train |
| 170465050 | CC(CCC1=CC=C(C=C1)O)NCCC2=CC(=C(C=C2)O)O | 0 | train |
| 170465049 | CN(C)[C@H]1[C@@H]2C[C@@H]3CC4=C(C=CC(=C4C(=C3C(=O)[C@@]2(C(=C(C1=O)C(=O)N)O)O)O)O)N(C)C.Cl | 0 | validation |

| | | | |
|---|---|---|---|
| 170465048 | C[C@H]1[C@H]([C@H](C[C@@H](O1)O[C@H]2C[C@@](CC3=C(C4=C(C(=C23)O)C(=O)C5=C(C4=O)C=CC=C5OC)O)(C(=O)C)O)N)O | 1 | test |
| 170465047 | CCC1=C(NC2=C1C(=O)C(CC2)CN3CCOCC3)C.Cl | 0 | train |
| 170465046 | C1=CC=C2C(=C1)C(=C3C=CC=CC3=N2)N.O.Cl | 1 | train |
| 170465045 | CC1=C(C(CCC1)(C)C)/C=C/C(=C/C=C/C(=C/C(=O)O)/C)/C | 1 | validation |
| 170465044 | C1=CC(=CN=C1)C(=O)O | 0 | test |
| 170465043 | C1C[C@H]2C(=O)N[C@@H](CSSCCC(=O)N[C@H](C(=O)NCC(=O)N[C@H](C(=O)N[C@H](C(=O)N2C1)CC3=CNC4=CC=CC=C43)CC(=O)O)CCCCN=C(N)N)C(=O)N | 0 | train |
| 170465042 | C1=CC=C(C=C1)C(C2=CC=CC=C2)S(=O)CC(=O)N | 0 | train |
| 170465041 | COC1=C2C(=CC3=C1OC=C3)C=CC(=O)O2 | 1 | validation |
| 170465040 | CCCCC1C(=O)N(N(C1=O)C2=CC=CC=C2)C3=CC=CC=C3 | 0 | test |
| 170465039 | CC(=O)OC1=CC=CC=C1C(=O)O | 0 | train |
| 170465038 | C[C@H](C(=O)O)N | 0 | train |
| 170465037 | C[N+](C)(C)CC(CC(=O)[O-])O.Cl | 0 | validation |
| 170465036 | CC1(CCCC(N1C)(C)C)C.[C@H]([C@@H](C(=O)O)O)(C(=O)O)O | 0 | test |
| 170465035 | CC1COC2=C3N1C=C(C(=O)C3=CC(=C2N4CCN(CC4)C)F)C(=O)O | 0 | train |
| 170465034 | CC(C)(C)NCC(C1=CC(=C(C=C1)O)CO)O | 0 | train |
| 170465033 | CC[C@]12CC[C@H]3[C@H]([C@@H]1CC[C@]2(C#C)O)CCC4=CC(=O)CC[C@H]34 | 0 | validation |
| 170465032 | COC1=CC2=C(C=CN=C2C=C1)[C@H]([C@@H]3CC4CCN3C[C@@H]4C=C)O | 0 | test |
| 170465031 | COC1=CC2=C(C=CN=C2C=C1)[C@@H]([C@H]3CC4CCN3C[C@@H]4C=C)O | 0 | train |
| 170465030 | C1=CC=C(C=C1)CNCCNCC2=CC=CC=C2.Cl.Cl | 0 | train |
| 170465029 | C1CN(CCN1CCOCC(=O)N)C(C2=CC=CC=C2)C3=CC=C(C=C3)Cl.Cl.Cl | 0 | validation |
| 170465028 | CN(C)CC[C@@H](C1=CC=C(C=C1)Br)C2=CC=CC=N2.C(=C¥C(=O)O)¥C(=O)O | 0 | test |
| 170465027 | C[C@H]1C[C@H]2[C@@H]3CCC4=CC(=O)C=C[C@@]4([C@]3([C@H](C[C@@]2([C@]1(C(=O)CO)O)C)O)F)C | 0 | train |
| 170465026 | CC1=CNN=C1.Cl | 0 | train |
| 170465025 | C(=O)(N)NO | 0 | validation |
| 170465024 | C1=CC(=C(C(=C1)Cl)Cl)C2=C(N=C(N=N2)N)N | 0 | test |
| 170465023 | CN(CC(CO)O)C(=O)C1=C(C(=C(C(=C1I)C(=O)NCC(CO)O)I)NC(=O)COC)I | 0 | train |
| 170465022 | CN(CCCNC(=O)C1CCCO1)C2=NC3=CC(=C(C=C3C(=N2)N)OC)OC.Cl | 0 | train |
| 170465021 | CC1(C(=O)N(C(=O)N1)C2=CC(=C(C=C2)[N+](=O)[O-])C(F)(F)F)C | 0 | validation |
| 170465020 | C[C@]12CC[C@H]3[C@H]([C@@H]1CCC2=O)CCC4=C3C=CC(=C4)O | 1 | test |
| 170465019 | C1=CC2=C(C(=C(C=C2Cl)I)O)N=C1 | 0 | train |
| 170465018 | CCN(CC)CCN1C(=O)CN=C(C2=C1C=CC(=C2)Cl)C3=CC=CC=C3F | 0 | train |
| 170465017 | CN(CCCl)CCCl.Cl | 0 | validation |
| 170465016 | C1=CC=C(C=C1)C2(C(=O)N(C(=O)N2)COP(=O)(O)O)C3=CC=CC=C3 | 0 | test |
| 170465015 | CC1=NC=C2N1C3=C(C=C(C=C3)Cl)C(=NC2)C4=CC=CC=C4F.Cl | 0 | train |
| 170465014 | C1[C@@H]([C@H](O[C@H]1N2C=C(C(=O)NC2=O)I)CO)O | 1 | train |
| 170465013 | C1=CC(=CC=C1[C@@H]2[C@H](C(=O)N2C3=CC=C(C=C3)F)CC[C@@H](C4=CC=C(C=C4)F)O)O | 0 | validation |
| 170465012 | C[C@@]1(C(=O)N2[C@H](C(=O)N3CCC[C@H]3[C@@]2(O1)O)CC4=CC=CC=C4)NC(=O)[C@@H]5C[C@H]6[C@@H](CC7=CNC8=CC=CC6=C78)N(C5)C | 0 | test |
| 170465011 | CC(=O)N[C@H]1CCC2=CC(=C(C(=C2C3=CC=C(C(=O)C=C13)OC)OC)OC)OC | 1 | train |
| 170465010 | COC1=CC(=C(C=C1)C(=O)C2=CC=CC=C2)O | 1 | train |
| 170465009 | CC1=CC2=C(NC3=CC=CC=C3N=C2S1)N4CCN(CC4)C | 0 | validation |
| 170465008 | C=CCN1CC[C@]23[C@@H]4C(=O)CC[C@]2([C@H]1CC5=C3C(=C(C=C5)O)O4)O | 0 | test |
| 170465007 | C(CC(C(F)F)(C(=O)O)N)CN.Cl | 0 | train |
| 170465006 | CC1=C(C(C(=C(N1)C)C(=O)OC)C2=CC=CC=C2[N+](=O)[O-])C(=O)OC | 0 | train |
| 170465005 | C1NC2=CC(=C(C=C2S(=O)(=O)N1)S(=O)(=O)N)Cl | 0 | validation |
| 170465004 | CCCCOC1=CC=C(C=C1)OCCCN2CCOCC2.Cl | 1 | test |
| 170465003 | CCOC(=O)[C@H](CCC1=CC=CC=C1)N[C@H]2CCC3=CC=CC=C3N(C2=O)CC(=O)O.Cl | 0 | train |
| 170465002 | C1=C(NN=C1)CCN.Cl | 0 | train |
| 170465001 | C1=C(NC=N1)CCN | 0 | validation |
| 170465000 | CCCCC[C@@H](/C=C/[C@H]1[C@@H](CC(=O)[C@@H]1C/C=C¥CCCC(=O)O)O)O | 0 | test |
| 170464999 | CC12CC3CC(C1)(CC(C3)(C2)N)C | 0 | train |
| 170464998 | CC1=CC(=C(C=C1)C)OCCCC(C)(C)C(=O)O | 0 | train |
| 170464997 | CC1=CC(=O)C2=CC=CC=C2C1=O | 1 | validation |
| 170464996 | CC1=C(C(=O)N2CCC[C@H](C2=N1)O)CCN3CCC(CC3)C4=NOC5=C4C=CC(=C5)F | 0 | test |
| 170464995 | CC(C)NCC(COC1=CC=CC=C1OCC=C)O.Cl | 0 | train |
| 170464994 | CC(C)C1=C(C(=CC=C1)C(C)C)O | 0 | train |
| 170464993 | C[C@H](CC1=CC=CC=C1)N(C)CC#C.Cl | 0 | validation |
| 170464992 | C1=CC(=CN=C1)CC(O)(P(=O)(O)[O-])P(=O)(O)[O-].[Na+].[Na+] | 0 | test |

| | | | |
|---|---|---|---|
| 170464991 | C1=CC=C2C(=C1)C(=O)OC23C4=C(C=C(C=C4)[O-])OC5=C3C=CC(=C5)[O-].[Na+].[Na+] | 0 | train |
| 170464990 | CC(C)(C)NCC(C1=CC(=CC(=C1)O)O)O.CC(C)(C)NCC(C1=CC(=CC(=C1)O)O)O.OS(=O)(=O)O | 0 | train |
| 170464989 | CN1C=NC(=C1SC2=NC=NC3=C2NC=N3)[N+](=O)[O-] | 0 | validation |
| 170464988 | CCC(C(=O)N)N1CCCC1=O | 0 | test |
| 170464987 | C1=CC=C(C=C1)C2=C(OC(=N2)CCC(=O)O)C3=CC=CC=C3 | 0 | train |
| 170464986 | C1=CC=C(C=C1)C(=O)OOC(=O)C2=CC=CC=C2 | 0 | train |
| 170464985 | C1=NC2=C(N1)C(=S)N=CN2 | 0 | validation |
| 170464984 | CCC(C)(C)C(=O)O[C@H]1C[C@H](C=C2[C@H]1[C@H]([C@H](C=C2)C)CC[C@@H]3C[C@H](CC(=O)O3)O)C | 0 | test |
| 170464983 | C[C@]12CC[C@H]3[C@H]([C@@H]1CC[C@]2(C#C)O)CCC4=C3C=CC(=C4)O | 0 | train |
| 170464982 | CC1=C(N2[C@@H]([C@@H](C2=O)NC(=O)[C@@H](C3=CC=CC=C3)N)SC1)C(=O)O | 0 | train |
| 170464981 | C[C@H]1C[C@H]2[C@@H]3CCC4=CC(=O)C=C[C@]4([C@]3([C@H](C[C@@]2([C@]1(C(=O)CO)O)C)O)Cl)C | 0 | validation |
| 170464980 | C1[C@H]([C@@H]2[C@H](O1)[C@H](CO2)O[N+](=O)[O-])O[N+](=O)[O-] | 0 | test |
| 170464979 | CC(C)CC1=CC=C(C=C1)C(C)C(=O)O | 0 | train |
| 170464978 | CCCC1=C2C(=CC3=C1OC(=CC3=O)C(=O)O)C(=O)C=C(N2CC)C(=O)O | 0 | train |
| 170464977 | CC[C@@]1(C[C@H]2C[C@@](C3=C(CCN(C2)C1)C4=CC=CC=C4N3)(C5=C(C=C6C(=C5)[C@]78CCN9[C@H]7[C@@](C=CC9)([C@H]([C@@]([C@@H]8N6C)(C(=O)OC)O)OC(=O)C)CC)OC)C(=O)OC)O.OS(=O)(=O)O | 1 | validation |
| 170464976 | CC[C@@]1(C[C@H]2C[C@@](C3=C(CCN(C2)C1)C4=CC=CC=C4N3)(C5=C(C=C6C(=C5)[C@]78CCN9[C@H]7[C@@](C=CC9)([C@H]([C@@]([C@@H]8N6C)(C(=O)OC)O)OC(=O)C)CC)OC)C(=O)OC)O.OS(=O)(=O)O | 1 | test |
| 170464975 | CCC(=O)O[C@@]1([C@@H](C[C@@H]2[C@@]1(C[C@@H]([C@]3(C2C[C@@H](C4=CC(=O)C=C[C@@]43C)F)F)O)C)C)C(=O)SCF | 0 | train |
| 170464974 | CNC[C@@H](C1=CC(=C(C=C1)O)O)O.Cl | 0 | train |
| 170464973 | CNC[C@@H](C1=CC(=C(C=C1)O)O)O | 0 | validation |
| 170464972 | C[N+](C)(C)CCOC(=O)N.[Cl-] | 0 | test |
| 170464971 | CC(C[N+](C)(C)C)OC(=O)C.[Cl-] | 0 | train |
| 170464970 | CC(=O)CCC1=CC2=C(C=C1)C=C(C=C2)OC | 1 | train |
| 170464969 | CCOC(=O)[C@H](CCC1=CC=CC=C1)N[C@@H](C)C(=O)N2CCC[C@H]2C(=O)O | 0 | validation |
| 170464968 | CN1C(=O)N2C=NC(=C2N=N1)C(=O)N | 0 | test |
| 170464967 | C1CCC(CC1)NC(=O)N(CCCl)N=O | 0 | train |
| 170464966 | CCC1=C(CN(C1=O)C(=O)NCCC2=CC=C(C=C2)S(=O)(=O)NC(=O)NC3CCC(CC3)C)C | 0 | train |
| 170464965 | CCCC1=CC(=O)NC(=S)N1 | 0 | validation |
| 170464964 | COC1=C(C=C(C=C1)Cl)C(=O)NCCC2=CC=C(C=C2)S(=O)(=O)NC(=O)NC3CCCCC3 | 0 | test |
| 170464963 | CC[C@@H]([C@H](C)O)N1C(=O)N(C=N1)C2=CC=C(C=C2)N3CCN(CC3)C4=CC=C(C=C4)OCC5C[C@](OC5)(CN6C=NC=N6)C7=C(C=C(C=C7)F)F | 1 | train |
| 170464962 | CC(=O)N(CC(CO)O)C1=C(C(=C(C(=C1I)C(=O)NCC(CO)O)I)C(=O)NCC(CO)O)I | 0 | train |
| 170464961 | C1=NC2=C(N1COC(CO)CO)NC(=NC2=O)N | 0 | validation |
| 170464960 | CCOC(=O)C1=C2CN(C(=O)C3=C(N2C=N1)C=CC(=C3)F)C | 0 | test |
| 170464959 | C[C@]12CCC(=O)C=C1CC[C@@H]3[C@@]2([C@H](C[C@]4([C@H]3CC[C@@]4(C(=O)CO)O)C)O)F | 0 | train |
| 170464958 | C1CN(CCN1CCOCCO)C(C2=CC=CC=C2)C3=CC=C(C=C3)Cl | 0 | train |
| 170464957 | C1=CC2=C(C=C1Cl)NC(=O)O2 | 0 | validation |
| 170464956 | C1=CC(=C(C=C1C2=C(C=C(C=C2)F)F)C(=O)O)O | 1 | test |
| 170464955 | C1=NC2=C(N1CCC(CO)CO)NC(=NC2=O)N | 0 | train |
| 170464954 | C1CCCN(CCC1)CCN=C(N)N.OS(=O)(=O)O.OS(=O)(=O)O | 0 | train |
| 170464953 | CN(C)CCC=C1C2=CC=CC=C2C=CC3=CC=CC=C31.Cl | 0 | validation |
| 170464952 | CCN(CC)CCNC(=O)C1=CC=C(C=C1)N | 0 | test |
| 170464951 | CCN(CC)CCOC1=CC=C(C=C1)C(=C(C2=CC=CC=C2)Cl)C3=CC=CC=C3 | 0 | train |
| 170464950 | CCCCN1C[C@@H]([C@H]([C@@H]([C@H]1CO)O)O)O | 0 | train |
| 170464949 | CCC(C)N1C(=O)N(C=N1)C2=CC=C(C=C2)N3CCN(CC3)C4=CC=C(C=C4)OC[C@H]5CO[C@](O5)(CN6C=NC=N6)C7=C(C=C(C=C7)Cl)Cl | 1 | validation |
| 170464948 | CC1=NC=C(N1CCO)[N+](=O)[O-] | 0 | test |
| 170464947 | CC(C)N(CCC(C1=CC=CC=C1)(C2=CC=CC=N2)C(=O)N)C(C)C | 0 | train |
| 170464946 | C1=C(OC(=C1)[N+](=O)[O-])/C=N/NC(=O)N | 1 | train |
| 170464945 | CC(C)(C)NCC(COC1=CC=CC2=C1C[C@@H]([C@@H](C2)O)O)O | 0 | validation |
| 170464944 | CC(=O)NS(=O)(=O)C1=CC=C(C=C1)N | 0 | test |
| 170464943 | C1CC(C2=CC=CC=C2C1)C3=NCCN3.Cl | 0 | train |
| 170464942 | C1=CC(=C(C=C1CCN)O)O | 0 | train |
| 170464941 | C1CN=C(N1)NC2=C(C3=NC=CN=C3C=C2)Br | 0 | validation |

| | | | |
|---|---|---|---|
| 170464940 | C(CNCCNCCNCCN)N | 0 | test |
| 170464939 | C1=CC=C2C(=C1)NC(=N2)C3=CSC=N3 | 1 | train |
| 170464938 | C[C@@H](C(=O)N1CCC[C@H]1C(=O)O)N[C@H](CCC2=CC=CC=C2)C(=O)O | 0 | train |
| 170464937 | CCN(CC)CCOC(=O)C1=CC=C(C=C1)N.Cl | 0 | validation |
| 170464936 | C1=CC(=C(C=C1Cl)O)OC2=C(C=C(C=C2)Cl)Cl | 0 | test |
| 170464935 | C1=C2C(=NC=NC2=O)NN1 | 0 | train |
| 170464934 | C[C@H]([C@@H]1[C@H]2CC(=C(N2C1=O)C(=O)O)SCCN=CN)O | 0 | train |
| 170464933 | CC(=O)O[C@H]1CC[C@@H]2[C@H]3CC[C@]4([C@H]([C@@H]3CCC2=C1)CC[C@]4(C#C)OC(=O)C)C | 0 | validation |
| 170464932 | CN1CCC[C@@H]1C2=CN=CC=C2 | 0 | test |
| 170464931 | CC1=CC=C(C=C1)/C(=C¥CN2CCCC2)/C3=CC=CC=N3 | 0 | train |
| 170464930 | CCC1=C(C(=NC(=N1)N)N)C2=CC=C(C=C2)Cl | 0 | train |
| 170464929 | CN1C=NC2=C1C(=O)N(C(=O)N2C)C.C(C(=O)O)C(CC(=O)O)(C(=O)O)O | 0 | validation |
| 170464928 | C1=CC2=C(C=C1OC(F)(F)F)SC(=N2)N | 1 | test |
| 170464927 | C1=CC=C(C(=C1)C(C2=CC=C(C=C2)Cl)C(Cl)Cl)Cl | 1 | train |
| 170464926 | CCN[C@H]1CN(S(=O)(=O)C2=C1C=C(S2)S(=O)(=O)N)CCCOC | 0 | train |
| 170464925 | CC1=C(C(=O)N2CCCCC2=N1)CCN3CCC(CC3)C4=NOC5=C4C=CC(=C5)F | 0 | validation |
| 170464924 | CC(=O)N(CC(CN(C1=C(C(=C(C(=C1I)C(=O)NCC(CO)O)I)C(=O)NCC(CO)O)I)C(=O)C)O)C2=C(C(=C(C(=C2I)C(=O)NCC(CO)O)I)C(=O)NCC(CO)O)I | 0 | test |
| 170464923 | CC(C)(C#N)C1=CC(=CC(=C1)CN2C=NC=N2)C(C)(C)C#N | 0 | train |
| 170464922 | CC(C)CC(C(=O)N[C@H](CCCN=C(N)N)C(=O)N1CCC[C@@H]1C(=O)NCC(=O)N)NC(=O)CNC(=O)[C@H](CC2=CC=C(C=C2)O)NC(=O)[C@@H](CO)NC(=O)[C@@H](CC3=CNC4=CC=CC=C43)NC(=O)C(CC5=CN=CN5)NC(=O)[C@H]6CCC(=O)N6.Cl | 0 | train |
| 170464921 | CC(C)C(=O)NC1=CC(=C(C=C1)[N+](=O)[O-])C(F)(F)F | 1 | validation |
| 170464920 | CCCCC1=C(C2=CC=CC=C2O1)C(=O)C3=CC(=C(C(=C3)I)OCCN(CC)CC)I | 0 | test |
| 170464919 | CC1=C2[C@H](C(=O)[C@@]3([C@H](C[C@@H]4[C@]([C@H]3[C@@H]([C@@](C2(C)C)(C[C@@H]1OC(=O)[C@@H]([C@H](C5=CC=CC=C5)NC(=O)OC(C)(C)C)O)O)OC(=O)C6=CC=CC=C6)(CO4)OC(=O)C)O)C)O | 0 | train |
| 170464918 | C1CC[N+]2(C1)[C@@H]3CC[C@H]2CC(C3)OC(=O)C(C4=CC=CC=C4)(C5=CC=CC=C5)O.[Cl-] | 0 | train |
| 170464917 | C[C@]12CC[C@H]3[C@H]([C@@H]1CC[C@@H]2O)[C@@H](CC4=C3C=CC(=C4)O)CCCCCCCCCS(=O)CCCC(C(F)(F)F)(F)F | 0 | validation |
| 170464916 | CC1=CC(=C(C(=C1CC2=NCCN2)C)O)C(C)(C)C | 0 | test |
| 170464915 | CCC1=CC=CC2=C1NC3=C2CCOC3(CC)CC(=O)O | 0 | train |
| 170464914 | COC(=O)NC1=NC2=C(N1)C=C(C=C2)C(=O)C3=CC=CC=C3 | 1 | train |
| 170464913 | CC[C@H](C)C(=O)O[C@H]1C[C@H](C=C2[C@H]1[C@H]([C@H](C=C2)C)CC[C@@H]3C[C@H](CC(=O)O3)O)C | 0 | validation |
| 170464912 | CC(C[N+](C)(C)C)OC(=O)N.[Cl-] | 0 | test |
| 170464911 | C1[C@@H]([C@H](O[C@H]1N2C=C(C(=O)NC2=O)C(F)(F)F)CO)O | 0 | train |
| 170464910 | CC1=CC=CC=C1C(C2=CC=CC=C2)OCCN(C)C.C(C(=O)O)C(CC(=O)O)(C(=O)O)O | 0 | train |
| 170464909 | C1CCC2(C1)CC(=O)N(C(=O)C2)CCCCN3CCN(CC3)C4=NC=CC=N4 | 0 | validation |
| 170464908 | C1=NC2=C(N1)C(=S)N=C(N2)N | 0 | test |
| 170464907 | C1C[C@H](N(C1)C(=O)[C@H](CCCCN)N[C@@H](CCC2=CC=CC=C2)C(=O)O)C(=O)O.O.O | 0 | train |
| 170464906 | C1CCC(CC1)C(=O)N2CC3C4=CC=CC=C4CCN3C(=O)C2 | 0 | train |
| 170464905 | C1=CC(=CN=C1)C(=O)N | 0 | validation |
| 170464904 | CC(C)C(CCCN(C)CCC1=CC(=C(C=C1)OC)OC)(C#N)C2=CC(=C(C=C2)OC)OC | 0 | test |
| 170464903 | CN(C/C=C/C1=CC=CC=C1)CC2=CC=CC3=CC=CC=C32.Cl | 1 | train |
| 170464902 | C[NH+](C)CCC(C1=CC=CC=C1)C2=CC=CC=[NH+]2.C(=C¥C(=O)[O-])¥C(=O)[O-] | 0 | train |
| 170464901 | C1=CC(=C(C(=C1)Cl)SC(CCC2=CC=C(C=C2)Cl)CN3C=CN=C3)Cl.[N+](=O)(O)[O-] | 1 | validation |
| 170464900 | C1CN=C(N1)NC2=C(C=CC=C2Cl)Cl | 0 | test |
| 170464899 | CC1=CC(=CC=C1)CN2CCN(CC2)C(C3=CC=CC=C3)C4=CC=C(C=C4)Cl.Cl.Cl | 0 | train |
| 170464898 | CCCNC(=O)NS(=O)(=O)C1=CC=C(C=C1)Cl | 0 | train |
| 170464897 | CCCSC1=CC2=C(C=C1)N=C(N2)NC(=O)OC | 1 | validation |
| 170464896 | CCN(CC)CCOC(=O)C1(CCCCC1)C2CCCCC2 | 1 | test |
| 170464895 | C1=CC(=CC=C1N)S(=O)(=O)C2=CC=C(C=C2)N | 0 | train |
| 170464894 | CCCCC(CC)COC(=O)C=CC1=CC=C(C=C1)OC | 1 | train |
| 170464893 | CC1=CC(=NC(=N1)NS(=O)(=O)C2=CC=C(C=C2)N)C | 0 | validation |
| 170464892 | CC(C)NCC(COC1=CC=CC2=C1C=CN2)O | 0 | test |
| 170464891 | CC(C)C1=NC(=CS1)CN(C)C(=O)N[C@@H](C(C)C)C(=O)N[C@@H](CC2=CC=CC=C2)C[C@@H]([C@H](CC3=CC=CC=C3)NC(=O)OCC4=CN=CS4)O | 0 | train |
| 170464890 | C[C@@H](C1=NC=NC=C1F)[C@](CN2C=NC=N2)(C3=C(C=C(C=C3)F)F)O | 0 | train |
| 170464889 | [C-]#N.[C-]#N.[C-]#N.[C-]#N.[C-]#N.[N-]=O.O.O.[Na+].[Na+].[Fe+4] | 0 | validation |

| | | | |
|---|---|---|---|
| 170464888 | C1=C(C(=C(C(=C1I)NC(=O)CCCCC(=O)NC2=C(C=C(C(=C2I)C(=O)O)I)I)I)C(=O)O)I | 0 | test |
| 170464887 | C1=CC2=C(C(=C1)OCC(COC3=CC=CC4=C3C(=O)C=C(O4)C(=O)[O-])O)C(=O)C=C(O2)C(=O)[O-].[Na+].[Na+] | 0 | train |
| 170464886 | CC1=C(C(=C(C=C1)Cl)NC2=CC=CC=C2C(=O)[O-])Cl.O.[Na+] | 0 | train |
| 170464885 | C1C(=C(N2[C@H](S1)[C@@H](C2=O)NC(=O)[C@@H](C3=CC=CC=C3)N)C(=O)O)Cl | 0 | validation |
| 170464884 | C[C@]12CC[C@H]3[C@H]([C@@H]1CC[C@]2(C#C)O)CCC4=CC5=C(C[C@]34C)C=NO5 | 0 | test |
| 170464883 | [C@@H]([C@@H]([C@H](C(=O)O)O)O)([C@@H](C(=O)O)O)O | 0 | train |
| 170464882 | CN1CCC2=CC(=C3C=C2[C@@H]1CC4=CC=C(C=C4)OC5=C6[C@@H](CC7=CC(=C(C=C7)O)O3)[N+](CCC6=CC(=C5O)OC)(C)C)OC.Cl.[Cl-] | 0 | train |
| 170464881 | CC(C)[N+](C)(CCOC(=O)C1C2=CC=CC=C2OC3=CC=CC=C13)C(C)C.[Br-] | 0 | validation |
| 170464880 | C(CCl)NC(=O)N(CCCl)N=O | 0 | test |
| 170464879 | C1=COC(=C1)CNC2=CC(=C(C=C2C(=O)O)S(=O)(=O)N)Cl | 0 | train |
| 170464878 | C[N+]1=CC=CC(=C1)OC(=O)N(C)C.[Br-] | 0 | train |
| 170464877 | C1=C(N=C(S1)N=C(N)N)CSCCC(=NS(=O)(=O)N)N | 0 | validation |
| 170464876 | C[N+]1(CCC(=C(C2=CC=CC=C2)C3=CC=CC=C3)CC1)C.COS(=O)(=O)[O-] | 0 | test |
| 170464875 | CN(C)CCC1=CNC2=C1C=C(C=C2)C[C@H]3COC(=O)N3 | 0 | train |
| 170464874 | C1CCNC(C1)CNC(=O)C2=C(C=CC(=C2)OCC(F)(F)F)OCC(F)(F)F | 0 | train |
| 170464873 | CCCC1=NC2=C(C=C(C=C2N1CC3=CC=C(C=C3)C4=CC=CC=C4C(=O)O)C5=NC6=CC=CC=C6N5C)C | 0 | validation |
| 170464872 | CC1=CC2=C(C=C1C)N(C=N2)[C@@H]3[C@@H]([C@@H]([C@H](O3)CO)OP(=O)([O-])O[C@H](C)CNC(=O)CC[C@@]4([C@H]([C@@H]5[C@]6([C@@]([C@@H](/C(=C(/C7=N/C(=C¥C8=N/C(=C(¥C4=N5)/C)/[C@H](C8(C)C)CCC(=O)N)/[C@H]([C@]7(C)CC(=O)N)CCC(=O)N)¥C)/[N-]6)CCC(=O)N)(C)CC(=O)N)C)CC(=O)N)C)O.[C-]#N.[Co+3] | 0 | test |
| 170464871 | C[C@@H]1CCN([C@H](C1)C(=O)O)C(=O)[C@H](CCCN=C(N)N)NS(=O)(=O)C2=CC=CC3=C2NC[C@@H](C3)C | 0 | train |
| 170464870 | C[C@]12CCC(=O)C=C1[C@@H]3C[C@@H]3[C@@H]4[C@@H]2CC[C@]5([C@H]4[C@@H]6C[C@@H]6[C@@]57CCC(=O)O7)C | 0 | train |
| 170464869 | CC1=C2C(=NC=C1)N(C3=C(C=CC=N3)C(=O)N2)C4CC4 | 0 | validation |
| 170464868 | C1CC(C1)(C(=O)O)C(=O)O.[NH2-].[NH2-].[Pt+2] | 0 | test |
| 170464867 | CCOC(=O)N1CCC(=C2C3=C(CCC4=C2N=CC=C4)C=C(C=C3)Cl)CC1 | 1 | train |
| 170464866 | CC1=CC=C(C=C1)N(CC2=NCCN2)C3=CC(=CC=C3)O | 0 | train |
| 170464865 | CN1CCC(=C2C3=CC=CC=C3CCC4=C2N=CC=C4)CC1 | 0 | validation |
| 170464864 | C1CN(CCC1N2C3=CC=CC=C3NC2=O)CCCC(C4=CC=C(C=C4)F)C5=CC=C(C=C5)F | 0 | test |
| 170464863 | C1C[C@@H](O[C@@H]1CO)N2C=NC3=C2NC=NC3=O | 0 | train |
| 170464862 | C(CS(=O)(=O)[O-])S.[Na+] | 0 | train |
| 170464861 | C([C@@H]1[C@@H]([C@@H]([C@H]([C@@H](O1)O[C@@H]2[C@H](O[C@@]([C@H]2O)(CO)O)CO)O)O)O)O | 0 | validation |
| 170464860 | C1C2CC3CC1CC(C2)(C3)N | 0 | test |
| 170464859 | CC1=C(C(=C(C2=C1COC2=O)O)C/C=C(¥C)/CCC(=O)OCCN3CCOCC3)OC | 0 | train |
| 170464858 | COC1=C(C=C2C(=C1)CC(C2=O)CC3CCN(CC3)CC4=CC=CC=C4)OC.Cl | 0 | train |
| 170464857 | CC1=CN=C(S1)NC(=O)C2=C(C3=CC=CC=C3S(=O)(=O)N2C)O | 0 | validation |
| 170464856 | CN1C=CNC1=S | 0 | test |
| 170464855 | COC1=CC=CC=C1OCC(CO)O | 0 | train |
| 170464854 | C1CN(P(=O)(OC1)NCCCl)CCCl | 0 | train |
| 170464853 | CN1CCN(CC1)C2=C3C=CC=CC3=NC4=C(N2)C=C(C=C4)Cl | 0 | validation |
| 170464852 | CN(C)C(=O)C(CCN1CCC(CC1)(C2=CC=C(C=C2)Cl)O)(C3=CC=CC=C3)C4=CC=CC=C4 | 1 | test |
| 170464851 | CCN(C1=CC=CC=C1C)C(=O)C=CC | 1 | train |
| 170464850 | CCN(CC)C(=O)/C(=C/C1=CC(=C(C(=C1)O)O)[N+](=O)[O-])/C#N | 1 | train |
| 170464849 | CCOC(=O)C1=C(NC(=C(C1C2=C(C(=CC=C2)Cl)Cl)C(=O)OC)C)C | 0 | validation |
| 170464848 | CCN(CC)CCNC(=O)C1=CC(=C(C=C1OC)N)Cl | 0 | test |
| 170464847 | C[C@]12CC(=O)[C@H]3[C@H]([C@@H]1CC[C@@]2(C(=O)CO)O)CCC4=CC(=O)C=C[C@]34C | 0 | train |
| 170464846 | CCC1(C(=O)NCNC1=O)C2=CC=CC=C2 | 0 | train |
| 170464845 | CC[C@H]1[C@H](COC1=O)CC2=CN=CN2C | 0 | validation |
| 170464844 | C[N+](C)(C)CC(=O)O.[Cl-] | 0 | test |
| 170464843 | CC(=O)OC1=CC=C(C=C1)C(C2=CC=C(C=C2)OC(=O)C)C3=CC=CC=N3 | 1 | train |
| 170464842 | C(C(C(=O)O)N)S | 0 | train |
| 170464841 | C1CN(CCC1(C2=CC=C(C=C2)Cl)O)CCCC(=O)C3=CC=C(C=C3)F | 1 | validation |
| 170464840 | C1=CC(=C(C=C1N)C(=O)O)O | 0 | test |
| 170464839 | CCCN(CCC)S(=O)(=O)C1=CC=C(C=C1)C(=O)O | 0 | train |
| 170464838 | C[C@@]1([C@H]2C[C@H]3[C@@H](C(=O)C(=C([C@]3(C(=O)C2=C(C4=C1C=CC=C4O)O)O)O)C(=O)N)N(C)C)O.Cl | 0 | train |

| | | | |
|---|---|---|---|
| 170464837 | CC(=O)OCC(=O)[C@]1([C@@H](C[C@@H]2[C@@]1(C[C@@H]([C@]3([C@H]2CCC4=CC(=O)C=C[C@@]43C)F)O)C)OC(=O)C)O | 0 | validation |
| 170464836 | CCC[C@@H](C(=O)OCC)N[C@@H](C)C(=O)N1[C@H]2CCCC[C@H]2C[C@H]1C(=O)O | 0 | test |
| 170464835 | CCC(=O)O[C@H]1CC[C@@H]2[C@@]1(CC[C@H]3[C@H]2CCC4=CC(=O)CC[C@]34C)C | 0 | train |
| 170464834 | C1CN(CCN1CCCN2C3=CC=CC=C3SC4=C2C=C(C=C4)Cl)CCO | 1 | train |
| 170464833 | C1=CC(=CC=C1C(=O)N[C@@H](CCC(=O)O)C(=O)O)NCC2=CN=C3C(=N2)C(=O)N=C(N3)N | 0 | validation |
| 170464832 | C[C@]12C[C@@H]([C@H]3[C@H]([C@@H]1C[C@@H]4[C@]2(OC(O4)(C)C)C(=O)CO)CCC5=CC(=O)C=C[C@]35C)O | 0 | test |
| 170464831 | CC1([C@@H](N2[C@H](S1)[C@@H](C2=O)NC(=O)[C@@H](C3=CC=CC=C3)N)C(=O)O)C | 0 | train |
| 170464830 | CN1[C@@H]2CC(C[C@H]1[C@H]3[C@@H]2O3)OC(=O)[C@H](CO)C4=CC=CC=C4.Br | 0 | train |
| 170464829 | CCCCOC1=NC2=CC=CC=C2C(=C1)C(=O)NCCN(CC)CC | 0 | validation |
| 170464828 | COC1=C(C=C2C(=C1)N=CN=C2NC3=CC(=C(C=C3)F)Cl)OCCCN4CCOCC4 | 0 | test |
| 170464827 | CN(C(=O)N[C@@H]1[C@H]([C@@H]([C@H](O[C@@H]1O)CO)O)O)N=O | 0 | train |
| 170464826 | CCOC1=CC=CC=C1OCCN[C@H](C)CC2=CC(=C(C=C2)OC)S(=O)(=O)N.Cl | 0 | train |
| 170464825 | CCCNC(C)C(=O)NC1=C(SC=C1C)C(=O)OC.Cl | 0 | validation |
| 170464824 | CCC1=C2C=C(C=CC2=NC3=C1CN4C3=CC5=C(C4=O)COC(=O)[C@@]5(CC)O)OC(=O)N6CCC(CC6)N7CCCCC7.Cl | 0 | test |
| 170464823 | CC1=C(C=NO1)C(=O)NC2=CC=C(C=C2)C(F)(F)F | 1 | train |
| 170464822 | CC(C)C1CCC(CC1)C(=O)N[C@H](CC2=CC=CC=C2)C(=O)O | 0 | train |
| 170464821 | CNC(=O)CN(CCN(CCN(CC(=O)NC)CC(=O)[O-])CC(=O)[O-])CC(=O)[O-].O.[Gd+3] | 0 | validation |
| 170464820 | CC1=CN(C(=O)NC1=O)[C@H]2C=C[C@H](O2)CO | 0 | test |
| 170464819 | CN1CC(=O)N2[C@@H](C1=O)CC3=C([C@H]2C4=CC5=C(C=C4)OCO5)NC6=CC=CC=C36 | 0 | train |
| 170464818 | C[C@]12CC[C@H]3[C@H]([C@@H]1CC[C@@H]2C(=O)NC(C)(C)C)CC[C@@H]4[C@@]3(C=CC(=O)N4)C | 0 | train |
| 170464817 | CN1C(=C(C2=CC=CC=C2S1(=O)=O)O)C(=O)NC3=CC=CC=N3 | 0 | validation |
| 170464816 | CN1CCC(CC1)OC(C2=CC=CC=C2)C3=CC=CC=C3.Cl | 1 | test |
| 170464815 | CC(C)NCC(COC1=CC=C(C=C1)COCCOC(C)C)O.CC(C)NCC(COC1=CC=C(C=C1)COCCOC(C)C)O.C(=C/C(=O)O)¥C(=O)O | 0 | train |
| 170464814 | CC1=C(C(=O)C2=C(C1=O)N3C[C@H]4[C@@H]([C@@]3([C@@H]2COC(=O)N)OC)N4)N | 1 | train |
| 170464813 | CC1=C(C(=CC=C1)NC2=CC=CC=C2C(=O)O)C | 0 | validation |
| 170464812 | C1=C2C(=CC(=C1Cl)S(=O)(=O)N)S(=O)(=O)NC(N2)C(Cl)Cl | 0 | test |
| 170464811 | C1C2=CC=CC=C2N(C3=CC=CC=C3C1=O)C(=O)N | 0 | train |
| 170464810 | C1CC1C#C[C@]2(C3=C(C=CC(=C3)Cl)NC(=O)O2)C(F)(F)F | 1 | train |
| 170464809 | COCCCC/C(=N¥OCCN)/C1=CC=C(C=C1)C(F)(F)F | 0 | validation |
| 170464808 | COC1=CC=CC=C1OCC(COC(=O)N)O | 0 | test |
| 170464807 | C1=CC=C(C=C1)C(C2=CC=CC=C2)(C3=CC=CC=C3Cl)N4C=CN=C4 | 1 | train |
| 170464806 | C1=CC=C2C(=C1)C=NN=C2NN.Cl | 1 | train |
| 170464805 | C1CCC(CC1)C(CCN2CCCC2)(C3=CC=CC=C3)O.Cl | 0 | validation |
| 170464804 | CN(C)C(=N)N=C(N)N.Cl | 0 | test |
| 170464803 | CCC(=C(CC)C1=CC=C(C=C1)O)C2=CC=C(C=C2)O | 0 | train |
| 170464802 | CCC1(CC(=O)NC1=O)C | 0 | train |
| 170464801 | CC[N+](C)(C)C1=CC(=CC=C1)O.[Cl-] | 0 | validation |
| 170464800 | CC(C)N=C(N)/N=C(¥N)/NC1=CC=C(C=C1)Cl.Cl | 0 | test |
| 170464799 | CC(C1=CC2=C(C=C1)C3=C(N2)C=CC(=C3)Cl)C(=O)O | 0 | train |
| 170464798 | CC1=C(C(=O)C2=CC=CC=C2C1=O)C/C=C(¥C)/CCC[C@H](C)CCC[C@H](C)CCCC(C)C | 0 | train |
| 170464797 | C1CN=C(N1)CC2=CC3=CC=CC=C3C=C2.Cl | 0 | validation |
| 170464796 | C[C@H](C1=CC(=CC(=C1)C(F)(F)F)C(F)(F)F)O[C@@H]2[C@@H](N(CCO2)CC3=NC(=O)N=N3)C4CCC(CC4)F | 0 | test |
| 170464795 | C[C@@H]1C[C@H]2[C@@H]3CCC4=CC(=O)C=C[C@@]4([C@]3([C@H](C[C@@]2([C@H]1C(=O)CO)C)O)F)C | 0 | train |
| 170464794 | C[C@@H]([C@@H](C1=CC(=C(C=C1)O)O)O)N | 0 | train |
| 170464793 | C1C(=O)N=C(N1N=CC2=CC=C(O2)C3=CC=C(C=C3)[N+](=O)[O-])[O-].[Na+] | 0 | validation |
| 170464792 | CC(=O)N[C@@H](CS)C(=O)O | 0 | test |
| 170464791 | C1=CC=C(C=C1)C(=O)NCC(=O)O | 0 | train |
| 170464790 | CC(=O)CC(C1=CC=CC=C1)C2=C(C3=CC=CC=C3OC2=O)[O-].[K+] | 0 | train |
| 170464789 | C[C@]12CC[C@H]3[C@H]([C@@H]1CC[C@@H]2OC(=O)CCC4CCCC4)CCC5=C3C=CC(=C5)O | 1 | validation |
| 170464788 | COC1=CC(=CC(=C1OC)OC)[C@H]2[C@@H]3[C@H](COC3=O)[C@H](C4=CC5=C(C=C24)OCO5)O | 1 | test |
| 170464787 | CN(C)C1=CC=C(C=C1)C(=C2C=CC(=[N+](C)C)C=C2)C3=CC=C(C=C3)N(C)C.[Cl-] | 1 | train |

| | | | |
|---|---|---|---|
| 170464786 | C1=CC(=CC=C1C(=N)N)OCCCCCOC2=CC=C(C=C2)C(=N)N.C(CS(=O)(=O)O)O.C(CS(=O)(=O)O)O | 0 | train |
| 170464785 | C1=CC=C(C=C1)C2(C(=O)NC(=O)N2)C3=CC=CC=C3 | 0 | validation |
| 170464784 | C[C@@H]1[C@H]([C@@H]([C@H]([C@H](O1)O[C@@H]2[C@H](O[C@@H]([C@@H]([C@H]2O)O)O[C@@H]3[C@H](O[C@H]([C@@H]([C@H]3O)O)O)CO)CO)O)O)N[C@H]4C=C([C@H]([C@@H]([C@H]4O)O)O)CO | 0 | test |
| 170464783 | CC(=O)OCC[N+](C)(C)C.[Cl-] | 0 | train |
| 170464782 | C1=CC(=C(C=C1F)F)C(CN2C=NC=N2)(CN3C=NC=N3)O | 0 | train |
| 170464781 | C1CC2=C(C=CC(=C2)Cl)C(=C3CCNCC3)C4=C1C=CC=N4 | 1 | validation |
| 170464780 | CNC(=C[N+](=O)[O-])NCCSCC1=CSC(=N1)CN(C)C | 0 | test |
| 170464779 | CCCCCOC(=O)NC1=NC(=O)N(C=C1F)[C@H]2[C@@H]([C@@H]([C@H](O2)C)O)O | 0 | train |
| 170464778 | CNCCCC12CCC(C3=CC=CC=C31)C4=CC=CC=C24 | 1 | train |
| 170464777 | CN(C)CCCN1C2=CC=CC=C2CCC3=CC=CC=C31 | 0 | validation |
| 170464776 | CCCCOC1=CC=C(C=C1)C(=O)CCN2CCCCC2.Cl | 0 | test |
| 170464775 | CCCCC(=O)O[C@@]1(CC[C@@H]2[C@@]1(C[C@@H]([C@H]3[C@H]2CCC4=CC(=O)CC[C@]34C)O)C)C(=O)CO | 0 | train |
| 170464774 | CC(=O)OCC(CCN1C=NC2=CN=C(N=C21)N)COC(=O)C | 0 | train |
| 170464773 | CC1=C(C(=CC=C1)C)OCC(=O)N[C@@H](CC2=CC=CC=C2)[C@H](C[C@H](CC3=CC=CC=C3)NC(=O)[C@H](C(C)C)N4CCCNC4=O)O | 0 | validation |
| 170464772 | CC(C)(C)C1=CC(=CC(=C1O)C(C)(C)C)SC(C)(C)SC2=CC(=C(C(=C2)C(C)(C)C)O)C(C)(C)C | 0 | test |
| 170464771 | CCCCOC1=C(C=CC(=C1)C(=O)OCCN(CC)CC)N.Cl | 0 | train |
| 170464770 | C1=CC(=CC=C1CCCC(=O)O)N(CCCl)CCCl | 0 | train |
| 170464769 | CCN1C=C(C(=O)C2=CC(=C(C=C21)N3CCNCC3)F)C(=O)O | 0 | validation |
| 170464768 | C[N+]12CCC(CC1)C(C2)OC(=O)C(C3=CC=CC=C3)(C4=CC=CC=C4)O.[Br-] | 0 | test |
| 170464767 | CCN(CC)C(=S)SSC(=S)N(CC)CC | 1 | train |
| 170464766 | CN(C)CCCN1C2=CC=CC=C2SC3=CC=CC=C31.Cl | 0 | train |
| 170464765 | C1CN=C(N1)CN(CC2=CC=CC=C2)C3=CC=CC=C3.Cl | 0 | validation |
| 170464764 | C1=NC2=C(N1[C@H]3[C@H]([C@@H]([C@H](O3)CO)O)O)N=C(N=C2N)F | 0 | test |
| 170464763 | C1[C@H]2[C@@H]([C@@H](S1)CCCCC(=O)O)NC(=O)N2 | 0 | train |
| 170464762 | C1=C(C(=O)NC(=O)N1)F | 0 | train |
| 170464761 | CN1CCN(CC1)CCCN2C3=C(SC4=CC=CC=C24)C=CC(=C3)Cl.C(=C¥C(=O)O)¥C(=O)O.C(=C¥C(=O)O)¥C(=O)O | 0 | validation |
| 170464760 | C[C@H]1/C=C/C=C(¥C(=O)NC¥2=C(C3=C(C(=C4C(=C3C(=O)/C2=C¥NN5CCN(CC5)C)C(=O)[C@](O4)(O/C=C/[C@@H]([C@H]([C@H]([C@@H]([C@@H]([C@@H]([C@H]1O)C)O)C)OC(=O)C)C)OC)C)C)O)O)/C | 0 | test |
| 170464759 | COC1=NC(=NC2=C1N=CN2[C@H]3[C@H]([C@@H]([C@H](O3)CO)O)O)N | 0 | train |
| 170464758 | CN(C)CCC=C1C2=CC=CC=C2CCC3=CC=CC=C31.Cl | 0 | train |
| 170464757 | C1CC1N2C=C(C(=O)C3=CC(=C(C=C32)N4CCNCC4)F)C(=O)O.Cl | 0 | validation |
| 170464756 | C1CCC(CC1)C(CCN2CCCCC2)(C3=CC=CC=C3)O.Cl | 1 | test |
| 170464755 | CCCNCC(COC1=CC=CC=C1C(=O)CCC2=CC=CC=C2)O | 0 | train |
| 170464754 | CCN(CC1=CC=NC=C1)C(=O)C(CO)C2=CC=CC=C2 | 0 | train |
| 170464753 | CCN(CC)CCCC(C)NC1=C2C=CC(=CC2=NC=C1)Cl.OP(=O)(O)O.OP(=O)(O)O | 0 | validation |
| 170464752 | CC(=O)N(CCCCCNC(=O)CCC(=O)N(CCCCCNC(=O)CCC(=O)N(CCCCCN)O)O)O.CS(=O)(=O)O | 0 | test |
| 170464751 | COC1=C(N=CN=C1OC)NS(=O)(=O)C2=CC=C(C=C2)N | 0 | train |
| 170464750 | CCC(/C=C¥Cl)(C#C)O | 0 | train |
| 170464749 | CC(C)(C)C1=CC=C(C=C1)C(=O)CC(=O)C2=CC=C(C=C2)OC | 0 | validation |
| 170464748 | C1=CC(=CC=C1N/C(=N/C(=NCCCCCCN=C(/N=C(/NC2=CC=C(C=C2)Cl)¥N)N)N)/N)Cl | 0 | test |
| 170464747 | CC(C(=O)C1=CC(=CC=C1)Cl)NC(C)(C)C.Cl | 0 | train |
| 170464746 | CC1(O[C@@H]2CO[C@@]3([C@H]([C@@H]2O1)OC(O3)(C)C)COS(=O)(=O)N)C | 0 | train |
| 170464745 | C1=CC=C2C(=C1)C(=O)NC2(C3=CC(=C(C=C3)Cl)S(=O)(=O)N)O | 0 | validation |
| 170464744 | CC(C)NC[C@H](COC1=CC=C(C=C1)CC(=O)N)O | 0 | test |
| 170464743 | CC(C)(C)NC[C@@H](COC1=CC=CC2=C1CCCC2=O)O.Cl | 0 | train |
| 170464742 | CC(C)(C)C(=O)OCOP(=O)(COCCN1C=NC2=C1N=CN=C2N)OCOC(=O)C(C)(C)C | 0 | train |
| 170464741 | CCCCOCCOCCOCC1=CC2=C(C=C1CCC)OCO2 | 1 | validation |
| 170464740 | CC1=C(C(=CC=C1)C)OCC(C)N.Cl | 0 | test |
| 170464739 | C1C(=O)NC(=O)N1N=CC2=CC=C(O2)[N+](=O)[O-] | 1 | train |
| 170464738 | CC1=NC(=NC=C1)NS(=O)(=O)C2=CC=C(C=C2)N | 0 | train |
| 170464737 | CC1=CN(C(=O)NC1=O)[C@H]2C[C@@H]([C@H](O2)CO)N=[N+]=[N-] | 0 | validation |
| 170464736 | CC1=C(C=CN=C1CS(=O)C2=NC3=CC=CC=C3[N-]2)OCCCOC.[Na+] | 1 | test |
| 170464735 | CCCCNC1=C(C(=CC(=C1)C(=O)O)S(=O)(=O)N)OC2=CC=CC=C2 | 0 | train |
| 170464734 | CCCC(C)C1(C(=O)NC(=NC1=O)[S-])CC.[Na+] | 0 | train |

| | | | |
|---|---|---|---|
| 170464733 | CC[C@]12CC[C@H]3[C@H]([C@@H]1CC[C@]2(C#C)OC(=O)C)CCC4=C/C(=N/O)/CC[C@H]34 | 0 | validation |
| 170464732 | C[C@]12C[C@@H]([C@H]3[C@H]([C@@H]1CC[C@@]2(C(=O)CO)O)CCC4=CC(=O)C=C[C@]34C)O | 0 | test |
| 170464731 | CCOC(=O)[C@H](CCC1=CC=CC=C1)N[C@@H](C)C(=O)N2[C@H]3CCC[C@H]3C[C@H]2C(=O)O | 0 | train |
| 170464730 | CC(O)(P(=O)(O)[O-])P(=O)(O)[O-].[Na+].[Na+] | 0 | train |
| 170464729 | CCN(CC)CC(=O)NC1=C(C=CC=C1C)C | 0 | validation |
| 170464728 | C([C@@H]([C@@H]1C(=C(C(=O)O1)O)O)O)O | 0 | test |
| 170464727 | C1=CC(=CC=C1C[C@@H](C(=O)O)N)N(CCCl)CCCl | 0 | train |
| 170464726 | [NH2-].[NH2-].Cl[Pt+2]Cl | 0 | train |
| 170464725 | C(CO)N(C1=C(C(=C(C(=C1I)C(=O)NCC(CO)O)I)C(=O)NCC(CO)O)I)C(=O)CO | 0 | validation |
| 170464724 | C1=CC=C(C=C1)COC2=CC=C(C=C2)O | 1 | test |
| 170464723 | C1=C/C(=C/C=C/2¥N/C(=C¥3/C=CC=CC3=O)/N(N2)C4=CC=C(C=C4)C(=O)O)/C(=O)C=C1 | 0 | train |
| 170464722 | C1CN(CC=C1N2C3=CC=CC=C3NC2=O)CCCC(=O)C4=CC=C(C=C4)F | 0 | train |
| 170464721 | CCN(C)C(=O)OC1=CC=CC(=C1)[C@H](C)N(C)C.[C@H]([C@@H](C(=O)O)O)(C(=O)O)O | 0 | validation |
| 170464720 | CN(C)CCOC(=O)C(C1=CC=CC=C1)C2(CCCC2)O.Cl | 0 | test |
| 170464719 | CCCCC(CC)COC(=O)C(=C(C1=CC=CC=C1)C2=CC=CC=C2)C#N | 1 | train |
| 170464718 | CC(C)OC(=O)C(C)(C)OC1=CC=C(C=C1)C(=O)C2=CC=C(C=C2)Cl | 1 | train |
| 170464717 | CC(=O)OCC(=O)[C@@]12[C@@H](C[C@@H]3[C@@]1(C[C@@H]([C@]4([C@H]3C[C@@H](C5=CC(=O)C=C[C@@]54C)F)F)O)C)OC(O2)(C)C | 0 | validation |
| 170464716 | C1=CC=NC(=C1)NS(=O)(=O)C2=CC=C(C=C2)N/N=C¥3/C=CC(=O)C(=C3)C(=O)O | 0 | test |
| 170464715 | C1CCC(CC1)(CC(=O)O)CN | 0 | train |
| 170464714 | CN(C)C1=NC(=NC(=N1)N(C)C)N(C)C | 0 | train |
| 170464713 | C[C@@H]1[C@H]([C@H](C[C@@H](O1)O[C@@H]2[C@H](O[C@H](C[C@@H]2O)O[C@@H]3[C@H](O[C@H](C[C@@H]3O)O[C@H]4CC[C@]5([C@@H](C4)CC[C@@H]6[C@@H]5CC[C@]7([C@@]6(CC[C@@H]7C8=CC(=O)OC8)O)C)C)C)C)O)O | 1 | validation |
| 170464712 | C1=CC=C2C(=C1)C=CC3=CC=CC=C3N2C(=O)N | 0 | test |
| 170464711 | CCCCCCCCCC(=O)O[C@H]1CC[C@@H]2[C@@]1(CC[C@H]3[C@H]2CCC4=CC(=O)CC[C@H]34)C | 0 | train |
| 170464710 | C[C@H](/C=C/[C@H](C1CC1)O)[C@H]2CC[C@@H]¥3[C@@]2(CCC/C3=C¥C=C/4¥C[C@H](C[C@@H](C4=C)O)O)C | 1 | train |
| 170464709 | C[C@@H](CN1CC(=O)NC(=O)C1)N2CC(=O)NC(=O)C2 | 0 | validation |
| 170464708 | CC1=CC(=O)N(C(=C1)C2CCCCC2)O.C(CO)N | 1 | test |
| 170464707 | C1=CC=NC(=C1)NS(=O)(=O)C2=CC=C(C=C2)N | 0 | train |
| 170464706 | C1=CC=C(C=C1)C2=NC3=C(N=C2N)N=C(N=C3N)N | 0 | train |
| 170464705 | CC1=C(C2=C(N1C(=O)C3=CC=C(C=C3)Cl)C=CC(=C2)OC)CC(=O)O | 0 | validation |
| 170464704 | C1CCN(CC1)C2=NC(=N)N(C(=C2)N)O | 0 | test |
| 170464703 | C1=CN=C(C=N1)C(=O)N | 0 | train |
| 170464702 | CN(C)CCCN1C2=CC=CC=C2CCC3=C1C=C(C=C3)Cl | 0 | train |
| 170464701 | CC1=NC=CN1CC2CCC3=C(C2=O)C4=CC=CC=C4N3C | 0 | validation |
| 170464700 | C(CC(=O)O)C(=O)CN.Cl | 0 | test |
| 170464699 | CC(=O)O[C@@H]1[C@@H](SC2=CC=CC=C2N(C1=O)CCN(C)C)C3=CC=C(C=C3)OC | 0 | train |
| 170464698 | C1CN(CC2=C1SC=C2)CC3=CC=CC=C3Cl | 1 | train |
| 170464697 | CCCNC(C)C(=O)NC1=CC=CC=C1C.Cl | 0 | validation |
| 170464696 | CCN(CCCC(C)NC1=C2C=CC(=CC2=NC=C1)Cl)CCO.OS(=O)(=O)O | 0 | test |
| 170464695 | C[C@@H]1C[C@H]2[C@@H]3CCC4=CC(=O)C=C[C@@]4([C@H]3[C@H](C[C@@]2([C@@]1(C)C(=O)OC)C)O)C | 0 | train |
| 170464694 | C1CCN(CC1)C2=NC(=NC3=C2N=C(N=C3N4CCCCC4)N(CCO)CCO)N(CCO)CCO | 0 | train |
| 170464693 | CC1=CC=C(C=C1)C2=CC(=NN2C3=CC=C(C=C3)S(=O)(=O)N)C(F)(F)F | 0 | validation |
| 170464692 | CC(=O)NC1=NN=C(S1)S(=O)(=O)N | 0 | test |
| 170464691 | CC(C)CN1C=NC2=C1C3=CC=CC=C3N=C2N | 0 | train |
| 170464690 | C1=C(C(=C(C(=C1Cl)Cl)CC2=C(C(=CC(=C2Cl)Cl)Cl)O)O)Cl | 1 | train |
| 170464689 | C(CN)C(O)(P(=O)(O)O)P(=O)(O)O | 0 | validation |
| 170464688 | CN(CC1=CN=C2C(=N1)C(=NC(=N2)N)N)C3=CC=C(C=C3)C(=O)N[C@@H](CCC(=O)O)C(=O)O | 0 | test |
| 170464687 | COC1=CC=C(C=C1)O | 0 | train |
| 170464686 | C(C(CO)(CO)N)O | 0 | train |
| 170464685 | CC(=O)OCC(=O)[C@]1(CC[C@@H]2[C@@]1(CC(=O)[C@H]3[C@H]2CCC4=CC(=O)CC[C@]34C)C)O | 0 | validation |
| 170464684 | CC[C@@H](CO)NC(=O)[C@H]1CN([C@@H]2CC3=CN(C4=CC=CC(=C34)C2=C1)C)C | 0 | test |
| 170464683 | C[C@]12CC[C@H]3[C@H]([C@@H]1CC[C@]2(C#C)O)CCC4=CC(=O)CC[C@H]34 | 0 | train |

| | | | |
|---|---|---|---|
| 170464682 | CC(C)NC[C@H](COC1=CC=CC2=CC=CC=C21)O | 0 | train |
| 170464681 | C1CC(CCC1C2=CC=C(C=C2)Cl)C3=C(C4=CC=CC=C4C(=O)C3=O)O | 0 | validation |
| 170464680 | C(CN(CC(=O)[O-])CC(=O)[O-])N(CC(=O)[O-])CC(=O)[O-].[Na+].[Fe+3] | 0 | test |
| 170464679 | CC(C)NCC(C1=CC(=C(C=C1)O)O)O | 0 | train |
| 170464678 | CC[N+](CC)(CC1=CC=CC=C1)CC(=O)NC2=C(C=CC=C2C)C.C1=CC=C(C=C1)C(=O)[O-] | 0 | train |
| 170464677 | C1=CC=C(C=C1)N2C(=O)C(C(=O)N2C3=CC=CC=C3)CCS(=O)C4=CC=CC=C4 | 0 | validation |
| 170464676 | C[C@@H]1[C@@H]2[C@H](C(=O)N2C(=C1S[C@H]3C[C@H](NC3)C(=O)N(C)C)C(=O)O)[C@@H](C)O | 0 | test |
| 170464675 | CC(C)CC1CN2CCC3=CC(=C(C=C3C2CC1=O)OC)OC | 0 | train |
| 170464674 | COC1=C(C=C(C=C1)C2=CC3=C(C=C2)C=C(C=C3)C(=O)O)C45CC6CC(C4)CC(C6)C5 | 0 | train |
| 170464673 | CC1=CC=CC=C1S(=O)(=O)NC(=O)C2=CC(=C(C=C2)CC3=CN(C4=C3C=C(C=C4)NC(=O)OC5CCCC5)C)OC | 0 | validation |
| 170464672 | COC1=CC(=C(C=C1)OC)C(CNC(=O)CN)O.Cl | 0 | test |
| 170464671 | CCOC(=O)C1=CN=C(C=C1)C#CC2=CC3=C(C=C2)SCCC3(C)C | 0 | train |
| 170464670 | CCCCC1=NC2(CCCC2)C(=O)N1CC3=CC=C(C=C3)C4=CC=CC=C4C5=NNN=N5 | 0 | train |
| 170464669 | CC(C)(C1=CC=C(C=C1)C(CCCN2CCC(CC2)C(C3=CC=CC=C3)(C4=CC=CC=C4)O)O)C(=O)O.Cl | 0 | validation |
| 170464668 | C[C@@H]1[C@@H](C(=O)N[C@@H](C(=O)N2CCC[C@H]2C(=O)N(CC(=O)N([C@H](C(=O)O1)C(C)C)C)C(C)C)NC(=O)C3=C4C(=C(C=C3)C)OC5=C(C(=O)C(=C(C5=N4)C(=O)N[C@H]6[C@H](OC(=O)[C@@H](N(C(=O)CN(C(=O)[C@@H]7CCCN7C(=O)[C@H](NC6=O)C(C)C)C)C(C)C)C)N)C | 0 | test |
| 170464667 | CCNC(=O)N(CCCN(C)C)C(=O)[C@@H]1C[C@H]2[C@@H](CC3=CNC4=CC=CC2=C34)N(C1)CC=C | 0 | train |
| 170464666 | C[C@@H]1C[C@H]2[C@@H]3CCC4=CC(=O)C=C[C@@]4([C@]3([C@H](C[C@@]2([C@]1(C(=O)CCl)OC(=O)C5=CC=CO5)C)O)Cl)C | 0 | train |
| 170464665 | CC1NC2=CC(=C(C=C2C(=O)N1C3=CC=CC=C3C)S(=O)(=O)N)Cl | 0 | validation |
| 170464664 | CC(C)NC1=C(N=CC=C1)N2CCN(CC2)C(=O)C3=CC4=C(N3)C=CC(=C4)NS(=O)(=O)C | 0 | test |
| 170464663 | CCCCCCCCCCC[C@@H](C[C@H]1[C@@H](C(=O)O1)CCCCCC)OC(=O)C(CC(C)C)NC=O | 0 | train |
| 170464662 | CC(=O)N[C@@H]1[C@H](C=C(O[C@H]1[C@@H]([C@@H](CO)O)O)C(=O)O)N=C(N)N | 0 | train |
| 170464661 | C1=CC(=CC=C1O)O | 1 | validation |
| 170464660 | C[C@]12C[C@@H]([C@]3([C@H]([C@@H]1C[C@@H]4[C@]2(OC(O4)(C)C)C(=O)CO)CCC5=CC(=O)C=C[C@@]53C)F)O | 0 | test |
| 170464659 | C1CN2C(=CC=C2C(=O)C3=CC=CC=C3)[C@@H]1C(=O)O.C(C(CO)(CO)N)O | 1 | train |
| 170464658 | CC1=C(N2[C@@H]([C@@H](C2=O)NC(=O)[C@@H](C3=CC=C(C=C3)O)N)SC1)C(=O)O | 0 | train |
| 170464657 | C1=NC(=NN1[C@H]2[C@@H]([C@@H]([C@H](O2)CO)O)O)C(=O)N | 0 | validation |
| 170464656 | CN(C)CCN(CC1=CC=C(C=C1)OC)C2=CC=CC=N2 | 0 | test |
| 170464655 | CN(C)NN=C1C(=NC=N1)C(=O)N | 0 | train |
| 170464654 | CN(C)C[C@H]1CCCC[C@@]1(C2=CC(=CC=C2)OC)O | 0 | train |
| 170464653 | C1=CC(=CC=C1N)S(=O)(=O)N | 0 | validation |
| 170464652 | CC(C)NCC(COC1=CC=C(C=C1)CCOC)O.CC(C)NCC(COC1=CC=C(C=C1)CCOC)O.[C@@H]([C@H](C(=O)O)O)(C(=O)O)O | 0 | test |
| 170464651 | CC1=C(N=CN1)CSCCNC(=NC)NC#N | 0 | train |
| 170464650 | CCN(CC)C(C)C(=O)C1=CC=CC=C1.Cl | 0 | train |
| 170464649 | CC1=C(C=CN=C1CS(=O)C2=NC3=CC=CC=C3N2)OCC(F)(F)F | 1 | validation |
| 170464648 | CC¥1=C(C2=C(/C1=C¥C3=CC=C(C=C3)S(=O)C)C=CC(=C2)F)CC(=O)O | 0 | test |
| 170464647 | CC(C)(CO)[C@H](C(=O)NCCCO)O | 0 | train |
| 170464646 | C[C@@H](C(=O)NC1=C(C(=C(C(=C1I)C(=O)NC(CO)CO)I)C(=O)NC(CO)CO)I)O | 0 | train |
| 170464645 | C(C(F)(F)F)(Cl)Br | 0 | validation |
| 170464644 | C[C@H](CS)C(=O)N1CCC[C@H]1C(=O)O | 0 | test |
| 170464643 | C[C@](CC1=CC(=C(C=C1)O)O)(C(=O)O)N.C[C@](CC1=CC(=C(C=C1)O)O)(C(=O)O)N.O.O.O | 0 | train |
| 170464642 | C1=CC=C(C(=C1)CC(=O)[O-])NC2=C(C=CC=C2Cl)Cl.[Na+] | 0 | train |
| 170464641 | C1COC(=O)N1/N=C/C2=CC=C(O2)[N+](=O)[O-] | 1 | validation |
| 170464640 | C1=NC2=C(N1COCCO)NC(=NC2=O)N | 0 | test |
| 170464639 | CCN(CC)C1=CC=C(C=C1)C(=C2C=CC(=[N+](CC)CC)C=C2)C3=C(C=CC(=C3)S(=O)(=O)[O-])S(=O)(=O)[O-].[Na+] | 1 | train |
| 170464638 | CC(=O)NC1=C(C(=C(C(=C1I)C(=O)O)I)NC(=O)C)I.O.O | 0 | train |
| 170464637 | CC1=CC(=NO1)NS(=O)(=O)C2=CC=C(C=C2)N | 0 | validation |
| 170464636 | CC1=C(ON=C1C)NS(=O)(=O)C2=CC=C(C=C2)N | 0 | test |
| 170464635 | C[C@]12CCC(=O)C=C1CC[C@@H]3[C@@H]2[C@H](C[C@]4([C@H]3CC[C@@]4(C(=O)CO)O)C)O | 0 | train |
| 170464634 | CC1=NC=C(C(=C1O)CO)CO | 0 | train |

| | | | |
|---|---|---|---|
| 170464633 | COC1=CC(=CC(=C1OC)OC)CC2=CN=C(N=C2N)N | 0 | validation |
| 170464632 | CC(=O)OCC(=O)[C@]1(CC[C@@H]2[C@@]1(C[C@@H]([C@H]3[C@H]2CCC4=CC(=O)C=C[C@]34C)O)C)O | 0 | test |
| 170464631 | C[C@]12CCC(=O)C=C1CC[C@@H]3[C@@H]2CC[C@]4([C@H]3CC[C@]4(C)O)C | 0 | train |
| 170464630 | C[C@]12C[C@@H]([C@]3([C@H]([C@@H]1C[C@@H]4[C@]2(OC(O4)(C)C)C(=O)CO)C[C@@H](C5=CC(=O)C=C[C@@]53C)F)F)O | 0 | train |
| 170464629 | C[C@H]1C[C@H]2[C@@H]3CC[C@@]([C@]3(C[C@@H]([C@@]2([C@@]4(C1=CC(=O)C=C4)C)F)O)C)(C(=O)C)O | 0 | validation |
| 170464628 | C[C@]12CC[C@H]3[C@H]([C@@H]1CCC2=O)CC(=C)C4=CC(=O)C=C[C@]34C | 1 | test |
| 170464627 | C[C@H]1[C@H]([C@H](C[C@@H](O1)O[C@H]2C[C@@](CC3=C(C4=C(C(=C23)O)C(=O)C5=CC=CC=C5C4=O)O)(C(=O)C)O)N)O.Cl | 0 | train |
| 170464626 | CN1C(=O)CN=C(C2=C1C=CC(=C2)Cl)C3=CC=CC=C3 | 0 | train |
| 170464625 | C[N+](C)(C)CCOC(=O)CCC(=O)OCC[N+](C)(C)C.[Cl-].[Cl-] | 0 | validation |
| 170464624 | C1CNP(=O)(OC1)N(CCCl)CCCl.O | 0 | test |
| 170464623 | C[N+]1([C@@H]2CC(C[C@H]1[C@H]3[C@@H]2O3)OC(=O)[C@H](CO)C4=CC=CC=C4)C.[Br-] | 0 | train |
| 170464622 | C[C@H]1C[C@@H]2[C@H](CC[C@]3([C@H]2CC[C@@]3(C(=O)C)OC(=O)C)C)[C@@]4(C1=CC(=O)CC4)C | 0 | train |
| 144214049 | CC(C)(C1=CC=C(C=C1)O)C2=CC=C(C=C2)O | 1 | validation |
| 144214048 | CO/C=C(¥C1=CC=CC=C1OC2=NC=NC(=C2)OC3=CC=CC=C3C#N)/C(=O)OC | 0 | test |
| 144214047 | CCCCOCCOCCOCC1=CC2=C(C=C1CCC)OCO2 | 1 | train |
| 144214046 | CC(C)OC1=CC=CC(=C1)NC(=O)C2=CC=CC=C2C(F)(F)F | 1 | train |
| 144214045 | CCCCC(CC)COC(=O)C1=CC=CC=C1C(=O)OCC(CC)CCCC | 0 | validation |
| 144214044 | C(C(C(C(C(F)(F)S(=O)(=O)O)(F)F)(F)F)(F)F)(C(C(C(F)(F)F)(F)F)(F)F)(F)F | 0 | test |
| 144214043 | CCCSP(=O)(OCC)SCCC | 1 | train |
| 144214042 | CCOP(=O)(NC(C)C)OC1=CC(=C(C=C1)SC)C | 1 | train |
| 144214041 | C1=CC(=C(C=C1Cl)Cl)OCCCC(=O)O | 0 | validation |
| 144214040 | C1=CC(=C(C=C1Cl)O)OC2=C(C=C(C=C2)Cl)Cl | 0 | test |
| 144214039 | CCCN(CCC)C1=C(C=C(C=C1[N+](=O)[O-])S(=O)(=O)N)[N+](=O)[O-] | 1 | train |
| 144214038 | CC(C)(C)C(C(N1C=NC=N1)OC2=CC=C(C=C2)Cl)O | 0 | train |
| 144214037 | CCCCC(CC)COC(=O)C1=CC=CC=C1C(=O)O | 0 | validation |
| 144214036 | COC1=CC=C(C=C1)C(C2=CC=C(C=C2)OC)C(Cl)(Cl)Cl | 1 | test |
| 144214035 | CC1=C(C(=O)CC1OC(=O)C2C(C2(C)C)C=C(C)C)CC=C | 1 | train |
| 144214034 | C1=C(C(=NC(=C1Cl)Cl)OCC(=O)O)Cl | 0 | train |
| 144214033 | CCN(CC)C(=S)SSC(=S)N(CC)CC | 1 | validation |
| 144214032 | COC(=O)C1=C(C(=C(C(=C1Cl)Cl)C(=O)OC)Cl)Cl | 1 | test |
| 144214031 | COC1=CC(=C(C=C1C=O)OC)OC | 0 | train |
| 144214030 | CC1=C(C=C(C(=O)N1)C#N)C2=CC=NC=C2 | 0 | train |
| 144214029 | CC1=CC(=CC(=C1O)[N+](=O)[O-])[N+](=O)[O-] | 0 | validation |
| 144214028 | Cl[In](Cl)Cl | 0 | test |
| 144214027 | CC(C)OC(=O)C1=CC(=CC(=C1)[N+](=O)[O-])C(=O)OC(C)C | 0 | train |
| 144214026 | C1CN1P(=S)(N2CC2)N3CC3 | 0 | train |
| 144214025 | CCCCCCCCCC(CC)C1=CC=C(C=C1)S(=O)(=O)O | 0 | validation |
| 144214024 | C1=CC2=C3C(=C1)C=CC4=CC=CC(=C43)C=C2 | 1 | test |
| 144214023 | C1=CC(=CC=C1C(=O)O)Cl | 0 | train |
| 144214022 | C1=CC(=CC=C1[C@H]([C@@H](CO)NC(=O)C(Cl)Cl)O)[N+](=O)[O-] | 0 | train |
| 144214021 | C1=CC=C(C=C1)C(=O)NO | 0 | validation |
| 144214020 | C1=CC=C(C=C1)C2=CC(=O)C3=CC=CC=C3O2 | 1 | test |
| 144214019 | C[C@]12CCC(=O)C=C1CC[C@@H]3[C@@H]2CC[C@]4([C@H]3CC[C@]4(C)O)C | 0 | train |
| 144214018 | CCCCOC(=O)C1=CC=CC=C1C(=O)OCC2=CC=CC=C2 | 1 | train |
| 144214017 | C1=CC=C2C(=C1)C=C(C=C2[N+](=O)[O-])[N+](=O)[O-] | 1 | validation |
| 144214016 | C1=CC=C2C(=C1)C(=CN2)CO | 1 | test |
| 144214015 | C1=CC=C2C(=C1)C=CO2 | 0 | train |
| 144214014 | CN1C(=O)C2=CC=CC=C2C1=O | 0 | train |
| 144214013 | CCCC(=O)C1=CC(=C(C=C1O)O)O | 0 | validation |
| 144214012 | C(C(C(F)(F)S(=O)(=O)[O-])(F)F)(C(F)(F)F)(F)F.[K+] | 0 | test |
| 144214011 | CS(=O)(=O)C1=CC(=C(C=C1)C(=O)C2C(=O)CCCC2=O)[N+](=O)[O-] | 0 | train |
| 144214010 | COC1=NN(C(=O)S1)CSP(=S)(OC)OC | 1 | train |
| 144214009 | CCOC(=O)CC(C(=O)OCC)SP(=S)(OC)OC | 1 | validation |
| 144214008 | CC1(CCC(=CC2=CC=C(C=C2)Cl)C1(CN3C=NC=N3)O)C | 0 | test |
| 144214007 | C1=CC=C(C=C1)CC2=C(C=CC(=C2)Cl)O | 1 | train |
| 144214006 | C1=CC=C(C(=C1)C2=CC=C(C=C2)Cl)NC(=O)C3=C(N=CC=C3)Cl | 1 | train |

| | | | |
|---|---|---|---|
| 144214005 | C1=CC2=C(C=C1[N+](=O)[O-])SC(=N2)N | 1 | validation |
| 144214004 | C1=CC=C2C=C3C(=CC2=C1)C(=NC3=N)N | 1 | test |
| 144214003 | C(CO)CBr | 0 | train |
| 144214002 | C1=CC=C(C=C1)NC(=NC2=CC=CC=C2)N | 0 | train |
| 144214001 | C1=CC2=C(C=CC=C2N)C(=C1)N | 1 | validation |
| 144214000 | C1=CC=C(C=C1)C2=CC=CC=C2Br | 0 | test |
| 144213999 | C=CC(=O)NCNC(=O)C=C | 0 | train |
| 144213998 | C1=CC(=C(C=C1Cl)N)N | 0 | train |
| 144213997 | C1=CC(=C(C=C1[N+](=O)[O-])O)N | 1 | validation |
| 144213996 | C1=C(OC(=C1)[N+](=O)[O-])/C=N/NC(=O)N | 1 | test |
| 144213995 | CC1=C(C(=CC=C1)[N+](=O)[O-])[N+](=O)[O-] | 1 | train |
| 144213994 | CC1=C(C=C(C=C1)N)O | 1 | train |
| 144213993 | C1=CC(=CC(=C1)[N+](=O)[O-])[N+](=O)[O-] | 0 | validation |
| 144213992 | C1=CC(=CC=C1N)[N+](=O)[O-] | 0 | test |
| 144213991 | C1=CC(=CC=C1N)Cl | 0 | train |
| 144213990 | C1[C@@H]2[C@H]3[C@@H]([C@H]1[C@H]4[C@@H]2O4)[C@]5(C(=C([C@@]3(C5(Cl)Cl)Cl)Cl)Cl)Cl | 1 | train |
| 144213989 | CCOCCOC(=O)C | 0 | validation |
| 144213988 | C1=CC(=C(C=C1N)[N+](=O)[O-])O | 1 | test |
| 144213987 | C1=CC(=C(C=C1N)[N+](=O)[O-])N | 1 | train |
| 144213986 | C1=CC=C2C(=C1)C=C3C=CC=CC3=C2[N+](=O)[O-] | 1 | train |
| 144213985 | CN1C=NC(=C1SC2=NC=NC3=C2NC=N3)[N+](=O)[O-] | 0 | validation |
| 144213984 | C1=CC=C(C=C1)OC(=O)C2=CC(=CC=C2)C(=O)OC3=CC=CC=C3 | 1 | test |
| 144213983 | C1=CC=C(C=C1)C2=NC3=CC=CC=C3C(=C2)C(=O)O | 1 | train |
| 144213982 | C1(=C(C(=C(C(=C1Cl)Cl)Cl)Cl)Cl)N | 0 | train |
| 144213981 | C(CO)N | 0 | validation |
| 144213980 | C1=CC=C(C=C1)N=NC2=CC=C(C=C2)N | 1 | test |
| 144213979 | CC(C)C(C)(C)C(C)(C)C(C)(C)S | 0 | train |
| 144213978 | C1C=CC2C1C3CC2C=C3 | 0 | train |
| 144213977 | C1=CC=C2C(=C1)NC(=S)S2 | 1 | validation |
| 144213976 | CCCCCCCCC1=CC=C(C=C1)O | 1 | test |
| 144213975 | C1=CC=C(C(=C1)N)N | 1 | train |
| 144213974 | C1=C(C(=C(C(=C1Cl)Cl)[N+](=O)[O-])Cl)Cl | 0 | train |
| 144213973 | CCCOC(=O)C1=CC=C(C=C1)O | 1 | validation |
| 144213972 | CC(C)(C)CC(C)(C)C1=CC=C(C=C1)O | 0 | test |
| 144213971 | C1=CC=C2C(=C1)NC3=CC=CC=C3S2 | 1 | train |
| 144213970 | COCCOCCO | 0 | train |
| 144213969 | C1=CC(=CC=C1S(=O)(=O)C2=CC=C(C=C2)Cl)Cl | 1 | validation |
| 144213968 | C1=CC=C2C(=C1)C=CC=C2CC(=O)O | 0 | test |
| 144213967 | C1=CC2=C(C(=C1)O)N=CC=C2 | 1 | train |
| 144213966 | C1=CC(=CC=C1N)SC2=CC=C(C=C2)N | 1 | train |
| 144213965 | COC1=CC=CC=C1O | 0 | validation |
| 144213964 | CC(C)(C)C1=CC(=C(C=C1)O)O | 1 | test |
| 144213963 | CN(C)C1=CC=C(C=C1)N=NS(=O)(=O)[O-].[Na+] | 1 | train |
| 144213962 | CCC(=C1C(=O)CC(CC1=O)C2=C(C=C(C=C2C)C)C)NOCC | 0 | train |
| 144213961 | CC(C)C(=O)NC1=CC(=C(C=C1)[N+](=O)[O-])C(F)(F)F)O | 1 | validation |
| 144213960 | CC[C@]12CC[C@H]3[C@H]([C@@H]1CC[C@]2(C#C)O)CCC4=CC(=O)CC[C@H]34 | 0 | test |
| 144213959 | CCC(=C(C1=CC=C(C=C1)O)C2=CC=C(C=C2)OCCN(C)C)C3=CC=CC=C3 | 1 | train |
| 144213958 | C[C@]12CC[C@H]3[C@H]([C@@H]1CC[C@@H]2O)[C@@H](CC4=C3C=CC(=C4)O)CCCCCCCCCS(=O)CCCC(C(F)(F)F)(F)F | 0 | train |
| 144213957 | C[C@]12CCC(=O)C[C@@H]1CC[C@@H]3[C@@H]2CC[C@]4([C@H]3CC[C@@H]4O)C | 0 | validation |
| 144213956 | C[C@]12C=CC3=C4CCC(=O)C=C4CC[C@H]3[C@@H]1CC[C@@H]2O | 1 | test |
| 144213955 | C1CCC(CC1)C(=O)C2=CC=CC=C2 | 0 | train |
| 144213954 | C[C@H]1C[C@@H](C(=O)[C@@H](C1)[C@@H](CC2CC(=O)NC(=O)C2)O)C | 0 | train |
| 144213953 | C[C@]12CC[C@H]3[C@H]([C@@H]1CCC2=O)CCC4=C3C=CC(=C4)O | 1 | validation |
| 144213952 | C1=CC(=CC=C1C2=COC3=C(C2=O)C=CC(=C3)O)O | 1 | test |
| 144213951 | CCCCCCCC[Si](OCC)(OCC)OCC | 0 | train |
| 144213950 | CCCCCCCCCC1=CC=C(C=C1)O | 1 | train |
| 144213949 | C1=CC=C(C(=C1)C(C2=CC=C(C=C2)O)C3=CC=C(C=C3)O)C(=O)O | 1 | validation |
| 144213948 | C[C@]12CC[C@H]3[C@H]([C@@H]1CC[C@H]2O)CCC4=C3C=CC(=C4)O | 1 | test |
| 144213947 | C[C@]12CCC(=O)C=C1CC[C@@H]3[C@@H]2[C@H](C[C@]4([C@H]3CC[C@@H]4C(=O)CO)C)O | 0 | train |

| | | | |
|---|---|---|---|
| 144213946 | C1=CC(=CC=C1C(C2=CC=C(C=C2)O)(C(F)(F)F)C(F)(F)F)O | 1 | train |
| 144213945 | CC12CC1(C(=O)N(C2=O)C3=CC(=CC(=C3)Cl)Cl)C | 0 | validation |
| 144213944 | C1=CC(=C(C=C1O)O)C(=O)C2=C(C=C(C=C2)O)O | 1 | test |
| 144213943 | CCCCCCCCCCCCCCCCCC(=O)OCCCCCCCCCCCCCCC(C)C | 0 | train |
| 144213942 | CC(C)C1=CC=C(C=C1)S(=O)(=O)[O-].[Na+] | 0 | train |
| 144213941 | CC(=O)CC(C)(C)C1CCCCC1 | 0 | validation |
| 144213940 | COC1=CC=CC=C1/C=C/C=O | 0 | test |
| 144213939 | CC1CCCC(=O)C1=O | 0 | train |
| 144213938 | CC1=CC=C(C=C1)C(C)NC(=O)[C@H](C(C)C)NC(=O)OC(C)C | 0 | train |
| 144213937 | C1CCC(CC1)CCCO | 0 | validation |
| 144213936 | CN(CCN(C1=CC=CC=N1)CC2=CC=CS2)C.CN(CCN(C1=CC=CC=N1)CC2=CC=CS2)C.C(=C/C(=O)O)¥C(=O)O.C(=C/C(=O)O)¥C(=O)O.C(=C/C(=O)O)¥C(=O)O | 0 | test |
| 144213935 | CC(C)CCOCC1=CC=CC=C1 | 0 | train |
| 144213934 | CCC(=O)C1CC(CC=C1C)C(C)C | 0 | train |
| 144213933 | CCCCCCCCC1=CC=C(C=C1)S(=O)(=O)[O-].[Na+] | 0 | validation |
| 144213932 | CC/C=C¥CCOC(=O)CC | 0 | test |
| 144213931 | CC(=CCC/C(=C/C/C=C(¥C)/C=C)/C)C | 0 | train |
| 144213930 | CCCC1CCOC(S1)C | 0 | train |
| 144213929 | CC/C=C¥CCCCCO | 0 | validation |
| 144213928 | CC[C@]12CC[C@H]3[C@H]([C@@H]1CCC2(C#C)O)CCC4=CC(=O)CC[C@H]34 | 1 | test |
| 144213927 | CCCCCCCCC(=O)OCC(C)OC(=O)CCCCCCCC | 0 | train |
| 144213926 | C(CCCCS)CCCS | 0 | train |
| 144213925 | CC(C)CC(=O)OC(C)(CCC=C(C)C)C=C | 0 | validation |
| 144213924 | C1CCC(CC1)(C#N)N=NC2(CCCCC2)C#N | 0 | test |
| 144213923 | C(CN(CCO)CCO)C#N | 0 | train |
| 144213922 | CC1C(=NC(O1)C)C | 0 | train |
| 144213921 | CC(C(=O)O)O | 0 | validation |
| 144213920 | CCCCCCCCCCCC | 0 | test |
| 144213919 | CC1=CC=C(C=C1)S | 0 | train |
| 144213918 | C1CC(N2C=NC=C2C1)C3=CC=C(C=C3)C#N.Cl | 0 | train |
| 144213917 | CN(C)CCC=C1C2=CC=CC=C2CCC3=CC=CC=C31.Cl | 1 | validation |
| 144213916 | CCCCN1C=C[N+](=C1)C.[Cl-] | 0 | test |
| 144213915 | C/C(=C¥C1=CC=CC=C1)/[N+](=O)[O-] | 1 | train |
| 144213914 | CC(=C)CCl | 0 | train |
| 144213913 | CC(C)CC(C)(C1=CC=C(C=C1)O)C2=CC=C(C=C2)O | 1 | validation |
| 144213912 | C=COCC1CCC(CC1)CO | 0 | test |
| 144213911 | CC(=CC(=O)C)C | 0 | train |
| 144213910 | CC/C(=C¥C(=N/O)¥C(=O)N)/C(C)[N+](=O)[O-] | 0 | train |
| 144213909 | C1=C2C(=C(C(=C1Br)O)Br)OC3=C(C(=C(C=C3C24C5=C(C(=C(C(=C5Cl)Cl)Cl)Cl)C(=O)O4)Br)O)Br | 0 | validation |
| 144213908 | CCCCCCCCC=O | 0 | test |
| 144213907 | CC(=O)C1=CC=C(C=C1)O | 0 | train |
| 144213906 | CC1=C(C=CC(=C1)F)C2=CC(=NC=C2N(C)C(=O)C(C)(C)C3=CC(=CC(=C3)C(F)(F)F)C(F)(F)F)N4CC[C@H]([C@H]4CO)O | 0 | train |
| 144213905 | CC(CCO)CC(C)(C)C | 0 | validation |
| 144213904 | C1=CC=C(C(=C1)CC2=CC=CC=C2O)O | 1 | test |
| 144213903 | CCCCCC(=CC1=CC=CC=C1)C=O | 0 | train |
| 144213902 | CC(C)C[C@H]1C(=O)N2CCC[C@H]2[C@]3(N1C(=O)[C@](O3)(C(C)C)NC(=O)[C@H]4CN([C@@H]5CC6=C(NC7=CC=CC(=C67)C5=C4)Br)C)O.CS(=O)(=O)O | 1 | train |
| 144213901 | C1CCC(CC1)SN2C(=O)C3=CC=CC=C3C2=O | 0 | validation |
| 144213900 | C1=CC=C(C=C1)C(=O)OC2=CC=CC=C2 | 0 | test |
| 144213899 | CC1=C(C=CO1)SSC | 0 | train |
| 144213898 | CS(=O)(=O)C1=C(C=C(C=C1)[C@@H](C[C@H]2CCC(=O)C2)C(=O)NC3=NC=CN=C3)Cl | 0 | train |
| 144213897 | [O-]S(=O)(=O)OOS(=O)(=O)[O-].[Na+].[Na+] | 0 | validation |
| 144213896 | CC1=CC[C@H]2C[C@@H]1C2(C)C | 0 | test |
| 144213895 | COC(=O)OC | 0 | train |
| 144213894 | C1=CC=C(C=C1)OC(=O)C2=CC=C(C=C2)O | 0 | train |
| 144213893 | CCCCCC1C(CCC1=O)CC(=O)OC | 0 | validation |
| 144213892 | C1=CC=C2C(=C1)C=CC3=CC4=C(C=CC5=CC=CC=C54)C=C32 | 1 | test |
| 144213891 | C1=CSC(=C1)/C=C/S(=O)(=O)NC(=O)C2=C(C=C(C=C2)F)Br | 0 | train |
| 144213890 | CCCN([C@@H]1CCC2=C(C1)C=C(C=C2)OC)C3CCN(CC3)C(=O)C4CCNCC4 | 0 | train |
| 144213889 | CCC(C)C(=O)O | 0 | validation |

| | | | |
|---|---|---|---|
| 144213888 | CCCC[Sn](Cl)(Cl)Cl | 0 | test |
| 144213887 | CC(C)(C1=CC(=C(C(=C1)Cl)O)Cl)C2=CC(=C(C(=C2)Cl)O)Cl | 0 | train |
| 144213886 | C1=CC(=N)C=CC1=C(C2=CC=C(C=C2)N)C3=CC=C(C=C3)N.Cl | 0 | train |
| 144213885 | CCC(C)OC(=O)C1=CC=C(C=C1)O | 1 | validation |
| 144213884 | CC(=C)C(=O)O[C@@H]1C[C@H]2CC[C@@]1(C2(C)C)C | 0 | test |
| 144213883 | CC1=CC(=C(C=C1)O)N2N=C3C=CC=CC3=N2 | 1 | train |
| 144213882 | CC(C)(C)C1=CC=C(C=C1)OC(=O)C2=CC=CC=C2O | 1 | train |
| 144213881 | C1CN(CCC1C=C(C2=CC=CC=C2)C3=CC=CC=C3)C(=O)CCCCCCCC4=CN=CC=C4 | 0 | validation |
| 144213880 | C1=C(SC(=N1)N)[N+](=O)[O-] | 0 | test |
| 144213879 | CCCCC(CC)COC(=O)C1=CC=C(C=C1)N(C)C | 1 | train |
| 144213878 | C(C(CO)(CO)CO)O | 0 | train |
| 144213877 | C1=CC(=CC=C1C#N)C(C2=CC=C(C=C2)C#N)N3C=NC=N3 | 0 | validation |
| 144213876 | C1=CC=C(C=C1)OC2=CC=CC(=C2)CO | 0 | test |
| 144213875 | C1=CC=C2C(=C1)C(OS2(=O)=O)(C3=CC=C(C=C3)O)C4=CC=C(C=C4)O | 0 | train |
| 144213874 | C1=C(C=C(C=C1Cl)Cl)N | 0 | train |
| 144213873 | CC(C)(C#N)C1=CC(=CC(=C1)CN2C=NC=N2)C(C)(C)C#N | 0 | validation |
| 144213872 | C1=CC2=C(C=CC(=C2)S(=O)(=O)[O-])C=C1O.[K+] | 0 | test |
| 144213871 | CC(C)(C)C1=CC(=CC(=C1O)C(C)(C)C)CC2=CC(=C(C(=C2)C(C)(C)C)O)C(C)(C)C | 0 | train |
| 144213870 | COC1=CC2=C(C=CN=C2C=C1)[C@H]([C@@H]3CC4CCN3C[C@@H]4C=C)O.O.O.Cl | 0 | train |
| 144213869 | CC(C)(C)C(=O)C1=CC=CC=C1 | 0 | validation |
| 144213868 | C1CCC(C1)O | 0 | test |
| 144213867 | C1CCC(=O)C1 | 0 | train |
| 144213866 | CC(C)(C)C1=CC=C(C=C1)C2=CC=C(C=C2)C(C)(C)C | 0 | train |
| 144213865 | C[C@](CS(=O)(=O)C1=CC=C(C=C1)F)(C(=O)NC2=CC(=C(C=C2)C#N)C(F)(F)F)O | 0 | validation |
| 144213864 | C1=CC(=C(C(=C1C(=O)C2=CC(=C(C(=C2)O)O)O)O)O)O | 0 | test |
| 144213863 | CC(=O)O[C@@H]1C[C@H]2CC[C@@]1(C2(C)C)C | 0 | train |
| 144213862 | CC(C)(C)C1=CC=C(C=C1)S | 1 | train |
| 144213861 | CCCCCCCCCCCCCC[P+](CCCC)(CCCC)CCCC.[Cl-] | 1 | validation |
| 144213860 | C1[C@@H]([C@H](C(=O)O1)CC2=CC(=CC=C2)O)CC3=CC(=CC=C3)O | 0 | test |
| 144213859 | CCCCC1=NC2=C(N1CC3=CC=C(C=C3)C4=CC=CC=C4C5=NN=N[N-]5)N=CC=C2.[Na+] | 0 | train |
| 144213858 | CC(COC)N | 0 | train |
| 144213857 | CCC1=CC=CC(=C1N)C | 0 | validation |
| 144213856 | C1=C(OC(=C1)C=O)CO | 0 | test |
| 144213855 | CCCCOCCOC(=O)C1=CC=CC=C1C(=O)OCCOCCCC | 0 | train |
| 144213854 | CCC1=CC(=CC(=C1N)CC)CC2=CC(=C(C(=C2)CC)N)CC | 1 | train |
| 144213853 | CCN1C2=C(C=C(C=C2)N)C3=CC=CC=C31 | 1 | validation |
| 144213852 | CCCCOCCOCCSC#N | 0 | test |
| 144213851 | CCCCCCCC/C=C¥CCCCCCCC(=O)OC(CO)CO | 0 | train |
| 144213850 | C1=CC(=C(C=C1OC2=C(C=C(C=C2I)CC(=O)O)I)I)O | 1 | train |
| 144213849 | CCCCCCC(=O)C1=CC=CC=C1 | 1 | validation |
| 144213848 | CCCCCC1=CC=CO1 | 0 | test |
| 144213847 | C1=CC2=C(C=CC(=C2)S(=O)(=O)[O-])C=C1O.[Na+] | 0 | train |
| 144213846 | CC(C)(Cl)Cl | 0 | train |
| 144213845 | C1=CC=C(C=C1)NCC(=O)[O-].[K+] | 0 | validation |
| 144213844 | C1=CC=C(C=C1)/C=C/S(=O)(=O)[N-]C(=O)C2=C(C=C(C=C2)F)Cl.[Na+] | 0 | test |
| 144213843 | C1=CC2=C(C(=C1)O)N=CC=C2.C1=CC2=C(C(=C1)O)N=CC=C2.OS(=O)(=O)O | 0 | train |
| 144213842 | C([C@@H]([C@@H]1C(=C(C(=O)O1)[O-])O)O)O.C([C@@H]([C@@H]1C(=C(C(=O)O1)[O-])O)O)O.O.O.[Ca+2] | 0 | train |
| 144213841 | CCC(O)OCC(C)OC(CC)O | 0 | validation |
| 144213840 | C(Cl)(Cl)(Cl)Br | 0 | test |
| 144213839 | CC(CCl)OP(=O)(OC(C)CCl)OC(C)CCl | 1 | train |
| 144213838 | CC1=C(C=CC(=C1)OC)N | 0 | train |
| 144213837 | CCCCCCC(C)(C)S | 0 | validation |
| 144213836 | CCCCC1CCC(CC1)O | 0 | test |
| 144213835 | C1=CC=C(C=C1)C2=CC(=CC=C2)C3=CC=CC=C3 | 1 | train |
| 144213834 | CCC(COC(=O)C=C)(COC(=O)C=C)COC(=O)C=C | 1 | train |
| 144213833 | CC(C)CCCCCCCOP(OCCCCCCCC(C)C)OC1=CC=CC=C1 | 0 | validation |
| 144213832 | CC(=O)[C@H]1[C@@H](C[C@@H]2[C@@]1(CC[C@H]3[C@H]2CC=C4[C@@]3(CC[C@@H](C4)O)C)C)C#N | 0 | test |
| 144213831 | C1CC(C1)C(=O)C2=CC=CC=C2 | 0 | train |
| 144213830 | CC(C)(CS(=O)(=O)O)NC(=O)C=C | 0 | train |
| 144213829 | [NH4+].[N+](=O)([O-])[O-] | 0 | validation |

| | | | |
|---|---|---|---|
| 144213828 | CCCCCCCC/C=C¥CCCCCCCC(=O)OCC | 0 | test |
| 144213827 | CCCCCCCCCCCCCC(=O)OCC | 0 | train |
| 144213826 | CN1CCC(CC1)NC(=O)C2=CC(=C(C=C2)NC3=NC=C4C(=N3)N(CC(C(=O)N4C)(F)F)C5CCCC5)OC | 1 | train |
| 144213825 | CC1=C2C(=NC3=C(C=C(C=C3)[N+](=O)[O-])C(=N2)C4=CC=CC=C4Cl)NN1 | 0 | validation |
| 144213824 | COCC(=O)O | 0 | test |
| 144213823 | CC(=O)C1=NC=CS1 | 0 | train |
| 144213822 | C1=CC=C(C(=C1)CC2=CC=C(C=C2)O)O | 1 | train |
| 144213821 | CC1=C(C(=CC=C1)O)C | 0 | validation |
| 144213820 | CCCCC(CC)COC(=O)Cl | 0 | test |
| 144213819 | CC1=CC(=C(C=C1C)C)C | 0 | train |
| 144213818 | C[C@H]1CCCC(=O)CCC/C=C/C2=CC(=CC(=C2C(=O)O1)O)O | 0 | train |
| 144213817 | CC(C)C1=CC2=CC[C@@H]3[C@@]([C@H]2CC1)(CCC[C@@]3(C)C(=O)OC)C | 1 | validation |
| 144213816 | C1=CC=C2C(=C1)C(=O)OC23C4=C(C=C(C=C4)O)OC5=C3C=CC(=C5)O | 0 | test |
| 144213815 | C=CCO | 0 | train |
| 144213814 | CC1=CC=C(C=C1)S(=O)(=O)N | 0 | train |
| 144213813 | CC(=O)[O-].CC(=O)[O-].O.O.O.[Pb+2] | 0 | validation |
| 144213812 | CC1=C2CC[C@@H](C(=O)N2C3=CC=CC=C13)CC4=C(NC=N4)C.Cl | 0 | test |
| 144213811 | CCCC[N+]1=CC=CC=C1.[Cl-] | 0 | train |
| 144213810 | CC1=C(C=CC(=C1)OC)NC2=CC=CC=C2 | 1 | train |
| 144213809 | C1=CC=C(C=C1)C2=NC(C(=O)NC3=C2C=C(C=C3)Cl)O | 0 | validation |
| 144213808 | C1=CC=C(C=C1)CCOC(=O)C2=CC=CC=C2N | 1 | test |
| 144213807 | C1CCCN(CC1)CC2=CC=C(S2)C3=NC(=NO3)CCN4C(=O)C5=CC=CC=C5C4=O.C(=C/C(=O)O)¥C(=O)O | 0 | train |
| 144213806 | CC1(C2CCC(C2)(C1O)C)C | 0 | train |
| 144213805 | CCC1(CCC(=O)NC1=O)C2=CC=C(C=C2)N | 0 | validation |
| 144213804 | CC1=CC2C3CC(C2C1)C=C3C | 0 | test |
| 144213803 | CN1C(=NN(C1=O)C(=O)[N-]S(=O)(=O)C2=CC=CC=C2OC(F)(F)F)OC.[Na+] | 0 | train |
| 144213802 | C[C@]12CC[C@](C[C@H]1C3=CC(=O)[C@@H]4[C@]5(CC[C@@H](C([C@@H]5CC[C@]4([C@@]3(CC2)C)C)(C)C)O[C@@H]6[C@@H]([C@H]([C@@H]([C@H](O6)C(=O)O)O)O)O[C@H]7[C@@H]([C@H]([C@@H]([C@H](O7)C(=O)O)O)O)O)C)(C)C(=O)O | 0 | train |
| 144213801 | C1=C(C=C(C(=C1I)OC2=CC(=C(C(=C2)I)O)I)I)CC(=O)O | 0 | validation |
| 144213800 | CC(C)SCC1=CC=CO1 | 0 | test |
| 144213799 | CCCCCCOC1=CC=C(C=C1)N | 1 | train |
| 144213798 | C=CC(=O)OCCC(=O)O | 0 | train |
| 144213797 | C1=CC(=CC=C1C(=O)C2=C(C=C(C=C2)O)O)O | 1 | validation |
| 144213796 | CCCCCCCCCCCCOC(=O)C1=CC(=C(C(=C1)O)O)O | 0 | test |
| 144213795 | CCCCC(CC)COC(=O)CS | 0 | train |
| 144213794 | CC(=O)CC(=O)NC1=CC(=C(C=C1OC)Cl)OC | 1 | train |
| 144213793 | CCCC[P+](CCCC)(CCCC)CCCC.[Cl-] | 0 | validation |
| 144213792 | CC1CC2=C(CC1(C)C(=O)C)C(CCC2)(C)C | 0 | test |
| 144213791 | NS(=O)(=O)O | 0 | train |
| 144213790 | C[C@@H]1CC[C@@H]2[C@@]13C[C@H](C2(C)C)C(=C(C3)C(=O)C)C | 1 | train |
| 144213789 | CN1CCC2=CC=CC3=C2[C@H]1CC4=C3C(=C(C=C4)O)O.CN1CCC2=CC=CC3=C2[C@H]1CC4=C3C(=C(C=C4)O)O.O.Cl.Cl | 1 | validation |
| 144213788 | CC(=O)CC(C)(C)O | 0 | test |
| 144213787 | CCC(CC1=C(C(=C(C=C1I)I)N)I)C(=O)O | 1 | train |
| 144213786 | CCOC(=O)C1=CC=C(C=C1)C | 0 | train |
| 144213785 | CCCCCCCC/C=C¥CCCCCCCC(=O)OCCO | 0 | validation |
| 144213784 | CC(C)(C)C1=CSC(=N1)C2=CC3=C(O2)C=CC(=C3)OCC4=CC=CC=C4CC(=O)O | 1 | test |
| 144213783 | CCCCCC1=CC=C(C=C1)Cl | 0 | train |
| 144213782 | CCCCCCCC/C=C/CCCCCCCC(=O)OCCOCC(C1C(CC(O1)OCCO)OCCO)OCCO | 0 | train |
| 144213781 | CCCCCCOC1=CC=C(C=C1)O | 1 | validation |
| 144213780 | CCCCCCCO | 0 | test |
| 144213779 | COC1=C(C=C(C(=C1)O)C(=O)C2=CC=CC=C2)S(=O)(=O)O | 0 | train |
| 144213778 | CCC(C)(CCCC(C)C)O | 0 | train |
| 144213777 | CCC1=CC=CC=C1CC | 0 | validation |
| 144213776 | C1=CC=C(C=C1)C(=O)NC2=CC=CC=C2SSC3=CC=CC=C3NC(=O)C4=CC=CC=C4 | 0 | test |
| 144213775 | CCCCCCOC(=O)CCCCCCCC(=O)OCCCCCC | 0 | train |
| 144213774 | CC1CCC(C(C1)O)C(C)C | 0 | train |
| 144213773 | C=CCN(CC=C)C(=O)C(Cl)Cl | 0 | validation |
| 144213772 | CC(C)CCCCCCOC(=O)C1=CC=CC=C1C(=O)OCCCCCCC(C)C | 0 | test |

| | | | |
|---|---|---|---|
| 144213771 | CC1=CC=CC=C1C(=O)OC | 0 | train |
| 144213770 | C(CCl)C#N | 0 | train |
| 144213769 | CCCCCCCCCCCCOC(=O)C | 0 | validation |
| 144213768 | CCCCCCCOC(=O)C1=CC=C(C=C1)O | 1 | test |
| 144213767 | C1[C@H]([C@H](OC2=CC(=CC(=C21)O)O)C3=CC(=C(C(=C3)O)O)O)OC(=O)C4=CC(=C(C(=C4)O)O)O | 0 | train |
| 144213766 | C[NH+](C)CCC(C1=CC=C(C=C1)Cl)C2=CC=CC=N2.C(=C¥C(=O)[O-])¥C(=O)O | 0 | train |
| 144213765 | CC(C)CC(C)CC(CC(C)C)O | 0 | validation |
| 144213764 | CCCC[C@H]1CN(C(=O)OC12CCN(CC2)C3(CCN(CC3)C(=O)C4=C(N=CN=C4C)C)C)CC5CCOCC5 | 0 | test |
| 144213763 | CC1=CC(=C(C(=C1)C)C(=O)O)C | 0 | train |
| 144213762 | CCCCCCCCCCCCOCC1CO1 | 0 | train |
| 144213761 | C1=CC(=CN=C1)N | 0 | validation |
| 144213760 | [B-](F)(F)(F)F.[Na+] | 0 | test |
| 144213759 | CC(C)(/C=N/OC(=O)NC)S(=O)C | 0 | train |
| 144213758 | CCCCCC1=CCCC1=O | 0 | train |
| 144213757 | CCNCC(=C)C | 0 | validation |
| 144213756 | CCCCC1=CC=C(C=C1)N | 0 | test |
| 144213755 | C1=CC(=CC=C1N/C(=N/C(=NCCCCCCN=C(/N=C(/NC2=CC=C(C=C2)Cl)¥N)N)N)/N)Cl | 1 | train |
| 144213754 | CC1=C2C(=C(C(=C1C)OC(=O)C)C)CC[C@@](O2)(C)CCC[C@H](C)CCC[C@H](C)CCCC(C)C | 0 | train |
| 144213753 | C(CN(CC(=O)[O-])CC(=O)[O-])N(CC(=O)[O-])CC(=O)[O-].[Na+].[Fe+3] | 0 | validation |
| 144213752 | CCC(=O)C=CC | 0 | test |
| 144213751 | CC1=C(C=CO1)C(=O)NC2=CC=C(C=C2)C(=O)N3C4=CC=CC=C4/C(=C/C(=O)N5CCC(CC5)N6CCCCC6)/C(CC3)(F)F.CC1=C(C=CO1)C(=O)NC2=CC=C(C=C2)C(=O)N3C4=CC=CC=C4/C(=C/C(=O)N5CCC(CC5)N6CCCCC6)/C(CC3)(F)F.C(=C/C(=O)O)¥C(=O)O | 0 | train |
| 144213750 | C1=CC=C(C=C1)C(=O)C2=CC=CC=C2O | 0 | train |
| 144213749 | CCCCC(CC)COC(=O)CCCCCCCCC(=O)OCC(CC)CCCC | 0 | validation |
| 144213748 | CCCCCCCCCCCCCCCC[N+]1=CC=CC=C1.[Br-] | 0 | test |
| 144213747 | CCCC1=CC=C(C=C1)N | 0 | train |
| 144213746 | CCCCC1=CC=C(C=C1)Cl | 0 | train |
| 144213745 | CCCCN(CCCC)CCCCCCN(CCCC)CCCC | 0 | validation |
| 144213744 | CC(=CC[C@@H]1C(O1)(C)[C@H]2[C@@H]([C@@H](CC[C@]23CO3)OC(=O)NC(=O)CCl)OC)C | 0 | test |
| 144213743 | CC1=C(C=CC(=C1)C(C(F)(F)F)(C(F)(F)F)F)NC(=O)C2=C(C(=CC=C2)I)C(=O)NC(C)(C)CS(=O)(=O)C | 0 | train |
| 144213742 | CC(C)C(=O)C1=CC=CC=C1 | 0 | train |
| 144213741 | CCCCCCCCOC(=O)C1=CC=C(C=C1)O | 0 | validation |
| 144213740 | CC1=CC[C@@H]2C[C@H]1C2(C)C | 0 | test |
| 144213739 | CC(=O)OCCOC(=O)C | 0 | train |
| 144213738 | CC(=O)NC1=CC(=C(C=C1)OC)N | 0 | train |
| 144213737 | C1(=NC(=O)N(C(=O)N1Cl)Cl)[O-].O.O.[Na+] | 0 | validation |
| 144213736 | C([C@@H]1[C@@H]([C@@H]([C@H]([C@H](O1)O[C@]2([C@H]([C@@H]([C@H](O2)CCl)O)O)CCl)O)O)Cl)O | 0 | test |
| 144213735 | CC(C)(C)C(=O)CCl | 0 | train |
| 144213734 | CC1([C@H]([C@H]1C(=O)O[C@H](C#N)C2=CC(=CC=C2)OC3=CC=CC=C3)/C=C(/C(F)(F)F)¥Cl)C | 0 | train |
| 144213733 | CCCCCCOC(=O)C1=CC=C(C=C1)O | 1 | validation |
| 144213732 | CCCCCCCCCCC=CC1CC(=O)OC1=O | 0 | test |
| 144213731 | C1C(O1)COC2=CC=C(C=C2)CC3=CC=C(C=C3)OCC4CO4 | 0 | train |
| 144213730 | CC(=CCO)C | 0 | train |
| 144213729 | C1=CC=C(C=C1)COC(=O)C2=CC=CC=C2 | 0 | validation |
| 144213728 | CC(C)CCCCCOC(=O)CS | 0 | test |
| 144213727 | COC1=CC=C(C=C1)N2C(=CC(=N2)C(F)F)C3=CC=C(C=C3)S(=O)C | 0 | train |
| 144213726 | C1=CC=C2C(=C1)C3=CC=CC=C3S2 | 0 | train |
| 144213725 | CCCOCC(C)O | 0 | validation |
| 144213724 | CC[Si](OC(=O)C)(OC(=O)C)OC(=O)C | 0 | test |
| 144213723 | CC(=O)[O-].CC(=O)[O-].[Mn+2] | 1 | train |
| 144213722 | CC1=C[C@@H]2[C@H](CC[C@]3([C@H]2CC(=C)[C@@]3(C(=O)C)OC(=O)C)C)[C@@]4(C1=CC(=O)CC4)C | 1 | train |
| 144213721 | C1=CC(=CC(=C1)N(CC2=CN=CC=C2)C3=CC(=C(C=C3)OC(F)F)OC(F)F)C(=O)O | 0 | validation |
| 144213720 | CC1=CCC(CC1)C(=C)C | 0 | test |
| 144213719 | CC1=CC(=C(C(=C1)C(C)(C)C)O)C(C)(C)C | 1 | train |

| | | | |
|---|---|---|---|
| 144213718 | CN1C2=NC(=NC=C2CN(C1=O)C3=CC=CC=C3Br)NC4=CC5=C(C=C4)OC(CO5)CO | 0 | train |
| 144213717 | C1(=C(SSC1=O)Cl)Cl | 1 | validation |
| 144213716 | CC1=CC(=C(C=C1)C(=O)[N-]S(=O)(=O)/C=C/C2=CC=C(C=C2)OC)Cl.[Na+] | 0 | test |
| 144213715 | CN1C(=CN=C1COC(=O)N)[N+](=O)[O-] | 0 | train |
| 144213714 | C1=CC(=NC(=C1)N)N | 0 | train |
| 144213713 | CN1CCOCC1 | 0 | validation |
| 144213712 | C1=CC=C(C=C1)OP(=O)(O)OC2=CC=CC=C2 | 0 | test |
| 144213711 | CCCCCCCCC/C=C/CC1CC(=O)OC1=O | 0 | train |
| 144213710 | CC1=CC2=C(C=C1)C(=O)OC2=O | 0 | train |
| 144213709 | CCC1=CC(=C(C(=C1)C(C)(C)C)O)C(C)(C)C | 1 | validation |
| 144213708 | C1C(O1)COC2=CC=C(C=C2)C(C3=CC=C(C=C3)OCC4CO4)C(C5=CC=C(C=C5)OCC6CO6)C7=CC=C(C=C7)OCC8CO8 | 1 | test |
| 144213707 | CCCC[Sn](CCCC)(CCCC)OC(=O)C1=CC=CC=C1 | 1 | train |
| 144213706 | [N+](=O)([O-])[O-].[K+] | 0 | train |
| 144213705 | C1CC(OC1)CO | 0 | validation |
| 144213704 | CC(C)(C1=CC=CC=C1)C2=CC=C(C=C2)NC3=CC=C(C=C3)C(C)(C)C4=CC=CC=C4 | 0 | test |
| 144213703 | C1=CC(=CC=C1COC(CN2C=CN=C2)C3=C(C=C(C=C3)Cl)Cl)Cl.[N+](=O)(O)[O-] | 1 | train |
| 144213702 | CCC/C=C/C=O | 0 | train |
| 144213701 | CCCC[N+]1(CCCC1)C.[Cl-] | 0 | validation |
| 144213700 | C([C@H]([C@H]([C@@H]([C@H](CO)O)O)O)O)O | 0 | test |
| 144213699 | C1CCC(CC1)OC(=O)CC(C(=O)OC2CCCCC2)S(=O)(=O)[O-].[Na+] | 0 | train |
| 144213698 | CCC(C)(C)C1CCC(CC1)OC | 0 | train |
| 144213697 | CC1=CCC2CC1C2(C)C | 0 | validation |
| 144213696 | CCOCC(=O)O | 0 | test |
| 144213695 | C1=CC=C(C=C1)CCO | 0 | train |
| 144213694 | CCCCC(CC)CO[N+](=O)[O-] | 0 | train |
| 144213693 | CCC(=O)C1=CC=C(C=C1)Cl | 0 | validation |
| 144213692 | CCCOC(=O)C1=CC=CC=C1C(=O)OCCC | 0 | test |
| 144213691 | CCN(CC)C(=O)C1=CC(=C(C=C1N(CC)CCN(C)C)N2CCC(CC2)C3=CC=CC=C3)S(=O)(=O)CC4=CC=CC=C4.C(=O)(C(F)(F)F)O | 0 | train |
| 144213690 | C1=CC(=CC=C1/C=C/C2=CC(=CC(=C2)O)O)O | 1 | train |
| 144213689 | CC1=CC2=C(C=C1)OC(=C2C3=CC=C(C=C3)Cl)CN(CC4=CC=CC=C4)C(=O)NC5=C(C=C(C=C5F)F)F | 0 | validation |
| 144213688 | C1=CC=C(C=C1)C(=O)C2=C(C(=C(C=C2)O)O)O | 0 | test |
| 144213687 | CC1=CC(=CC(=C1)O)C | 0 | train |
| 144213686 | CCN(CCN)C1=CC=CC(=C1)C | 0 | train |
| 144213685 | CC1=CC(=O)[N-]S(=O)(=O)O1.[K+] | 0 | validation |
| 144213684 | CC(C)CC(=O)OCC=C | 0 | test |
| 144213683 | CCCCCCCC/C=C¥CCCCCCCC1=NCCN1CCO | 1 | train |
| 144213682 | CCOC(=O)C1C(O1)C2=CC=CC=C2 | 0 | train |
| 144213681 | CC(=CCCC(C)(C=C)OC=O)C | 0 | validation |
| 144213680 | C(NC(=O)N(CO)C1C(=O)N(C(=O)N1CO)CO)O | 0 | test |
| 144213679 | CC(=C)C(=O)OCCOCCOCCOC(=O)C(=C)C | 0 | train |
| 144213678 | C1=CC(=CC=C1C2=C(C(=O)C3=C(C=C(C=C3O2)O)O)O)O | 1 | train |
| 144213677 | C1=CC(=CC(=C1)CN)CN | 0 | validation |
| 144213676 | CCCCCCCCCCCCC1=CC=CC=C1O | 1 | test |
| 144213675 | CC1COC2=CC=CC=C2N1C(=O)C(Cl)Cl | 1 | train |
| 144213674 | CCC1=CC(=C(C(=C1)C(C)(C)C)O)CC2=C(C(=CC(=C2)CC)C(C)(C)C)O | 1 | train |
| 144213673 | CCCCOC1=CC=C(C=C1)N | 1 | validation |
| 144213672 | C[N+](C)(CC=C)CC=C.[Cl-] | 0 | test |
| 144213671 | C1=CC2=C(C=CC(=C2)SSC3=CC4=C(C=C3)C=C(C=C4)O)C=C1O | 1 | train |
| 144213670 | CC(C)(C)C1=CC=C(C=C1)N | 0 | train |
| 144213669 | CS(=O)(=O)C1=C(C=C(C=C1)[C@@H](CC2CCCC2)C(=O)NC3=NC=CN=C3)Cl | 0 | validation |
| 144213668 | CC(=C)C(=O)OCCC[Si](OC)(OC)OC | 0 | test |
| 144213667 | CC(=C)OC(=O)C | 0 | train |
| 144213666 | CC1=C(C2=C(O1)C(=CC=C2)NC(=O)C3=C(C=CC=C3Cl)Cl)C(C)(C)O | 0 | train |
| 144213665 | C1=CC=C(C=C1)OC2=CC=CC(=C2)C=O | 0 | validation |
| 144213664 | CCCCCCC(C)O | 0 | test |
| 144213663 | C(=O)(C(=O)O)O | 0 | train |
| 144213662 | C1=CN=CN1 | 0 | train |
| 144213661 | CC(C)(CNC1=NC=C2C(=CC(=O)N(C2=N1)C)OC3=C(C=C(C=C3)F)F)O.Cl | 0 | validation |
| 144213660 | CCCC1CCC(=O)CC1 | 0 | test |

| | | | |
|---|---|---|---|
| 144213659 | CCCCCC=O | 0 | train |
| 144213658 | CC(=O)CCC1=CC=CC=C1 | 0 | train |
| 144213657 | CC(=O)N1[C@@H]2[C@H]1[C@@]3([C@H](C4=C(C=C(C=C4OC)C=O)N(C2)O3)COC(=O)N)OC(=O)C | 1 | validation |
| 144213656 | C1=CC(=C(C=C1C(F)(F)F)[N+](=O)[O-])Cl | 0 | test |
| 144213655 | CC1=C(C=CC(=C1)Cl)OCCCC(=O)O | 0 | train |
| 144213654 | CC1=C(C(=CC=C1)C)[N+](=O)[O-] | 0 | train |
| 144213653 | C1=CC(=CC=C1N)S(=O)(=O)N | 0 | validation |
| 144213652 | CC1=C(C=CC(=C1)Cl)OC(C)C(=O)O | 0 | test |
| 144213651 | CCOC1=CC=C(C=C1)N | 0 | train |
| 144213650 | C1=CC=C(C=C1)NC2=CC=C(C=C2)[N+](=O)[O-] | 0 | train |
| 144213649 | CCCCCC1=CC(=CC(=C1)O)O | 1 | validation |
| 144213648 | COC1=CC=C(C=C1)C=O | 0 | test |
| 144213647 | CCO[Si](CCCN)(OCC)OCC | 0 | train |
| 144213646 | CN(C)CC1=CC(=C(C(=C1)CN(C)C)O)CN(C)C | 0 | train |
| 144213645 | CN(C)CCCN | 0 | validation |
| 144213644 | CC(C)COC(=O)C(=C)C | 0 | test |
| 144213643 | CCCCC(CC)C=O | 0 | train |
| 144213642 | C1CCC2=CC=CC=C2C1 | 0 | train |
| 144213641 | CCCCCCCC/C=C¥CCCCCCCC(=O)O | 0 | validation |
| 144213640 | CCCCCCOC(=O)C(=C)C | 0 | test |
| 144213639 | CC/C=C¥C/C=C¥C/C=C¥CCCCCCCC(=O)O | 0 | train |
| 144213638 | C1=CC=C2C(=C1)NC=N2 | 0 | train |
| 144213637 | CCO[Si](CCC#N)(OCC)OCC | 0 | validation |
| 144213636 | C1=CC=C2C(=C1)C(=O)NC2=O | 0 | test |
| 144213635 | CCCCC(CC)COC(=O)C | 0 | train |
| 144213634 | CC(C)(C)C(=O)C1C(=O)C2=CC=CC=C2C1=O | 1 | train |
| 144213633 | CCCCCCCCCCCCOC(=O)C(=C)C | 0 | validation |
| 144213632 | CC(C)(C1=CC=C(C=C1)OCC2CO2)C3=CC=C(C=C3)OCC4CO4 | 0 | test |
| 144213631 | [NH4+].[O-]Cl(=O)(=O)=O | 0 | train |
| 144213630 | CCCCC(CC)COP(=O)(OC1=CC=CC=C1)OC2=CC=CC=C2 | 1 | train |
| 144213629 | C1CC2C(O2)CC1COC(=O)C3CCC4C(C3)O4 | 0 | validation |
| 144213628 | CSC1=CC=C(C=C1)Cl | 0 | test |
| 144213627 | C1=CC2=C(C=C(C=C2C=C1N)S(=O)(=O)O)O | 0 | train |
| 144213626 | C1=CC=C(C=C1)OCCOC2=CC=CC=C2 | 1 | train |
| 144213625 | C(CCCCCC(=O)O)CCCCC(=O)O | 0 | validation |
| 144213624 | C1=CC=NC(=C1)Cl | 0 | test |
| 144213623 | C(C(=O)O)S | 0 | train |
| 144213622 | C1=CC(=CC=C1F)Br | 0 | train |
| 144213621 | C1CCCNCC1 | 0 | validation |
| 144213620 | CCCCCCCCCCC=C | 0 | test |
| 144213619 | COC(=O)C1=CC=C(C=C1)C=O | 0 | train |
| 144213618 | C1=CC(=CC=C1C(=O)O)O | 0 | train |
| 144213617 | CC(C)(CO)COC(=O)C(C)(C)CO | 0 | validation |
| 144213616 | C1=CC=C2C=C3C=C(C=CC3=CC2=C1)N | 1 | test |
| 144213615 | CC(C)(CO)CO | 0 | train |
| 144213614 | CC1=CC(=O)N=C(N1)C(C)C | 0 | train |
| 144213613 | CCCCOC(=O)C=C | 0 | validation |
| 144213612 | C(CC=O)CC=O | 0 | test |
| 144213611 | C1=CC=C(C=C1)P(C2=CC=CC=C2)C3=CC=CC=C3 | 1 | train |
| 144213610 | COC(=O)C1CCC(CC1)C(=O)OC | 0 | train |
| 144213609 | C1=CC=C(C(=C1)N)[N+](=O)[O-] | 0 | validation |
| 144213608 | C(C(=O)O)(Br)Br | 0 | test |
| 144213607 | CCCCCNCCCCC | 0 | train |
| 144213606 | CC(C)(C1=CC(=CC=C1)C(C)(C)N=C=O)N=C=O | 0 | train |
| 144213605 | CO[Si](CCCOCC1CO1)(OC)OC | 0 | validation |
| 144213604 | CC(C)OC(=O)C | 0 | test |
| 144213603 | C1(=NC(=NC(=N1)Cl)Cl)Cl | 0 | train |
| 144213602 | CCN(CC)C1=CC2=C(C=C1)C(=CC(=O)O2)C | 1 | train |
| 144213601 | CC(=O)C(=O)O | 0 | validation |
| 144213600 | C1=CC=C2C=C(C(=CC2=C1)C(=O)O)O | 1 | test |
| 144213599 | CC(C)OC=O | 0 | train |

| | | | |
|---|---|---|---|
| 144213598 | C[Si](C)(C)N[Si](C)(C)C | 0 | train |
| 144213597 | CC1CCC2CC1C2(C)C | 0 | validation |
| 144213596 | CC(=O)CC(=O)NC1=CC=CC=C1 | 0 | test |
| 144213595 | C1COCCN1SSC2=NC3=CC=CC=C3S2 | 0 | train |
| 144213594 | CCCCCCCCCCCCCC | 0 | train |
| 144213593 | CCCCOC(=O)COC(=O)C1=CC=CC=C1C(=O)OCCCC | 0 | validation |
| 144213592 | COC(=O)CCSCCC(=O)OC | 0 | test |
| 144213591 | C1=CC=C2C(=C1)NC(=S)N2 | 0 | train |
| 144213590 | C(=S)(NN)NN | 0 | train |
| 144213589 | C1=CC(=C(C(=C1Cl)Cl)Cl)Cl | 0 | validation |
| 144213588 | C1=CC=C(C=C1)/C=C/C=O | 1 | test |
| 144213587 | CCOP(=S)(OCC)OC1=NC2=CC=CC=C2N=C1 | 1 | train |
| 144213586 | CC(COC)O | 0 | train |
| 144213585 | CC1=CCC(=C(C)C)CC1 | 0 | validation |
| 144213584 | CCCCCCC(CCCCCCCCCCC(=O)OC)O | 0 | test |
| 144213583 | CC(C)(/C=N/OC(=O)NC)S(=O)(=O)C | 0 | train |
| 144213582 | CC(=O)CC(=O)N(C)C | 0 | train |
| 144213581 | C(=O)(N)N=NC(=O)N | 0 | validation |
| 144213580 | C(CCCCC(=O)O)CCCC(=O)O | 0 | test |
| 144213579 | C1(=O)[N-]C(=O)N(C(=O)N1Cl)Cl.[Na+] | 0 | train |
| 144213578 | C1=CC(=CC=C1C(F)(F)F)Cl | 0 | train |
| 144213577 | C[N+](C)(C)CC1=CC=CC=C1.[Cl-] | 0 | validation |
| 144213576 | C1CC(=O)NC1 | 0 | test |
| 144213575 | CCCCC(CC)C(=O)Cl | 0 | train |
| 144213574 | C1=CC=C(C=C1)S | 0 | train |
| 144213573 | CC(C)(C#N)O | 0 | validation |
| 144213572 | COC1=C2C=CC(=O)OC2=CC3=C1C=CO3 | 1 | test |
| 144213571 | CCCCC(CC)COC(=O)/C=C¥C(=O)OCC(CC)CCCC | 0 | train |
| 144213570 | CCCC(C(CC)CO)O | 0 | train |
| 144213569 | C=CC(=O)OCC(CO)(COC(=O)C=C)COC(=O)C=C | 1 | validation |
| 144213568 | C1=CC=C(C=C1)[O-].[Na+] | 0 | test |
| 144213567 | C1=CC(=CC(=C1)[N+](=O)[O-])C(=O)O | 0 | train |
| 144213566 | C1=CC=C(C=C1)NN=NC2=CC=CC=C2 | 1 | train |
| 144213565 | C1=CC(=C(C=C1Cl)Cl)OCC(=O)[O-].[Na+] | 0 | validation |
| 144213564 | C(CC(=O)O)C(=O)O | 0 | test |
| 144213563 | OP(=O)(O)O | 0 | train |
| 144213562 | C(COCCOCCOCCO)O | 0 | train |
| 144213561 | C/C=C(¥C)/C=C/C=C(C)C | 0 | validation |
| 144213560 | CC(C)(C)C=C | 0 | test |
| 144213559 | CC1=CCC(CC1)C(C)(C)OC(=O)C | 0 | train |
| 144213558 | C1=CC=C2C(=C1)OC3=CC=CC=C3S2 | 0 | train |
| 144213557 | C1COCCN1 | 0 | validation |
| 144213556 | C(CO)N1C(=O)N(C(=O)N(C1=O)CCO)CCO | 0 | test |
| 144213555 | C1=CC(=CC=C1N)S(=O)(=O)NC2=NC=CS2 | 0 | train |
| 144213554 | C1CCC(CC1)N=C=NC2CCCCC2 | 1 | train |
| 144213553 | C(C(C(CC(=O)O)C(=O)O)C(=O)O)C(=O)O | 0 | validation |
| 144213552 | CCOC(=O)C(=C)C#N | 0 | test |
| 144213551 | CC1(CC2=C(O1)C(=CC=C2)O)C | 0 | train |
| 144213550 | C[Si]1(O[Si](O[Si](O1)(C)C)(C)C)C | 0 | train |
| 144213549 | C1=CC=C2C(=C1)N=CS2 | 0 | validation |
| 144213548 | C1=CC(=C(C=C1Br)Br)Br | 0 | test |
| 144213547 | CCCCOCCOC(=O)C | 0 | train |
| 144213546 | C1=CC(=CC=C1C(=O)O)C(=O)O | 0 | train |
| 144213545 | CO[Si](CCCNCCN)(OC)OC | 0 | validation |
| 144213544 | CCCCC(CC)COC(=O)C=C | 0 | test |
| 144213543 | CC(C(=O)O)Br | 0 | train |
| 144213542 | CC(C)C1=C(C(=CC=C1)C(C)C)O | 0 | train |
| 144213541 | CCCCCCCCCCCCCl | 0 | validation |
| 144213540 | C1=CC(=CC=C1NC(=O)C2=CC(=CC(=C2O)Br)Br)Br | 1 | test |
| 144213539 | CC(=O)C=CC1=CC=CC=C1 | 0 | train |
| 144213538 | CC(=O)CC(=O)NC1=CC=CC=C1OC | 0 | train |
| 144213537 | C1CCC(C(C1)N)N | 0 | validation |

| | | | |
|---|---|---|---|
| 144213536 | C1=CC(=CC(=C1)Cl)N(CCO)CCO | 0 | test |
| 144213535 | CC1=CC(=C2C(=C1)C(=O)C3=CC(=CC(=C3C2=O)O)O)O | 1 | train |
| 144213534 | CC(C)C1=CC=C(C=C1)C(C)C | 0 | train |
| 144213533 | CN(C)CCOCCN(C)C | 0 | validation |
| 144213532 | CC1=CC(=C(C=C1)[N+](=O)[O-])C | 0 | test |
| 144213531 | CC1COC(=O)O1 | 0 | train |
| 144213530 | B(C1=CC=CC=C1)(C2=CC=CC=C2)C3=CC=CC=C3 | 1 | train |
| 144213529 | C12=C(C(=C(C(=C1Cl)Cl)Cl)Cl)C(=O)OC2=O | 0 | validation |
| 144213528 | CCCCOCC1CO1 | 0 | test |
| 144213527 | CC1=CC(=C(C=C1)[N+](=O)[O-])[N+](=O)[O-] | 1 | train |
| 144213526 | COC(=O)C1=CC(=CC=C1)C(=O)OC | 0 | train |
| 144213525 | CC=CC(=O)O | 0 | validation |
| 144213524 | CCCCC(CC)COS(=O)(=O)[O-].[Na+] | 0 | test |
| 144213523 | C1=CC(=C(C=C1Cl)Cl)N | 0 | train |
| 144213522 | C1=CC=[N+](C(=C1)[S-])[O-].C1=CC=[N+](C(=C1)[S-])[O-].[Zn+2] | 1 | train |
| 144213521 | CCCCO[Al](OCCCC)OCCCC | 0 | validation |
| 144213520 | CC1=CC(=CC=C1)C | 0 | test |
| 144213519 | CC(C)NC1=CC=C(C=C1)NC2=CC=CC=C2 | 1 | train |
| 144213518 | CC1=CC=C(C=C1)C(C)(C)C | 0 | train |
| 144213517 | CCC(C)CO | 0 | validation |
| 144213516 | C1=CC(=CC(=C1)Cl)N | 0 | test |
| 144213515 | COC(=O)C1=CC=CC=C1 | 0 | train |
| 144213514 | CCOC(=O)C1=CC=CC=C1N | 0 | train |
| 144213513 | C1CCCCCC(CCCCC1)O | 0 | validation |
| 144213512 | CCC(COC(=O)C(=C)C)(COC(=O)C(=C)C)COC(=O)C(=C)C | 0 | test |
| 144213511 | CCC1(C(=O)NCNC1=O)C2=CC=CC=C2 | 0 | train |
| 144213510 | C(CBr)Cl | 0 | train |
| 144213509 | C1(=O)NC(=O)NC(=O)N1 | 0 | validation |
| 144213508 | C(=O)(N)NC(=O)N | 0 | test |
| 144213507 | C1=CC(=CC(=C1)C#N)C#N | 0 | train |
| 144213506 | CC1=CC(=C(C=C1)N)[N+](=O)[O-] | 1 | train |
| 144213505 | C1=CC=C2C(=C1)C=CC3=C2C=CC=N3 | 1 | validation |
| 144213504 | CN(C)C1=CC(=CC=C1)O | 1 | test |
| 144213503 | CC1=CC=CC2=NNN=C12 | 0 | train |
| 144213502 | C1=CC=C2C(=C1)C3=CC=CC4=C3C5=C(C=CC=C25)C=C4 | 1 | train |
| 144213501 | CCCCNS(=O)(=O)C1=CC=CC=C1 | 0 | validation |
| 144213500 | COC1=CC2=C(C=C1)N=C(S2)N | 1 | test |
| 144213499 | C[C@@]1(C(=O)N2[C@H](C(=O)N3CCC[C@H]3[C@@]2(O1)O)CC4=CC=CC=C4)NC(=O)[C@H]5CN([C@@H]6CC7=CNC8=CC=CC(=C78)C6=C5)C.C[C@@]1(C(=O)N2[C@H](C(=O)N3CCC[C@H]3[C@@]2(O1)O)CC4=CC=CC=C4)NC(=O)[C@H]5CN([C@@H]6CC7=CNC8=CC=CC(=C78)C6=C5)C.[C@@H]([C@H](C(=O)O)O)(C(=O)O)O | 0 | train |
| 144213498 | CS(=O)(=O)C1=CC=C(C=C1)Cl | 0 | train |
| 144213497 | CC1=CC2=C(C=C1)OC(=O)C=C2 | 0 | validation |
| 144213496 | C1=C(C(=C(C(=C1Cl)Cl)Cl)Cl)Cl | 0 | test |
| 144213495 | CC1=CC(=C(C=C1)N)S(=O)(=O)O | 0 | train |
| 144213494 | CC(C)(C#N)N=NC(C)(C)C#N | 0 | train |
| 144213493 | C1CC(C(CCC(C(CCC(C1Br)Br)Br)Br)Br)Br | 0 | validation |
| 144213492 | CCCC=NO | 0 | test |
| 144213491 | CC(CCC#N)C#N | 0 | train |
| 144213490 | C[Sn](Cl)(Cl)Cl | 0 | train |
| 144213489 | CC(=C)C#N | 0 | validation |
| 144213488 | CCCCCCCCCCCCCC(=O)OC(C)C | 0 | test |
| 144213487 | C[Si]1(O[Si](O[Si](O[Si](O[Si](O1)(C)C)(C)C)(C)C)(C)C)C | 0 | train |
| 144213486 | C1C[C@@H](O[C@@H]1CO)N2C=CC(=NC2=O)N | 0 | train |
| 144213485 | C=CC(=O)OCCCCCCOC(=O)C=C | 0 | validation |
| 144213484 | C(C(CO)(CO)N)O | 0 | test |
| 144213483 | C1=CC(=CC=C1C(=O)Cl)C(=O)Cl | 0 | train |
| 144213482 | CCCCC/C=C/C=C/C=O | 0 | train |
| 144213481 | C1=CC=C(C=C1)OCC(=O)O | 0 | validation |
| 144213480 | C(COCCO)N | 0 | test |
| 144213479 | C1=CC(=CC=C1OC2=CC=C(C=C2)Br)Br | 0 | train |

| | | | |
|---|---|---|---|
| 144213478 | CC1(CN=C(NC1)NN=C(/C=C/C2=CC=C(C=C2)C(F)(F)F)/C=C/C3=CC=C(C=C3)C(F)(F)F)C | 1 | train |
| 144213477 | C1=CC=C2C(=C1)C(=O)N=NN2 | 0 | validation |
| 144213476 | CCOP(=O)(Cl)Cl | 0 | test |
| 144213475 | CC(C)CC(C)CC(=O)CC(C)C | 0 | train |
| 144213474 | CSCCC=O | 0 | train |
| 144213473 | C1=CC(=CC=C1Br)Br | 0 | validation |
| 144213472 | C(C#N)C(=O)O | 0 | test |
| 144213471 | CCN(CC)C1=CC(=C(C=C1)C(=O)C2=CC=CC=C2C(=O)O)O | 1 | train |
| 144213470 | CC(=C)C(=O)OCCOC(=O)C(=C)C | 0 | train |
| 144213469 | CC1(C2CCC(C2)C1=C)C | 0 | validation |
| 144213468 | CC1=C(C=C(C=C1)[N+](=O)[O-])S(=O)(=O)O | 0 | test |
| 144213467 | CC1=CC(=CC(=C1N)C)C2=CC(=C(C(=C2)C)N)C | 1 | train |
| 144213466 | CC(C)(CO)N | 0 | train |
| 144213465 | CCCCCCCCC | 0 | validation |
| 144213464 | C1[C@@H](O[C@@H](S1)CO)N2C=CC(=NC2=O)N | 0 | test |
| 144213463 | CC1=CC=CC=C1NC(=O)CC(=O)C | 0 | train |
| 144213462 | CC(=C)C(=O)OCC1CO1 | 0 | train |
| 144213461 | C(C(=O)[O-])Cl.[Na+] | 0 | validation |
| 144213460 | CCCC=C(CC)C=O | 0 | test |
| 144213459 | CC(=O)OCC1=CC=CO1 | 0 | train |
| 144213458 | CCNC1=NC(=NC(=N1)SC)NC(C)(C)C | 1 | train |
| 144213457 | CC1=C(C2=CC=CC=C2C=C1)C | 0 | validation |
| 144213456 | C=CCNCC=C | 0 | test |
| 144213455 | C1=CC(=C(C(=C1)F)C(=O)NC(=O)NC2=CC=C(C=C2)Cl)F | 1 | train |
| 144213454 | C[Sn](C)(Cl)Cl | 0 | train |
| 144213453 | CC1=CC=C(C=C1)S(=O)(=O)N=C=O | 0 | validation |
| 144213452 | CCOP(=S)(OCC)OP(=S)(OCC)OCC | 0 | test |
| 144213451 | CC(=CCCC(C)(C=C)OC(=O)C)C | 0 | train |
| 144213450 | COCN(COC)C1=NC(=NC(=N1)N(COC)COC)N(COC)COC | 0 | train |
| 144213449 | C1CCC(CC1)OC(=O)C2=CC=CC=C2C(=O)OC3CCCCC3 | 1 | validation |
| 144213448 | C1=CC=C(C=C1)S(=O)(=O)O | 0 | test |
| 144213447 | C(=C/Cl)¥Cl | 0 | train |
| 144213446 | B(OC(C)C)(OC(C)C)OC(C)C | 0 | train |
| 144213445 | CCCCOC(=O)CC(CC(=O)OCCCC)(C(=O)OCCCC)OC(=O)C | 1 | validation |
| 144213444 | C(COCCOCCOCCOCCO)O | 0 | test |
| 144213443 | CCCCC(CC)COCC1CO1 | 0 | train |
| 144213442 | C1=CC(=C(C=C1N)C(=O)O)O | 0 | train |
| 144213441 | CC1=CC=C(C=C1)C(=O)OC | 0 | validation |
| 144213440 | C(CCCN)CCN | 0 | test |
| 144213439 | CC1(CC(CC(C1)(C)CN)N)C | 0 | train |
| 144213438 | CCCCOCNC(=O)C=C | 0 | train |
| 144213437 | C1=CC=C2C(=C1)NC(=N2)N | 0 | validation |
| 144213436 | C[Si](C)(C)O[Si](C)(C)C | 0 | test |
| 144213435 | C1=C2C(=CC3=C1C(=O)OC3=O)C(=O)OC2=O | 0 | train |
| 144213434 | C1=CC2=C(C=C1C(=O)O)C(=O)OC2=O | 0 | train |
| 144213433 | C1=CC(=C(C(=C1Cl)O)Cl)Cl | 0 | validation |
| 144213432 | COCCOCCOCCO | 0 | test |
| 144213431 | C(CNCCN)N | 0 | train |
| 144213430 | CC(C(=O)OC)OC1=CC=C(C=C1)OC2=C(C=C(C=N2)C(F)(F)F)Cl | 0 | train |
| 144213429 | CCCC[Sn](CCCC)(Cl)Cl | 1 | validation |
| 144213428 | C1=CC2=C(C3=C(C=CC=N3)C=C2)N=C1 | 1 | test |
| 144213427 | C1COCCN1SSN2CCOCC2 | 0 | train |
| 144213426 | C1=CC=C(C(=C1)C(=O)O)O | 0 | train |
| 144213425 | CCCCCCOCCOCCO | 0 | validation |
| 144213424 | CC(CC1=CC=C(C=C1)C(C)(C)C)C=O | 0 | test |
| 144213423 | CCOP(=O)(OCC)OCC | 0 | train |
| 144213422 | CC1=CCC(CC1OC(=O)C)C(=C)C | 0 | train |
| 144213421 | CC(=O)C1=CC=CC=N1 | 0 | validation |
| 144213420 | CCCCC(CC)C(=O)[O-].[K+] | 0 | test |
| 144213419 | C1=CC=C(C=C1)[S-].[Na+] | 0 | train |
| 144213418 | CCCCCCCCCCCCS | 0 | train |

| | | | |
|---|---|---|---|
| 144213417 | CC(=O)NS(=O)(=O)C1=CC=C(C=C1)N | 0 | validation |
| 144213416 | COCCO[Si](C=C)(OCCOC)OCCOC | 0 | test |
| 144213415 | C1(=C(C(=C(C(=C1Cl)Cl)C(=O)Cl)Cl)Cl)C(=O)Cl | 0 | train |
| 144213414 | C1CCC(CC1)NC2CCCCC2 | 0 | train |
| 144213413 | C1=CC=C(C(=C1)O)S(=O)(=O)O | 0 | validation |
| 144213412 | C/C(=C¥C=C¥C(=C¥C=C¥C(=O)OC)¥C)/C=C¥C=C(/C)¥C=C/C=C(/C)¥C=C/C(=O)O | 1 | test |
| 144213411 | CC(COCC(C)O)O | 0 | train |
| 144213410 | C1=COC(=C1)CO | 0 | train |
| 144213409 | C1CSSC1CCCCC(=O)O | 0 | validation |
| 144213408 | CC(C)CC(=O)CC(C)C | 0 | test |
| 144213407 | CC(C)(C)C1CCC(CC1)O | 0 | train |
| 144213406 | C1=CC(=C(C=C1C(F)(F)F)Cl)Cl | 0 | train |
| 144213405 | CC1=NC(=NC(=N1)OC)NC(=O)NS(=O)(=O)C2=C(SC=C2)C(=O)OC | 0 | validation |
| 144213404 | CCCCC(CC)COP(=O)(O)OCC(CC)CCCC | 0 | test |
| 144213403 | CC(=O)OCC1=CC2=C(C=C1)OCO2 | 0 | train |
| 144213402 | CC(C)CCCCCCCOC(=O)C=C | 0 | train |
| 144213401 | CCCCOCCOCCOC(=O)CCCCC(=O)OCCOCCOCCCC | 0 | validation |
| 144213400 | CCOP(OCC)OCC | 0 | test |
| 144213399 | CC1CC(CC(C1)(C)C)OC(=O)C2=CC=CC=C2O | 0 | train |
| 144213398 | C1=CC(=CC(=C1)N)C(F)(F)F | 0 | train |
| 144213397 | CCCCC/C=C¥C/C=C¥CCCCCCCC(=O)O | 0 | validation |
| 144213396 | C1CCCCCC(=O)NCCCCC1 | 1 | test |
| 144213395 | CC(COC1=CC=CC=C1)O | 0 | train |
| 144213394 | CN(C)C(=O)C(C1=CC=CC=C1)C2=CC=CC=C2 | 1 | train |
| 144213393 | CCCCCCCCCCCCCOS(=O)(=O)O.C(CO)N(CCO)CCO | 0 | validation |
| 144213392 | C1=CC=C(C=C1)NC2=CC=CC3=CC=CC=C32 | 1 | test |
| 144213391 | COC1=C(C=C(C=C1)C=O)OC | 0 | train |
| 144213390 | CCCCCCCCCCCCN(C)C | 0 | train |
| 144213389 | CCCCC(CC)COC(=O)C(=C)C | 0 | validation |
| 144213388 | C=C(CC(=O)O)C(=O)O | 0 | test |
| 144213387 | C1=CC=C(C=C1)P(Cl)Cl | 0 | train |
| 144213386 | C1=CC(=C(C=C1[N+](=O)[O-])S(=O)(=O)[O-])C=CC2=C(C=C(C=C2)[N+](=O)[O-<br>])S(=O)(=O)[O-].[Na+].[Na+] | 0 | train |
| 144213385 | C1(=C(N=C(C(=N1)Cl)N)N)C(=O)N=C(N)N.Cl | 0 | validation |
| 144213384 | CC=C1CC2CC1C=C2 | 0 | test |
| 144213383 | C(CCl)OP(OCCCl)OCCCl | 0 | train |
| 144213382 | COCCCN | 0 | train |
| 144213381 | CCCCO[P+](=O)OCCCC | 0 | validation |
| 144213380 | [NH4+].NS(=O)(=O)[O-] | 0 | test |
| 144213379 | CC1=CC(=C(C=C1C2(C3=CC=CC=C3S(=O)(=O)O2)C4=CC(=C(C=C4C)O)C)C)O | 0 | train |
| 144213378 | CCCCCCCCCCCCC1=CC=CC=C1 | 0 | train |
| 144213377 | CCCCCCCCCC(=O)OC | 0 | validation |
| 144213376 | C1=CC(=CC=C1N=O)O | 0 | test |
| 144213375 | C1=CC=C2C(=C1)C(=O)OC(=O)N2 | 0 | train |
| 144213374 | C1=CC=C(C(=C1)C(F)(F)F)[N+](=O)[O-] | 0 | train |
| 144213373 | C1=CC(=CC(=C1)C(=O)Cl)C(=O)Cl | 0 | validation |
| 144213372 | CCCCOCCOCCOC(=O)C | 0 | test |
| 144213371 | CC(C)CC1=CC=CC=C1 | 0 | train |
| 144213370 | C1=CC(=C(C=C1Cl)O)Cl | 0 | train |
| 144213369 | C[C@@]12CC[C@@H](C1(C)C)CC2=O | 0 | validation |
| 144213368 | CCCCCCCCCCCCC=C | 0 | test |
| 144213367 | C[C@@H]1[C@H]([C@H](C[C@@H](O1)O[C@@H]2[C@H](O[C@H](C[C@@H]2O)O[C@@H]3[C<br>@H](O[C@H](C[C@@H]3O)O[C@H]4CC[C@]5([C@@H](C4)CC[C@@H]6[C@@H]5C[C@H]([C@<br>]7([C@@]6(CC[C@@H]7C8=CC(=O)OC8)O)C)O)C)C)O)O | 1 | train |
| 144213366 | CN(C)C1=CC=C(C=C1)N=O | 1 | train |
| 144213365 | C1CCCCCC(=O)CCCCC1 | 0 | validation |
| 144213364 | CCOP(=O)(OCC)OC1=CC=C(C=C1)[N+](=O)[O-] | 0 | test |
| 144213363 | C1=CC=C(C=C1)COC(=O)C2=CC=CC=C2O | 1 | train |
| 144213362 | C(=C¥C(=O)O)¥C(=O)O | 0 | train |
| 144213361 | CC1=CC(=CC=C1)NC(=O)OC2=CC=CC(=C2)NC(=O)OC | 1 | validation |
| 144213360 | CCCCCCCCCCCCC | 0 | test |
| 144213359 | CC(C)C#N | 0 | train |

| | | | |
|---|---|---|---|
| 144213358 | C(#N)C(Br)Br | 0 | train |
| 144213357 | C(CCO)CO | 0 | validation |
| 144213356 | COC1=CC=C(C=C1)N | 0 | test |
| 144213355 | C1=CC=C2C=C(C=CC2=C1)O | 1 | train |
| 144213354 | CC(=CCCC(C)(C=C)O)C | 0 | train |
| 144213353 | CC(C)N(C1=CC=CC=C1)C(=O)CCl | 1 | validation |
| 144213352 | CCCCCCC(CCCCCCCCCCC(=O)O)O | 0 | test |
| 144213351 | CCCOCCO | 0 | train |
| 144213350 | COCCOC(=O)C1=CC=CC=C1C(=O)OCCOC | 0 | train |
| 144213349 | CN(C)CCC#N | 0 | validation |
| 144213348 | C1=CC=C2C(=C1)C(=O)C(=C(C2=O)Cl)Cl | 0 | test |
| 144213347 | CCCCC(=O)O | 0 | train |
| 144213346 | COP(=O)(C)OC | 0 | train |
| 144213345 | C[C@@H]1CC[C@H]([C@@H](C1)O)C(C)C | 0 | validation |
| 144213344 | C(=S)(N)NNC(=S)N | 0 | test |
| 144213343 | CC1=CC(=O)N(N1C)C2=CC=CC=C2 | 0 | train |
| 144213342 | CC1=C(C(=CC=C1)C)NC(=O)CN2CCCC2=O | 0 | train |
| 144213341 | CCC[N+](=O)[O-] | 0 | validation |
| 144213340 | C[C@@]12[C@H](C=C[C@@]3([C@@H]1[C@@H]([C@]45[C@H]3CC[C@](C4)(C(=C)C5)O)C(=O)O)OC2=O)O | 0 | test |
| 144213339 | CC1=CC(=CC=C1)N=NC2=CC=C(C=C2)N(C)C | 1 | train |
| 144213338 | C1(=C(C(=O)C(=C(C1=O)Cl)Cl)Cl)Cl | 0 | train |
| 144213337 | CCOC(=O)/C=C¥C(=O)OCC | 0 | validation |
| 144213336 | C1=CC=C(C=C1)N/N=C/2¥C(=O)C=CC3=CC(=CC(=C32)S(=O)(=O)[O-])S(=O)(=O)[O-].[Na+].[Na+] | 0 | test |
| 144213335 | C1=CC=C(C=C1)NN | 1 | train |
| 144213334 | C1=CC(=C(C=C1N)N)O.Cl.Cl | 0 | train |
| 144213333 | CCCC(=O)C | 0 | validation |
| 144213332 | C1CCC(=NO)CC1 | 0 | test |
| 144213331 | C[C@]12C[C@@H]([C@]3([C@H]([C@@H]1C[C@@H]4[C@]2(OC(O4)(C)C)C(=O)CO)CCC5=CC(=O)C=C[C@@]53C)F)O | 0 | train |
| 144213330 | C1=CC(=C(C=C1Cl)CC2=C(C=CC(=C2)Cl)O)O | 1 | train |
| 144213329 | CC1=CC=CC=C1C=O | 0 | validation |
| 144213328 | C(CCCC(=O)O)CCCC(=O)O | 0 | test |
| 144213327 | CCCCCCCCCCCC(=O)O | 0 | train |
| 144213326 | CC1([C@H]([C@H]1C(=O)O[C@H](C#N)C2=CC(=CC=C2)OC3=CC=CC=C3)C=C(Br)Br)C | 1 | train |
| 144213325 | CCCCCCCCCCCCCCCC(=O)OC/C=C(¥C)/C=C/C=C(¥C)/C=C/C1=C(CCCC1(C)C)C | 0 | validation |
| 144213324 | C(C[N+](=O)[O-])C(=O)O | 0 | test |
| 144213323 | CC1=CC(=NC(=N1)NS(=O)(=O)C2=CC=C(C=C2)N)C | 0 | train |
| 144213322 | C[C@@H]1CC(=O)C=C([C@]12C(=O)C3=C(O2)C(=C(C=C3OC)OC)Cl)OC | 0 | train |
| 144213321 | C1=CC=C(C=C1)CCN=C(N)N=C(N)N.Cl | 0 | validation |
| 144213320 | CC1=CC(=C(C=C1)O)C(C)(C)C | 0 | test |
| 144213319 | CN(C)C1=CC=C(C=C1)C(=N)C2=CC=C(C=C2)N(C)C.Cl | 0 | train |
| 144213318 | CC1=CN=CC=C1 | 0 | train |
| 144213317 | [Cl-].[Cl-].[Cd+2] | 1 | validation |
| 144213316 | C[C@@H]1CC[C@H]([C@@H](C1)O)C(C)C | 0 | test |
| 144213315 | C1=CC=C(C=C1)NC(=S)N | 0 | train |
| 144213314 | C([C@H]([C@@H]1[C@@H]([C@@H]2[C@H](O1)O[C@@H](O2)C(Cl)(Cl)Cl)O)O)O | 0 | train |
| 144213313 | C1=CC(=CC=C1CCCC(=O)O)N(CCCl)CCCl | 0 | validation |
| 144213312 | NN.OS(=O)(=O)O | 0 | test |
| 144213311 | CCOP(=S)(OCC)SCSC(C)(C)C | 0 | train |
| 144213310 | CC(CCC(=O)O)(C1=CC=C(C=C1)O)C2=CC=C(C=C2)O | 0 | train |
| 144213309 | CCC1=CC(=CC=C1)O | 0 | validation |
| 144213308 | C1=NNC(=N1)N | 0 | test |
| 144213307 | C1=CC(=C(C=C1C(=O)O)C(=O)O)C(=O)O | 0 | train |
| 144213306 | CC1=NC=C(N1C)[N+](=O)[O-] | 0 | train |
| 144213305 | CC1CC(=O)O1 | 0 | validation |
| 144213304 | C[C@]12C[C@@H]([C@H]3[C@H]([C@@H]1CC[C@@]2(C(=O)CO)O)CCC4=CC(=O)C=C[C@]34C)O | 0 | test |
| 144213303 | CCC1=CC=C(C=C1)O | 0 | train |
| 144213302 | C1=NC(=NC(=O)N1[C@H]2[C@@H]([C@@H]([C@H](O2)CO)O)O)N | 0 | train |
| 144213301 | C1=CC(=CC=C1SC2=CC(=C(C=C2Cl)Cl)Cl)Cl | 1 | validation |

| | | | |
|---|---|---|---|
| 144213300 | C(=S)(N)NN | 0 | test |
| 144213299 | C(CCCCCC(=O)O)CCCCCC(=O)O | 0 | train |
| 144213298 | COC1=CC=CC=C1[N+](=O)[O-] | 0 | train |
| 144213297 | CC1=CC2=C(C=C1C)N(C3=NC(=O)NC(=O)C3=N2)C[C@@H]([C@@H]([C@@H](CO)O)O)O | 0 | validation |
| 144213296 | C1=CC=C(C=C1)C#N | 0 | test |
| 144213295 | CC(COC1=CC=CC=C1)N(CCCl)CC2=CC=CC=C2.Cl | 1 | train |
| 144213294 | CC1=CC=C(C=C1)S(=O)(=O)OC2=CC=C(C=C2)N=NC3=C(C=C(C=C3)C4=CC(=C(C=C4)NN=C5C(=O)C=CC6=CC(=CC(=C65)S(=O)(=O)[O-])S(=O)(=O)[O-])C)C.[Na+].[Na+] | 0 | train |
| 144213293 | CCCCCC/C=C/CCCCCCCC(=O)O | 0 | validation |
| 144213292 | C(#N)[S-].[Na+] | 0 | test |
| 144213291 | CN(C(=O)N[C@@H]1[C@H]([C@@H]([C@H](O[C@@H]1O)CO)O)O)N=O | 0 | train |
| 144213290 | CC(C)OC(=O)NC1=CC=CC=C1 | 1 | train |
| 144213289 | C1=C(C=C(C(=C1Br)O)Br)Br | 0 | validation |
| 144213288 | C1=COC(=C1)CNC2=CC(=C(C=C2C(=O)O)S(=O)(=O)N)Cl | 0 | test |
| 144213287 | C1=CC(=O)C=CC1=O | 0 | train |
| 144213286 | C1=CC=C(C=C1)C2=NC(=NC(=N2)N)N | 1 | train |
| 144213285 | C=CC(=O)OCCO | 0 | validation |
| 144213284 | C(C(CBr)Br)OP(=O)(OCC(CBr)Br)OCC(CBr)Br | 1 | test |
| 144213283 | CCCCCCCCCCCCCCCC(=O)O | 0 | train |
| 144213282 | COC(=O)[C@H](CC1=CC=CC=C1)NC(=O)[C@H](CC(=O)O)N | 0 | train |
| 144213281 | CC1=C(C2=C(C=C1)C(=O)C3=CC=CC=C3C2=O)N | 1 | validation |
| 144213280 | C(C(CCl)Cl)Cl | 0 | test |
| 144213279 | C1CCCN(CCC1)N=O | 0 | train |
| 144213278 | CC1=CC=NC=C1 | 0 | train |
| 144213277 | CCOC(=O)C1C(O1)(C)C2=CC=CC=C2 | 0 | validation |
| 144213276 | C(C(CCl)O)O | 0 | test |
| 144213275 | C([C@@H]([C@@H]1C(=C(C(=O)O1)O)O)O)O | 0 | train |
| 144213274 | CC(C)C(C1=CC=C(C=C1)Cl)C(=O)OC(C#N)C2=CC(=CC=C2)OC3=CC=CC=C3 | 1 | train |
| 144213273 | CCNC(=S)NCC | 0 | validation |
| 144213272 | C1=CNC(=S)NC1=O | 0 | test |
| 144213271 | CC(=O)NC1=CC=C(C=C1)C(=O)CCl | 1 | train |
| 144213270 | CCCCCCO | 0 | train |
| 144213269 | C1=CC=C(C=C1)N(N=O)[O-].[NH4+] | 1 | validation |
| 144213268 | C=CC#N | 0 | test |
| 144213267 | C1=CC=C2C(=C1)C(=O)NS2(=O)=O | 0 | train |
| 144213266 | C1=CC=C(C=C1)OC(=O)C2=CC=CC=C2O | 0 | train |
| 144213265 | CCCCCCCCCl | 0 | validation |
| 144213264 | C1=CC(=C(C=C1N(CCO)CCO)[N+](=O)[O-])NCCO | 0 | test |
| 144213263 | CC=CC1=CC2=C(C=C1)OCO2 | 0 | train |
| 144213262 | C1=CC=C(C=C1)CC=O | 0 | train |
| 144213261 | CC(=C)C(=O)OCC=C | 0 | validation |
| 144213260 | C1=CC(=CC=C1N)N | 1 | test |
| 144213259 | C1=CC(=C(C=C1[N+](=O)[O-])N)C(=O)O | 0 | train |
| 144213258 | CC(=O)[C@]1(CC[C@@H]2[C@@]1(CC[C@H]3[C@H]2C=C(C4=CC(=O)[C@@H]5C[C@@H]5[C@]34C)Cl)C)OC(=O)C | 1 | train |
| 144213257 | CCCCCCCCCCCCN | 0 | validation |
| 144213256 | COCCl | 0 | test |
| 144213255 | CCCCCCCCCCN | 0 | train |
| 144213254 | C1=CC=C2C(=C1)C=CN2 | 0 | train |
| 144213253 | C1=CC=C(C=C1)NN.Cl | 1 | validation |
| 144213252 | CCCCCC(=O)C | 0 | test |
| 144213251 | C1=CC2=NNN=C2C=C1 | 0 | train |
| 144213250 | CC(=CCCC(=CCCC(=O)C)C)C | 0 | train |
| 144213249 | C1=C(C(=C(C(=C1Cl)Cl)Cl)O)Cl | 0 | validation |
| 144213248 | CCOC(=O)CC(=O)OCC | 0 | test |
| 144213247 | COC1=CC=C(C=C1)CC=C | 0 | train |
| 144213246 | C1=CC=C(C(=C1)C=O)O | 0 | train |
| 144213245 | CCCN(CCC)S(=O)(=O)C1=CC=C(C=C1)C(=O)O | 0 | validation |
| 144213244 | C1CCC(CC1)C(=O)N2CC3C4=CC=CC=C4CCN3C(=O)C2 | 0 | test |
| 144213243 | CC(=O)NCCC1=CNC2=C1C=C(C=C2)OC | 0 | train |
| 144213242 | C1(C(C(C(C(C1Cl)Cl)Cl)Cl)Cl)Cl | 0 | train |
| 144213241 | C1CCC(CC1)NSC2=NC3=CC=CC=C3S2 | 1 | validation |

| | | | |
|---|---|---|---|
| 144213240 | CC1=CC(=C(C(=C1)C(C)(C)C)O)CC2=C(C(=CC(=C2)C)C(C)(C)C)O | 1 | test |
| 144213239 | C1CN(CCN1)CCN | 0 | train |
| 144213238 | CCCCCCCCCCCC#N | 0 | train |
| 144213237 | CC1=C(C=CC(=C1)Cl)N.Cl | 0 | validation |
| 144213236 | C1=C(C=C(C(=C1Cl)O)SC2=CC(=CC(=C2O)Cl)Cl)Cl | 0 | test |
| 144213235 | [Cl-].[Cl-].[Cl-].[Fe+3] | 0 | train |
| 144213234 | CCCCC/C=C¥C/C=C¥CCCCCCCC(=O)OC | 0 | train |
| 144213233 | C1=C(C=C(C(=C1Cl)N)Cl)N | 1 | validation |
| 144213232 | C1=CC(=CC=C1N)Cl.Cl | 1 | test |
| 144213231 | CC1([C@@H](N2[C@H](S1)[C@@H](C2=O)NC(=O)COC3=CC=CC=C3)C(=O)[O-])C.[K+] | 0 | train |
| 144213230 | C1CCN(C1)N=O | 0 | train |
| 144213229 | C1(C(=O)NC(=O)N1)NC(=O)N | 0 | validation |
| 144213228 | C1=C(C(=CC(=C1Cl)N)Cl)C2=CC(=C(C=C2Cl)N)Cl | 1 | test |
| 144213227 | C1=CC(=CC=C1N)Br | 0 | train |
| 144213226 | COC1=CC=C(C=C1)N.Cl | 0 | train |
| 144213225 | C1CN2CCN1CC2 | 0 | validation |
| 144213224 | C1=CC(=C(C=C1N)Cl)N.OS(=O)(=O)O | 1 | test |
| 144213223 | CC(C)CCC(=O)C | 0 | train |
| 144213222 | CC(C)NCC(COC1=CC=CC2=CC=CC=C21)O.Cl | 0 | train |
| 144213221 | C1=CC(=CN=C1)C(=O)O | 0 | validation |
| 144213220 | CCOC1=C(C=CC(=C1)C=O)O | 0 | test |
| 144213219 | CC1(C2CCC(O1)(CC2)C)C | 0 | train |
| 144213218 | COC1=CC=C(C=C1)C2=COC3=CC(=CC(=C3C2=O)O)O | 1 | train |
| 144213217 | C1=CC=C(C=C1)NC2=CC3=CC=CC=C3C=C2 | 1 | validation |
| 144213216 | CC(=CCCC(=O)C)C | 0 | test |
| 144213215 | CCNC1=C(C=C2C(=C1)OC3=CC(=NCC)C(=CC3=C2C4=CC=CC=C4C(=O)OCC)C)C.Cl | 1 | train |
| 144213214 | COC1=CC=C(C=C1)C(=C(C2=CC=C(C=C2)OC)Cl)C3=CC=C(C=C3)OC | 0 | train |
| 144213213 | CC(C(=O)[O-])O.CC(C(=O)[O-])O.[Ca+2] | 0 | validation |
| 144213212 | CCOC1=CC=C(C=C1)NC(=O)C | 0 | test |
| 144213211 | C1=CC(=C(C=C1C2=CC(=C(C=C2)N)Cl)Cl)N.Cl.Cl | 1 | train |
| 144213210 | CCCC[Sn](CCCC)(CCCC)CCCC | 1 | train |
| 144213209 | C([C@@H](C(=O)O)N)O/C(=C/[N+]#N)/[O-] | 0 | validation |
| 144213208 | CCCCOCCOP(=O)(OCCOCCCC)OCCOCCCC | 0 | test |
| 144213207 | C=CCN=C=S | 0 | train |
| 144213206 | CCOC1=CC=C(C=C1)NC(=O)CC(C)O | 0 | train |
| 144213205 | CC1=C(C=CC(=C1)Cl)O | 0 | validation |
| 144213204 | C1=CC(=CN=C1)CCl.Cl | 0 | test |
| 144213203 | CC1=CC=C(C=C1)OP(=O)(OC2=CC=C(C=C2)C)OC3=CC=C(C=C3)C | 1 | train |
| 144213202 | CCC1=CC=CC=C1O | 0 | train |
| 144213201 | C1=CC(=CC=C1C2=CC(=O)C3=C(C=C(C=C3O2)O)O)O | 1 | validation |
| 144213200 | CCC1=CC=C(C=C1)C(C2=CC=C(C=C2)CC)C(Cl)Cl | 1 | test |
| 144213199 | C1=C(C(=CC(=C1Cl)Cl)Cl)OCC(=O)O | 0 | train |
| 144213198 | C1CN(CCN1)CCO | 0 | train |
| 144213197 | CN(C)C(=S)SC(=S)N(C)C | 1 | validation |
| 144213196 | C(CBr)Br | 0 | test |
| 144213195 | CC(CN1C2=CC=CC=C2SC3=CC=CC=C31)N(C)C.Cl | 0 | train |
| 144213194 | C1CC2=CC=CC3=C(C=CC1=C23)[N+](=O)[O-] | 1 | train |
| 144213193 | COC1=CC=C(C=C1)O | 0 | validation |
| 144213192 | C(C#CCO)O | 0 | test |
| 144213191 | C[C@H]1C(=O)O[C@@H]2CCN3[C@@H]2C(=CC3)COC(=O)[C@]([C@]1(C)O)(C)O | 0 | train |
| 144213190 | CN(C)N=O | 0 | train |
| 144213189 | CCOC(=O)N(C)N=O | 0 | validation |
| 144213188 | C(CC(=O)O)CC(=O)O | 0 | test |
| 144213187 | C1=CC(=CC=C1OC2=C(C=C(C=N2)Cl)Cl)OC3=C(C=C(C=N3)Cl)Cl | 0 | train |
| 144213186 | C1=CC=C(C=C1)N.Cl | 0 | train |
| 144213185 | CCCCN(CC)C(=O)SCCC | 0 | validation |
| 144213184 | C1COCCN1N=O | 0 | test |
| 144213183 | CCC(C)C=O | 0 | train |
| 144213182 | C1=CC=C(C=C1)C(=O)C2=C(C=C(C=C2)O)O | 1 | train |
| 144213181 | CC(=O)NC1=CC2=C(C=C1)C3=C(C2)C=C(C=C3)NC(=O)C | 1 | validation |
| 144213180 | CC(C)CON=O | 0 | test |
| 144213179 | CC1=C(C(CCC1)(C)C)/C=C/C(=O)C | 0 | train |

| | | | |
|---|---|---|---|
| 144213178 | CC(C1=CC=CC=C1)(C2=CC=CC=N2)OCCN(C)C.C(CC(=O)O)C(=O)O | 0 | train |
| 144213177 | C1=CC(=CC=C1O)Cl | 0 | validation |
| 144213176 | CC(CNCC(C)O)O | 0 | test |
| 144213175 | CC(=CCl)C | 0 | train |
| 144213174 | C(=O)/C(=C(¥C(=O)O)/Cl)/Cl | 0 | train |
| 144213173 | CC(C)C(=O)C | 0 | validation |
| 144213172 | CC1=CC(=C(C=C1NN=C2C=C(C3=CC=CC=C3C2=O)S(=O)(=O)[O-])S(=O)(=O)[O-])C.[Na+].[Na+] | 0 | test |
| 144213171 | C1=CC=C(C=C1)C(C#N)C2=CC=CC=C2 | 0 | train |
| 144213170 | CNC(=S)N(C)C | 0 | train |
| 144213169 | COC1=CC(=C(C=C1)C(=O)C2=CC=CC=C2)O | 1 | validation |
| 144213168 | CC1=C(ON=C1C)NS(=O)(=O)C2=CC=C(C=C2)N | 0 | test |
| 144213167 | CN(C(=O)N)N=O | 0 | train |
| 144213166 | C1=CC=C2C=C(C=CC2=C1)NC3=CC=C(C=C3)NC4=CC5=CC=CC=C5C=C4 | 0 | train |
| 144213165 | CCN(CC)C(=O)C(=C(C)OP(=O)(OC)OC)Cl | 0 | validation |
| 144213164 | C1=CC=C2C(=C1)C3=CC=CC=C3N2 | 1 | test |
| 144213163 | CNC[C@@H](C1=CC(=CC=C1)O)O.Cl | 0 | train |
| 144213161 | CN(C)CCO | 0 | validation |
| 144213160 | C1=CC(=CC=C1O)S(=O)(=O)C2=CC=C(C=C2)O | 0 | test |
| 144213159 | C1=CC(=C(C=C1[As](=O)(O)O)[N+](=O)[O-])O | 0 | train |
| 144213158 | CC(=O)OCC1=CC=CC=C1 | 0 | train |
| 144213157 | CC(C)(C(=O)O)OC1=CC=C(C=C1)C2CC2(Cl)Cl | 0 | validation |
| 144213156 | C1=CC(=C(C=C1N)Cl)Cl | 0 | test |
| 144213155 | C1CCC(CC1)NC(=S)NC2CCCCC2 | 0 | train |
| 144213154 | CCCCNC(=O)N | 0 | train |
| 144213153 | CCCCOCCOCCOCCO | 0 | validation |
| 144213152 | COC1=C(C(=C(C(=C1Cl)Cl)Cl)Cl)Cl | 1 | test |
| 144213151 | C([N+](=O)[O-])([N+](=O)[O-])([N+](=O)[O-])[N+](=O)[O-] | 0 | train |
| 144213150 | C1=CC=C(C=C1)C2=NC3=C(N=C2N)N=C(N=C3N)N | 1 | train |
| 144213149 | C1=CC=NC(=C1)C#N | 0 | validation |
| 144213148 | C1=CC=C2C(=C1)OC3=CC=CC=C3O2 | 0 | test |
| 144213147 | CC1=C(C(=O)N(N1C)C2=CC=CC=C2)N(C)C | 0 | train |
| 144213146 | CC(C)CC1=CC=C(C=C1)C(C)C(=O)O | 0 | train |
| 144213145 | C1=CC2=C(C=C1[N+](=O)[O-])NC=N2 | 0 | validation |
| 144213144 | CC1=CC=C(O1)C(=O)O | 0 | test |
| 144213143 | C(=C/C(=O)O)¥C(=O)O | 0 | train |
| 144213142 | C1=CC(=CC(=C1)Cl)[N+](=O)[O-] | 0 | train |
| 144213141 | C(=O)N | 0 | validation |
| 144213140 | CC[N+](=O)[O-] | 0 | test |
| 144213139 | C1=CC=C(C=C1)NC2=CC=C(C=C2)N=O | 1 | train |
| 144213138 | C1=CC(=C(C=C1N)N)Cl | 1 | train |
| 144213137 | C1CCCN(CC1)N=O | 0 | validation |
| 144213136 | CC1=NN(C(=O)C1)C2=CC=CC=C2 | 0 | test |
| 144213135 | CC(CC(C)(C)O)O | 0 | train |
| 144213134 | C1C=CCS1(=O)=O | 0 | train |
| 144213133 | COC1=C2C(=CC3=C1OC=C3)C=CC(=O)O2 | 1 | validation |
| 144213132 | CCC[C@@H]1O[C@@H]2C[C@H]3[C@@H]4CCC5=CC(=O)C=C[C@@]5([C@H]4[C@H](C[C@@]3([C@@]2(O1)C(=O)CO)C)O)C | 0 | test |
| 144213131 | C=CC(CCl)Cl | 0 | train |
| 144213130 | C1=CC=C2C(=C1)N=C(S2)Br | 1 | train |
| 144213129 | CCN(CC)C(=S)[S-].O.O.O.[Na+] | 1 | validation |
| 144213128 | C1=CC(=C(C=C1[N+](=O)[O-])N)N | 1 | test |
| 144213127 | C1=CC=C(C=C1)S(=O)(=O)OC2=C(C=C(C=C2)Cl)Cl | 1 | train |
| 144213126 | C(C(=O)CCl)Cl | 0 | train |
| 144213125 | CC1=NC=CN1 | 0 | validation |
| 144213124 | COC1=CC=CC=C1N.Cl | 1 | test |
| 144213123 | C1CO[C@@H]([C@H](O1)Cl)Cl | 0 | train |
| 144213122 | C1=CC=C2C(=C1)C=CC=C2N | 1 | train |
| 144213121 | C1=CC=C(C(=C1)C(C2=CC=C(C=C2)Cl)C(Cl)Cl)Cl | 1 | validation |
| 144213120 | C1CCC(CC1)O | 0 | test |
| 144213119 | C1=CC(=C(C=C1[N+](=O)[O-])[N+](=O)[O-])Cl | 1 | train |
| 144213118 | C1=CC=C(C(=C1)C(=O)N)O | 0 | train |

| | | | |
|---|---|---|---|
| 144213117 | [O-]Cl=O.[Na+] | 0 | validation |
| 144213116 | CO[P+](=O)OC | 0 | test |
| 144213115 | CC1=C(C=CC(=C1)C2=CC(=C(C=C2)N)C)N.Cl.Cl | 0 | train |
| 144213114 | CC(C)C1=CC=C(C=C1)CO | 0 | train |
| 144213113 | CC1=CC=CC(=C1)C(=O)O | 0 | validation |
| 144213112 | CCCCNC(=O)NS(=O)(=O)C1=CC=C(C=C1)C | 0 | test |
| 144213111 | CC1=NC=C(N1CCO)[N+](=O)[O-] | 0 | train |
| 144213110 | CCCCC(CC)COC(=O)C1=CC=C(C=C1)O | 0 | train |
| 144213109 | C=CC1=CC=CC=C1C=C | 0 | validation |
| 144213108 | C[C@H](CCC(=O)O)[C@H]1CC[C@@H]2[C@@]1(CC[C@H]3[C@H]2CC[C@H]4[C@@]3(CC[C@H](C4)O)C)C | 0 | test |
| 144213107 | C1=CC=C(C=C1)C(C(=O)C2=CC=CC=C2)O | 1 | train |
| 144213106 | CCCNC(=O)NS(=O)(=O)C1=CC=C(C=C1)Cl | 0 | train |
| 144213105 | C1=CC(=C(C=C1[N+](=O)[O-])N)O | 1 | validation |
| 144213104 | CC(=O)N(C)C | 0 | test |
| 144213103 | CC(C)OC(=O)COC1=C(C=C(C=C1)Cl)Cl | 0 | train |
| 144213102 | CN(C)P(=O)(N(C)C)N(C)C | 0 | train |
| 144213101 | CC1=CC(=C(C=C1)C)OCCCC(C)(C)C(=O)O | 0 | validation |
| 144213100 | C1=CC=C2C(=C1)C=CC=C2NCCN.Cl.Cl | 0 | test |
| 144213099 | COC1=CC=C(C=C1)OC | 0 | train |
| 144213098 | CCCCCCCCCCCCCC(=O)O | 0 | train |
| 144213097 | C1(=C(C(C(=C1Cl)Cl)(Cl)Cl)Cl)Cl | 0 | validation |
| 144213096 | CCCCCCC=O | 0 | test |
| 144213095 | CCCCC1(C(=O)N(N(C1=O)C2=CC=CC=C2)C3=CC=CC=C3)COC(=O)CCC(=O)O | 0 | train |
| 144213094 | C1=CC=C(C=C1)OC2=CC=CC=C2 | 0 | train |
| 144213093 | C1=CC(=CN=C1)C(=O)N | 0 | validation |
| 144213092 | C1CN(P(=O)(OC1)NCCCl)CCCl | 0 | test |
| 144213091 | C(I)(I)I | 0 | train |
| 144213090 | CC(=O)NC1=NN=C(S1)S(=O)(=O)N | 0 | train |
| 144213089 | CC1=CC=CC=C1N.Cl | 0 | validation |
| 144213088 | C1=CN=C(C=N1)C(=O)N | 0 | test |
| 144213087 | C1C2=CC=CC=C2C3=C1C=C(C=C3)[N+](=O)[O-] | 1 | train |
| 144213086 | CCC1=NC=CC(=C1)C(=S)N | 0 | train |
| 144213085 | C1=CC=C2C(=C1)C=CC=C2O | 1 | validation |
| 144213084 | C(=S)(N)N | 0 | test |
| 144213083 | C1=CC(=CC=C1S(=O)(=O)C2=CC(=C(C=C2Cl)Cl)Cl)Cl | 1 | train |
| 144213082 | CC(C)N1C2=CC=CC=C2C(=C1/C=C/[C@H](C[C@H](CC(=O)O)O)O)C3=CC=C(C=C3)F | 0 | train |
| 144213081 | CC1=CC(=O)C(C(=O)O1)C(=O)C | 0 | validation |
| 144213080 | CCCCC1C(=O)N(N(C1=O)C2=CC=CC=C2)C3=CC=CC=C3 | 0 | test |
| 144213079 | CCCCCCCC(=O)OCC(COC(=O)CCCCCCC)OC(=O)CCCCCCC | 0 | train |
| 144213078 | C[C@]12CC[C@H]3[C@H]([C@@H]1CCC2=O)CC=C4[C@@]3(CC[C@@H](C4)O)C | 0 | train |
| 144213077 | C1CCC(CC1)N.Cl | 0 | validation |
| 144213076 | C1=CC=C2C(=C1)C=CC=C2[N+](=O)[O-] | 0 | test |
| 144213075 | C1=CC=C(C(=C1)[N+](=O)[O-])O | 0 | train |
| 144213074 | CCCCOC(=O)COC1=C(C=C(C=C1)Cl)Cl | 0 | train |
| 144213073 | CC1=CN=C(S1)NC(=O)C2=C(C3=CC=CC=C3S(=O)(=O)N2C)O | 1 | validation |
| 144213072 | CC1=C(C=C(C=C1)N)Cl | 0 | test |
| 144213071 | CCN1C=C(C(=O)C2=C1N=C(C=C2)C)C(=O)O | 0 | train |
| 144213070 | CC(=C)C(=O)NC1=CC(=C(C=C1)Cl)Cl | 1 | train |
| 144213069 | C[C@H](/C=C/[C@H](C)C(C)C)[C@H]1CC[C@@H]¥2[C@@]1(CCC/C2=C¥C=C/3¥C[C@H](CCC3=C)O)C | 1 | validation |
| 144213068 | CC1=CC=C(C=C1)C=C | 0 | test |
| 144213067 | C1CCC(=O)CC1 | 0 | train |
| 144213066 | CCN(C(=O)N)N=O | 0 | train |
| 144213065 | CC(CN(CC(C)O)CC(C)O)O | 0 | validation |
| 144213064 | CN(C1=CC=CC=C1)N=O | 0 | test |
| 144213063 | C1CCN(CC1)C(=O)/C=C/C=C/C2=CC3=C(C=C2)OCO3 | 1 | train |
| 144213062 | C1CC(=O)OC2=CC=CC=C21 | 0 | train |
| 144213061 | C[C@]12CC[C@H]3[C@H]([C@@H]1CC[C@@H]2C(=O)NC(C)(C)C)CC[C@@H]4[C@@]3(C=CC(=O)N4)C | 0 | validation |
| 144213060 | CC1=CC[C@@H](CC1=O)C(=C)C | 0 | test |
| 144213059 | C1=CC(=CC=C1OS(=O)(=O)C2=CC=C(C=C2)Cl)Cl | 1 | train |

| | | | |
|---|---|---|---|
| 144213058 | C1=C(C(=C(C(=C1Cl)Cl)CC2=C(C(=CC(=C2Cl)Cl)Cl)O)O)Cl | 0 | train |
| 144213057 | COP(=O)(OC)OC | 0 | validation |
| 144213056 | C[C@H]1[C@@H]([C@H]([C@H]([C@@H](O1)OC[C@@H]2[C@H]([C@@H]([C@H]([C@@H](O2)OC3=CC(=C4C(=O)C[C@H](OC4=C3)C5=CC(=C(C=C5)OC)OC)O)O)O)O)O)O)O | 0 | test |
| 144213055 | C1=CC=C(C=C1)N=NC2=C(N=C(C=C2)N)N.Cl | 1 | train |
| 144213054 | CC1=C(C(CCC1)(C)C)/C=C/C(=C/C=C/C(=C/COC(=O)C)/C)/C | 0 | train |
| 144213053 | C(=C(Cl)Cl)(C(=C(Cl)Cl)Cl)Cl | 0 | validation |
| 144213052 | CC(=O)C1=CC=C(C=C1)S(=O)(=O)NC(=O)NC2CCCCC2 | 0 | test |
| 144213051 | C1=CC=C(C=C1)C2=CC=C(C=C2)O | 1 | train |
| 144213050 | C1=CC=NC(=C1)CCl.Cl | 0 | train |
| 144213049 | C1=CC2=C(C=C1S(=O)(=O)[O-])C(=O)/C(=C¥3/C(=O)C4=C(N3)C=CC(=C4)S(=O)(=O)[O-])/N2.[Na+].[Na+] | 0 | validation |
| 144213048 | C1=CC=C2C(=C1)C(=CN2)CC(=O)O | 0 | test |
| 144213047 | C(#N)N=C(N)N | 0 | train |
| 144213046 | CC(=O)CCC(=O)O | 0 | train |
| 144213045 | C1CCC(CC1)C2CCCCC2=O | 0 | validation |
| 144213044 | C[C@]12CCCC([C@@H]1CC[C@@]([C@@H]2CC[C@](C)(C=C)O)(C)O)(C)C | 0 | test |
| 144213043 | CC(=O)OCC1=CC=C(C=C1)OC | 0 | train |
| 144213042 | CC(CCC1=CC=CC=C1)NCC(C2=CC(=C(C=C2)O)C(=O)N)O.Cl | 0 | train |
| 144213041 | CC(=CCOC(=O)C)C | 0 | validation |
| 144213040 | COC1=CC(=C(C=C1)OC)C(CNC(=O)CN)O.Cl | 0 | test |
| 144213039 | CCCC1=CC(=C(C=C1)O)OC | 0 | train |
| 144213038 | CC(C)C(=O)OC(C)(CCC=C(C)C)C=C | 0 | train |
| 144213037 | C=COCC1CCC(CC1)COC=C | 0 | validation |
| 144213036 | C1=CC=C(C=C1)COC(=O)NN | 0 | test |
| 144213035 | CCC(=O)OCCOC1=CC=CC=C1 | 0 | train |
| 144213034 | CCCCC(=O)C=CC | 0 | train |
| 144213033 | CC1=CC(=C(C(=C1)O)C)C | 0 | validation |
| 144213032 | C[Si](C)(CCCN)O[Si](C)(C)CCCN | 0 | test |
| 144213031 | C1=CC(=CC(=C1)C(F)(F)F)CC#N | 0 | train |
| 144213030 | CC/C(=C(/CC)¥C1=CC=C(C=C1)OC(=O)CC)/C2=CC=C(C=C2)OC(=O)CC | 0 | train |
| 144213029 | CCCC(C)C(=O)OCC | 0 | validation |
| 144213028 | CC(C)C1=CC2=C(C=C1)N=CC=C2 | 0 | test |
| 144213027 | CCCCCC(=O)C=C | 0 | train |
| 144213026 | CC1=C(C=CO1)S | 0 | train |
| 144213025 | CC(=CCCC(=CC(=O)O)C)C | 0 | validation |
| 144213024 | CC(=CCCC(=CC(=O)OC)C)C | 0 | test |
| 144213023 | CC(C1=CC=CC=C1)C(OC)OC | 0 | train |
| 144213022 | CC(=CCOC(=O)C1=CC=CC=C1)C | 0 | train |
| 144213019 | CC(C)C(=O)OCC=CC1=CC=CC=C1 | 0 | train |
| 144213018 | CC(CC=O)CC(C)(C)C | 0 | train |
| 144213017 | COC1=C(C=C(C=C1)N)N.O.OS(=O)(=O)O | 0 | validation |
| 144213016 | CCCC1CCCC(=O)O1 | 0 | test |
| 144213015 | CCCCCC(=O)OCCCCC | 0 | train |
| 144213014 | COP(=O)(O)OP(=O)(O)OC | 0 | train |
| 144213013 | CCCCCCCCCCCC(=O)O.C(CO)N(CCO)CCO | 0 | validation |
| 144213012 | CC(C)(C)OC(=O)C1=CC=CC=C1C(=O)O | 0 | test |
| 144213011 | CC(CCC=C(C)C)CC(=O)O | 0 | train |
| 144213010 | CCC(C)O[Al](OC(C)CC)O/C(=C¥C(=O)OCC)/C | 0 | train |
| 144213009 | CC/C=C¥CCOC(C)OCC | 0 | validation |
| 144213008 | C1=CN=C(C=N1)CCS | 0 | test |
| 144213007 | CC1=CC(=C(C(=C1)C)C(=O)P(=O)(C2=CC=CC=C2)C(=O)C3=C(C=C(C=C3C)C)C)C | 0 | train |
| 144213006 | C1=CC(=CC=C1N/C(=N/C(=NCCCCCCN=C(/N=C(/NC2=CC=C(C=C2)Cl)¥N)N)N)/N)Cl.Cl.Cl | 1 | train |
| 144213005 | CC(=CCC/C(=C/CC/C(=C/COC(=O)C)/C)/C)C | 0 | validation |
| 144213004 | CC1CC2=CC=CC=C2N1NC(=O)C3=CC(=C(C=C3)Cl)S(=O)(=O)N | 0 | test |
| 144213003 | C/C=C(¥C)/C(=O)OCCC1=CC=CC=C1 | 0 | train |
| 144213002 | C1CN(CCN1CCCN2C(=O)N3C=CC=CC3=N2)C4=CC(=CC=C4)Cl.Cl | 0 | train |
| 144213001 | CCOC1=CC=C(C=C1)O | 0 | validation |
| 144213000 | CC1=CC2=C(C=C1)OCC(=O)CO2 | 0 | test |
| 144212999 | COC1=CC=C(C=C1)C(=O)OC | 0 | train |
| 144212998 | CC1=CC(=C(C=C1)O)OC | 0 | train |

| | | | |
|---|---|---|---|
| 144212997 | CCOC(=O)C1=CC=CC=C1OC | 0 | validation |
| 144212996 | CCC(=O)OCCC(C)CCC=C(C)C | 0 | test |
| 144212995 | CN(CC#C)CC1=CC=CC=C1.Cl | 0 | train |
| 144212994 | CC1C(C2=C(C1(C)C)C(=O)CCC2)(C)C | 0 | train |
| 144212993 | CCCC1CCC(=O)O1 | 0 | validation |
| 144212992 | C1CCC(C1)S | 0 | test |
| 144212991 | C[C@]12CCCC([C@@H]1CC[C@@]3([C@@H]2CCO3)C)(C)C | 0 | train |
| 144212990 | CC1=C(C=C(C=C1)NC(=O)N(C)C)NC(=O)N(C)C | 0 | train |
| 144212989 | C1=CC=C(C=C1)CCCOC(=O)C=CC2=CC=CC=C2 | 0 | validation |
| 144212988 | CC(=O)OCCCCCCCCC=C | 0 | test |
| 144212986 | CC(=O)C1=CC2=C(CCCC2)C=C1 | 0 | train |
| 144212985 | CC(=CCCC(C)(C=C)OC(=O)C1=CC=CC=C1)C | 1 | validation |
| 144212984 | CC(=O)CC(=O)NC1=CC2=C(C=C1)NC(=O)N2 | 0 | test |
| 144212983 | CC1=C(C(CCC1)(C)C)CCC(=O)C | 0 | train |
| 144212982 | CC1=NN=C(C(=O)N1N)C2=CC=CC=C2 | 0 | train |
| 144212980 | CC/C=C¥CCOC(=O)C1=CC=CC=C1 | 0 | test |
| 144212979 | C1(=O)C(=O)NC(=O)NC1=O.O.O.O.O | 0 | train |
| 144212978 | CCO[Si](C)(CCCOCC1CO1)OCC | 0 | train |
| 144212977 | CC(C)(CO)C(C(=O)NCCCO)O | 0 | validation |
| 144212976 | CC1C=C[C-]=C1.[C-]#[O+].[C-]#[O+].[C-]#[O+].[Mn] | 1 | test |
| 144212975 | CC(=O)CCC1=CC2=C(C=C1)OCO2 | 0 | train |
| 144212974 | CC(C)CC(CC1=CC=CC=C1)O | 0 | train |
| 144212973 | CCCCCCCCCC=O | 0 | validation |
| 144212972 | CC(C)(C)N(C(=O)C1=CC=CC=C1)NC(=O)C2=CC=C(C=C2)Cl | 0 | test |
| 144212970 | CC1=CC=C(C=C1)N(CCO)CCO | 0 | train |
| 144212969 | C(CC(=O)O)[C@@H](C(=O)O)N.Cl | 0 | validation |
| 144212968 | C=CC(=O)OCCCCO | 0 | test |
| 144212967 | CC(C)CCCCCCCOC(=O)C1=CC(=C(C=C1)C(=O)OCCCCCCCC(C)C)C(=O)OCCCCCCCC(C)C | 0 | train |
| 144212966 | CC(CCC=C(C)C)CC(=O)OC | 0 | train |
| 144212965 | C/C=C(¥C)/C(=O)OCC1=CC=CC=C1 | 0 | validation |
| 144212964 | C[N+](C)(C)CCO.[OH-] | 0 | test |
| 144212963 | C1C(O1)CN(CC2CO2)C3=CC=C(C=C3)OCC4CO4 | 0 | train |
| 144212962 | CC1=CC(=CC=C1)O | 0 | train |
| 144212961 | C(CO)N(CCO)C=O | 0 | validation |
| 144212960 | CC1=CC=C(C=C1)OC(=O)CC(C)C | 0 | test |
| 144212959 | CCCC1=CC=C(C=C1)O | 0 | train |
| 144212958 | C[C@]12CC[C@H]3C(=CCC4=C3C=CC(=C4)O)[C@@H]1CCC2=O | 1 | train |
| 144212957 | CCCCC(C)C(=O)O | 0 | validation |
| 144212956 | CC1=CC(=CC(=C1C(=O)C)C)C(C)(C)C | 0 | test |
| 144212955 | CCOC(=O)C=CC1=CC=CC=C1 | 0 | train |
| 144212954 | C1CC(CC=C1)CO | 0 | train |
| 144212953 | CC1=CC=C(S1)C=O | 0 | validation |
| 144212952 | CC(=CCCC(C)(C=C)OC(=O)C=CC1=CC=CC=C1)C | 1 | test |
| 144212951 | CCOC(=O)COC1=CC=C(C=C1)C | 0 | train |
| 144212950 | C1=CC=C(C=C1)CCOC(=O)C=CC2=CC=CC=C2 | 1 | train |
| 144212949 | CC1=CCC2C(C1)C2(C)C | 0 | validation |
| 144212948 | CC(C)(CCC1=CC=CC=C1)O | 0 | test |
| 144212947 | CC/C=C¥CCOC(=O)C(C)C | 0 | train |
| 144212946 | CC1CCC(=O)C1=O | 0 | train |
| 144212945 | C1=CC=C(C=C1)C[N+]2=CC=CC3=CC=CC=C32.[Cl-] | 0 | validation |
| 144212944 | C1CC2C(C1)C3CC2CC3=O | 0 | test |
| 144212943 | CCCCCC(=O)OCC/C=C¥CC | 0 | train |
| 144212942 | C[C@@H](CCCC(=C)C)CCO | 0 | train |
| 144212941 | CC1CCC(=O)O1 | 0 | validation |
| 144212940 | CCCC(CCC)C(=O)[O-].[Na+] | 0 | test |
| 144212939 | C1=CC=C(C=C1)CCOC(=O)C2=CC=CC=C2 | 0 | train |
| 144212938 | C1CCCCCCCC(=O)OCCCCCCC1 | 0 | train |
| 144212937 | CCCCCCCCCC(C)C=O | 0 | validation |
| 144212936 | CCCCCCCCCCCCCCCCCC(=O)OCC(CC)CCCC | 0 | test |
| 144212935 | CCCCCCCCCCCSCC(=O)O | 0 | train |
| 144212934 | CCCCCCCCC1CCC(=O)O1 | 1 | train |
| 144212933 | CC1=CC=C(C=C1)OC(=O)C2=CC=CC=C2O | 0 | validation |

| | | | |
|---|---|---|---|
| 144212932 | CCCCCCCOC(=O)C | 0 | test |
| 144212931 | CCCCCCCCCCCCCCCC[N+](C)(C)CC.[Br-] | 0 | train |
| 144212930 | CCC1=NC=CN=C1C | 0 | train |
| 144212929 | COC1=CC(=CC=C1)Br | 0 | validation |
| 144212928 | C[C@]12CCC(=O)C=C1[C@H](C[C@@H]3[C@@H]2[C@H](C[C@]4([C@H]3C[C@@H]5[C@]4(OC(O5)(C)C)C(=O)CO)C)O)F | 0 | test |
| 144212927 | CC1=CC=C(C=C1)C2=CC=CC=C2 | 0 | train |
| 144212926 | CSCCCN=C=S | 0 | train |
| 144212925 | CCCCCCCCCCCC[N+](C)(C)CCCCCCCCCCCC.[Br-] | 1 | validation |
| 144212924 | CCCC(=O)OCCC1=CC=CC=C1 | 0 | test |
| 144212923 | CC(=O)NCCCS(=O)(=O)[O-].CC(=O)NCCCS(=O)(=O)[O-].[Ca+2] | 0 | train |
| 144212922 | CCCCCCCCCOC(=O)CCCCC(=O)OCCCCCCC | 0 | train |
| 144212921 | C1=CN(C=C1)CC2=CC=CO2 | 0 | validation |
| 144212920 | CCCCCCCCCCOC(=O)C1=CC=CC=C1C(=O)OCCCCCCCC | 0 | test |
| 144212919 | CC/C=C¥CCCCO | 0 | train |
| 144212918 | CC1=C(C(=O)C=CO1)OC(=O)C(C)C | 0 | train |
| 144212917 | CC1C(=O)CCO1 | 0 | validation |
| 144212916 | CCCCCCOC(=O)C(C)CC | 0 | test |
| 144212915 | CC(=O)OC(C1=CC=CC=C1)C(Cl)(Cl)Cl | 0 | train |
| 144212913 | CC1=NN(C(=O)C1N=NC2=CC=CC=C2)C3=CC=C(C=C3)S(=O)(=O)[O-].[Na+] | 0 | validation |
| 144212912 | C1C2=CC=CC=C2C3=C1C=C(C=C3)O | 1 | test |
| 144212911 | CCCCC1=CC=C(C=C1)O | 0 | train |
| 144212910 | C[C@H]1C[C@H]2[C@@H]3CC[C@@]([C@]3(C[C@@H]([C@@]2([C@@]4(C1=CC(=O)C=C4)C)F)O)C)(C(=O)C)O | 0 | train |
| 144212909 | C1CCC2=NC=CN=C2C1 | 0 | validation |
| 144212908 | CC1=CN=C(C=N1)SC | 0 | test |
| 144212907 | CCC(=O)OC(C)(CCC=C(C)C)C=C | 0 | train |
| 144212906 | CC1=C(C=CO1)SSC2=C(OC=C2)C | 0 | train |
| 144212905 | CCCCCCCCC(=O)OCC | 0 | validation |
| 144212904 | CC(=O)OCCCC1=CC=CC=C1 | 0 | test |
| 144212903 | C[C@@H]1C[C@H]2[C@@H]3CCC4=CC(=O)C=C[C@@]4([C@]3([C@H](C[C@@]2([C@]1(C(=O)COP(=O)([O-])[O-])O)C)O)F)C.[Na+].[Na+] | 0 | train |
| 144212902 | C1=CC(=CC=C1CC2=CC=C(C=C2)N=C=O)N=C=O | 0 | train |
| 144212901 | C(Cl)C(C(C(C(F)(F)F)(F)F)(F)F)(F)F | 0 | validation |
| 144212900 | C[C@H]1C[C@H](CC(C1)(C)C)O | 0 | test |
| 144212899 | CCCCCC/C=C/C(=O)OC | 0 | train |
| 144212898 | CC1(SC(SC(S1)(C)C)(C)C)C | 0 | train |
| 144212897 | CC(C)C1=CC=C(C=C1)C#N | 0 | validation |
| 144212896 | CC1CCC(C(=O)C1)C(C)(C)S | 0 | test |
| 144212895 | CCCCCCCC(=O)OCCCCCC | 0 | train |
| 144212894 | C=CC(=O)OCCOC(=O)C=C | 0 | train |
| 144212893 | CCCCC(CC)COC(=O)/C=C/C1=CC=C(C=C1)OC | 0 | validation |
| 144212892 | CC1=CC(=C(C=C1)C(C)C)OC | 0 | test |
| 144212891 | CCCCCCOC(=O)CCCCC | 0 | train |
| 144212890 | CC/C=C¥CCOC(=O)/C(=C/C)/C | 0 | train |
| 144212889 | CCCCCCCCCCCOC(=O)C | 0 | validation |
| 144212888 | CC(=O)OC1C(C2CCC1(C2)C)(C)C | 0 | test |
| 144212887 | CCCCCC1=CC=CC(=O)O1 | 0 | train |
| 144212886 | CCCCC1CCC(=O)O1 | 0 | train |
| 144212885 | CC(=O)OC(C)(C)CCCC(=C)C=C | 0 | validation |
| 144212884 | CC1=C(SC(=N1)C)C(=O)C | 0 | test |
| 144212883 | CCC(=O)C(=O)CC | 0 | train |
| 144212882 | CC1CC(C(=O)C1=O)C | 0 | train |
| 144212881 | CC(=O)OC1=C(C=C(C=C1)[N+](=O)[O-])CCl | 0 | validation |
| 144212880 | C1CN(CCN1CCOCCO)C(C2=CC=CC=C2)C3=CC=C(C=C3)Cl.Cl | 0 | test |
| 144212879 | CC1=C(C(=CC=C1)C(C)(C)C)O | 0 | train |
| 144212878 | CCCCOC(=O)CCCCCCCCC=C | 0 | train |
| 144212877 | C1=CC=C2C=C(C=CC2=C1)OC(=O)C3=CC=CC=C3N | 1 | validation |
| 144212876 | CCCCCC=CCC(=O)OC | 0 | test |
| 144212875 | CC=CC=CCOC(=O)C(C)C | 0 | train |
| 144212874 | CC(C)COC(=O)C=CC1=CC=CC=C1 | 1 | train |
| 144212873 | C1CCCCCOCCCCOC(=O)CCCC1 | 0 | validation |

| | | | |
|---|---|---|---|
| 144212872 | CCCCCCC(CCOC(=O)C)O | 0 | test |
| 144212871 | CCC(=O)OCC1=CC=C(C=C1)OC | 0 | train |
| 144212870 | CC=C(C)C(=O)OCCC(C)CCC=C(C)C | 0 | train |
| 144212869 | CCCC1=CC=CC=C1O | 0 | validation |
| 144212868 | CC1=CC(=CC(=C1)OC)O | 0 | test |
| 144212867 | CC1=CCC(C=C1)C(C)C | 0 | train |
| 144212866 | C[C@H](CCC(=O)NCC(=O)O)[C@H]1CC[C@@H]2[C@@]1([C@H](C[C@H]3[C@H]2[C@@H](C[C@H]4[C@@]3(CC[C@H](C4)O)C)O)O)C | 0 | train |
| 144212865 | C/C=C/C=C/CO | 0 | validation |
| 144212864 | CC1=CCC(CC1=O)C(=C)C | 0 | test |
| 144212863 | CCCCCCCC#N | 0 | train |
| 144212862 | CC(=CCC/C(=C/COC=O)/C)C | 0 | train |
| 144212861 | C[C@]12CCCC([C@@H]1CC[C@@]3([C@@H]2CC(=O)O3)C)(C)C | 0 | validation |
| 144212859 | CC(C)C(=O)OC1=C(C=C(C=C1)C=O)OC | 0 | train |
| 144212858 | CC1=C(C(=O)CO1)O | 0 | train |
| 144212857 | CC(CCCC(C)(C)O)CC(OC)OC | 0 | validation |
| 144212856 | CCOC1=C(C=C(C=C1)/C=C¥C)O | 0 | test |
| 144212854 | C=CCCCCCCCCO | 0 | train |
| 144212853 | CCCCCCCCCOC(=O)C | 0 | validation |
| 144212852 | COC1=CC=C(C=C1)COC=O | 0 | test |
| 144212851 | CSC1=NC=CN=C1 | 0 | train |
| 144212850 | CC(C)(CC1=CC=CC=C1)O | 0 | train |
| 144212849 | C1CCCCCC(=O)CCCC=CCCCC1 | 1 | validation |
| 144212848 | CC(C)CCCCCCC1=CC=C(C=C1)OCCOCCO | 0 | test |
| 144212847 | C1=C(C(=C(C(=C1[N+](=O)[O-])Cl)[N+](=O)[O-])Cl)C(F)(F)F | 1 | train |
| 144212846 | C[C@@H]1CC[C@H]([C@H](C1)O)C(=C)C | 0 | train |
| 144212845 | CCCCCCCCCOC(=O)C=C | 0 | validation |
| 144212844 | CCCCCC(=O)OCCC1=CC=CC=C1 | 0 | test |
| 144212843 | C[C@]12CC[C@](C[C@H]1C3=CC(=O)[C@@H]4[C@]5(CC[C@@H](C([C@@H]5CC[C@]4([C@@]3(CC2)C)C)(C)C)O[C@@H]6[C@@H]([C@H]([C@@H]([C@H](O6)C(=O)O)O)O)O[C@H]7[C@@H]([C@H]([C@@H]([C@H](O7)C(=O)[O-])O)O)O)C)(C)C(=O)O.[NH4+].O.O.O | 0 | train |
| 144212842 | CC(C)(C)C(=O)/C=C/C1=CC=C(C=C1)Cl | 1 | train |
| 144212841 | CC1=C(C(CCC1)(C)C)C=O | 0 | validation |
| 144212840 | [O-]Cl(=O)=O.[K+] | 0 | test |
| 144212839 | CC1CCC2=NC=CN=C12 | 0 | train |
| 144212838 | COC1=CC=C(C=C1)CO | 0 | train |
| 144212837 | CC1(COP(OC1)OC2=CC=CC=C2)C | 0 | validation |
| 144212836 | C1=CC(=C(C(=C1)F)C#N)F | 0 | test |
| 144212835 | CC(C(=O)C)O | 0 | train |
| 144212834 | CCCCCC(C)O | 0 | train |
| 144212833 | C1CC(CC=C1)C2=CC=CC=C2 | 0 | validation |
| 144212832 | CC(C)COC(=O)C1=CC=CC=C1O | 0 | test |
| 144212831 | C1=CC(=C(C=C1OC2=C(C=C(C=C2I)C[C@@H](C(=O)[O-])N)I)I)O.[Na+] | 1 | train |
| 144212830 | CC(CCC(=O)O)(C#N)N=NC(C)(CCC(=O)O)C#N | 0 | train |
| 144212829 | CC(C1CCCCC1)O | 0 | validation |
| 144212828 | CCCCCCCCCNC | 0 | test |
| 144212827 | CC(=O)OCCN(CCOC(=O)C)C1=CC=C(C=C1)N=NC2=C(C=C(C=C2)[N+](=O)[O-])C#N | 1 | train |
| 144212826 | C(=O)(N)[O-].[NH4+] | 0 | train |
| 144212825 | CN(C)CC(C1=CC=C(C=C1)OC)C2(CCCCC2)O.Cl | 0 | validation |
| 144212824 | C1(=C(C(=C(C(=C1F)F)Br)F)F)F | 0 | test |
| 144212823 | CC1=CC2=C(C=C1)NCCC2 | 1 | train |
| 144212822 | CCCCCCCCCCCCCCCC(=O)OCC | 0 | train |
| 144212821 | CC(C)CC(=O)OCC1=CC=CC=C1 | 0 | validation |
| 144212820 | C1=CC=C2C=NC=CC2=C1 | 0 | test |
| 144212819 | CCCC(=O)C(CC)SC1=C(OC=C1)C | 0 | train |
| 144212818 | O.O.Cl[In](Cl)Cl | 0 | train |
| 144212817 | CC1C(=C(C(=O)O1)O)C | 0 | validation |
| 144212816 | CC(COCCO)OCCO | 0 | test |
| 144212815 | CCC(C)C(=O)OCCC1=CC=CC=C1 | 0 | train |
| 144212814 | CC(C)C1CCC(C1(CN2C=NC=N2)O)CC3=CC=C(C=C3)Cl | 0 | train |
| 144212813 | C1=CC=C2C(=C1)C=NN=C2NN.Cl | 1 | validation |
| 144212812 | CCCCCC(CC)OC(=O)CCCOC1=C(C=C(C=C1)Cl)Cl | 0 | test |

| | | | |
|---|---|---|---|
| 144212811 | C1=CC=C(C=C1)CCC=O | 0 | train |
| 144212810 | CC(=O)CCC1=CC(=C(C=C1)O)OC | 0 | train |
| 144212809 | C/C=C(¥C)/C(=O)OC/C=C(¥C)/CCC=C(C)C | 0 | validation |
| 144212808 | CCCCCCOC(=O)C(C)C | 0 | test |
| 144212807 | CCCCCCCCCCC=CC#N | 0 | train |
| 144212806 | CC1=C(C(=CC=C1)C)N(C(C)C(=O)OC)C(=O)C2=CC=CO2 | 1 | train |
| 144212805 | CCCC(=O)OCCC(C)CCC=C(C)C | 0 | validation |
| 144212804 | CC(C)(CO)[C@H](C(=O)NCCC(=O)[O-])O.[Na+] | 0 | test |
| 144212803 | CC(=O)SCC1=CC=CO1 | 0 | train |
| 144212802 | C1=CC=C(C=C1)C=CCOC(=O)C=CC2=CC=CC=C2 | 1 | train |
| 144212801 | C1CCN(CC1)CN2CCCCC2 | 0 | validation |
| 144212800 | CC1=CC=CC=C1S | 0 | test |
| 144212799 | CC(=O)O[Pb](C1=CC=CC=C1)(C2=CC=CC=C2)C3=CC=CC=C3 | 1 | train |
| 144212798 | C[Si](O[Si](C)(C)C)O[Si](C)(C)C | 0 | train |
| 144212797 | C1=CC=C2C(=C1)N=NN2O | 0 | validation |
| 144212796 | CC(C)OC(=O)C(CC(=O)O)(CC(=O)O)O | 1 | test |
| 144212795 | C1=CSC(=C1)SSC2=CC=CS2 | 1 | train |
| 144212794 | CCCCCCCCCCCC(=O)N(CC)CC | 1 | train |
| 144212793 | CC1=NC=C(C(=C1O)CO)CO.Cl | 0 | validation |
| 144212792 | CC(=CC(=O)O)C | 0 | test |
| 144212791 | CC/C=C¥CC/C=C/CO | 0 | train |
| 144212790 | CC(C)N1C(=O)C2=CC=CC=C2C1=O | 0 | train |
| 144212789 | C1=CC2=NNN=C2C=C1Cl | 0 | validation |
| 144212788 | CO[Si](CCCNC1=CC=CC=C1)(OC)OC | 0 | test |
| 144212787 | CC1(CCC(=O)[C@@H]2C13CCC(C3)C2(C)C)C | 0 | train |
| 144212786 | C1CN(CCN1CC2=CC3=C(C=C2)OCO3)C(=O)COC4=CC=C(C=C4)Cl.Cl | 1 | train |
| 144212785 | CC(C)(C)NC[C@@H](COC1=NSN=C1N2CCOCC2)O.C(=C¥C(=O)O)¥C(=O)O | 0 | validation |
| 144212784 | C[C@H]1C[C@H]2[C@@H]3CCC4=CC(=O)C=C[C@@]4([C@]3([C@H](C[C@@]2([C@]1(C(=O)COP(=O)([O-])[O-])O)C)O)F)C.[Na+].[Na+] | 0 | test |
| 144212783 | C=CC1=CC=C(C=C1)S(=O)(=O)[O-].[Na+] | 0 | train |
| 144212782 | C1CCCCCCCC(=O)CCCCCC1 | 1 | train |
| 144212781 | [N+](=O)([O-])[O-].[N+](=O)([O-])[O-].O.O.O.O.O.O.[Mg+2] | 0 | validation |
| 144212780 | CC1=CCCC(C1/C=C/C(=O)CCC=C)(C)C | 1 | test |
| 144212779 | CCCCC1CCCCC(=O)O1 | 0 | train |
| 144212778 | CC(C)CCOC(=O)C1=CC=CC=C1 | 0 | train |
| 144212777 | CCC(C)(C)C1CCC(=O)CC1 | 0 | validation |
| 144212776 | CCC(C)C1N=C(C(S1)C)C | 0 | test |
| 144212775 | CC(C)CC=C(C=O)C1=CC=CC=C1 | 0 | train |
| 144212774 | CCOC(=O)CC1(OCC(O1)C)C | 0 | train |
| 144212773 | CC(=CCC/C(=C¥CCC(C)(C=C)O)/C)C | 1 | validation |
| 144212772 | CC(CN(CCN(CC(C)OCCO)CC(C)OCCO)CC(C)OCCO)OCCO | 0 | test |
| 144212771 | CC(C)C1CCC(CC1)O | 0 | train |
| 144212770 | C1=C(C(=C(C(=C1Br)Br)Br)Br)OC2=CC(=C(C(=C2Br)Br)Br)Br | 0 | train |
| 144212769 | CC(=O)OCCC1CCCCC1 | 0 | validation |
| 144212768 | C(=O)(N)N.OO | 0 | test |
| 144212767 | CCCCCCCCOCCC#N | 0 | train |
| 144212766 | COC(=O)C1=CC(=C(C=C1)C(=O)OC)C(=O)OC | 0 | train |
| 144212765 | C1=CC=C2C(=C1)C=C(O2)C=O | 0 | validation |
| 144212764 | C1=CC=C2C(=C1)C(=NS2(=O)=O)[O-].C1=CC=C2C(=C1)C(=NS2(=O)=O)[O-].O.[Ca+2] | 0 | test |
| 144212763 | CCCCCCC#CC(=O)OC | 0 | train |
| 144212762 | CCCCCCOC(=O)CCC | 0 | train |
| 144212761 | CCC(=O)OC/C=C/C1=CC=CC=C1 | 0 | validation |
| 144212760 | CCCCCCOC(=O)CC | 0 | test |
| 144212759 | CN=C(NCC1CCOC1)N[N+](=O)[O-] | 0 | train |
| 144212758 | CC1=C(C=CC(=C1C2=NOCC2)S(=O)(=O)C)C(=O)C3=CNN(C3=O)C | 0 | train |
| 144212757 | CCCO | 0 | validation |
| 144212756 | CNC | 0 | test |
| 144212755 | C=O | 0 | train |
| 144212754 | C[Si](=O)C | 0 | train |
| 144212753 | CC1=CC=C(C=C1)S(=O)(=O)[N-]Cl.O.O.O.[Na+] | 0 | validation |
| 144212752 | COC1=CC(=NC(=N1)OC2=C(C(=CC=C2)OC3=NC(=CC(=N3)OC)OC)C(=O)[O-])OC.[Na+] | 0 | test |
| 144212751 | C1C(COC1(CN2C=NC=N2)C3=C(C=C(C=C3)Cl)Cl)Br | 1 | train |

| | | | |
|---|---|---|---|
| 144212750 | COC1=NC=C(C2=NC(=NN21)S(=O)(=O)NC3=C(C=CC=C3F)F)F | 0 | train |
| 144212749 | CC1(CSC(CS1)(C)O)O | 0 | validation |
| 144212748 | CC1=CC=CC=C1C(=O)NC2=CC(=CC=C2)OC(C)C | 1 | test |
| 144212747 | CCOC(=O)OC1=C(C(=O)NC12CCC(CC2)OC)C3=C(C=CC(=C3)C)C | 0 | train |
| 144212746 | C(C(=O)O)F | 0 | train |
| 144212745 | C1=CC(=CC=C1O)O[C@H]2[C@@H]([C@H]([C@@H]([C@H](O2)CO)O)O)O | 0 | validation |
| 144212744 | C[C@]12CC[C@H]3[C@H]([C@@H]1CC[C@]2(C#C)O)CCC4=CC(=O)CC[C@H]34 | 0 | test |
| 144212743 | CC(C)NCC(COC1=CC=C(C=C1)CC(=O)N)O | 0 | train |
| 144212742 | C1=CC=C(C=C1)C=C(C2=CC=CC=C2)C3=CC=CC=C3 | 1 | train |
| 144212741 | C1=CC(=CC(=C1)NC(=O)C2=CC(=CC(=C2O)Br)Br)C(F)(F)F | 0 | validation |
| 144212740 | C1(=NC(=O)N(C(=O)N1Cl)Cl)[O-].[K+] | 0 | test |
| 144212739 | CC(C)CCCCCOC(=O)CCCCC(=O)OCCCCCC(C)C | 0 | train |
| 144212738 | CC(COP(=O)(OCC(C)Cl)OCC(C)Cl)Cl | 1 | train |
| 144212737 | CNC(=O)OC1=CC=CC=C1C2OCCO2 | 0 | validation |
| 144212736 | CC1=C2C(=CC=C1)SC3=NN=CN23 | 1 | test |
| 144212735 | CCCCCCCCCCCOC(=O)C1=CC=CC=C1C(=O)OCCCCCCCCCCC | 0 | train |
| 144212734 | CC(C)C1=C(C(=C(N1CC[C@H](C[C@H](CC(=O)O)O)O)C2=CC=C(C=C2)F)C3=CC=CC=C3)C(=O)NC4=CC=CC=C4 | 0 | train |
| 144212733 | C1=CC(=C(C=C1N)[N+](=O)[O-])NCCO | 0 | validation |
| 144212732 | CC(C)(C)NC(=O)[C@@H]1CN(CCN1C[C@H](C[C@@H](CC2=CC=CC=C2)C(=O)N[C@@H]3[C@@H](CC4=CC=CC=C34)O)O)CC5=CN=CC=C5.OS(=O)(=O)O | 0 | test |
| 144212731 | CC1=CC=C(C=C1)C(=O)C2=CC(=C(C(=C2)O)O)[N+](=O)[O-] | 1 | train |
| 144212730 | CCCC1=C(C=CC(=C1O)C(=O)C)OCCCOC2=CC=C(C=C2)OCC(=O)O | 0 | train |
| 144212729 | CCCC1=C(C=CC(=C1O)C(=O)C)OCCCCC2=NNN=N2 | 0 | validation |
| 144212728 | CC1=C(C=CC(=C1)SCC2=C(N=C(S2)C3=CC(=C(C=C3)C(F)(F)F)F)C)OCC(=O)O | 0 | test |
| 144212727 | CC(=CC[C@@H]1[C@](O1)(C)[C@H]2[C@@H]([C@@H](CC[C@]23CO3)OC(=O)/C=C/C=C/C=C/C=C/C(=O)O)OC)C | 0 | train |
| 144212726 | CCCCCCCCN1C(=O)C(=C(S1)Cl)Cl | 1 | train |
| 144212725 | CC1=C(C(=CC(=C1)CCC(=O)OCCOCCOCCOC(=O)CCC2=CC(=C(C(=C2)C(C)(C)C)O)C)C(C)(C)C)O | 0 | validation |
| 144212724 | CC1=CC2=C(C=C1C(=C)C3=CC=C(C=C3)C(=O)O)C(CCC2(C)C)(C)C | 1 | test |
| 144212723 | C[C@]12CC[C@H]3[C@H]([C@@H]1CC[C@@H]2O)CC[C@]45[C@@]3(CC(=C([C@H]4O5)O)C#N)C | 1 | train |
| 144212722 | C1=CC=C2C(=C1)C(=O)C3=C(C2=O)SC(=C(S3)C#N)C#N | 1 | train |
| 144212721 | CCCCC(CC)COC(=O)C1=C(C(=C(C(=C1Br)Br)Br)Br)C(=O)OCC(CC)CCCC | 0 | validation |
| 144212720 | CC(C)COC(=O)COC1=C(C=C(C=C1)Cl)Cl | 0 | test |
| 144212719 | C[C@]12CC[C@H]3[C@H]([C@@H]1CC[C@@H]2C(=O)CO)CCC4=CC(=O)CC[C@]34C | 0 | train |
| 144212718 | CC1(O[C@@H]2CO[C@@]3([C@H]([C@@H]2O1)OC(O3)(C)C)COS(=O)(=O)N)C | 0 | train |
| 144212717 | C1=CC2=C(C(=C1)O)C(=O)C3=C(C=C(C=C3C2=O)CO)O | 0 | validation |
| 144212716 | CC(=O)O[C@H]1[C@H]([C@@H]2[C@]([C@H](CCC2(C)C)O)([C@@]3([C@@]1(O[C@@](CC3=O)(C)C=C)C)O)C)O | 0 | test |
| 144212715 | CN(C)CCOC(=O)COC1=CC=C(C=C1)Cl.Cl | 1 | train |
| 144212714 | C[C@@H]1CCC=C2[C@]1(C[C@@H](CC2)C(=C)C)C | 0 | train |
| 144212713 | CC(C)CCCC(=O)O | 0 | validation |
| 144212712 | C1(=O)NC(=O)N(C(=O)N1Cl)Cl | 0 | test |
| 144212711 | CCC(C)(CCC(C)C)C(=O)[O-].CCC(C)(CCC(C)C)C(=O)[O-].[Zn+2] | 0 | train |
| 144212710 | CC(=C[C@@H]1[C@H](C1(C)C)C(=O)OCC2=COC(=C2)CC3=CC=CC=C3)C | 1 | train |
| 144212709 | CC/C=C¥CC/C=C/C(OCC)OCC | 0 | validation |
| 144212708 | CC(CCCC(C)(C)O)C=C | 0 | test |
| 144212707 | C(=C/N=O)¥NO | 0 | train |
| 144212706 | CC1=C(C(=CC=C1)S(=O)(=O)[O-])C.[NH4+] | 0 | train |
| 144212705 | CCCCCCCCCC(CC)C1=CC=C(C=C1)S(=O)(=O)[O-].CCCCCCCCCC(CC)C1=CC=C(C=C1)S(=O)(=O)[O-].[Ca+2] | 0 | validation |
| 144212704 | CCC/C(=C¥1/C(=O)CC(C(=C1[O-])C(=O)OC)(C)C)/NOCC=C.[Na+] | 0 | test |
| 144212703 | CC1=C(C=CC(=C1)Cl)OCC(=O)[O-].[Na+] | 0 | train |
| 144212702 | C[C@@H]1CC(=O)C=C2[C@]1(C[C@@H](CC2)C(=C)C)C | 1 | train |
| 144212701 | CCCCCCCCCCCCCCCCCCOS(=O)(=O)[O-].[Na+] | 0 | validation |
| 144212700 | COC(=O)NC1=NC2=C(N1)C=C(C=C2)S(=O)C3=CC=CC=C3 | 0 | test |
| 144212699 | CS(=O)(=O)C1=C(C(=C(C=C1)C(=O)C2C(=O)CCCC2=O)Cl)COCC(F)(F)F | 0 | train |
| 144212698 | CN(C)C1CSSSC1.C(=O)(C(=O)O)O | 0 | train |
| 144212697 | C[C@@]1([C@H]2C[C@@H]([C@H]3[C@@H](C(=O)C(=C([C@]3(C(=O)C2=C(C4=C1C=CC=C4O)O)O)O)O)C(=O)N)N(C)C)O)O | 0 | validation |

| | | | |
|---|---|---|---|
| 144212696 | CCCCCCCCCC[N+](C)(CCCCCCCCCC)CCC[Si](OC)(OC)OC.[Cl-] | 0 | test |
| 144212695 | CC(=C)C1CCC(CC1)(C)O | 0 | train |
| 144212694 | CC(C)COC(=O)CC(C(=O)OCC(C)C)S(=O)(=O)[O-].[Na+] | 0 | train |
| 144212693 | C1=CC2=C(C(=C1)O)N=CC=C2.C(C(=O)O)C(CC(=O)O)(C(=O)O)O | 1 | validation |
| 144212692 | CC1(C(=O)N(C(=O)N1Br)Cl)C | 0 | test |
| 144212691 | CCCCCCCCCCCCOCCOS(=O)(=O)[O-].[Na+] | 0 | train |
| 144212690 | CC(C)C1=C(C(=C(N1CC[C@H](C[C@H](CC(=O)[O-])O)O)C2=CC=C(C=C2)F)C3=CC=CC=C3)C(=O)NC4=CC=CC=C4.CC(C)C1=C(C(=C(N1CC[C@H](C[C@H](CC(=O)[O-])O)O)C2=CC=C(C=C2)F)C3=CC=CC=C3)C(=O)NC4=CC=CC=C4.[Ca+2] | 0 | train |
| 144212689 | CN/C(=C¥[N+](=O)[O-])/NCCSCC1=CC=C(O1)CN(C)C | 0 | validation |
| 144212688 | C1CCC2(C1)CC(=O)N(C(=O)C2)CCCCN3CCN(CC3)C4=NC=CC=N4 | 0 | test |
| 144212687 | CN(C)C(=N)N=C(N)N | 0 | train |
| 144212686 | C1CCN(CC1)CCOC2=CC=C(C=C2)C(=O)C3=C(SC4=C3C=CC(=C4)O)C5=CC=C(C=C5)O.Cl | 1 | train |
| 144212685 | C1=CC=C(C=C1)C(COC(=O)N)COC(=O)N | 0 | validation |
| 144212684 | CN(CCOC1=CC=C(C=C1)CC2C(=O)NC(=O)S2)C3=CC=CC=N3.C(=C¥C(=O)O)¥C(=O)O | 1 | test |
| 144212683 | C1CN(CCN1CCOCCO)C2=NC3=CC=CC=C3SC4=CC=CC=C42.C1CN(CCN1CCOCCO)C2=NC3=CC=CC=C3SC4=CC=CC=C42.C(=C/C(=O)O)¥C(=O)O | 1 | train |
| 144212682 | CC1=C(C(C(=C(N1)C)C(=O)OCC(C)C)C2=CC=CC=C2[N+](=O)[O-])C(=O)OC | 1 | train |
| 144212681 | C[C@]12C[C@@H]([C@]3([C@H]([C@@H]1C[C@@H]4[C@]2(OC(O4)(C)C)C(=O)CO)C[C@@H](C5=CC(=O)C=C[C@@]53C)F)F)O | 0 | validation |
| 144212680 | CC(=O)O.CC(=O)O.C1=CC(=CC=C1N/C(=N/C(=NCCCCCCN=C(/N=C(/NC2=CC=C(C=C2)Cl)¥N)N)N)/N)Cl | 1 | test |
| 144212679 | COC1=C(C(=NC=C1)CS(=O)C2=NC3=C([N-]2)C=CC(=C3)OC(F)F)OC.[Na+] | 0 | train |
| 144212678 | CNC(=S)[S-].[Na+] | 0 | train |
| 144212677 | CC1=C[C@@H]2[C@H](CC[C@]3([C@H]2CC[C@@]3(C(=O)C)OC(=O)C)C)[C@@]4(C1=CC(=O)CC4)C | 0 | validation |
| 144212676 | CC(C)CCO | 0 | test |
| 144212675 | CN(C)N | 0 | train |
| 144212674 | C/C=C(¥C)/C(=O)C | 0 | train |
| 144212673 | CCC(C)(C)O | 0 | validation |
| 144212672 | CN(C)C | 0 | test |
| 144212671 | CN1CCC[C@H]1C2=CN=CC=C2.CN1CCC[C@H]1C2=CN=CC=C2.OS(=O)(=O)O | 0 | train |
| 144212670 | CC(C1=CC(=C(C=C1)C2=CC=CC=C2)F)C(=O)O | 0 | train |
| 144212669 | C1CN(CCN1)C2=NC3=CC=CC=C3OC4=C2C=C(C=C4)Cl | 0 | validation |
| 144212668 | CC[N+](CC)(CC1=CC=CC=C1)CC(=O)NC2=C(C=CC=C2C)C.C1=CC=C2C(=C1)C(=NS2(=O)=O)[O-] | 0 | test |
| 144212667 | CC1=CC=C(C=C1)OC(=O)C | 0 | train |
| 144212666 | CCCC(=O)C(=O)C | 0 | train |
| 144212665 | COC1=CC(=CC=C1)OC | 0 | validation |
| 144212664 | CCCCCCOC(=O)C1=CC=CC=C1 | 0 | test |
| 144212663 | CCC(=O)OC(C)C1=CC=CC=C1 | 0 | train |
| 144212662 | CCCC(=O)C1=CC=CC=C1 | 0 | train |
| 144212661 | CC1=CC(CC(C1)(C)C)O | 0 | validation |
| 144212660 | C1CNCCN1.Cl.Cl | 0 | test |
| 144212659 | C1=CC=C(C=C1)N/N=C/2¥C(=CC3=CC(=C(C(=C3C2=O)N)N=NC4=CC=C(C=C4)C5=CC=C(C=C5)N=NC6=C(C=C(C=C6)N)N)S(=O)(=O)[O-])S(=O)(=O)[O-].[Na+].[Na+] | 0 | train |
| 144212658 | CC(CO)C1=CC=CC=C1 | 0 | train |
| 144212657 | CC(=O)O[C@H](CC(=O)[O-])C[N+](C)(C)C.Cl | 0 | validation |
| 144212656 | C1=CC=C(C=C1)CN(CC2=CC=CC=C2)CC3=CC=CC=C3 | 1 | test |
| 144212655 | C1=CC=C(C=C1)C2(C(=O)[N-]C(=O)N2)C3=CC=CC=C3.[Na+] | 1 | train |
| 144212654 | CCOC(=O)CCC1=CC=CC=C1 | 0 | train |
| 144212653 | CCNC(=O)C1CC(CCC1C(C)C)C | 0 | validation |
| 144212652 | C1=CC=C(C=C1)C2=CC(=O)C3=C(C=C(C=C3O2)O)O | 1 | test |
| 144212651 | COC1=CC=CC=C1C(=O)OC | 0 | train |
| 144212650 | CCCCCC(=O)O[C@@]1(CC[C@@H]2[C@@]1(CC[C@H]3[C@H]2CCC4=CC(=O)CC[C@]34C)C)C(=O)C | 1 | train |
| 144212649 | CCC1=CC=C(C=C1)C=O | 0 | validation |
| 144212648 | CC(C)[N+](C)(CCOC(=O)C1C2=CC=CC=C2OC3=CC=CC=C13)C(C)C.[Br-] | 0 | test |
| 144212647 | C1=CC=C(C=C1)COC(=O)C2=CC=C(C=C2)O | 1 | train |
| 144212646 | C1=CC=C(C=C1)CCC(=O)O | 0 | train |
| 144212645 | CCCCCCC1CCCC(=O)O1 | 0 | validation |

| | | | |
|---|---|---|---|
| 144212644 | CC(C)COC(=O)C1=CC=CC=C1 | 0 | test |
| 144212643 | CCOC(=O)CC(=O)C1=CC=CC=C1 | 0 | train |
| 144212642 | C(NC(=O)NC1C(=O)NC(=O)N1CO)NC(=O)NC2C(=O)NC(=O)N2CO | 0 | train |
| 144212641 | C[C@]12CCC(=O)C=C1CC[C@@H]3[C@@H]2CC[C@]4([C@H]3CC[C@]4(C#C)O)C | 0 | validation |
| 144212640 | CC(CC=O)C1=CC=CC=C1 | 0 | test |
| 144212639 | C1=CC=C(C=C1)[Bi](C2=CC=CC=C2)(C3=CC=CC=C3)(Cl)Cl | 1 | train |
| 144212638 | CC1=C(SC=[N+]1CC2=CN=C(N=C2N)C)CCO.[N+](=O)([O-])[O-] | 0 | train |
| 144212637 | CCOC(=O)N1C=CN(C1=S)C | 0 | validation |
| 144212636 | [Li+].C(C(C(C(F)(F)S(=O)(=O)[O-])(F)F)(F)F)(F)F)(C(C(C(F)(F)F)(F)F)(F)F)(F)F | 0 | test |
| 144212635 | CC(C)NC(=O)C1=CC=C(C=C1)CNNC.Cl | 0 | train |
| 144212634 | CC1=C(C=CN=C1CS(=O)C2=NC3=CC=CC=C3[N-]2)OCCCOC.[Na+] | 1 | train |
| 144212633 | CCCN[C@H]1CCC2=C(C1)SC(=N2)N.O.Cl.Cl | 0 | validation |
| 144212632 | [O-]S(=O)(=O)SSS(=O)(=O)[O-].[K+].[K+] | 0 | test |
| 144212631 | CC1=CN=C(C(=N1)C)C | 0 | train |
| 144212630 | CC1=C(N=C(C(=N1)C)C)C | 0 | train |
| 144212629 | CCCCCCC1(CCC(=O)O1)C | 0 | validation |
| 144212628 | F[Si-2](F)(F)(F)(F)F.[Na+].[Na+] | 0 | test |
| 144212627 | CCCCOC(=O)C(C)CC | 0 | train |
| 144212626 | CC(=O)C1=NC=CN=C1 | 0 | train |
| 144212625 | CCC1=CC(=C(C=C1)O)OC | 0 | validation |
| 144212624 | C[C@]12CCCC([C@@H]3[C@H]1CC[C@@H]3C2=C)(C)C | 0 | test |
| 144212623 | CC1CC=C(C(C1(C)C)/C=C/C(=O)C)C | 0 | train |
| 144212622 | C[C@@H]1CC[C@@H]2[C@]13CC=C(C(C3)C2(C)C)C | 0 | train |
| 144212621 | CC(=CCC=C(C)C=C)C | 0 | validation |
| 144212620 | C([C@@H]1[C@@H]2[C@@H]([C@H]([C@H](O1)O[C@@H]3[C@H](O[C@@H]([C@@H]([C@H]3O)O)O[C@@H]4[C@H](O[C@@H]([C@@H]([C@H]4O)O)O[C@@H]5[C@H](O[C@@H]([C@@H]([C@H]5O)O)O[C@@H]6[C@H](O[C@@H]([C@@H]([C@H]6O)O)O[C@@H]7[C@H](O[C@@H]([C@@H]([C@H]7O)O)O[C@@H]8[C@H](O[C@@H]([C@@H]([C@H]8O)O)O[C@@H]9[C@H](O[C@@H](O2)[C@@H]([C@H]9O)O)CO)CO)CO)CO)CO)CO)CO)O)O)O | 0 | test |
| 144212619 | CCCCCCC/C=C/C=O | 0 | train |
| 144212618 | CC1=CC[C@@H](C=C1)C(C)C | 0 | train |
| 144212617 | CCCSC1=CC2=C(C=C1)N=C(N2)NC(=O)OC | 1 | validation |
| 144212616 | CC(C)C1=CC2=C(C=C1)[C@]3(CCC[C@@]([C@@H]3CC2)(C)CN)C.CC(=O)O | 1 | test |
| 144212615 | CC1=CCC(CC1)C(C)(C)O | 0 | train |
| 144212614 | COC1=CC=CC=C1OC | 0 | train |
| 144212613 | CCCCCCCCCOC(=O)C | 0 | validation |
| 144212612 | CC1=CC[C@H](CC1)C(=C)C | 0 | test |
| 144212611 | CCCCCCCCCCC(CCCCCCCC)CO | 0 | train |
| 144212610 | C=CCCCCCCCCC=O | 0 | train |
| 144212609 | CCOC(=O)CCC(=O)C | 0 | validation |
| 144212608 | C12(C3(C(C(C(C1(C(C(C(C2(F)F)(F)F)(F)F)(F)F)F)(F)F)(F)F)(C(C(C(C3(F)F)(F)F)(F)F)(F)F)F)F)F | 0 | test |
| 144212607 | CC/C=C¥CCOC(=O)C | 0 | train |
| 144212606 | CC1CCC2(O1)C(=CCCC2(C)C)C | 0 | train |
| 144212605 | CCCCC(=O)O[C@@]1([C@H](C[C@@H]2[C@@]1(C[C@@H]([C@]3([C@H]2CCC4=CC(=O)C=C[C@@]43C)F)O)C)C)C(=O)CO | 0 | validation |
| 144212604 | CC1=CC=CC=C1CCO | 0 | test |
| 144212603 | C[C@]12CCC(=O)C(=C1CC[C@@H]3[C@@H]2CC[C@]4([C@H]3CCC4=O)C)O | 0 | train |
| 144212602 | C[C@]12CC[C@H](C1(C)C)C[C@H]2O | 0 | train |
| 144212601 | CC1=C(C(CCC1)(C)C)CC=O | 0 | validation |
| 144212600 | C([C@@H]1[C@@H]([C@@H]([C@H]([C@@H](O1)O[C@H]([C@@H](CO)O)[C@@H]([C@H](CO)O)O)O)O)O)O.O | 0 | test |
| 144212599 | CC1=C2C(=CC=C1)N=CC=N2 | 0 | train |
| 144212598 | CC(C)(C)C1CCCCC1=O | 0 | train |
| 144212597 | P#[In] | 0 | validation |
| 144212596 | C1=CC=C2C(=C1)C=CC=C2NC(=O)C3=CC=CC=C3C(=O)O | 1 | test |
| 144212595 | CCCCCCCCCCCCCCCCC | 0 | train |
| 144212594 | C1CCC2=NC3=CC=CC=C3C(=C2C1)N | 0 | train |
| 144212593 | CC(C)CCCCCCCCCCCCCCC(=O)O | 0 | validation |
| 144212592 | C1=CC(=CC=C1C2=CC=C(C=C2)F)N | 1 | test |
| 144212591 | C1=CC(=CC=C1[N+](=O)[O-])[As](=O)(O)O | 0 | train |
| 144212590 | CC1(CCC(C1(CN2C=NC=N2)O)CC3=CC=C(C=C3)Cl)C | 0 | train |

| | | | |
|---|---|---|---|
| 144212589 | CC(/C(=N/OC(=O)NC)/C)S(=O)(=O)C | 0 | validation |
| 144212588 | CC(C)C1=CC2=C(C=C1)C(=CC(=C2S(=O)(=O)[O-])C(C)C)C(C)C.[Na+] | 0 | test |
| 144212587 | CCC(=O)O[C@H]1CC[C@@H]2[C@@]1(CC[C@H]3[C@H]2CCC4=CC(=O)CC[C@]34C)C | 0 | train |
| 144212586 | C[C@@H]1[C@H]([C@H](C[C@@H](O1)O[C@@H]2[C@H](O[C@H](C[C@@H]2O)O[C@@H]3[C@H](O[C@H](C[C@@H]3O)O[C@H]4CC[C@]5([C@@H](C4)CC[C@@H]6[C@@H]5CC[C@]7([C@@]6(CC[C@@H]7C8=CC(=O)OC8)O)C)C)C)C)O)O | 0 | train |
| 144212585 | C1CN(CCN1C(C(Cl)(Cl)Cl)NC=O)C(C(Cl)(Cl)Cl)NC=O | 0 | validation |
| 144212584 | CC(C)(C)NC1=NC(=NC(=N1)NC2CC2)SC | 1 | test |
| 144212583 | C(CN)N.I.I | 0 | train |
| 144212582 | CC1=C(C=C(C=C1)N)S(=O)(=O)O | 0 | train |
| 144212581 | CC1=NN(C(=O)C1N=NC2=CC=C(C=C2)S(=O)(=O)[O-])C3=CC(=C(C=C3Cl)S(=O)(=O)[O-])Cl.[Na+].[Na+] | 0 | validation |
| 144212580 | C1=CC=C(C=C1)C(=O)[O-].C1=CC=C(C=C1)C(=O)[O-].[Mg+2] | 0 | test |
| 144212579 | CC(C)(C)C1=CC(=C(C=C1)OP(=O)(NC)OC)Cl | 1 | train |
| 144212578 | C1CN(CCN1CCOCC(=O)O)C(C2=CC=CC=C2)C3=CC=C(C=C3)Cl.Cl.Cl | 0 | train |
| 144212577 | CCCCC(CC)COC(=O)COC1=C(C=C(C=C1)Cl)C | 0 | validation |
| 144212576 | C1C(C(C(=O)N1C2=CC=CC(=C2)C(F)(F)F)Cl)CCl | 0 | test |
| 144212575 | C1=CC(=C(C=C1NC(=O)N)Cl)Cl | 0 | train |
| 144212574 | C1CCC2(CC1)N(CCO2)C(=O)C(Cl)Cl | 1 | train |
| 144212573 | CN(C1=CC=C(C=C1)[N+](=O)[O-])N=O | 0 | validation |
| 144212572 | CC1=C(C(=C(C=C1)Cl)N)Cl | 0 | test |
| 144212571 | CC(C)[C@@H](C1=CC=C(C=C1)OC(F)F)C(=O)OC(C#N)C2=CC(=CC=C2)OC3=CC=CC=C3 | 1 | train |
| 144212570 | CC1(CCC=C2[C@@]13CC[C@@H](C3)C2(C)C)C | 0 | train |
| 144212569 | C1=CC(=CN=C1)CNC(=O)NC2=CC=C(C=C2)[N+](=O)[O-] | 1 | validation |
| 144212568 | CC1=CC=CC=C1OCC2=CC=CC=C2/C(=N¥OC)/C(=O)OC | 1 | test |
| 144212567 | C1CN1CCC(=O)OCC(CO)(COC(=O)CCN2CC2)COC(=O)CCN3CC3 | 0 | train |
| 144212566 | CCOC(=O)C1=CC=CC=C1C | 0 | train |
| 144212565 | CC1=C(C(=C(C(=C1[N+](=O)[O-])C(C)(C)C)[N+](=O)[O-])C)[N+](=O)[O-] | 1 | validation |
| 144212564 | CCC(CC)(C(=O)NC(=O)N)Br | 0 | test |
| 144212563 | C(=O)O[Al](OC=O)OC=O | 0 | train |
| 144212562 | C(COC(=O)CS)OC(=O)CS | 0 | train |
| 144212561 | CCC(C)(CCC(C)C)C(=O)O | 0 | validation |
| 144212560 | CC(=O)O[Al].O.O | 0 | test |
| 144212559 | CCCCCCCCP(=O)(O)O | 0 | train |
| 144212558 | C1=CC=C(C=C1)[Sn](C2=CC=CC=C2)(C3=CC=CC=C3)F | 1 | train |
| 144212557 | C1=CC=C(C=C1)[Bi](C2=CC=CC=C2)C3=CC=CC=C3 | 1 | validation |
| 144212556 | C1=CC(=CC=C1N[C@@H](CCC(=O)N)C(=O)O)[N+](=O)[O-].Cl | 0 | test |
| 144212555 | C=CCOCC(CCl)O | 0 | train |
| 144212554 | CC1=C(C=C2C(=C1[N+](=O)[O-])C(CC2(C)C)(C)C)[N+](=O)[O-] | 1 | train |
| 144212553 | C[C@@H]1[C@H](C[C@@H]([C@H](O1)OC2[C@@H]([C@H](C([C@@H]([C@@H]2O)O)O)O)O)N)N=C(C(=O)O)N.Cl | 0 | validation |
| 144212552 | COC(=O)C1=CC(=CC(=C1)Cl)Cl | 0 | test |
| 144212551 | [C-]#N.[C-]#N.[K+].[Au+] | 1 | train |
| 144212550 | CCCCCCCCCC[N+](C)(C)[O-] | 0 | train |
| 144212549 | CC(C)CCC(=O)CCC(C)C | 0 | validation |
| 144212548 | C1=CC=C2C(=C1)C(=O)C3=CC(=C4C(=C3C2=O)NC5=C(C=C6C(=C5N4)C(=O)C7=CC=CC=C7C6=O)Cl)Cl | 0 | test |
| 144212547 | CC1=CC(=C(C(=C1)C)C)C | 0 | train |
| 144212546 | CCCCCCCCCCCCOS(=O)(=O)O.CC[C@@H]1[C@@]([C@@H]([C@H](C(=O)[C@@H](C[C@@]([C@@H]([C@H]([C@@H]([C@H](C(=O)O1)C)O[C@H]2C[C@@]([C@H]([C@@H](O2)C)O)(C)OC)C)O[C@H]3[C@@H]([C@H](C[C@H](O3)C)N(C)C)OC(=O)CC)(C)O)C)C)O)(C)O | 0 | train |
| 144212545 | CC(=O)C1=CC=C(C=C1)OC(=O)C | 0 | validation |
| 144212544 | CCOC(=S)SSC(=S)OCC | 0 | test |
| 144212543 | CN1[C@@H]2CC(C[C@H]1[C@H]3[C@@H]2O3)OC(=O)[C@H](CO)C4=CC=CC=C4.Cl | 0 | train |
| 144212542 | CCN(CCC#N)C1=CC=C(C=C1)N=NC2=C(C=C(C=C2Cl)[N+](=O)[O-])Cl | 1 | train |
| 144212541 | CCC(C)C1=CC(=CC(=C1OC(=O)OC(C)C)[N+](=O)[O-])[N+](=O)[O-] | 0 | validation |
| 144212540 | CCNC1=NC(=NC(=N1)SC)NC(C)C(C)C | 1 | test |
| 144212539 | CC1([C@@H]([C@@H]1C(=O)OCC2=COC(=C2)CC3=CC=CC=C3)/C=C/4¥CCSC4=O)C | 1 | train |
| 144212538 | CCN(CC)C1=C(C=C(C(=C1[N+](=O)[O-])N)C(F)(F)F)[N+](=O)[O-] | 1 | train |
| 144212537 | C1C(=NN(C1=O)C2=CC=C(C=C2)S(=O)(=O)O)C(=O)O | 0 | validation |
| 144212536 | CCOP(=S)(NC(C)C)OC1=CC=CC=C1C(=O)OC(C)C | 1 | test |
| 144212535 | CC1=CC(=C(C=C1)C(=O)OC)C2=NC(C(=O)N2)(C)C(C)C | 0 | train |

| | | | |
|---|---|---|---|
| 144212534 | C/C=C/C(=O)OC1=C(C=C(C=C1CCCCCC(C)C)[N+](=O)[O-])[N+](=O)[O-] | 0 | train |
| 144212533 | CCOC(=O)COC1=C(C=C(C=C1)Cl)Cl | 0 | validation |
| 144212532 | CC1=CC(=C(C=C1)C)S(=O)(=O)[O-].[Na+] | 0 | test |
| 144212531 | CC1C(O1)C2CCC3(C(C2)O3)C | 0 | train |
| 144212530 | CC(=O)CC(=O)NC1=C(C=CC(=C1)OC)OC | 0 | train |
| 144212529 | CCCCCOC(=O)CCCC | 0 | validation |
| 144212528 | CC(=C)C(=O)NCCC[N+](C)(C)C.[Cl-] | 0 | test |
| 144212527 | CC1=CC(=C(C(=C1)C(C)(C)C)OC(=O)NC)C(C)(C)C | 1 | train |
| 144212526 | CCOP(=S)(OCC)OC1=NOC(=C1)C2=CC=CC=C2 | 1 | train |
| 144212525 | CCC(C1=CC=CC=C1)C(=O)NC(=O)N | 0 | validation |
| 144212524 | C1=CC(=C(C(=C1)Cl)C(=S)N)Cl | 0 | test |
| 144212523 | C1CCCCCC(=O)OCCOC(=O)CCCC1 | 0 | train |
| 144212522 | CCN1CCCC1CNC(=O)C2=C(C=CC(=C2)S(=O)(=O)N)OC | 0 | train |
| 144212521 | CC/C=C¥CCCCCCCCCCOC(=O)C | 0 | validation |
| 144212520 | C1=CC2=C(C=CC(=C2[O-])[N+]#N)C(=C1)S(=O)(=O)[O-].[Na+] | 0 | test |
| 144212519 | CCOC1=NC2=CC=CC(=C2N1CC3=CC=C(C=C3)C4=CC=CC=C4C5=NNN=N5)C(=O)OC(C)OC(=O)OC6CCCCC6 | 0 | train |
| 144212518 | C[C@@H]1OC[C@@H]2[C@@H](O1)[C@@H]([C@H]([C@@H](O2)OC3C4COC(=O)[C@@H]4[C@@H](C5=CC6=C(C=C35)OCO6)C7=CC(=C(C(=C7)OC)O)OC)O)O | 0 | train |
| 144212517 | C1C(C(CS1(=O)=O)(Cl)Cl)(Cl)Cl | 1 | validation |
| 144212516 | COC1=C(C=C(C=C1)[N+](=O)[O-])[N+](=O)[O-] | 0 | test |
| 144212515 | CCCCCCCC[N+](C)(CCCCCCCC)CCCCCCCC.[Cl-] | 1 | train |
| 144212514 | CN1C(S(=O)(=O)CCC1=O)C2=CC=C(C=C2)Cl | 0 | train |
| 144212513 | CC(C)C(C)(C#N)N | 0 | validation |
| 144212512 | CC1=C(C(CCC1)(C)C)/C=C/C(=C¥C=C¥C(=C¥C(=O)O)¥C)/C | 1 | test |
| 144212511 | C1CCC(CC1)(C#N)N | 0 | train |
| 144212510 | CCO[Si](CCCS)(OCC)OCC | 0 | train |
| 144212509 | [O-]Cl(=O)(=O)=O.[K+] | 0 | validation |
| 144212508 | CN(C)C1=CC=C(C=C1)SC#N | 0 | test |
| 144212507 | C1=CC=C(C=C1)OC2=CC=C(C=C2)O | 1 | train |
| 144212506 | CC1=C(C=C(C=C1)NC(=O)C)Cl | 0 | train |
| 144212505 | COC(=O)CCC(=O)O | 0 | validation |
| 144212504 | CC1=C(SC=N1)CCO | 0 | test |
| 144212503 | CC(=O)C1=CC2=CC=CC=C2C=C1 | 1 | train |
| 144212502 | CC(=O)NC1=CC=CC=C1O | 1 | train |
| 144212501 | CN(C)C1=CC=NC=C1 | 0 | validation |
| 144212500 | C(F)(F)(F)S(=O)(=O)O | 0 | test |
| 144212499 | C1=CC=C(C=C1)SC2=CC=CC=C2 | 0 | train |
| 144212498 | C(CSSCCO)O | 0 | train |
| 144212497 | C1=C(C=C(C=C1C(=O)Cl)C(=O)Cl)C(=O)Cl | 0 | validation |
| 144212496 | CCO[Si](CCCN=C=O)(OCC)OCC | 0 | test |
| 144212495 | C=CCSSCC=C | 0 | train |
| 144212494 | CO[Si](CCCS)(OC)OC | 0 | train |
| 144212493 | CC[Hg]SC1=CC=CC=C1C(=O)[O-].[Na+] | 1 | validation |
| 144212492 | Cl[Ni]Cl | 0 | test |
| 144212491 | COC1=C(C=C2C(=C1)[C@]34CCN5[C@H]3C[C@@H]6[C@@H]7[C@H]4N2C(=O)C[C@@H]7OCC=C6C5)OC | 0 | train |
| 144212490 | CN1[C@@H]2CC[C@H]1CC(C2)OC(=O)C(CO)C3=CC=CC=C3.CN1[C@@H]2CC[C@H]1CC(C2)OC(=O)C(CO)C3=CC=CC=C3.O.OS(=O)(=O)O | 0 | train |
| 144212489 | CNC(=S)NN | 1 | validation |
| 144212488 | CC(CC1=CC(=C(C=C1)O)O)C(C)CC2=CC(=C(C=C2)O)O | 1 | test |
| 144212487 | C1=CC(=C(C=C1C2=CC(=C(C=C2)N)N)N)N | 1 | train |
| 144212486 | C/C(=C(/C)¥N=O)/NO | 0 | train |
| 144212485 | CCO[Si](C1=CC=CC=C1)(OCC)OCC | 0 | validation |
| 144212484 | CCO[Si](C=C)(OCC)OCC | 0 | test |
| 144212483 | C1CCOC(=O)C1 | 0 | train |
| 144212482 | C1=CC=C(C=C1)C(=N)N.Cl | 0 | train |
| 144212481 | CC1C(=O)OC(C(=O)O1)C | 0 | validation |
| 144212480 | C[N+](=CCl)C.[Cl-] | 0 | test |
| 144212479 | CCCCCCCCO[C@H]1[C@@H]([C@H]([C@@H]([C@H](O1)CO)O)O)O | 0 | train |
| 144212478 | C1[C@H]2[C@@H]([C@@H](S1)CCCCC(=O)O)NC(=O)N2 | 0 | train |
| 144212477 | CC1=CC=CC=C1CCl | 0 | validation |

| | | | |
|---|---|---|---|
| 144212476 | C1=CC=C(C=C1)CC(=O)C2=CC=CC=C2 | 1 | test |
| 144212475 | C1CC(=O)CC(=O)C1 | 0 | train |
| 144212474 | C(C(CO)(CO)N)O.Cl | 0 | train |
| 144212473 | C1=C(C=C(C(=C1I)OC2=CC(=C(C(=C2)I)O)I)I)C[C@@H](C(=O)O)N | 0 | validation |
| 144212472 | C1CN(CCN1CCO)CCS(=O)(=O)[O-].[Na+] | 0 | test |
| 144212471 | CC(=O)S[C@@H]1CC2=CC(=O)CC[C@@]2([C@@H]3[C@@H]1[C@@H]4CC[C@]5([C@]4(CC3)C)CCC(=O)O5)C | 0 | train |
| 144212470 | C[C@@H]1CC[C@H](C(=O)C1)C(C)C | 0 | train |
| 144212469 | C1=CC=C(C(=C1)C2=C3C=C(C(=O)C(=C3OC4=C(C(=C(C=C24)Br)[O-])Br)Br)Br)C(=O)[O-].[Na+].[Na+] | 0 | validation |
| 144212468 | CCN(CC)C1=CC=C(C=C1)C(=C2C=CC(=[N+](CC)CC)C=C2)C3=C(C=C(C=C3)S(=O)(=O)[O-])S(=O)(=O)[O-].[Na+] | 1 | test |
| 144212467 | COC(C1=CC=CC=C1)(C(=O)C2=CC=CC=C2)OC | 1 | train |
| 144212466 | CCCC[N+](CCCC)(CCCC)CCCC.[Br-] | 0 | train |
| 144212465 | C1=C(C=C(C(=C1S(=O)(=O)[O-])O)O)S(=O)(=O)[O-].[Na+].[Na+] | 0 | validation |
| 144212464 | COCCOCCOCCOCCOC | 0 | test |
| 144212463 | C1COCCN1CCCS(=O)(=O)O | 0 | train |
| 144212462 | CC(C)C1=C(C(=CC=C1)C(C)C)N | 0 | train |
| 144212461 | CCCCC(CC)COC(=O)CC#N | 0 | validation |
| 144212460 | COC1=CC2=CC=CC=C2C=C1 | 0 | test |
| 144212459 | C[N+](C)(C)CC(=O)O.[Cl-] | 0 | train |
| 144212458 | CC(=O)[C@H]1CC[C@@H]2[C@@]1(CC[C@H]3[C@H]2CC=C4[C@@]3(CC[C@@H](C4)O)C)C | 0 | train |
| 144212457 | CCN1CCCC1=O | 0 | validation |
| 144212456 | C1=C(NC(=O)N=C1)N | 0 | test |
| 144212455 | CC[N+](CC)(CC)CC.[Br-] | 0 | train |
| 144212454 | C1=CC=C(C=C1)/C=C/C(=O)O | 0 | train |
| 144212453 | COC1=CC=CC(=C1)C=O | 0 | validation |
| 144212452 | CC1=CC=C(C=C1)C(C)(C)O | 0 | test |
| 144212451 | C1C=CC=C1 | 0 | train |
| 144212450 | CCCCCCCCCC1=CC=C(C=C1)OCCOCCO | 0 | train |
| 144212449 | CC1(CC(CC(N1)(C)C)N)C | 0 | validation |
| 144212448 | C1=CC=C2C(=C1)C(OS2(=O)=O)(C3=CC=C(C=C3)O)C4=CC=C(C=C4)[O-].[Na+] | 0 | test |
| 144212447 | C1=CC(=CC=C1C(=O)C2=CC=C(C=C2)Cl)Cl | 1 | train |
| 144212446 | CC[N+](CC)(CC)CC.[Cl-] | 0 | train |
| 144212445 | COC1=C(C=CC(=C1)[N+](=O)[O-])N | 0 | validation |
| 144212444 | CCCCCCNCCCCCC | 0 | test |
| 144212443 | CCCCOC(=O)CC#N | 0 | train |
| 144212442 | C=CCOCC(CO)O | 0 | train |
| 144212441 | C1=CC(=C(C=C1C(=O)O)N)Cl | 0 | validation |
| 144212440 | CC1=C(C=C(C=C1)Cl)Cl | 0 | test |
| 144212439 | C1=CC(=CC=C1C=O)F | 0 | train |
| 144212438 | C(CCCCl)CCCl | 0 | train |
| 144212437 | C1=CC(=CC=C1C#N)C(=O)O | 0 | validation |
| 144212436 | CC(=O)C1=CN=CC=C1 | 0 | test |
| 144212435 | CC1=C(C=C(C=C1)C(=O)N)N | 0 | train |
| 144212434 | CC(=O)CC(C)(C)NC(=O)C=C | 0 | train |
| 144212433 | Cl[Au](Cl)Cl | 1 | validation |
| 144212432 | CC(C)(CCO)OC | 0 | test |
| 144212431 | CCN(CC)CCOC(=O)C1=CC=C(C=C1)N.Cl | 0 | train |
| 144212430 | CC(=CCCC(=CC#N)C)C | 0 | train |
| 144212429 | CCCCCCCCBr | 0 | validation |
| 144212428 | C1=CC=C(C(=C1)C(=O)O)NC2=CC=CC(=C2)C(F)(F)F | 1 | test |
| 144212427 | C1=CC=C(C(=C1)C#N)O | 0 | train |
| 144212426 | CCOC(=O)C(=O)OCC | 0 | train |
| 144212425 | C1=NC2=C(N1)C(=NC=N2)N | 0 | validation |
| 144212424 | C1CCN(CC1)C=O | 0 | test |
| 144212423 | C#CC1(CCCCC1)O | 0 | train |
| 144212422 | CC1=C(C(=C(C=C1C2(C3=CC=CC=C3S(=O)(=O)O2)C4=CC(=C(C(=C4C)Br)O)Br)Br)O)Br | 1 | train |
| 144212421 | C1CCC(CC1)C2CCCCC2 | 0 | validation |
| 144212420 | CN(C)C1=CC=C(C=C1)C(C2=CC=CC=C2)C3=CC=C(C=C3)N(C)C | 0 | test |
| 144212419 | C1=CC=C(C=C1)N/C=C/C=C/C=NC2=CC=CC=C2.Cl | 0 | train |

| | | | |
|---|---|---|---|
| 144212418 | CCCCCCCCCCCCCCOS(=O)(=O)[O-].[Na+] | 0 | train |
| 144212417 | CCCC[N+](C)(CCCC)CCCC.[Cl-] | 0 | validation |
| 144212416 | CCCOC(OCCC)OCCC | 0 | test |
| 144212415 | C(=NN)(N)N.Cl | 0 | train |
| 144212414 | CC1=NC(=NC(=N1)OC)N | 0 | train |
| 144212413 | C1=CC(=C(C=C1O)S(=O)(=O)[O-])O.[K+] | 0 | validation |
| 144212412 | CCOC1=CC=CC=C1OCC | 0 | test |
| 144212411 | COC1=CC=CC=C1OCC(CO)O | 0 | train |
| 144212410 | CC(=C)C(=O)OCCCCCCOC(=O)C(=C)C | 0 | train |
| 144212409 | C1=CC=C(C=C1)COC(=O)CBr | 0 | validation |
| 144212408 | C1=CC=C2C(=C1)C(=O)C(C2=O)C3=NC4=C(C=C(C=C4C=C3)S(=O)(=O)[O-])S(=O)(=O)[O-].[Na+].[Na+] | 1 | test |
| 144212407 | COC1=CC(=C(C=C1)OC)Cl | 0 | train |
| 144212406 | C(CN(CCO)CCO)N(CCO)CCO | 0 | train |
| 144212405 | C1=CC=C2C(=C1)C=CC(=O)C2=NNC3=CC=C(C=C3)S(=O)(=O)[O-].[Na+] | 0 | validation |
| 144212404 | CN(C)C1=CC2=C(C=C1)N=C3C=CC(=[N+](C)C)C=C3S2.[Cl-] | 1 | test |
| 144212403 | CCN(CC1=CC(=CC=C1)S(=O)(=O)[O-])C2=CC=C(C=C2)C(=C3C=CC(=[N+](CC)CC4=CC(=CC=C4)S(=O)(=O)[O-])C=C3)C5=C(C=C(C=C5)O)S(=O)(=O)[O-].[Na+].[Na+] | 1 | train |
| 144212402 | C1=CC=C(C(=C1)C2=C3C=C(C(=O)C(=C3OC4=C(C(=C(C=C24)I)[O-])I)I)I)C(=O)[O-].[Na+].[Na+] | 0 | train |
| 144212401 | CCCCCCCCCCCC[N+](C)(C)C.[Br-] | 0 | validation |
| 144212400 | CC1=CC(=C(C=C1)NC(=O)CC(=O)C)C | 0 | test |
| 144212399 | CCCCCCCCCCOS(=O)(=O)[O-].[Na+] | 0 | train |
| 144212398 | CC(C)(C)NC(=O)N | 0 | train |
| 144212397 | C1=CC=C2C(=C1)C(OS2(=O)=O)(C3=CC(=C(C(=C3)Br)O)Br)C4=CC(=C(C(=C4)Br)O)Br | 1 | validation |
| 144212396 | CCCCCCCCCCCCCCCC[N+](C)(C)CC1=CC=CC=C1.[Cl-] | 1 | test |
| 144212395 | C1=CC(=C(C=C1N)N)S(=O)(=O)O | 0 | train |
| 144212394 | CC1(CC(CC(N1C)(C)C)O)C | 0 | train |
| 144212393 | C1COCCN1CCCN | 0 | validation |
| 144212392 | CCC(C)C1=C(C(=CC=C1)C(C)CC)O | 0 | test |
| 144212391 | C1=CC(=CN=C1)CN | 0 | train |
| 144212390 | C1=CC(=C(C=C1NC(=O)C2=CC(=CC(=C2O)Cl)Cl)Cl)Cl | 1 | train |
| 144212389 | C1=CC(=CC=C1S(=O)(=O)O)Cl | 0 | validation |
| 144212388 | C1COCCN1CCN | 0 | test |
| 144212387 | C(O)[P+](CO)(CO)CO.[Cl-] | 0 | train |
| 144212386 | C=CCCC(=O)O | 0 | train |
| 144212385 | CCCCNC1=CC=CC=C1 | 0 | validation |
| 144212384 | CCCCCCCCCCCCCC[N+](C)(C)C.[Br-] | 0 | test |
| 144212383 | C1=CC(=CC(=C1)Cl)NN=C(C#N)C#N | 1 | train |
| 144212382 | CCCCCCCCCCCC[N+](C)(C)CC1=CC=CC=C1.[Cl-] | 1 | train |
| 144212381 | C1=CC(=CC=C1CC2=CC=C(C=C2)N3C(=O)C=CC3=O)N4C(=O)C=CC4=O | 1 | validation |
| 144212380 | CCOC(=O)CC(=O)CCl | 0 | test |
| 144212379 | CC(C)C1=CC=CC=C1O | 0 | train |
| 144212378 | C1=CC(=CC=C1CBr)Br | 0 | train |
| 144212377 | C1=CC=C(C=C1)P(=O)(O)O | 0 | validation |
| 144212376 | C(CCCBr)CCBr | 0 | test |
| 144212375 | CC(C)(C)NCCO | 0 | train |
| 144212374 | CC(=O)CC(=O)OC(C)(C)C | 0 | train |
| 144212373 | C=CCC1=CC=CC=C1O | 0 | validation |
| 144212372 | C1[C@H]([C@@H]([C@H](C(O1)O)O)O)O | 0 | test |
| 144212371 | C1=CC=C(C=C1)P(=O)(C2=CC=CC=C2)C3=CC=CC=C3 | 1 | train |
| 144212370 | CCOC(=O)CP(=O)(OCC)OCC | 0 | train |
| 144212369 | C(C(C(=O)O)O)(C(=O)O)O | 0 | validation |
| 144212368 | C1=CC(=C(N=C1)C(=O)O)C(=O)O | 0 | test |
| 144212367 | CCOCCOCCOCC | 0 | train |
| 144212366 | CC1=C(C=CC=C1Cl)Cl | 0 | train |
| 144212365 | C1=C(C=C(C=C1N)N)C(=O)O | 0 | validation |
| 144212364 | CC1=CC(=CC(=C1Cl)C)O | 0 | test |
| 144212363 | CCCCCCCCCCCCBr | 0 | train |
| 144212362 | C(COS(=O)(=O)O)N | 0 | train |
| 144212361 | CC1=CC2=C(C=C1)C(=O)C3=CC=CC=C3C2=O | 1 | validation |

| | | | |
|---|---|---|---|
| 144212360 | C1C=CC[C@H]2[C@@H]1C(=O)NC2=O | 0 | test |
| 144212359 | CC1CN(NC1=O)C2=CC=CC=C2 | 1 | train |
| 144212358 | C1=CC=C2C(=C1)C(=O)C3=C(C=CC(=C3C2=O)O)O | 1 | train |
| 144212357 | C1=CC=C(C=C1)C(CO)O | 0 | validation |
| 144212356 | CCCCCC(C=C)O | 0 | test |
| 144212355 | COC(=O)C1=CN=CC=C1 | 0 | train |
| 144212354 | CNC[C@@H]([C@H]([C@@H]([C@@H](CO)O)O)O)O | 0 | train |
| 144212353 | CC1=CC(=O)CCC1 | 0 | validation |
| 144212352 | C1(C(C(C(C(C1O)O)O)O)O)O | 0 | test |
| 144212351 | CC(=CCC/C(=C/CC/C(=C/CO)/C)/C)C | 1 | train |
| 144212350 | CCOP(O)OCC | 0 | train |
| 144212349 | CC(C)(C)C1CCC(=O)CC1 | 0 | validation |
| 144212348 | C1=CC=C(C=C1)C(=O)C(=O)C2=CC=CC=C2 | 1 | test |
| 144212347 | C1=C(C(=CC(=C1C(=O)O)C(=O)O)C(=O)O)C(=O)O | 0 | train |
| 144212346 | CC(=O)C1CCOC1=O | 0 | train |
| 144212345 | CC(CO)(CO)[N+](=O)[O-] | 0 | validation |
| 144212344 | C1COCCN1C=O | 0 | test |
| 144212343 | CC1=NC2=CC=CC=C2C=C1 | 0 | train |
| 144212342 | CCC(=O)C1=CC=CC=C1 | 0 | train |
| 144212341 | C1=CC=C(C=C1)C2=CC(=O)C3=C(O2)C=CC4=CC=CC=C43 | 1 | validation |
| 144212340 | CC1=CC=C(C=C1)C(=O)C | 0 | test |
| 144212339 | C1COCCN1CCO | 0 | train |
| 144212338 | CCOC(=O)C1=CC=CC=C1 | 0 | train |
| 144212337 | COC(=O)CCCCC(=O)O | 0 | validation |
| 144212336 | CN1[C@@H]2CC(C[C@H]1[C@H]3[C@@H]2O3)OC(=O)[C@H](CO)C4=CC=CC=C4.Br | 0 | test |
| 144212335 | C[C@H]1C[C@H]2[C@@H]3CC[C@]([C@]3(C[C@@H]([C@@H]2[C@@]4(C1=CC(=O)C=C4)C)O)C)(C(=O)CO)O | 0 | train |
| 144212334 | C1CN2CC3=CCO[C@H]4CC(=O)N5[C@H]6[C@H]4[C@H]3C[C@H]2[C@@]61C7=CC=CC=C75.C1CN2CC3=CCO[C@H]4CC(=O)N5[C@H]6[C@H]4[C@H]3C[C@H]2[C@@]61C7=CC=CC=C75.OS(=O)(=O)O | 0 | train |
| 144212333 | CC(=O)[O-].CC(=O)[O-].[Zn+2] | 0 | validation |
| 144212332 | CCCCNP(=S)(N)N | 0 | test |
| 144212331 | COC(=O)C1=CC=CC=C1Cl | 0 | train |
| 144212330 | CCCCCOC(=O)CCC | 0 | train |
| 144212329 | CCCCCCC1=CC=C(C=C1)N | 0 | validation |
| 144212328 | C(COCCCl)OCCCl | 0 | test |
| 144212327 | C/C=C/C/C=C¥CCCCCCCCOC(=O)C | 0 | train |
| 144212326 | COP(=O)(OC)OC1=CC=C(C=C1)[N+](=O)[O-] | 0 | train |
| 144212325 | CC(C)CCCC(C)C/C=C/C(=C/C(=O)OCC#C)/C | 0 | validation |
| 144212324 | CC1([C@@H]([C@@H]1C(=O)O[C@@H](C#N)C2=CC(=CC=C2)OC3=CC=CC=C3)C=C(Cl)Cl)C | 1 | test |
| 144212323 | CC(C)C1=CC(=CC=C1)OC(=O)NC | 1 | train |
| 144212322 | CCOC(=O)N(C)C(=O)CSP(=S)(OCC)OCC | 1 | train |
| 144212321 | CC(C(=O)O[Hg]C1=CC=CC=C1)O | 1 | validation |
| 144212320 | C1=CC2=C(C(=C1)OCC(COC3=CC=CC4=C3C(=O)C=C(O4)C(=O)[O-])O)C(=O)C=C(O2)C(=O)[O-].[Na+].[Na+] | 0 | test |
| 144212319 | C([C@@H]1[C@@H]([C@@H]([C@H](C(O1)O)N)O)O)O.Cl | 0 | train |
| 144212318 | C1=CC=C(C=C1)CN(CC2=CC=CC=C2)O | 1 | train |
| 144212317 | CC(=O)[O-].CC(=O)[O-].[Hg+2] | 1 | validation |
| 144212316 | CCOC(=O)C1=NN(C(=O)C1)C2=CC=CC=C2 | 0 | test |
| 144212315 | COC1=C(C(=CC=C1)OC)C(=O)O | 0 | train |
| 144212314 | C1C(=O)N=C(N1N=CC2=CC=C(O2)C3=CC=C(C=C3)[N+](=O)[O-])[O-].[Na+] | 0 | train |
| 144212313 | CC1=CC(=C(C(=C1)Cl)N)C | 0 | validation |
| 144212312 | CC(C)(C)C1=NNC(=S)N(C1=O)N | 0 | test |
| 144212311 | CNC(=O)C(C1=CC=CC=C1)C2=CC=CC=C2 | 0 | train |
| 144212310 | C1=CC=C(C=C1)NC(=O)C2=C(C=CC(=C2)[N+](=O)[O-])Cl | 0 | train |
| 144212309 | COC1=CC=CC=C1NC(=O)C2=CC3=CC=CC=C3C=C2O | 1 | validation |
| 144212308 | C[C@@H]1CC[C@@H](C(=O)C1)C(C)C | 0 | test |
| 144212307 | C1CC(=O)N(C1)CC(=O)N | 0 | train |
| 144212306 | C1CCC(=O)N(CC1)SSN2CCCCCC2=O | 0 | train |
| 144212305 | CC(C)(C)C(=O)CCC1=CC=C(C=C1)Cl | 0 | validation |
| 144212304 | CCC1(C(=O)NC(=O)N1)C | 0 | test |

| | | | |
|---|---|---|---|
| 144212303 | C1=CC=C(C=C1)NC2=CC=C(C=C2)O | 0 | train |
| 144212302 | COC(C1=CC=CC=C1)C(=O)O | 0 | train |
| 144212301 | CC(=O)C(C(=O)N(C)C)Cl | 0 | validation |
| 144212300 | CC1=C(C(=CC=C1)NC2=CC=CC=C2C(=O)O)C | 0 | test |
| 144212299 | CC(C)CCCCCCN | 0 | train |
| 144212298 | CC1=C(C=CC(=C1)Br)NC(=O)CCl | 1 | train |
| 144212297 | COC(=O)C1=C(C=CS1)S(=O)(=O)N | 0 | validation |
| 144212296 | CC(C)NC(=O)C1=CC=CC=C1N | 0 | test |
| 144212295 | CCOC(=O)C=CC(=O)O | 1 | train |
| 144212294 | COC(=O)C(CBr)Br | 0 | train |
| 144212293 | C1=CC(=CC=C1OC2=CC=C(C=C2)S(=O)(=O)NN)S(=O)(=O)NN | 0 | validation |
| 144212292 | CC(C)NC(=O)NC(C)C | 0 | test |
| 144212291 | CC(=CCC/C(=C¥COC(=O)C)/C)C | 0 | train |
| 144212290 | CC1=C(C=CC=C1O)O | 0 | train |
| 144212289 | CC/C=C/CCO | 0 | validation |
| 144212288 | C1=CN(C(=O)N=C1N)[C@H]2[C@H]([C@@H]([C@H](O2)CO)O)O | 0 | test |
| 144212287 | CC(C)(C)C1=CC(=CC(=C1O)C(C)(C)C)CCC(=O)OCC(COC(=O)CCC2=CC(=C(C(=C2)C(C)(C)C)O)C(C)(C)C)(COC(=O)CCC3=CC(=C(C(=C3)C(C)(C)C)O)C(C)(C)C)COC(=O)CCC4=CC(=C(C(=C4)C(C)(C)C)O)C(C)(C)C | 0 | train |
| 144212286 | C[C@]12CC[C@H]3[C@H]([C@@H]1CC[C@@]2(C#C)O)CCC4=CCCC[C@H]34 | 0 | train |
| 144212285 | CC[C@@]12CCCN3[C@@H]1C4=C(CC3)C5=CC=CC=C5N4[C@](C2)(C(=O)OC)O | 1 | validation |
| 144212284 | C(#N)[S-].[NH4+] | 0 | test |
| 144212283 | CCCCCCCCCCCCCCOP(=O)(O)O | 0 | train |
| 144212282 | CCCCCCCCSCCO | 0 | train |
| 144212281 | CN(C(=O)NC1=CC=C(C=C1)Br)OC | 1 | validation |
| 144212280 | CCOC(=O)CN1C2=C(C=CC=C2Cl)SC1=O | 1 | test |
| 144212279 | CCCCCC[C@H](C/C=C¥CCCCCCCC(=O)OCC(CO)O)O | 0 | train |
| 144212278 | CCCCN1C(=O)C2=CC=CC=C2C1=O | 0 | train |
| 144212277 | C1=CC(=C(C=C1S(=O)(=O)O)N)S(=O)(=O)O | 0 | validation |
| 144212276 | CC1=NC2=C(C1(C)C)C3=CC=CC=C3C=C2 | 1 | test |
| 144212275 | C1CN(C(=O)N1)CCN | 0 | train |
| 144212274 | CC1(CC2=C(O1)C(=CC=C2)N)C | 0 | train |
| 144212273 | C1=C(C=C(C(=C1Br)O)Br)C(=O)O | 0 | validation |
| 144212272 | CC(CCCC(C)(C)OC)CC=O | 0 | test |
| 144212271 | CCCCCSCCCCC | 0 | train |
| 144212270 | C[C@@H](C1=CC2=C(C=C1)C=C(C=C2)OC)C(=O)O | 0 | train |
| 144212269 | C1CCN[C@@H](C1)C2=CN=CC=C2 | 0 | validation |
| 144212268 | C(CC(CBr)(C#N)Br)C#N | 1 | test |
| 144212267 | CC(CN/C=C/1¥C=CC=CC1=O)N/C=C/2¥C=CC=CC2=O | 1 | train |
| 144212266 | C(=S)(N)NC(=S)N | 0 | train |
| 144212265 | CC(C)CCCC(C)(C)O | 0 | validation |
| 144212264 | CCC(C)SSC(C)CC | 0 | test |
| 144212263 | C1N2CN3CN1C[N+](C2)(C3)C/C=C¥Cl.[Cl-] | 0 | train |
| 144212262 | CC1=NN(C(=C1)N)C2=CC=CC=C2 | 1 | train |
| 144212261 | C1=CC(=C(C=C1[C@H](CN)O)O)O | 0 | validation |
| 144212260 | C1=CC(=C(C=C1OC2=C(C=C(C=C2I)C[C@@H](C(=O)O)N)I)I)O | 0 | test |
| 144212259 | C1CCC(C(C1)C2=CC=CC=C2)O | 0 | train |
| 144212258 | C1=CC(=CC=C1C(=O)CBr)O | 0 | train |
| 144212257 | C[C@]12CC[C@H]3[C@H]([C@@H]1CC[C@]2(C#C)O)CCC4=CC5=C(C[C@]34C)C=NO5 | 0 | validation |
| 144212256 | CCCC(C)C1=CC=CC=C1O | 0 | test |
| 144212255 | CC1=C(C(=C(C(=C1C)[N+](=O)[O-])C(C)(C)C)[N+](=O)[O-])C | 1 | train |
| 144212254 | CCCCCCCCCCO[C@H]1[C@@H]([C@H]([C@@H]([C@H](O1)CO)O)O)O | 0 | train |
| 144212253 | C1=CC(=CC=C1NC2=NC(=NC(=N2)N(CCO)CCO)NC3=CC(=C(C=C3)/C=C/C4=C(C=C(C=C4)NC5=NC(=NC(=N5)NC6=CC=C(C=C6)S(=O)(=O)[O-])N(CCO)CCO)S(=O)(=O)[O-])S(=O)(=O)[O-])S(=O)(=O)[O-].[Na+].[Na+].[Na+].[Na+] | 0 | validation |
| 144212252 | CN1C(=O)C=CS1.Cl | 0 | test |
| 144212251 | CC1=NN(C(=O)C1N=NC2=CC=CC=C2)C3=C(C=CC(=C3)S(=O)(=O)[O-])Cl.[Na+] | 1 | train |
| 144212250 | CCC(C)(CCCC(C)C)OC(=O)C | 0 | train |
| 144212249 | C1=CC(=C(C=C1Cl)Cl)CC(=O)O | 0 | validation |
| 144212248 | C1=CC=C2C(=C1)C(C(=O)N2)(C3=CC=C(C=C3)O)C4=CC=C(C=C4)O | 0 | test |
| 144212247 | CCCCCCCC(=O)N(C)C | 0 | train |
| 144212246 | CC(=O)NC1=C(C(=C(C(=C1I)C(=O)O)I)NC(=O)C)I | 0 | train |

| | | | |
|---|---|---|---|
| 144212245 | CCCCOC(=O)CO | 0 | validation |
| 144212244 | CC1=CC=CC=C1C(C2=CC=CC=C2)OCCN(C)C.Cl | 0 | test |
| 144212243 | CCCCCCCCCCCCCCCC(=O)OC[C@@H]([C@@H]1C(=C(C(=O)O1)O)O)O | 0 | train |
| 144212242 | CC1=CC(=C(C=C1C)Cl)O | 0 | train |
| 144212241 | CCCCCCCCCCCCSC#N | 0 | validation |
| 144212240 | CCCCCCCC1CCCC1=O | 0 | test |
| 144212239 | C1=CC(=C(C(=C1)Cl)C(=O)N)Cl | 0 | train |
| 144212238 | CCOC(=O)C1=CC=CC=C1C(=C2C=C(C(=O)C(=C2)Br)Br)C3=CC(=C(C(=C3)Br)O)Br | 0 | train |
| 144212237 | C1=CC=C(C=C1)C(CC(C2=CC=C(C=C2)C3=CC=C(C=C3)Br)O)C4=C(C5=CC=CC=C5OC4=O)O | 0 | validation |
| 144212236 | CC(=CC1C(C1(C)C)C(=O)OC(C#N)C2=CC(=CC=C2)OC3=CC=CC=C3)C | 1 | test |
| 144212235 | COP(=S)(OC)OC1=CC(=C(C=C1Cl)Br)Cl | 1 | train |
| 144212234 | CCOP(=O)(C(=O)N)[O-].[NH4+] | 0 | train |
| 144212233 | CCCCOCCOC(=O)COC1=CC(=C(C=C1Cl)Cl)Cl | 0 | validation |
| 144212232 | CCOC1=C(C=C(C=C1)NC(=O)OC(C)C)OCC | 0 | test |
| 144212231 | CC(C)OC(=O)C(C1=CC=C(C=C1)Cl)(C2=CC=C(C=C2)Cl)O | 1 | train |
| 144212230 | CCCCOCCOC(=O)COC1=NC(=C(C=C1Cl)Cl)Cl | 1 | train |
| 144212229 | CCCCC(CC)COC(=O)COC1=CC(=C(C=C1Cl)Cl)Cl | 0 | validation |
| 144212228 | CC(C)(CO)[N+](=O)[O-] | 0 | test |
| 144212227 | C1=CC(=C(C(=C1Cl)CC(=O)O)Cl)Cl | 0 | train |
| 144212226 | CCOC(=O)NC(=S)NC1=CC=CC=C1NC(=S)NC(=O)OCC | 0 | train |
| 144212225 | COP(=S)(OC)OC1=C(C=C(C=C1)[N+](=O)[O-])Cl | 0 | validation |
| 144212224 | CN(C)S(=O)(=O)C1=CC=C(C=C1)OP(=S)(OC)OC | 1 | test |
| 144212223 | CCOC(=O)C1CC(=O)C(=C(C2CC2)O)C(=O)C1 | 0 | train |
| 144212222 | CCOP(=O)(N=C1SCCS1)OCC | 0 | train |
| 144212221 | CC(C)(C#N)NC1=NC(=NC(=N1)NC2CC2)Cl | 0 | validation |
| 144212220 | COP(=S)(C1=CC=CC=C1)OC2=CC(=C(C=C2Cl)Br)Cl | 1 | test |
| 144212219 | CN(C)C(=O)NC1=CC=C(C=C1)OC2=CC=C(C=C2)Cl | 1 | train |
| 144212218 | CCCN(CCC)C1=C(C=C(C=C1[N+](=O)[O-])S(=O)(=O)C)[N+](=O)[O-] | 1 | train |
| 144212217 | CC(=O)CC(=O)NC1=CC=C(C=C1)S(=O)(=O)[O-].[K+] | 0 | validation |
| 144212216 | C1=CC=C(C(=C1)C(F)(F)F)NNC2=C3C(=CC(=CC3=O)S(=O)(=O)[O-])C=CC2=N.[Na+] | 0 | test |
| 144212215 | CC(=CCCC(=CC=NO)C)C | 0 | train |
| 144212214 | CC1=CC=CC=C1S(=O)(=O)[O-].[Na+] | 0 | train |
| 144212213 | C1=CC=C(C=C1)C(=O)OCCOCCOCCOC(=O)C2=CC=CC=C2 | 0 | validation |
| 144212212 | CC1CCC(CC1S)C(C)CS | 0 | test |
| 144212211 | CCOP(=O)(CN(CCO)CCO)OCC | 0 | train |
| 144212210 | CCCCCCCCSCC | 0 | train |
| 144212209 | CNC(=O)NC1=CC(=C(C=C1)Cl)Cl | 1 | validation |
| 144212208 | C1C2=CC=CC=C2C3=C1C=C(C=C3)NC(=O)C(F)(F)F | 1 | test |
| 144212207 | CC1=CC(=CC=C1)NC(=S)N(C)C | 0 | train |
| 144212206 | COC1=CC=C(C=C1)C(=O)/C(=C¥C(=O)[O-])/Br.[Na+] | 1 | train |
| 144212205 | CCC(C)(C)C1CCCCC1OC(=O)C | 0 | validation |
| 144212204 | C1=CC(=CC=C1CCO)NC2=C3C(=C(C=C2)O)C(=O)C4=C(C=CC(=C4C3=O)[N+](=O)[O-])O | 1 | test |
| 144212203 | C1=CC=C(C=C1)NC2=C(C=C(C=C2)NC3=C(C=C(C=C3)[N+](=O)[O-])[N+](=O)[O-])S(=O)(=O)[O-].[Na+] | 0 | train |
| 144212202 | CC1=CC(=C(C=C1N=NC2=CC=C(C=C2)S(=O)(=O)[O-])OC)N=NC3=CC=C(C=C3)OC.[Na+] | 0 | train |
| 144212201 | CC1=CC(=CC=C1)OCCOC2=CC=CC(=C2)C | 1 | validation |
| 144212200 | C1=CC=C(C=C1)C(=O)NOCC(=O)O | 0 | test |
| 144212199 | CC(C(=O)O)OC1=C(C=CC(=C1)Cl)Cl | 0 | train |
| 144212198 | CC(CCOC(=O)C)CC(C)(C)C | 0 | train |
| 144212197 | C1=C(OC(=C1)[N+](=O)[O-])C2=NNC(=O)O2 | 1 | validation |
| 144212196 | C1CCC(CC1)C2CCC(=O)CC2 | 0 | test |
| 144212195 | CCCCCC1CCCC(=O)O1 | 0 | train |
| 144212194 | CC(C(CO)C(C)O)O | 0 | train |
| 144212193 | CN(C)N=NC1=CC=CC=C1 | 0 | validation |
| 144212192 | CCN(CC)CCOC1=CC=C(C=C1)C(=C(C2=CC=CC=C2)Cl)C3=CC=CC=C3.C(C(=O)O)C(CC(=O)O)(C(=O)O)O | 1 | test |
| 144212191 | C1=CC=C(C(=C1)C(=O)NC(=O)NC2=CC=C(C=C2)OC(F)(F)F)Cl | 0 | train |
| 144212190 | CC1=C(C=NO1)C(=O)NC2=CC=C(C=C2)C(F)(F)F | 1 | train |
| 144212189 | CC1=CN=C2C(=C1)C=CC(=C2C(=O)O)Cl | 0 | validation |

| | | | |
|---|---|---|---|
| 144212188 | CCCCC(CC)COCCO | 0 | test |
| 144212187 | CCCCC[Si](OCC)(OCC)OCC | 0 | train |
| 144212186 | CC(C)N(CCO)C(C)C | 0 | train |
| 144212185 | CC(=O)O[Al]OC(=O)C.O | 0 | validation |
| 144212184 | C1=CC(=CC(=C1)[N+](=O)[O-])C(=O)[O-].[Na+] | 0 | test |
| 144212183 | C([C@@H]1[C@@H]([C@@H]([C@H]([C@@H](O1)O[C@@H]2[C@H](O[C@H]([C@@H]([C@H]2O)O)O)CO)O)O)O)O | 0 | train |
| 144212182 | C(CN(CC#N)CC#N)N(CC#N)CC#N | 0 | train |
| 144212181 | CC(C)C1=CC2=CC[C@@H]3[C@@]([C@H]2CC1)(CCC[C@@]3(C)C(=O)[O-])C.[Na+] | 1 | validation |
| 144212180 | CC1CC(=CC(C1C=O)C)C | 0 | test |
| 144212179 | CC1=CC(=C(C=C1)NN=C2C(=O)C=CC(=NNC3=CC=C(C=C3)S(=O)(=O)[O-])C2=O)C.[Na+] | 0 | train |
| 144212178 | C1=CC(=CC=C1C(=O)O)[Hg]Cl | 1 | train |
| 144212177 | CC(=O)CC(C1=CC=C(C=C1)Cl)C2=C(C3=CC=CC=C3OC2=O)O | 0 | validation |
| 144212176 | [Ca+2].[Br-].[Br-] | 0 | test |
| 144212175 | CCOP(=S)(OCC)OC1=CC=C(C=C1)S(=O)C | 1 | train |
| 144212174 | CC(C(=O)O)OC1=CC=C(C=C1)OC2=C(C=C(C=N2)C(F)(F)F)Cl | 0 | train |
| 144212173 | C1C(C2(C(=C(C1(C2(Cl)Cl)Cl)Cl)Cl)Cl)CBr | 1 | validation |
| 144212172 | CC1=CC(=NN1C(=O)N(C)C)OC(=O)N(C)C | 0 | test |
| 144212171 | CCOC(=O)NC1=CC(=CC=C1)OC(=O)NC2=CC=CC=C2 | 1 | train |
| 144212170 | CC1=C(C(=CC=C1)C)N(C(=O)COC)N2CCOC2=O | 0 | train |
| 144212169 | C1COC(O1)(CN2C=NC=N2)C3=C(C=C(C=C3)Cl)Cl | 0 | validation |
| 144212168 | CC(C)(C)C(=NOC(=O)NC)CSC | 1 | test |
| 144212167 | CS(=O)(=O)NC1=C(C=C(C=C1)[N+](=O)[O-])OC2=CC=CC=C2 | 0 | train |
| 144212166 | CC[C@H]1C(=O)N(CC(=O)N([C@H](C(=O)N[C@H](C(=O)N([C@H](C(=O)N[C@H](C(=O)N[C@@H](C(=O)N([C@H](C(=O)N([C@H](C(=O)N([C@H](C(=O)N([C@H](C(=O)N1)[C@@H]([C@H](C)C/C=C/C)O)C(C)C)C)CC(C)C)C)CC(C)C)C)C)CC(C)C)C(C)C)CC(C)C)C)C | 0 | train |
| 144212165 | COC1=CC=C(C=C1)C2=COC3=C(C2=O)C=CC(=C3)O | 1 | validation |
| 144212164 | C1=CN=CC=C1C2=CNC(=O)C(=C2)N | 0 | test |
| 144212163 | C[C@H](CC1=CC=CC=C1)N(C)CC#C.Cl | 0 | train |
| 144212162 | CC(C)(C(=O)O)OC1=CC=C(C=C1)CCNC(=O)C2=CC=C(C=C2)Cl | 0 | train |
| 144212161 | CC(C(=O)C1=CC(=CC=C1)Cl)NC(C)(C)C.Cl | 0 | validation |
| 144212160 | CC1=CC(=NC(=N1)NC(=O)NS(=O)(=O)C2=CC=CC=C2C(=O)OC)C | 0 | test |
| 144212159 | CN(C)C(=O)C1=C(N=CC=C1)S(=O)(=O)NC(=O)NC2=NC(=CC(=N2)OC)OC | 0 | train |
| 144212158 | CC#C[C@@]1(CC[C@@H]2[C@@]1(C[C@@H](C3=C4CCC(=O)C=C4CC[C@@H]23)C5=CC=C(C=C5)N(C)C)C)O | 0 | train |
| 144212157 | C[C@@H]1[C@H]2C[C@]2(CC1=O)C(C)C | 0 | validation |
| 144212156 | CCC/C=C/CO | 0 | test |
| 144212155 | CCCCCCCCCCCC(=O)C | 0 | train |
| 144212154 | C[C@]12CC[C@H]3[C@H]([C@@H]1CC[C@]2(C#C)O)CCC4=C3C=CC(=C4)OC | 1 | train |
| 144212153 | CC1=C(C(=CC=C1)N)C | 1 | validation |
| 144212152 | CC[C@@H]1[C@@]([C@@H]([C@H](C(=NOCOCCOC)[C@@H](C[C@@]([C@@H]([C@H]([C@@H]([C@H](C(=O)O1)C)O[C@H]2C[C@@]([C@H]([C@@H](O2)C)O)(C)OC)C)O[C@H]3[C@@H]([C@H](C[C@H](O3)C)N(C)C)O)(C)O)C)C)O)(C)O | 0 | test |
| 144212151 | CC1=NC=C(N=C1)C(=O)NCCC2=CC=C(C=C2)S(=O)(=O)NC(=O)NC3CCCCC3 | 0 | train |
| 144212150 | CCCCCCCCCCCCCCCC[N+](C)(C)CC1=CC=CC=C1.[Cl-] | 0 | train |
| 144212149 | CC1=C(C(C(=C(N1)C)C(=O)OCCN(C)CC2=CC=CC=C2)C3=CC(=CC=C3)[N+](=O)[O-])C(=O)OC.Cl | 1 | validation |
| 144212148 | CCOCC(C)O | 0 | test |
| 144212147 | CC(C)CC(C)CC(C)CC(C)COC(=O)C1=CC=CC=C1C(=O)OCC(C)CC(C)CC(C)CC(C)C | 1 | train |
| 144212146 | CC1=CC=NC2=CC=CC=C12 | 1 | train |
| 144212145 | COC(=O)NC1=NC2=C(N1)C=C(C=C2)SC3=CC=CC=C3 | 1 | validation |
| 144212144 | C1=C(N=C(S1)N=C(N)N)CSCCC(=NS(=O)(=O)N)N | 0 | test |
| 144212143 | CCCC/C=C/C=C¥C=O | 0 | train |
| 144212142 | CC/C=C¥CC1=C(CCC1=O)C | 0 | train |
| 144212141 | CCCC/C=C¥CCCCCCCCCC=O | 0 | validation |
| 144212140 | CCC(C)C1=NC=CN=C1OC | 0 | test |
| 144212139 | CC(C(C)S)S | 0 | train |
| 144212138 | CCCC(=O)OC | 0 | train |
| 144212137 | CCC1=NC=CN=C1CC | 0 | validation |
| 144212136 | CN(C)CCCN1C2=CC=CC=C2CCC3=C1C=C(C=C3)Cl.Cl | 1 | test |

| | | | |
|---|---|---|---|
| 144212135 | C[C@]12CC[C@H]3[C@H]([C@@H]1CC[C@@H]2OC(=O)CCC4CCCC4)CCC5=C3C=CC(=C5)O | 1 | train |
| 144212134 | CCCCNC(=O)NS(=O)(=O)C1=CC=C(C=C1)N | 0 | train |
| 144212133 | CC1=NN(C(=O)C1)C2=CC=C(C=C2)C(=O)O | 1 | validation |
| 144212132 | C1=CN2[C@H]3[C@H]([C@@H]([C@H](O3)CO)O)OC2=NC1=N | 0 | test |
| 144212131 | CCNC1=NC(=NC(=N1)OC)NC(C)C | 0 | train |
| 144212130 | CC(C)CC(C)(C#N)N | 0 | train |
| 144212129 | C1CC(OC1)N2C=C(C(=O)NC2=O)F | 0 | validation |
| 144212128 | CC1=CC(=C(C=C1Cl)C)Cl | 0 | test |
| 144212127 | CCC(CC)NC1=CC(=C(C=C1)C)C | 0 | train |
| 144212126 | CCO[Si](CCCNC(=O)N)(OCC)OCC | 0 | train |
| 144212125 | C1=CC=C(C=C1)OP(=O)(O)O | 0 | validation |
| 144212124 | CC1CN1P(=O)(N2CC2C)N3CC3C | 0 | test |
| 144212123 | COC(=O)NCCC[Si](OC)(OC)OC | 0 | train |
| 144212122 | C=CCS(=O)(=O)[O-].[Na+] | 0 | train |
| 144212121 | C[Si](C)(C)O[Si](O)(O)O | 0 | validation |
| 144212120 | CC(=C)C(=O)OCCCCCCCCCCOC(=O)C(=C)C | 1 | test |
| 144212119 | C1=CC=C(C=C1)NC2=CC3=CC(=CC(=C3C=C2)O)S(=O)(=O)O | 0 | train |
| 144212118 | C1=C(C(=CC(=C1Cl)Cl)Cl)S(=O)(=O)O | 0 | train |
| 144212117 | CC1(CN(NC1=O)C2=CC=CC=C2)CO | 1 | validation |
| 144212116 | CCN(CC)C1CCCCC1 | 0 | test |
| 144212115 | CCCCNS(=O)(=O)C1=CC=C(C=C1)C | 1 | train |
| 144212114 | CCCCN(CCCC)C(=S)[S-].[Na+] | 0 | train |
| 144212113 | C(C(CCl)O)S(=O)(=O)[O-].[Na+] | 0 | validation |
| 144212112 | CCCC[Sn](CCCC)(CCCC)OC(=O)C=C | 1 | test |
| 144212111 | CCOC(=O)NCCC[Si](OCC)(OCC)OCC | 1 | train |
| 144212110 | CC(C)O.CC(C)O.C(CO)N(CCO)CCO.C(CO)N(CCO)CCO.[Ti] | 0 | train |
| 144212109 | C1COC(O1)CON=C(C2=CC=C(C=C2)Cl)C(F)(F)F | 0 | validation |
| 144212108 | CCC(C)(CCC(C)C)C(=O)[O-].CCC(C)(CCC(C)C)C(=O)[O-].[Ca+2] | 0 | test |
| 144212107 | CCC(C)(CCC(C)C)C(=O)O[Sn](C)(C)OC(=O)C(C)(CC)CCC(C)C | 0 | train |
| 144212106 | CP(=O)([O-])OCCC[Si](O)(O)O.[Na+] | 0 | train |
| 144212105 | C=CCOC(=O)C1=CC(=C(C=C1)C(=O)OCC=C)C(=O)OCC=C | 0 | validation |
| 144212104 | CCOC(=O)/C=C(/O)¥C.CCOC(=O)/C=C(/O)¥C.CC(O)C.CC(O)C.[Ti] | 0 | test |
| 144212103 | C[Si](NC1CCCCC1)(NC2CCCCC2)NC3CCCCC3 | 0 | train |
| 144212102 | CC1=CCC(CC1)(C(C)C)O | 0 | train |
| 144212101 | CCCCOC(=O)C(C)OC(=O)CCC | 0 | validation |
| 144212100 | CCC(=O)OCC1=CC=CC=C1 | 0 | test |
| 144212099 | C=CCOC(=O)CCC1CCCCC1 | 0 | train |
| 144212098 | CC(=O)OC(C)(C)CC1=CC=CC=C1 | 0 | train |
| 144212097 | CCCCC1=C(NC(=NC1=O)NCC)C | 0 | validation |
| 144212096 | CCCCCCC(=O)OCC=C | 0 | test |
| 144212095 | COP(=S)(OC)OC1=CC=C(C=C1)SC2=CC=C(C=C2)OP(=S)(OC)OC | 1 | train |
| 144212094 | CCCCCCCCOCCCN | 0 | train |
| 144212093 | CC1C2CC(C1(C)C)CC2C3CCCC(C3)O | 0 | validation |
| 144212092 | CCC(=O)OC(C)(C)C1CCC(=CC1)C | 0 | test |
| 144212091 | CCC(C)C1CCCCC1=O | 0 | train |
| 144212090 | C1=CC=C(C=C1)CCCC2=CC=CC=N2 | 1 | train |
| 144212089 | CCCCN(CCC(=O)OCC)C(=O)C | 0 | validation |
| 144212088 | CC(=O)NC1=C2C(=CC(=C1)S(=O)(=O)[O-<br>])C=C(/C(=N/NC3=CC=CC=C3OC)/C2=O)S(=O)(=O)[O-].[Na+].[Na+] | 0 | test |
| 144212087 | CCCCC1=CC2=C(C=CC=C2S(=O)(=O)[O-])C=C1.[Na+] | 0 | train |
| 144212086 | COCCCCCCCCCOCCC[Si](C)(O[Si](C)(C)C)O[Si](C)(C)C | 0 | train |
| 144212085 | CC1=C(C=C(C=C1)C(C)C2=CC(=C(C=C2)C)C)C | 0 | validation |
| 144212084 | CCC(C)(C)C1=CC(=C(C=C1O)C(C)(C)CC)O | 0 | test |
| 144212083 | CC1=CC(=C(C=C1)S(=O)(=O)[O-])C.[Na+] | 0 | train |
| 144212082 | CCOC(=S)[S-].[Na+] | 1 | train |
| 144212081 | CCCCCCCC/C=C/CCCOC(=O)C | 0 | validation |
| 144212080 | C1CCC(CC1)C2CCC(CC2)O | 0 | test |
| 144212079 | CC(C)C1=CC=C(C=C1)S(=O)(=O)O | 0 | train |
| 144212078 | CC(C)CP(=S)(CC(C)C)[S-].[Na+] | 0 | train |
| 144212077 | C1=CC(=CC=C1C2=CSC(=N2)NN)[N+](=O)[O-] | 1 | validation |
| 144212076 | CC(=C)O[Si](C=C)(OC(=C)C)OC(=C)C | 0 | test |

| | | | |
|---|---|---|---|
| 144212075 | C(CSCSCCO)O | 0 | train |
| 144212074 | CCCCCCCCCCCCN1CCCCCC1=O | 1 | train |
| 144212073 | C1C2=CC=CC=C2N(C1=O)C3=C(C=CC=C3Cl)Cl | 1 | validation |
| 144212072 | CN1CC(C2=C(C1)C(=CC=C2)N)C3=CC=CC=C3 | 0 | test |
| 144212071 | C([C@H]([C@@H]([C@H]([C@H](CO)O)O)O)O)O | 0 | train |
| 144212070 | CCN(CC#N)C1=CC=CC(=C1)C | 0 | train |
| 144212069 | C1=CC=C2C(=C1)C(=O)C3=C(C=C(C(=C3C2=O)N)S(=O)(=O)O)Br | 0 | validation |
| 144212068 | COP(=O)(OC)O/C(=C¥Cl)/C1=CC(=C(C=C1Cl)Cl)Cl | 1 | test |
| 144212067 | CC(C)(C)C1=CC(=CC(=C1O)C(C)(C)C)[N+](=O)[O-] | 1 | train |
| 144212066 | CN(C)CCCNCCC#N | 0 | train |
| 144212065 | CC(C)(C)NC(=O)OC1=CC=CC(=C1)NC(=O)N(C)C | 0 | validation |
| 144212064 | C(C(CO[N+](=O)[O-])(CO[N+](=O)[O-])CO[N+](=O)[O-])O[N+](=O)[O-] | 0 | test |
| 144212063 | CC1=CC(=C(C=C1)NC=NC)C | 0 | train |
| 144212062 | CCCCNC1CC(NC(C1)(C)C)(C)C | 0 | train |
| 144212061 | C1=C(C=NC(=C1Cl)Cl)C(Cl)(Cl)Cl | 1 | validation |
| 144212060 | C1=CC(=CC=C1C2=CSC(=N2)NN)N | 1 | test |
| 144212059 | CCC(CO)(CO)[N+](=O)[O-] | 0 | train |
| 144212058 | CCOP(=S)(OCC)SCSC1=C(C=CC(=C1)Cl)Cl | 0 | train |
| 144212057 | CN(C)CCOC1=CC=C(C=C1)/C(=C(/CCCl)¥C2=CC=CC=C2)/C3=CC=CC=C3.C(C(=O)O)C(CC(=O)O)(C(=O)O)O | 1 | validation |
| 144212056 | CCCCCCCCCCOC(=O)C=C | 0 | test |
| 144212055 | CC(C)NC(=O)C1=CC=C(C=C1)CNNC | 0 | train |
| 144212054 | C1=CC(=CC(=C1)S(=O)(=O)C2=CC=CC(=C2)N)N | 0 | train |
| 144212053 | CC(C#CC(C)O)O | 1 | validation |
| 144212052 | CCN(CCO)C1=CC=CC(=C1)C | 1 | test |
| 144212051 | CC(=C)C(=O)OCCOCCOC(=O)C(=C)C | 0 | train |
| 144212050 | CC(=O)O[C@@H]1CC2CCC1(C2(C)C)C | 0 | train |
| 144212049 | C1C(O1)COCCOCC2CO2 | 0 | validation |
| 144212048 | CC(C)(C1=CC(=C(C(=C1)Br)OCCO)Br)C2=CC(=C(C(=C2)Br)OCCO)Br | 1 | test |
| 144212047 | C=CC(=O)OCCN1C(=O)N(C(=O)N(C1=O)CCOC(=O)C=C)CCOC(=O)C=C | 1 | train |
| 144212046 | CC(CC(C)(C)CCN)CN | 0 | train |
| 144212045 | C=CC(=O)OCC1CCCO1 | 0 | validation |
| 144212044 | CC1CC(=O)CC(C1)(C)C | 0 | test |
| 144212043 | C[Si](C)(C=C)O[Si](C)(C)C=C | 0 | train |
| 144212042 | COC1=CC(=C(C=C1N)OC)Cl | 1 | train |
| 144212041 | CC(=O)NCCO | 0 | validation |
| 144212040 | C=CC(=O)OCCOC1=CC=CC=C1 | 0 | test |
| 144212039 | C=CC(=O)OCC(CO)(COCC(COC(=O)C=C)(COC(=O)C=C)COC(=O)C=C)COC(=O)C=C | 1 | train |
| 144212038 | CN(C)CCN(C)CCO | 0 | train |
| 144212037 | CCCCCCOC(=O)C=C | 0 | validation |
| 144212036 | C1=CC=C(C=C1)CCCC2=CC=NC=C2 | 0 | test |
| 144212035 | CC(=C)C(=O)OCCNC(C)(C)C | 0 | train |
| 144212034 | CC(=C)CS(=O)(=O)[O-].[Na+] | 0 | train |
| 144212033 | CC(=C)C1=CC(=CC=C1)C(C)(C)N=C=O | 0 | validation |
| 144212032 | CC(=C)C(=O)OCC1CCCO1 | 0 | test |
| 144212031 | CCCCOC(=O)CCC(=O)C | 0 | train |
| 144212030 | COC(=O)CCCCCCCCC(=O)OC | 0 | train |
| 144212029 | CC(=C)C(=O)OCC1=CC=CC=C1 | 0 | validation |
| 144212028 | C=CCOC1=C(C=C(C=C1Br)Br)Br | 1 | test |
| 144212027 | C[C@@]1([C@H]2C[C@H]3[C@@H](C(=O)C(=C([C@]3(C(=O)C2=C(C4=C(C=CC(=C41)Cl)O)O)O)O)O)C(=O)N)N(C)C)O.Cl | 1 | train |
| 144212026 | C1CC(CCC1COCC2CO2)COCC3CO3 | 0 | train |
| 144212025 | C=COCCOCCOCCOC=C | 0 | validation |
| 144212024 | CCC(CC1=CC=CC=C1)(C(=O)C2=CC=C(C=C2)N3CCOCC3)N(C)C | 1 | test |
| 144212023 | C=CC(=O)OCC(COC1=CC=CC=C1)O | 0 | train |
| 144212022 | CCOC(=O)CCCCCCCCC=C | 0 | train |
| 144212021 | CC(CN)O | 0 | validation |
| 144212020 | CCC(C)NC(=O)N | 0 | test |
| 144212019 | CCCCCCCCCCCCN.Cl | 1 | train |
| 144212018 | C1=CC(=CC=C1C(=O)OCCCOC(=O)C2=CC=C(C=C2)N)N | 1 | train |
| 144212017 | C1CC(=O)N(C1)CCO | 0 | validation |
| 144212016 | CCCCCC1=CC=C(C=C1)N | 0 | test |

| | | | |
|---|---|---|---|
| 144212015 | CC1=NN(C(=O)C1)C2=CC(=CC=C2)S(=O)(=O)O | 0 | train |
| 144212014 | CN(C1CCCCC1)S(=O)(=O)C2=CC=CC=C2N | 1 | train |
| 144212013 | C1=C(C=C(C(=C1Cl)O)C(=O)O)Cl | 0 | validation |
| 144212012 | C1=CC2=C(C=C1C3=CC4=C(C=C3)C(=O)OC4=O)C(=O)OC2=O | 0 | test |
| 144212011 | CCC1=CC2=C(C=C1)C(=O)C3=CC=CC=C3C2=O | 1 | train |
| 144212010 | C1=CC=C(C(=C1)OC(F)(F)F)S(=O)(=O)N | 0 | train |
| 144212009 | CC/C=C¥CCOC(=O)C1=CC=CC=C1O | 0 | validation |
| 144212008 | CN(C)CCCNCCCN(C)C | 0 | test |
| 144212007 | CCC(CO)(CO)COCC(CC)(CO)CO | 0 | train |
| 144212006 | CC(COC(=O)C)OC(=O)C | 0 | train |
| 144212005 | C1CCNC(C1)CCO | 0 | validation |
| 144212004 | CCCCS(=O)(=O)Cl | 0 | test |
| 144212003 | CC(C)(C)C1=CC(=CC=C1)O | 0 | train |
| 144212002 | C=CC(=O)OCC(COC(=O)C=C)(COC(=O)C=C)COC(=O)C=C | 1 | train |
| 144212001 | C1=CC=C(C=C1)C2=NNN=N2 | 0 | validation |
| 144212000 | CC(=C)C(=O)OCC(COC1=CC=C(C=C1)C(C)(C)C2=CC=C(C=C2)OCC(COC(=O)C(=C)C)O)O | 1 | test |
| 144211999 | CCCCC(CC)CO[P+](=O)OCC(CC)CCCC | 0 | train |
| 144211998 | CC(=C)C(=O)OCCCCOC(=O)C(=C)C | 0 | train |
| 144211997 | CC(C)(C(=O)O)OC1=CC=C(C=C1)Cl | 0 | validation |
| 144211996 | CCN(CC1=CC(=CC=C1)S(=O)(=O)[O-<br>])C2=CC=C(C=C2)C(=C3C=CC(=[N+](CC)CC4=CC(=CC=C4)S(=O)(=O)[O-<br>])C=C3)C5=CC=CC=C5.[Na+] | 0 | test |
| 144211995 | C(COCC#CCOCCO)O | 0 | train |
| 144211994 | CCOC(=O)C(=C(C1=CC=CC=C1)C2=CC=CC=C2)C#N | 1 | train |
| 144211993 | C=COCCCCO | 0 | validation |
| 144211992 | CC(C)(C(=O)C1=CC=CC=C1)O | 0 | test |
| 144211991 | C1=CC=C(C=C1)CCCO | 0 | train |
| 144211990 | CC(C)(C)C1=CC=C(C=C1)C=C | 0 | train |
| 144211989 | CC1=C(C(=C(C=C1Br)Br)OC)Br | 0 | validation |
| 144211988 | CC(=C)C(=O)NCCCN(C)C | 0 | test |
| 144211987 | CCCCCCCCC(CCCCCC)C(=O)O | 0 | train |
| 144211986 | C=CC(=O)OCCOC(=O)CCC(=O)O | 0 | train |
| 144211985 | CC(C)C1N(CCO1)CCOC(=O)NCCCCCCNC(=O)OCCN2CCOC2C(C)C | 0 | validation |
| 144211984 | CC1=CC(=C(C(=C1C(=O)OC)O)C)O | 1 | test |
| 144211983 | C1=CC=C(C=C1)S(=O)(=O)C2=CC=C(C=C2)Cl | 1 | train |
| 144211982 | C1=CC=C(C=C1)OP(=O)(OC2=CC=CC=C2)Cl | 0 | train |
| 144211981 | CC(C)(C(=O)C1=CC=C(C=C1)SC)N2CCOCC2 | 1 | validation |
| 144211980 | CC(=C)C(=O)OCC(C)(C)COC(=O)C(=C)C | 0 | test |
| 144211979 | CCC(CO)(COCC=C)COCC=C | 0 | train |
| 144211978 | C1CC(CC(C1)CN=C=O)CN=C=O | 0 | train |
| 144211977 | CCCCCCCCCCC1CO1 | 0 | validation |
| 144211976 | CC1(C(=O)N(C(=O)N1Cl)Br)C | 0 | test |
| 144211975 | C=CC(=O)N1CCOCC1 | 0 | train |
| 144211974 | C1=CC=C2C=C(C=CC2=C1)S(=O)(=O)O | 0 | train |
| 144211973 | C1=CC(=CC=C1C(=O)N)N | 0 | validation |
| 144211972 | C1=CC=C2C(=C1)C(=CC(=C2O)C(=O)O)O | 1 | test |
| 144211971 | CC1=C(C=CC(=C1)S(=O)(=O)[O-])N/N=C/2¥C(=O)C=CC3=CC=CC=C32.[Na+] | 0 | train |
| 144211970 | C1C(O1)COC2=CC=C(C=C2)C(C3=CC=C(C=C3)OCC4CO4)C5=CC=C(C=C5)OCC6CO6 | 0 | train |
| 144211969 | C1C(COCO1)(CO)CO | 0 | validation |
| 144211968 | CC(=C)C(=O)OCCOP(=O)(O)OCCOC(=O)C(=C)C | 0 | test |
| 144211967 | C1=CC=C(C=C1)OC(=O)C2=C(C3=CC=CC=C3C=C2)O | 1 | train |
| 144211966 | CCCCOC(=O)CCS | 0 | train |
| 144211965 | CC1=CC[C@@H](C1(C)C)CC=O | 0 | validation |
| 144211964 | CCC(=O)NC1=CC=CC(=C1)N | 0 | test |
| 144211963 | CC1=CC(=C(C=C1)NC(=O)C)C | 0 | train |
| 144211962 | C[C@@]12CC[C@@H](C1(C)C)C[C@H]2O | 0 | train |
| 144211961 | CC1C=C(CCC1C=O)C | 0 | validation |
| 144211960 | C1=CC=C(C=C1)OC2=CC=CC(=C2)CBr | 1 | test |
| 144211959 | CC(CC1=CC2=C(C=C1)OCO2)C=O | 0 | train |
| 144211958 | CC1=CC=CC=C1S(=O)(=O)N | 0 | train |
| 144211957 | C1=C(C=C(C(=C1Cl)O)Cl)N | 1 | validation |
| 144211956 | CCC(COC(=O)CCS)(COC(=O)CCS)COC(=O)CCS | 0 | test |

| | | | |
|---|---|---|---|
| 144211955 | CC1=C(C=C(C=C1)C=O)C | 0 | train |
| 144211954 | C1=CC=C(C(=C1)NC(=O)C2=C(C=CC(=C2)Cl)O | 1 | train |
| 144211953 | CC(=C)C(=O)[O-].[Na+] | 0 | validation |
| 144211952 | CCC(C)(C)C1CCC(CC1)O | 0 | test |
| 144211951 | C1=CC=C2C(=C1)C(=O)OC23C4=CC(=C(C(=C4OC5=C(C(=C(C=C35)I)O)I)I)O)I | 0 | train |
| 144211950 | C1=CC(=C(C=C1C=O)Br)F | 0 | train |
| 144211949 | C1=CN=CC=C1CCCC2=CC=NC=C2 | 0 | validation |
| 144211948 | CC(=O)CCC1=CC=C(C=C1)OC(=O)C | 0 | test |
| 144211947 | C1=CC(=C(C(=C1)Cl)CCl)F | 1 | train |
| 144211946 | CC1=CC=C(C=C1)C(=O)C2=CC=CC=C2 | 1 | train |
| 144211945 | C(=C(I)I)(I)I | 1 | validation |
| 144211944 | CC1=C(C(=C(C(=C1[N+](=O)[O-])C(C)(C)C)[N+](=O)[O-])C)C(=O)C | 1 | test |
| 144211943 | C1=CC=C(C=C1)S(=O)(=O)C2=CC=CC=C2N | 1 | train |
| 144211942 | CCCCC(CC)C(=O)[O-].CCCCC(CC)C(=O)[O-].[Ba+2] | 0 | train |
| 144211941 | C1=CC=C(C(=C1)S(=O)(=O)N)Cl | 0 | validation |
| 144211940 | CC(CCO)OC | 0 | test |
| 144211939 | CCN1CN(CN(C1)CC)CC | 0 | train |
| 144211938 | C1=CN=CC=C1CCO | 0 | train |
| 144211937 | CC(C)CCOC(=O)CC(C)C | 0 | validation |
| 144211936 | CC(=CCCC(=CC(OC)OC)C)C | 0 | test |
| 144211935 | CCOCCOCCOC(=O)C=C | 0 | train |
| 144211934 | CC(=O)OC1=CC=C(C=C1)C=C | 1 | train |
| 144211933 | COC(=O)C1=C(C=CC(=C1)OC2=C(C=C(C=C2)Cl)Cl)[N+](=O)[O-] | 1 | validation |
| 144211932 | CC1=CC(=CC=C1)NC2=CC=CC=C2 | 1 | test |
| 144211931 | CC1(C(C(C1O)(C)C)O)C | 0 | train |
| 144211930 | C1CCOC(C1)N2C=NC3=C2N=CN=C3NCC4=CC=CC=C4 | 1 | train |
| 144211929 | C1=CC(=C(C=C1C=O)[N+](=O)[O-])Cl | 0 | validation |
| 144211928 | C1CC2C3CC(C2C1CO)CC3CO | 0 | test |
| 144211927 | C1=CC=C(C=C1)COC(=O)N=NC(=O)OCC2=CC=CC=C2 | 1 | train |
| 144211926 | C1(=NC(=S)NN1)N | 0 | train |
| 144211925 | CCOC1=CC(=C(C=C1)N)[N+](=O)[O-] | 0 | validation |
| 144211924 | CC(CCOC(=O)C(=C)C)OC(=O)C(=C)C | 0 | test |
| 144211923 | C(C(CO)(CO)COCC(CO)(CO)CO)O | 0 | train |
| 144211922 | CCCOC(=O)C1=CC=CC=C1 | 0 | train |
| 144211921 | C[C@]12CC[C@H]3[C@H]([C@@H]1C[C@H]([C@@H]2O)O)CCC4=C3C=CC(=C4)O | 0 | validation |
| 144211920 | C1=CC(=NC(=C1)Cl)C(=O)O | 0 | test |
| 144211919 | C(CSCCS)S | 0 | train |
| 144211918 | C1=CC(=CC2=CC(=C(C=C21)O)S(=O)(=O)[O-])S(=O)(=O)[O-].[Na+].[Na+] | 0 | train |
| 144211917 | CCCCP(CCCC)CCCC | 0 | validation |
| 144211916 | C(C(C(=O)O)S(=O)(=O)O)C(=O)O | 0 | test |
| 144211915 | CC(=O)/C=C/C1=CC=CC=C1 | 0 | train |
| 144211914 | CC1=CC(=NC=C1)N | 0 | train |
| 144211913 | COC1=CC(=C(C=C1)N)[N+](=O)[O-] | 0 | validation |
| 144211912 | [I-].[I-].[Hg+2] | 1 | test |
| 144211911 | CNC(=C[N+](=O)[O-])NCCSCC1=CSC(=N1)CN(C)C | 0 | train |
| 144211910 | CCCCCCCCCCCCOCCO | 1 | train |
| 144211909 | CCC(C)COC(=O)CC(C)C | 0 | validation |
| 144211908 | C[C@@]12CCN([C@@H]1N(C3=C2C=C(C=C3)OC(=O)NC)C)C | 0 | test |
| 144211907 | CN(C)[C@H]1[C@@H]2C[C@@H]3CC4=C(C=CC(=C4C(=C3C(=O)[C@@]2(C(=C(C1=O)C(=O)N)O)O)O)O)N(C)C.Cl | 0 | train |
| 144211906 | CC1(C(=O)N(C(=O)N1)C2=CC(=C(C=C2)[N+](=O)[O-])C(F)(F)F)C | 0 | train |
| 144211905 | C1C(COCO1)([N+](=O)[O-])Br | 0 | validation |
| 144211904 | CC[N+](CC)(CC1=CC=CC=C1)CC(=O)NC2=C(C=CC=C2C)C.C1=CC=C(C=C1)C(=O)[O-] | 0 | test |
| 144211903 | C1CN(CCC1N2C3=CC=CC=C3NC2=O)CCCC(C4=CC=C(C=C4)F)C5=CC=C(C=C5)F | 1 | train |
| 144211902 | C1=CC2=C3C(=C1)C(=O)NC(=O)C3=CC=C2 | 0 | train |
| 144211901 | C1=CC=C2C(=C1)C3=CC=CC=C3C2(C4=CC=C(C=C4)O)C5=CC=C(C=C5)O | 1 | validation |
| 144211900 | CCOC(=N)CC(=O)OCC.Cl | 0 | test |
| 144211899 | CCOC(=O)CCN(C(C)C)SN(C)C(=O)OC1=CC=CC2=C1OC(C2)(C)C | 1 | train |
| 144211898 | C1[C@@H]2[C@H](COS(=O)O1)[C@@]3(C(=C([C@]2(C3(Cl)Cl)Cl)Cl)Cl)Cl | 1 | train |
| 144211897 | CCOP(=S)(OCC)O/N=C(¥C#N)/C1=CC=CC=C1 | 1 | validation |
| 144211896 | CCOCN1C(=C(C(=C1C(F)(F)F)Br)C#N)C2=CC=C(C=C2)Cl | 1 | test |
| 144211895 | CCOP(=S)(OCC)OC1=NN(C=N1)C2=CC=CC=C2 | 1 | train |

| | | | |
|---|---|---|---|
| 144211894 | CCC1=CC=CC(=C1N([C@@H](C)COC)C(=O)CCl)C | 1 | train |
| 144211893 | CC1=C(C=CC(=C1)Cl)N=CN(C)C | 0 | validation |
| 144211892 | CCS(=O)(=O)CCSP(=O)(OC)OC | 0 | test |
| 144211891 | C1=CC=C2C(=C1)NC(=N2)C3=CC=CO3 | 1 | train |
| 144211890 | CCOP(=O)(OCC)OC(=CCl)C1=C(C=C(C=C1)Cl)Cl | 1 | train |
| 144211889 | CCC1=NC(=CC(=N1)OP(=S)(OC)OC)OCC | 0 | validation |
| 144211888 | C[C@@H]1CN(C[C@@H](O1)C)CC(C)CC2=CC=C(C=C2)C(C)(C)C | 0 | test |
| 144211887 | CC(=CC1C(C1(C)C)C(=O)OCC2=CC(=CC=C2)OC3=CC=CC=C3)C | 1 | train |
| 144211886 | CCOC(=O)[C@@H](C)OC1=CC=C(C=C1)OC2=NC3=C(O2)C=C(C=C3)Cl | 1 | train |
| 144211885 | CCC1COC(O1)(CN2C=NC=N2)C3=C(C=C(C=C3)Cl)Cl | 1 | validation |
| 144211884 | C1=CC(=C(C(=C1)F)C(=O)NC(=O)NC2=CC(=C(C(=C2)Cl)OC3=C(C=C(C=N3)C(F)(F)F)Cl)Cl)F | 1 | test |
| 144211883 | CCCN(CC1CC1)C2=C(C=C(C=C2[N+](=O)[O-])C(F)(F)F)[N+](=O)[O-] | 1 | train |
| 144211882 | C1=CC=C(C=C1)C(C2=CC=C(C=C2)Cl)C(=O)C3C(=O)C4=CC=CC=C4C3=O | 0 | train |
| 144211881 | CCOC(=O)C(C1=CC=CC=C1)SP(=S)(OC)OC | 1 | validation |
| 144211880 | CC(C)OC(=O)C(C)N(C1=CC(=C(C=C1)F)Cl)C(=O)C2=CC=CC=C2 | 1 | test |
| 144211879 | CCNC(=O)C(C)OC(=O)NC1=CC=CC=C1 | 0 | train |
| 144211878 | CCCCCC(C)OC(=O)COC1=C2C(=C(C=C1)Cl)C=CC=N2 | 0 | train |
| 144211877 | CCC1OCC(O1)COC2=CC=C(C=C2)OC3=CC=CC=C3 | 1 | validation |
| 144211876 | CCCCOCCOC(=O)COC1=C(C=C(C=C1)Cl)Cl | 0 | test |
| 144211875 | CC(C)C1=CC(=CC=C1)O | 0 | train |
| 144211874 | CC1=C(C(=CC=C1)C)N(C(C)C(=O)OC)C(=O)CC2=CC=CC=C2 | 1 | train |
| 144211873 | C1=C(C(=O)NC(=C1Cl)Cl)Cl | 0 | validation |
| 144211872 | CCCCC(CC)CCC(CC(C)C)OS(=O)(=O)[O-].[Na+] | 0 | test |
| 144211871 | CCC(C)C1=CC(=CC(=C1OC(=O)C=C(C)C)[N+](=O)[O-])[N+](=O)[O-] | 1 | train |
| 144211870 | C1=CC=C2C(=C1)C(=O)OC23C4=CC(=C(C(=C4OC5=C(C(=C(C=C35)Br)O)Br)Br)O)Br | 0 | train |
| 144211869 | C1CN=C(N1)NC2=C(C=CC=C2Cl)Cl.Cl | 0 | validation |
| 144211868 | CCOC1=CC=C(C=C1)NC(=O)CC(=O)C | 0 | test |
| 144211867 | CCCCCCCCN1CCCC1=O | 1 | train |
| 144211866 | C1=CC(=C(C=C1Cl)Cl)CCl | 0 | train |
| 144211865 | CCCCCCCC1=CC=C(C=C1)O | 1 | validation |
| 144211864 | C[C@H]1[C@@H]([C@H]([C@H]([C@@H](O1)O[C@H]2C[C@H]([C@@]3([C@@H]4[C@@H](CC[C@@]3(C2)O)[C@]5(CC[C@@H]([C@]5(C[C@H]4O)C)C6=CC(=O)OC6)O)CO)O)O)O)O | 1 | test |
| 144211863 | CC(CCCN)CN | 0 | train |
| 144211862 | CC(=O)CC(C1=CC=CC=C1)C2=C(C3=CC=CC=C3OC2=O)[O-].[Na+] | 0 | train |
| 144211861 | CN(C)CC1=CC=CC=C1O | 0 | validation |
| 144211860 | COC1=CC(=C(C=C1N)OC)S(=O)(=O)NC2=CC=CC=C2 | 1 | test |
| 144211859 | CC(=O)C1=CC(=CC2=C1CCC2(C)C)C(C)(C)C | 1 | train |
| 144211858 | CC(C)OC(=O)CCC(=O)OC(C)C | 0 | train |
| 144211857 | C1=CC=C(C(=C1)C=CC2=CC=C(C=C2)C3=CC=C(C=C3)C=CC4=CC=CC=C4S(=O)(=O)[O-])S(=O)(=O)[O-].[Na+].[Na+] | 0 | validation |
| 144211856 | CC(C)(C)C1(CO1)CCC2=CC=C(C=C2)Cl | 0 | test |
| 144211855 | C1=CC=C(C=C1)C(=O)NC2=CC3=C(C=C2)C(=O)C(=NNC4=CC=C(C=C4)N=NC5=CC=C(C=C5)S(=O)(=O)[O-])C(=C3)S(=O)(=O)[O-].[Na+].[Na+] | 0 | train |
| 144211854 | CC(C)C1=CC2=C(C=C1)SC3=CC=CC=C3C2=O | 1 | train |
| 144211853 | CC[NH+]=C1C=CC(=C(C2=CC=C(C=C2)N(CC)CC)C3=CC=C(C=C3)N(CC)CC)C4=CC=CC=C14.[Cl-] | 1 | validation |
| 144211852 | CCCCCCCC(=O)OCC(CO)O | 0 | test |
| 144211851 | CC1=CC(=O)OC2=C1C=CC(=C2)N(C)C | 1 | train |
| 144211850 | C([C@H]([C@H]([C@@H](C=O)O)O)O)O | 0 | train |
| 144211849 | CCOC(=O)N1CCC(=C2C3=C(CCC4=C2N=CC=C4)C=C(C=C3)Cl)CC1 | 1 | validation |
| 144211848 | C1=CC(=CC=C1N)OC2=CC(=C(C=C2)N)N | 1 | test |
| 144211847 | CC(=O)[C@]1(CC[C@@H]2[C@@]1(CC[C@H]3[C@H]2CCC4=CC(=O)CC[C@]34C)C)O | 0 | train |
| 144211846 | CCCCCCCCCCCCN(CCO)CCO | 1 | train |
| 144211845 | CC1CCC(CC1)CO | 0 | validation |
| 144211844 | C[Si]1(O[Si](O[Si](O[Si](O1)(C)C=C)(C)C=C)(C)C=C)C=C | 0 | test |
| 144211843 | C1=CC=C2C(=C1)C(=O)C3=C(C2=O)C(=C(C=C3O)O)O | 1 | train |
| 144211842 | CC(=O)NC1=C2C(=CC(=C1)S(=O)(=O)[O-])C=C(/C(=N¥NC3=CC=CC=C3)/C2=O)S(=O)(=O)[O-].[Na+].[Na+] | 0 | train |
| 144211841 | CC1=CC(=C(C=C1C(C)(C)C)C(C)(C)C)O | 0 | validation |
| 144211840 | C1=CC=C(C=C1)N(CCO)CCO | 0 | test |
| 144211839 | C1=CC=C2C(=C1)C=CC=C2S(=O)(=O)[O-].[Na+] | 0 | train |

| | | | |
|---|---|---|---|
| 144211838 | CCCCCC=CCC1CC(=O)OC1=O | 0 | train |
| 144211837 | C1=CC=C(C=C1)SCCO | 0 | validation |
| 144211836 | CC(C)(C)NCCNC(C)(C)C | 0 | test |
| 144211835 | C(COCCOCCN)N | 0 | train |
| 144211834 | CC(C)(COC(=O)C1=CC=CC=C1)COC(=O)C2=CC=CC=C2 | 0 | train |
| 144211833 | CC(C)(CO)N(C)C | 0 | validation |
| 144211832 | CCCCN(CCCC)C(=O)N(CCCC)CCCC | 1 | test |
| 144211831 | CCCCOC(=O)CCC(=O)OCCCC | 0 | train |
| 144211830 | C(CS)C(=O)OCC(COC(=O)CCS)(COC(=O)CCS)COC(=O)CCS | 0 | train |
| 144211829 | CC(C)OC(=O)C1=CC=CC=C1C(=O)OC(C)C | 1 | validation |
| 144211828 | CN(C)CCOCCN(C)CCO | 0 | test |
| 144211827 | C(CN)CN(CCO)CCO | 0 | train |
| 144211826 | C(CN)COCCOCCOCCCN | 0 | train |
| 144211825 | CN(C1CCCCC1)C2CCCCC2 | 0 | validation |
| 144211824 | CCCCCCCCCCCCCC(=O)OCC(CO)O | 0 | test |
| 144211823 | C1=CC(=CC(=C1)S(=O)(=O)[O-])S(=O)(=O)[O-].[Na+].[Na+] | 0 | train |
| 144211822 | C1=CC2=C(C=CC(=C2)S(=O)(=O)O)C=C1N | 0 | train |
| 144211821 | CCC[N+](CCC)(CCC)CCC.[Br-] | 0 | validation |
| 144211820 | C1=CC=C2C(=C1)C3=CC=CC=C3[P+](=O)O2 | 0 | test |
| 144211819 | C1=CC=C2C(=C1)N=C(S2)[S-].[Na+] | 0 | train |
| 144211818 | CCCOC(=O)C1=C(C(=C(C(=C1Cl)Cl)Cl)Cl)C(=O)OCCC | 1 | train |
| 144211817 | CC1CC(CC(C1)(C)C)O | 0 | validation |
| 144211816 | CCCSSCCC | 0 | test |
| 144211815 | CCN(CC)C(=O)N(CC)CC | 0 | train |
| 144211814 | C/C/1=C¥CCC(=C)[C@H]2CC([C@@H]2CC1)(C)C | 0 | train |
| 144211813 | CC(C)OC(=O)C1=CC=CC=C1 | 0 | validation |
| 144211812 | CC(C)C[C@@H](C(=O)O)NC(=O)OCC1C2=CC=CC=C2C3=CC=CC=C13 | 1 | test |
| 144211811 | C1=CC=C(C=C1)P(=O)(Cl)Cl | 0 | train |
| 144211810 | CCOP(=S)(OCC)S | 0 | train |
| 144211809 | Cl[Zn]Cl | 0 | validation |
| 144211808 | CCN(CCC#N)C1=CC=CC=C1 | 1 | test |
| 144211807 | COC(=O)/C=C¥C(=O)O | 0 | train |
| 144211806 | CCCCCCC(C)NC1=CC=C(C=C1)NC2=CC=CC=C2 | 1 | train |
| 144211805 | CCOC(=O)COC(=O)C1=CC=CC=C1C(=O)OCC | 0 | validation |
| 144211804 | C1C(=NN(C1=O)C2=CC=C(C=C2)S(=O)(=O)O)C(=O)[O-].[Na+] | 0 | test |
| 144211803 | CC(=O)C(=O)[O-].[Na+] | 0 | train |
| 144211802 | CCC1=NC=C(N1)C | 0 | train |
| 144211801 | CCOC1=CC(=C(C=C1)OCC)Cl | 0 | validation |
| 144211800 | CCOC(=O)CC(CC(=O)OCC)(C(=O)OCC)OC(=O)C | 1 | test |
| 144211799 | C(COCCS)OCCS | 0 | train |
| 144211798 | CC(=O)CC(=O)NC1=CC=C(C=C1)OC | 0 | train |
| 144211797 | COCC1=CC=C(C=C1)COC | 0 | validation |
| 144211796 | CCOC1=CC=C(C=C1)N=NC2=CC(=C(C=C2)C=CC3=C(C=C(C=C3)N=NC4=CC=C(C=C4)OC C)S(=O)(=O)[O-])S(=O)(=O)[O-].[Na+].[Na+] | 0 | test |
| 144211795 | C1=CC=C(C=C1)C2=C(C=C(C=C2)O)Cl | 1 | train |
| 144211794 | C1=CC(=CC=C1C#N)Cl | 0 | train |
| 144211793 | CCCCC(CC)COC(=O)C1=CC=CC=C1 | 0 | validation |
| 144211792 | C1=CC=C2C(=C1)C(=O)C3=C(C2=O)C=C(C=C3)S(=O)(=O)[O-].[Na+] | 0 | test |
| 144211791 | C(COCCOCCC#N)C#N | 0 | train |
| 144211790 | C1CC(CCC1CC2CCC(CC2)N=C=O)N=C=O | 0 | train |
| 144211789 | COCCOCCOCCOCCOCCO | 0 | validation |
| 144211788 | CCC1COC(=O)O1 | 0 | test |
| 144211787 | C=CCOC(=O)/C=C¥C(=O)OCC=C | 0 | train |
| 144211786 | CCOC(=O)CC1=CC=CC2=CC=CC=C21 | 1 | train |
| 144211785 | CCCCC(CC)C(C#C)O | 0 | validation |
| 144211784 | C1CCC(CC1)N2CCCC2=O | 0 | test |
| 144211783 | C=CS(=O)(=O)[O-].[Na+] | 0 | train |
| 144211782 | CCC(=O)/C=C/C1C(=CCCC1(C)C)C | 1 | train |
| 144211781 | C1=CC=C2C=C(C(=CC2=C1)C(=O)NC3=CC(=CC=C3)[N+](=O)[O-])O | 1 | validation |
| 144211780 | C1=CC(=C(C=C1[N+](=O)[O-])[N+](=O)[O-])SC#N | 1 | test |
| 144211779 | C1CCC(CC1)(C(=O)C2=CC=CC=C2)O | 0 | train |
| 144211778 | C1=CC=C(C(=C1)[N+](=O)[O-])[O-].[Na+] | 0 | train |

| | | | |
|---|---|---|---|
| 144211777 | C1=CC(=CC(=C1)C(=O)NN)C(=O)NN | 0 | validation |
| 144211776 | CC(=O)CCC1=CC=C(C=C1)O | 0 | test |
| 144211775 | CC(C1=CC=C(C=C1)Cl)(C2=CC=C(C=C2)Cl)O | 1 | train |
| 144211774 | C1=CC=C2C(=C1)C(=NC2=N)N | 1 | train |
| 144211773 | CC[C@H](C1=CC=C(C=C1)O)[C@@H](CC)C2=CC=C(C=C2)O | 1 | validation |
| 144211772 | C[C@H]1C[C@H]2[C@@H]3CCC4=CC(=O)C=C[C@@]4([C@]3([C@H](C[C@@]2([C@]1(C(=O)CO)O)O)C)O)F)C | 0 | test |
| 144211771 | CC(C)(C)C(=O)C(=O)O | 0 | train |
| 144211770 | CC(=O)O | 0 | train |
| 144211769 | CCOC1=CC(=C(C=C1N2CCOCC2)OCC)[N+](=O)[O-] | 1 | validation |
| 144211768 | C=CCOCC(CO)(COCC=C)COCC=C | 1 | test |
| 144211767 | CCN(CC)C1=CC2=C(C=C1)C(=C3C=CC(=[N+](CC)CC)C=C3O2)C4=CC=CC=C4C(=O)O.[Cl-] | 1 | train |
| 144211766 | C1=CC=C(C=C1)NC(=O)N | 0 | train |
| 144211765 | C1=CC(=C(C(=C1)F)C(=O)NC(=O)NC2=CC(=C(C(=C2F)Cl)F)Cl)F | 0 | validation |
| 144211764 | CCN(CC)C1=NC(=CC(=N1)OP(=S)(OCC)OCC)C | 1 | test |
| 144211763 | CC1=C(C=CC(=C1)OCCOCC[N+](C)(C)CC2=CC=CC=C2)C(C)(C)CC(C)(C)C.[Cl-] | 1 | train |
| 144211762 | CCCO[Si](OCCC)(OCCC)OCCC | 0 | train |
| 144211761 | CCOC(=O)C(C1=CC=CC=C1)Br | 0 | validation |
| 144211760 | C1=CC(=CC=C1N)[As](=O)(O)O | 0 | test |
| 144211759 | CC1=CC(=C(C=C1)NC=O)C | 0 | train |
| 144211758 | CC(CO)O | 0 | train |
| 144211757 | C[C@]12CCC(=O)C=C1CC[C@@H]3[C@@H]2C(=O)C[C@]4([C@H]3CC[C@@]4(C(=O)CO)O)C | 0 | validation |
| 144211756 | CC(=O)NC1=C(C(=C(C(=C1I)C(=O)[O-])I)NC(=O)C)I.[Na+] | 0 | test |
| 144211755 | COC(=O)CCCC(=O)O | 0 | train |
| 144211754 | CCOC(=O)NNC1=NN=CC2=CC=CC=C21.Cl | 0 | train |
| 144211753 | CC1=C(C(=C(C=C1/C(=C¥2/C=C(C(=O)C(=C2C)Br)Br)/C3=CC=CC=C3S(=O)(=O)[O-])Br)O)Br.[Na+] | 1 | validation |
| 144211752 | COC1=CC(=C(C(=C1)OC)C(=O)CCCN2CCCC2)OC.Cl | 0 | test |
| 144211751 | CC1(C(=O)C(C1=O)(C)C)C | 0 | train |
| 144211750 | CCCCCCCCCCCC[N+](C)(C)CC(=O)[O-] | 0 | train |
| 144211749 | C1=CC(=CC=C1C(C2=CC=C(C=C2)Cl)C(=O)O)Cl | 0 | validation |
| 144211748 | CC1=CC(=O)[C@H]2C[C@@H]1C2(C)C | 0 | test |
| 144211747 | C1=CC=NC(=C1)C2=CC=CC=N2 | 0 | train |
| 144211746 | C1[C@@H]2C=C[C@@H]1[C@H]3[C@@H]2[C@]4(C(=C([C@@]3(C4(Cl)Cl)Cl)Cl)Cl)Cl | 1 | train |
| 144211745 | COC(=O)C1(C2=CC=CC=C2C3=CC=CC=C31)O | 1 | validation |
| 144211744 | CCCCC1=C(N=C(N=C1OS(=O)(=O)N(C)C)NCC)C | 1 | test |
| 144211743 | CCOP(=S)(OCC)SCSC1=CC=C(C=C1)Cl | 1 | train |
| 144211742 | CC1CCCCC1NC(=O)NC2=CC=CC=C2 | 1 | train |
| 144211741 | CC1=CC(=CC(=C1)OC(=O)NC)C(C)C | 0 | validation |
| 144211740 | CC1=C(C=CC(=C1)OC(=O)NC)N(C)C | 1 | test |
| 144211739 | COC(=O)COC1=C(C=C(C=C1)Cl)Cl | 0 | train |
| 144211738 | CCOP(=S)(OCC)SCN1C(=O)C2=CC=CC=C2N=N1 | 1 | train |
| 144211737 | C(C(=O)[O-])F.[Na+] | 0 | validation |
| 144211736 | CCC(C)NC1=NC(=NC(=N1)NCC)OC | 0 | test |
| 144211735 | CC¥1=C(C2=C(/C1=C¥C3=CC=C(C=C3)S(=O)C)C=CC(=C2)F)CC(=O)O | 1 | train |
| 144211734 | CN(C)CCCN1C2=CC=CC=C2CCC3=CC=CC=C31.Cl | 0 | train |
| 144211733 | CC1([C@@H](N2[C@H](S1)[C@@H](C2=O)NC(=O)[C@@H](C3=CC=CC=C3)N)C(=O)O)C | 0 | validation |
| 144211732 | CC1=N[C@@]2([C@H](O1)C[C@@H]3[C@@]2(C[C@@H]([C@H]4[C@H]3CCC5=CC(=O)C=C[C@]45C)O)C)C(=O)COC(=O)C | 0 | test |
| 144211731 | CCC(C)(C#N)N=NC(C)(CC)C#N | 0 | train |
| 144211730 | C1=C2C=C(C=C(C2=C(C=C1S(=O)(=O)[O-])N)O)S(=O)(=O)O.O.[Na+] | 0 | train |
| 144211729 | CC1=C(C=CC(=C1)Cl)N=CN(C)C.Cl | 0 | validation |
| 144211728 | COP(=O)(O)OC | 0 | test |
| 144211727 | CC(C)CC(C)N(C1=CC=CC=C1)C2=CC=C(C=C2)N | 1 | train |
| 144211726 | C[C@@H](CCCC(C)(C)OC)C/C=C/C(=C/C(=O)OC(C)C)/C | 0 | train |
| 144211725 | C1=CC2=C(C=C1Cl)OC(=O)N2 | 0 | validation |
| 144211724 | C1CN(CCN1CCCN)CCCN | 0 | test |
| 144211723 | C1COC(C(O1)O)O | 0 | train |
| 144211722 | CC[Sn](CC)(CC)Br | 1 | train |
| 144211721 | CC(=O)OC1CCCCC1C(C)(C)C | 0 | validation |

| | | | |
|---|---|---|---|
| 144211720 | CC(C)COCNC(=O)C=C | 0 | test |
| 144211719 | CC(=O)O.CC(=O)O.CC(=O)O.CC(=O)O.CC(=O)O.CC(=O)O.CC(=O)O.O.O.[Cr].[Cr].[Cr] | 0 | train |
| 144211718 | CC(C)(C)C1=CC(=CC(=C1O)C(C)(C)C)CN(C)C | 1 | train |
| 144211717 | CC1CC(CC(C1)(C)C)OC(=O)C(=C)C | 0 | validation |
| 144211716 | CCC1(COCOC1)CO | 0 | test |
| 144211715 | CC(C)(C1CCC(CC1)O)C2CCC(CC2)O | 1 | train |
| 144211714 | CCO[Si](CC(C)C)(OCC)OCC | 0 | train |
| 144211713 | CCC(C)(CCC(C)C)C(=O)OC=C | 0 | validation |
| 144211712 | C(COCCN)N | 0 | test |
| 144211711 | C(C(=O)[O-])(Cl)Cl.[Na+] | 0 | train |
| 144211710 | CCCCCCOC(=O)C | 0 | train |
| 144211709 | C12C(NC(=O)N1)NC(=O)N2 | 0 | validation |
| 144211708 | CNC(=S)NC | 0 | test |
| 144211707 | C1=CC=C2C=C(C=CC2=C1)OCC(=O)O | 0 | train |
| 144211706 | CCCCOC(=O)C(=O)OCCCC | 0 | train |
| 144211705 | COC(=O)N | 0 | validation |
| 144211704 | C1=CC=C(C=C1)C2=CC=CC=C2[O-].O.O.O.O.[Na+] | 1 | test |
| 144211703 | C1C(O1)CN(CC2CO2)C3=CC=C(C=C3)CC4=CC=C(C=C4)N(CC5CO5)CC6CO6 | 0 | train |
| 144211702 | CCCCCCCCCC(=O)C | 0 | train |
| 144211701 | COC(=O)/C=C/C1=CC=CC=C1 | 1 | validation |
| 144211700 | CCOC1=CC=CC=C1O | 0 | test |
| 144211699 | CC(=CCCC(=CCCC(C)(C=C)O)C)C | 1 | train |
| 144211698 | CCCCCCOC1=CC=CC=C1C(=O)O | 0 | train |
| 144211697 | CC1=CC=C(C=C1)S(=O)(=O)[O-].[Na+] | 0 | validation |
| 144211696 | [Na+].[I-] | 0 | test |
| 144211695 | CC1=C(C=C(C=C1)C(C)C)O | 0 | train |
| 144211694 | CCCCCCCOC(=O)CCC | 0 | train |
| 144211693 | CCC1=CC=CC=C1N | 0 | validation |
| 144211692 | CC1=CC[C@H](CC1)[C@](C)(CCC=C(C)C)O | 0 | test |
| 144211691 | CCO[Si](CC)(OCC)OCC | 0 | train |
| 144211690 | CC(C)N(C1=CC=CC=C1)C(C)C | 0 | train |
| 144211689 | CC(C)C1=CC(=C(C=C1)O)C(C)C | 0 | validation |
| 144211688 | CC(O)(P(=O)(O)O)P(=O)(O)O.O | 1 | test |
| 144211687 | C1(=C(SC(=C1Cl)Cl)Cl)Cl | 0 | train |
| 144211686 | CCCCCCCCCCCC[N+](C)(C)CC1=CC=CC=C1.[Cl-] | 1 | train |
| 144211685 | C1CC(=O)N(C1=O)Cl | 0 | validation |
| 144211684 | CC1=CC(=O)C2=CC=CC=C2C1=O | 1 | test |
| 144211683 | Cl[Pd]Cl | 0 | train |
| 144211682 | C1=CC=C2C(=C1)C(=CN2)CC(=O)C(=O)O | 1 | train |
| 144211681 | CC(C)(C)C(=O)CC(=O)OC | 0 | validation |
| 144211680 | CC1=CC(=CC=C1)N(C)C(=S)OC2=CC3=CC=CC=C3C=C2 | 1 | test |
| 144211679 | C1=CC=C(C=C1)CC(=O)C(=O)O | 0 | train |
| 144211678 | CC(C)C1=CC=CC=C1OC(=O)NC | 1 | train |
| 144211677 | CN(C(=O)NC1=CC=C(C=C1)Cl)OC | 1 | validation |
| 144211676 | CCOC(=O)C1=CN2C(=CC(=N2)OP(=S)(OCC)OCC)N=C1C | 1 | test |
| 144211675 | CCCSP(=S)(OCC)OC1=CC=C(C=C1)SC | 1 | train |
| 144211674 | CCN(CC)C(=O)SCC | 0 | train |
| 144211673 | CCSCC1=CC=CC=C1OC(=O)NC | 1 | validation |
| 144211672 | CCOP(=O)(SC1=CC=CC=C1)SC2=CC=CC=C2 | 1 | test |
| 144211671 | C1=CC(=CC=C1[N+](=O)[O-])OC2=C(C=C(C=C2)C(F)(F)F)[N+](=O)[O-] | 1 | train |
| 144211670 | CCCCNC(=O)NCCCC | 0 | train |
| 144211669 | CCCCCCCCCCCC(=O)N(C)CC(=O)O | 0 | validation |
| 144211668 | C1=CC=C(C(=C1)C(=O)O)C(=O)[O-].[K+] | 0 | test |
| 144211667 | CCN(CCO)CCO | 0 | train |
| 144211666 | CCCCOC(=O)C(C)O | 0 | train |
| 144211665 | C[C@@]12CC[C@@H](C1)C([C@@H]2O)(C)C | 0 | validation |
| 144211664 | CC1=CC(=C(C(=C1)Cl)OP(=S)(OC)OC)Cl | 1 | test |
| 144211663 | C1=CC=C2C(=C1)C=CC=C2CC(=O)[O-].[K+] | 0 | train |
| 144211662 | CC(COC(C)(C)C)OCCCO | 0 | train |
| 144211661 | CCC(=O)O[C@@H]1C[C@H]2CC[C@@]1(C2(C)C)C | 0 | validation |
| 144211660 | [Na+].[Br-] | 0 | test |
| 144211659 | CCCCOCCOCCOCCOCCO | 0 | train |

| | | | |
|---|---|---|---|
| 144211658 | CCCCCCCCCCCC(=O)NCCCN(C)C | 1 | train |
| 144211657 | CCOC1=CC=C(C=C1)NC(=O)N | 0 | validation |
| 144211656 | CCCC1C(=CC(=CN1C2=CC=CC=C2)CC)CC | 1 | test |
| 144211655 | C1=CC=C(C(=C1)C(CN2C=NC=N2)(C3=CC=C(C=C3)F)O)F | 0 | train |
| 144211654 | CC(C)(C)C1=CC=C(C=C1)C(=O)O | 0 | train |
| 144211653 | CN(C)C1=CC=C(C=C1)N=NC2=CC=CC=C2C(=O)O | 1 | validation |
| 144211652 | COC(=O)NC1=CC(=C(C=C1)Cl)Cl | 1 | test |
| 144211651 | COP(=S)(OC)OC1=CC(=C(C=C1Cl)Cl)Cl | 1 | train |
| 144211650 | CCCN(CCCl)C1=C(C=C(C=C1[N+](=O)[O-])C(F)(F)F)[N+](=O)[O-] | 1 | train |
| 144211649 | CC1=C(OC(=C1C(=O)NC2=CC=CC=C2)C)C | 1 | validation |
| 144211648 | CC1=C(S(=O)(=O)CCO1)C(=O)NC2=CC=CC=C2 | 0 | test |
| 144211647 | C1=C(C=C(C(=C1I)O)I)C#N | 0 | train |
| 144211646 | CC(C)C1=CC=C(C=C1)NC(=O)N(C)C | 1 | train |
| 144211645 | CC(C)OC(=O)C1=CC=CC=C1OP(=S)(N)OC | 1 | validation |
| 144211644 | CCCCCCCCOS(=O)(=O)[O-].[Na+] | 0 | test |
| 144211643 | C1=CC=C(C=C1)[Hg]C2=CC=CC=C2 | 1 | train |
| 144211642 | CC(C)OC(=S)[S-].[Na+] | 0 | train |
| 144211641 | C1=CC=C(C=C1)S(=O)(=O)OC2=CC=C(C=C2)Cl | 1 | validation |
| 144211640 | CC(C)C1=CC=C(C=C1)O | 0 | test |
| 144211639 | N(=O)[O-].[K+] | 0 | train |
| 144211638 | CCC(C)N | 0 | train |
| 144211637 | CC1=C(C=C(C=C1C(=O)N)[N+](=O)[O-])[N+](=O)[O-] | 0 | validation |
| 144211636 | C1=CC=C(C=C1)[As]=O | 1 | test |
| 144211635 | CCCC(CN1C=NC=N1)C2=C(C=C(C=C2)Cl)Cl | 1 | train |
| 144211634 | CCCCOC(=O)[C@H](C)O | 0 | train |
| 144211633 | COS(=O)(=O)[O-].[Na+] | 0 | validation |
| 144211632 | CC1=CC=CC=C1N=C(N)N=C(N)N | 0 | test |
| 144211631 | CCOC(OCC)OCC | 0 | train |
| 144211630 | C(=O)(C(Cl)(Cl)Cl)[O-].[Na+] | 0 | train |
| 144211629 | C1COCCN1CCOCCN2CCOCC2 | 0 | validation |
| 144211628 | CO[Si](C1=CC=CC=C1)(OC)OC | 0 | test |
| 144211627 | CCO[Si](C)(OCC)OCC | 0 | train |
| 144211626 | CCOC(=O)C(F)(F)F | 0 | train |
| 144211625 | CC(=O)OC(C)(C)C | 0 | validation |
| 144211624 | C1=CC=C(C=C1)CNC2=NC=NC3=C2NC=N3 | 1 | test |
| 144211623 | CC(CN(CCCN(C)C)CCCN(C)C)O | 0 | train |
| 144211622 | CCCCOC(CC)O | 0 | train |
| 144211621 | CCCCOC(CC)OC(C)COC(CC)O | 0 | validation |
| 144211620 | [Li+].C1=C(C=C(C=C1C(=O)O)S(=O)(=O)O)C(=O)[O-] | 0 | test |
| 144211619 | CCCCOCCOC(=O)CCCCCCCCC(=O)OCCOCCCC | 0 | train |
| 144211618 | C1=CC=C(C=C1)NC(=O)C2=CC=CC=C2O | 1 | train |
| 144211617 | C1=CC=C2C(=C1)C(=CN2)C[C@H](C(=O)O)N | 0 | validation |
| 144211616 | CC1OC(OC(O1)C)C | 0 | test |
| 144211615 | C(C(=O)O)O | 0 | train |
| 144211614 | C(C(=O)N)F | 0 | train |
| 144211613 | CCCCCCCCCCCCCCCC[N+]1=CC=CC=C1.[Cl-] | 0 | validation |
| 144211612 | CC(=S)N | 0 | test |
| 144211611 | II | 0 | train |
| 144211610 | [K+].[I-] | 0 | train |
| 144211609 | CC(C)CCCO | 0 | validation |
| 144211606 | CC(C)(C)N | 0 | train |
| 144211605 | O=[Si]=O | 0 | validation |
| 144211604 | C1=CC=C2C(=C1)C(=CN2)CCCC(=O)O | 0 | test |
| 144211603 | C1=CC2=C(C3=C(C=CC=N3)C=C2)N=C1.O.Cl | 1 | train |
| 144211602 | CCCCC(CC)COC(=O)OOC(C)(C)C | 0 | train |
| 144211601 | CO[Si](CCCN1C(=O)N(C(=O)N(C1=O)CCC[Si](OC)(OC)OC)CCC[Si](OC)(OC)OC)(OC)OC | 0 | validation |
| 144211600 | CC(=O)O[Si](C=C)(OC(=O)C)OC(=O)C | 0 | test |
| 144211599 | CC(COC(C)COC(=O)C=C)OCC(C)OC(=O)C=C | 0 | train |
| 144211598 | CC1=NN(C(=O)C1)C2=CC=C(C=C2)S(=O)(=O)O | 0 | train |
| 144211597 | CC(C)(C)C1CCCCC1O | 0 | validation |
| 144211596 | C1=CC=C(C=C1)COC(=O)C(=O)OCC2=CC=CC=C2 | 0 | test |
| 144211595 | C1(=NC(=NC(=N1)[S-])[S-])[S-].[Na+].[Na+].[Na+] | 0 | train |

| | | | |
|---|---|---|---|
| 144211594 | CC(=C)C(=O)OCCOP(=O)(O)O | 0 | train |
| 144211593 | CC(C)CCCCCOC(=O)CCS | 0 | validation |
| 144211592 | CC1=C(C=CC(=C1)C(=C2C=CC(=NC3=C(C=C(C=C3)S(=O)(=O)[O-])S(=O)(=O)O)C=C2)C4=CC=C(C=C4)NC5=CC=C(C=C5)S(=O)(=O)[O-])N.[Na+].[Na+] | 0 | test |
| 144211591 | CCCCCCC(CCCC)CO | 0 | train |
| 144211590 | CC(=CCC/C(=C¥C#N)/C)C | 0 | train |
| 144211589 | CCN1C2=C(C=C(C=C2)[N+](=O)[O-])C3=CC=CC=C31 | 1 | validation |
| 144211588 | C=CC(=O)OCC1CCC2C1C3CC(C2C3)COC(=O)C=C | 0 | test |
| 144211587 | CCCCOC(=O)CCCCCCCC(=O)OCCCC | 0 | train |
| 144211586 | CC1(CC(=O)CC(N1[O])(C)C)C | 0 | train |
| 144211585 | C=CC1=CC(=CC=C1)CCl | 0 | validation |
| 144211584 | CC(C)OP(=O)(CP(=O)(OC(C)C)OC(C)C)OC(C)C | 0 | test |
| 144211583 | COC(=O)C1=C(C=CC(=C1)Cl)[N+](=O)[O-] | 0 | train |
| 144211582 | CCCCCCCCCCCCSC | 0 | train |
| 144211581 | C=COCCOCCOC=C | 0 | validation |
| 144211580 | CCCCN(CCCCO)N=O | 0 | test |
| 144211579 | CCC(CO)CO | 0 | train |
| 144211578 | CC(C)CC(=O)C1C(=O)C2=CC=CC=C2C1=O | 1 | train |
| 144211577 | CC(CCC1=CC=CC=C1)CCO | 0 | validation |
| 144211576 | C1=CC(=CN=C1)C(C2=CC=C(C=C2)Cl)(C3=CC=C(C=C3)Cl)O | 0 | test |
| 144211575 | C1CCC(CC1)N(C2CCCCC2)SC3=NC4=CC=CC=C4S3 | 1 | train |
| 144211574 | CN1C(C(N(C1=O)C)O)O | 0 | train |
| 144211573 | CC1CC(C2=C(C1(C)C)C=C(C(=C2)C(=O)C)C)(C)C | 1 | validation |
| 144211572 | C1CCC(C1)N(CC2=CC=C(C=C2)Cl)C(=O)NC3=CC=CC=C3 | 1 | test |
| 144211571 | CCOC(=O)COC(=O)C1=C(C=CC(=C1)OC2=C(C=C(C=C2)C(F)(F)F)Cl)[N+](=O)[O-] | 1 | train |
| 144211570 | CCOC(=O)C1=NOC(C1)(C2=CC=CC=C2)C3=CC=CC=C3 | 0 | train |
| 144211569 | C1CC(C2=CC=CC=C2C1)C3=C(C4=CC=CC=C4OC3=O)O | 0 | validation |
| 144211568 | C1=CC=C(C(=C1)C(C2=CC=C(C=C2)F)(C3=CN=CN=C3)O)Cl | 0 | test |
| 144211567 | C1(=C(C(=NC(=C1Cl)Cl)C(Cl)(Cl)Cl)Cl)Cl | 0 | train |
| 144211566 | CC(C)(C)C1=CC=C(C=C1)CCC=O | 0 | train |
| 144211565 | CC(C)(C)OC(=O)C1=CC=C(C=C1)O | 1 | validation |
| 144211564 | CCCCCCCCSC(=O)OC1=CC(=NN=C1C2=CC=CC=C2)Cl | 0 | test |
| 144211563 | C1=CC(=NC=C1C(Cl)(Cl)Cl)Cl | 0 | train |
| 144211562 | CCCCCCOC1=C(C(=C(C(=C1)C)O)C)C | 1 | train |
| 144211561 | CC(C)COP(=O)(OCC(C)C)OCC(C)C | 0 | validation |
| 144211560 | CO[Si](C)(C1=CC=CC=C1)OC | 0 | test |
| 144211559 | C(#N)C1=C(C(=C(C(=N1)Cl)Cl)Cl)Cl | 1 | train |
| 144211558 | CC1COC2=CC=CC=C2N1 | 1 | train |
| 144211557 | C1=C(N=CS1)C#N | 0 | validation |
| 144211556 | CCCCCCCCCCCCCC[N+]1=CC=CC=C1.[Br-] | 0 | test |
| 144211555 | CC(C)(C)C1=CC(=CC(=C1O)C(C)(C)C)CCC(=O)NN | 1 | train |
| 144211554 | CC1(C(=O)NC(=O)NC1=O)C | 0 | train |
| 144211553 | CCCCCCCCCCCCN1CCOCC1 | 0 | validation |
| 144211552 | CC(=O)CN(CC(=O)C)N=O | 0 | test |
| 144211551 | CCCCCCCCCCCCCCN(CCO)CCO | 1 | train |
| 144211550 | CC1=CC(=C(C(=C1)C)C2=C(C3(CCCC3)OC2=O)OC(=O)CC(C)(C)C)C | 0 | train |
| 144211549 | CCCCC(CC)C(=O)OC(C)C | 0 | validation |
| 144211548 | CC(C)CCCC(C)CC=O | 0 | test |
| 144211547 | C1=CC=C2C(=C1)C=C(C(=N2)C(=O)O)C(=O)O | 0 | train |
| 144211546 | CCOC1=CC2=C(C=C1)NC(CC2C)(C)C | 1 | train |
| 144211545 | C1CCC(C1)C2=C(C=CC(=C2)Cl)O | 1 | validation |
| 144211544 | CCCOCCOCCOCCO | 0 | test |
| 144211543 | CCCC(CC)C1=CC=CC=C1 | 0 | train |
| 144211542 | CCOC(=O)CC(C(=O)C)C(=O)C | 0 | train |
| 144211541 | CC1=CC=C(C=C1)N(SC(F)(Cl)Cl)S(=O)(=O)N(C)C | 1 | validation |
| 144211540 | CC(C)(C)C1=CC=C(C=C1)CCOC2=NC=NC3=CC=CC=C32 | 0 | test |
| 144211539 | C1=CC(=CC(=C1)C(F)(F)F)C(=O)CC2=CC=C(C=C2)C#N | 1 | train |
| 144211538 | C1=CC(=CN=C1)C(=O)[O-].[Na+] | 0 | train |
| 144211537 | C1=C2C(=NC(=O)NC2=O)NN1 | 0 | validation |
| 144211536 | CCCSP(=S)(OCC)OC1=C(C=C(C=C1)Cl)Cl | 1 | test |
| 144211535 | CCCCCCCCC(=O)NCC1=CC(=C(C=C1)O)OC | 0 | train |
| 144211534 | CCCCCC(=O)OCC | 0 | train |

| | | | |
|---|---|---|---|
| 144211533 | C1=CC(=C(C=C1O)O)C2=C(C(=O)C3=C(C=C(C=C3O2)O)O)O.O.O | 0 | validation |
| 144211532 | C1C=CC2=CC=CC=C21 | 1 | test |
| 144211531 | C1=COC(=C1)CNC2=NC=NC3=C2NC=N3 | 0 | train |
| 144211530 | CCCOC(=O)NCCCN(C)C | 0 | train |
| 144211529 | CN1CCCC1 | 0 | validation |
| 144211528 | CCOC(=S)[S-].[K+] | 0 | test |
| 144211527 | C1=CC=C(C=C1)O[P+](=O)OC2=CC=CC=C2 | 0 | train |
| 144211526 | CCOC(=O)NCCOC1=CC=C(C=C1)OC2=CC=CC=C2 | 1 | train |
| 144211525 | CCCCCC(=O)CC | 0 | validation |
| 144211524 | CC(C)OC1=CC=CC=C1OC(=O)NC | 0 | test |
| 144211523 | CC1=CC(=C(C=C1)OP(=S)(NC(C)C)OC)[N+](=O)[O-] | 1 | train |
| 144211522 | C=CC(=O)OCCC#N | 0 | train |
| 144211521 | C(CO)NN | 0 | validation |
| 144211520 | COC1=C(C=CC(=C1)CC=C)OC(=O)CC2=CC=CC=C2 | 0 | test |
| 144211519 | C1=C(C=C(C(=C1Br)O)Br)C#N | 0 | train |
| 144211518 | CC1=CC(=C(C(=C1Cl)C)Cl)O | 0 | train |
| 144211517 | CC(C)CC(=NO)C | 0 | validation |
| 144211516 | C1=CC=C(C=C1)CNCCO | 0 | test |
| 144211515 | CCC(C)(CCC=C(C)C)O | 0 | train |
| 144211514 | CC(=O)NC1=CC=C(C=C1)CC(=O)O | 0 | train |
| 144211513 | C(C#N)C#N | 1 | validation |
| 144211512 | CC(CCC=C(C)C)CC=O | 0 | test |
| 144211511 | CCOC(=O)CC(C)C | 0 | train |
| 144211510 | CCCCOC(=O)[C@@H](C)OC1=CC=C(C=C1)OC2=NC=C(C=C2)C(F)(F)F | 1 | train |
| 144211509 | CC1=CC(=NN1CO)C | 0 | validation |
| 144211508 | CN=C(NCC1=CN=C(S1)Cl)N[N+](=O)[O-] | 0 | test |
| 144211507 | C1=CC=C(C=C1)S(=O)(=O)C2=CC=CC=C2 | 1 | train |
| 144211506 | CC(C)OC1=CC=CC=C1O | 0 | train |
| 144211505 | C1CC=COC1 | 0 | validation |
| 144211504 | CCCCC(CC)(CO)CO | 0 | test |
| 144211503 | CCCCOP(=O)(O)OCCCC | 0 | train |
| 144211502 | CC(C1CC1)C(CN2C=NC=N2)(C3=CC=C(C=C3)Cl)O | 0 | train |
| 144211501 | COC1=CC(=NC(=N1)NC(=O)[N-]S(=O)(=O)C2=C(C=CC=N2)OCC(F)(F)F)OC.[Na+] | 0 | validation |
| 144211500 | CC(=C)C(=O)OCCOC(=O)CC(=O)C | 0 | test |
| 144211499 | CC12CCCC=C1C(=O)OC2=O | 0 | train |
| 144211498 | C1=CC=C(C(=C1)NC2=NC(=NC(=N2)Cl)Cl)Cl | 0 | train |
| 144211497 | CCCCCCCCCC(=O)OCC | 0 | validation |
| 144211496 | CCCN(CCOC1=C(C=C(C=C1Cl)Cl)Cl)C(=O)N2C=CN=C2 | 1 | test |
| 144211495 | C1C=CCC2C1C(=O)NC2=O | 0 | train |
| 144211494 | C1=C(C=NC(=C1Cl)NC2=C(C=C(C(=C2[N+](=O)[O-])Cl)C(F)(F)F)[N+](=O)[O-])C(F)(F)F | 1 | train |
| 144211493 | CC(C#C)N(C)C(=O)NC1=CC=C(C=C1)Cl | 1 | validation |
| 144211492 | C1CC(CC(C1)CN)CN | 0 | test |
| 144211491 | CC(CCCC(C)(C)O)CC=O | 0 | train |
| 144211490 | CC1=CCCC(C1/C=C/C(=O)C)(C)C | 0 | train |
| 144211489 | CCOP(=O)(OCC)OP(=O)(OCC)OCC | 0 | validation |
| 144211488 | CCNCCO | 0 | test |
| 144211487 | CCCCNC(=O)N1C2=CC=CC=C2N=C1NC(=O)OC | 1 | train |
| 144211486 | CCCC1=CC=C(C=C1)OC | 0 | train |
| 144211485 | CCCOC(=O)CCCCC(=O)OCCC | 0 | validation |
| 144211484 | CCOC(=O)CCCCCCCCC(=O)OCC | 0 | test |
| 144211483 | CCOC(=O)C(C)OC1=CC=C(C=C1)OC2=NC3=C(O2)C=C(C=C3)Cl | 1 | train |
| 144211482 | CC(=O)CCC1=CC=C(C=C1)OC | 0 | train |
| 144211481 | CC(C)(C#C)NC(=O)C1=CC(=CC(=C1)Cl)Cl | 0 | validation |
| 144211480 | CCCN(CC)CC1COC2(O1)CCC(CC2)C(C)(C)C | 0 | test |
| 144211479 | CC1([C@H]2CCC(=C)[C@@H]1C2)C | 0 | train |
| 144211478 | CNC(=O)CSP(=S)(OC)OC | 0 | train |
| 144211477 | CCOP(=S)(OCC)OC1=C(C=C(C=C1)Cl)Cl | 1 | validation |
| 144211476 | C[N+](C)(C)CC(=O)[O-] | 0 | test |
| 144211475 | COCCCOCCCOC | 0 | train |
| 144211474 | C1(=S)NNC(=S)S1 | 0 | train |
| 144211473 | CNC(=O)ON=C(C(=O)N(C)C)SC | 0 | validation |
| 144211472 | CCN(CC)C(=O)SCC1=CC=C(C=C1)Cl | 1 | test |

| | | | |
|---|---|---|---|
| 144211471 | CN1COCN(C1=N[N+](=O)[O-])CC2=CN=C(S2)Cl | 0 | train |
| 144211470 | C/C(=C¥C(=O)OC)/OP(=O)(OC)OC | 0 | train |
| 144211469 | C(C=O)Cl | 0 | validation |
| 144211468 | C1=CC=C2C(=C1)C(=O)C3=CC=CC=C3O2 | 1 | test |
| 144211467 | CC(C)CC1=C(C(=NC(=C1C(=O)SC)C(F)(F)F)C(F)F)C(=O)SC | 1 | train |
| 144211466 | CCOC(=O)C1=CC=C(C=C1)N | 1 | train |
| 144211465 | CC(C1=CC2=CC=CC=C2S1)N(C(=O)N)O | 1 | validation |
| 144211464 | C1=CC=C(C=C1)CC2=CC=CC=C2 | 0 | test |
| 144211463 | CC(C)(C(=O)OCC=C)OC(=O)C1=C(C=CC(=C1)N2C(=O)C=C(N(C2=O)C)C(F)(F)F)Cl | 1 | train |
| 144211462 | C1C[N+]2=CC=CC=C2C3=CC=CC=[N+]31.O.[Br-].[Br-] | 1 | train |
| 144211461 | CC1(C(=O)N(C(=O)O1)NC2=CC=CC=C2)C3=CC=C(C=C3)OC4=CC=CC=C4 | 1 | validation |
| 144211460 | CC(C(=O)O)OC1=C(C=C(C=C1)Cl)Cl | 0 | test |
| 144211459 | C1=CC(=C(C=C1[N+](=O)[O-])Cl)C(=O)N | 0 | train |
| 144211458 | CCC(C)OC(=O)N1CCCCC1CCO | 0 | train |
| 144211457 | CCCCCCC1CCOC1=O | 0 | validation |
| 144211456 | CC(C)OC(=O)NC1=CC(=CC=C1)Cl | 1 | test |
| 144211455 | CC(C)CC(C)(C#C)O | 0 | train |
| 144211454 | CC/C=C¥C#N | 0 | train |
| 144211453 | CC(C)OCCO | 0 | validation |
| 144211452 | CCCCC(CN1C=NC=N1)(C#N)C2=CC=C(C=C2)Cl | 0 | test |
| 144211451 | C(CCl)P(=O)(O)O | 0 | train |
| 144211450 | CCC(C)(C)C(=O)OC1=C(C(=O)OC12CCCCC2)C3=CC(=CC(=C3)Cl)Cl | 0 | train |
| 144211449 | CCCOC1=NN(C(=O)N1C)C(=O)[N-]S(=O)(=O)C2=CC=CC=C2C(=O)OC.[Na+] | 0 | validation |
| 144211448 | CCCCN | 0 | test |
| 144211447 | COC(=O)N(C1=CC=CC=C1COC2=NN(C=C2)C3=CC=C(C=C3)Cl)OC | 1 | train |
| 144211446 | C1=CC(=NC(=C1)C(=O)O)C(=O)O | 0 | train |
| 144211445 | C1=COC(=C1)C(=O)O | 0 | validation |
| 144211444 | CC1=CC=CC=C1CO[C@H]2C[C@@]3(CC[C@]2(O3)C)C(C)C | 0 | test |
| 144211443 | CC1=CC=C(CC1)C(C)C | 0 | train |
| 144211442 | CCOP(=O)(OCC)OC1=NC(=NC(=C1)C)C(C)C | 0 | train |
| 144211441 | C=CN1CCCCCC1=O | 0 | validation |
| 144211440 | C1CCCCCCCOC(=O)CCCCCC1 | 0 | test |
| 144211439 | CC(C)C(=O)OCCC1=CC=CC=C1 | 0 | train |
| 144211438 | CC1=CC=CC=C1OCC(=O)O | 0 | train |
| 144211437 | CCC(C)NC1=C(C=C(C=C1[N+](=O)[O-])C(C)(C)C)[N+](=O)[O-] | 1 | validation |
| 144211436 | CC(=O)C1=CC=CC=C1O | 0 | test |
| 144211435 | CC(=NOC(=O)NC)SC | 0 | train |
| 144211434 | CCCCNC(=O)OCC#CI | 1 | train |
| 144211433 | CC(=O)OCC1=CC=C(C=C1)OC | 0 | validation |
| 144211432 | COC1=CC=C(C=C1)CO | 0 | test |
| 144211431 | C1=C(C=C(C(=C1Cl)N2C(=C(C(=N2)C#N)S(=O)C(F)(F)F)N)Cl)C(F)(F)F | 0 | train |
| 144211430 | CCC(C(C(CC(C(C(C)Cl)Cl)Cl)Cl)Cl)Cl | 1 | train |
| 144211429 | CCOC1C(C2=C(O1)C=CC(=C2)OS(=O)(=O)C)(C)C | 1 | validation |
| 144211428 | CCC(C)N1C(=O)C(=C(NC1=O)C)Br | 0 | test |
| 144211427 | CC1COC(O1)(CN2C=NC=N2)C3=C(C=C(C=C3)OC4=CC=C(C=C4)Cl)Cl | 0 | train |
| 144211426 | CCC(=C1C(=O)CC(CC1=O)C2CCOCC2)NOC/C=C/Cl | 0 | train |
| 144211425 | CCCC(=O)OC/C=C(¥C)/CCC=C(C)C | 0 | validation |
| 144211424 | CCCOC(=O)CC | 0 | test |
| 144211423 | CCCCC(CC)C(=O)OCCOCCOCCOCCOC(=O)C(CC)CCCC | 0 | train |
| 144211422 | C1(=NC(=NC(=N1)Cl)N)N | 0 | train |
| 144211421 | CCCCCCCCCCCCCCCCCC(=O)OCC(CO)O | 0 | validation |
| 144211420 | CCOC(=O)CC(C(=O)OCC)SP(=O)(OC)OC | 0 | test |
| 144211419 | CN(C)C(=S)SSC(=S)N(C)C | 1 | train |
| 144211418 | CC1(C(=O)N(C(=O)O1)C2=CC(=CC(=C2)Cl)Cl)C=C | 0 | train |
| 144211417 | CC1=CC(=CC(=C1)C(=O)N(C(C)(C)C)NC(=O)C2=C(C(=CC=C2)OC)C)C | 0 | validation |
| 144211416 | CCOP(=S)(OCC)OC1=CC(=C(C=C1Cl)Br)Cl | 1 | test |
| 144211415 | CCCCCC[C@H](C/C=C¥CCCCCCCC(=O)O)O | 0 | train |
| 144211414 | C1=CC(=C(C=C1Cl)Cl)OCCCC(=O)[O-].[Na+] | 0 | train |
| 144211413 | C(C(=O)O)OC1=NC(=C(C(=C1Cl)N)Cl)F | 0 | validation |
| 144211412 | CCCCCCC1CCC(=O)O1 | 0 | test |
| 144211411 | C1=CC=C(C=C1)NC(=O)NC2=CC(=NC=C2)Cl | 1 | train |
| 144211410 | C[C@@]1(C(=O)N(C(=N1)SC)NC2=CC=CC=C2)C3=CC=CC=C3 | 1 | train |

| | | | |
|---|---|---|---|
| 144211409 | CC(C)(C)NC(=O)C=C | 0 | validation |
| 144211408 | CC1=NN(C(=O)N1C(F)F)C2=CC(=C(C=C2Cl)Cl)NS(=O)(=O)C | 0 | test |
| 144211407 | CC(C)(C)C1=CC=C(C=C1)CSC2=C(C(=O)N(N=C2)C(C)(C)C)Cl | 0 | train |
| 144211406 | C1=CC=C(C=C1)CNCC2=CC=CC=C2 | 0 | train |
| 144211405 | CC(C)N1C(=NC(C)(C)C)SCN(C1=O)C2=CC=CC=C2 | 1 | validation |
| 144211404 | CCNCC.Cl | 0 | test |
| 144211403 | CCC(C)S | 0 | train |
| 144211402 | CCCCOC(=O)C(C)OC1=CC=C(C=C1)OC2=NC=C(C=C2)C(F)(F)F | 0 | train |
| 144211401 | CC(C)C1=CC=C(C=C1)CC(C)C=O | 0 | validation |
| 144211400 | CC(C)C1=CC2=C(C=C1)C=C(C=C2)C(C)C | 1 | test |
| 144211399 | CCN(CC(=C)C)C1=C(C=C(C=C1[N+](=O)[O-])C(F)(F)F)[N+](=O)[O-] | 0 | train |
| 144211398 | CCC(=O)OCC | 0 | train |
| 144211397 | COCCO | 0 | validation |
| 144211396 | CC1=CC=CC=C1C(C2=CC=CC=C2)OCCN(C)C.C(C(=O)O)C(CC(=O)O)(C(=O)O)O | 0 | test |
| 144211395 | C/C(=C¥C(=O)N(C)C)/OP(=O)(OC)OC | 0 | train |
| 144211394 | C1=CC=C2C(=C1)C(=O)N(C2=O)SC(Cl)(Cl)Cl | 1 | train |
| 144211393 | C1CC1(C(=O)NC2=C(C=C(C=C2)Cl)Cl)C(=O)O | 1 | validation |
| 144211392 | C(C#N)NCC#N | 0 | test |
| 144211391 | CC(C)(C(=O)C1=CC=C(C=C1)OCCO)O | 0 | train |
| 144211390 | CC(C)N(C1=CC=C(C=C1)F)C(=O)COC2=NN=C(S2)C(F)(F)F | 1 | train |
| 144211389 | C1=CC=C2C(=C1)N=C(S2)SCSC#N | 1 | validation |
| 144211388 | CC(C)NC1=NC(=NC(=N1)N)Cl | 0 | test |
| 144211387 | C(CCBr)CBr | 0 | train |
| 144211386 | C[Si](O[Si](C)(C)C)O[Si](C)(C)C | 0 | train |
| 144211385 | CC1=CC(=O)OC2=C1C=CC(=C2)O.O | 0 | validation |
| 144211384 | C(CCC(=O)NN)CC(=O)NN | 0 | test |
| 144211383 | CCOC(=O)/C=C(¥C)/C=C/C[C@@H](C)CCCC(C)C | 0 | train |
| 144211382 | C1=CC=C2C(=C1)C(=O)N(N=N2)CCl | 1 | train |
| 144211381 | CC1=C(C=CC=C1COC(=O)C2C(C2(C)C)/C=C(/C(F)(F)F)¥Cl)C3=CC=CC=C3 | 0 | validation |
| 144211380 | CCOP(=O)(OCC)OC1=NC(=C(C=C1Cl)Cl)Cl | 0 | test |
| 144211379 | Cl[Yb](Cl)Cl | 0 | train |
| 144211378 | C#CCN1C(=O)COC2=CC(=C(C=C21)N3C(=O)C4=C(C3=O)CCCC4)F | 1 | train |
| 144211377 | C1(=C(C(=C(C(=C1Cl)Cl)Cl)Cl)Cl)[N+](=O)[O-] | 1 | validation |
| 144211376 | CCC(C)SP(=O)(N1CCSC1=O)OCC | 1 | test |
| 144211375 | C(SC#N)SC#N | 1 | train |
| 144211374 | C1=CC=C(C(=C1)C=O)S(=O)(=O)[O-].[Na+] | 0 | train |
| 144211373 | CC(C)C(=O)OCCOC1=CC=CC=C1 | 0 | validation |
| 144211372 | CC(C)(C)C1=CC(=CC(=C1O)C(C)(C)C)OC | 0 | test |
| 144211371 | CCC(CC)C(=O)O | 0 | train |
| 144211370 | CC(=C)C(=O)OCCOC1=CC=CC=C1 | 0 | train |
| 144211369 | CCCC(=C1C(=O)CC(CC1=O)CC(C)SCC)NOCC | 1 | validation |
| 144211368 | CC/C=C/CCCCCCCCCCOC(=O)C | 0 | test |
| 144211367 | C[C@H]1[C@@H](SC(=O)N1C(=O)NC2CCCCC2)C3=CC=C(C=C3)Cl | 1 | train |
| 144211366 | C1=CC(=C2C(=C1)OC(O2)(F)F)C3=CNC=C3C#N | 1 | train |
| 144211365 | C1(=C(C(=NC(=C1Cl)Cl)C(=O)O)Cl)N | 0 | validation |
| 144211364 | CC(=NOCC1=CC=CC=C1C(=NOC)C(=O)OC)C2=CC(=CC=C2)C(F)(F)F | 1 | test |
| 144211363 | CC(=NC#N)N(C)CC1=CN=C(C=C1)Cl | 0 | train |
| 144211362 | COC(=O)C1=CC=CC=C1C(=O)OC | 0 | train |
| 144211361 | CC1=C(C(=O)C[C@@H]1OC(=O)[C@@H]2[C@H](C2(C)C)C=C(C)C)CC=C | 1 | validation |
| 144211360 | CC(=O)OCC=CC1=CC=CC=C1 | 0 | test |
| 144211359 | CC1(CCCCC1)C(=O)NC2=C(C(=C(C=C2)O)Cl)Cl | 0 | train |
| 144211358 | CCCOCCC | 0 | train |
| 144211357 | CCOC(=O)C(C)OC1=CC=C(C=C1)OC2=CN=C3C=C(C=CC3=N2)Cl | 1 | validation |
| 144211356 | CCCCCC1CCC(=O)O1 | 0 | test |
| 144211355 | CCC1=NN(C(=C1Cl)C(=O)NCC2=CC=C(C=C2)C(C)(C)C)C | 0 | train |
| 144211354 | C(COC(=O)Cl)OCCOC(=O)Cl | 0 | train |
| 144211353 | C(C(=O)O)OCC(=O)O | 0 | validation |
| 144211352 | CO[Si](C)(C)OC | 0 | test |
| 144211351 | C1CNC(=S)N1 | 0 | train |
| 144211350 | CCCCCCCCCCCCCCC(C(=O)O)Br | 0 | train |
| 144211349 | CCNC1=NC(=NC(=N1)Cl)NC(C)(C)C#N | 0 | validation |
| 144211348 | CC(CCC=C(C)C)CCOC=O | 0 | test |

| | | | |
|---|---|---|---|
| 144211347 | CC(C)C1=CC(=CC(=C1)C(C)C)C(C)C | 0 | train |
| 144211346 | CCCCOC(=O)[C@@H](C)OC1=CC=C(C=C1)OC2=C(C=C(C=C2)C#N)F | 0 | train |
| 144211345 | C1CN(C(=N1)N[N+](=O)[O-])CC2=CN=C(C=C2)Cl | 0 | validation |
| 144211344 | CC(=O)OCCC1=CC=CC=C1 | 0 | test |
| 144211343 | C1=CC=C(C=C1)NC2=CC=CC=C2 | 0 | train |
| 144211342 | COC1=CC=C(C=C1)C(C2CC2)(C3=CN=CN=C3)O | 0 | train |
| 144211341 | CCOC1=C(C=CC(=C1)C(C)(C)C)C2COC(=N2)C3=C(C=CC=C3F)F | 1 | validation |
| 144211340 | CC1=C(C=CN=C1CS(=O)C2=NC3=CC=CC=C3N2)OCC(F)(F)F | 1 | test |
| 144211339 | C1=CC(=C(C=C1Cl)Cl)CC#N | 0 | train |
| 144211338 | CCNC1=NC(=NC(=N1)Cl)NC(C)C | 0 | train |
| 144211337 | CCCCCCCCCCCCC1=CC=C(C=C1)O | 1 | validation |
| 144211336 | CCCCCCC(CO)O | 0 | test |
| 144211335 | C1=CC=C2C=C(C=CC2=C1)S(=O)(=O)[O-].[Na+] | 1 | train |
| 144211334 | CC(=CC1C(C1(C)C)C(=O)OCN2C(=O)C3=C(C2=O)CCCC3)C | 1 | train |
| 144211333 | CN(C)C(=O)NC1=CC(=C(C=C1)Cl)Cl | 1 | validation |
| 144211332 | [Cl-].[Cl-].[Ba+2] | 0 | test |
| 144211331 | CC1=CC(=C(C=C1)C)S(=O)(=O)O | 0 | train |
| 144211330 | CNCCO | 0 | train |
| 144211329 | CCC1=CN=C(C(=C1)C(=O)O)C2=NC(C(=O)N2)(C)C(C)C | 0 | validation |
| 144211328 | CC/C=C¥CCO | 0 | test |
| 144211327 | COC1=CN=C(N2C1=NC(=N2)NS(=O)(=O)C3=C(C=CC=C3OCC(F)F)C(F)(F)F)OC | 0 | train |
| 144211326 | COP(=O)(OC)SCN1C2=NC=C(C=C2OC1=O)Cl | 0 | train |
| 144211325 | C1CCC(CC1)NS(=O)(=O)O | 0 | validation |
| 144211324 | CC(C)C1(C(=O)NC(=N1)C2=NC=C(C=C2C(=O)O)COC)C | 0 | test |
| 144211323 | CCCCCCCCCCOS(=O)(=O)[O-].[Na+] | 0 | train |
| 144211322 | CCCCCCOC(=O)CCCCC(=O)OCCCCCC | 0 | train |
| 144211321 | CC(C)NC1=NC(=NC(=N1)N=[N+]=[N-])SC | 1 | validation |
| 144211320 | CC1=C(N=C(N=C1OC(=O)N(C)C)N(C)C)C | 0 | test |
| 144211319 | C[Si](C)(C)O[Si](C)(C)O[Si](C)(C)C | 0 | train |
| 144211318 | CC(C)C1(C(=O)NC(=N1)C2=C(C=CC=N2)C(=O)O)C | 0 | train |
| 144211317 | B(O)(O)O | 0 | validation |
| 144211316 | C(CCO)CC(CO)O | 0 | test |
| 144211315 | COP(=S)(OC)OC1=CC=C(C=C1)[N+](=O)[O-] | 1 | train |
| 144211314 | C/C(=N¥NC(=O)NC1=CC(=CC(=C1)F)F)/C2=C(C=CC=N2)C(=O)[O-].[Na+] | 0 | train |
| 144211313 | COC(=O)/C=C¥C(=O)OC | 0 | validation |
| 144211312 | C1=CC(=C(C=C1Cl)Cl)C(CN2C=NC=N2)COC(C(F)F)(F)F | 0 | test |
| 144211311 | CCOC(=O)C(CC1=CC(=C(C=C1Cl)F)N2C(=O)N(C(=N2)C)C(F)F)Cl | 1 | train |
| 144211310 | COC1=CC(=C(C=C1)OC)OC | 0 | train |
| 144211309 | CNCCCC12CCC(C3=CC=CC=C31)C4=CC=CC=C24.Cl | 1 | validation |
| 144211308 | C1=CC(=CC=C1N=C=O)Cl | 1 | test |
| 144211307 | CN1CN(C(=S)SC1)C | 1 | train |
| 144211306 | C(=O)(N)NNC(=O)N | 0 | train |
| 144211305 | C1=CC(=CC=C1C(C2=CC=C(C=C2)Cl)(C(Cl)(Cl)Cl)O)Cl | 1 | validation |
| 144211304 | C(CSCCC(=O)O)C(=O)O | 0 | test |
| 144211303 | CCCCNC(=S)NCCCC | 1 | train |
| 144211302 | CCCCSCCCC | 0 | train |
| 144211301 | CC(C)NC1=NC(=NC(=N1)Cl)NC(C)C | 0 | validation |
| 144211300 | C1=CC=C(C=C1)NC(=O)CCl | 1 | test |
| 144211299 | CCC1=CC=CC(=C1N(C(C)COC)C(=O)CCl)C | 1 | train |
| 144211298 | C1(=C(C(=NC(=C1Cl)Cl)C(=O)O)Cl)Cl | 1 | train |
| 144211297 | CCCCOC(=O)C1=CC=CC=C1C(=O)OCCCC | 1 | validation |
| 144211296 | CCCCCC#CC(=O)OC | 0 | test |
| 144211295 | CCCC/C=C/CCCCO | 0 | train |
| 144211294 | CCC(C)CCCC(C)(C)O | 0 | train |
| 144211293 | C(CC#N)CC(CCC#N)C#N | 0 | validation |
| 144211292 | C1CC(=O)OC(=O)C1 | 0 | test |
| 144211291 | CCC(=O)OCCC1=CC=CC=C1 | 0 | train |
| 144211290 | C1=CC=C(C=C1)C2=CC=C(C=C2)N.Cl | 1 | train |
| 144211289 | CC(=O)C(CC(=O)OC)C(=O)OC | 0 | validation |
| 144211288 | C1CCCCCC(=O)OCCOC(=O)CCCCC1 | 0 | test |
| 144211287 | C1=CC=C2C(=C1)NC(=N2)C3=CSC=N3 | 1 | train |
| 144211286 | CC(=C)[C@H]1CCC(=CC1)C=O | 0 | train |

| | | | |
|---|---|---|---|
| 144211285 | CNC(=O)CSP(=O)(OC)OC | 0 | validation |
| 144211284 | CCCCCCCCCCCC(=O)OCC | 0 | test |
| 144211283 | CNC(=O)N(C)C1=NC2=CC=CC=C2S1 | 1 | train |
| 144211282 | CCOP(=S)(OC1=CN=C(N=C1)C(C)(C)C)OC(C)C | 0 | train |
| 144211281 | C(C(=O)NCO)Cl | 0 | validation |
| 144211280 | CC1=CCC(=CC1)C(C)C | 0 | test |
| 144211279 | COC=O | 0 | train |
| 144211278 | CC(C)CCCC(C)CCO | 0 | train |
| 144211277 | CN(C)S(=O)(=O)N(C1=CC=CC=C1)SC(F)(Cl)Cl | 1 | validation |
| 144211276 | C1=CC(=C(C(=C1)F)C(=O)NC(=O)NC2=CC(=C(C=C2)OC(C(OC(F)(F)F)F)(F)F)Cl)F | 0 | test |
| 144211275 | CC1(CON(C1=O)CC2=CC=CC=C2Cl)C | 1 | train |
| 144211274 | CS(=O)C | 0 | train |
| 144211273 | CC(=C)C(=O)OCCOCCOCCOCCOC(=O)C(=C)C | 0 | validation |
| 144211272 | CCN(CC1=C(C=CC=C1Cl)F)C2=C(C=C(C=C2[N+](=O)[O-])C(F)(F)F)[N+](=O)[O-] | 1 | test |
| 144211271 | C(CCCCC(=O)O)CCCCC(=O)O | 0 | train |
| 144211270 | C1(C(C(C(C(C1Cl)Cl)Cl)Cl)Cl)Cl | 0 | train |
| 144211269 | CC(=O)C1=CC=CN1 | 0 | validation |
| 144211268 | CCC[Si](OC)(OC)OC | 0 | test |
| 144211267 | CCCCCC/C=C¥CCCCCCCC(=O)O | 0 | train |
| 144211266 | [C@H]1([C@@H]2[C@@H]([C@H](C(=O)O2)O)O[C@H]1O)O | 0 | train |
| 144211265 | CC(C1=CC=CC=C1)OC(=O)C | 0 | validation |
| 144211264 | C1=CC=C(C(=C1)C2C(O2)(CN3C=NC=N3)C4=CC=C(C=C4)F)Cl | 0 | test |
| 144211263 | C1=CC(=C(C=C1[N+](=O)[O-])[N+](=O)[O-])ONC=C2C=C(C(=O)C(=C2)Br)Br | 1 | train |
| 144211262 | CC[C@@H]1[C@H](CC[C@@]2(O1)C[C@@H]3C[C@H](O2)C/C=C(/C[C@H](/C=C/C=C/4¥CO[C@H]5[C@@]4([C@@H](C=C([C@H]5O)C)C(=O)O3)O)C)¥C)C | 1 | train |
| 144211261 | CC1=NC(=NC(=C1)C2CC2)NC3=CC=CC=C3 | 1 | validation |
| 144211260 | CCC(C)(C(=O)CCl)NC(=O)C1=CC(=C(C(=C1)Cl)C)Cl | 1 | test |
| 144211259 | COC1=CC(=C2C=CC(=O)OC2=C1)OC | 1 | train |
| 144211258 | CC(CCC=C(C)C)C=O | 0 | train |
| 144211257 | CCCCOCN(C1=C(C=CC=C1CC)CC)C(=O)CCl | 1 | validation |
| 144211256 | CCC(CC)C(C)CCOC(=O)C | 0 | test |
| 144211255 | C=CC(=O)OCCCCOC(=O)C=C | 1 | train |
| 144211254 | C1=CC=C(C=C1)OC(=O)C2=CC=CC=C2C(=O)OC3=CC=CC=C3 | 0 | train |
| 144211253 | C1=CC=NC(=C1)CCO | 0 | validation |
| 144211252 | CC1(OC[C@H]2[C@@H](O1)[C@H]3[C@@](O2)(OC(O3)(C)C)C(=O)O)C | 0 | test |
| 144211251 | CC(CCCC(C)(C)O)CCO | 0 | train |
| 144211250 | C1=CC=C(C=C1)CN | 0 | train |
| 144211249 | CN(C)CCCN(C)C | 0 | validation |
| 144211248 | CC1=CC[C@H](CC1=O)C(=C)C | 0 | test |
| 144211247 | CC1=CC(=C(C=C1)N=CN(C)C=NC2=C(C=C(C=C2)C)C)C | 0 | train |
| 144211246 | COP(=S)(OC)SCN1C(=O)C2=CC=CC=C2N=N1 | 1 | train |
| 144211245 | CCC(=O)C=CC1=CC=C(C=C1)OC | 1 | validation |
| 144211244 | CCCCCCCC/C=C¥CCCCCCCC(=O)N(C)CC(=O)O | 0 | test |
| 144211243 | CCC(CC)CO | 0 | train |
| 144211242 | CC(C)(C)C(C(CC1=CC=C(C=C1)Cl)N2C=NC=N2)O | 0 | train |
| 144211241 | CCOC1=NC(=NS1)C(Cl)(Cl)Cl | 0 | validation |
| 144211240 | CS(=O)(=O)C1=C(C=CC(=C1)C(F)(F)F)C(=O)C2=C(ON=C2)C3CC3 | 0 | test |
| 144211239 | CC1=CC(=CC=C1)Cl | 0 | train |
| 144211238 | CCN(CC1=CC(=CC=C1)S(=O)(=O)O)C2=CC=CC=C2 | 0 | train |
| 144211237 | CC(=CC1C(C1(C)C)C(=O)OCC2=COC(=C2)CC3=CC=CC=C3)C | 1 | validation |
| 144211236 | CN(C)NC(=O)CCC(=O)O | 0 | test |
| 144211235 | CCCN(CCC)C1=C(C=C(C=C1[N+](=O)[O-])C(F)(F)F)[N+](=O)[O-] | 0 | train |
| 144211234 | CCN(CC)C(=O)C1=CC(=CC=C1)C | 0 | train |
| 144211233 | C1=CC(=NC(=C1)Cl)C(Cl)(Cl)Cl | 0 | validation |
| 144211232 | CC1=CC(=O)OC2=C1C=CC(=C2)O | 0 | test |
| 144211231 | C1=C(C=C(C(=C1I)I)C(=O)O)I | 0 | train |
| 144211230 | CCOC(=O)C1=CC=C(C=C1)N(C)C | 1 | train |
| 144211229 | CN=C=S | 0 | validation |
| 144211228 | COC1=C(C=C(C=C1)C(=CC(=O)N2CCOCC2)C3=CC=C(C=C3)Cl)OC | 0 | test |
| 144211227 | COP(=S)(OC)OC1=CC(=C(C=C1Cl)I)Cl | 1 | train |
| 144211226 | CC1=CC(=C(C=C1Cl)C(C)C)O | 0 | train |
| 144211225 | CCCCCCCCCCCCCC[N+](C)(C)C.[Cl-] | 1 | validation |

| | | | |
|---|---|---|---|
| 144211224 | C1=CC(=C(C(=C1)F)C(=O)NC(=O)NC2=CC(=C(C=C2Cl)OC(C(C(F)(F)F)F)(F)F)Cl)F | 0 | test |
| 144211223 | CC1=C(C=CC(=C1)OP(=S)(OC)OC)[N+](=O)[O-] | 1 | train |
| 144211222 | C1=CC(=C(C=C1O)C(F)(F)F)[N+](=O)[O-] | 1 | train |
| 144211221 | C1=CC=C(C(=C1)C2=NN=C(N=N2)C3=CC=CC=C3Cl)Cl | 1 | validation |
| 144211220 | CCCCCC(CCC)CO | 0 | test |
| 144211219 | CN(C)C(=O)NC1=CC=CC=C1 | 0 | train |
| 144211218 | CC1=CN=C(C(=C1)C(=O)O)C2=NC(C(=O)N2)(C)C(C)C | 0 | train |
| 144211217 | CC1=C(C(=C(C(=C1F)F)COC(=O)C2C(C2(C)C)/C=C(/C(F)(F)F)¥Cl)F)F | 0 | validation |
| 144211216 | CC1=CC=C(C=C1)S(=O)(=O)[N-]Cl.O.O.[Na+] | 0 | test |
| 144211215 | CC1=NN(C(=C1/C=N/OCC2=CC=C(C=C2)C(=O)OC(C)(C)C)OC3=CC=CC=C3)C | 0 | train |
| 144211214 | CCCCCCC(C)OC(=O)COC1=NC(=C(C(=C1Cl)N)Cl)F | 1 | train |
| 144211213 | CCOP(=S)(OCC)OC(C(Cl)(Cl)Cl)Cl | 1 | validation |
| 144211212 | C1=CC(=C(C=C1Cl)Cl)CO | 0 | test |
| 144211211 | C1=CC=C2C(=C1)N=CC(=N2)NS(=O)(=O)C3=CC=C(C=C3)N | 0 | train |
| 144211210 | CC(C)NC1=NC(=NC(=N1)OC)NC(C)C | 0 | train |
| 144211209 | CC1(C(C1C(=O)OC(C#N)C2=CC(=CC=C2)OC3=CC=CC=C3)C=C(Cl)Cl)C | 1 | validation |
| 144211208 | CCCC(=O)OCC1=CC=CC=C1 | 0 | test |
| 144211207 | C1=CC(=C(C=C1Cl)Cl)OCC(=O)O | 0 | train |
| 144211206 | CCC1=CC=CC(=C1N(COCC)C(=O)CCl)C | 1 | train |
| 144211205 | CCCCOC(=O)C1=CC=CC=C1C(=O)O | 0 | validation |
| 144211204 | CC(C)(C)C(/C(=C¥C1=C(C=C(C=C1)Cl)Cl)/N2C=NC=N2)O | 1 | test |
| 144211203 | CC(C)(C)C(=O)C(N1C=NC=N1)OC2=CC=C(C=C2)Cl | 0 | train |
| 144211202 | CCCCC(CC)COCCC#N | 0 | train |
| 144211201 | CCN(C1CCCCC1)C(=O)SCC | 0 | validation |
| 144211200 | C1=C[N+](=CC=C1Cl)[O-] | 0 | test |
| 144211199 | CC1(C(C1(C)C)C(=O)OC(C#N)C2=CC(=CC=C2)OC3=CC=CC=C3)C | 1 | train |
| 144211198 | CC1=CC=C(C2=CC=CC=C12)C | 0 | train |
| 144211197 | C(CCO)CCO | 0 | validation |
| 144211196 | CC1=NNC(=O)N(C1)/N=C/C2=CN=CC=C2 | 0 | test |
| 144211195 | C1=CC=C(C=C1)[Hg]Cl | 1 | train |
| 144211194 | C1=CC2=C(C=C1OC3=CC4=C(C=C3)C(=O)OC4=O)C(=O)OC2=O | 0 | train |
| 144211193 | C1=CC(=NC(=C1Cl)C(=O)O)Cl | 0 | validation |
| 144211192 | CCC1CCC(=O)O1 | 0 | test |
| 144211191 | CC(=O)NP(=O)(OC)SC | 0 | train |
| 144211190 | CC(=O)CC(C)(C)OC | 0 | train |
| 144211189 | CC1(CC(CC(N1[O])(C)C)O)C | 0 | validation |
| 144211188 | CC(C)OC(=O)NNC1=C(C=CC(=C1)C2=CC=CC=C2)OC | 1 | test |
| 144211187 | C1(=C(SN=C1Cl)C(=O)O)Cl | 0 | train |
| 144211186 | CCCCCCCCCO | 0 | train |
| 144211185 | CCOC(=O)C(C)OC(=O)C1=C(C=CC(=C1)OC2=C(C=C(C=C2)C(F)(F)F)Cl)[N+](=O)[O-] | 1 | validation |
| 144211184 | CCC(CC)NC1=C(C=C(C(=C1[N+](=O)[O-])C)C)[N+](=O)[O-] | 1 | test |
| 144211183 | CCCOCC(=NC1=C(C=C(C=C1)Cl)C(F)(F)F)N2C=CN=C2 | 1 | train |
| 144211182 | CC(C)NC(=O)N1CC(=O)N(C1=O)C2=CC(=CC(=C2)Cl)Cl | 0 | train |
| 144211181 | CSC(=O)C1=C2C(=CC=C1)N=NS2 | 0 | validation |
| 144211180 | CCNP(=S)(OC)O/C(=C/C(=O)OC(C)C)/C | 1 | test |
| 144211179 | C(#N)C1=C(C(=C(C(=C1Cl)Cl)Cl)C#N)Cl | 1 | train |
| 144211178 | CCOP(=S)(OCC)SCCSCC | 0 | train |
| 144211177 | COP(=O)(C(C(Cl)(Cl)Cl)O)OC | 0 | validation |
| 144211176 | CCCC1COC(O1)(CN2C=NC=N2)C3=C(C=C(C=C3)Cl)Cl | 1 | test |
| 144211175 | C1(=O)N(C(=O)N(C(=O)N1Cl)Cl)Cl | 0 | train |
| 144211174 | CC1=C(C=CC(=C1)Cl)N | 0 | train |
| 144211173 | CC1=CC(=O)NO1 | 0 | validation |
| 144211172 | C/C(=N¥OC(=O)N(SN(C(=O)O/N=C(/SC)¥C)C)C)/SC | 1 | test |
| 144211171 | CNC(=O)OC1=CC=CC2=CC=CC=C21 | 1 | train |
| 144211170 | CCCCCCCCCCCCCCCC[N+](C)(C)C.[Br-] | 1 | train |
| 144211169 | CCOC(=O)COC1=C(C=C(C(=C1)N2C(=O)C(=C(C=N2)C(F)(F)F)C)F)Cl | 0 | validation |
| 144211168 | COC(=O)NS(=O)(=O)C1=CC=C(C=C1)N | 0 | test |
| 144211167 | CC(=O)NC1=CC=C(C=C1)S(=O)(=O)N | 0 | train |
| 144211166 | COC1=CC(=NC(=N1)SC2=C(C(=CC=C2)Cl)C(=O)[O-])OC.[Na+] | 0 | train |
| 144211165 | CCC(=O)NC1=CC(=C(C=C1)Cl)Cl | 0 | validation |
| 144211164 | C1=CC=C(C=C1)C=CCO | 0 | test |
| 144211163 | CC(C)(C=NOC(=O)NC)SC | 0 | train |

| | | | |
|---|---|---|---|
| 144211162 | COP(=O)(OC)OC(C(Cl)(Cl)Br)Br | 0 | train |
| 144211161 | C1=CC(=CC=C1OC2=C3C(=CC(=CC3=NC=C2)Cl)Cl)F | 1 | validation |
| 144211160 | C[C@H](C(=O)O)O | 0 | test |
| 144211159 | C1=CC=C(C=C1)OCCO | 0 | train |
| 144211158 | CC[C@H](C)[C@@H]1[C@H](C=C[C@@]2(O1)C[C@@H]3C[C@H](O2)C/C=C(/[C@H]([C@H](/C=C/C=C/4¥CO[C@H]5[C@@]4([C@@H](C=C([C@H]5O)C)C(=O)O3)O)C)O[C@H]6C[C@@H]([C@H]([C@@H](O6)C)O[C@H]7C[C@@H]([C@H]([C@@H](O7)C)O)OC)OC)¥C)C | 1 | train |
| 144211157 | COP(=O)(OC)OC=C(Cl)Cl | 0 | validation |
| 144211156 | C1=CC(=CN=C1)CO | 0 | test |
| 144211155 | C1=C(C=C(C(=C1Cl)N)Cl)[N+](=O)[O-] | 0 | train |
| 144211154 | C1=CC(=CC=C1NC(=O)N)Cl | 0 | train |
| 144211153 | C/C=C/C=C/CCCCCCCO | 0 | validation |
| 144211152 | CC(C)C(C(C)(C)COC(=O)C1=CC=CC=C1)OC(=O)C2=CC=CC=C2 | 1 | test |
| 144211151 | COC(=O)CSC1=C(C=C(C(=C1)N=C2N3CCCCN3C(=O)S2)F)Cl | 0 | train |
| 144211150 | CCOP(=S)(OCC)OC1=NN(C(=N1)Cl)C(C)C | 1 | train |
| 144211149 | CC(=C)C(=O)OCCOC1CC2CC1C3C2CC=C3 | 0 | validation |
| 144211148 | CC1=C(C(=O)N(C(=O)N1)C(C)(C)C)Cl | 0 | test |
| 144211147 | COC(=O)NC(=S)NC1=CC=CC=C1NC(=S)NC(=O)OC | 0 | train |
| 144211146 | CCCCC/C=C¥CCC=O | 0 | train |
| 144211145 | CC1=NC2=NC(=NN2C=C1)S(=O)(=O)NC3=C(C=CC=C3F)F | 0 | validation |
| 144211144 | CCOC1=C(C=CC(=C1)OC2=C(C=C(C=C2)C(F)(F)F)Cl)[N+](=O)[O-] | 0 | test |
| 144211143 | C(CNC(=S)[S-])NC(=S)[S-].[Mn+2] | 1 | train |
| 144211142 | CCOP(=S)(OCC)SCCl | 0 | train |
| 144211141 | CO/N=C(¥C1=CC=CC=C1OC2=C(C(=NC=N2)OC3=CC=CC=C3Cl)F)/C4=NOCCO4 | 1 | validation |
| 144211140 | CC1C(=O)C(=C(O1)C)O | 0 | test |
| 144211139 | CC(C)CC1=C(C(=NC(=C1C(=O)OC)C(F)F)C(F)(F)F)C2=NCCS2 | 1 | train |
| 144211138 | CCCCCOC(=O)COC1=C(C=C(C(=C1)N2C(=O)C3=C(C2=O)CCCC3)F)Cl | 0 | train |
| 144211137 | C1=CC=C(C=C1)CCC2=CC=CC=C2 | 0 | validation |
| 144211136 | Cl[Dy](Cl)Cl | 0 | test |
| 144211135 | C1=CC(=CC(=C1)OC2=C(C=CC=N2)C(=O)NC3=C(C=C(C=C3)F)F)C(F)(F)F | 0 | train |
| 144211134 | CCSC(=O)N1CCCCCC1 | 0 | train |
| 144211133 | CC1(CC(=O)CC(N1)(C)C)C | 0 | validation |
| 144211132 | CCCCCC/C=C/C=O | 0 | test |
| 144211131 | [N+](=O)([O-])[O-].[N+](=O)([O-])[O-].[Ca+2] | 0 | train |
| 144211130 | CCCCN(CCO)CCO | 0 | train |
| 144211129 | CC/C(=C/CCC(C)(C=C)O)/C | 0 | validation |
| 144211128 | CCOP(=S)(OCC)SCN1C2=C(C=C(C=C2)Cl)OC1=O | 1 | test |
| 144211127 | CCOC(=O)CC1=CC=CC=C1 | 0 | train |
| 144211126 | CSC1=C(C(=C(C(=C1Cl)Cl)Cl)Cl)Cl | 1 | train |
| 144211125 | CC1=CC(=NC(=N1)NC2=CC=CC=C2)C | 1 | validation |
| 144211124 | CC(C)NC1=NC(=NC(=N1)SC)NC(C)C | 1 | test |
| 144211123 | C/C(=C¥C(=O)NC)/OP(=O)(OC)OC | 0 | train |
| 144211122 | CC(C)(C)C1=CC=C(C=C1)OC2CCCCC2OS(=O)OCC#C | 1 | train |
| 144211121 | CCN(C1=CC=CC=C1C)C(=O)C=CC | 1 | validation |
| 144211120 | C[C@@H](CCC=C(C)C)C=C | 0 | test |
| 144211119 | CC(C)(C)C1=NN=C(S1)N(C)C(=O)NC | 1 | train |
| 144211118 | C1C=CCC2C1C(=O)N(C2=O)SC(C(Cl)Cl)(Cl)Cl | 1 | train |
| 144211117 | CCC1=C(C(=O)C=CO1)O | 0 | validation |
| 144211116 | CCCCCCCCC(CCCCCC)CO | 0 | test |
| 144211115 | CC[N+](C)(CC)CC.[Cl-] | 0 | train |
| 144211114 | COCCOC(=O)CC#N | 0 | train |
| 144211113 | CC(C)C1(C(=O)NC(=N1)C2=NC3=CC=CC=C3C=C2C(=O)O)C | 0 | validation |
| 144211112 | C1=CC=C(C=C1)C(CCC2=CC=C(C=C2)Cl)(CN3C=NC=N3)C#N | 1 | test |
| 144211111 | CCOP(=S)(OCC)SC(CCl)N1C(=O)C2=CC=CC=C2C1=O | 1 | train |
| 144211110 | CN(C(=O)NC1=CC(=C(C=C1)Br)Cl)OC | 1 | train |
| 144211109 | COC(=O)CC1=CC=CC=C1 | 0 | validation |
| 144211108 | C1=CC(=C(C(=C1)Cl)C#N)Cl | 0 | test |
| 144211107 | CC1=C(C(=CC=C1)C)N(C(C)C(=O)OC)C(=O)COC | 0 | train |
| 144211106 | CC(C(C)O)O | 0 | train |
| 144211105 | CC1=CC=C(C=C1)C2=C(N=C(N2S(=O)(=O)N(C)C)C#N)Cl | 0 | validation |

| | | | |
|---|---|---|---|
| 144211104 | CC[C@H](C)[C@@H]1[C@H](C=C[C@@]2(O1)C[C@@H]3C[C@H](O2)C/C=C(/[C@H]([C@H](/C=C/C=C/4¥CO[C@H]5[C@@]4([C@@H](C=C([C@H]5O)C)C(=O)O3)O)C)O[C@H]6C[C@@H]([C@H]([C@@H](O6)C)O[C@H]7C[C@@H]([C@H]([C@@H](O7)C)[NH2+]C)OC)OC)¥C)C.C1=CC=C(C=C1)C(=O)[O-] | 1 | test |
| 144211103 | CCCCC(CC)CN1C(=O)C2C3CC(C2C1=O)C=C3 | 1 | train |
| 144211102 | CC(C)[C@@H](C1=CC=C(C=C1)Cl)C(=O)O[C@H](C#N)C2=CC(=CC=C2)OC3=CC=CC=C3 | 1 | train |
| 144211101 | C1CC1NC2=NC(=NC(=N2)N)N | 0 | validation |
| 144211100 | C1=CC(=CC(=C1)OCCO)OCCO | 1 | test |
| 144211099 | C1C2C(COS(=O)(=O)O1)C3(C(=C(C2(C3(Cl)Cl)Cl)Cl)Cl)Cl | 1 | train |
| 144211098 | CC(C)NC1=C2C(=C(C=C1)NC(C)C)C(=O)C3=CC=CC=C3C2=O | 0 | train |
| 144211097 | CC1(CCC(C2=C1C=CC(=C2)C(=O)NC3=CC=C(C=C3)C(=O)O)(C)C)C | 1 | validation |
| 144211096 | CC(C(=O)OC)OC1=CC=C(C=C1)OC2=C(C=C(C=C2)Cl)Cl | 0 | test |
| 144211095 | CNC(=O)OC1=CC=CC(=C1)N=CN(C)C.Cl | 0 | train |
| 144211094 | CC1(OC2=C(O1)C(=CC=C2)OC(=O)NC)C | 0 | train |
| 144211093 | CC(=O)N | 0 | validation |
| 144211092 | C1=C(C=C(C(=C1[N+](=O)[O-])Cl)C(=O)O)Cl | 0 | test |
| 144211091 | CCCCN(CC)C1=C(C=C(C=C1[N+](=O)[O-])C(F)(F)F)[N+](=O)[O-] | 1 | train |
| 144211090 | C[N+]1(CCCCC1)C.[Cl-] | 0 | train |
| 144211089 | C1C=CCC2C1C(=O)N(C2=O)SC(Cl)(Cl)Cl | 1 | validation |
| 144211088 | [N+](=O)(O)[O-].[N+](=O)(O)[O-].[Cd] | 1 | test |
| 144211087 | C(CNCCC#N)C#N | 0 | train |
| 144211086 | CCOP(=S)(OCC)OC1=CC2=C(C=C1)C(=C(C(=O)O2)Cl)C | 1 | train |
| 144211085 | C1=C(C=C(C=C1C(=O)O)[N+](=O)[O-])C(=O)O | 0 | validation |
| 144211084 | C[As](=O)(C)O | 0 | test |
| 144211083 | C1=CC2=C(C=CC(=C2N=C1)O)S(=O)(=O)O.O | 1 | train |
| 144211082 | CCOC1=NC(=CC2=NC(=NN21)S(=O)(=O)NC3=C(C=CC=C3Cl)Cl)F | 0 | train |
| 144211081 | C1=CC(=C(C(=C1)F)C(=O)NC(=O)NC2=C(C=C(C=C2)OC3=C(C=C(C=C3)C(F)(F)F)Cl)F)F | 0 | validation |
| 144211080 | C1=CC(=NC(=C1Cl)C(=O)O)Cl.C(CO)N | 0 | test |
| 144211079 | CCSC(=O)N(CC(C)C)CC(C)C | 0 | train |
| 144211078 | C[Si](CN1C=NC=N1)(C2=CC=C(C=C2)F)C3=CC=C(C=C3)F | 1 | train |
| 144211077 | C1=CC(=C(C=C1C(F)(F)F)Cl)OC2=CC(=C(C=C2)[N+](=O)[O-])C(=O)O | 0 | validation |
| 144211076 | CCCCCCCCCCCC(=O)OCC(CO)O | 0 | test |
| 144211075 | COC1=C(C=C(C=C1)Cl)C(=O)NCCC2=CC=C(C=C2)S(=O)(=O)NC(=O)NC3CCCCC3 | 0 | train |
| 144211074 | C[Si]1(N[Si](N[Si](N1)(C)C)(C)C)C | 0 | train |
| 144211073 | C[N+](C)(C)CC1=CC=CC=C1.[OH-] | 0 | validation |
| 144211072 | COC1=CC=C(C=C1)CCO | 0 | test |
| 144211071 | C1=CC=C(C=C1)[Sn](C2=CC=CC=C2)C3=CC=CC=C3.O | 1 | train |
| 144211070 | CCNC1=NC(=NC(=N1)N)Cl | 0 | train |
| 144211069 | CCCSP(=O)(OCC)OC1=C(C=C(C=C1)Br)Cl | 1 | validation |
| 144211068 | CCO[Si](CCC1CCC2C(C1)O2)(OCC)OCC | 0 | test |
| 144211067 | C(CO)CO | 0 | train |
| 144211066 | C1=CC(=C(C=C1[N+](=O)[O-])Cl)NC(=O)C2=C(C=CC(=C2)Cl)O | 1 | train |
| 144211065 | CCNC1=NC(=NC(=N1)Cl)NCC | 0 | validation |
| 144211064 | C1=CC(=C(C(=C1Cl)C(=O)O)Cl)Cl | 1 | test |
| 144211063 | CC1=C(C=CC(=C1)Cl)OCC(=O)O | 0 | train |
| 144211062 | CN1C(=NC(=O)N(C1=O)C2CCCCC2)N(C)C | 0 | train |
| 144211061 | C1=CC(=CC=C1OCC(=O)O)Cl | 0 | validation |
| 144211060 | CC(C)(C)C1=CC(=CC(=C1O)[N+](=O)[O-])[N+](=O)[O-] | 0 | test |
| 144211059 | CC(C)N1C(=O)C2=CC=CC=C2NS1(=O)=O | 0 | train |
| 144211058 | C1=CN=CC=C1C#N | 0 | train |
| 144211057 | COP(=O)(N)SC | 0 | validation |
| 144211056 | COC1=CC(=C(C=C1Cl)OC)Cl | 0 | test |
| 144211055 | C1=CC(=C(C2=NC=C(C=C21)Cl)C(=O)O)Cl | 0 | train |
| 144211054 | C=CCOC1=NC(=NC(=N1)OCC=C)OCC=C | 1 | train |
| 144211053 | CNC(=S)[S-].O.O.O.[Na+] | 0 | validation |
| 144211052 | COC(=O)C1=CC=CC=C1C(=O)O | 0 | test |
| 144211051 | C1=CC=C2C(=C1)C(=O)C(C2=O)C3=NC4=CC=CC=C4C(=C3O)Br | 1 | train |
| 144211050 | CC(C)OP(=S)(OC(C)C)SCCNS(=O)(=O)C1=CC=CC=C1 | 1 | train |
| 144211049 | CN(C(=O)NC1=CC(=C(C=C1)Cl)Cl)OC | 1 | validation |
| 144211048 | CC(C)(C)C1=NN=C(N(C1=O)N)SC | 0 | test |
| 144211047 | CCOP(=S)(OCC)OC1=NC(=NC(=C1)C)C(C)C | 1 | train |
| 144211046 | CCCOC(=O)C1=CN=C(C=C1)C(=O)OCCC | 0 | train |

| | | | |
|---|---|---|---|
| 144211045 | C[Si](C)O[Si](C)(C)O[Si](C)(C)O[Si](C)C | 0 | validation |
| 144211044 | CCCN(CCC)C(=O)SCC | 0 | test |
| 144211043 | CC1=NC(=NC(=N1)OC)NC(=O)[N-]S(=O)(=O)C2=C(C=CC(=C2)I)C(=O)OC.[Na+] | 0 | train |
| 144211042 | C1=CC(=CC=C1C(CO)C2=CC=C(C=C2)Cl)Cl | 1 | train |
| 144211041 | C[C@H](C(=O)OCC#C)OC1=CC=C(C=C1)OC2=NC=C(C=C2F)Cl | 0 | validation |
| 144211040 | COC(=O)[C@]12CC3=C(C1=NN(CO2)C(=O)N(C4=CC=C(C=C4)OC(F)(F)F)C(=O)OC)C=CC(=C3)Cl | 0 | test |
| 144211039 | CC(C)[C@H](C(=O)OC(C#N)C1=CC(=CC=C1)OC2=CC=CC=C2)NC3=C(C=C(C=C3)C(F)(F)F)Cl | 0 | train |
| 144211038 | C1=CC=C(C=C1)N2C(=O)C(=C(C=N2)N)Cl | 0 | train |
| 144211037 | C=CCOC(CN1C=CN=C1)C2=C(C=C(C=C2)Cl)Cl | 1 | validation |
| 144211036 | [Cu]I | 0 | test |
| 144211035 | CCN(CC)C1=NC(=CC(=N1)OP(=S)(OC)OC)C | 1 | train |
| 144211034 | CC1=C(C=CC(=C1)Cl)OC | 0 | train |
| 144211033 | CC(COC1=CC=C(C=C1)OC2=CC=CC=C2)OC3=CC=CC=N3 | 1 | validation |
| 144211032 | CN(C)C(=O)NC1=CC=CC(=C1)C(F)(F)F | 0 | test |
| 144211031 | C(NC(=O)NCO)O | 0 | train |
| 144211030 | CCC1=C(C(=CC=C1)CC)N(COC)C(=O)CCl | 1 | train |
| 144211029 | CC(C)C1=CC=C(C=C1)C=O | 0 | validation |
| 144211028 | C[C@@H](CCC=C(C)C)CCO | 0 | test |
| 144211027 | CC1=CC=C(C=C1)S(=O)(=O)O.O | 0 | train |
| 144211026 | CC1=C(C(CCC1)(C)C)C=CC(=O)C | 0 | train |
| 144211025 | CN1C(=CC(=[N+]1C)C2=CC=CC=C2)C3=CC=CC=C3.COS(=O)(=O)[O-] | 0 | validation |
| 144211024 | CN(C)C1=CC=C(C=C1)C=O | 0 | test |
| 144211023 | CC1=C(SCCO1)C(=O)NC2=CC=CC=C2 | 1 | train |
| 144211022 | CC1=CSC(=C1N(C(C)COC)C(=O)CCl)C | 1 | train |
| 144211021 | C1=C(C(=CC(=C1Cl)Cl)Cl)[O-].[Na+] | 0 | validation |
| 144211020 | C1=CC2=C(C=CC=C2S(=O)(=O)[O-])C(=C1)S(=O)(=O)[O-].O.[Na+].[Na+] | 0 | test |
| 144211019 | CCNC1=NC(=NC(=N1)SC)NCC | 0 | train |
| 144211018 | CNC1=C(C(=O)N(N=C1)C2=CC=CC(=C2)C(F)(F)F)Cl | 1 | train |
| 144211017 | COP(=S)(OC)OC1=NC(=C(C=C1Cl)Cl)Cl | 1 | validation |
| 144211016 | C1=CC=C(C=C1)COC(=O)C=CC2=CC=CC=C2 | 1 | test |
| 144211015 | CCOC(=O)COC1=C(C=C(C(=C1)C2=NN(C(=C2Cl)OC(F)F)C)F)Cl | 0 | train |
| 144211014 | C1CSC(=NC#N)N1CC2=CN=C(C=C2)Cl | 0 | train |
| 144211013 | CCC(C)(CC)C1=NOC(=C1)NC(=O)C2=C(C=CC=C2OC)OC | 0 | validation |
| 144211012 | CC(=C)[C@H]1CC2=C(O1)C=CC3=C2O[C@@H]4COC5=CC(=C(C=C5[C@@H]4C3=O)OC)OC | 0 | test |
| 144211011 | CCCN(CCC)C1=C(C=C(C(=C1[N+](=O)[O-])N)C(F)(F)F)[N+](=O)[O-] | 1 | train |
| 144211010 | C1COCO1 | 0 | train |
| 144211009 | CCCC(C)C(CC)O | 0 | validation |
| 144211008 | CC1(C(C1C(=O)OCC2=CC(=CC=C2)OC3=CC=CC=C3)C=C(Cl)Cl)C | 1 | test |
| 144211007 | C1=C[N+](=CC=C1[N+](=O)[O-])[O-] | 1 | train |
| 144211006 | C1=CC=C(C=C1)CCOC(=O)CC2=CC=CC=C2 | 0 | train |
| 144211005 | C(=O)(C(C(C(C(C(C(C(F)(F)F)(F)F)(F)F)(F)F)(F)F)(F)F)(F)F)O | 0 | validation |
| 144211004 | CC(=O)C1=CC=C(C=C1)OC | 0 | test |
| 144211003 | C1=CC=C(C=C1)C(=O)CC(=O)C2=CC=CC=C2 | 1 | train |
| 144211002 | CCC1=CC=C(C=C1)C(=O)NN(C(=O)C2=CC(=CC(=C2)C)C)C(C)(C)C | 0 | train |
| 144211001 | CCCCC(CN1C=NC=N1)(C2=C(C=C(C=C2)Cl)Cl)O | 1 | validation |
| 144211000 | C1=CC=C2C(=C1)C(=O)C3=C(C=CC(=C3C2=O)N)N | 0 | test |
| 144210999 | CCC1=CN=C(C(=C1)C(=O)O)C(=O)O | 0 | train |
| 144210998 | CCCOC(=O)NCCCN(C)C.Cl | 0 | train |
| 144210997 | CCNC(=O)NC(=O)/C(=N/OC)/C#N | 0 | validation |
| 144210996 | C[C@@]1([C@H]2[C@@H]([C@H]3[C@@H](C(=O)C(=C([C@]3(C(=O)C2=C(C4=C1C=CC=C4O)O)O)O)C(=O)N)N(C)C)O)O.O.O | 0 | test |
| 144210995 | C(#N)N | 0 | train |
| 144210994 | C1=CC=C(C=C1)C=C[N+](=O)[O-] | 1 | train |
| 144210993 | CC(C(=O)O)OC1=CC(=CC=C1)Cl | 0 | validation |
| 144210992 | COC(CC1=CC=CC=C1)OC | 0 | test |
| 144210991 | C1=CC=C(C=C1)C2=CC=CC=C2O | 0 | train |
| 144210990 | CCCCC/C=C¥C=C¥C(=O)OCC | 0 | train |
| 144210989 | C1=CC=C(C=C1)N2C(=O)C=CC2=O | 1 | validation |
| 144210988 | CC(C)N(C(C)C)C(=O)SCC(=C(Cl)Cl)Cl | 0 | test |

| | | | |
|---|---|---|---|
| 144210987 | C=CCN(CC=C)CC=C | 0 | train |
| 144210986 | C1=CC(=CC(=C1)Cl)NC(=O)OCC#CCCl | 1 | train |
| 144210985 | CC(C)OC1=C(C=C(C(=C1)N2C(=O)OC(=N2)C(C)(C)C)Cl)Cl | 1 | validation |
| 144210984 | CC1=NC(=CC=C1)N | 0 | test |
| 144210983 | CCNC1=NC(=NC(=N1)SC)NC(C)C | 1 | train |
| 144210982 | COC(=O)CC1=CC=CC2=CC=CC=C21 | 1 | train |
| 144210981 | CCCCCCCCCCCCN(CCCCCCCCCCCC)CCCCCCCCCCCC | 0 | validation |
| 144210980 | CCOP(=S)(OCC)OC1=CC=C(C=C1)[N+](=O)[O-] | 1 | test |
| 144210979 | C1=CC(=O)NNC1=O | 0 | train |
| 144210978 | COC1=C(C=CC(=C1C(=O)O)Cl)Cl | 0 | train |
| 144210977 | CC1=C(C=CC=C1Cl)[N+](=O)[O-] | 0 | validation |
| 144210976 | C1CCOC2(C1)CCCCO2 | 0 | test |
| 144210975 | CC1=C(C=CC(=C1)OP(=S)(OC)OC)SC | 1 | train |
| 144210974 | C1C2C(COS(=O)O1)C3(C(=C(C2(C3(Cl)Cl)Cl)Cl)Cl)Cl | 1 | train |
| 144210973 | C1=CC(=CC=C1C(C2=CC=C(C=C2)O)C(Cl)(Cl)Cl)O | 1 | validation |
| 144210972 | CCN(CC)C(=O)C(C)OC1=CC=CC2=CC=CC=C21 | 1 | test |
| 144210971 | C1=CC(=CC(=C1)C(F)(F)F)C(=O)O | 0 | train |
| 144210970 | CCOC(=O)C1=NN(C(=N1)C(Cl)(Cl)Cl)C2=C(C=C(C=C2)Cl)Cl | 1 | train |
| 144210969 | C1=C(C=C(C(=C1Cl)N)[N+](=O)[O-])Cl | 0 | validation |
| 144210968 | C1=CC=C(C=C1)NC(=O)NC2=CN=NS2 | 1 | test |
| 144210967 | C1=CC=C(C(=C1)C(C2=CC=C(C=C2)Cl)(C3=CN=CN=C3)O)Cl | 1 | train |
| 144210966 | C1CC(CCC1C(=O)O)C(=O)O | 0 | train |
| 144210965 | C1=CSC(=C1)C2=CC=C(S2)C3=CC=CS3 | 1 | validation |
| 144210964 | C1=C(OC(=C1)[N+](=O)[O-])C2=NN=C(O2)N | 1 | test |
| 144210963 | Cl[Nd](Cl)Cl | 0 | train |
| 144210962 | CC1(C(C1C(=O)OC(C#N)C2=CC(=C(C=C2)F)OC3=CC=CC=C3)C=C(Cl)Cl)C | 0 | train |
| 144210961 | CC1=C(C(=O)C[C@H]1OC(=O)[C@H]2[C@@H](C2(C)C)C=C(C)C)CC#C | 1 | validation |
| 144210960 | CCCC(=O)OC(C)(C)CC1=CC=CC=C1 | 0 | test |
| 144210959 | CCCCSP(=O)(SCCCC)SCCCC | 1 | train |
| 144210958 | Cl[Sb](Cl)Cl | 0 | train |
| 144210957 | CCCCCCCCOC(=O)C1=CC(=C(C=C1)C(=O)OCCCCCCCC)C(=O)OCCCCCCCC | 0 | validation |
| 144210956 | CC(C)CCCCCCOC(=O)C1=CC(=C(C=C1)C(=O)OCCCCCCC(C)C)C(=O)OCCCCCCC(C)C | 0 | test |
| 144210955 | CC(C)CCCCCCOC(=O)C1CCCCC1C(=O)OCCCCCCC(C)C | 0 | train |
| 144210954 | C(CO)O | 0 | train |
| 144210953 | COS(=O)(=O)OC | 0 | validation |
| 144210952 | CC#CC1=CC(=NC(=N1)NC2=CC=CC=C2)C | 1 | test |
| 144210951 | CC1=C(C(=CC=C1)NC2=CC(=NC(=N2)SCC(=O)O)Cl)C | 0 | train |
| 144210950 | CCC(=O)C(CC(C)N(C)C)(C1=CC=CC=C1)C2=CC=CC=C2.Cl | 0 | train |
| 144210949 | C[C@H]1C[C@@H]2[C@H]([C@H]([C@]3(O2)CC[C@H]4[C@@H]5CC=C6C[C@H](CC[C@@]6([C@H]5CC4=C3C)C)O)C)NC1 | 0 | validation |
| 144210948 | CC(C)C1=C(C(=CC=C1)C(C)C)N2C(=O)C3=C(C2=O)C=C(C=C3)O | 0 | test |
| 144210947 | CCCCCCC(CCCCCCCCCCC(=O)OCC(COC(=O)C)OC(=O)C)OC(=O)C | 0 | train |
| 144210946 | C=CCC1=C(C=CC(=C1)S(=O)(=O)C2=CC(=C(C=C2)O)CC=C)O | 0 | train |
| 144210945 | C1C2=C(C=C(C=C2)O)C3=CC=CC=C31 | 1 | validation |
| 144210944 | C1=CC(=CC=C1C(=O)N[C@@H](CCC(=O)O)C(=O)O)NCC2=CN=C3C(=N2)C(=NC(=N3)N)N | 0 | test |
| 144210943 | C1=CC=C(C=C1)COC(=O)C2=CC=CC=C2C(=O)O | 1 | train |
| 144210942 | CO/C=C(¥C1=CC=CC=C1COC2=CC=CC(=N2)C(F)(F)F)/C(=O)OC | 1 | train |
| 144210941 | C1(C(C(C(C(C1Cl)Cl)Cl)Cl)Cl)Cl | 0 | validation |
| 144210940 | CC1(CC2=C(O1)C(=CC=C2)OC(=O)NC)C | 1 | test |
| 144210939 | C1=CC=C(C(=C1)C(C2=CC=C(C=C2)Cl)C(Cl)(Cl)Cl)Cl | 1 | train |
| 144210938 | C12C(C(C3C1O3)Cl)C4(C(=C(C2(C4(Cl)Cl)Cl)Cl)Cl)Cl | 1 | train |
| 144210937 | CCC(C)N(C)C(=O)C1=CC2=CC=CC=C2C(=N1)C3=CC=CC=C3Cl | 0 | validation |
| 144210936 | C1CCC(CC1)SSC2CCCCC2 | 0 | test |
| 144210935 | C1=CC=C2C(=C1)C=C(C3=CC=CC=C23)O | 1 | train |
| 144210934 | C1=CC2=C3C(=C1)C=CC4=C(C=CC(=C43)C=C2)O | 1 | train |
| 144210933 | C(=O)(C(C(C(C(C(C(C(F)(F)F)(F)F)(F)F)(F)F)(F)F)(F)F)(F)F)O | 0 | validation |
| 144210932 | CN1[C@@H](CCC1=O)C2=CN=CC=C2 | 0 | test |
| 144210931 | CCCCC(CC)COC(=O)C1=CC(=C(C=C1)C(=O)OCC(CC)CCCC)C(=O)OCC(CC)CCCC | 0 | train |
| 144210930 | C1[C@@H]2[C@@H]3[C@H]([C@H]1[C@H]4[C@@H]2O4)[C@@]5(C(=C([C@]3(C5(Cl)Cl)Cl)Cl)Cl)Cl | 1 | train |
| 144210929 | CC(C)COC(=O)C1=CC=CC=C1C(=O)OCC(C)C | 0 | validation |

| | | | |
|---|---|---|---|
| 144210928 | CN(C)CCOC(C1=CC=CC=C1)C2=CC=CC=C2.Cl | 0 | test |
| 144210927 | C1=CC=C(C=C1)OC2=CC=CC(=C2)C(=O)O | 0 | train |
| 144210926 | C(Cl)C(C(C(C(C(C(F)(F)F)(F)F)(F)F)(F)F)(F)F)(F)F | 0 | train |
| 144210925 | C(=O)(C(C(C(C(C(C(C(C(C(F)(F)F)(F)F)(F)F)(F)F)(F)F)(F)F)(F)F)(F)F)(F)F)O | 0 | validation |
| 144210924 | CC(=C)C(=O)OCCC(C(C(C(C(C(F)(F)F)(F)F)(F)F)(F)F)(F)F)(F)F | 0 | test |
| 144210923 | C(=O)(C(C(C(C(C(C(C(C(C(C(F)(F)F)(F)F)(F)F)(F)F)(F)F)(F)F)(F)F)(F)F)(F)F)(F)F)O | 1 | train |
| 144210922 | CCNC1=CC=CC=C1 | 0 | train |
| 144210921 | C1=CC=C2C(=C1)C(=O)[N-]S2(=O)=O.O.[Na+] | 0 | validation |
| 144210920 | CCCC(CCC)C(=O)O | 0 | test |
| 144210919 | C1C(=O)NC(=O)N1N=CC2=CC=C(O2)[N+](=O)[O-] | 0 | train |
| 144210918 | C[C@@]1([C@H]2[C@@H]([C@H]3[C@@H](C(=O)C(=C([C@]3(C(=O)C2=C(C4=C1C=CC=C4 O)O)O)O)O)C(=O)N)N(C)C)O)O.Cl | 0 | train |
| 144210917 | CCCCC(CC)COC(=O)CCCCCCCC(=O)OCC(CC)CCCC | 0 | validation |
| 144210916 | CC1=CC=C(C=C1)C2=CC(=NN2C3=CC=C(C=C3)S(=O)(=O)N)C(F)(F)F | 0 | test |
| 144210915 | CCCCCCCCCC[N+](C)(C)CCCCCCCCCC.[Cl-] | 1 | train |
| 144210914 | CC(=O)OCCOCCOCCOC(=O)C | 0 | train |
| 144210913 | C1[C@@H]([C@H](O[C@H]1N2C=NC3=C2N=C(N=C3N)Cl)CO)O | 0 | validation |
| 144210912 | C(=O)(C(C(C(C(C(C(C(F)(F)F)(F)F)(F)F)(F)F)(F)F)(F)F)(F)F)[O-].[NH4+] | 0 | test |
| 144210911 | CCOC(=O)C(C1=CC=C(C=C1)Cl)(C2=CC=C(C=C2)Cl)O | 1 | train |
| 144210910 | CCCCCCCCCC(CC)C1=CC=C(C=C1)S(=O)(=O)O.C(CO)N(CCO)CCO | 0 | train |
| 144210909 | C12(C3(C4(C5(C3(C(C1(C5(C2(C4(Cl)Cl)Cl)Cl)Cl)(Cl)Cl)Cl)Cl)Cl)Cl)Cl | 0 | validation |
| 144210908 | CCCCCCCCCCCCCN1CC(OC(C1)C)C | 0 | test |
| 144210907 | CCNC1=NC(=NC(=N1)Cl)NC(C)(C)C | 1 | train |
| 144210906 | CC(=O)O[Hg]C1=CC=CC=C1 | 1 | train |
| 144210905 | C1=C(C=C(C(=C1N)Cl)C(=O)O)Cl | 0 | validation |
| 144210904 | C1=CC2=C3C(=C1)C=CC3=CC=C2 | 1 | test |
| 144210903 | C1[C@H]2[C@@H]([C@H](C1Cl)Cl)[C@@]3(C(=C(C2(C3(Cl)Cl)Cl)Cl)Cl)Cl | 1 | train |
| 144210902 | C1=CC(=CC=C1C2=COC3=CC(=CC(=C3C2=O)O)O)O | 1 | train |
| 144210901 | CCC1=CN=C(C=C1)CCOC2=CC=C(C=C2)CC3C(=O)NC(=O)S3.Cl | 1 | validation |
| 144210900 | CC1=CN(C(=O)NC1=O)[C@H]2C=C[C@H](O2)CO | 0 | test |
| 144210899 | CC(=O)N[C@H]1CCC2=CC(=C(C(=C2C3=CC=C(C(=O)C=C13)OC)OC)OC)OC | 1 | train |
| 144210898 | C1=CC(=C(C=C1F)F)C(CN2C=NC=N2)(CN3C=NC=N3)O | 0 | train |
| 144210897 | C1CC(=O)NC(=O)C1N2C(=O)C3=CC=CC=C3C2=O | 0 | validation |
| 144210896 | C[C@]12CCC(=O)C=C1CC[C@@H]3[C@@H]2CC[C@]4([C@H]3CCC4=O)C | 0 | test |
| 144210895 | CC1=CN(C(=O)NC1=O)[C@H]2C[C@@H]([C@H](O2)CO)N=[N+]=[N-] | 0 | train |
| 144210894 | C1=CN=CC=C1C2=CC=NC=C2 | 0 | train |
| 144210893 | CC(C)CCCCCCC1=CC=C(C=C1)O | 1 | validation |
| 144210892 | C1C[C@@H](O[C@@H]1CO)N2C=NC3=C2NC=NC3=O | 0 | test |
| 144210891 | C1=CC=C(C=C1)N(C2=CC=CC=C2)N=O | 0 | train |
| 144210890 | C1=CC=C2C(=C1)C=CC3=CC=CC=C32 | 0 | train |
| 144210889 | C1C2=CC=CC=C2C3=CC=CC=C31 | 0 | validation |
| 144210888 | CN(C)C1=CC=C(C=C1)C(=C2C=CC(=[N+](C)C)C=C2)C3=CC=C(C=C3)N(C)C.[Cl-] | 1 | test |
| 144210887 | CCC(O)OCCCOC | 0 | train |
| 144210886 | CCN(CC)C1=CC=CC=C1 | 0 | train |
| 144210885 | CC1=CC(=CC=C1)N | 0 | validation |
| 144210884 | CCCC1=CC=CC=C1 | 0 | test |
| 144210883 | CCCCCCCCN1C(=O)C=CS1 | 1 | train |
| 144210882 | CCC(O)OC(CC)O | 0 | train |
| 144210881 | CCCCC(CC)COC(=O)CCCCC(=O)OCC(CC)CCCC | 0 | validation |
| 144210880 | C1=CC(=C(C=C1Cl)Cl)Cl | 0 | test |
| 144210879 | CCCCCCCCCCCCCCCCCC(=O)O | 0 | train |
| 144210878 | CC(C)(C1=CC(=C(C(=C1)Br)O)Br)C2=CC(=C(C(=C2)Br)O)Br | 1 | train |
| 144210877 | C1=CC(=CC=C1C(Cl)(Cl)Cl)Cl | 0 | validation |
| 144210876 | CC(C)CC(C)(C#CC(C)(CC(C)C)O)O | 0 | test |
| 144210875 | C1=CC(=CC=C1N)S(=O)(=O)C2=CC=C(C=C2)N | 0 | train |
| 144210874 | CCCCCCCC(=O)OC | 0 | train |
| 144210873 | C([C@@H]([C@@H]1C(=C(C(=O)O1)O)[O-])O)O.[Na+] | 0 | validation |
| 144210872 | CCCCCCCCCCCC(=O)OC | 0 | test |
| 144210871 | C1=CC(=CC=C1N/N=C¥2/C(=NN(C2=O)C3=CC=C(C=C3)S(=O)(=O)[O-])C(=O)[O- ])S(=O)(=O)[O-].[Na+].[Na+].[Na+] | 0 | train |
| 144210870 | CCCCCCCCCCCCCCCO | 0 | train |
| 144210869 | CC(CN(CCN(CC(C)O)CC(C)O)CC(C)O)O | 0 | validation |

| | | | |
|---|---|---|---|
| 144210868 | C[C@H]1/C=C/C=C(¥C(=O)NC¥2=C(C3=C(C(=C4C(=C3C(=O)/C2=C¥NN5CCN(CC5)C)C(=O)[C@](O4)(O/C=C/[C@@H]([C@H]([C@H]([C@@H]([C@@H]([C@@H]([C@H]1O)C)O)C)OC(=O)C)C)OC)C)C)O)O)/C | 0 | test |
| 144210867 | CCCCCCCCOC(=O)C1=CC=CC=C1C(=O)OCCCCCCCC | 1 | train |
| 144210866 | CCCCCCCCCCCO | 0 | train |
| 144210865 | C[Si]1(O[Si](O[Si](O[Si](O1)(C)C)(C)C)(C)C)C | 0 | validation |
| 144210864 | CCC12COCN1COC2 | 0 | test |
| 144210863 | CCCCCCOCCO | 0 | train |
| 144210862 | C(C(C(C(C(F)(F)S(=O)(=O)[O-])(F)F)(F)F)(F)F)(C(C(C(F)(F)F)(F)F)(F)F)(F)F.[K+] | 0 | train |
| 144210861 | CCCC[Sn](CCCC)(CCCC)Cl | 1 | validation |
| 144210860 | CC(=CCC/C(=C¥CO)/C)C | 0 | test |
| 144210859 | C1=CC=NC(=C1)NS(=O)(=O)C2=CC=C(C=C2)N/N=C¥3/C=CC(=O)C(=C3)C(=O)O | 0 | train |
| 144210858 | CC(C)(C)C1=CC=CC=C1 | 0 | train |
| 144210857 | CC/C(=C(¥C1=CC=CC=C1)/C2=CC=C(C=C2)OCCN(C)C)/C3=CC=CC=C3 | 1 | validation |
| 144210856 | CCCCCCCCCCCCCCO | 0 | test |
| 144210855 | CO[C@H]1[C@@H](C[C@@H]2CN3CCC4=C([C@H]3C[C@@H]2[C@@H]1C(=O)OC)NC5=C4C=CC(=C5)OC)OC(=O)C6=CC(=C(C(=C6)OC)OC)OC | 1 | train |
| 144210854 | CC(C)C(=O)NC1=CC(=C(C=C1)[N+](=O)[O-])C(F)(F)F | 1 | train |
| 144210853 | CCCCCCCCCCCCCCC | 0 | validation |
| 144210852 | C1=CC(C2C1C3(C(=C(C2(C3(Cl)Cl)Cl)Cl)Cl)Cl)Cl | 1 | test |
| 144210851 | CCN(CC)N=O | 0 | train |
| 144210850 | CN1C=CNC1=S | 0 | train |
| 144210849 | CC(=O)CC(C1=CC=CC=C1)C2=C(C3=CC=CC=C3OC2=O)O | 0 | validation |
| 144210848 | CC(C)CCCCCCCOC(=O)C1=CC=CC=C1C(=O)OCCCCCCCC(C)C | 0 | test |
| 144210847 | C1CNP(=O)(OC1)N(CCCl)CCCl.O | 0 | train |
| 144210846 | [As]#[In] | 0 | train |
| 144210845 | C(CO)N(CCO)CCO | 0 | validation |
| 144210844 | CC/C(=C(/CC)¥C1=CC=C(C=C1)O)/C2=CC=C(C=C2)O | 1 | test |
| 144210843 | C1=CC=C(C=C1)C(=O)OCCCCCCOC(=O)C2=CC=CC=C2 | 0 | train |
| 144210842 | C1=CC=C(C=C1)C(=O)OCCCCCOC(=O)C2=CC=CC=C2 | 0 | train |
| 144210841 | CCCCCCCCOC(=O)CCCCCCCCC(=O)OCCCCCCCC | 0 | validation |
| 144210840 | C1=CC=C(C=C1)C(=O)OCCOCCOC(=O)C2=CC=CC=C2 | 0 | test |
| 144210839 | CCCCCCOC(=O)CC(CC(=O)OCCCCCC)(C(=O)OCCCCCC)OC(=O)CCC | 0 | train |
| 144210838 | C=CCC1=CC2=C(C=C1)OCO2 | 0 | train |
| 144210837 | C(CO)C(C(C(C(C(C(F)(F)F)(F)F)(F)F)(F)F)(F)F)(F)F | 0 | validation |
| 144210836 | CS(=O)(=O)NC(=O)C1=C(C=CC(=C1)OC2=C(C=C(C=C2)C(F)(F)F)Cl)[N+](=O)[O-] | 0 | test |
| 144210835 | CN(C)CCCN1C2=CC=CC=C2SC3=C1C=C(C=C3)Cl.Cl | 1 | train |
| 144210834 | CCN(CC1=CC(=CC=C1)S(=O)(=O)[O-])C2=CC=C(C=C2)C(=C3C=CC(=[N+](CC)CC4=CC(=CC=C4)S(=O)(=O)[O-])C=C3)C5=CC=CC=C5S(=O)(=O)[O-].[Na+].[Na+] | 1 | train |
| 144210833 | CC[C@@H]1[C@@]([C@@H]([C@H](C(=O)[C@@H](C[C@@]([C@@H]([C@H]([C@@H]([C@H](C(=O)O1)C)O[C@H]2C[C@@]([C@H]([C@@H](O2)C)O)(C)OC)C)O[C@H]3[C@@H]([C@H](C[C@H](O3)C)N(C)C)O)(C)O)C)C)O)(C)O | 0 | validation |
| 144210832 | C(CCl)OCCCl | 0 | test |
| 144210831 | CCNC1=CC=CC(=C1)C | 0 | train |
| 144210830 | C1=CC=C(C=C1)OC2=CC=C(C=C2)Br | 0 | train |
| 144210829 | CCCCC1=CC=CC=C1 | 0 | validation |
| 144210828 | CCCCOC(=O)C1=CC=CC=C1 | 0 | test |
| 144210827 | COCCOCCOCCOC | 0 | train |
| 144210826 | C1=CC=C(C=C1)[N+](=O)[O-] | 0 | train |
| 144210825 | CC1=CC(=C(C=C1)N)N | 1 | validation |
| 144210824 | CCCCCCCCCC(CC)C1=CC=C(C=C1)S(=O)(=O)[O-].[Na+] | 0 | test |
| 144210823 | C1=C(C(=CC(=C1Cl)Cl)Cl)Cl | 0 | train |
| 144210822 | C1=CC=C(C=C1)OP(=O)(OC2=CC=CC=C2)OC3=CC=CC=C3 | 1 | train |
| 144210821 | CN(C)C(=S)[S-].CN(C)C(=S)[S-].[Zn+2] | 1 | validation |
| 144210820 | C1N2CN3CN1CN(C2)C3 | 0 | test |
| 144210819 | C1=CC(=C(C(=C1)Cl)Cl)Cl | 0 | train |
| 144210818 | CC1=C2C=CC3=CC=CC=C3C2=C(C4=CC=CC=C14)C | 1 | train |
| 144210817 | C1=CC=C2C(=C1)C=CC3=CC4=CC=CC=C4C=C32 | 1 | validation |
| 144210816 | CC(C)(C)CC(C)(C)C1=CC(=C(C=C1)O)N2N=C3C=CC=CC3=N2 | 1 | test |
| 144210815 | CC1=CC(=O)NC(=S)N1 | 0 | train |
| 144210814 | CCCCC(CC)COC(=O)CC(C(=O)OCC(CC)CCCC)S(=O)(=O)[O-].[Na+] | 0 | train |

| | | | |
|---|---|---|---|
| 144210813 | C1=CC=C(C=C1)N=NC2=CC=CC=C2 | 1 | validation |
| 144210812 | CCOC(=O)N | 0 | test |
| 144210811 | CC1=C(C=C(C=C1)[N+](=O)[O-])[N+](=O)[O-] | 0 | train |
| 144210810 | C1=CC=C(C=C1)NNC2=CC=CC=C2 | 1 | train |
| 144210809 | [Cl-].[Cl-].[Hg+2] | 1 | validation |
| 144210808 | C1=CC(=CC(=C1)N)N | 0 | test |
| 144210807 | C1=CC=C2C(=C1)C(=O)NS2 | 1 | train |
| 144210806 | C1=CN(C(=O)N=C1N)[C@H]2[C@H]([C@@H]([C@H](O2)CO)O)O.Cl | 0 | train |
| 144210805 | CC(C)(C)C(CCC1=CC=C(C=C1)Cl)(CN2C=NC=N2)O | 0 | validation |
| 144210804 | CC1=CC(=C(C=C1)C(C)(C)C)O | 0 | test |
| 144210803 | CCCCCCCCCCCCCCCC | 0 | train |
| 144210802 | CCCCCCCCOC1=CC(=C(C=C1)C(=O)C2=CC=CC=C2)O | 0 | train |
| 144210801 | C([C@H]([C@H]([C@@H]([C@@H](CO)O)O)O)O)O | 0 | validation |
| 144210800 | C[C@](CC1=CC(=C(C=C1)O)O)(C(=O)O)N.C[C@](CC1=CC(=C(C=C1)O)O)(C(=O)O)N.O.O.O | 0 | test |
| 144210799 | C1=CC(=CC=C1NC(=O)NC2=CC(=C(C=C2)Cl)Cl)Cl | 1 | train |
| 144210798 | C1=CC=C2C(=C1)C3=CC=CC4=C3C2=CC=C4 | 1 | train |
| 144210797 | CC1=C(C=CC=C1[N+](=O)[O-])[N+](=O)[O-] | 0 | validation |
| 144210796 | COC(=O)NC1=NC2=CC=CC=C2N1 | 1 | test |
| 144210795 | C1=CC(=CC=C1[N+](=O)[O-])[O-].[Na+] | 0 | train |
| 144210794 | CC(C)N(CC1=CC=CC=C1)C(=O)C(C)(C)C | 1 | train |
| 144210793 | C1CN(CCC1(C2=CC=C(C=C2)Cl)O)CCCC(=O)C3=CC=C(C=C3)F | 1 | validation |
| 144210792 | CC(C)OP(=O)(C)OC(C)C | 0 | test |
| 144210791 | CCCCN(CCCC)SN(C)C(=O)OC1=CC=CC2=C1OC(C2)(C)C | 1 | train |
| 144210790 | CCCOC1=C(C(=C(S1)C(=O)N2CCC(CC2)C3=C(C=CC(=C3)CN)F)C)Br.Cl | 1 | train |
| 144210789 | CN1CCN([C@H](C1)C2=CC=C(C=C2)F)CC3=CC(=CO3)C4=CC5=C(C=C4)NC(=O)O5.O.Cl | 0 | validation |
| 144210788 | CC1=C(C(=C2CCC(OC2=C1C)(C)COC3=CC=C(C=C3)CC4C(=O)NC(=O)S4)C)O | 0 | test |
| 144210787 | CC1=C(C2=CC=CC=C2N1CCCCC(=O)O)C3=CN=CC=C3 | 1 | train |
| 144210786 | C1CCC(C1)(C2=CC3=C(C=C2)[C@@H]([C@H](CO3)CC4=CC=C(C=C4)C5=CC=CC=C5)O)C(=O)O | 0 | train |
| 144210785 | CC1=C(N=C(O1)C2=CC=CC=C2)CCOC3=CC=CC4=C3C=CN4CCC(=O)O | 0 | validation |
| 144210784 | CC1=CC(=CC(=C1OC2=C3CCCC3=C(C=C2)O)C)NC(=O)C(=O)O | 0 | test |
| 144210783 | CC1=NOC(=C1)COC2=C(C=C(C=C2)Cl)CNC3=NC=NC4=C3N=CN4[C@H]5[C@@H]([C@@H]([C@H](O5)C(=O)NC)N)O | 0 | train |
| 144210782 | C1=C2C(C(=O)N(C2=CC(=C1F)Cl)C(=O)N)C(=O)C3=CC(=CS3)Cl | 1 | train |
| 144210781 | C1[C@@H](C=C[C@@H]1OC2=CC=CC(=C2)OC3=CC=C(C=C3)F)N(C(=O)N)O | 1 | validation |
| 144210780 | CC(C)(C1=COC(=C1)S(=O)(=O)NC(=O)NC2=C3CCCC3=CC4=C2CCC4)O | 0 | test |
| 144210779 | CCOC1=CC=CC(=C1)C2(CCC(CC2)C)N3CCN(CC3)C4=CC=CC=C4.CS(=O)(=O)O | 0 | train |
| 144210778 | C1[C@H]([C@H](C2=C(O1)C=CC(=C2)OCC3=NC4=C(S3)C=CC(=C4)F)O)CC5=CC=CC(=C5)C(=O)O | 0 | train |
| 144210777 | C1=CC2=C(C=C1Cl)N(C(=O)N(C2=O)CC3=C(C=C(C=C3)Br)F)CC(=O)O | 0 | validation |
| 144210776 | CN[C@H]1CC[C@H](C2=C1C=C(C=C2)S(=O)(=O)N)C3=CC(=C(C=C3)Cl)Cl | 1 | test |
| 144210775 | CC(C)C1=NN=C2N1C=C(C=C2)C3=C(N=CO3)C4=C(C=CC(=C4)F)F | 1 | train |
| 144210774 | C1CCC(CC1)(CC2=NC(=O)ON2)CN.Cl | 0 | train |
| 144210773 | C1CN2C(=O)[C@@H](N=C(C3=CC(=CC1=C32)N)C4=CC=CC=C4)NC(=O)C5=CN=CC=C5 | 0 | validation |
| 144210772 | CC1=C(C=CC(=C1)OC2=C(C=C(C=C2)S(=O)(=O)N)CN(C)C)SC | 0 | test |
| 144210771 | CCCOC1=C(C=C(C=C1)S(=O)(=O)N2CCN(CC2)CC)C3=NC(=O)C4=NN(C(=C4N3)CC)CC5=CC=CC=N5 | 0 | train |
| 144210770 | CN(C[C@@H](CC[N+]12CCC(CC1)(CC2)C3CCCCC3)C4=CC(=C(C=C4)Cl)Cl)C(=O)C5=CC(=CC(=C5)C(F)(F)F)C(F)(F)F.CS(=O)(=O)[O-] | 1 | train |
| 144210769 | COCC1=NN=C(N1C2=C3C(=CC(=C2Cl)Cl)NC(=O)C(=O)N3)C4=C[N+](=CC=C4)[O-] | 0 | validation |
| 144210768 | COCCOC[C@H](CC1(CCCC1)C(=O)NC2CCC(CC2)C(=O)O)C(=O)OC3=CC4=C(CCC4)C=C3 | 0 | test |
| 144210767 | C1CN2CCC1[C@H](C2)NC(=O)C3=CC4=C(C=C3)OCCO4 | 0 | train |
| 144210766 | C1CN2CCC1[C@H](C2)NC(=O)C3=NC=C4C(=C3)C=CO4 | 0 | train |
| 144210765 | CC[C@H]1CC[C@@H]([C@@H](N1)C2=CC=CC=C2)NCC3=C(C=C4[C@H]5C[C@H]5C(=O)N(C4=C3)C)OC | 0 | validation |
| 144210764 | C[C@H](C(=O)O)OC1=CC(=C(C=C1)CNC(=O)C2=C(N=CC=C2)OC3=CC4=C(C=C3)OCO4)F | 0 | test |
| 144210763 | CC(C)(C1=CC=C(C=C1)CNC(=O)C2=C(N=CC=C2)OC3=CC4=NON=C4C=C3)O | 1 | train |
| 144210762 | CNCC1=C(C=CC(=C1)F)OC2=CC(=C(C=C2)Cl)Cl | 1 | train |
| 144210761 | CC[C@@H]1C[C@@H](C2=C(N1C(=O)OC(C)C)C=CC(=C2)C(F)(F)F)N(CC3=CC(=CC(=C3)C(F)(F)F)C(F)(F)F)C(=O)C | 0 | validation |

| | | | |
|---|---|---|---|
| 144210760 | CC(C)(C1=CC=C(C=C1)CNC(=O)C2=C(N=CC=C2)OC3=CC=C(C=C3)F)O | 1 | test |
| 144210759 | CN1CCN(CC1)C2=CC=CC=C2/C=C¥3/C(=O)N(CCS3)C4=CC(=C(C=C4)Cl)Cl | 1 | train |
| 144210758 | CNCC1=CC=C(C=C1)NC(=O)C2=CNC3=C2C(=O)CCC3 | 1 | train |
| 144210757 | CC(CC1=CC=C(C=C1)OCC(=O)O)NCC(C2=CSC(=N2)C(F)(F)F)O | 0 | validation |
| 144210756 | CC1=NC=CN1C2=CC=C(C=C2)SC3=CC=CC(=C3)C4(CCOCC4)C(=O)N.CS(=O)(=O)O | 1 | test |
| 144210755 | CC1=C2C(=CC(=C1)CCN3CCN(CC3)C4=NSC5=CC=CC=C54)C(CC(=O)N2)(C)C.CS(=O)(=O)O | 1 | train |
| 144210754 | C1CN(CCC1CC2=CC=C(C=C2)F)CCS(=O)C3=CC4=C(C=C3)NC(=O)O4 | 0 | train |
| 144210753 | CC(C)OC1=C(SC2=C1C=C(C=C2)OC)C(=O)NC3=NN=N[N-]3.[Na+] | 1 | validation |
| 144210752 | C[C@@H]1CN(C[C@@H](N1C2=NC(=NC=C2)[C@@H](C)O)C)C3=NC(=NC(=N3)C)C | 0 | test |
| 144210751 | C1COCCC1(C(=O)NO)NS(=O)(=O)C2=CC=C(C=C2)OC3=CC=C(C=C3)F | 0 | train |
| 144210750 | C1CCC(C1)(C(=O)NO)N(CCC(=O)O)S(=O)(=O)C2=CC=C(C=C2)OC3=CC=C(C=C3)F | 0 | train |
| 144210749 | C1CN(CCC1(C2=CC=C(C=C2)F)O)[C@@H]3COC4=C([C@@H]3O)C=CC(=C4)O | 1 | validation |
| 144210748 | CCN1C2=C(C(=N1)C)NC(=C3C=CC(=O)C=C3)CNC2=O | 0 | test |
| 144210747 | CC1=CC(=C(C=C1CO)C(C)(C)C)SC2=C(C[C@@](OC2=O)(CCC3=CC=C(C=C3)N)C(C)C)O | 0 | train |
| 144210746 | CC[C@H](C)[C@@H](C(=O)O)N1C(=O)C2=CC=CC=C2S1 | 0 | train |
| 144210745 | C1CCC2=NC3=CC(=CC(=C3CN2CC1)Cl)Cl.Cl | 1 | validation |
| 144210744 | CN([C@H]1CC[C@@]2(CCCO2)C[C@@H]1N3CCCC3)C(=O)CC4=C5C=COC5=CC=C4 | 0 | test |
| 144210743 | CCCCN(CCCC)N=O | 0 | train |
| 144210742 | CCOP(=S)(C1=CC=CC=C1)OC2=CC=C(C=C2)[N+](=O)[O-] | 1 | train |
| 144210741 | CC1=C(S(=O)(=O)CCS1(=O)=O)C | 1 | validation |
| 144210740 | CCCN(CCC)C1=C(C=C(C=C1[N+](=O)[O-])C(C)C)[N+](=O)[O-] | 1 | test |
| 144210739 | CCC(C)C1=CC(=CC(=C1O)[N+](=O)[O-])[N+](=O)[O-] | 0 | train |
| 144210738 | C1=CC=C(C=C1)NC(=O)C2=CC=CC=C2I | 1 | train |
| 144210737 | C(C(=O)O)(Cl)Cl | 0 | validation |
| 144210736 | C1=CC=C(C(=C1)[N+](=O)[O-])[N+](=O)[O-] | 1 | test |
| 144210735 | CCO[Si](CCCNC(=O)N)(OCC)OCC | 0 | train |
| 144210734 | CC1=CC=C(C=C1)S(=O)(=O)NC(=O)NN2CCCCCC2 | 0 | train |
| 144210733 | C1=CC=C(C=C1)C(C2=CC=CC=C2)(C3=CC=CC=C3Cl)N4C=CN=C4 | 1 | validation |
| 144210732 | CC1CC(OC(O1)C)OC(=O)C | 0 | test |
| 144210731 | C[C@]12CC[C@H]3[C@H]([C@@H]1CC[C@]2(C#C)O)CCC4=C3C=CC(=C4)O | 0 | train |
| 144210730 | C(C(C(C(C(F)(F)S(=O)(=O)N)(F)F)(F)F)(F)F)(C(C(C(F)(F)F)(F)F)(F)F)(F)F | 0 | train |
| 144210729 | C1=CC(=C(C(=C1)F)C(=O)NC(=O)NC2=CC(=C(C(=C2)Cl)OC(C(F)F)(F)F)Cl)F | 1 | validation |
| 144210728 | C([C@@H]1[C@H]([C@@H]([C@H]([C@H](O1)O[C@]2([C@H]([C@@H]([C@H](O2)CO)O)O)CO)O)O)O)O | 0 | test |
| 144210727 | CCCC[Sn](CCCC)(CCCC)OC(=O)C(=C)C | 1 | train |
| 144210726 | CC(C)(C=NO)SC | 0 | train |
| 144210725 | C1=CC(=CC=C1N/N=C¥2/C3=C(C=CC2=O)C=C(C=C3)S(=O)(=O)[O-])S(=O)(=O)[O-].[Na+].[Na+] | 0 | validation |
| 144210724 | CCCCCC(=O)O | 0 | test |
| 144210723 | C1=CC(=CC=C1C2=CC=C(C=C2)N)N | 1 | train |
| 144210722 | C1=CC=C(C(=C1)C=O)C=O | 1 | train |
| 144210721 | CCN(CC)C(=S)SSC(=S)N(CC)CC | 1 | validation |
| 144210720 | CC(C1=CC=C(C=C1)O)(C2=CC=C(C=C2)O)C3=CC=C(C=C3)O | 0 | test |
| 144210719 | C1/C=C/CC/C=C/CC/C=C/C1 | 0 | train |
| 144210718 | CCC(C)(C)C(=O)O[C@H]1C[C@H](C=C2[C@H]1[C@H]([C@H](C=C2)C)CC[C@@H]3C[C@H](CC(=O)O3)O)C | 0 | train |
| 144210717 | CC(C)CCCCCOC(=O)C=C | 0 | validation |
| 144210716 | CCOP(=S)(OCC)SCSCC | 0 | test |
| 144210715 | C(CO)C(C(C(C(C(C(C(C(F)(F)F)(F)F)(F)F)(F)F)(F)F)(F)F)(F)F)(F)F | 0 | train |
| 144210714 | C(C(C(C(F)(F)S(=O)(=O)[O-])(F)F)(F)F)(C(C(F)(F)F)(F)F)(F)F.[K+] | 0 | train |
| 144210713 | CCCCC1=C(C2=CC=CC=C2O1)C(=O)C3=CC(=C(C(=C3)I)OCCN(CC)CC)I.Cl | 1 | validation |
| 144210712 | C(=O)(C(C(C(C(C(F)(F)F)(F)F)(F)F)(F)F)(F)F)O | 0 | test |
| 144210711 | CC1=CC(=C(C=C1S(=O)(=O)[O-])OC)N/N=C¥2/C3=C(C=CC2=O)C=C(C=C3)S(=O)(=O)[O-].[Na+].[Na+] | 0 | train |
| 144210710 | CN(C)CCCN(C)CCCN(C)C | 0 | train |
| 144210709 | CN(C)C(=S)[S-].[Na+] | 1 | validation |
| 144210708 | CCC1=CC(=C(C=C1)O)C(C)(C)C | 0 | test |
| 144210707 | CCCCCCCC/C=C¥CCCCCCCC(=O)OC[C@H]([C@@H]1[C@@H]([C@H](CO1)O)O)O | 0 | train |
| 144210706 | CCCN(CCC)N=O | 0 | train |
| 144210705 | CC1=C(C=C(C=C1)[N+](=O)[O-])C | 0 | validation |

| | | | |
|---|---|---|---|
| 144210704 | CC(=O)OC[C@@H]1[C@H]([C@@H]([C@H]([C@H](O1)O[C@]2([C@H]([C@@H]([C@H](O2)COC(=O)C)OC(=O)C)OC(=O)C)COC(=O)C)OC(=O)C)OC(=O)C)OC(=O)C | 0 | test |
| 144210703 | CC(C)(C)C1=CC(=C(C=C1O)C(C)(C)C)O | 0 | train |
| 144210702 | CCCC(=O)OCCC(C)C | 0 | train |
| 144210701 | C=CCCCCCCCCC(=O)O | 0 | validation |
| 144210700 | CCCCCCC(=O)OCC | 0 | test |
| 144210699 | CCCCCCCCCCCCCCCCCOS(=O)(=O)[O-].[Na+] | 0 | train |
| 144210698 | C(=O)(C(C(C(C(C(C(C(C(F)(F)F)(F)F)(F)F)(F)F)(F)F)(F)F)(F)F)(F)F)(F)F)O | 0 | train |
| 144210697 | COC(=O)C1=C(C(=C(C(=C1Cl)Cl)C(=O)OC)Cl)Cl | 1 | validation |
| 144210696 | CCOC1=CC=C(C=C1)C(C)(C)COCC2=CC(=CC=C2)OC3=CC=CC=C3 | 1 | test |
| 144210695 | CCNS(=O)(=O)C(C(C(C(C(C(C(C(F)(F)F)(F)F)(F)F)(F)F)(F)F)(F)F)(F)F)(F)F | 0 | train |
| 144210694 | CCCCCCCCCCCCCCCOS(=O)(=O)[O-].[Na+] | 0 | train |
| 144210693 | COC(=O)CC(=O)OC | 0 | validation |
| 144210692 | CCCCCCCCCCCCN1CCCC1=O | 1 | test |
| 144210691 | CC(C)CCOC(=O)C | 0 | train |
| 144210690 | C(C(C(=O)O)O)C(=O)O | 0 | train |
| 144210689 | C1(=C(C(=NC(=C1Cl)Cl)Cl)Cl)Cl | 0 | validation |
| 144210688 | CC(CO)OCC(C)OCC(C)OC | 0 | test |
| 144210687 | CCOCCOCCOCCO | 0 | train |
| 144210686 | CCCCCCCCN(C)CCCCCCCC | 0 | train |
| 144210685 | CN(C)C(=O)NC1=CC=C(C=C1)Cl | 0 | validation |
| 144210684 | CCCCCCCCCC(=O)O | 0 | test |
| 144210683 | CC1=NC=C(C(=C1O)CO)CO | 0 | train |
| 144210682 | CC(C(=O)O)(Cl)Cl | 0 | train |
| 144210681 | COC(=O)CCCCC(=O)OC | 0 | validation |
| 144210680 | CC(C)C1=CC(=CC=C1)C(C)C | 0 | test |
| 144210679 | CC1(C(=O)NC(=O)N1)C | 0 | train |
| 144210678 | CCCCCOC(=O)C | 0 | train |
| 144210677 | C1=CC=C2C(=C1)C(=CN2)C[C@@H](C(=O)O)N | 0 | validation |
| 144210676 | CC1=CC(=C(C=C1)C(C)C)O | 0 | test |
| 144210675 | CCCCCCC1=C(C=C(C=C1)O)O | 0 | train |
| 144210674 | CCCCCCCCCCCC[N+](C)(C)C.[Cl-] | 0 | train |
| 144210673 | CC(=CCC/C(=C/COC(=O)C)/C)C | 0 | validation |
| 144210672 | C1=CC(=CN=C1)C#N | 0 | test |
| 144210671 | CC(C)COC(=O)CCCCC(=O)OCC(C)C | 0 | train |
| 144210670 | COC1=C(C=CC(=C1)C=O)O | 0 | train |
| 144210669 | CC(=CCCC(=C)C=C)C | 0 | validation |
| 144210668 | COC(=O)CCC(=O)OC | 0 | test |
| 144210667 | CCCCCCCCN(C)C | 0 | train |
| 144210666 | CC1=CC(=C(C(=C1)C)O)C | 0 | train |
| 144210665 | COC(=O)C1=CC=CC=C1N | 0 | validation |
| 144210664 | CC1=CC=C(C=C1)Cl | 0 | test |
| 144210663 | CCCCCCCC(=O)O | 0 | train |
| 144210662 | C(CCC(=O)O)CC(=O)O | 0 | train |
| 144210661 | CC(=O)C1=CC=CC=C1 | 0 | validation |
| 144210660 | CCCCCCC(=O)O | 0 | test |
| 144210659 | CC1=CC(=CC(=C1)C)C | 0 | train |
| 144210658 | C1=CC=C(C(=C1)O)Cl | 0 | train |
| 144210657 | CC1=CC=CC2=CC=CC=C12 | 0 | validation |
| 144210656 | CC1=CC=C(C=C1)C(C)C | 0 | test |
| 144210655 | CCCCCCCC=O | 0 | train |
| 144210654 | CCCCCCCCCC | 0 | train |
| 144210653 | CCOC(=O)C1=CC=C(C=C1)O | 1 | validation |
| 144210652 | CC1=C(C(=CC=C1)[N+](=O)[O-])C | 0 | test |
| 144210651 | CCC1=C(C(=CC=C1)CC)N | 0 | train |
| 144210650 | CCCCOC(=O)C1=CC=C(C=C1)O | 1 | train |
| 144210649 | CCCCCCCCCCCCCCCS(=O)(=O)OC1=CC=CC=C1 | 0 | validation |
| 144210648 | CCCCCCCCCCCC(=O)OCC(C)OC(=O)CCCCC(=O)O | 0 | test |
| 144210647 | CC1=CC=CC=C1Cl | 0 | train |
| 144210646 | CCOC1=CC2=C(C=C1)NC(C=C2C)(C)C | 1 | train |
| 144210645 | CC1=CC(=CC(=C1O)[N+](=O)[O-])[N+](=O)[O-] | 0 | validation |
| 144210644 | CC1=CC(=C(C=C1)C)O | 0 | test |

| | | | |
|---|---|---|---|
| 144210643 | CCCCCCCCCCCCCCCCO | 0 | train |
| 144210642 | CCCCC(CC)COC(=O)CCCCC(=O)OCC1=CC=CC=C1 | 0 | train |
| 144210641 | CCCCC(CC)COC(=O)C1=CC=C(C=C1)C(=O)OCC(CC)CCCC | 0 | validation |
| 144210640 | CCCCC(CC)COP(=O)(OCC(CC)CCCC)OCC(CC)CCCC | 0 | test |
| 144210639 | CC1=CC2=C(C=C1)N=CC=C2 | 0 | train |
| 144210638 | C(=O)(N)NO | 0 | train |
| 144210637 | CS(=O)(=O)OCCCCOS(=O)(=O)C | 0 | validation |
| 144210636 | CC(C)(C1=CC=CC=C1)C2=CC(=C(C=C2)O)C(C)(C)C3=CC=CC=C3 | 1 | test |
| 144210635 | CC(C)C(C(C)(C)COC(=O)C(C)C)OC(=O)C(C)C | 0 | train |
| 144210634 | COC1=C(C=CC(=C1)C2=CC(=C(C=C2)N)OC)N.Cl.Cl | 1 | train |
| 144210633 | C(CNC(=S)[S-])NC(=S)[S-].C(CNC(=S)[S-])NC(=S)[S-].[Mn+2].[Zn+2] | 1 | validation |
| 144210632 | C1=CC(=CC=C1C(=N)N)OCCCCCOC2=CC=C(C=C2)C(=N)N.C(CS(=O)(=O)O)O.C(CS(=O)(=O)O)O | 0 | test |
| 144210631 | C1CC2C(O2)CC1C3CO3 | 0 | train |
| 144210630 | CC(C)CCCCCCCOC(=O)CCCCC(=O)OCCCCCCCC(C)C | 0 | train |
| 144210629 | CCCCC(CC)C(=O)OCCOCCOCCOC(=O)C(CC)CCCC | 0 | validation |
| 144210628 | CC(C)NCC(C1=CC(=C(C=C1)O)O)O.Cl | 0 | test |
| 144210627 | CC(=O)[C@H]1CC[C@@H]2[C@@]1(CC[C@H]3[C@H]2CCC4=CC(=O)CC[C@]34C)C | 1 | train |
| 144210626 | C(COCCOCCO)O | 0 | train |
| 144210625 | CC1=C(C(CCC1)(C)C)/C=C/C(=C/C=C/C(=C/C(=O)O)/C)/C | 1 | validation |
| 144210624 | CCCCCOC(=O)C1=CC=CC=C1C(=O)OCCCCC | 1 | test |
| 144210623 | CC=CC1=CC(=C(C=C1)O)OC | 0 | train |
| 144210622 | C([C@@H]1[C@@H]2[C@@H]([C@H]([C@H](O1)O[C@@H]3[C@H](O[C@@H]([C@@H]([C@H]3O)O)O[C@@H]4[C@H](O[C@@H]([C@@H]([C@H]4O)O)O[C@@H]5[C@H](O[C@@H]([C@@H]([C@H]5O)O)O[C@@H]6[C@H](O[C@@H]([C@@H]([C@H]6O)O)O[C@@H]7[C@H](O[C@H](O2)[C@@H]([C@H]7O)O)CO)CO)CO)CO)CO)O)O)O | 0 | train |
| 144210621 | C1C(O1)CO | 0 | validation |
| 144210620 | CC1=CC=CC=C1N=NC2=CC(=C(C=C2)N)C | 1 | test |
| 144210619 | COS(=O)(=O)C | 0 | train |
| 144210618 | CC1(CC(CC(C1)(C)CN=C=O)N=C=O)C | 0 | train |
| 144210617 | C1=CC=C(C=C1)N/N=C/2¥C(=O)C=CC3=CC=CC=C32 | 1 | validation |
| 144210616 | C(C(CO)(CBr)CBr)O | 0 | test |
| 144210615 | C(C(CO)(CO)[N+](=O)[O-])O | 0 | train |
| 144210614 | CC1=C2C(=CC(=C1C(=O)O)O)C(=O)C3=C(C2=O)C(=C(C(=C3O)O)[C@H]4[C@@H]([C@H]([C@@H]([C@H](O4)CO)O)O)O)O | 0 | train |
| 144210613 | C1=CC(=C(C=C1[N+](=O)[O-])Cl)Cl | 0 | validation |
| 144210612 | C1NC2=CC(=C(C=C2S(=O)(=O)N1)S(=O)(=O)N)Cl | 0 | test |
| 144210611 | CC(C)CCCCCCOC(=O)CCCCC(=O)OCCCCCCC(C)C | 0 | train |
| 144210610 | C1C2=C(C(=CC=C2)O)C(=O)C3=C1C=CC=C3O | 0 | train |
| 144210609 | C1=CC=C2C3=C4C(=CC=C3)C5=CC=CC=C5C4=CC2=C1 | 1 | validation |
| 144210608 | C[C@]12CC[C@H]3[C@H]([C@@H]1CC[C@@H]2O)CCC4=C3C=CC(=C4)O | 1 | test |
| 144210607 | CC1=C(C(CCC1)(C)C)/C=C/C(=C/C=C/C(=C/CO)/C)/C | 1 | train |
| 144210606 | CCOC(=O)CCC(=O)OCC | 0 | train |
| 144210605 | C1=CC(=C(C=C1C2=C(C(=O)C3=C(C=C(C=C3O2)O)O)O)O)O | 1 | validation |
| 144210604 | C(C(CCl)O)Cl | 0 | test |
| 144210603 | CC(C)(C1=CC=CC=C1)OO | 0 | train |
| 144210602 | C1=CC=C2C(=C1)C(=O)OC2(C3=CC=C(C=C3)O)C4=CC=C(C=C4)O | 1 | train |
| 144210601 | C1=CC=C(C=C1)C2(C(=O)NC(=O)N2)C3=CC=CC=C3 | 1 | validation |
| 144210600 | CCCC1=CC(=O)NC(=S)N1 | 0 | test |
| 144210599 | CN(C)C1=CC=C(C=C1)C(=O)C2=CC=C(C=C2)N(C)C | 1 | train |
| 144210598 | CC1=C(C=CC(=C1)C2=CC(=C(C=C2)N)C)N | 1 | train |
| 144210597 | CCOC(=O)C(C)(C)OC1=CC=C(C=C1)Cl | 0 | validation |
| 144210596 | C1=CC(=CC=C1[N+](=O)[O-])OC2=C(C=C(C=C2)Cl)Cl | 0 | test |
| 144210595 | C1=NC2=C(N1)C(=S)N=C(N2)N | 0 | train |
| 144210594 | CC/C(=C(¥C1=CC=CC=C1)/C2=CC=C(C=C2)OCCN(C)C)/C3=CC=CC=C3.C(C(=O)O)C(CC(=O)O)(C(=O)O)O | 1 | train |
| 144210593 | C1C2C=CC1C3C2C(=O)OC3=O | 0 | validation |
| 144210592 | CCCCCCCCOC(=O)C1=CC(=C(C(=C1)O)O)O | 0 | test |
| 144210591 | CCC1(C(=O)NC(=NC1=O)[O-])C2=CC=CC=C2.[Na+] | 0 | train |
| 144210590 | CC[C@H](C)C(=O)O[C@H]1C[C@@H](C=C2[C@H]1[C@H]([C@H](C=C2)C)CC[C@H](C[C@H](CC(=O)[O-])O)O)O.[Na+] | 0 | train |

| | | | |
|---|---|---|---|
| 144210589 | CN(CC1=CN=C2C(=N1)C(=NC(=N2)N)N)C3=CC=C(C=C3)C(=O)N[C@@H](CCC(=O)O)C(=O)O | 0 | validation |
| 144210588 | CC[C@H](C)C(=O)O[C@H]1C[C@H](C=C2[C@H]1[C@H]([C@H](C=C2)C)CC[C@@H]3C[C@H](CC(=O)O3)O)C | 0 | test |
| 144210587 | CC(=O)N1CCN(CC1)C2=CC=C(C=C2)OC[C@H]3CO[C@](O3)(CN4C=CN=C4)C5=C(C=C(C=C5)Cl)Cl | 1 | train |
| 144210586 | CC1=C(C2=C(N1C(=O)C3=CC=C(C=C3)Cl)C=CC(=C2)OC)CC(=O)O | 0 | train |
| 144210585 | C1=CC=C(C(=C1)CC(=O)[O-])NC2=C(C=CC=C2Cl)Cl.[Na+] | 0 | validation |
| 144210584 | CCOC1=CC=CC(=C1)N2C=C(N=C2C3=CC=C(C=C3)C)C(=O)N4CCN(CC4)C5=CC6=CC=CC=C6C(=C5)C(=O)[O-].[Na+] | 0 | test |
| 144210583 | CC(C)[C@@]1(CC[C@H](C1)[NH2+][C@H]2CCOC[C@H]2OC)C(=O)N3CCC4=NC=C(C=C4C3)C(F)(F)F.C(CC(=O)[O-])C(=O)O | 0 | train |
| 144210582 | C1CC(CCC1NC2=NC=NC3=C2C=NN3)OC[C@@H](C4=CC=CC=C4F)F | 0 | train |
| 144210581 | CC1=NC=C(C=N1)[C@@H](CCCCCCC2=NC3=C(CCCN3)C=C2)CC(=O)O | 0 | validation |
| 144210580 | CC1=CN(C2=C1C=C(C=C2)F)NC(=O)C3=CN=C(N=C3C)C4=CC=CC=N4 | 1 | test |
| 144210579 | CCOC(=O)C1=CC=C(C=C1)C2CCC(CC2)NC[C@@H](COC3=CC(=C(C=C3)O)NS(=O)(=O)C)O.Cl | 0 | train |
| 144210578 | CC(=O)NC1(CCN(CC1)CC[C@]2(CN(C(=O)CO2)C3=CC=CC=C3)C4=CC(=C(C=C4)Cl)Cl)C5=CC(=CC=C5)F.C(CC(=O)O)C(=O)O | 1 | train |
| 144210577 | CC(C)N1CCC(CC1)NC(=O)C2=CC3=CC(=NC=C3N2CC(=O)NC4=NC=C(C=C4)Cl)OCCOC.CC(=O)O | 0 | validation |
| 144210576 | C1CC1N2CCC(CC2)NC(=O)C3=NC4=C(N3CC5=NOC(=C5)C6=CC=C(S6)Cl)C=CC(=C4)C(=O)[O-].[Na+] | 0 | test |
| 144210575 | CCN(CC1=CN=CC=C1)C(=O)C2=C(C(=CC=C2)Cl)[C@]3(C4=C(C=CC(=C4)Cl)N(C3=O)CC5=C(C=C(C=C5)OC)OC)C.Cl | 1 | train |
| 144210574 | CCOC(=O)O[C@H](C)OC(=O)C1=CC2=C(C=C1)N(C(=C2)C(=O)NC3CCN(CC3)C(C)C)CC4=NOC(=C4)C5=CC=C(S5)Cl.Cl | 1 | train |
| 144210573 | C1CN(CCC1C(=O)O)C2=C(C=C(C=C2)F)NC(=O)NC(=O)C3=CC(=C(C=C3Cl)F)F | 0 | validation |
| 144210572 | C1=CC=C(C(=C1)C(=O)O)N2C=C(C(=O)C3=CC(=C(C=C32)Cl)NC4=C(C=C(C=C4Cl)F)F)C(=O)O | 0 | test |
| 144210571 | COC1=CC=C(C=C1)C(=O)NC2=NC3=CC=CC=C3C(=C2C#N)NCC4=CC=CC=C4 | 1 | train |
| 144210570 | CC[C@@H](C1=CC=CC=C1)NC(=O)C2=C(C=CC(=C2)F)[N-]S(=O)(=O)C3=CC=CC4=C3N=CC=C4.[Na+] | 0 | train |
| 144210569 | C1CN(CCC1C2=CC(=CC=C2)CN)C(=O)C3=CC=C(O3)C#CC4=CC=CC=C4F | 0 | validation |
| 144210568 | CC1=CC(=C2C(=C1)C=C(N2CC(=O)O)C(=O)NC3=NC(=C(S3)CCC4CCCCC4)C5=CC(=C(C=C5OC)Cl)OC)C.C(=O)(C(F)(F)F)O | 0 | test |
| 144210567 | CC(C)C1=CC(=CC2=C1C(=O)N(S2(=O)=O)COC3=CC(=O)N4C=CC=C(C4=N3)OCCN5CCCCC5)OC | 0 | train |
| 144210566 | C1=CC(=CC(=C1)F)CN2C3=C(C=C(C=C3)F)C=C2C(=O)NC4=CC5=C(C=C4)NC=C5 | 1 | train |
| 144210565 | C1CN2CCC1[C@@H](C2)[C@@H](C3=CC=CC=C3)NC(=O)C4=C(C=CC(=C4Cl)C(F)(F)F)Cl.Cl | 1 | validation |
| 144210564 | COC1=CC=CC(=C1OC)[C@@H](C2CCN(CC2)CCC3=CC=C(C=C3)F)O | 0 | test |
| 144210563 | CCOC(=O)[C@]1([C@H](C2=CC=CC=C2O1)N)C3=CC=CC=C3 | 0 | train |
| 144210562 | CCOC(=O)COC1=CC2=C(CCC(C2)NCC(C3=CC(=CC=C3)Cl)O)C=C1.Cl | 0 | train |
| 144210561 | C1CN2CCC1N(CC2)C(=O)OC3=CC=C(C=C3)Br | 0 | validation |
| 144210560 | CN(C)C(=O)[C@@H]1C[C@H](CN1[C@]2(C3=C(C=CC(=C3)Cl)N(C2=O)S(=O)(=O)C4=C(C=C(C=C4)OC)OC)C5=CC=CC=C5OC)O | 1 | test |
| 144210559 | CCC1=C(N(N=C1C(=O)NN2CCCCC2)C3=C(C=C(C=C3)Cl)Cl)C4=CC=C(C=C4)Br | 0 | train |
| 144210558 | CC(C)[C@@H](C(=O)N[C@H]1CC2=CC=CC=C2[C@H]3CCC[C@H](N3C1=O)C(=O)O)SC(=O)C | 0 | train |
| 144210557 | C1CN(CCN1CCC2=CC(=O)N(C3=C2C=CC(=C3)F)CC(=O)N)C4=NC=CC5=C4C=CS5 | 0 | validation |
| 144210556 | CN(C)C(=O)C1(CCN(CC1)CCC2(CN(CCO2)C(=O)C3=CC=CC=C3)C4=CC(=C(C=C4)Cl)Cl)N5CCCCC5.Cl | 1 | test |
| 144210555 | C[C@@H]1CCC[C@@H](N1CC2=CC=C(C=C2)C[C@H](C(=O)N(C)C(C)C)NC(=O)C[C@H](C3=CC4=C(C=C3)OCO4)NS(=O)(=O)C5=CC6=C(C=C5)C=C(C=C6)OC)C.Cl | 1 | train |
| 144210554 | C1CCN[C@@H](C1)[C@H](C2=CC=CC=C2)NC(=O)C3=C(C(=CC=C3)C(F)(F)F)Cl | 1 | train |
| 144210553 | CCN(CC)CCNC1=C2C(=C(C=C1)CNC=O)SC3=C(C2=O)C=C(C=C3)OC | 1 | validation |
| 144210552 | C1CC2CC(CC1N2C3=NC=CC=N3)NCC(=O)N4CC(C[C@H]4C#N)(F)F | 0 | test |
| 144210551 | CC(C)C1=C(C=C(C=C1)C(=O)N=C(N)N)S(=O)(=O)C.CS(=O)(=O)O | 0 | train |
| 144210550 | COC1=CC=CC(=C1)C2=C(C=C(C=C2)/C=C¥CN3CCCCCC3)Cl.Cl | 0 | train |
| 144210549 | CN(C)C(=O)NC1(CCN(CC1)CCC[C@]2(CCCN(C2)C(=O)C3=CC=CC=C3)C4=CC(=C(C=C4)Cl)Cl)C5=CC=CC=C5 | 0 | validation |

| | | | |
|---|---|---|---|
| 144210548 | CN(C)C(=O)NC1(CCN(CC1)CC[C@]2(CN(CCO2)C(=O)C3=CC=CC=C3)C4=CC(=C(C=C4)F)F)C5=CC=CC=C5.Cl | 0 | test |
| 144210547 | C1C2C(C3=C1C=C(C=C3)Cl)(N=C(S2)C4=CC=CC=C4)O | 1 | train |
| 144210546 | CCCCC1=NC2=C(C=C1)[C@@H]([C@H]([C@@H]2C3=C(C=C(C=C3)OC)C[C@H](C)C(=O)[O-])C(=O)[O-])C4=CC5=C(C=C4)OCO5.[Na+].[Na+] | 0 | train |
| 144210545 | CC(C)(C1=NC2=NC=C(N2N=C1)C3=CC(=C(C=C3)F)C4=C(C=CC=C4F)C#N)O | 0 | validation |
| 144210544 | C1=CC(=CC(=C1)C2=N[N-]C(=N2)C(=O)N)C3=C(C=CC(=C3)F)OCC(C(F)(F)F)(F)F.O.[K+] | 0 | test |
| 144210543 | CCN1C(=CC(=N1)CC2=CC=CC=C2)C3CCN(CC3)C[C@H]4C[C@@H](C[C@@H]4C5=CC(=CC=C5)F)N(C)[C@H](C(C)C)C(=O)O | 0 | train |
| 144210542 | CCC1=C(C=CC(=C1)SCC2=C(N=C(S2)C3=CC=C(C=C3)C(F)(F)F)CN4CCN(CC4)C5=CC=C(C=C5)OC)OCC(=O)O | 0 | train |
| 144210541 | C1=CC=C(C=C1)OC2=NC=CC(=N2)C3=C(N=CN3C(CO)CO)C4=CC=C(C=C4)F | 0 | validation |
| 144210540 | C1=CC(=C(C=C1N(CC(=O)N)CC(F)(F)F)C(F)(F)F)C#N | 0 | test |
| 144210539 | CCN(C(C)C)C(=O)C1=CC(=CC(=C1)OC[C@H](C)NC2=CC=NC=C2)C.CC1=CC=C(C=C1)S(=O)(=O)O | 0 | train |
| 144210538 | CC1=C(N=C(O1)C2=CC=CC=C2)CCOC3=CC=C(C=C3)C[C@@H](C(=O)O)NC4=CC=CC=C4C(=O)C5=CC=CC=C5 | 0 | train |
| 144210537 | C1[C@H](C2=C(O1)C=C(C=C2)OCC3=CC=CC=C3)N(C(=O)N)O | 1 | validation |
| 144210536 | CC1=C(C=C2C(=C1)CCN2C(=O)NC3=CN=C(C=C3)OC4=C(N=CC=C4)C)C(F)(F)F.Cl | 0 | test |
| 144210535 | CC1=NC2=CC=CC=C2N1C3CC4CCC(C3)N4CCC5(CCN(CC5)C(=O)C6=CC(=C(C=C6Cl)F)S(=O)(=O)NC)C7=CC(=CC=C7)F | 0 | train |
| 144210534 | CC1=NC(=NO1)C2=CC3=C(CCN(CC3)CCC4CCC(CC4)NC(=O)/C=C/C5=CC=C(C=C5)F)C=C2.Cl | 0 | train |
| 144210533 | CC(=O)C1=CC2=C(C=C1)OC([C@@H]([C@H]2NC(=O)C3=CC=C(C=C3)F)O)(C)C | 0 | validation |
| 144210532 | CCN1CCC2(CC1)COC3=CC4=C(C=C23)N(CC4)C(=O)C5=CC=C(C=C5)C6=C(C=C(C=C6)C7=NN=C(O7)C)C.Cl | 1 | test |
| 144210531 | CCCCN1C(=O)[C@H](NC(=O)C12CCN(CC2)CC3=CC=C(C=C3)OC4=CC=C(C=C4)C(=O)O)[C@@H](C5CCCCC5)O.Cl | 0 | train |
| 144210530 | C1C[C@H](CN(C1)CCC2=CC3=C(C=C2)OCO3)OC(C4=CC=CC=C4)C5=CC=CC=C5 | 0 | train |
| 144210529 | CC(C)C1=C(C(=CC=C1)C(C)C)NC(=O)[N-]S(=O)(=O)C2=CC(=CO2)C(C)(C)O.[Na+] | 0 | validation |
| 144210528 | CCOC1=NC2=C(C=C1)NC=C(C2=O)C(=O)NCC3=CC=CC=C3 | 1 | test |
| 144210527 | CCC1=NN(C2=C1C=CC(=C2)C3(CCC(CC3)C(=O)O)C#N)C4CCCCC4 | 0 | train |
| 144210526 | C1[C@H]([C@H](CN1C(=O)[C@@H]([C@H](CC2=CC=CC=C2)NC(=O)C3=CC4=C(N3)C=CC(=C4)Cl)O)O)O | 0 | train |
| 144210525 | COC1=C(C=C(C=C1)OC(F)(F)F)CN[C@H]2CCCN[C@H]2C3=CC=CC=C3 | 1 | validation |
| 144210524 | CCC1=C2C(=CC(=C1)C3=C(N(S(=O)(=O)C4=CC=CC=C43)C5=CC=CC=C5C(F)(F)F)C(=O)[O-])OCO2.[K+] | 0 | test |
| 144210523 | CC1=CC2=C3C(=C1)C(=N[C@H](C(=O)N3CC2)NC(=O)C4=CC=NC=C4)C5=CC=CC=C5 | 0 | train |
| 144210522 | CC(C)(C)C1=CC(=CC(=C1O)C(C)(C)C)/C=C¥2/C(=O)N=C(S2)N.CS(=O)(=O)O | 1 | train |
| 144210521 | CC1=C(C=CC(=C1)CC2=CC(=C(C=C2)N)C)N | 1 | validation |
| 144210520 | CN1C=C(C(=O)C(=C1)C2=CC(=CC=C2)C(F)(F)F)C3=CC=CC=C3 | 0 | test |
| 144210519 | CN1C(=O)C=C(S1)Cl | 0 | train |
| 144210518 | CCC(C)(C1=CC=C(C=C1)O)C2=CC=C(C=C2)O | 1 | train |
| 144210517 | C1=NN=CN1N | 0 | validation |
| 144210516 | C[C@]12CC(=O)[C@H]3[C@H]([C@@H]1CC[C@@]2(C(=O)CO)O)CCC4=CC(=O)C=C[C@]34C | 0 | test |
| 144210515 | C1=CC=C2C(=C1)C=CC3=CC=CC=C3N2C(=O)N | 0 | train |
| 144210514 | C(C(CCl)OP(=O)(OC(CCl)CCl)OC(CCl)CCl)Cl | 0 | train |
| 144210513 | C1=CN=CC=C1C(=O)NN | 0 | validation |
| 144210512 | CC(=O)NC | 0 | test |
| 144210511 | C1=CC(=CC=C1C(C2=CC=C(C=C2)Cl)C(Cl)Cl)Cl | 1 | train |
| 144210510 | C1(C(C2(C(=C(C1(C2(Cl)Cl)Cl)Cl)Cl)Cl)C(=O)O)C(=O)O | 0 | train |
| 144210509 | C1=C(C=C(C(=C1Cl)O)Cl)Cl | 1 | validation |
| 144210508 | C1CC(=O)OC1 | 0 | test |
| 144210507 | C(=O)(C(Cl)(Cl)Cl)O | 0 | train |
| 144210506 | C1=C(C(=CC(=C1Cl)Cl)Cl)O | 0 | train |
| 144210505 | CC1=C(C=C(C=C1)O)C | 0 | validation |
| 144210504 | CC1(C(=O)N(C(=O)N1Cl)Cl)C | 0 | test |
| 144210503 | CCOC(=O)C1=CC=CC=C1C(=O)OCC | 1 | train |
| 144210502 | C/C=C/C=C/C(=O)O | 0 | train |
| 144210501 | C1=CC=C(C=C1)C(Cl)(Cl)Cl | 0 | validation |
| 144210500 | C1=CC=C(C=C1)C(=O)O | 0 | test |

| | | | |
|---|---|---|---|
| 144210499 | CC1=CC=CC=C1N | 0 | train |
| 144210498 | C[C@]12C[C@@H]([C@]3([C@H]([C@@H]1C[C@H]([C@@]2(C(=O)CO)O)O)CCC4=CC(=O)C=C[C@@]43C)F)O | 0 | train |
| 144210497 | CC(C)(C)OOC(C)(C)CCC(C)(C)OOC(C)(C)C | 0 | validation |
| 144210496 | C[N+](C)(C)CC1CO1.[Cl-] | 0 | test |
| 144210495 | CCOP(=S)(OCC)SCSP(=S)(OCC)OCC | 1 | train |
| 144210494 | CN1CCC[C@H]1C2=CN=CC=C2 | 0 | train |
| 144210493 | CCCN(CCC)C(=O)SCCC | 0 | validation |
| 144210492 | C1[C@@H]2C=CC1[C@H]3[C@@H]2[C@]4(C(=C(C3(C4(Cl)Cl)Cl)Cl)Cl)Cl | 1 | test |
| 144210491 | C1=CC=C(C=C1)C(Cl)Cl | 0 | train |
| 144210490 | C1=COC(=C1)C=O | 0 | train |
| 144210489 | CCOS(=O)(=O)OCC | 0 | validation |
| 144210488 | CC1=CC=C(C=C1)N | 1 | test |
| 144210487 | C1=CC=C(C=C1)C2=CC=CC=C2 | 0 | train |
| 144210486 | CCCCOC(=O)CCCCC(=O)OCCCC | 0 | train |
| 144210485 | CCCCCCOC(=O)C1=CC=CC=C1C(=O)OCCCCCC | 1 | validation |
| 144210484 | CCCC(=O)O | 0 | test |
| 144210483 | C1CCC(=O)NCC1 | 0 | train |
| 144210482 | CCC(=NO)C | 0 | train |
| 144210481 | C(CCCN=C=O)CCN=C=O | 0 | validation |
| 144210480 | CC1=C(C=C(C=C1)N=C=O)N=C=O | 0 | test |
| 144210479 | C([C@H]([C@@H]1C(=C(C(=O)O1)O)[O-])O)O.[Na+] | 0 | train |
| 144210478 | CC1(C(=O)N(C(=O)N1Br)Br)C | 0 | train |
| 144210477 | CC1=C(C(=CC=C1)C)O | 0 | validation |
| 144210476 | C(C(=O)O)N(CC(=O)O)CC(=O)O | 0 | test |
| 144210475 | [C@@H]([C@H](C(=O)O)O)(C(=O)O)O | 0 | train |
| 144210474 | CCCCOC(=O)CCCCCCCCC(=O)OCCCC | 0 | train |
| 144210473 | C1=CC=C(C(=C1)O)O | 1 | validation |
| 144210472 | CC1=CCCC(C1/C=C(¥C)/C(=O)C)(C)C | 0 | test |
| 144210471 | CC(C)OC(=O)C(C)(C)OC1=CC=C(C=C1)C(=O)C2=CC=C(C=C2)Cl | 1 | train |
| 144210470 | C[C@@]1([C@H]2C[C@H]3[C@@H](C(=O)C(=C([C@]3(C(=O)C2=C(C4=C1C=CC=C4O)O)O)O)O)C(=O)N)N(C)C)O | 0 | train |
| 144210469 | CCCCCCCCCCCCOS(=O)(=O)[O-].[Na+] | 0 | validation |
| 144210468 | CCOP(=S)(OCC)OC1=NC(=C(C=C1Cl)Cl)Cl | 1 | test |
| 144210467 | CC(COCC(C)OC(=O)C1=CC=CC=C1)OC(=O)C2=CC=CC=C2 | 1 | train |
| 144210466 | CC(=O)OC1=CC=CC=C1C(=O)O | 0 | train |
| 144210465 | C=CC(=O)N | 0 | validation |
| 144210464 | C(C(CO)O)O | 0 | test |
| 144210463 | CC1=C(C=C(C=C1)N)N | 1 | train |
| 144210462 | CCCCCCC(=CC1=CC=CC=C1)C=O | 0 | train |
| 144210461 | C1=CC(=CC=C1CC2=CC=C(C=C2)N)N | 1 | validation |
| 144210460 | C1=CC(=CC(=C1)O)O | 0 | test |
| 144210459 | COC(=O)C1=CC=C(C=C1)C(=O)OC | 0 | train |
| 144210458 | CCCCCCCCCCCCCO | 0 | train |
| 144210457 | COC(=O)C1=CC=CC=C1O | 0 | validation |
| 144210456 | CC(=CCC/C(=C/CO)/C)C | 0 | test |
| 144210455 | C=CCOC(=O)C1=CC=CC=C1C(=O)OCC=C | 0 | train |
| 144210454 | CC1=C(C=C(C=C1)[N+](=O)[O-])N | 0 | train |
| 144210453 | C1=CC(=CC=C1N)OC2=CC=C(C=C2)N | 0 | validation |
| 144210452 | C/C=C/C1=CC=C(C=C1)OC | 0 | test |
| 144210451 | C1=CC(=CC=C1C(=O)O)[N+](=O)[O-] | 0 | train |
| 144210450 | CN(C)C1=CC=C(C=C1)CC2=CC=C(C=C2)N(C)C | 1 | train |
| 144210449 | CCC1=C(C(=NC(=N1)N)N)C2=CC=C(C=C2)Cl | 0 | validation |
| 144210448 | C1=CC(=S)N(C=C1)[O-].[Na+] | 1 | test |
| 144210447 | C1=CC(=CC=C1C(=O)N[C@@H](CCC(=O)O)C(=O)O)NCC2=CN=C3C(=N2)C(=O)N=C(N3)N | 0 | train |
| 144210446 | C(C(CO)([N+](=O)[O-])Br)O | 0 | train |
| 144210445 | C1=CC=C(C=C1)C(=O)[O-].[Na+] | 0 | validation |
| 144210444 | C(C(=O)O)C(CC(=O)O)(C(=O)O)O | 0 | test |
| 144210443 | CCCCOCCO | 0 | train |
| 144210442 | COC1=C(C=C(C=C1)[N+](=O)[O-])N | 0 | train |
| 144210441 | CC(C)(C)C1=CC(=C(C(=C1)C(C)(C)C)O)C(C)(C)C | 1 | validation |

| | | | |
|---|---|---|---|
| 144210440 | CCCCCCCCCCCCCO | 0 | test |
| 144210439 | C1C(O1)CN2C(=O)N(C(=O)N(C2=O)CC3CO3)CC4CO4 | 0 | train |
| 144210438 | CC1=C(C(=O)C=CO1)O | 0 | train |
| 144210437 | C1=CC(=CC=C1[N+](=O)[O-])O | 0 | validation |
| 144210436 | CN(C)C(=O)Cl | 0 | test |
| 144210435 | COC(=O)CCCC(=O)OC | 0 | train |
| 144210434 | CC(C)(C)C1=CC(=C(C=C1)O)C(C)(C)C | 0 | train |
| 144210433 | C1=NC=NN1 | 0 | validation |
| 144210432 | C1=C(C(=O)NC(=O)N1)F | 0 | test |
| 144210431 | CC(=O)OCC(COC(=O)C)OC(=O)C | 0 | train |
| 144210430 | C1C(O1)C2=CC=CC=C2 | 0 | train |
| 144210429 | C1=CC(=CC=C1C(=C(Cl)Cl)C2=CC=C(C=C2)Cl)Cl | 1 | validation |
| 144210428 | CCCCC(CC)C(=O)O | 0 | test |
| 144210427 | CCCCOP(=O)(OCCCC)OCCCC | 1 | train |
| 144210426 | C1=CC(=CC(=C1)C(=O)O)C(=O)O | 0 | train |
| 144210425 | COC1=C(C=CC(=C1)CC=C)O | 0 | validation |
| 144210424 | C1CNCCN1 | 0 | test |
| 144210423 | CC(C)(C)C1=CC=C(C=C1)O | 0 | train |
| 144210422 | CC1(C(=O)NC(=O)N1CO)C | 0 | train |
| 144210421 | C1=CC=C2C(=C1)C(=O)C3=C(C2=O)C=C(C=C3)N | 1 | validation |
| 144210420 | CC(=O)NC1=CC=C(C=C1)O | 0 | test |
| 144210419 | CC(C)(C)OOC(C)(C)C | 0 | train |
| 144210418 | C1(=O)C2(C3(C4(C1(C5(C2(C3(C(C45Cl)(Cl)Cl)Cl)Cl)Cl)Cl)Cl)Cl)Cl | 1 | train |
| 144210417 | CN1CCCC1=O | 0 | validation |
| 144210416 | CCCCCCCCCO | 0 | test |
| 144210415 | CC(=CCCC(=CC=O)C)C | 0 | train |
| 144210414 | C1CC2C(C(C1O2)C(=O)O)C(=O)O | 1 | train |
| 144210413 | COP(=S)(OC)SCN1C(=O)C2=CC=CC=C2C1=O | 0 | validation |
| 144210412 | CC1=C(C(=CC=C1)N)N | 1 | test |
| 144210411 | CC(C)(C)C1=C(C(=CC=C1)C(C)(C)C)O | 0 | train |
| 144210410 | CCC(C)(C)C1=CC(=C(C=C1)O)C(C)(C)CC | 0 | train |
| 144210409 | C(#N)C(C(=O)N)(Br)Br | 0 | validation |
| 144210408 | CCC(C)(C)C1=CC=C(C=C1)O | 0 | test |
| 144210407 | C1=CC(=CC=C1C(=O)O)N | 0 | train |
| 144210406 | CCC(C)C1=CC=C(C=C1)O | 0 | train |
| 144210405 | CC(CCO)O | 0 | validation |
| 144210404 | CCCCOCCOCCO | 0 | test |
| 144210403 | CC(C)CC(C)O | 0 | train |
| 144210402 | CC(C)(C)C1=C(C=CC(=C1)OC)O | 0 | train |
| 144210401 | CC(C)(C)C1=C(C=CC(=C1)O)O | 1 | validation |
| 144210400 | CCOCCOCCO | 0 | test |
| 144210399 | C1=C(C=C(C(=C1O)O)O)C(=O)OC2=CC(=CC(=C2O)O)C(=O)OC[C@@H]3[C@H]([C@@H]([C@H]([C@@H](O3)OC(=O)C4=CC(=C(C(=C4)OC(=O)C5=CC(=C(C(=C5)O)O)O)O)O)OC(=O)C6=CC(=C(C(=C6)OC(=O)C7=CC(=C(C(=C7)O)O)O)O)O)OC(=O)C8=CC(=C(C(=C8)OC(=O)C9=CC(=C(C(=C9)O)O)O)O)O)OC(=O)C1=CC(=C(C(=C1)OC(=O)C1=CC(=C(C(=C1)O)O)O)O)O | 0 | train |
| 144210398 | CCCCCCCCCCCO | 0 | train |
| 144210397 | COCCOCCOC | 0 | validation |
| 144210396 | CCCCCCCCCCC | 0 | test |
| 144210395 | CC1=CC(=O)/C(=C(/C)¥[O-])/C(=O)O1.[Na+] | 0 | train |
| 144210394 | C(CCl)OP(=O)(OCCCl)OCCCl | 0 | train |
| 144210393 | C=CN1CCCC1=O | 0 | validation |
| 144210392 | CC1=C(C=C(C=C1)S(=O)(=O)[O-])C.[Na+] | 0 | test |
| 144210391 | COC1=C(C=C(C=C1)CC=C)OC | 0 | train |
| 144210390 | CC(C)(C)OOC(=O)C1=CC=CC=C1 | 0 | train |
| 144210389 | CC(C)(C1=CC=CC=C1)OOC(C)(C)C2=CC=CC=C2 | 1 | validation |
| 144210388 | C1=CC=C2C(=C1)C=CC=N2 | 0 | test |
| 144210387 | C1=CC(=CC=C1C(C2=CC=C(C=C2)Cl)C(Cl)(Cl)Cl)Cl | 1 | train |
| 144210386 | C1COS(=O)(=O)C1 | 0 | train |
| 144210385 | CCCC(=O)OCC | 0 | validation |
| 144210384 | C(COCCO)O | 0 | test |
| 144210383 | CCCCCCCC1CCC(=O)O1 | 0 | train |

| | | | |
|---|---|---|---|
| 144210382 | CC1=CC=C(C=C1)N(C)C | 1 | train |
| 144210381 | CCCCC=O | 0 | validation |
| 144210380 | CCCOC(=O)C1=CC(=C(C(=C1)O)O)O | 1 | test |
| 144210379 | CC1=C(C(=C(C=C1)C)O)C | 0 | train |
| 144210378 | C1=CC=C(C=C1)C(=O)C2=CC=CC=C2 | 0 | train |
| 144210377 | C1=CC=C2C(=C1)C=CC(=O)O2 | 0 | validation |
| 144210376 | CC(C)(C1=CC=CC=C1)C2=CC=C(C=C2)O | 1 | test |
| 144210375 | CC1=C(C(=CC=C1)C)N | 0 | train |
| 144210374 | CC1=C(C=CC(=C1)O)Cl | 0 | train |
| 144210373 | COC1=CC=CC=C1N | 0 | validation |
| 144210372 | C1=CC=C2C(=C1)C3=CC=CC=C3O2 | 0 | test |
| 144210371 | CC1=CC(=C(C=C1)O)C | 0 | train |
| 144210370 | CC1=CC(=O)CC(C1)(C)C | 0 | train |
| 144210369 | CC1=CC(=C(C=C1)C)C | 0 | validation |
| 144210368 | C1=CC=C2C=C(C=CC2=C1)N | 1 | test |
| 144210367 | CCC(C)C1=CC=CC=C1O | 0 | train |
| 144210366 | C1=CC=C(C(=C1)Cl)Cl | 0 | train |
| 144210365 | C1=CC=C(C=C1)C(=O)CCl | 0 | validation |
| 144210364 | C1=CC=C(C=C1)CO | 0 | test |
| 144210363 | C(CCl)O | 0 | train |
| 144210362 | C1=CC=C(C=C1)O | 0 | train |
| 144210361 | COC1=C(C=CC(=C1)C2=CC(=C(C=C2)N)OC)N | 1 | validation |
| 144210360 | C1=CC(=C(C=C1Cl)Cl)O | 0 | test |
| 144210359 | CN(C)C=O | 0 | train |
| 144210358 | C1CCC(CC1)N | 0 | train |
| 144210357 | CCN(CC)CCO | 0 | validation |
| 144210356 | CN(C)C1=CC=CC=C1 | 0 | test |
| 144210355 | C(=O)(N)N | 0 | train |
| 144210354 | C(CO)NCCO | 0 | train |
| 144210353 | C1=CC(=CC=C1[N+](=O)[O-])Cl | 0 | validation |
| 144210352 | CC1=CC=CC=C1[N+](=O)[O-] | 0 | test |
| 144210351 | CCOCCO | 0 | train |
| 144210350 | C1=CC(=C(C=C1[N+](=O)[O-])[N+](=O)[O-])O | 0 | train |
| 144210349 | CC1=CC=C(C=C1)O | 0 | validation |
| 144210348 | CN1C2=C(C(=O)N(C1=O)C)NC=N2 | 0 | test |
| 144210347 | C1=CC=C(C=C1)NC2=CC=C(C=C2)N | 1 | train |
| 144210346 | C(CCC#N)CC#N | 0 | train |
| 144210345 | N(=O)[O-].[Na+] | 0 | validation |
| 144210344 | [N-]=[N+]=[N-].[Na+] | 0 | test |
| 144210343 | C1(=C(C(=C(C(=C1Cl)Cl)Cl)Cl)Cl)O | 1 | train |
| 144210342 | CCCCCCCCC(=O)O | 0 | train |
| 144210341 | CC1=CC=CC=C1O | 0 | validation |
| 144210340 | CCCCN(CCCC)CCCC | 0 | test |
| 144210339 | CCCCC(CC)CO | 0 | train |
| 144210338 | C1=CC(=CC=C1Cl)Cl | 0 | train |
| 144210337 | CC1=CC=C(C=C1)[N+](=O)[O-] | 0 | validation |
| 144210336 | CN1C=NC2=C1C(=O)NC(=O)N2C | 0 | test |
| 144210335 | C1=CC(=CC=C1O)O | 1 | train |
| 144210334 | C1=CC=C2C=C3C=CC=CC3=CC2=C1 | 1 | train |
| 144210333 | C1CC2=CC=CC3=C2C1=CC=C3 | 1 | validation |
| 144210332 | CC1=CC(=C(C=C1)OC)N | 0 | test |
| 144210331 | CC(C)(C)C1=CC=CC=C1O | 0 | train |
| 144210330 | CN1C=NC2=C1C(=O)N(C(=O)N2C)C | 0 | train |
| 144210329 | COC(=O)C1=CC=C(C=C1)O | 1 | validation |
| 144210328 | C1=CC=C2C=CC=CC2=C1 | 0 | test |
| 144210327 | CC1=CC(=CC=C1)[N+](=O)[O-] | 0 | train |
| 144210326 | CC(CO)O | 0 | train |
| 144210325 | C1=CC(=CC(=C1)Cl)Cl | 0 | validation |
| 144210324 | CCOC(=O)CC(CC(=O)OCC)(C(=O)OCC)O | 1 | test |
| 144210323 | CC(CCC=C(C)C)CCO | 0 | train |
| 144210322 | C1=CC=C(C=C1)OP(OC2=CC=CC=C2)OC3=CC=CC=C3 | 0 | train |
| 144210321 | CC(=C)C(=O)N | 0 | validation |

| | | | |
|---|---|---|---|
| 144210320 | CC(Cl)(Cl)Cl | 0 | test |
| 144210201 | C1(=C(C(=C(C(=C1Br)Br)Br)Br)Br)Br | 0 | validation |
| 144210200 | C1=CC=C2C(=C1)C=CC=C2NC(=S)N | 0 | test |
| 144210199 | CCCC[Sn](CCCC)(CCCC)O[Sn](CCCC)(CCCC)CCCC | 1 | train |
| 144210198 | C1CCC(CC1)N=C=NC2CCCCC2 | 1 | train |
| 144210197 | CC1=CC(=C2C(=C1)C(=O)C3=CC(=CC(=C3C2=O)O)O)O | 1 | validation |
| 144210196 | CCCCCCCCCCCCCCCCCCCl | 0 | test |
| 144210195 | CCCCCCCCCCCC=C | 0 | train |
| 144210194 | CCCCCCCCCCCC(=O)N(C)CC(=O)[O-].[Na+] | 0 | train |
| 144210193 | C1(=C(C(=C(C(=C1Cl)Cl)Cl)Cl)Cl)Cl | 0 | validation |
| 144210192 | CCCCCCCCCCCCCCCCN(C)C | 0 | test |
| 144210191 | C=CC(=O)OCCOCCOCCOCCOC(=O)C=C | 1 | train |
| 144210190 | CC(C)(C1=CC=C(C=C1)O)C2=CC=C(C=C2)O | 1 | train |
| 144210189 | CCCCOC(=O)C1=CC=CC=C1C(=O)OCC2=CC=CC=C2 | 1 | validation |
| 144210188 | COC1=CC=CC=C1O | 0 | test |
| 144210187 | CC(C)(C)C1=CC(=C(C=C1)O)O | 1 | train |
| 144210186 | C1=C(OC(=C1)[N+](=O)[O-])/C=N/NC(=O)N | 0 | train |
| 144210185 | C1=CC=C(C=C1)C2=CC(=O)C3=CC=CC=C3O2 | 1 | validation |
| 144210184 | C1=CC2=C(C(=C1)O)N=CC=C2 | 1 | test |
| 144210183 | C1=CC=C(C=C1)CC2=C(C=CC(=C2)Cl)O | 1 | train |
| 144210182 | C1=CC(=C(C=C1Cl)N)N | 1 | train |
| 144210181 | CC1=CC=CC=C1OP(=O)(OC2=CC=CC=C2C)OC3=CC=CC=C3C | 1 | validation |
| 144210180 | CC(CCl)OP(=O)(OC(C)CCl)OC(C)CCl | 1 | test |
| 144210179 | CC(C)CCCCCCCOP(=O)(OC1=CC=CC=C1)OC2=CC=CC=C2 | 1 | train |
| 144210178 | CC(C)(C)C1=CC=C(C=C1)OP(=O)(OC2=CC=CC=C2)OC3=CC=CC=C3 | 1 | train |
| 144210177 | C1=CC(=C(C=C1C(=O)Cl)Cl)F | 0 | validation |
| 144210176 | C1=C(NC=N1)/C=C/C(=O)O | 0 | test |
| 144210175 | [O-][Se](=O)[O-].[Na+].[Na+] | 1 | train |
| 144210174 | C1=CC=C(C=C1)C(=O)/C=C/C2=CC=C(C=C2)[N+](=O)[O-] | 1 | train |
| 144210173 | CC1=NC2=CC=CC=C2S1 | 0 | validation |
| 144210172 | CC1=NC2=CC=CC=C2C1(C)C | 0 | test |
| 144210171 | C1(=NC(=NN1)N)N | 1 | train |
| 144210170 | C1CC2=CC=CC=C2C(=O)C3=CC=CC=C31 | 1 | train |
| 144210169 | C1C2=CC=CC=C2C3=C1C=C(C=C3)N | 1 | validation |
| 144210168 | NO.Cl | 0 | test |
| 144210167 | O.O.O.O.O.O.O.O.O=[Zr].Cl.Cl | 0 | train |
| 144210166 | C1=CC=C(C=C1)NN=C2C=CC(=O)C=C2 | 1 | train |
| 144210165 | C1=CC=C2C=C3C4=CC=CC=C4C5=CC=CC=C5C3=CC2=C1 | 1 | validation |
| 144210164 | COC1=NC(=NC2=C1NC=N2)N | 0 | test |
| 144210163 | C1=CC=C2C(=C1)C=C3C=CC=CC3=C2CO | 1 | train |
| 144210162 | C1=CC=C2C(=C1)C(=O)C3=C(C2=O)C=C(C=C3)Cl | 0 | train |
| 144210161 | C1=CC=C2C(=C1)C=C3C=CC=CC3=C2C#N | 0 | validation |
| 144210160 | C1=CC=C2C(=C1)C(=C3C=CC=CC3=C2Cl)C=O | 1 | test |
| 144210159 | CC1(C2CCC1(C(=O)C2=O)C)C | 0 | train |
| 144210158 | C1=CC=C2C=C3C=C(C=CC3=CC2=C1)Cl | 1 | train |
| 144210157 | C1=CC=C2C(=C1)C(=NN=C2Cl)Cl | 0 | validation |
| 144210156 | C1=CC=C2C(=C1)C=C3C=CC=CC3=C2Br | 1 | test |
| 144210155 | COC1=CC(=C(C=C1)N)OC | 1 | train |
| 144210154 | CC(=O)C1=CC2=C(C=CC3=CC=CC=C32)C=C1 | 1 | train |
| 144210153 | C1=CC(=CC(=C1)[N+](=O)[O-])C=O | 0 | validation |
| 144210152 | CC[Ge](CC)(CC)Cl | 0 | test |
| 144210151 | C1=CC=NC(=C1)C(=O)O | 0 | train |
| 144210150 | CN1C(=O)C2=C(N=CN2)N=C1N | 0 | train |
| 144210149 | CN1C=NC2=C1C(=NC=N2)N | 0 | validation |
| 144210148 | C1=CC=C2C(=C1)N=CC=N2 | 0 | test |
| 144210147 | C1C2=CC=CC=C2C(=O)C3=CC=CC=C31 | 1 | train |
| 144210146 | CC(=CCC1=C(C2=CC=CC=C2C(=O)C1=O)O)C | 1 | train |
| 144210145 | C1=CC2=C3C(=C1)C(=O)C(=O)C3=CC=C2 | 1 | validation |
| 144210144 | CC(=O)C1=CC2=C(C=C1)C3=CC=CC=C3C2 | 1 | test |
| 144210143 | C[C@@H]1CC[C@@H]2[C@]13CC[C@@]([C@H](C3)C2(C)C)(C)O | 1 | train |
| 144210142 | CC1CN1 | 0 | train |
| 144210141 | C1=CC=C2C(=C1)C(=O)NN2 | 0 | validation |

| | | | |
|---|---|---|---|
| 144210140 | C1=CC=C2C(=C1)C=C3C=CC=CC3=C2C(=O)O | 1 | test |
| 144210139 | C1=CC=C(C=C1)C(=O)CBr | 1 | train |
| 144210138 | C(#N)C(=C(C#N)C#N)C#N | 1 | train |
| 144210137 | C1CCC2=NCCCN2CC1 | 0 | validation |
| 144210136 | CC1=CC=CC2=C1C=C3C=CC4=C5C3=C2C=CC5=CC=C4 | 1 | test |
| 144210135 | C1=CC2=C(C=C1[N+](=O)[O-])C(=O)C(=O)N2 | 0 | train |
| 144210134 | CCOC(=O)C(C(=O)C)Cl | 0 | train |
| 144210133 | C1=CC=C2C(=C1)NC(=O)O2 | 0 | validation |
| 144210132 | C1=CC=C2C(=C1)C(=CC(=C2N)N=NC3=CC=C(C=C3)C4=CC=C(C=C4)N=NC5=C(C6=CC=CC=C6C(=C5)S(=O)(=O)[O-])N)S(=O)(=O)[O-].[Na+].[Na+] | 0 | test |
| 144210131 | C1=CC=C2C(=C1)C=C(C3=CC=CC=C23)Br | 1 | train |
| 144210130 | C1=CC=C(C(=C1)C2=C3C=C(C(=O)C(=C3OC4=C(C(=C(C=C24)I)[O-])I)I)I)C(=O)[O-].[Na+].[Na+] | 0 | train |
| 144210129 | C1=C(N=C(N=C1Cl)N)Cl | 0 | validation |
| 144210128 | C1=CC(=CC=C1C=O)[N+](=O)[O-] | 1 | test |
| 144210127 | C1=CC=C(C(=C1)C=O)[N+](=O)[O-] | 0 | train |
| 144210126 | CN1C=NC2=C1NC(=NC2=O)N | 0 | train |
| 144210125 | CCCCON=O | 0 | validation |
| 144210124 | C1=CC(=O)NC1=O | 1 | test |
| 144210123 | C1=CC2=C(C=C1[N+](=O)[O-])C=NN2 | 0 | train |
| 144210122 | C1=CC=C(C(=O)C=C1)O | 0 | train |
| 144210121 | C1=CC=C2C(=C1)C(=C3C=CC=CC3=C2Br)Br | 0 | validation |
| 144210120 | COC1=CC(=CC(=C1OC)OC)[C@H]2[C@@H]3[C@H](COC3=O)[C@H](C4=CC5=C(C=C24)OCO5)O | 1 | test |
| 144210119 | CN(C)C1=CC=C(C=C1)CCO | 0 | train |
| 144210118 | C1=CC=C2C(=C1)C(=O)N=CN2 | 0 | train |
| 144210117 | C1=CC=C2C(=C1)C3=CC=CC=C3C2=O | 0 | validation |
| 144210116 | CN(C)C1=CC=C(C=C1)/C=C/C2=CC=C(C=C2)[N+](=O)[O-] | 1 | test |
| 144210115 | C1CS1 | 0 | train |
| 144210114 | C1=C(N=C(N=C1Cl)Cl)Cl | 0 | train |
| 144210113 | C1=CC=C(C(=C1)O)F | 0 | validation |
| 144210112 | COS(=O)(=O)C(F)(F)F | 0 | test |
| 144210111 | C1CC2=C(C=C3C=CC4=C5C3=C2C=CC5=CC=C4)C(=O)C1 | 1 | train |
| 144210110 | C1=CC=C(C=C1)C2=CC=CC=C2F | 0 | train |
| 144210109 | C1=CN=CC=N1 | 0 | validation |
| 144210108 | CC1=C(C=CC(=C1)O)N | 1 | test |
| 144210107 | CC1=C(C(=CC=C1)O)N | 1 | train |
| 144210106 | C1=CC=C2C(=C1)C3=C4C2=CC=CC4=C(C=C3)N | 1 | train |
| 144210105 | C1=CC=C2C=NN=CC2=C1 | 0 | validation |
| 144210104 | C1=CC=C2C(=C1)C=C(C3=CC=CC=C23)C#N | 1 | test |
| 144210103 | CCOC(=O)CC(=O)C1=CC(=C(N=C1Cl)Cl)F | 0 | train |
| 144210102 | CC(C)(C)OC(=O)C1=CC(=CC=C1)N | 0 | train |
| 144210101 | CC[C@@H]1[C@@]([C@@H]([C@H](C(=O)[C@@H](C[C@@]([C@@H](C([C@@H]([C@H](C(=O)O1)C)O[C@H]2C[C@@]([C@H]([C@@H](O2)C)O)(C)OC)C)O[C@H]3[C@@H]([C@H](C[C@H](O3)C)N(C)C)O)(C)OC)C)C)O)(C)O | 0 | validation |
| 144210100 | CC1=CC=C(C=C1)NC2=C3C(=C(C=C2)O)C(=O)C4=CC=CC=C4C3=O | 0 | test |
| 144210099 | C1=CC=C2C(=C1)C(=O)C3=C(C2=O)C=C(C=C3)O | 1 | train |
| 144210098 | C1=CC=C2C(=C1)C(=CC=C2N)N | 1 | train |
| 144210097 | CC1=CNC(=S)NC1=O | 0 | validation |
| 144210096 | CCCCCON=O | 0 | test |
| 144210095 | C=CC(=O)OCCCl | 0 | train |
| 144210094 | C1=COC(=C1)/C=C/C=O | 0 | train |
| 144210093 | COC1=CC=C(C=C1)NC2=CC=C(C=C2)OC | 1 | validation |
| 144210092 | C1=CC2=CC3=C(C(=CC=C3)O)C(=C2C(=C1)O)O | 0 | test |
| 144210091 | C1=CC(=CC=C1N)[As](=O)(O)[O-].[Na+] | 0 | train |
| 144210090 | C1=C(C=C(C(=C1S(=O)(=O)O)O)N)Cl | 0 | train |
| 144210089 | C1C=CC=[C-]1.[CH-]1C=CC=C1.Cl[Zr+2]Cl | 1 | validation |
| 144210088 | C1=CC(=CC=C1CC(C(=O)O)N)F | 0 | test |
| 144210087 | [CH-]1C=CC=C1.[CH-]1C=CC=C1.[Cr+2] | 0 | train |
| 144210086 | CC1=CC=C(C=C1)OP(=O)(OC2=CC=CC=C2)OC3=CC=CC=C3 | 1 | train |
| 144210085 | C1=C(C=C(C(=C1[N+](=O)[O-])O)N)[N+](=O)[O-] | 0 | validation |
| 144210084 | C1=CC2=C(C(=C1)[N+](=O)[O-])C(=CC=C2)[N+](=O)[O-] | 1 | test |

| | | | |
|---|---|---|---|
| 144210083 | COC1=CC=CC=C1/C=C/C=O | 1 | train |
| 144210082 | CC1=C(C=C(C=C1)N)OC | 1 | train |
| 144210081 | CCC(CC)C=O | 0 | validation |
| 144210080 | CC1CCCC1 | 0 | test |
| 144210079 | C(C(CS)O)O | 0 | train |
| 144210078 | C1=CC=C(C=C1)C(=O)OC2=CC=CC=C2 | 0 | train |
| 144210077 | CC(=O)CC(=O)C1=CC=CC=C1 | 0 | validation |
| 144210076 | C[C@H]1[C@H](NC(=O)N1C)C2=CC=CC=C2 | 0 | test |
| 144210075 | C1CN(NC1=O)C2=CC=CC=C2 | 1 | train |
| 144210074 | C1=CC=C2C(=C1)C(=O)C(=O)N2 | 0 | train |
| 144210073 | C1=CC(=C(C=C1[N+](=O)[O-])[N+](=O)[O-])S(=O)(=O)O.O.O | 1 | validation |
| 144210072 | CC(=O)C1CCCCC1=O | 0 | test |
| 144210071 | CCCCCC(C#C)O | 0 | train |
| 144210070 | CC[C@]12CC[C@H]3[C@H]([C@@H]1CC[C@]2(C#C)O)CCC4=CC(=O)CC[C@H]34 | 0 | train |
| 144210069 | [Be+2].O.O.O.O.[O-]S(=O)(=O)[O-] | 0 | validation |
| 144210068 | CCCCCCCCCCCCCBr | 0 | test |
| 144210067 | CCCCCCCCC(=O)Cl | 0 | train |
| 144210066 | [Cl-].[Cl-].[Cu+2] | 0 | train |
| 144210065 | C(CCCCCC(=O)O)CCCCCBr | 1 | validation |
| 144210064 | C(CCBr)CCl | 0 | test |
| 144210063 | CCCCCCCCCCCBr | 0 | train |
| 144210062 | CCCCCCCCCBr | 0 | train |
| 144210061 | C1=CC(=C(C(=C1)Cl)S(=O)(=O)Cl)Cl | 0 | validation |
| 144210060 | CCCCCCl | 0 | test |
| 144210059 | CCCCCCCCCCCCCCCBr | 0 | train |
| 144210058 | CCOC(=O)CS | 0 | train |
| 144210057 | COCC(CO)O | 0 | validation |
| 144210056 | COC1=C(C=C(C=C1)C=O)O | 0 | test |
| 144210055 | CCC1=CC=CC(=C1)C | 0 | train |
| 144210054 | CCCOC(=O)C(C)O | 0 | train |
| 144210053 | CCN(CC)CCNC(=O)C1=CC=C(C=C1)N.Cl | 0 | validation |
| 144210052 | C1=CC(=CC=C1CCC(=O)C2=C(C=C(C=C2O)O)O)O | 1 | test |
| 144210051 | CC(=O)C(=O)OC | 0 | train |
| 144210050 | C=CCCC=C | 0 | train |
| 144210049 | C[N+](C)(C)CC(=O)[O-].O | 0 | validation |
| 144210048 | CCCC(C)CC | 0 | test |
| 144210047 | C1=CC=C(C=C1)OCCBr | 0 | train |
| 144210046 | CCC(C)(C)CC | 0 | train |
| 144210045 | C(CN)CNCCCN | 0 | validation |
| 144210044 | CN1[C@@H]2CC[C@H]1CC(C2)OC(=O)C(CO)C3=CC=CC=C3.CN1[C@@H]2CC[C@H]1CC(C2)<br>OC(=O)C(CO)C3=CC=CC=C3.OS(=O)(=O)O | 0 | test |
| 144210043 | CCCCCCCCCCCCCCCCl | 0 | train |
| 144210042 | C1=CC(=C(C=C1SC#N)[N+](=O)[O-])N | 0 | train |
| 144210041 | C1(=C(N=C(N=C1N)N)N)N.OS(=O)(=O)O | 0 | validation |
| 144210040 | C1=CC=C(C=C1)NC2=CC=CC=C2N | 1 | test |
| 144210039 | CC1=NC2=CC=CC=C2C(=O)O1 | 1 | train |
| 144210038 | CCOC(=O)C1=CC=C(C=C1)I | 0 | train |
| 144210037 | CNC[C@@H](C1=CC(=C(C=C1)O)O)O.[C@@H]([C@H](C(=O)O)O)(C(=O)O)O | 0 | validation |
| 144210036 | CCN(CC)CCCC(C)NC1=C2C=CC(=CC2=NC=C1)Cl.OP(=O)(O)O.OP(=O)(O)O | 0 | test |
| 144210035 | C/C=C(¥C)/C=O | 0 | train |
| 144210034 | C1CC2=CC=CC=C2NC3=CC=CC=C31 | 1 | train |
| 144210033 | C1=COC(=C1)C(=O)C(=O)C2=CC=CO2 | 0 | validation |
| 144210032 | CCCCCCCCCCCCCl | 0 | test |
| 144210031 | CCCCCCCCCCl | 0 | train |
| 144210030 | CN1CCC2=CC=CC3=C2[C@H]1CC4=C3C(=C(C=C4)O)O.CN1CCC2=CC=CC3=C2[C@H]1CC4<br>=C3C(=C(C=C4)O)O.O.Cl.Cl | 1 | train |
| 144210029 | CCOC(=O)C(C)(C)C | 0 | validation |
| 144210028 | C1=CN=C(N=C1Cl)Cl | 0 | test |
| 144210027 | CC[C@@H](C1=CC=CC=C1)N | 0 | train |
| 144210026 | C1=CC(=CC=C1N)F | 0 | train |
| 144210025 | CC(CC(=O)Cl)CC(C)(C)C | 0 | validation |
| 144210024 | C1=CC(=CC=C1C(=O)C(=O)C2=CC=C(C=C2)Br)Br | 0 | test |

| | | | |
|---|---|---|---|
| 144210023 | CCCCCCCCCCCCCCCCCBr | 0 | train |
| 144210022 | C1=CC(=C(C=C1[N+](=O)[O-])Cl)F | 0 | train |
| 144210021 | CCC(=O)C1=CC(=CC=C1)Cl | 0 | validation |
| 144210020 | C(CCCCCBr)CCCCCO | 1 | test |
| 144210019 | C1=CC=C(C=C1)C(=O)CC(=O)C(F)(F)F | 1 | train |
| 144210018 | C1=CC(=C(C=C1O)C=O)Br | 1 | train |
| 144210017 | C1(=C(C(=O)C1=O)O)O | 0 | validation |
| 144210016 | C1=CC2=C(C=C1N=C=S)C(=O)OC23C4=C(C=C(C=C4)O)OC5=C3C=CC(=C5)O | 0 | test |
| 144210015 | C1[C@H]([C@@H]([C@H]([C@@H]([C@H]1N)O[C@@H]2[C@@H]([C@H]([C@@H]([C@H](O2)CN)O)O)O)O)O[C@@H]3[C@@H]([C@H]([C@@H]([C@H](O3)CO)O)N)O)N.OS(=O)(=O)O | 0 | train |
| 144210014 | CCCCCCCCCCl | 0 | train |
| 144210013 | COC(=O)C1=CC=C(C=C1)CBr | 1 | validation |
| 144210012 | CCOCCOC(=O)C(=C)C | 1 | test |
| 144210011 | CC(C)(C)OC(=O)N1C[C@H](C[C@H]1C(=O)O)F | 0 | train |
| 144210010 | C(CCCCl)CCO | 0 | train |
| 144210009 | C(#N)[N-]C#N.[Na+] | 0 | validation |
| 144210008 | C(CC(=O)O)C(C(=O)O)N.O | 0 | test |
| 144210007 | CCCCN(CCCC)C(=S)SSC(=S)N(CCCC)CCCC | 1 | train |
| 144210006 | CCO/C=C¥1/C(=O)OC(=N1)C2=CC=CC=C2 | 1 | train |
| 144210005 | CCCCCCCCCCCC[N+](C)(C)CCCS(=O)(=O)[O-] | 0 | validation |
| 144210004 | C[C@H]1/C=C/C=C(¥C(=O)NC2=CC(=C3C(=C2O)C(=C(C4=C3C(=O)[C@](O4)(O/C=C/[C@@H]([C@H]([C@H]([C@@H]([C@@H]([C@@H]([C@H]1O)C)O)C)OC(=O)C)C)OC)C)O)[O-])/C.[Na+] | 1 | test |
| 144210003 | C1=CNC(=S)N=C1 | 0 | train |
| 144210002 | [B-](F)(F)(F)F.[K+] | 0 | train |
| 144210000 | CC(C)CC(=O)C(=O)C | 0 | test |
| 144209999 | CN1CCCCC1CCN2C3=CC=CC=C3SC4=C2C=C(C=C4)SC.Cl | 1 | train |
| 144209998 | C1=CC=C(C=C1)C(=CC=O)C2=CC=CC=C2 | 1 | train |
| 144209997 | [Li+].[Li+].[O-]S(=O)(=O)[O-] | 0 | validation |
| 144209996 | CCN1C=C[N+](=C1)C.[N+](=O)([O-])[O-] | 0 | test |
| 144209995 | CCCCN1CN(C=C1)C.Br | 0 | train |
| 144209994 | CC(C)(C)C1=CC=C(C=C1)C(=O)CC(=O)C2=CC=C(C=C2)OC | 1 | train |
| 144209993 | C(C(=O)C(Cl)Cl)Cl | 1 | validation |
| 144209992 | CCCCCCCCCCCCCC=O | 0 | test |
| 144209991 | C1=CC=C2C(=C1)C(=CN2)CC(C(=O)O)N | 1 | train |
| 144209990 | C1=C2C(=CC(=C1Cl)Cl)OC3=CC(=C(C=C3O2)Cl)Cl | 1 | train |
| 144209989 | C1=C(C=C(C(=C1Cl)[O-])SC2=CC(=CC(=C2[O-])Cl)Cl)Cl.[Na+].[Na+] | 1 | validation |
| 144209988 | CC1=CC(=C(C2=CC=C(C=C2)N)C3=CC=C(C=C3)N)C=CC1=N.Cl | 0 | test |
| 144209987 | C1=CC=C2C(=C1)C(=CC=C2S(=O)(=O)[O-])N/N=C¥3/C4=C(C=C(C=C4)S(=O)(=O)[O-])C=C(C3=O)S(=O)(=O)[O-].[Na+].[Na+].[Na+] | 0 | train |
| 144209986 | CC1=CC(=C(C=C1C)N/N=C¥2/C3=C(C=C(C=C3)S(=O)(=O)[O-])C=C(C2=O)S(=O)(=O)[O-])C.[Na+].[Na+] | 0 | train |
| 144209985 | CCN1C=C(C(=O)C2=CC3=C(C=C21)OCO3)C(=O)O | 0 | validation |
| 144209984 | C1CN(CCN1)N=O | 0 | test |
| 144209983 | O=[Mo](=O)=O | 0 | train |
| 144209982 | CN(C)C1=NC(=NC(=N1)N(C)C)N(C)C | 0 | train |
| 144209981 | C1CCN(CC1)C(=S)SSSSSSC(=S)N2CCCCC2 | 1 | validation |
| 144209980 | C1CN(CCN1N=O)N=O | 0 | test |
| 144209979 | C1CCC(CC1)NS(=O)(=O)[O-].[Na+] | 0 | train |
| 144209978 | CN(C)C(=S)[S-].CN(C)C(=S)[S-].[Cu+2] | 1 | train |
| 144209977 | O=[Bi].Cl | 0 | validation |
| 144209976 | C(C(=O)O)C(CC(=O)[O-])(C(=O)[O-])O.[NH4+].[NH4+] | 0 | test |
| 144209975 | [NH4+].[NH4+].[O-]S(=O)(=S)[O-] | 0 | train |
| 144209974 | C(=O)([O-])[O-].[Sr+2] | 0 | train |
| 144209973 | C(=O)[O-].[K+] | 0 | validation |
| 144209972 | C([C@H]([C@H]([C@@H]([C@H](C(=O)[O-])O)O)O)O)O.[K+] | 0 | test |
| 144209971 | CC(C)(C)CC(C)(C)C1=CC=C(C=C1)NC2=CC=C(C=C2)C(C)(C)CC(C)(C)C | 0 | train |
| 144209970 | CCCCCCCCCCCCCCCCCCOCCO | 0 | train |
| 144209969 | CC(=O)[O-].CC(=O)[O-].CC(=O)[O-].[Sb+3] | 0 | validation |
| 144209968 | C1C[C@@H]2[C@@H]3CC[C@H](C3)[C@@H]2C1 | 0 | test |
| 144209967 | COC(=O)C1=CC2=C(C=C1)C=C(C=C2)C(=O)OC | 0 | train |

| | | | |
|---|---|---|---|
| 144209966 | CCCCCCCCCCCCCCCC(=O)OC | 0 | train |
| 144209965 | C(C(C(C(C(C(F)(F)F)(F)F)(F)F)(F)F)(F)F)(C(C(C(F)(F)F)(F)F)(F)F)(F)F)(F)F | 1 | validation |
| 144209964 | CC(C)C1=CC=C(C=C1)OP(=O)(OC2=CC=C(C=C2)C(C)C)OC3=CC=C(C=C3)C(C)C | 1 | test |
| 144209963 | CC(C)C1=CC=C(C=C1)OP(=O)(OC2=CC=C(C=C2)C(C)C)OC3=CC=C(C=C3)C(C)C | 1 | train |
| 144209962 | CC(C)C1=CC=C(C=C1)OP(=O)(OC2=CC=C(C=C2)C(C)C)OC3=CC=C(C=C3)C(C)C | 1 | train |
| 144209961 | CC1(CC(CC(N1)(C)C)OC(=O)CCCCCCCCC(=O)OC2CC(NC(C2)(C)C)(C)C)C | 0 | validation |
| 144209960 | CCCCCCCCCCCCCCCCCCOC(=O)C(=C)C | 0 | test |
| 144209959 | CCCCCCCCCCCCCCCC/C=C/C1CC(=O)OC1=O | 0 | train |
| 144209958 | CCCCCCCCCC(CC)C1=CC=C(C=C1)S(=O)(=O)O | 1 | train |
| 144209957 | C1=CC=C(C=C1)C2=CC=C(C=C2)C3=CC=CC=C3 | 1 | validation |
| 144209956 | CC(C)C1=CC=CC=C1C(C)C | 1 | test |
| 144209955 | C/C=C/C=C/C(=O)[O-].[K+] | 0 | train |
| 144209954 | CC(C)(C#N)N | 0 | train |
| 144209953 | CCCCCCCCCCCCCCOC(=O)CCSCCC(=O)OCCCCCCCCCCCCC | 0 | validation |
| 144209952 | C=CC(=O)[O-].[Na+] | 1 | test |
| 144209951 | C(N(CP(=O)(O)O)CP(=O)(O)O)P(=O)(O)O | 0 | train |
| 144209950 | COC(=O)C1CC(=O)C(CC1=O)C(=O)OC | 0 | train |
| 144209949 | C(C(=O)[O-])S.[NH4+] | 0 | validation |
| 144209948 | CC1=CC(=C(C=C1)N/N=C¥2/C3=CC=CC=C3C=C(C2=O)C(=O)[O-])S(=O)(=O)[O-].[Ca+2] | 0 | test |
| 144209947 | CCCCCCCCCCCCCCCCCI | 0 | train |
| 144209946 | CCCCCCCCCCCCCCC=C | 0 | train |
| 144209945 | C1=CC=C(C=C1)C(=O)[O-].[K+] | 0 | validation |
| 144209944 | C(=O)(C(=O)[O-])[O-].[Ca+2] | 0 | test |
| 144209943 | C([C@H]([C@H]([C@@H]([C@H](C(=O)O)O)O)O)O)O | 0 | train |
| 144209942 | CCCCCCCC/C=C¥CCCCCCCC(=O)OCCCC | 0 | train |
| 144209941 | C(C(=O)O)NCC(=O)O | 0 | validation |
| 144209940 | CCCCC(CC)C(=O)[O-].CCCCC(CC)C(=O)[O-].[Ca+2] | 0 | test |
| 144209939 | CC(=O)[O-].[Na+] | 0 | train |
| 144209938 | CC(=O)[O-].[K+] | 0 | train |
| 144209937 | CCCCCCCCCCCCCCCCC=C | 0 | validation |
| 144209936 | CCCCCCCCCCCCCCN(C)C | 0 | test |
| 144209935 | CC(C)CC(CC(C)C)O | 0 | train |
| 144209934 | C(CN(CC(=O)O)CC(=O)O)N(CC(=O)O)CC(=O)O | 0 | train |
| 144209933 | CCC(=O)[O-].[Na+] | 0 | validation |
| 144209932 | C1CCCCCCCCCCC1 | 0 | test |
| 144209931 | [C-]#N.[Cu+] | 0 | train |
| 144209930 | C1=CC2=C3C(=C1)C4=CC=CC5=C4C6=C(C=C5)C=CC(=C36)C=C2 | 0 | train |
| 144209929 | CCCCCCCCCCCCN=C(N)N.CC(=O)O | 0 | validation |
| 144209928 | [NH3+]O.[NH3+]O.[O-]S(=O)(=O)[O-] | 0 | test |
| 144209926 | C(/C=C/CBr)Br | 0 | train |
| 144209925 | C1=CC=C(C=C1)NO | 1 | validation |
| 144209924 | C1=CC=C(C=C1)/C=C/C2=CC=CC=C2 | 1 | test |
| 144209923 | CN1[C@@H]2CC[C@H]1CC(C2)O | 0 | train |
| 144209922 | CCCCCCCCCCCCOC(=O)C1=CC(=C(C(=C1)O)O)O | 0 | train |
| 144209921 | CC1=C(C(CC=C1)(C)C)C=O | 0 | validation |
| 144209920 | CCCCCCCCCCCCCCCCBr | 0 | test |
| 144209919 | CCCCCCCCCCCCCCBr | 0 | train |
| 144209918 | CC(C)(C)C(=O)CC(=O)C(C)(C)C | 0 | train |
| 144209917 | CCCCCCBr | 0 | validation |
| 144209916 | C(CCBr)CCBr | 0 | test |
| 144209915 | CCCCCBr | 0 | train |
| 144209914 | C(CBr)CBr | 0 | train |
| 144209913 | CCCCC/C(=C/C1=CC=CC=C1)/CO | 1 | validation |
| 144209912 | [N+](=O)([O-])[O-].[N+](=O)([O-])[O-].[Ba+2] | 0 | test |
| 144209911 | C1=CC(=CC=C1CBr)[N+](=O)[O-] | 0 | train |
| 144209910 | CCCCCCCCCCCCCC[P+](CCCC)(CCCC)CCCC.CCCCCCCCCCCCC1=CC=CC=C1S(=O)(=O)[O-<br>] | 1 | train |
| 144209909 | CCC[N+]1(CCCC1)C.C(F)(F)(F)S(=O)(=O)[O-] | 0 | validation |
| 144209908 | CCC[N+]1(CCCC1)C.[I-] | 0 | test |
| 144209907 | CCC[N+]1(CCCC1)C.F[P-](F)(F)(F)(F)F | 0 | train |
| 144209906 | CCC[N+]1(CCCC1)C.[Cl-] | 0 | train |

| | | | |
|---|---|---|---|
| 144209905 | CCC[N+]1(CCCC1)C.[Br-] | 0 | validation |
| 144209904 | CC[N+]1(CCCC1)C.C(F)(F)(F)S(=O)(=O)[O-] | 0 | test |
| 144209903 | CC[N+]1(CCCC1)C.[Br-] | 0 | train |
| 144209902 | [B-](F)(F)(F)F.CC[N+]1(CCCC1)C | 0 | train |
| 144209901 | [B-](F)(F)(F)F.CCCC[N+]1(CCCC1)C | 0 | validation |
| 144209900 | CCCC[N+]1(CCCC1)C.[Br-] | 0 | test |
| 144209899 | CCCCCC[N+]1=CC=CC=C1.C(F)(F)(F)S(=O)(=O)[O-] | 0 | train |
| 144209898 | [B-](F)(F)(F)F.CCCCCC[N+]1=CC=CC=C1 | 0 | train |
| 144209897 | CCCCCC[N+]1=CC=CC=C1.[I-] | 0 | validation |
| 144209896 | [B-](F)(F)(F)F.CC[N+]1=CC=CC=C1 | 0 | test |
| 144209895 | CC[N+]1=CC=CC=C1.[I-] | 0 | train |
| 144209894 | CC[N+]1=CC=CC=C1.C(F)(F)(F)S(=O)(=O)[O-] | 0 | train |
| 144209893 | CC[N+]1=CC=CC=C1.[Br-] | 0 | validation |
| 144209892 | CCCC[N+]1=CC=CC=C1.C(F)(F)(F)S(=O)(=O)[O-] | 0 | test |
| 144209891 | [B-](F)(F)(F)F.CCCC[N+]1=CC=CC=C1 | 0 | train |
| 144209890 | CCCC[N+]1=CC=CC=C1.[I-] | 0 | train |
| 144209889 | CCCC[N+]1=CC=C(C=C1)C.C(F)(F)(F)S(=O)(=O)[O-] | 0 | validation |
| 144209888 | [B-](F)(F)(F)F.CCCC[N+]1=CC=C(C=C1)C | 0 | test |
| 144209887 | CCCC[N+]1=CC=C(C=C1)C.[I-] | 0 | train |
| 144209886 | CCCC[N+]1=CC=CC(=C1)C.C(F)(F)(F)S(=O)(=O)[O-] | 0 | train |
| 144209885 | CCCC[N+]1=CC=C(C=C1)C.F[P-](F)(F)(F)(F)F | 0 | validation |
| 144209884 | [B-](F)(F)(F)F.CCCC[N+]1=CC=CC(=C1)C | 0 | test |
| 144209883 | CCCC[N+]1=CC=C(C=C1)C.[Cl-] | 1 | train |
| 144209882 | CCCC[N+]1=CC=CC(=C1)C.[I-] | 0 | train |
| 144209881 | CCCC[N+]1=CC=C(C=C1)C.[Br-] | 1 | validation |
| 144209880 | CCCC[N+]1=CC=CC(=C1)C.F[P-](F)(F)(F)(F)F | 0 | test |
| 144209879 | CCCC[N+]1=CC=CC(=C1)C.[Cl-] | 0 | train |
| 144209878 | CCCC[N+]1=CC=CC=C1C.[I-] | 0 | train |
| 144209877 | CCCC[N+]1=CC=CC(=C1)C.[Br-] | 0 | validation |
| 144209876 | CCCC[N+]1=CC=CC=C1C.F[P-](F)(F)(F)(F)F | 0 | test |
| 144209875 | CCCC[N+]1=CC=CC=C1C.C(F)(F)(F)S(=O)(=O)[O-] | 0 | train |
| 144209874 | CCCC[N+]1=CC=CC=C1C.[Cl-] | 0 | train |
| 144209873 | [B-](F)(F)(F)F.CCCC[N+]1=CC=CC=C1C | 0 | validation |
| 144209872 | CCCC[N+]1=CC=CC=C1C.[Br-] | 0 | test |
| 144209871 | CCCCCCCCCCCCCCC(CO)NC(=O)CCCCCCCCCCCCC | 0 | train |
| 144209870 | [B-](F)(F)(F)F.CCC[N+]1(CCCCC1)C | 0 | train |
| 144209869 | CCC[N+]1(CCCCC1)C.[I-] | 0 | validation |
| 144209868 | CCC[N+]1(CCCCC1)C.F[P-](F)(F)(F)(F)F | 0 | test |
| 144209867 | CCC[N+]1(CCCCC1)C.[Br-] | 0 | train |
| 144209866 | CCCC[N+]1(CCCCC1)C.[I-] | 0 | train |
| 144209865 | CCC[N+]1(CCCCC1)C.C(F)(F)(F)S(=O)(=O)[N-]S(=O)(=O)C(F)(F)F | 0 | validation |
| 144209864 | CCCC[N+]1(CCCCC1)C.F[P-](F)(F)(F)(F)F | 0 | test |
| 144209863 | CCCC[N+]1(CCCCC1)C.C(F)(F)(F)S(=O)(=O)[O-] | 0 | train |
| 144209862 | CCCC[N+]1(CCCCC1)C.[Br-] | 0 | train |
| 144209861 | [B-](F)(F)(F)F.CCCC[N+]1(CCCCC1)C | 0 | validation |
| 144209860 | CCCC[N+]1(CCCCC1)C.C(F)(F)(F)S(=O)(=O)[N-]S(=O)(=O)C(F)(F)F | 0 | test |
| 144209859 | CCCCCCCCCCCCCCN1C=C[N+](=C1)C.F[P-](F)(F)(F)(F)F | 0 | train |
| 144209858 | CCCCCCCCCCCCCCN1C=C[N+](=C1)C.[Cl-] | 1 | train |
| 144209857 | CCCN1C=C[N+](=C1)C.F[P-](F)(F)(F)(F)F | 0 | validation |
| 144209856 | CCCCCCCCCCCCCCN1C=C[N+](=C1)C.C(F)(F)(F)S(=O)(=O)[N-]S(=O)(=O)C(F)(F)F | 1 | test |
| 144209855 | CCCN1CN(C=C1)C.Cl | 0 | train |
| 144209854 | [B-](F)(F)(F)F.CCCN1C=C[N+](=C1)C | 0 | train |
| 144209853 | CCCN1CN(C=C1)C.Br | 0 | validation |
| 144209852 | CCCN1C=C[N+](=C1)C.[I-] | 0 | test |
| 144209851 | CCCCCCCCN1CN(C=C1)C.Br | 0 | train |
| 144209850 | CCCCCCCCCCCCCCCCCCN1C=C[N+](=C1)C.F[P-](F)(F)(F)(F)F | 1 | train |
| 144209849 | [B-](F)(F)(F)F.C[N+]1=CN(C=C1)CCO | 0 | validation |
| 144209848 | [B-](F)(F)(F)F.CCCCCCN1C=C[N+](=C1)C | 0 | test |
| 144209847 | CCCCCCN1C=C[N+](=C1)C.C(F)(F)(F)S(=O)(=O)[N-]S(=O)(=O)C(F)(F)F | 0 | train |
| 144209846 | CCCCCCCCCCCCCCCCN1C=C[N+](=C1)C.C(F)(F)(F)S(=O)(=O)[N-]S(=O)(=O)C(F)(F)F | 1 | train |
| 144209845 | [B-](F)(F)(F)F.CCCCCCCCCCCCCCCCN1C=C[N+](=C1)C | 1 | validation |
| 144209844 | CCCCCCCCCCCCCCCCN1C=C[N+](=C1)C.F[P-](F)(F)(F)(F)F | 0 | test |

| | | | |
|---|---|---|---|
| 144209843 | CCN1C=C[N+](=C1)C.CC1=CC=C(C=C1)S(=O)(=O)[O-] | 0 | train |
| 144209842 | [B-](F)(F)(F)F.CCN1C=C[N+](=C1)C | 0 | train |
| 144209841 | CCN1C=C[N+](=C1)C.[I-] | 0 | validation |
| 144209840 | CCN1C=C[N+](=C1)C.OS(=O)(=O)[O-] | 0 | test |
| 144209839 | CCN1C=C[N+](=C1)C.COS(=O)(=O)[O-] | 0 | train |
| 144209838 | CCN1C=C[N+](=C1)C.CS(=O)(=O)[O-] | 0 | train |
| 144209837 | CCN1C=C[N+](=C1)C.CCOS(=O)(=O)[O-] | 0 | validation |
| 144209836 | CCN1C=C[N+](=C1)C.CCOP(=O)([O-])OCC | 1 | test |
| 144209835 | CCN1C=C[N+](=C1C)C.C(F)(F)(F)S(=O)(=O)[O-] | 0 | train |
| 144209834 | [B-](F)(F)(F)F.CCN1C=C[N+](=C1C)C | 0 | train |
| 144209833 | CCN1C=C[N+](=C1)C.[Br-] | 0 | validation |
| 144209832 | CCN1C=C[N+](=C1C)C.F[P-](F)(F)(F)(F)F | 0 | test |
| 144209831 | CCN1C=CN(C1C)C.Br | 0 | train |
| 144209830 | CCCCCCCCCCCCN1C=C[N+](=C1)C.[I-] | 0 | train |
| 144209829 | CCN1C=C[N+](=C1C)C.C(F)(F)(F)S(=O)(=O)[N-]S(=O)(=O)C(F)(F)F | 0 | validation |
| 144209828 | CCCCCCCCCCCCN1C=C[N+](=C1)C.C(F)(F)(F)S(=O)(=O)[O-] | 1 | test |
| 144209827 | [B-](F)(F)(F)F.CCCCCCCCCCCCN1C=C[N+](=C1)C | 1 | train |
| 144209826 | CCCCCCCCCCCCN1C=C[N+](=C1)C.[Br-] | 0 | train |
| 144209825 | CCCCCCCCCCN1C=C[N+](=C1C)CCCCCCCCCC.[Cl-] | 1 | validation |
| 144209824 | CCCN1C=C[N+](=C1C)C.[I-] | 0 | test |
| 144209823 | CCCN1C=C[N+](=C1C)C.C(F)(F)(F)S(=O)(=O)[N-]S(=O)(=O)C(F)(F)F | 0 | train |
| 144209822 | CCCCCCCCCCN1C=C[N+](=C1)C.F[P-](F)(F)(F)(F)F | 0 | train |
| 144209821 | CCCCCCCCCCN1C=C[N+](=C1)C.[Cl-] | 0 | validation |
| 144209820 | CCCCCCCCCCN1C=C[N+](=C1)C.[Br-] | 0 | test |
| 144209819 | [B-](F)(F)(F)F.CCCCN1C=C[N+](=C1)C | 0 | train |
| 144209818 | CCCCN1C=C[N+](=C1)C.Cl[Fe-](Cl)(Cl)Cl | 0 | train |
| 144209817 | CCCCN1C=C[N+](=C1)C.C(F)(F)(F)S(=O)(=O)[O-] | 0 | validation |
| 144209816 | CCCCN1C=C[N+](=C1)C.[I-] | 0 | test |
| 144209815 | CCCCN1C=C[N+](=C1)C.C(F)(F)(F)S(=O)(=O)[N-]S(=O)(=O)C(F)(F)F | 1 | train |
| 144209814 | CCCCN1C=C[N+](=C1C)C.C(F)(F)(F)S(=O)(=O)[O-] | 0 | train |
| 144209813 | CCCC[P+](CC)(CCCC)CCCC.CCOP(=O)([O-])OCC | 0 | validation |
| 144209812 | CCCCCCCCCCCCCC[P+](CCCCCC)(CCCCCC)CCCCCC.C(F)(F)(F)S(=O)(=O)[N-]S(=O)(=O)C(F)(F)F | 0 | test |
| 144209811 | CCCCCCCCCCCCCC[P+](CCCCCC)(CCCCCC)CCCCCC.C(=[N-])=NC#N | 1 | train |
| 144209810 | CCCSSCC=C | 0 | train |
| 144209808 | C[C@](CC1=CC(=C(C=C1)O)O)(C(=O)O)N | 0 | test |
| 144209807 | CC(C)OC(=O)C(=C)C | 0 | train |
| 144209806 | C[C@@H]1CC[C@H]([C@@H](C1)O)C(C)C | 0 | train |
| 144209805 | C1C(O1)CBr | 0 | validation |
| 144209804 | CC[C@H](C)COC(=O)/C=C/C1=CC=C(C=C1)N=CC2=CC=C(C=C2)OC | 1 | test |
| 144209803 | CCCC[N+]1(CCCC1)C.C(F)(F)(F)S(=O)(=O)[O-] | 0 | train |
| 144209802 | C[C@]12CC[C@H]3[C@H]([C@@H]1CC[C@@H]2O)[C@@H](CC4=C3C=CC(=C4)O)CCCCCCCCCS(=O)CCCC(C(F)(F)F)(F)F | 0 | train |
| 144209801 | C1CCN(CC1)CCOC2=CC=C(C=C2)C(=O)C3=C(SC4=C3C=CC(=C4)O)C5=CC=C(C=C5)O | 1 | validation |
| 144209800 | C[C@H]1[C@@H]([C@H]([C@H]([C@@H](O1)OC[C@@H]2[C@H]([C@@H]([C@H]([C@@H](O2)OC3=C(OC4=CC(=CC(=C4C3=O)O)O)C5=CC(=C(C=C5)O)O)O)O)O)O)O)O | 0 | test |
| 144209799 | CCCCN(CCCC)C(=S)[S-].CCCCN(CCCC)C(=S)[S-].[Zn+2] | 1 | train |
| 144209798 | CC(=O)O[Sn](C1=CC=CC=C1)(C2=CC=CC=C2)C3=CC=CC=C3 | 1 | train |
| 144209797 | [Cl-].[Cl-].[Sn+2] | 0 | validation |
| 144209796 | C1=CC(=C(C=C1C2=CC(=C(C=C2)N)N)N)N.Cl.Cl.Cl.Cl | 0 | test |
| 144209795 | O=[Se]=O | 0 | train |
| 144209794 | C1=CC(=C(C(=C1C2=CC(=C(C(=C2)Br)Br)Br)Br)Br)Br | 1 | train |
| 144209793 | [N+](=O)([O-])[O-].[Na+] | 0 | validation |
| 144209792 | O.O.O.O.O.O.[O-]S(=O)(=O)[O-].[Ni+2] | 0 | test |
| 144209791 | CCCCN(CCCC)C(=S)[S-].CCCCN(CCCC)C(=S)[S-].[Ni+2] | 1 | train |
| 144209790 | CC1=C2CCC3=C2C(=CC4=C3C=CC5=CC=CC=C54)C=C1 | 0 | train |
| 144209789 | C1=NC2=C(N1)C(=S)N=CN2 | 1 | validation |
| 144209788 | C1CCCNCCC1 | 0 | test |
| 144209787 | CN(C)C(=S)[S-].CN(C)C(=S)[S-].CN(C)C(=S)[S-].[Fe+3] | 1 | train |
| 144209786 | C(CNC(=S)[S-])NC(=S)[S-].[Na+].[Na+] | 0 | train |
| 144209785 | CNC.CN(C)C(=S)S | 1 | validation |
| 144209784 | C1=CC(=CC=C1N)N=NC2=CC=C(C=C2)N | 0 | test |

| | | | |
|---|---|---|---|
| 144209783 | [Cl-].[Cl-].[Ca+2] | 0 | train |
| 144209782 | C1=CC(=CC=C1C2=CC=C(C=C2)N)N.Cl.Cl | 0 | train |
| 144209781 | C12C(O[Sb]3OC(C(O[Sb](O1)OC2=O)C(=O)[O-])C(=O)O3)C(=O)[O-].O.O.O.[K+].[K+] | 0 | validation |
| 144209780 | C1=CC=C2C(=C1)C(=O)C3=C(C2=O)C(=C(C=C3Br)Br)N | 1 | test |
| 144209779 | [H+].[B-](F)(F)(F)F | 0 | train |
| 144209778 | [O-]S(=O)(=O)[O-].[Fe+2] | 0 | train |
| 144209777 | CC(C)COC=C | 0 | validation |
| 144209776 | CCO[Si](CCCSSSSCCC[Si](OCC)(OCC)OCC)(OCC)OCC | 0 | test |
| 144209775 | CC(C)(C1=CC(=C(C(=C1)Br)OCC=C)Br)C2=CC(=C(C(=C2)Br)OCC=C)Br | 0 | train |
| 144209774 | C1=CC=C2C(=C1)C(=O)C3=C(C=C(C(=C3C2=O)N)S(=O)(=O)[O-])Br.[Na+] | 0 | train |
| 144209773 | CO[Si](C=C)(OC)OC | 0 | validation |
| 144209772 | C1=CC(=CC=C1N)S(=O)(=O)[O-].[Na+] | 0 | test |
| 144209771 | CCCCCCCCCCCCCCCCCC(=O)OCC(CO)O | 0 | train |
| 144209770 | C[N+](C)(C)C.[OH-] | 0 | train |
| 144209769 | CC(C)(C1=CC=CC=C1)C2=CC(=C(C(=C2)N3N=C4C=CC=CC4=N3)O)C(C)(C)C5=CC=CC=C5 | 0 | validation |
| 144209768 | CCCCCCCCCCCCCC[N+](C)(C)CC1=CC=CC=C1.[Cl-] | 1 | test |
| 144209767 | C=CCOCC(CS(=O)(=O)[O-])O.[Na+] | 0 | train |
| 144209766 | C[N+](C)(C)CCOC(=O)C=C.[Cl-] | 0 | train |
| 144209765 | CC(=C)C(=O)OCCC[Si](Cl)(Cl)Cl | 0 | validation |
| 144209764 | C1=C2C=C(C=C(C2=C(C=C1S(=O)(=O)[O-])N)O)S(=O)(=O)O.[Na+] | 0 | test |
| 144209763 | CC(C)(CS(=O)(=O)[O-])NC(=O)C=C.[Na+] | 0 | train |
| 144209762 | CC(=C)C(=O)OCC[N+](C)(C)C.[Cl-] | 1 | train |
| 144209761 | C[N+](C)(C)CC(CCl)O.[Cl-] | 0 | validation |
| 144209760 | COP(=S)(OC)Cl | 0 | test |
| 144209759 | CCCCCCCCCCCCOS(=O)(=O)[O-].[NH4+] | 0 | train |
| 144209758 | C1=CC(=CC=C1O)S(=O)(=O)[O-].[Na+] | 0 | train |
| 144209757 | CCCCCCCCCCCCCCCCCC#N | 0 | validation |
| 144209756 | CCCCCCCCCCCCCCCC(=O)OC(C)C | 0 | test |
| 144209755 | CC(=O)[O-].CC(=O)[O-].[Mg+2] | 0 | train |
| 144209754 | CC(=O)NC1=CC=C(C=C1)S(=O)(=O)Cl | 0 | train |
| 144209753 | CCCCCCCCCCCCCCCC[N+](C)(C)C.[Cl-] | 0 | validation |
| 144209752 | CC(=C)CC(C)(C)C | 0 | test |
| 144209751 | C1=CC(=C(C=C1S(=O)(=O)O)C(=O)O)C(=O)O | 0 | train |
| 144209750 | C1=CC=C(C=C1)[Si](C2=CC=CC=C2)(Cl)Cl | 0 | train |
| 144209749 | CC(C)OC(C)C | 0 | validation |
| 144209748 | CCCCCCCCCCCCCCCC(=O)O | 0 | test |
| 144209747 | CC1=NC(=NC(=N1)OC)N(C)C(=O)NS(=O)(=O)C2=CC=CC=C2C(=O)OC | 0 | train |
| 144209746 | C1=CC=C2C(=C1)C=CC3=C2C=CC4=CC=CC=C43 | 1 | train |
| 144209745 | COC1=CC=C(C=C1)/C=C/C2=CC(=CC(=O)O2)OC | 1 | validation |
| 144209744 | C[C@H](CCCC(C)C)[C@H]1CC[C@@H]¥2[C@@]1(CCC/C2=C¥C=C/3¥C[C@H](CCC3=C)O)C | 1 | test |
| 144209743 | C1=CC=C2C(=C1)C(=O)/C(=C¥3/C(=O)C4=CC=CC=C4N3)/N2 | 0 | train |
| 144209742 | CC(C)(C)CC(C)(C)C1=CC=C(C=C1)OCCO | 0 | train |
| 144209741 | C(=C(Cl)Cl)Cl | 0 | validation |
| 144209740 | C1C=CC=[C-]1.C1C=CC=[C-]1.[Cl-].[Cl-].[Ti+4] | 0 | test |
| 144209739 | C=CC(=O)OCCOCCOCCOCCOC(=O)C=C | 1 | train |
| 144209738 | CC(C)(C1=CC(=C(C(=C1)Br)OCC(CBr)Br)Br)C2=CC(=C(C(=C2)Br)OCC(CBr)Br)Br | 0 | train |
| 144209735 | OS(=O)(=O)[O-].[Na+] | 0 | train |
| 144209734 | O.O.[O-][Cr](=O)(=O)O[Cr](=O)(=O)[O-].[Na+].[Na+] | 0 | train |
| 144209733 | C[C@H](CCC(=O)[O-])[C@H]1CC[C@@H]2[C@@]1([C@H](C[C@H]3[C@H]2[C@@H](C[C@H]4[C@@]3(CC[C@H](C4)O)C)O)O)C.[Na+] | 0 | validation |
| 144209732 | [O-]Br(=O)=O.[Na+] | 0 | test |
| 144209731 | CC(C)(C)NC(=O)[C@@H]1C[C@@H]2CCCC[C@@H]2CN1C[C@H]([C@H](CC3=CC=CC=C3)NC(=O)[C@H](CC(=O)N)NC(=O)C4=NC5=CC=CC=C5C=C4)O.CS(=O)(=O)O | 1 | train |
| 144209730 | CCCSC(=O)Cl | 0 | train |
| 144209729 | CC(COC(=O)C(=C)C)O | 0 | validation |
| 144209728 | P(Cl)(Cl)(Cl)(Cl)Cl | 0 | test |
| 144209727 | C=CC1=CC=C(C=C1)C=C | 0 | train |
| 144209726 | CCCCCCCC/C=C¥CCCCCCCC(=O)N(CCO)CCO | 0 | train |
| 144209725 | [CH-]1C=CC=C1.[CH-]1C=CC=C1.[Ni+2] | 0 | validation |

| | | | |
|---|---|---|---|
| 144209724 | C(Cl)Cl | 0 | test |
| 144209723 | CN(C)C1=CC2=C(C=C1)N=C3C=CC(=[N+](C)C)C=C3S2.O.O.O.[Cl-] | 1 | train |
| 144209722 | C[Hg]Cl | 1 | train |
| 144209721 | CO | 0 | validation |
| 144209720 | C1=CC(=CC=C1C[C@@H](C(=O)O)N)N(CCCl)CCCl | 0 | test |
| 144209719 | CN(C)C1=CC=C(C=C1)C(=C2C=CC(=[N+](C)C)C=C2)C3=CC=CC=C3.CN(C)C1=CC=C(C=C1)C(=C2C=CC(=[N+](C)C)C=C2)C3=CC=CC=C3.C(=O)(C(=O)O)O.C(=O)(C(=O)[O-])O.C(=O)(C(=O)[O-])O | 1 | train |
| 144209718 | C(CS(=O)(=O)O)N | 1 | train |
| 144209717 | C1=CC(=CC=C1C(=O)O)S(=O)(=O)N(Cl)Cl | 0 | validation |
| 144209716 | C/C=C/1¥C(=O)C[C@@H]2[C@@]1(CC[C@H]3[C@H]2CCC4=CC(=O)CC[C@]34C)C | 1 | test |
| 144209715 | C(=O)C=O | 0 | train |
| 144209713 | C1=C(C=C(C(=C1O)O)O)C(=O)O | 0 | validation |
| 144209712 | [CH-]1C=CC=C1.[CH-]1C=CC=C1.[Fe+2] | 0 | test |
| 144209711 | CCOC(=O)C=C | 0 | train |
| 144209710 | CCOC(=O)C | 0 | train |
| 144209709 | CC[N+]1=C2C=C(C=CC2=C3C=CC(=CC3=C1C4=CC=CC=C4)N)N.[Br-] | 0 | validation |
| 144209708 | CCO | 0 | test |
| 144209707 | CCCCCCCCCCCOC(=O)C1=CC=CC=C1C(=O)OCCCCCCCCCCC | 0 | train |
| 144209706 | CC1=C(C=CC(=C1)C2=CC(=C(C=C2)N=NC3=C(C4=CC=CC=C4C(=C3)S(=O)(=O)[O-])N)C)N=NC5=C(C6=CC=CC=C6C(=C5)S(=O)(=O)[O-])N.[Na+].[Na+] | 0 | train |
| 144209705 | C[C@@H]1CC[C@@]2([C@H]([C@H]3[C@@H](O2)[C@H]([C@@H]4[C@@]3(CC[C@H]5[C@H]4CC[C@@H]6[C@@]5(C[C@H]([C@@H](C6)O[C@H]7[C@@H]([C@H]([C@H]([C@H](O7)CO)O[C@H]8[C@@H]([C@H]([C@@H]([C@H](O8)CO)O)O[C@H]9[C@@H]([C@H]([C@@H](CO9)O)O)O)O[C@H]2[C@@H]([C@H]([C@@H]([C@H](O2)CO)O)O[C@H]2[C@@H]([C@H]([C@@H]([C@H](O2)CO)O)O)O)O)O)O)O)O)C)C)O)C)OC1 | 1 | validation |
| 144209704 | CCNCC | 0 | test |
| 144209703 | C1=CC=C2C(=C1)C=CC3=CC4=C(C=CC5=CC=CC=C54)C=C32 | 1 | train |
| 144209702 | C1=CC=C2C(=C1)C=CC(=N2)C3C(=O)C4=CC=CC=C4C3=O | 1 | train |
| 144209701 | CC(C)C1=CC=CC=C1 | 0 | validation |
| 144209700 | [CH-]1C=CC=C1.[CH-]1C=CC=C1.[Co+2] | 0 | test |
| 144209699 | O.O.O.O.O.O.O.[O-]S(=O)(=O)[O-].[Co+2] | 0 | train |
| 144209698 | [NH2-].[NH2-].Cl[Pt+2]Cl | 0 | train |
| 144209697 | CC1=CC(=C2C(=C1)C(=O)C3=C(C2=O)C(=CC=C3)O)O | 1 | validation |
| 144209696 | CC(=O)[O-].CC(=O)[O-].CC(=O)[O-].[Cr+3] | 0 | test |
| 144209695 | CCC(CC(C(C(C(C(C)Cl)Cl)Cl)Cl)Cl)Cl | 1 | train |
| 144209694 | CCC(CCC(C(CC(C(C(CCl)Cl)Cl)Cl)Cl)Cl)Cl | 0 | train |
| 144209693 | C1[C@H]([C@H]([C@@H](C[C@@]1(C(=O)O)O)OC(=O)C=CC2=CC(=C(C=C2)O)O)O)O | 0 | validation |
| 144209692 | C(Cl)(Cl)Cl | 0 | test |
| 144209690 | O=[Cd] | 0 | train |
| 144209689 | CCCC=O | 0 | validation |
| 144209688 | B(F)(F)F.O.O | 0 | test |
| 144209687 | [CH-]1C=CC=C1.[CH-]1C=CC=C1.Cl[V]Cl | 1 | train |
| 144209686 | CC(C)(C)CC(C)(C)C1=CC=C(C=C1)OCCOCC[N+](C)(C)CC2=CC=CC=C2.[Cl-] | 0 | train |
| 144209685 | C1=CC=CC=C1 | 0 | validation |
| 144209684 | C1=CC=C(C=C1)N=NC2=C(C=C(C=C2)N)N.Cl | 1 | test |
| 144209682 | C[C@H]1/C=C/C=C/C=C/C=C/C=C/C=C/C=C/[C@@H](C[C@H]2[C@@H]([C@H](C[C@](O2)(C[C@H](C[C@H]([C@@H](CC[C@H](C[C@H](CC(=O)O[C@H]([C@@H]([C@@H]1O)C)C)O)O)O)O)O)O)O)C(=O)O)O[C@H]3[C@H]([C@H]([C@@H]([C@H](O3)C)O)N)O | 1 | train |
| 144209681 | C[C@H]1[C@H]([C@H](C[C@@H](O1)O[C@H]2C[C@@](CC3=C(C4=C(C(=C23)O)C(=O)C5=C(C4=O)C=CC=C5OC)O)(C(=O)CO)O)N)O.Cl | 0 | validation |
| 144209680 | C[C@@H]1[C@@H](C(=O)N[C@@H](C(=O)N2CCC[C@H]2C(=O)N(CC(=O)N([C@H](C(=O)O1)C(C)C)C)C)C(C)C)NC(=O)C3=C4C(=C(C=C3)C)OC5=C(C(=O)C(=C(C5=N4)C(=O)N[C@H]6[C@H](OC(=O)[C@@H](N(C(=O)CN(C(=O)[C@@H]7CCCN7C(=O)[C@H](NC6=O)C(C)C)C)C)C(C)C)C)N)C | 0 | test |
| 144209679 | CC#N | 0 | train |
| 144209678 | CC(=O)C | 0 | train |
| 144209677 | CC=O | 1 | validation |
| 144209676 | C1=CC(=CC=C1C=O)C(=O)O | 0 | test |
| 144209675 | COC1=C(C=CC(=C1)C2=CC(=C(C=C2)N=C=O)OC)N=C=O | 0 | train |
| 144209674 | C1=CC(=CC=C1C=CC(=O)O)C=CC(=O)O | 0 | train |
| 144209673 | CC(C)O | 0 | validation |

| | | | |
|---|---|---|---|
| 144209672 | CC1=C(C2=C(C=C1)C(=O)C3=CC=CC=C3C2=O)[N+](=O)[O-] | 0 | test |
| 144209671 | CCCCCCCCC/C=C/CC1CC(=O)OC1=O | 0 | train |
| 144209670 | CC1=CC=C(O1)C | 0 | train |
| 144209669 | C1=CC(=C(C=C1O)O)C(=O)O | 0 | validation |
| 144209668 | C1=C(C(=CC(=C1Cl)Cl)Cl)C2=CC(=C(C=C2Cl)Cl)Cl | 1 | test |
| 144209667 | C1=C(C(=CC(=C1Br)Br)Br)OC2=CC(=C(C=C2Br)Br)Br | 0 | train |
| 144209666 | CC1=C(C2=C(C=C1)N=C(S2)C3=CC=C(C=C3)N)S(=O)(=O)O | 1 | train |
| 144209665 | CC1=CC(NC2=CC=CC=C12)(C)C | 1 | validation |
| 144209664 | C(CCl)Cl | 0 | test |
| 144209663 | C(C(Cl)Cl)Cl | 0 | train |
| 144209662 | CC(=O)C=CC1=CC2=C(C=C1)OCO2 | 0 | train |
| 144209661 | CCOP(=O)(OCC)OCC | 0 | validation |
| 144209660 | C=CN1CCCC1=O | 0 | test |
| 144209659 | [N-]=[N+]=[N-].[Na+] | 1 | train |
| 144209658 | C(C[N+](=O)[O-])C(=O)O | 0 | train |
| 144209657 | C1=CC(=CC=C1N)Br | 1 | validation |
| 144209656 | CN1C(=O)CN=C(C2=C1C=CC(=C2)Cl)C3=CC=CC=C3 | 0 | test |
| 144209655 | CCCC[Sn](Cl)(Cl)Cl | 1 | train |
| 144209654 | CC(C)(C1=CC=CC=C1)C2=CC(=C(C=C2)O)C(C)(C)C3=CC=CC=C3 | 1 | train |
| 144209653 | CC(C(=O)O)O | 0 | validation |
| 144209652 | CC(C)C(C(C)(C)COC(=O)C(C)C)OC(=O)C(C)C | 0 | test |
| 144209651 | CCOC(=O)C(=C)C#N | 0 | train |
| 144209650 | CCCCCCCC | 0 | train |
| 144209649 | CC(C)N=C=NC(C)C | 0 | validation |
| 144209648 | C1COCCN1 | 1 | test |
| 144209647 | C1(=NC(=NC(=N1)Cl)Cl)Cl | 1 | train |
| 144209646 | C(CC(=O)O)CC(=O)O | 1 | train |
| 144209645 | C1=CC=C(C=C1)CO | 0 | validation |
| 144209644 | C1CC2C(C(C1O2)C(=O)O)C(=O)O | 1 | test |
| 144209643 | CCC1=CC=CC(=C1N(COCC)C(=O)CCl)C | 1 | train |
| 144209642 | C1=CC(=CC=C1[N+](=O)[O-])O | 0 | train |
| 144209641 | COC1=C(C=CC(=C1)C2=CC(=C(C=C2)N)OC)N | 1 | validation |
| 144209640 | C1=CC(=CC=C1NC(=O)C2=CC(=CC(=C2O)Br)Br)Br | 0 | test |
| 144209639 | CC1=C(C(CCC1)(C)C)/C=C/C(=C/C=C/C(=C/CO)/C)/C | 1 | train |
| 144209638 | C(COCCOCCO)O | 0 | train |
| 144209637 | C=CCC1=CC2=C(C=C1)OCO2 | 0 | validation |
| 144209636 | C1=CC(=CC=C1[N+](=O)[O-])OC2=C(C=C(C=C2)Cl)Cl | 1 | test |
| 144209635 | COC1=CC=C(C=C1)N | 0 | train |
| 144209634 | C1CCC(CC1)NSC2=NC3=CC=CC=C3S2 | 1 | train |
| 144209633 | CC1=CC(=CC=C1)[N+](=O)[O-] | 0 | validation |
| 144209632 | CC(C)CC(C)CC(CC(C)C)O | 0 | test |
| 144209631 | CC=CC1=CC(=C(C=C1)O)OC | 1 | train |
| 144209630 | CCC(=O)[O-].CCC(=O)[O-].[Ca+2] | 0 | train |
| 144209629 | CCOCC(=O)O | 0 | validation |
| 144209628 | C(/C=C/CO)O | 1 | test |
| 144209627 | CC(C)COC(=O)C1=CC=CC=C1C(=O)OCC(C)C | 0 | train |
| 144209626 | C[Si](C)(C)N[Si](C)(C)C | 1 | train |
| 144209625 | C1=CC=C2C(=C1)C=CC(=O)O2 | 1 | validation |
| 144209624 | CCCCCCCCC(=O)O | 0 | test |
| 144209623 | C1=CC=C(C=C1)C(=O)C2=CC=CC=C2 | 1 | train |
| 144209622 | CCOP(=O)(OCC)OC1=CC=C(C=C1)[N+](=O)[O-] | 0 | train |
| 144209621 | CC1=CC(=O)[N-]S(=O)(=O)O1.[K+] | 0 | validation |
| 144209620 | CC(=O)C1=CC=CC=C1 | 1 | test |
| 144209619 | CCCCC(CC)COC(=O)C1=CC=C(C=C1)N(C)C | 1 | train |
| 144209618 | CCCCCCOC(=O)C1=CC=CC=C1C(=O)OCCCCCC | 1 | train |
| 144209617 | C(=C/Cl)¥Cl | 0 | validation |
| 144209616 | C1=CNC(=S)NC1=O | 1 | test |
| 144209615 | C1=CC(=CC(=C1)O)O | 0 | train |
| 144209614 | CN(C)C1=CC=C(C=C1)C(=O)C2=CC=C(C=C2)N(C)C | 1 | train |
| 144209613 | C1=CC=C(C=C1)C2=CC=CC=C2O | 0 | validation |
| 144209612 | CCOC(=O)C(C)(C)OC1=CC=C(C=C1)Cl | 0 | test |
| 144209611 | CC1=NC=C(N1CCO)[N+](=O)[O-] | 0 | train |

| | | | |
|---|---|---|---|
| 144209610 | CN1CCOCC1 | 0 | train |
| 144209609 | CC(C)CON=O | 0 | validation |
| 144209608 | CC(C)(C1=CC(=CC=C1)C(C)(C)N=C=O)N=C=O | 0 | test |
| 144209607 | CCC1=NC=CC(=C1)C(=S)N | 0 | train |
| 144209606 | CC(CCO)O | 1 | train |
| 144209605 | CC/C(=C(/CC)¥C1=CC=C(C=C1)O)/C2=CC=C(C=C2)O | 1 | validation |
| 144209604 | CC(C)CC(=O)CC(C)C | 1 | test |
| 144209603 | C(C(=O)O)C(CC(=O)O)(C(=O)O)O | 0 | train |
| 144209602 | CCCCOCCOCCO | 0 | train |
| 144209601 | C1=CC=C(C=C1)C(=O)O | 1 | validation |
| 144209600 | CCOS(=O)(=O)OCC | 0 | test |
| 144209599 | C1=NC2=C(N1)C(=S)N=C(N2)N | 0 | train |
| 144209598 | C1CC2=CC=CC3=C2C1=CC=C3 | 0 | train |
| 144209597 | CCCCOCCO | 0 | validation |
| 144209596 | CCN1C2=C(C=C(C=C2)N)C3=CC=CC=C31 | 1 | test |
| 144209595 | C1=CC=C2C(=C1)NC(=N2)C3=CSC=N3 | 1 | train |
| 144209594 | C1=CC(=CC=C1S(=O)(=O)C2=CC(=C(C=C2Cl)Cl)Cl)Cl | 1 | train |
| 144209593 | CO[C@H]1[C@@H](C[C@@H]2CN3CCC4=C([C@H]3C[C@@H]2[C@@H]1C(=O)OC)NC5=C4C=CC(=C5)OC)OC(=O)C6=CC(=C(C(=C6)OC)OC)OC | 1 | validation |
| 144209592 | CC1=CC=C(C=C1)C=C | 0 | test |
| 144209591 | CC1=CC=CC=C1[N+](=O)[O-] | 0 | train |
| 144209590 | C1=CC(=CC=C1SC2=CC(=C(C=C2Cl)Cl)Cl)Cl | 1 | train |
| 144209589 | CC1=CC=C(C=C1)C(=O)OC | 0 | validation |
| 144209588 | CCC(C)(CCCC(C)C)O | 0 | test |
| 144209587 | C(I)(I)I | 0 | train |
| 144209586 | C1CC2C(O2)CC1COC(=O)C3CCC4C(C3)O4 | 0 | train |
| 144209585 | COC1=CC=C(C=C1)CC=C | 0 | validation |
| 144209584 | CC(=CCC/C(=C/CO)/C)C | 1 | test |
| 144209583 | CC(C)(C1=CC=CC=C1)OOC(C)(C)C2=CC=CC=C2 | 1 | train |
| 144209582 | CC1(C(=O)N(C(=O)N1Cl)Cl)C | 0 | train |
| 144209581 | CCOP(=S)(OCC)OC1=NC(=C(C=C1Cl)Cl)Cl | 1 | validation |
| 144209580 | CCCC(=O)O | 1 | test |
| 144209579 | C1=CC=C2C3=C4C(=CC=C3)C5=CC=CC=C5C4=CC2=C1 | 1 | train |
| 144209578 | C1CCC(CC1)N | 0 | train |
| 144209577 | CC1=CC2=C(C=C1)N=CC=C2 | 0 | validation |
| 144209576 | CCNC1=NC(=NC(=N1)Cl)NCC | 0 | test |
| 144209575 | C1=C(SC(=N1)N)[N+](=O)[O-] | 0 | train |
| 144209574 | C1=CC(=C(C=C1C(=O)C2=C(C=C(C=C2)O)O)O)O | 0 | train |
| 144209573 | CN1C2=C(C(=O)N(C1=O)C)NC=N2 | 0 | validation |
| 144209572 | CC(C)(C)C1=CC(=C(C(=C1)C(C)(C)C)O)C(C)(C)C | 1 | test |
| 144209571 | C1=CC(=C(C(=C1)O)O)O | 0 | train |
| 144209570 | CC1=C(C=CC(=C1)CC2=CC(=C(C=C2)N)C)N | 1 | train |
| 144209569 | C1=CC=C(C(=C1)N)[N+](=O)[O-] | 0 | validation |
| 144209568 | C1=CC(=CN=C1)CCl.Cl | 0 | test |
| 144209567 | COC(=O)C1=CC=C(C=C1)C=O | 0 | train |
| 144209566 | C1=CC=C(C=C1)OC2=CC=CC(=C2)C=O | 0 | train |
| 144209565 | COC1=CC=C(C=C1)O | 0 | validation |
| 144209564 | CCCCOCNC(=O)C=C | 1 | test |
| 144209563 | C1C2C(COS(=O)O1)C3(C(=C(C2(C3(Cl)Cl)Cl)Cl)Cl)Cl | 1 | train |
| 144209562 | CC1=CC=C(C=C1)S(=O)(=O)O | 1 | train |
| 144209561 | C(C(=O)O)(Cl)Cl | 0 | validation |
| 144209560 | C1(=O)NC(=O)NC(=O)N1 | 0 | test |
| 144209559 | COC1=CC=C(C=C1)C(=C(C2=CC=C(C=C2)OC)Cl)C3=CC=C(C=C3)OC | 1 | train |
| 144209558 | CCN(CC)C(=O)SCC1=CC=C(C=C1)Cl | 1 | train |
| 144209557 | C1=CC(=CC=C1C2=CC=C(C=C2)N)N | 1 | validation |
| 144209556 | CSC1=CC=C(C=C1)Cl | 0 | test |
| 144209555 | COC1=C2C=CC(=O)OC2=CC3=C1C=CO3 | 1 | train |
| 144209554 | C1CCN(C1)N=O | 0 | train |
| 144209553 | C(=S)(N)NNC(=S)N | 0 | validation |
| 144209552 | CCCCC(CC)COC(=O)C1=CC=C(C=C1)O | 0 | test |
| 144209551 | C([N+](=O)[O-])([N+](=O)[O-])([N+](=O)[O-])[N+](=O)[O-] | 0 | train |
| 144209550 | CCCCC1(C(=O)N(N(C1=O)C2=CC=CC=C2)C3=CC=CC=C3)COC(=O)CCC(=O)O | 0 | train |

| | | | |
|---|---|---|---|
| 144209549 | CCC(C)(C)C1=CC=C(C=C1)O | 0 | validation |
| 144209548 | CN1CCCC1=O | 0 | test |
| 144209547 | CCCCCC1=CC(=CC(=C1)O)O | 1 | train |
| 144209546 | C[C@H](/C=C/[C@H](C)C(C)C)[C@H]1CC[C@@H]¥2[C@@]1(CCC/C2=C¥C=C/3¥C[C@H](CCC3=C)O)C | 0 | train |
| 144209545 | COC1=C(C=C(C=C1)CC=C)OC | 0 | validation |
| 144209544 | CC(C)CCCCCOC(=O)C=C | 0 | test |
| 144209543 | C1=CC(=CC=C1O)O | 1 | train |
| 144209542 | COCCO[Si](C=C)(OCCOC)OCCOC | 0 | train |
| 144209541 | CCOP(=S)(OCC)SCCSCC | 1 | validation |
| 144209540 | C1=CC(=CC=C1C(=O)O)O | 0 | test |
| 144209539 | C1=C(C=C(C(=C1Cl)N)Cl)[N+](=O)[O-] | 0 | train |
| 144209538 | COCCOCCOC | 1 | train |
| 144209537 | C1(C(C2(C(=C(C1(C2(Cl)Cl)Cl)Cl)Cl)Cl)C(=O)O)C(=O)O | 1 | validation |
| 144209536 | CC(C)NC1=NC(=NC(=N1)SC)NC(C)C | 1 | test |
| 144209535 | CN(C)C1=CC=C(C=C1)C(=N)C2=CC=C(C=C2)N(C)C.Cl | 0 | train |
| 144209534 | CCCCN(CCCC)SN(C)C(=O)OC1=CC=CC2=C1OC(C2)(C)C | 1 | train |
| 144209533 | C1=C(C(=O)NC(=O)N1)F | 1 | validation |
| 144209532 | CCCCN(CCCC)N=O | 0 | test |
| 144209531 | C1C[C@@H](O[C@@H]1CO)N2C=NC3=C2NC=NC3=O | 0 | train |
| 144209530 | COC1=C(C=C(C(=C1)O)C(=O)C2=CC=CC=C2)S(=O)(=O)O | 0 | train |
| 144209529 | CC(C)(C)C(CCC1=CC=C(C=C1)Cl)(CN2C=NC=N2)O | 0 | validation |
| 144209528 | CC1=C(C(CCC1)(C)C)/C=C/C(=C/C=C/C(=C/C(=O)O)/C)/C | 1 | test |
| 144209527 | CC1=CC=C(C=C1)C(C)(C)C | 0 | train |
| 144209526 | C[C@H]1[C@@H]([C@H]([C@H]([C@@H](O1)OC[C@@H]2[C@H]([C@@H]([C@H]([C@@H](O2)OC3=CC(=C4C(=O)C[C@H](OC4=C3)C5=CC(=C(C=C5)OC)OC)O)O)O)O)O)O)O | 0 | train |
| 144209525 | CCC1=CC=CC=C1O | 1 | validation |
| 144209524 | CC(C)(C)C1=CC=C(C=C1)O | 0 | test |
| 144209523 | C(SC#N)SC#N | 1 | train |
| 144209522 | CC(C)(C)CC(C)(C)C1=CC=CC=C1O | 0 | train |
| 144209521 | C1=CC=C(C=C1)NNC2=CC=CC=C2 | 1 | validation |
| 144209520 | C[Sn](Cl)(Cl)Cl | 0 | test |
| 144209519 | CCC(O)OC(CC)O | 0 | train |
| 144209518 | CC(C)(C)C1=CC(=C(C=C1)O)C(C)(C)C | 0 | train |
| 144209517 | C(#N)C(Br)Br | 0 | validation |
| 144209516 | COC(=O)C1=CC=C(C=C1)O | 1 | test |
| 144209515 | C1=CC=C(C(=C1)O)O | 1 | train |
| 144209514 | COP(=S)(OC)SCN1C(=O)C2=CC=CC=C2C1=O | 0 | train |
| 144209513 | COC(=O)[C@H](CC1=CC=CC=C1)NC(=O)[C@H](CC(=O)O)N | 0 | validation |
| 144209512 | CCCCOC(=O)COC(=O)C1=CC=CC=C1C(=O)OCCCC | 1 | test |
| 144209511 | C1=CC(=C(C=C1[N+](=O)[O-])N)N | 1 | train |
| 144209510 | CC1=CC(=O)CC(C1)(C)C | 1 | train |
| 144209509 | CC(C)(Cl)Cl | 0 | validation |
| 144209508 | C1=CC(=CC=C1C(=O)C2=C(C=C(C=C2)O)O)O | 1 | test |
| 144209507 | CC(C)(C)C1=C(C=CC(=C1)O)O | 1 | train |
| 144209506 | C1=CC(=C(C=C1C2=C(C(=O)C3=C(C=C(C=C3O2)O)O)O)O)O | 1 | train |
| 144209505 | [O-][Cr](=O)(=O)O[Cr](=O)(=O)[O-].[K+].[K+] | 0 | validation |
| 144209504 | C1=CC=C2C(=C1)C(=NN2CC3=C(C=C(C=C3)Cl)Cl)C(=O)O | 0 | test |
| 144209503 | CC1=CC=CC=C1O | 1 | train |
| 144209502 | C1=CC=C(C=C1)N.Cl | 0 | train |
| 144209501 | COP(=S)(OC)OC1=CC=C(C=C1)[N+](=O)[O-] | 0 | validation |
| 144209500 | CC(C)CCCCCCCOC(=O)CCCCC(=O)OCCCCCCCC(C)C | 0 | test |
| 144209499 | CCCCCCC(=CC1=CC=CC=C1)C=O | 0 | train |
| 144209498 | C1=CC=C(C=C1)NC2=CC=C(C=C2)[N+](=O)[O-] | 1 | train |
| 144209497 | C1=CC=C(C=C1)C2(C(=O)NC(=O)N2)C3=CC=CC=C3 | 1 | validation |
| 144209496 | COC(=O)C1CCC(CC1)C(=O)OC | 0 | test |
| 144209495 | C(C(=O)O)(Br)Br | 0 | train |
| 144209494 | CC(C)CCCCCCOC(=O)C1=CC=CC=C1C(=O)OCCCCCCC(C)C | 0 | train |
| 144209493 | COC(=O)NC1=NC2=CC=CC=C2N1 | 1 | validation |
| 144209492 | CCOP(=S)(OCC)SCN1C2=C(C=C(C=C2)Cl)OC1=O | 1 | test |
| 144209491 | [NH4+].[O-]Cl(=O)(=O)=O | 0 | train |
| 144209490 | C(Cl)(Cl)(Cl)Br | 0 | train |

| | | | |
|---|---|---|---|
| 144209489 | C1=CC(=CC=C1[N+](=O)[O-])Cl | 0 | validation |
| 144209488 | C1(C(C(C(C(C1Cl)Cl)Cl)Cl)Cl)Cl | 0 | test |
| 144209487 | CCC[N+](=O)[O-] | 1 | train |
| 144209486 | C1=CC(=C(C=C1O)O)C(=O)C2=C(C=C(C=C2)O)O | 1 | train |
| 144209485 | CC(C)(C)OOC(=O)C1=CC=CC=C1 | 0 | validation |
| 144209484 | CCCOC(=O)C1=CC(=C(C(=C1)O)O)O | 1 | test |
| 144209483 | OP(=O)(O)O | 0 | train |
| 144209482 | C1=CC=C2C(=C1)C(=CN2)CC(=O)O | 0 | train |
| 144209481 | C1=CC=NC(=C1)Cl | 0 | validation |
| 144209480 | C/C=C/C1=CC=C(C=C1)OC | 0 | test |
| 144209479 | CC(=C)C(=O)N | 0 | train |
| 144209478 | CC(COCC(C)OC(=O)C1=CC=CC=C1)OC(=O)C2=CC=CC=C2 | 0 | train |
| 144209477 | C1(=C(C(C(=C1Cl)Cl)(Cl)Cl)Cl)Cl | 0 | validation |
| 144209476 | C/C=C(¥C)/C=C/C=C(C)C | 0 | test |
| 144209475 | C1=CC=C(C=C1)OC2=CC=CC=C2 | 0 | train |
| 144209474 | CCCCC(CC)C(=O)OCCOCCOCCOC(=O)C(CC)CCCC | 0 | train |
| 144209473 | C1=CC=C2C(=C1)OC3=CC=CC=C3S2 | 0 | validation |
| 144209472 | CC1=C(C(=C(C=C1)C)O)C | 0 | test |
| 144209471 | C1=CC=C2C(=C1)C=CC3=CC=CC=C3N2C(=O)N | 0 | train |
| 144209470 | CC1=C(C=CC(=C1)Cl)OCC(=O)O | 0 | train |
| 144209469 | [NH4+].[N+](=O)([O-])[O-] | 0 | validation |
| 144209468 | CCCCN(CC)C1=C(C=C(C=C1[N+](=O)[O-])C(F)(F)F)[N+](=O)[O-] | 1 | test |
| 144209467 | C[C@]12CCC(=O)C=C1CC[C@@H]3[C@@H]2CC[C@]4([C@H]3CCC4=O)C | 0 | train |
| 144209466 | COC(=O)C1=CC=C(C=C1)C(=O)OC | 1 | train |
| 144209465 | C1C(O1)COCCCCOCC2CO2 | 0 | validation |
| 144209464 | CCCCCCOC(=O)C1=CC=C(C=C1)O | 1 | test |
| 144209463 | CC1=C(ON=C1C)NS(=O)(=O)C2=CC=C(C=C2)N | 0 | train |
| 144209462 | CC(CN1C2=CC=CC=C2SC3=CC=CC=C31)N(C)C.Cl | 0 | train |
| 144209461 | CCCCCCC1=C(C=C(C=C1)O)O | 0 | validation |
| 144209460 | C(C(CCl)O)O | 0 | test |
| 144209459 | CC1=CC=CC=C1N=NC2=CC(=C(C=C2)N)C | 1 | train |
| 144209458 | CC(CCO)CC(C)(C)C | 0 | train |
| 144209457 | [Cl-].[Cl-].[Hg+2] | 1 | validation |
| 144209456 | CCC1=CC=CC=C1CC | 0 | test |
| 144209455 | C1=CC(=C(C=C1N(CCO)CCO)[N+](=O)[O-])NCCO | 0 | train |
| 144209454 | CC(C)(C)C=C | 0 | train |
| 144209453 | CC(CCC(=O)O)(C1=CC=C(C=C1)O)C2=CC=C(C=C2)O | 0 | validation |
| 144209452 | CC1=CC(=C(C=C1)N)S(=O)(=O)O | 0 | test |
| 144209451 | C1=CC=C(C=C1)NN=NC2=CC=CC=C2 | 1 | train |
| 144209450 | CC1=CC(=C(C(=C1)C)O)C | 0 | train |
| 144209449 | CCCCCCCC(=O)Cl | 0 | validation |
| 144209448 | COP(=O)(N)SC | 0 | test |
| 144209447 | C=CCN=C=S | 0 | train |
| 144209446 | C1=CC(=CC(=C1)Cl)C2=CC(=CC(=C2)Cl)Cl | 1 | train |
| 144209445 | CCOC(=O)C1C(O1)C2=CC=CC=C2 | 0 | validation |
| 144209444 | CNC(=O)CSP(=S)(OC)OC | 0 | test |
| 144209443 | C(CCO)CO | 0 | train |
| 144209442 | C1=CC=C(C(=C1)C2=CC=CC=C2Cl)Cl | 1 | train |
| 144209441 | C1=CC(=CC=C1N)S(=O)(=O)NC2=NC=CS2 | 0 | validation |
| 144209440 | C1CNCCN1 | 1 | test |
| 144209439 | CC(=O)O[Hg]C1=CC=CC=C1 | 1 | train |
| 144209438 | C1=CC(=C(C=C1F)F)C(CN2C=NC=N2)(CN3C=NC=N3)O | 0 | train |
| 144209437 | C1=CC=C(C(=C1)C(C2=CC=C(C=C2)Cl)C(Cl)(Cl)Cl)Cl | 1 | validation |
| 144209436 | CC(=C)C(=O)OCCOCCOCCOC(=O)C(=C)C | 1 | test |
| 144209435 | C1=CC(=CC(=C1)Cl)N | 0 | train |
| 144209434 | C=CC(=O)OCCC(=O)O | 1 | train |
| 144209433 | C[C@@H]1CC(=O)C=C([C@]12C(=O)C3=C(O2)C(=C(C=C3OC)OC)Cl)OC | 0 | validation |
| 144209432 | CC(=CCO)C | 0 | test |
| 144209431 | CCCCCCCCOC(=O)C1=CC=CC=C1C(=O)OCCCCCCCC | 0 | train |
| 144209430 | C1=CC2=C(C=C(C=C2C=C1N)S(=O)(=O)O)O | 0 | train |
| 144209429 | CCCCC(CC)COC(=O)CCCCC(=O)OCC(CC)CCCC | 0 | validation |
| 144209428 | C1CN(CCN1)CCN | 0 | test |

| | | | |
|---|---|---|---|
| 144209427 | [Cl-].[Cl-].[Cd+2] | 1 | train |
| 144209426 | CCCN(CCC)C1=C(C=C(C=C1[N+](=O)[O-])C(C)C)[N+](=O)[O-] | 1 | train |
| 144209425 | CC(C)(C=NOC(=O)NC)SC | 0 | validation |
| 144209424 | C1=CC=C2C=C3C=CC=CC3=CC2=C1 | 1 | test |
| 144209423 | C(CCl)C#N | 0 | train |
| 144209422 | C1=CC(=C(C=C1C2=CC(=C(C=C2)N)Cl)Cl)N | 1 | train |
| 144209421 | CC1=CC(=C(C=C1)[N+](=O)[O-])C | 1 | validation |
| 144209420 | CCC(C)(C1=CC=C(C=C1)O)C2=CC=C(C=C2)O | 1 | test |
| 144209419 | CC1=CC(=NC(=N1)NS(=O)(=O)C2=CC=C(C=C2)N)C | 0 | train |
| 144209418 | C1=CC=C(C=C1)C2=CC=C(C=C2)O | 1 | train |
| 144209417 | C1=CC=C2C(=C1)C(=O)OC2(C3=CC=C(C=C3)O)C4=CC=C(C=C4)O | 1 | validation |
| 144209416 | CCC1=CC=CC2=C1NC3=C2CCOC3(CC)CC(=O)O | 0 | test |
| 144209415 | CC[N+](=O)[O-] | 0 | train |
| 144209414 | CC1COC2=CC=CC=C2N1C(=O)C(Cl)Cl | 1 | train |
| 144209413 | CC1=C(C(=O)C=CO1)O | 0 | validation |
| 144209412 | CC1=CC2=C(C=C1)C(=O)OC2=O | 0 | test |
| 144209411 | C1=COC(=C1)CNC2=CC(=C(C=C2C(=O)O)S(=O)(=O)N)Cl | 0 | train |
| 144209410 | C1=CC=C(C=C1)[O-].[Na+] | 0 | train |
| 144209409 | CCCCCNCCCCC | 0 | validation |
| 144209408 | C1C=CCC2C1C(=O)OC2=O | 0 | test |
| 144209407 | CCCCCCCCCC(=O)O | 0 | train |
| 144209406 | CCCCCCCCCCCCCO | 0 | train |
| 144209405 | C(C#N)Br | 0 | validation |
| 144209404 | C(CCCN=C=O)CCN=C=O | 0 | test |
| 144209403 | CCC1=C(C(=CC=C1)CC)N(COC)C(=O)CCl | 1 | train |
| 144209402 | CC(=O)NP(=O)(OC)SC | 0 | train |
| 144209401 | CC1=CN(C(=O)NC1=O)[C@H]2C[C@@H]([C@H](O2)CO)N=[N+]=[N-] | 0 | validation |
| 144209400 | C1CCC(=O)NCC1 | 1 | test |
| 144209399 | CC(=C)C(=O)O[C@@H]1C[C@H]2CC[C@@]1(C2(C)C)C | 1 | train |
| 144209398 | CC1=CC=C(O1)C(=O)O | 0 | train |
| 144209397 | CN(C)NC(=O)CCC(=O)O | 0 | validation |
| 144209396 | C1=CC=C(C=C1)NN.Cl | 1 | test |
| 144209395 | C1=CC=C(C=C1)CCN=C(N)N=C(N)N.Cl | 1 | train |
| 144209394 | CC1=C(C=CC(=C1)C2=CC(=C(C=C2)N)C)N.Cl.Cl | 1 | train |
| 144209393 | C(C(=O)O)N(CC(=O)O)CC(=O)O | 0 | validation |
| 144209392 | C[C@@H]1CC[C@@H]2[C@@]13C[C@H](C2(C)C)C(=C(C3)C(=O)C)C | 1 | test |
| 144209391 | C(=C¥C(=O)O)¥C(=O)O | 0 | train |
| 144209390 | CCN(CCN)C1=CC=CC(=C1)C | 0 | train |
| 144209389 | C1=COC(=C1)C=O | 0 | validation |
| 144209388 | CC1=C(C=C(C=C1)[N+](=O)[O-])S(=O)(=O)O | 0 | test |
| 144209387 | COC(=O)CCC(=O)OC | 0 | train |
| 144209386 | CC1=CCC(CC1)C(C)(C)OC(=O)C | 0 | train |
| 144209385 | C[C@@]12CC[C@@H](C1(C)C)CC2=O | 0 | validation |
| 144209384 | CCCCCCCCCCCO | 0 | test |
| 144209383 | B(O)(O)O | 0 | train |
| 144209382 | CC1=NC(=NC(=N1)OC)NC(=O)NS(=O)(=O)C2=C(SC=C2)C(=O)OC | 0 | train |
| 144209381 | C(CCC#N)CC#N | 0 | validation |
| 144209380 | CC(=O)CC(C1=CC=CC=C1)C2=C(C3=CC=CC=C3OC2=O)O | 0 | test |
| 144209379 | CC1=CC(=CC(=C1)O)C | 1 | train |
| 144209378 | C1=CC=[N+](C(=C1)[S-])[O-].C1=CC=[N+](C(=C1)[S-])[O-].[Zn+2] | 1 | train |
| 144209377 | C1=CC(=CC(=C1)Cl)Cl | 1 | validation |
| 144209376 | CCCCCC/C=C/CCCCCCCC(=O)O | 1 | test |
| 144209375 | CC1=C(N(C(=C1)CC(=O)[O-])C)C(=O)C2=CC=C(C=C2)Cl.[Na+] | 0 | train |
| 144209374 | CC1([C@@H](N2[C@H](S1)[C@@H](C2=O)NC(=O)COC3=CC=CC=C3)C(=O)[O-])C.[K+] | 0 | train |
| 144209373 | C1=CC=C(C=C1)CCOC(=O)C2=CC=CC=C2N | 1 | validation |
| 144209372 | CC(=O)N(C)C | 0 | test |
| 144209371 | CCCCCCC=O | 1 | train |
| 144209370 | CCCCCC1C(CCC1=O)CC(=O)OC | 0 | train |
| 144209369 | CCCCC/C=C¥C/C=C¥CCCCCCCC(=O)O | 0 | validation |
| 144209368 | CCC(COC(=O)C=C)(COC(=O)C=C)COC(=O)C=C | 1 | test |
| 144209367 | CC(C)C(=O)NC1=CC(=C(C=C1)[N+](=O)[O-])C(F)(F)F | 1 | train |
| 144209366 | CCC(C)(C)C1=CC(=C(C=C1)O)C(C)(C)CC | 0 | train |

| | | | |
|---|---|---|---|
| 144209365 | CC1=C(C2=CC=CC=C2C=C1)C | 0 | validation |
| 144209364 | C(C(CCl)OP(=O)(OC(CCl)CCl)OC(CCl)CCl)Cl | 1 | test |
| 144209363 | C1=C2C(=C(C(=C1Br)O)Br)OC3=C(C(=C(C=C3C24C5=C(C(=C(C(=C5Cl)Cl)Cl)Cl)C(=O)O4)Br)O)Br | 0 | train |
| 144209362 | CCCCCC(=O)C | 0 | train |
| 144209361 | C(=O)(N)NC(=O)N | 0 | validation |
| 144209360 | CN1C=C(C(=O)C(=C1)C2=CC(=CC=C2)C(F)(F)F)C3=CC=CC=C3 | 0 | test |
| 144209359 | C(CCC(=O)O)CC(=O)O | 0 | train |
| 144209358 | CC1(C(=O)N(C(=O)O1)C2=CC(=CC(=C2)Cl)Cl)C=C | 0 | train |
| 144209357 | CC1=CC(=C(C=C1)[N+](=O)[O-])[N+](=O)[O-] | 1 | validation |
| 144209356 | C(=O)(N)N | 1 | test |
| 144209355 | C1=CC=C(C(=C1)Cl)Cl | 0 | train |
| 144209354 | CC(=CCCC(=CCCC(=O)C)C)C | 0 | train |
| 144209353 | N(=O)[O-].[Na+] | 0 | validation |
| 144209352 | C1=CC=C(C=C1)N/N=C/2¥C(=O)C=CC3=CC(=CC(=C32)S(=O)(=O)[O-])S(=O)(=O)[O-].[Na+].[Na+] | 0 | test |
| 144209351 | C1=CC=C(C=C1)N=NC2=C(N=C(C=C2)N)N.Cl | 1 | train |
| 144209350 | COC1=C(C=CC(=C1)C2=CC(=C(C=C2)N)OC)N.Cl.Cl | 1 | train |
| 144209349 | CCN(C(=O)N)N=O | 1 | validation |
| 144209348 | C1C(O1)COC2=CC=C(C=C2)C(C3=CC=C(C=C3)OCC4CO4)C(C5=CC=C(C=C5)OCC6CO6)C7=CC=C(C=C7)OCC8CO8 | 1 | test |
| 144209347 | CCCCCCCCCCCC(=O)O | 0 | train |
| 144209346 | CC(C)(C)C(=O)CCl | 0 | train |
| 144209345 | OS(=O)(=O)F | 0 | validation |
| 144209344 | CCCCCCOCCOCCO | 0 | test |
| 144209343 | COC(=O)C1=CC=CC=C1C(=O)OC | 0 | train |
| 144209342 | C1=CC=C(C=C1)NC2=CC=C(C=C2)N | 1 | train |
| 144209341 | C1CCC(=NO)CC1 | 0 | validation |
| 144209340 | CC1=CC=C(C=C1)N | 1 | test |
| 144209339 | CC(C)(C1=CC=C(C=C1)OCC2CO2)C3=CC=C(C=C3)OCC4CO4 | 0 | train |
| 144209338 | C1C2=CC=CC=C2C3=CC=CC=C31 | 0 | train |
| 144209337 | CC1=CC=C(C=C1)S(=O)(=O)OC2=CC=C(C=C2)N=NC3=C(C=C(C=C3)C4=CC(=C(C=C4)NN=C5C(=O)C=CC6=CC(=CC(=C65)S(=O)(=O)[O-])S(=O)(=O)[O-])C)C.[Na+].[Na+] | 0 | validation |
| 144209336 | CC(=CC1C(C1(C)C)C(=O)OCC2=COC(=C2)CC3=CC=CC=C3)C | 1 | test |
| 144209335 | C1CC(=O)OC2=CC=CC=C21 | 0 | train |
| 144209334 | C(CCl)OP(OCCCl)OCCCl | 0 | train |
| 144209333 | CC(CN)N | 0 | validation |
| 144209332 | C1=CC=C2C(=C1)N=C(S2)Br | 1 | test |
| 144209331 | [O-]Cl(=O)=O.[Na+] | 0 | train |
| 144209330 | C1COCCN1N=O | 0 | train |
| 144209329 | C1(=C(C(=C(C(=C1Cl)Cl)Cl)Cl)Cl)O | 1 | validation |
| 144209328 | C1CO[C@@H]([C@H](O1)Cl)Cl | 0 | test |
| 144209327 | CN(C)C1=CC=C(C=C1)N=O | 1 | train |
| 144209326 | CC(C)CC(C)(C1=CC=C(C=C1)O)C2=CC=C(C=C2)O | 1 | train |
| 144209325 | C([C@@H]([C@@H]1C(=C(C(=O)O1)O)O)O)O | 0 | validation |
| 144209324 | CCCCC(CC)COP(=O)(O)O | 0 | test |
| 144209323 | CCOC1=C(C=CC(=C1)C=O)O | 0 | train |
| 144209322 | CCCCCCCCCCCCO | 0 | train |
| 144209321 | CC1CC(OC(O1)C)OC(=O)C | 0 | validation |
| 144209320 | C(=O)(C(=O)O)O | 0 | test |
| 144209319 | C1CCC(=O)CC1 | 0 | train |
| 144209318 | C1=CC=C2C(=C1)C=CC=C2O | 1 | train |
| 144209317 | CC(CCC=C(C)C)CCO | 0 | validation |
| 144209316 | C(CCl)P(=O)(O)O | 0 | test |
| 144209315 | CC(=O)OC1=CC=CC=C1C(=O)O | 0 | train |
| 144209314 | C1=CC=C2C(=C1)C3=CC=CC=C3O2 | 0 | train |
| 144209313 | C1=CC(=C(C=C1N)Cl)Cl | 0 | validation |
| 144209312 | C1=CC=C(C=C1)P(C2=CC=CC=C2)C3=CC=CC=C3 | 1 | test |
| 144209311 | C1=CC(=C(C(=C1)Cl)Cl)Cl | 0 | train |
| 144209310 | C1C(O1)C2=CC=CC=C2 | 0 | train |
| 144209309 | C1=CC=C(C=C1)OCCO | 0 | validation |
| 144209308 | CCCN(CCC)S(=O)(=O)C1=CC=C(C=C1)C(=O)O | 0 | test |

| | | | |
|---|---|---|---|
| 144209307 | CC1CC(CC(C1)(C)C)OC(=O)C2=CC=CC=C2O | 0 | train |
| 144209306 | C1=CC=C(C=C1)C2=CC=CC=C2 | 0 | train |
| 144209305 | C1=CC=C2C(=C1)NC=N2 | 0 | validation |
| 144209304 | C1=CC(=CC(=C1)N)N | 0 | test |
| 144209303 | CCCCCCOCCO | 0 | train |
| 144209302 | CCC(O)OCC(C)OC(CC)O | 0 | train |
| 144209301 | C1CCC(CC1)OC(=O)CC(C(=O)OC2CCCCC2)S(=O)(=O)[O-].[Na+] | 0 | validation |
| 144209300 | C1=COC(=C1)CO | 0 | test |
| 144209299 | CCCCCCCCCCCC[N+](C)(C)C.[Cl-] | 0 | train |
| 144209298 | CCN(CC)C(=O)C(=C(C)OP(=O)(OC)OC)Cl | 1 | train |
| 144209297 | CC(C)(C1=CC=CC=C1)C2=CC=C(C=C2)NC3=CC=C(C=C3)C(C)(C)C4=CC=CC=C4 | 0 | validation |
| 144209296 | CC(C)NC1=NC(=NC(=N1)OC)NC(C)C | 0 | test |
| 144209295 | COC1=CC=C(C=C1)C2=COC3=CC(=CC(=C3C2=O)O)O | 1 | train |
| 144209294 | CC(=O)C(=O)O | 0 | train |
| 144209293 | CCCCC(CC)C=O | 0 | validation |
| 144209292 | COS(=O)(=O)C | 0 | test |
| 144209291 | CC(=CCCC(=CC=O)C)C | 0 | train |
| 144209290 | C[NH+](C)CCC(C1=CC=C(C=C1)Cl)C2=CC=CC=N2.C(=C¥C(=O)[O-])¥C(=O)O | 0 | train |
| 144209289 | C1=CC=C2C=C(C=CC2=C1)N | 1 | validation |
| 144209288 | CC1(C2CCC(O1)(CC2)C)C | 0 | test |
| 144209287 | CC(=CCC/C(=C/COC(=O)C)/C)C | 0 | train |
| 144209286 | C(CCCN)CCN | 0 | train |
| 144209285 | C1(C(=O)NC(=O)N1)NC(=O)N | 0 | validation |
| 144209284 | CCNCC(=C)C | 0 | test |
| 144209283 | CCCNCCC | 0 | train |
| 144209282 | CC(C)(C#N)N=NC(C)(C)C#N | 0 | train |
| 144209281 | CC(=O)OCCOC(=O)C | 0 | validation |
| 144209280 | CCNC1=NC(=NC(=N1)SC)NC(C)(C)C | 1 | test |
| 144209279 | CC1=C(C(=C2CC[C@@](OC2=C1C)(C)CCC[C@H](C)CCC[C@H](C)CCCC(C)C)C)O | 0 | train |
| 144209278 | CC(=CC(=O)C)C | 0 | train |
| 144209277 | CC(C)(C)C(C(CC1=CC=C(C=C1)Cl)N2C=NC=N2)O | 0 | validation |
| 144209276 | CC(C1=CC=CC=C1)(C2=CC=CC=N2)OCCN(C)C.C(CC(=O)O)C(=O)O | 0 | test |
| 144209275 | C(C(CO)O)O | 0 | train |
| 144209274 | C=CC#N | 0 | train |
| 144209273 | C1=CC=C(C=C1)C(=O)CCl | 1 | validation |
| 144209272 | C(CCl)OCCCl | 0 | test |
| 144209271 | C1=CC=C(C=C1)NC2=CC=CC3=CC=CC=C32 | 1 | train |
| 144209270 | C1[C@@H]2C=CC1[C@H]3[C@@H]2[C@]4(C(=C(C3(C4(Cl)Cl)Cl)Cl)Cl)Cl | 1 | train |
| 144209269 | CCCCC(CC)CO | 0 | validation |
| 144209268 | CC/C(=C(¥C1=CC=CC=C1)/C2=CC=C(C=C2)OCCN(C)C)/C3=CC=CC=C3.C(C(=O)O)C(CC(=O)O)(C(=O)O)O | 1 | test |
| 144209267 | C1(C(C(C(C(C1Cl)Cl)Cl)Cl)Cl)Cl | 1 | train |
| 144209266 | C(NC(=O)N(CO)C1C(=O)N(C(=O)N1CO)CO)O | 0 | train |
| 144209265 | C1CCCCCC(=O)NCCCCC1 | 1 | validation |
| 144209264 | C1=CC2=C(C=C1C(=O)O)C(=O)OC2=O | 0 | test |
| 144209263 | CCCCCCCCCCCCCC(=O)OC(C)C | 0 | train |
| 144209262 | C=CCN(CC=C)C(=O)C(Cl)Cl | 0 | train |
| 144209261 | C1=CC=C2C=C(C=CC2=C1)NC3=CC=C(C=C3)NC4=CC5=CC=CC=C5C=C4 | 1 | validation |
| 144209260 | C(CO)N(CCO)CCO | 0 | test |
| 144209259 | CC1=C(C(=CC=C1)C)N(C(C)C(=O)OC)C(=O)COC | 0 | train |
| 144209258 | C1=CC=C2C(=C1)C=CC3=CC4=CC=CC=C4C=C32 | 1 | train |
| 144209257 | C1=CN=C(C=N1)C(=O)N | 0 | validation |
| 144209256 | CC1=C(C=C(C=C1)N=C=O)N=C=O | 0 | test |
| 144209255 | C[C@]12CC[C@H]3[C@H]([C@@H]1CC[C@@H]2O)CCC4=C3C=CC(=C4)O | 1 | train |
| 144209254 | CC1=CC(=CC=C1)C | 1 | train |
| 144209253 | CC1=CC(=C(C=C1)N)[N+](=O)[O-] | 1 | validation |
| 144209252 | CC(=O)C1=NC=CS1 | 0 | test |
| 144209251 | CCCCC(CC)COC(=O)C=C | 1 | train |
| 144209250 | C[C@@]12[C@H](C=C[C@@]3([C@@H]1[C@@H]([C@]45[C@H]3CC[C@](C4)(C(=C)C5)O)C(=O)O)OC2=O)O | 0 | train |
| 144209249 | CCCCCC(=CC1=CC=CC=C1)C=O | 1 | validation |
| 144209248 | CC(COC)N | 1 | test |

| | | | |
|---|---|---|---|
| 144209247 | CC1(C(=O)NC(=O)N1)C | 0 | train |
| 144209246 | CN(C)CCCN | 0 | train |
| 144209245 | CC1COC(=O)O1 | 0 | validation |
| 144209244 | CC(C)(C)C1=CC=C(C=C1)OC2CCCCC2OS(=O)OCC#C | 0 | test |
| 144209243 | C1=CC=C2C(=C1)OC3=CC=CC=C3O2 | 1 | train |
| 144209242 | CC(CCC=C(C)C)C=C | 0 | train |
| 144209241 | CNC1=C(C(=O)N(N=C1)C2=CC=CC(=C2)C(F)(F)F)Cl | 1 | validation |
| 144209240 | CCCCOCCOCCOC(=O)CCCCC(=O)OCCOCCOCCCC | 0 | test |
| 144209239 | CC(C)(C)C1=CC=CC=C1O | 0 | train |
| 144209238 | COC1=C(C=CC(=C1)CC=C)O | 1 | train |
| 144209237 | CC1CCC2CC1C2(C)C | 1 | validation |
| 144209236 | C(CO)O | 1 | test |
| 144209235 | C1=C(C=C(C(=C1Cl)O)Cl)Cl | 0 | train |
| 144209234 | C1=CC(=NC(=C1)Cl)C(Cl)(Cl)Cl | 0 | train |
| 144209233 | C1=CC(=CC=C1N)OC2=CC=C(C=C2)N | 1 | validation |
| 144209232 | C1=CC=C(C=C1)OP(OC2=CC=CC=C2)OC3=CC=CC=C3 | 0 | test |
| 144209231 | C1=CC(=CC=C1OC2=CC=C(C=C2)Br)Br | 0 | train |
| 144209230 | COS(=O)(=O)OC | 0 | train |
| 144209229 | C[C@](CC1=CC(=C(C=C1)O)O)(C(=O)O)N.C[C@](CC1=CC(=C(C=C1)O)O)(C(=O)O)N.O.O.O | 0 | validation |
| 144209228 | C1=CC(=CC=C1NC(=O)NC2=CC(=C(C=C2)Cl)Cl)Cl | 1 | test |
| 144209227 | CC(=CCC/C(=C¥CO)/C)C | 0 | train |
| 144209226 | CCOP(=S)(OCC)OC1=NC2=CC=CC=C2N=C1 | 0 | train |
| 144209225 | C1=C(C(=CC(=C1Cl)N)Cl)C2=CC(=C(C=C2Cl)N)Cl | 1 | validation |
| 144209224 | CCC(=O)C(CC(C)N(C)C)(C1=CC=CC=C1)C2=CC=CC=C2.Cl | 0 | test |
| 144209223 | CC(C)C1=CC=C(C=C1)CO | 0 | train |
| 144209222 | C=CC(=O)OCC(CO)(COC(=O)C=C)COC(=O)C=C | 1 | train |
| 144209221 | C(C(CCl)Cl)Cl | 0 | validation |
| 144209220 | CC(=O)C1=CC=C(C=C1)S(=O)(=O)NC(=O)NC2CCCCC2 | 0 | test |
| 144209219 | C1=CC=C(C=C1)C2=NC3=C(N=C2N)N=C(N=C3N)N | 1 | train |
| 144209218 | C1=CC(=O)C=CC1=O | 1 | train |
| 144209217 | CCOC(=O)C1=CC=CC=C1N | 0 | validation |
| 144209216 | CC1=C(C=CC(=C1)Cl)O | 1 | test |
| 144209215 | CCOC(=O)CC(C(=O)OCC)SP(=O)(OC)OC | 0 | train |
| 144209214 | CC1=C(C=C(C=C1)S(=O)(=O)[O-])C.[Na+] | 0 | train |
| 144209213 | CCCCNC(=O)N | 0 | validation |
| 144209212 | CC1(C2CCC(C2)C1=C)C | 0 | test |
| 144209211 | CN(C)CCOCCN(C)C | 1 | train |
| 144209210 | CCCOC(=O)C | 0 | train |
| 144209209 | C(C(=O)[O-])Cl.[Na+] | 0 | validation |
| 144209208 | CCC1=C(C(=CC=C1)CC)N | 0 | test |
| 144209207 | CCCCCCCCCCCCCC[P+](CCCC)(CCCC)CCCC.[Cl-] | 1 | train |
| 144209206 | CC(C)C1(C(=O)NC(=N1)C2=NC3=CC=CC=C3C=C2C(=O)O)C | 0 | train |
| 144209205 | CCOC(=O)C(C)OC1=CC=C(C=C1)OC2=CN=C3C=C(C=CC3=N2)Cl | 1 | validation |
| 144209204 | CC(C)(C)C1=NN=C(N(C1=O)N)SC | 0 | test |
| 144209203 | CCOP(=S)(OCC)OC1=CC=C(C=C1)[N+](=O)[O-] | 1 | train |
| 144209202 | C1=CC=C2C=CC=CC2=C1 | 0 | train |
| 144209201 | CCCC(C(CC)CO)O | 0 | validation |
| 144209200 | CCCCC(CC)COC(=O)C1=CC(=C(C=C1)C(=O)OCC(CC)CCCC)C(=O)OCC(CC)CCCC | 0 | test |
| 144209199 | CC=CC1=CC=C(C=C1)OC | 0 | train |
| 144209198 | CC1=C(C(=CC=C1)[N+](=O)[O-])C | 0 | train |
| 144209197 | CC(=O)NS(=O)(=O)C1=CC=C(C=C1)N | 0 | validation |
| 144209196 | C1=CC2=C(C3=C(C=CC=N3)C=C2)N=C1 | 1 | test |
| 144209195 | CC1=CC(=C(C=C1)C)OCCCC(C)(C)C(=O)O | 0 | train |
| 144209194 | C[C@]12CC[C@H]3[C@H]([C@@H]1CCC2=O)CC=C4[C@@]3(CC[C@@H](C4)O)C | 0 | train |
| 144209193 | CC1=CC(=O)N=C(N1)C(C)C | 0 | validation |
| 144209192 | CCCOCCO | 0 | test |
| 144209191 | C1=CC(=CC=C1N)N | 1 | train |
| 144209190 | CCCCC(CC)COC(=O)C | 1 | train |
| 144209189 | CC(C)CC1=CC=CC=C1 | 1 | validation |
| 144209188 | C1CCN(CC1)C(=O)/C=C/C=C/C2=CC3=C(C=C2)OCO3 | 1 | test |
| 144209187 | C(CCCC(=O)O)CCCC(=O)O | 0 | train |

| | | | |
|---|---|---|---|
| 144209186 | CC1=C(SCCO1)C(=O)NC2=CC=CC=C2 | 1 | train |
| 144209185 | C1=CC=C2C(=C1)C(=O)N(C2=O)SC(Cl)(Cl)Cl | 1 | validation |
| 144209184 | CCNC1=NC(=NC(=N1)Cl)NC(C)C | 0 | test |
| 144209183 | COC1=CC(=C(C=C1)C(=O)C2=CC=CC=C2)O | 1 | train |
| 144209182 | COC1=CC=CC=C1[N+](=O)[O-] | 0 | train |
| 144209181 | CCCCC(CC)COCC1CO1 | 0 | validation |
| 144209180 | C1=CC=C(C=C1)C#N | 0 | test |
| 144209179 | CC(C)(C1=CC(=C(C(=C1)Br)O)Br)C2=CC(=C(C(=C2)Br)O)Br | 0 | train |
| 144209178 | CCN(CC)C(=O)C1=CC(=CC=C1)C | 0 | train |
| 144209177 | CC1=C2C(=CC(=C1C(=O)O)O)C(=O)C3=C(C2=O)C(=C(C(=C3O)O)[C@H]4[C@@H]([C@H]([C@@H]([C@H](O4)CO)O)O)O)O | 0 | validation |
| 144209176 | CSCCC=O | 0 | test |
| 144209175 | C1=CC=C(C=C1)NN | 1 | train |
| 144209174 | CCCCO[P+](=O)OCCCC | 0 | train |
| 144209173 | CC(CC(C)(C)O)O | 0 | validation |
| 144209172 | C1CN(CCN1)CCO | 0 | test |
| 144209171 | CC(CN(CCN(CC(C)O)CC(C)O)CC(C)O)O | 0 | train |
| 144209170 | C1=CC(=C(C=C1C(F)(F)F)Cl)Cl | 0 | train |
| 144209169 | CN(C)C(=S)[S-].[Na+] | 1 | validation |
| 144209168 | CCC(C)OC(=O)C1=CC=C(C=C1)O | 1 | test |
| 144209167 | C1=CC(=C(C(=C1)F)C(=O)NC(=O)NC2=CC=C(C=C2)Cl)F | 1 | train |
| 144209166 | CC(C)OC1=CC=CC=C1OC(=O)NC | 1 | train |
| 144209165 | CCCCCCCC/C=C¥CCCCCCCC(=O)O | 0 | validation |
| 144209164 | CC1=CN=CC=C1 | 0 | test |
| 144209163 | COP(=S)(OC)SCN1C(=O)C2=CC=CC=C2N=N1 | 1 | train |
| 144209162 | CC1=CC=C(C=C1)C2=CC(=NN2C3=CC=C(C=C3)S(=O)(=O)N)C(F)(F)F | 0 | train |
| 144209161 | CCCC(CCC)C(=O)O | 0 | validation |
| 144209160 | CCCN(CCC)C(=O)SCC | 0 | test |
| 144209159 | C=CCOC(=O)C1=CC=CC=C1C(=O)OCC=C | 0 | train |
| 144209158 | C1=CC=C(C=C1)S(=O)(=O)O | 1 | train |
| 144209157 | CC1C=C[C-]=C1.[C-]#[O+].[C-]#[O+].[C-]#[O+].[Mn] | 1 | validation |
| 144209156 | CC1(CC2=C(O1)C(=CC=C2)O)C | 0 | test |
| 144209155 | CCCCOCCOCCOC(=O)C | 0 | train |
| 144209154 | C1=CC(=C(C=C1[N+](=O)[O-])[N+](=O)[O-])Cl | 1 | train |
| 144209153 | CC1(CC2=C(O1)C(=CC=C2)OC(=O)NC)C | 1 | validation |
| 144209152 | C(CCCCCC(=O)O)CCCCC(=O)O | 0 | test |
| 144209151 | C1=CC=C(C(=C1)CC2=CC=C(C=C2)O)O | 1 | train |
| 144209150 | CC(CN(CC(C)O)CC(C)O)O | 0 | train |
| 144209149 | CC(C)N1C(=O)C2=CC=CC=C2NS1(=O)=O | 1 | validation |
| 144209148 | C1CCC(CC1)C(=O)N2CC3C4=CC=CC=C4CCN3C(=O)C2 | 0 | test |
| 144209147 | C1=CC=C2C(=C1)NC(=N2)N | 0 | train |
| 144209146 | CCC1(C(=O)NCNC1=O)C2=CC=CC=C2 | 0 | train |
| 144209145 | C1(=C(C(=C(C(=C1Cl)Cl)Cl)Cl)Cl)[N+](=O)[O-] | 1 | validation |
| 144209144 | CCOC1=CC=C(C=C1)N | 1 | test |
| 144209143 | CC1=C(C=C(C=C1)[N+](=O)[O-])N | 1 | train |
| 144209142 | C1=CC=C(C(=C1)C(=O)N)O | 0 | train |
| 144209141 | COC1=CC=C(C=C1)C=O | 0 | validation |
| 144209140 | C(=O)N | 0 | test |
| 144209139 | CC1=CC(=CC=C1)O | 1 | train |
| 144209138 | COC(=O)OC | 0 | train |
| 144209137 | C1=CC(=C(C=C1C(=O)O)C(=O)O)C(=O)O | 0 | validation |
| 144209136 | CC1=CC(=C(C=C1)O)C(C)(C)C | 0 | test |
| 144209135 | C([C@H]([C@H]([C@@H]([C@H](CO)O)O)O)O)O | 0 | train |
| 144209134 | C1=CC=C(C=C1)C(=O)C2=C(C(=C(C=C2)O)O)O | 0 | train |
| 144209133 | CCN(CC)C1=CC(=C(C=C1)C(=O)C2=CC=CC=C2C(=O)O)O | 1 | validation |
| 144209132 | CCCCSP(=O)(SCCCC)SCCCC | 1 | test |
| 144209131 | CCCCCC=O | 0 | train |
| 144209130 | CC1=CC=C(C=C1)C(C)C | 0 | train |
| 144209129 | C1=C(C(=C(C(=C1Cl)Cl)Cl)O)Cl | 0 | validation |
| 144209128 | CC(=O)NC1=CC=C(C=C1)O | 1 | test |
| 144209127 | CC(C)C#N | 0 | train |
| 144209126 | C1=CC=C2C(=C1)C=CC=C2NCCN.Cl.Cl | 1 | train |

| | | | |
|---|---|---|---|
| 144209125 | CN(C)C1=CC(=CC=C1)O | 1 | validation |
| 144209124 | CCOC1=CC=C(C=C1)NC(=O)C | 1 | test |
| 144209123 | COC1=C(C=CC(=C1)C=O)O | 0 | train |
| 144209122 | C1C(=O)NC(=O)N1N=CC2=CC=C(O2)[N+](=O)[O-] | 1 | train |
| 144209121 | C(CN(CC(=O)[O-])CC(=O)[O-])N(CC(=O)[O-])CC(=O)[O-].[Na+].[Fe+3] | 0 | validation |
| 144209120 | C1(=NC(=O)N(C(=O)N1Cl)Cl)[O-].O.O.[Na+] | 0 | test |
| 144209119 | C(C(CO)(CO)CO)O | 0 | train |
| 144209118 | CC1=CC(=C(C=C1C)C)C | 0 | train |
| 144209117 | C[C@@H]1CC[C@H]([C@@H](C1)O)C(C)C | 0 | validation |
| 144209116 | C(CC(=O)O)C(=O)O | 0 | test |
| 144209115 | CCCCCCCCCCC=CC1CC(=O)OC1=O | 0 | train |
| 144209114 | CC1=C(C(=CC=C1)C)NC(=O)CN2CCCC2=O | 0 | train |
| 144209113 | CCCCC(CN1C=NC=N1)(C#N)C2=CC=C(C=C2)Cl | 1 | validation |
| 144209112 | CC(=C)C(=O)OCCOC(=O)C(=C)C | 0 | test |
| 144209111 | CC12CC1(C(=O)N(C2=O)C3=CC(=CC(=C3)Cl)Cl)C | 0 | train |
| 144209110 | COC(=O)CCCCC(=O)OC | 0 | train |
| 144209109 | C1=CC(C2C1C3(C(=C(C2(C3(Cl)Cl)Cl)Cl)Cl)Cl)Cl | 1 | validation |
| 144209108 | CN(C)C(=S)[S-].CN(C)C(=S)[S-].[Zn+2] | 1 | test |
| 144209107 | C1=CC(=CC(=C1)N)C(F)(F)F | 0 | train |
| 144209106 | C1=C(C=C(C(=C1Cl)N)Cl)N | 1 | train |
| 144209105 | CC1=CC(=C(C=C1)OC)N | 1 | validation |
| 144209104 | C1=CC(=C(C=C1C2=CC(=C(C=C2)N)Cl)Cl)N.Cl.Cl | 1 | test |
| 144209103 | NS(=O)(=O)O | 1 | train |
| 144209102 | CC(=C)[C@H]1CC2=C(O1)C=CC3=C2O[C@@H]4COC5=CC(=C(C=C5[C@@H]4C3=O)OC)OC | 0 | train |
| 144209101 | CCCCCCCCCCCCOC(=O)C(=C)C | 0 | validation |
| 144209100 | CCO[Si](CCC#N)(OCC)OCC | 0 | test |
| 144209099 | CO[P+](=O)OC | 0 | train |
| 144209098 | CCCCCCCCCCCCN | 0 | train |
| 144209097 | C1=CC(=CC=C1C(=O)Cl)C(=O)Cl | 0 | validation |
| 144209096 | CC(=O)CC(=O)N(C)C | 0 | test |
| 144209095 | C1=CC(=CC=C1N/N=C¥2/C3=C(C=CC2=O)C=C(C=C3)S(=O)(=O)[O-])S(=O)(=O)[O-].[Na+].[Na+] | 0 | train |
| 144209094 | CCC(CC)NC1=C(C=C(C(=C1[N+](=O)[O-])C)C)[N+](=O)[O-] | 1 | train |
| 144209093 | CN(C)CC1=CC(=C(C(=C1)CN(C)C)O)CN(C)C | 0 | validation |
| 144209092 | CCCCC/C=C/C=C/C=O | 0 | test |
| 144209091 | C1=NN=CN1N | 0 | train |
| 144209090 | C1=C(OC(=C1)C=O)CO | 0 | train |
| 144209089 | C1=CC=C(C=C1)NC2=CC3=CC=CC=C3C=C2 | 1 | validation |
| 144209088 | C(C(CCl)O)Cl | 0 | test |
| 144209087 | C1=CC(=C(C=C1Cl)O)Cl | 0 | train |
| 144209086 | C1=CC=C(C=C1)C(C(=O)C2=CC=CC=C2)O | 1 | train |
| 144209085 | CCCCCCCCCCCCCCCCCC(=O)O | 0 | validation |
| 144209084 | CC(C)NCC(COC1=CC=CC2=CC=CC=C21)O.Cl | 0 | test |
| 144209083 | CCCCOCCOCCOCCO | 0 | train |
| 144209082 | C1=CC=C2C=C(C=CC2=C1)O | 1 | train |
| 144209081 | C1CCC(C(C1)N)N | 1 | validation |
| 144209080 | [Cl-].[Cl-].[Cl-].[Fe+3] | 0 | test |
| 144209079 | CCCCCCCCCC | 0 | train |
| 144209078 | C1=CC=C2C(=C1)C(=O)NS2(=O)=O | 0 | train |
| 144209077 | C1C(O1)CN(CC2CO2)C3=CC=C(C=C3)OCC4CO4 | 0 | validation |
| 144209076 | CCC(C)C1=CC(=CC(=C1O)[N+](=O)[O-])[N+](=O)[O-] | 0 | test |
| 144209075 | CC(C)OC1=C(C=C(C(=C1)N2C(=O)OC(=N2)C(C)(C)C)Cl)Cl | 1 | train |
| 144209074 | C1=CC=C2C(=C1)C(=O)N=NN2 | 0 | train |
| 144209073 | CN(C)C(=O)NC1=CC=CC(=C1)C(F)(F)F | 0 | validation |
| 144209072 | CCOC(=O)C1=CC=CC=C1C(=O)OCC | 1 | test |
| 144209071 | C(=S)(N)N | 0 | train |
| 144209070 | CCCCOP(=O)(OCCCC)OCCCC | 1 | train |
| 144209069 | CC1=C(C=C(C=C1)N)Cl | 1 | validation |
| 144209068 | CC1=CC(=O)NC(=S)N1 | 0 | test |
| 144209067 | C1=CC=C(C=C1)COC(=O)C2=CC=CC=C2O | 1 | train |

| | | | |
|---|---|---|---|
| 144209066 | C[C@H](CCC(=O)O)[C@H]1CC[C@@H]2[C@@]1(CC[C@H]3[C@H]2CC[C@H]4[C@@]3(CC[C@H](C4)O)C)C | 0 | train |
| 144209065 | CS(=O)(=O)C1=CC=C(C=C1)Cl | 0 | validation |
| 144209064 | CC(C)OC(=O)NC1=CC(=CC=C1)Cl | 1 | test |
| 144209063 | CC(=O)[O-].CC(=O)[O-].[Mn+2] | 0 | train |
| 144209062 | CCCCNC(=O)OCC#CI | 1 | train |
| 144209061 | CC(C)(C)C1=CC(=CC(=C1O)C(C)(C)C)CC2=CC(=C(C(=C2)C(C)(C)C)O)C(C)(C)C | 0 | validation |
| 144209060 | C1=CC(=C(C=C1Br)Br)Br | 0 | test |
| 144209059 | CC(C)CCCCCCCOC(=O)C=C | 0 | train |
| 144209058 | CCCN(CCC)N=O | 0 | train |
| 144209057 | CN(C(=O)N[C@@H]1[C@H]([C@@H]([C@H](O[C@@H]1O)CO)O)O)N=O | 0 | validation |
| 144209056 | CC1=CC2C3CC(C2C1)C=C3C | 0 | test |
| 144209055 | COCCOC(=O)C1=CC=CC=C1C(=O)OCCOC | 0 | train |
| 144209054 | C(C(CO)(CBr)CBr)O | 0 | train |
| 144209053 | CC1=C(C=CC=C1[N+](=O)[O-])[N+](=O)[O-] | 0 | validation |
| 144209052 | C1=CC=C(C=C1)OCC(=O)O | 0 | test |
| 144209051 | C1=CC=C2C(=C1)N=CS2 | 0 | train |
| 144209050 | CCCCCCCCCCCCCC(=O)O | 0 | train |
| 144209049 | C1C2=C(C(=CC=C2)O)C(=O)C3=C1C=CC=C3O | 0 | validation |
| 144209048 | C[C@@]1([C@H]2[C@@H]([C@H]3[C@@H](C(=O)C(=C([C@]3(C(=O)C2=C(C4=C1C=CC=C4O)O)O)O)O)C(=O)N)N(C)C)O)O.Cl | 0 | test |
| 144209047 | C(=O)(N)N=NC(=O)N | 0 | train |
| 144209046 | CS(=O)(=O)OCCCCOS(=O)(=O)C | 0 | train |
| 144209045 | CC(C(=O)O)OC1=C(C=C(C=C1)Cl)Cl | 0 | validation |
| 144209044 | C1CC1NC2=NC(=NC(=N2)N)N | 0 | test |
| 144209043 | C1=CC(=CC=C1C(Cl)(Cl)Cl)Cl | 0 | train |
| 144209042 | CC(C)OC=O | 0 | train |
| 144209041 | CCCCOCCOC(=O)C1=CC=CC=C1C(=O)OCCOCCCC | 1 | validation |
| 144209040 | CCCCCCCCCCCCCCO | 0 | test |
| 144209039 | CCOP(=S)(OCC)OC1=CC2=C(C=C1)C(=C(C(=O)O2)Cl)C | 1 | train |
| 144209038 | CC1=C(C=CC(=C1)OC)NC2=CC=CC=C2 | 0 | train |
| 144209037 | C1=CC(=C(C=C1C(F)(F)F)Cl)OC2=CC(=C(C=C2)[N+](=O)[O-])C(=O)O | 0 | validation |
| 144209036 | CC(C)C(C1=CC=C(C=C1)Cl)C(=O)OC(C#N)C2=CC(=CC=C2)OC3=CC=CC=C3 | 1 | test |
| 144209035 | CN(C)P(=O)(N(C)C)N(C)C | 0 | train |
| 144209034 | CNC(=O)OC1=CC=CC2=CC=CC=C21 | 1 | train |
| 144209033 | C1=CC=C2C(=C1)C3=CC=CC4=C3C2=CC=C4 | 1 | validation |
| 144209032 | CCC1=C(C(=NC(=N1)N)N)C2=CC=C(C=C2)Cl | 0 | test |
| 144209031 | C1=CC(=C(C=C1C(F)(F)F)[N+](=O)[O-])Cl | 0 | train |
| 144209030 | COC(=O)C1=CC=CC=C1 | 0 | train |
| 144209029 | CC1=C(C(=CC=C1)O)C | 0 | validation |
| 144209028 | CCCCCCC(=O)O | 0 | test |
| 144209027 | C1=CC(=O)NNC1=O | 0 | train |
| 144209026 | C1=CC=C2C(=C1)NC(=S)N2 | 0 | train |
| 144209025 | CCC[C@@H]1O[C@@H]2C[C@H]3[C@@H]4CCC5=CC(=O)C=C[C@@]5([C@H]4[C@H](C[C@@]3([C@@]2(O1)C(=O)CO)C)O)C | 0 | validation |
| 144209024 | CC(=O)OCC1=CC=CC=C1 | 0 | test |
| 144209023 | C1=CC2=C3C(=C1)C=CC3=CC=C2 | 1 | train |
| 144209022 | CCN(CC)C(=O)C(C)OC1=CC=CC2=CC=CC=C21 | 1 | train |
| 144209021 | C[C@@H]1CC[C@H]([C@@H](C1)O)C(C)C | 0 | validation |
| 144209020 | CC(C)(C)C1=C(C=CC(=C1)OC)O | 0 | test |
| 144209019 | CN(C)CCO | 0 | train |
| 144209018 | COCCOCCOCCO | 0 | train |
| 144209017 | CCCC[N+]1(CCCC1)C.[Cl-] | 0 | validation |
| 144209016 | CC1=CCC(CC1)C(=C)C | 0 | test |
| 144209015 | CC(C)N(C(C)C)C(=O)SCC(=C(Cl)Cl)Cl | 0 | train |
| 144209014 | CCOC1=CC=C(C=C1)NC(=O)CC(C)O | 0 | train |
| 144209013 | C1CN(P(=O)(OC1)NCCCl)CCCl | 0 | validation |
| 144209012 | CN1CCC[C@H]1C2=CN=CC=C2 | 0 | test |
| 144209011 | CN(C)C1=CC=CC=C1 | 0 | train |
| 144209010 | CC(CO)O | 0 | train |
| 144209009 | C1=CC=C(C=C1)OC(=O)C2=CC=CC=C2O | 0 | validation |
| 144209008 | CC1=CC2=C(C=C1)OC(=O)C=C2 | 1 | test |

| | | | |
|---|---|---|---|
| 144209007 | CCCCC1C(=O)N(N(C1=O)C2=CC=CC=C2)C3=CC=CC=C3 | 1 | train |
| 144209006 | C1CSSC1CCCCC(=O)O | 0 | train |
| 144209005 | CCC(C)C(=O)O | 0 | validation |
| 144209004 | C1CCC(CC1)NC(=S)NC2CCCCC2 | 1 | test |
| 144209003 | CC1=C2C=CC3=CC=CC=C3C2=C(C4=CC=CC=C14)C | 1 | train |
| 144209002 | C1=CC=C(C=C1)/C=C/C=O | 0 | train |
| 144209001 | CC(COC)O | 0 | validation |
| 144209000 | CCC1=CC=CC(=C1N)C | 0 | test |
| 144208999 | C1CCCCCC(CCCCC1)O | 0 | train |
| 144208998 | C1=CC=C2C(=C1)C(=O)NC2=O | 0 | train |
| 144208997 | CCCCCCCCS | 0 | validation |
| 144208996 | C(C(CBr)Br)OP(=O)(OCC(CBr)Br)OCC(CBr)Br | 1 | test |
| 144208995 | CC(C(=O)OC)OC1=CC=C(C=C1)OC2=C(C=C(C=N2)C(F)(F)F)Cl | 0 | train |
| 144208994 | CCCCN(CC)C(=O)SCCC | 0 | train |
| 144208993 | C[C@]12C[C@@H]([C@]3([C@H]([C@@H]1C[C@@H]4[C@]2(OC(O4)(C)C)C(=O)CO)CCC5=CC(=O)C=C[C@@]53C)F)O | 0 | validation |
| 144208992 | CCC(COC(=O)C(=C)C)(COC(=O)C(=C)C)COC(=O)C(=C)C | 0 | test |
| 144208991 | C1CCC2=CC=CC=C2C1 | 0 | train |
| 144208990 | CC(=O)[C@H]1CC[C@@H]2[C@@]1(CC[C@H]3[C@H]2CCC4=CC(=O)CC[C@]34C)C | 1 | train |
| 144208989 | CC1=C(C2=C(N1C(=O)C3=CC=C(C=C3)Cl)C=CC(=C2)OC)CC(=O)O | 0 | validation |
| 144208988 | CCCCCCCCCCCCCl | 0 | test |
| 144208987 | CCN(CC)C1=CC2=C(C=C1)C(=CC(=O)O2)C | 1 | train |
| 144208986 | CC(CCC#N)C#N | 0 | train |
| 144208985 | CC1=CC(=C(C=C1)O)C | 0 | validation |
| 144208984 | CCCC=NO | 0 | test |
| 144208983 | CCCCOC(=O)C1=CC=C(C=C1)O | 1 | train |
| 144208982 | CC1=C(C=C(C=C1)[N+](=O)[O-])[N+](=O)[O-] | 0 | train |
| 144208981 | CC=CC1=CC2=C(C=C1)OCO2 | 0 | validation |
| 144208980 | C1=NNC(=N1)N | 0 | test |
| 144208979 | CC(C)(CS(=O)(=O)O)NC(=O)C=C | 0 | train |
| 144208978 | CC(C)(CO)N | 0 | train |
| 144208977 | CCCCOC(=O)CC(CC(=O)OCCCC)(C(=O)OCCCC)OC(=O)C | 1 | validation |
| 144208976 | C(CCCCC(=O)O)CCCC(=O)O | 1 | test |
| 144208975 | C1(C(C(C(C(C1Cl)Cl)Cl)Cl)Cl)Cl | 0 | train |
| 144208974 | C1=CC(=C(C=C1[N+](=O)[O-])S(=O)(=O)[O-])C=CC2=C(C=C(C=C2)[N+](=O)[O-])S(=O)(=O)[O-].[Na+].[Na+] | 0 | train |
| 144208973 | C1COCCN1SSN2CCOCC2 | 0 | validation |
| 144208972 | CCC(=O)NC1=CC(=C(C=C1)Cl)Cl | 0 | test |
| 144208971 | C(=O)(C(Cl)(Cl)Cl)C(Cl)(Cl)Cl | 0 | train |
| 144208970 | C1=CC=C2C(=C1)C=CC3=C2C=CC=N3 | 1 | train |
| 144208969 | C1CCC(CC1)NC2CCCCC2 | 0 | validation |
| 144208968 | CC(=CCl)C | 0 | test |
| 144208967 | C(=C/C(=O)O)¥C(=O)O | 0 | train |
| 144208966 | CC1=CC=CC2=CC=CC=C12 | 0 | train |
| 144208965 | COC1=C2C(=CC3=C1OC=C3)C=CC(=O)O2 | 1 | validation |
| 144208964 | CCNC1=CC=CC=C1 | 0 | test |
| 144208963 | C1C2=CC=CC=C2C3=C1C=C(C=C3)[N+](=O)[O-] | 1 | train |
| 144208962 | CC1=CC=CC=C1C=O | 0 | train |
| 144208961 | C1=CC(=C(C(=C1Cl)O)Cl)Cl | 0 | validation |
| 144208960 | CN(C)C1=CC=C(C=C1)C(=C2C=CC(=[N+](C)C)C=C2)C3=CC=C(C=C3)N(C)C.[Cl-] | 1 | test |
| 144208959 | CCOC(=O)N | 0 | train |
| 144208958 | CC1=CC[C@H]2C[C@@H]1C2(C)C | 0 | train |
| 144208957 | CC(=C)C(=O)OCC=C | 0 | validation |
| 144208956 | CCCCCCCCCCCCS | 1 | test |
| 144208955 | CCCCCCCCCC(=O)OC | 0 | train |
| 144208954 | CCOP(=S)(OCC)OP(=S)(OCC)OCC | 0 | train |
| 144208953 | CC1=CC(=CC=C1)N=NC2=CC=C(C=C2)N(C)C | 1 | validation |
| 144208952 | C1=CC(=CC=C1N=O)O | 0 | test |
| 144208951 | CCOC(=O)C(C1=CC=C(C=C1)Cl)(C2=CC=C(C=C2)Cl)O | 1 | train |
| 144208950 | C([C@@H]1[C@@H]([C@@H]([C@H]([C@H](O1)O[C@]2([C@H]([C@@H]([C@H](O2)CCl)O)O)CCl)O)O)Cl)O | 0 | train |
| 144208949 | C1=CC(=C(C=C1Cl)Cl)OCC(=O)O | 0 | validation |

| | | | |
|---|---|---|---|
| 144208948 | C1=CC=C(C=C1)[N+](=O)[O-] | 0 | test |
| 144208947 | CC1=CC(=C(C=C1)N)N | 1 | train |
| 144208946 | CC(=C)OC(=O)C | 0 | train |
| 144208945 | CC1=NN(C(=O)C1)C2=CC=CC=C2 | 1 | validation |
| 144208944 | CCCCOCC1CO1 | 0 | test |
| 144208943 | CC(=O)OCC(COC(=O)C)OC(=O)C | 1 | train |
| 144208942 | CC(C)(/C=N/OC(=O)NC)S(=O)C | 0 | train |
| 144208941 | C1=CC(=CC=C1O)S(=O)(=O)C2=CC=C(C=C2)O | 0 | validation |
| 144208940 | C(CC=O)CC=O | 0 | test |
| 144208939 | CCCC1=CC(=O)NC(=S)N1 | 0 | train |
| 144208938 | C12=C(C(=C(C(=C1Cl)Cl)Cl)Cl)C(=O)OC2=O | 0 | train |
| 144208937 | CC(=C)C(=O)OCCC[Si](OC)(OC)OC | 0 | validation |
| 144208936 | CCCCCC1=CCCC1=O | 0 | test |
| 144208935 | C1=CC=C(C(=C1)C=O)O | 0 | train |
| 144208934 | C1=CC(=CC=C1C(C2=CC=C(C=C2)O)C(Cl)(Cl)Cl)O | 1 | train |
| 144208933 | CC(C)COC(=O)C | 0 | validation |
| 144208932 | CC(C)N(C1=CC=CC=C1)C(=O)CCl | 1 | test |
| 144208931 | CNC(=S)N(C)C | 0 | train |
| 144208930 | C1=CC=C(C=C1)C(=O)OCCOCCOC(=O)C2=CC=CC=C2 | 1 | train |
| 144208929 | CNC(=O)ON=C(C(=O)N(C)C)SC | 0 | validation |
| 144208928 | CC=CC(=O)O | 0 | test |
| 144208927 | CCCCOC(=O)C1=CC=CC=C1C(=O)OCCCC | 0 | train |
| 144208926 | CC1=CCC(CC1OC(=O)C)C(=C)C | 0 | train |
| 144208925 | CC/C=C¥C/C=C¥C/C=C¥CCCCCCCC(=O)O | 0 | validation |
| 144208924 | C(CBr)Cl | 0 | test |
| 144208923 | C(=C(Cl)Cl)(C(=C(Cl)Cl)Cl)Cl | 0 | train |
| 144208922 | C1=CC=C2C(=C1)C3=CC=CC4=C3C5=C(C=CC=C25)C=C4 | 1 | train |
| 144208921 | C1=CC(=CC=C1CC2=CC=C(C=C2)N)N | 1 | validation |
| 144208920 | C1=CC(=CC=C1C(=N)N)OCCCCCOC2=CC=C(C=C2)C(=N)N.C(CS(=O)(=O)O)O.C(CS(=O)(=O)O)O | 0 | test |
| 144208919 | C(COCCOCCOCCOCCO)O | 0 | train |
| 144208918 | CC(C)C(=O)C | 0 | train |
| 144208917 | C/C=C/C=C/C(=O)O | 0 | validation |
| 144208916 | CCCCCCCCCC(CC)C1=CC=C(C=C1)S(=O)(=O)[O-].[Na+] | 0 | test |
| 144208915 | C(C(=O)O)S | 0 | train |
| 144208914 | C1=CC(=CN=C1)C(=O)N | 0 | train |
| 144208913 | CCCCCCCCCl | 0 | validation |
| 144208912 | C1=C2C(=CC3=C1C(=O)OC3=O)C(=O)OC2=O | 0 | test |
| 144208911 | C[Si](C)(C)O[Si](C)(C)C | 0 | train |
| 144208910 | CC(C)OP(=O)(C)OC(C)C | 0 | train |
| 144208909 | C1=CC=C(C=C1)C(=O)NC2=CC=CC=C2SSC3=CC=CC=C3NC(=O)C4=CC=CC=C4 | 0 | validation |
| 144208908 | CC(COC1=CC=CC=C1)O | 0 | test |
| 144208907 | C1C=CCC2C1C(=O)N(C2=O)SC(C(Cl)Cl)(Cl)Cl | 1 | train |
| 144208906 | C1=CC=C(C=C1)NC2=CC=C(C=C2)N=O | 1 | train |
| 144208905 | C1=C(C(=CC(=C1Cl)Cl)Cl)Cl | 0 | validation |
| 144208904 | CCC(=NO)C | 0 | test |
| 144208903 | C[C@@]1(C(=O)N2[C@H](C(=O)N3CCC[C@H]3[C@@]2(O1)O)CC4=CC=CC=C4)NC(=O)[C@H]5CN([C@@H]6CC7=CNC8=CC=CC(=C78)C6=C5)C.C[C@@]1(C(=O)N2[C@H](C(=O)N3CCC[C@H]3[C@@]2(O1)O)CC4=CC=CC=C4)NC(=O)[C@H]5CN([C@@H]6CC7=CNC8=CC=CC(=C78)C6=C5)C.[C@@H]([C@H](C(=O)O)O)(C(=O)O)O | 1 | train |
| 144208902 | C1=CC(=CC=C1O)Cl | 0 | train |
| 144208901 | C1=CC=C2C(=C1)C3=CC=CC=C3N2 | 1 | validation |
| 144208900 | C1=CC(=CC=C1C(=O)O)N | 0 | test |
| 144208899 | C(CO)NCCO | 0 | train |
| 144208898 | C1=CC=C(C(=C1)C(C2=CC=C(C=C2)O)C3=CC=C(C=C3)O)C(=O)O | 0 | train |
| 144208897 | CCCCCCCCCCCCCCCO | 1 | validation |
| 144208896 | CC1=CC=C(C=C1)S(=O)(=O)N=C=O | 0 | test |
| 144208895 | CCOCCO | 0 | train |
| 144208894 | C1=CC(=C(C=C1N)N)Cl | 1 | train |
| 144208893 | C[N+](C)(CC=C)CC=C.[Cl-] | 0 | validation |
| 144208892 | CC(=C)C(=O)OCCN(C)C | 0 | test |
| 144208891 | CC1=CC=CC=N1 | 0 | train |

| | | | |
|---|---|---|---|
| 144208890 | CCCCNS(=O)(=O)C1=CC=CC=C1 | 0 | train |
| 144208889 | CCCCOC(=O)CC | 0 | validation |
| 144208888 | CCCCN1C=C[N+](=C1)C.[Cl-] | 0 | test |
| 144208887 | CC1=CN=C(S1)NC(=O)C2=C(C3=CC=CC=C3S(=O)(=O)N2C)O | 1 | train |
| 144208886 | C1=CC=C(C(=C1)C2=NN=C(N=N2)C3=CC=CC=C3Cl)Cl | 1 | train |
| 144208885 | C(#N)C1=C(C(=C(C(=C1Cl)Cl)Cl)C#N)Cl | 1 | validation |
| 144208884 | CC(=O)[C@]1(CC[C@@H]2[C@@]1(CC[C@H]3[C@H]2C=C(C4=CC(=O)[C@@H]5C[C@@H]5[C@]34C)Cl)C)OC(=O)C | 0 | test |
| 144208883 | CN1C=NC2=C1C(=O)N(C(=O)N2C)C | 1 | train |
| 144208882 | C1=CC2=C(C=C1[N+](=O)[O-])NC=N2 | 0 | train |
| 144208881 | CC(=O)OCC1=CC2=C(C=C1)OCO2 | 0 | validation |
| 144208880 | C1=CC=C(C=C1)NC(=S)N | 0 | test |
| 144208879 | CC1=C(C2=C(C=C1)C(=O)C3=CC=CC=C3C2=O)N | 1 | train |
| 144208878 | CCCCCCOC(=O)C(=C)C | 1 | train |
| 144208877 | COC(=O)NC(=S)NC1=CC=CC=C1NC(=S)NC(=O)OC | 0 | validation |
| 144208876 | C[C@@H]1[C@H]([C@H](C[C@@H](O1)O[C@@H]2[C@H](O[C@H](C[C@@H]2O)O[C@@H]3[C@H](O[C@H](C[C@@H]3O)O[C@H]4CC[C@]5([C@@H](C4)CC[C@@H]6[C@@H]5C[C@H]([C@]7([C@@]6(CC[C@@H]7C8=CC(=O)OC8)O)C)O)C)C)C)O)O | 1 | test |
| 144208875 | C1=CC=C2C(=C1)C=CN2 | 1 | train |
| 144208874 | CC(C)NC(=O)N1CC(=O)N(C1=O)C2=CC(=CC(=C2)Cl)Cl | 0 | train |
| 144208873 | CCCC[Sn](CCCC)(Cl)Cl | 1 | validation |
| 144208872 | [NH4+].NS(=O)(=O)[O-] | 0 | test |
| 144208871 | C[C@]12C[C@@H]([C@H]3[C@H]([C@@H]1CC[C@@]2(C(=O)CO)O)CCC4=CC(=O)C=C[C@]34C)O | 0 | train |
| 144208870 | CO[Si](CCCOCC1CO1)(OC)OC | 0 | train |
| 144208869 | CCCCCCCCCCCCCCCC(=O)O | 0 | validation |
| 144208868 | CCCC(=O)C | 1 | test |
| 144208867 | C1=CC(=CN=C1)C#N | 0 | train |
| 144208866 | C1=CN=CC=C1C2=CC=NC=C2 | 0 | train |
| 144208865 | CC1=C(C=CC(=C1)Cl)OC(C)C(=O)O | 0 | validation |
| 144208864 | CC(=O)CC(=O)NC1=CC(=C(C=C1OC)Cl)OC | 1 | test |
| 144208863 | CN1C(=NC(=O)N(C1=O)C2CCCCC2)N(C)C | 0 | train |
| 144208862 | CC(C)OC(=O)NC1=CC=CC=C1 | 1 | train |
| 144208861 | CN(C)N=O | 0 | validation |
| 144208860 | CCCOC(=O)C1=CC=CC=C1C(=O)OCCC | 0 | test |
| 144208859 | CC1=C(C=CC=C1N=C=O)N=C=O | 0 | train |
| 144208858 | C1C(O1)CN2C(=O)N(C(=O)N(C2=O)CC3CO3)CC4CO4 | 1 | train |
| 144208857 | C1=CC(=CC=C1C(=O)O)C(=O)O | 0 | validation |
| 144208856 | CC(=CCCC(C)(C=C)O)C | 0 | test |
| 144208855 | COC(=O)C1=CC=CC=C1N | 0 | train |
| 144208854 | C1(=C(C(=O)C(=C(C1=O)Cl)Cl)Cl)Cl | 0 | train |
| 144208853 | C1C[C@@H](O[C@@H]1CO)N2C=CC(=NC2=O)N | 0 | validation |
| 144208852 | C1=CC(=NC(=C1)N)N | 0 | test |
| 144208851 | CCC(O)OCCCOC | 1 | train |
| 144208850 | C1=CC=C2C(=C1)C(=O)C(=C(C2=O)Cl)Cl | 1 | train |
| 144208849 | CCCCCCC(C)(C)S | 0 | validation |
| 144208848 | C1=CC(=CN=C1)N | 0 | test |
| 144208847 | CO[Si](CCCNCCN)(OC)OC | 0 | train |
| 144208846 | CC(C)CC(C)(C#CC(C)(CC(C)C)O)O | 0 | train |
| 144208845 | CCNC1=CC=CC(=C1)C | 0 | validation |
| 144208844 | C(C(CO)(CO)N)O | 0 | test |
| 144208843 | C(C#N)C(=O)O | 0 | train |
| 144208842 | CC1=C(S(=O)(=O)CCS1(=O)=O)C | 1 | train |
| 144208841 | CC[Si](OC(=O)C)(OC(=O)C)OC(=O)C | 0 | validation |
| 144208840 | COC(=O)NS(=O)(=O)C1=CC=C(C=C1)N | 0 | test |
| 144208839 | COP(=O)(OC)OC(C(Cl)(Cl)Br)Br | 0 | train |
| 144208838 | CC(=C)CCl | 0 | train |
| 144208837 | C1=CC=C(C=C1)O | 0 | validation |
| 144208836 | C1=CC=C2C(=C1)C=CC3=CC=CC=C32 | 1 | test |
| 144208835 | CC=C1CC2CC1C=C2 | 0 | train |
| 144208834 | COC1=C(C=C(C=C1)[N+](=O)[O-])N | 0 | train |
| 144208833 | CCC1=CC=C(C=C1)C(C2=CC=C(C=C2)CC)C(Cl)Cl | 1 | validation |

| | | | |
|---|---|---|---|
| 144208832 | CCOP(=S)(OCC)SCSC(C)(C)C | 0 | test |
| 144208831 | CC1=CC2=C(C=C1C)N(C3=NC(=O)NC(=O)C3=N2)C[C@@H]([C@@H]([C@@H](CO)O)O)O | 0 | train |
| 144208830 | C(=O)(C(Cl)(Cl)Cl)O | 0 | train |
| 144208829 | C1=CC=C(C(=C1)C(C2=CC=C(C=C2)Cl)(C3=CN=CN=C3)O)Cl | 1 | validation |
| 144208828 | C1CC(=O)NC1 | 0 | test |
| 144208827 | C(COCCOCCOCCO)O | 0 | train |
| 144208826 | CCCCCOC(=O)C | 1 | train |
| 144208825 | C1=CC(=CC=C1N)Cl.Cl | 1 | validation |
| 144208824 | CCCC[Sn](CCCC)(CCCC)Cl | 1 | test |
| 144208823 | CCCCCCC(C)O | 1 | train |
| 144208822 | C1=CC=C(C=C1)C2=NC(=NC(=N2)N)N | 1 | train |
| 144208821 | CCC1=CC(=CC=C1)O | 0 | validation |
| 144208820 | CCCCC(CC)COP(=O)(O)OCC(CC)CCCC | 0 | test |
| 144208819 | C(#N)[S-].[Na+] | 0 | train |
| 144208818 | C1=CC=C(C=C1)[S-].[Na+] | 0 | train |
| 144208817 | CCNC1=NC(=NC(=N1)Cl)NC(C)(C)C#N | 0 | validation |
| 144208816 | CC1=NC=C(N1C)[N+](=O)[O-] | 0 | test |
| 144208815 | COP(=O)(OC)OC=C(Cl)Cl | 0 | train |
| 144208814 | C(COCCO)O | 0 | train |
| 144208812 | CCCCCCCCCCCCOS(=O)(=O)[O-].[Na+] | 0 | test |
| 144208811 | C1=CC(=C(C=C1[N+](=O)[O-])Cl)Cl | 0 | train |
| 144208810 | CCCCNC(=O)NS(=O)(=O)C1=CC=C(C=C1)C | 0 | train |
| 144208809 | C1=CC=C(C=C1)NC2=CC=CC=C2 | 0 | validation |
| 144208808 | C1=CC(=C(C=C1N)C(=O)O)O | 0 | test |
| 144208807 | CC1=C(C(=CC=C1)NC2=CC(=NC(=N2)SCC(=O)O)Cl)C | 0 | train |
| 144208806 | COC(=O)CC(=O)OC | 1 | train |
| 144208805 | COP(=O)(C(C(Cl)(Cl)Cl)O)OC | 0 | validation |
| 144208804 | C1N2CN3CN1CN(C2)C3 | 0 | test |
| 144208803 | C1=C(C(=CC(=C1Cl)Cl)Cl)OCC(=O)O | 0 | train |
| 144208802 | CC(C)CC(=O)O | 0 | train |
| 144208801 | CCOP(OCC)OCC | 0 | validation |
| 144208800 | CC(CNCC(C)O)O | 0 | test |
| 144208799 | C1(=O)[N-]C(=O)N(C(=O)N1Cl)Cl.[Na+] | 0 | train |
| 144208798 | CC1=CC(=O)N(N1C)C2=CC=CC=C2 | 0 | train |
| 144208797 | COCN(COC)C1=NC(=NC(=N1)N(COC)COC)N(COC)COC | 0 | validation |
| 144208796 | C1C(O1)COC2=CC=C(C=C2)CC3=CC=C(C=C3)OCC4CO4 | 0 | test |
| 144208795 | CCNC1=C(C=C2C(=C1)OC3=CC(=NCC)C(=CC3=C2C4=CC=CC=C4C(=O)OCC)C)C.Cl | 1 | train |
| 144208794 | C1=CC(=CC(=C1)C(=O)Cl)C(=O)Cl | 0 | train |
| 144208793 | CC1=C(C=CC(=C1)OP(=S)(OC)OC)SC | 1 | validation |
| 144208792 | CC1=CC=C(C=C1)S(=O)(=O)N | 0 | test |
| 144208791 | CCCCOCCOP(=O)(OCCOCCCC)OCCOCCCC | 0 | train |
| 144208790 | C1(=O)C2(C3(C4(C1(C5(C2(C3(C(C45Cl)(Cl)Cl)Cl)Cl)Cl)Cl)Cl)Cl)Cl | 1 | train |
| 144208789 | CC1=CC=CC(=C1)C(=O)O | 1 | validation |
| 144208788 | CC(=O)OCC1=CC=CO1 | 0 | test |
| 144208787 | C1=CC(=C(C=C1Cl)Cl)O | 0 | train |
| 144208786 | CC(COC1=CC=CC=C1)N(CCCl)CC2=CC=CC=C2.Cl | 1 | train |
| 144208785 | C[N+](C)(C)CC1=CC=CC=C1.[Cl-] | 1 | validation |
| 144208784 | CC1(C(C1C(=O)OCC2=CC(=CC=C2)OC3=CC=CC=C3)C=C(Cl)Cl)C | 1 | test |
| 144208783 | CCCCCCCCCCCO | 0 | train |
| 144208782 | CCC1=CC(=C(C(=C1)C(C)(C)C)O)C(C)(C)C | 1 | train |
| 144208781 | CCCCOCCOC(=O)C | 0 | validation |
| 144208780 | C1=CC=C(C=C1)CC=O | 0 | test |
| 144208779 | [N+](=O)([O-])[O-].[K+] | 0 | train |
| 144208778 | C1=CC=C(C(=C1)[N+](=O)[O-])[N+](=O)[O-] | 1 | train |
| 144208777 | C1=CC=C(C=C1)C2=CC(=CC=C2)C3=CC=CC=C3 | 1 | validation |
| 144208776 | C1(=C(C(=NC(=C1Cl)Cl)Cl)Cl)Cl | 1 | test |
| 144208775 | C1=CC=C(C=C1)S | 0 | train |
| 144208774 | COP(=O)(OC)OC | 0 | train |
| 144208773 | COCCl | 0 | validation |
| 144208772 | CCC(C)(CC)C1=NOC(=C1)NC(=O)C2=C(C=CC=C2OC)OC | 1 | test |
| 144208771 | CC(C)COC(=O)C(C)C | 0 | train |
| 144208770 | CCSC(=O)N1CCCCCC1 | 0 | train |

| | | | |
|---|---|---|---|
| 144208769 | COCC(=O)O | 0 | validation |
| 144208768 | C([N+](=O)[O-])(Cl)(Cl)Cl | 1 | test |
| 144208767 | CN(C)C(=S)SSC(=S)N(C)C | 1 | train |
| 144208766 | CC(C)COC(=O)C(=C)C | 1 | train |
| 144208765 | C1=CC=C2C=C3C=C(C=CC3=CC2=C1)N | 1 | validation |
| 144208764 | COC1=C(C=C(C=C1)C=O)OC | 1 | test |
| 144208763 | C1NC2=CC(=C(C=C2S(=O)(=O)N1)S(=O)(=O)N)Cl | 0 | train |
| 144208762 | C[C@]12CC(=O)[C@H]3[C@H]([C@@H]1CC[C@@]2(C(=O)CO)O)CCC4=CC(=O)C=C[C@]34C | 0 | train |
| 144208761 | CC(C)OC(=O)COC1=C(C=C(C=C1)Cl)Cl | 0 | validation |
| 144208760 | CC1(OC2=C(O1)C(=CC=C2)OC(=O)NC)C | 0 | test |
| 144208759 | CCCCC(=O)O | 0 | train |
| 144208758 | COC1=C(C(=C(C(=C1Cl)Cl)Cl)Cl)Cl | 1 | train |
| 144208757 | CC(=O)NC1=NN=C(S1)S(=O)(=O)N | 0 | validation |
| 144208756 | CCC(C)CO | 0 | test |
| 144208755 | C1CCCCCC(=O)CCCCC1 | 0 | train |
| 144208754 | C1=CC=C(C=C1)C(C#N)C2=CC=CC=C2 | 1 | train |
| 144208753 | C=CCNCC=C | 0 | validation |
| 144208752 | CN(C)C(=S)SC(=S)N(C)C | 1 | test |
| 144208751 | CCCCC(CC)COC(=O)CCCCCCCC(=O)OCC(CC)CCCC | 1 | train |
| 144208750 | C1=CC=C(C=C1)S(=O)(=O)OC2=C(C=C(C=C2)Cl)Cl | 1 | train |
| 144208749 | C=CCOC(CN1C=CN=C1)C2=C(C=C(C=C2)Cl)Cl | 1 | validation |
| 144208748 | C1=CC=C2C=C(C(=CC2=C1)C(=O)O)O | 1 | test |
| 144208747 | CCCCCCCCCCCCCCCC[N+]1=CC=CC=C1.[Br-] | 1 | train |
| 144208746 | CN=C=S | 0 | train |
| 144208745 | CC(=O)N[C@H]1CCC2=CC(=C(C(=C2C3=CC=C(C(=O)C=C13)OC)OC)OC)OC | 1 | validation |
| 144208744 | CC1=CC=C(C=C1)OP(=O)(OC2=CC=C(C=C2)C)OC3=CC=C(C=C3)C | 1 | test |
| 144208743 | C1=C(C(=CC(=C1Cl)Cl)Cl)O | 0 | train |
| 144208742 | CC1=C(C(=O)N(N1C)C2=CC=CC=C2)N(C)C | 1 | train |
| 144208741 | COC(=O)C1=CC=CC=C1O | 1 | validation |
| 144208740 | C1=CC(=CC=C1C2=CC(=O)C3=C(C=C(C=C3O2)O)O)O | 1 | test |
| 144208739 | CCCCC(CC)COC(=O)CS | 1 | train |
| 144208738 | C1CCC(CC1)SN2C(=O)C3=CC=CC=C3C2=O | 0 | train |
| 144208737 | C1=CC(=CC=C1N/N=C¥2/C(=NN(C2=O)C3=CC=C(C=C3)S(=O)(=O)[O-])C(=O)[O-])S(=O)(=O)[O-].[Na+].[Na+].[Na+] | 0 | validation |
| 144208736 | C1=CC2=C(C=CC(=C2)S(=O)(=O)[O-])C=C1O.[Na+] | 0 | test |
| 144208735 | CC(C)CC(C)CC(=O)CC(C)C | 0 | train |
| 144208734 | C(C(C(=O)O)O)C(=O)O | 0 | train |
| 144208733 | CCCCC(CC)COC(=O)C(=C)C | 0 | validation |
| 144208732 | COP(=O)(C)OC | 0 | test |
| 144208731 | CCC1=CC=C(C=C1)O | 0 | train |
| 144208730 | C1C=CCS1(=O)=O | 0 | train |
| 144208729 | CC1(C(C1(C)C)C(=O)OC(C#N)C2=CC(=CC=C2)OC3=CC=CC=C3)C | 1 | validation |
| 144208728 | CC1=C(C=CC=C1COC(=O)C2C(C2(C)C)/C=C(/C(F)(F)F)¥Cl)C3=CC=CC=C3 | 0 | test |
| 144208727 | CC1=CC(=C(C(=C1)C(C)(C)C)O)CC2=C(C(=CC(=C2)C)C(C)(C)C)O | 1 | train |
| 144208726 | CC(C)CCCCCCC1=CC=C(C=C1)O | 1 | train |
| 144208725 | CC(=O)NCCC1=CNC2=C1C=C(C=C2)OC | 0 | validation |
| 144208724 | C=CC(=O)N | 0 | test |
| 144208723 | CSSC | 0 | train |
| 144208722 | CC1=CC(=C(C=C1C2(C3=CC=CC=C3S(=O)(=O)O2)C4=CC(=C(C=C4C)O)C)C)O | 0 | train |
| 144208721 | CC1=C(C=C(C=C1)O)C | 0 | validation |
| 144208720 | C1=CC(=C(C=C1Cl)Cl)N | 0 | test |
| 144208719 | C1=CC=C2C(=C1)C(=O)C3=C(C2=O)C=C(C=C3)N | 1 | train |
| 144208718 | CC1(CC(CC(C1)(C)CN=C=O)N=C=O)C | 1 | train |
| 144208717 | CCCCCOC(=O)C1=CC=CC=C1C(=O)OCCCCC | 1 | validation |
| 144208716 | CC(=O)NC1=CC2=C(C=C1)C3=C(C2)C=C(C=C3)NC(=O)C | 1 | test |
| 144208715 | CCCCCC(=O)O | 0 | train |
| 144208714 | CC(=C)C(=O)NC1=CC(=C(C=C1)Cl)Cl | 1 | train |
| 144208713 | CC(C)(C)C1CCC(CC1)O | 0 | validation |
| 144208712 | B(OC(C)C)(OC(C)C)OC(C)C | 0 | test |
| 144208711 | C(#N)N=C(N)N | 0 | train |
| 144208710 | CCOC(=O)/C=C¥C(=O)OCC | 0 | train |

| | | | |
|---|---|---|---|
| 144208709 | C1=CC=C(C=C1)OP(=O)(OC2=CC=CC=C2)OC3=CC=CC=C3 | 1 | validation |
| 144208708 | CC(C(=O)O)(Cl)Cl | 0 | test |
| 144208707 | C[C@H]1C(=O)O[C@@H]2CCN3[C@@H]2C(=CC3)COC(=O)[C@]([C@]1(C)O)(C)O | 0 | train |
| 144208706 | CN(C1=CC=CC=C1)N=O | 0 | train |
| 144208705 | CC1=CC=C(C=C1)S(=O)(=O)NC(=O)NN2CCCCCC2 | 0 | validation |
| 144208704 | C[C@]12CC[C@H]3[C@H]([C@@H]1CC[C@@H]2C(=O)NC(C)(C)C)CC[C@@H]4[C@@]3(C=CC(=O)N4)C | 0 | test |
| 144208703 | C1CC2C(O2)CC1C3CO3 | 0 | train |
| 144208702 | C1=CN=CN1 | 0 | train |
| 144208701 | C1CNP(=O)(OC1)N(CCCl)CCCl.O | 0 | validation |
| 144208700 | CC(=O)C=CC1=CC=CC=C1 | 0 | test |
| 144208699 | C1=CC2=NNN=C2C=C1 | 0 | train |
| 144208698 | CC1=CC(=CC(=C1N)C)C2=CC(=C(C(=C2)C)N)C | 0 | train |
| 144208697 | CC(=O)C1=CC=CC=N1 | 0 | validation |
| 144208696 | COC1=CC=C(C=C1)N.Cl | 0 | test |
| 144208695 | C1=CC(=C(C(=C1Cl)Cl)Cl)Cl | 0 | train |
| 144208694 | CCN(CC)C1=CC=CC=C1 | 0 | train |
| 144208693 | C1=CC(=CC=C1C(C2=CC=C(C=C2)Cl)C(Cl)(Cl)Cl)Cl | 1 | validation |
| 144208692 | CC(=C)C(=O)OCC1CO1 | 0 | test |
| 144208691 | C1=CC=C(C=C1)N(N=O)[O-].[NH4+] | 1 | train |
| 144208690 | CCCCCCC(CCCCCCCCCCC(=O)O)O | 0 | train |
| 144208689 | CCCCC(CC)COC(=O)Cl | 0 | validation |
| 144208688 | CC(C)(C#N)O | 0 | test |
| 144208687 | C(CO)N1C(=O)N(C(=O)N(C1=O)CCO)CCO | 0 | train |
| 144208686 | CCCCCCCCCCCC(=O)OC | 0 | train |
| 144208685 | C([C@H]([C@H]([C@@H]([C@@H](CO)O)O)O)O)O | 0 | validation |
| 144208684 | CN1C(=CC(=[N+]1C)C2=CC=CC=C2)C3=CC=CC=C3.COS(=O)(=O)[O-] | 0 | test |
| 144208683 | CC(COCC(C)O)O | 0 | train |
| 144208682 | C[Si](CN1C=NC=N1)(C2=CC=C(C=C2)F)C3=CC=C(C=C3)F | 1 | train |
| 144208681 | CC(=O)CCC(=O)O | 0 | validation |
| 144208680 | C1CCCNCC1 | 0 | test |
| 144208679 | C1CCC(CC1)O | 0 | train |
| 144208678 | CCCCCCCC(=O)OCC(COC(=O)CCCCCCC)OC(=O)CCCCCCC | 0 | train |
| 144208677 | CCCCCCCCC | 0 | validation |
| 144208676 | C1=CC=C2C(=C1)C=CC=N2 | 0 | test |
| 144208675 | CCN1C=C(C(=O)C2=C1N=C(C=C2)C)C(=O)O | 0 | train |
| 144208674 | CCNC(=S)NCC | 0 | train |
| 144208673 | CC[C@H](C)C(=O)O[C@H]1C[C@H](C=C2[C@H]1[C@H]([C@H](C=C2)C)CC[C@@H]3C[C@H](CC(=O)O3)O)C | 0 | validation |
| 144208672 | C1=CC(=CC(=C1)C#N)C#N | 0 | test |
| 144208671 | C1=CC=C(C=C1)OC2=CC=CC(=C2)CO | 1 | train |
| 144208670 | CN(C)CCOC(C1=CC=CC=C1)C2=CC=CC=C2.Cl | 0 | train |
| 144208669 | C1=CC=C(C(=C1)C(=O)O)O | 0 | validation |
| 144208668 | CCCCCCCC(=O)OC | 0 | test |
| 144208667 | CCCC1COC(O1)(CN2C=NC=N2)C3=C(C=C(C=C3)Cl)Cl | 1 | train |
| 144208666 | C1[C@H]([C@H](OC2=CC(=CC(=C21)O)O)C3=CC(=C(C(=C3)O)O)O)OC(=O)C4=CC(=C(C(=C4)O)O)O | 0 | train |
| 144208665 | C1=CC(=CC=C1C(C2=CC=C(C=C2)O)(C(F)(F)F)C(F)(F)F)O | 1 | validation |
| 144208664 | CCCN(CCOC1=C(C=C(C=C1Cl)Cl)Cl)C(=O)N2C=CN=C2 | 1 | test |
| 144208663 | C1=CC(=C(C=C1[As](=O)(O)O)[N+](=O)[O-])O | 0 | train |
| 144208662 | CC1=CC=C(C=C1)[N+](=O)[O-] | 0 | train |
| 144208661 | CCN(CC)CCO | 0 | validation |
| 144208660 | C1=CC(=C(C=C1[N+](=O)[O-])[N+](=O)[O-])O | 0 | test |
| 144208659 | CC(C)(C)OOC(C)(C)C | 0 | train |
| 144208658 | COC1=CC=CC=C1N | 0 | train |
| 144208657 | CCCCCCCCN1C(=O)C=CS1 | 1 | validation |
| 144208656 | C1=CC=C(C=C1)N=NC2=CC=CC=C2 | 0 | test |
| 144208655 | C1=CC=C(C=C1)N(C2=CC=CC=C2)N=O | 0 | train |
| 144208654 | CC(C)(C)CC(C)(C)C1=CC(=C(C=C1)O)N2N=C3C=CC=CC3=N2 | 1 | train |
| 144208653 | CCC12COCN1COC2 | 0 | validation |
| 144208652 | CC1=C(C(CCC1)(C)C)/C=C/C(=O)C | 0 | test |
| 144208651 | C(CNCCN)N | 0 | train |

| | | | |
|---|---|---|---|
| 144208650 | CC1=CC(=CC(=C1)C)C | 0 | train |
| 144208649 | CC(C)(C)C1=NN=C(S1)N(C)C(=O)NC | 0 | validation |
| 144208648 | C1=CC(=C(C(=C1C(=O)C2=CC(=C(C(=C2)O)O)O)O)O)O | 0 | test |
| 144208647 | C1(=C(C(=C(C(=C1Cl)Cl)C(=O)Cl)Cl)Cl)C(=O)Cl | 0 | train |
| 144208646 | CCOC1=C(C=CC(=C1)OC2=C(C=C(C=C2)C(F)(F)F)Cl)[N+](=O)[O-] | 1 | train |
| 144208645 | C([C@H]([C@@H]1[C@@H]([C@@H]2[C@H](O1)O[C@@H](O2)C(Cl)(Cl)Cl)O)O)O | 0 | validation |
| 144208644 | CCO[Si](CCCN)(OCC)OCC | 0 | test |
| 144208643 | C1=CC(=CC=C1C(F)(F)F)Cl | 0 | train |
| 144208642 | C1=CC(=CC=C1C2=COC3=C(C2=O)C=CC(=C3)O)O | 1 | train |
| 144208641 | CC(C)OC(=O)C | 0 | validation |
| 144208640 | C1=CC=C(C(=C1)[N+](=O)[O-])O | 0 | test |
| 144208639 | CC1=CC[C@@H](CC1=O)C(=C)C | 0 | train |
| 144208638 | CC1=C(C=CC(=C1)OC)N | 0 | train |
| 144208637 | CN(C)C(=O)NC1=CC=C(C=C1)Cl | 0 | validation |
| 144208636 | CN(C)C(=O)NC1=CC(=C(C=C1)Cl)Cl | 1 | test |
| 144208635 | CC(CO)OCC(C)OCC(C)OC | 0 | train |
| 144208634 | CC(COC)OC(=O)C | 0 | train |
| 144208633 | C1=CC(=NC(=C1Cl)C(=O)O)Cl | 0 | validation |
| 144208632 | C1CCCN(CCC1)N=O | 0 | test |
| 144208631 | C(C(=O)CCl)Cl | 0 | train |
| 144208630 | CC1([C@H]([C@H]1C(=O)O[C@H](C#N)C2=CC(=CC=C2)OC3=CC=CC=C3)C=C(Br)Br)C | 1 | train |
| 144208629 | CC(C)(C)CCCCCCOC(=O)C1=CC=CC=C1C(=O)OCCCCCCC(C)(C)C | 0 | validation |
| 144208628 | C=CC(=O)OCCO | 0 | test |
| 144208627 | C1=CC(=C(C=C1Cl)CC2=C(C=CC(=C2)Cl)O)O | 0 | train |
| 144208626 | C1=CC(=CC=C1C2=COC3=CC(=CC(=C3C2=O)O)O)O | 1 | train |
| 144208625 | C(CBr)Br | 0 | validation |
| 144208624 | CC1=C(C(=CC=C1)C)N | 0 | test |
| 144208623 | COC1=CC2=C(C=C1)N=C(S2)N | 1 | train |
| 144208622 | CC(C)NC1=CC=C(C=C1)NC2=CC=CC=C2 | 1 | train |
| 144208621 | CC1=C(C(CCC1)(C)C)/C=C/C(=C/C=C/C(=C/COC(=O)C)/C)/C | 1 | validation |
| 144208620 | CCCCC(CC)COC(=O)/C=C¥C(=O)OCC(CC)CCCC | 1 | test |
| 144208619 | C1=C(C=C(C(=C1Br)O)Br)C#N | 1 | train |
| 144208618 | C1=CC(=CN=C1)C(=O)O | 0 | train |
| 144208617 | C1COS(=O)(=O)C1 | 0 | validation |
| 144208616 | CC1=CC(=C(C=C1)C(C)(C)C)O | 0 | test |
| 144208615 | CC(C(=O)O)Br | 0 | train |
| 144208614 | CCCC[N+]1=CC=CC=C1.[Cl-] | 0 | train |
| 144208613 | CCCCCCCC=O | 0 | validation |
| 144208612 | CCNC1=NC(=NC(=N1)SC)NC(C)C | 1 | test |
| 144208611 | CC(C)(C#C)NC(=O)C1=CC(=CC(=C1)Cl)Cl | 0 | train |
| 144208610 | CC(CC1=CC=C(C=C1)C(C)(C)C)C=O | 0 | train |
| 144208609 | CC1=C(C=C(C=C1)N)N | 1 | validation |
| 144208608 | C1=CC=C(C(=C1)O)Cl | 0 | test |
| 144208607 | CCOP(=S)(OCC)OC1=NC(=NC(=C1)C)C(C)C | 0 | train |
| 144208606 | CC1=CC=C(C=C1)Cl | 0 | train |
| 144208605 | C1=CC=C2C(=C1)C=CC=C2[N+](=O)[O-] | 0 | validation |
| 144208604 | CCCCC(CC)C(=O)O | 0 | test |
| 144208603 | CC(=O)CC(=O)NC1=CC=CC=C1 | 0 | train |
| 144208602 | C1=CC(=CC=C1N/C(=N/C(=NCCCCCCN=C(/N=C(/NC2=CC=C(C=C2)Cl)¥N)N)N)/N)Cl | 1 | train |
| 144208601 | CCCCC/C=C¥C/C=C¥CCCCCCCC(=O)OC | 0 | validation |
| 144208600 | C1CC(C(CCC(C(CCC(C1Br)Br)Br)Br)Br)Br | 0 | test |
| 144208599 | CC1(CC(CC(C1)(C)CN)N)C | 0 | train |
| 144208598 | CC1=CC=NC=C1 | 0 | train |
| 144208597 | C1=CC=C(C=C1)OCCOC2=CC=CC=C2 | 1 | validation |
| 144208596 | C[Si]1(O[Si](O[Si](O[Si](O1)(C)C)(C)C)(C)C)C | 0 | test |
| 144208595 | C([C@@H]1[C@H]([C@@H]([C@H]([C@H](O1)O[C@]2([C@H]([C@@H]([C@H](O2)CO)O)O)CO)O)O)O)O | 0 | train |
| 144208594 | CC(=O)NC1=CC(=C(C=C1)OC)N | 0 | train |
| 144208593 | CS(=O)(=O)NC(=O)C1=C(C=CC(=C1)OC2=C(C=C(C=C2)C(F)(F)F)Cl)[N+](=O)[O-] | 0 | validation |
| 144208592 | CC(C)NC1=NC(=NC(=N1)Cl)NC(C)C | 0 | test |
| 144208591 | CC1=CN(C(=O)NC1=O)[C@H]2C=C[C@H](O2)CO | 0 | train |
| 144208590 | CCCOCC(C)O | 0 | train |

| | | | |
|---|---|---|---|
| 144208589 | CCCNC(=O)NS(=O)(=O)C1=CC=C(C=C1)Cl | 0 | validation |
| 144208588 | C1=CC(=CC=C1CCCC(=O)O)N(CCCl)CCCl | 0 | test |
| 144208587 | CN(C)C1=CC=C(C=C1)CC2=CC=C(C=C2)N(C)C | 1 | train |
| 144208586 | CC1=C(C(=CC=C1)C)[N+](=O)[O-] | 0 | train |
| 144208585 | CCCCOC(=O)C=C | 0 | validation |
| 144208584 | C1(=C(N=C(C(=N1)Cl)N)N)C(=O)N=C(N)N.Cl | 0 | test |
| 144208583 | C[C@H]1/C=C/C=C(¥C(=O)NC¥2=C(C3=C(C(=C4C(=C3C(=O)/C2=C¥NN5CCN(CC5)C)C(=O)[C@](O4)(O/C=C/[C@@H]([C@H]([C@H]([C@@H]([C@@H]([C@@H]([C@H]1O)C)O)C)OC(=O)C)C)OC)C)C)O)O)/C | 1 | train |
| 144208582 | CC(C)CC1=CC=C(C=C1)C(C)C(=O)O | 1 | train |
| 144208581 | CCCCCCCCOC1=CC(=C(C=C1)C(=O)C2=CC=CC=C2)O | 0 | validation |
| 144208580 | CCCCCCCCCCC=C | 0 | test |
| 144208579 | CC(=CCCC(=O)C)C | 0 | train |
| 144208578 | CC(C)C1=CC=C(C=C1)C(C)C | 0 | train |
| 144208577 | C1=CC(=CC=C1F)Br | 0 | validation |
| 144208576 | CC(C(=O)[O-])O.CC(C(=O)[O-])O.[Ca+2] | 0 | test |
| 144208575 | C1=CC(=C(C=C1Cl)Cl)OCC(=O)[O-].[Na+] | 0 | train |
| 144208574 | CCC1=CC=CC(=C1N(C(C)COC)C(=O)CCl)C | 1 | train |
| 144208573 | C[C@]12CC[C@H]3[C@H]([C@@H]1CCC2=O)CCC4=C3C=CC(=C4)O | 1 | validation |
| 144208572 | C1=CC(=CC=C1/C=C/C2=CC(=CC(=C2)O)O)O | 1 | test |
| 144208571 | CCCCCCCCO | 0 | train |
| 144208570 | CCC(C)C1=CC=CC=C1O | 0 | train |
| 144208569 | CC(C)C1=C(C(=CC=C1)C(C)C)O | 0 | validation |
| 144208568 | CC1=CC=C(C=C1)N(C)C | 0 | test |
| 144208567 | CCCCC(CC)COP(=O)(OCC(CC)CCCC)OCC(CC)CCCC | 0 | train |
| 144208566 | C1=C(C=C(C(=C1N)Cl)C(=O)O)Cl | 0 | train |
| 144208565 | C1=CN=CC=C1C(=O)NN | 0 | validation |
| 144208564 | C[C@]12CCC(=O)C=C1CC[C@@H]3[C@@H]2[C@H](C[C@]4([C@H]3CC[C@@H]4C(=O)CO)C)O | 0 | test |
| 144208563 | C(=S)(NN)NN | 0 | train |
| 144208562 | CN(C)CCC#N | 0 | train |
| 144208561 | CC(C)(CO)CO | 0 | validation |
| 144208560 | CCOC(=O)CC(=O)OCC | 0 | test |
| 144208559 | C=CC(CCl)Cl | 0 | train |
| 144208558 | CN(C)C1CCCCC1 | 0 | train |
| 144208557 | CC(C)(C(=O)O)OC1=CC=C(C=C1)C2CC2(Cl)Cl | 0 | validation |
| 144208556 | COC1=CC=CC=C1N.Cl | 1 | test |
| 144208555 | CC1=CC(=C(C=C1)N=CN(C)C=NC2=C(C=C(C=C2)C)C)C | 0 | train |
| 144208554 | COC1=C(C=CC(=C1C(=O)O)Cl)Cl | 0 | train |
| 144208553 | CC1CC2=C(CC1(C)C(=O)C)C(CCC2)(C)C | 0 | validation |
| 144208552 | CC(=C)C#N | 0 | test |
| 144208551 | CC(C)(C)C(=O)C1C(=O)C2=CC=CC=C2C1=O | 1 | train |
| 144208550 | CCCN(CCC)C1=C(C=C(C=C1[N+](=O)[O-])C(F)(F)F)[N+](=O)[O-] | 1 | train |
| 144208549 | C1=CC(=C(C=C1[N+](=O)[O-])N)O | 0 | validation |
| 144208548 | C1=C(C(=C(C(=C1Cl)Cl)CC2=C(C(=CC(=C2Cl)Cl)Cl)O)O)Cl | 1 | test |
| 144208547 | C=CC(=O)OCCCCCCOC(=O)C=C | 0 | train |
| 144208546 | C(=S)(N)NN | 1 | train |
| 144208545 | C1=CC(=CC=C1N)S(=O)(=O)C2=CC=C(C=C2)N | 0 | validation |
| 144208544 | CC(C)CCC(=O)C | 0 | test |
| 144208543 | COC(=O)C1=CC(=CC=C1)C(=O)OC | 0 | train |
| 144208542 | CCCCCCCCCCCCC1=CC=CC=C1 | 0 | train |
| 144208541 | C1=CC=NC(=C1)C#N | 0 | validation |
| 144208540 | CCCCC(CC)COC(=O)CC(C(=O)OCC(CC)CCCC)S(=O)(=O)[O-].[Na+] | 0 | test |
| 144208539 | CN1C=CNC1=S | 0 | train |
| 144208538 | C1COCCN1SSC2=NC3=CC=CC=C3S2 | 1 | train |
| 144208537 | CCC1=CN=C(C(=C1)C(=O)O)C2=NC(C(=O)N2)(C)C(C)C | 0 | validation |
| 144208536 | CC(C)(/C=N/OC(=O)NC)S(=O)(=O)C | 0 | test |
| 144208535 | C1=CC=C(C=C1)COC(=O)C2=CC=CC=C2 | 0 | train |
| 144208534 | CCOC1=CC2=C(C=C1)NC(C=C2C)(C)C | 1 | train |
| 144208533 | CCCCCCO | 0 | validation |
| 144208532 | CN(C)C(=O)Cl | 0 | test |
| 144208531 | COC1=CC=C(C=C1)OC | 0 | train |

| | | | |
|---|---|---|---|
| 144208530 | CC1=C(C=C(C=C1)[N+](=O)[O-])C | 0 | train |
| 144208529 | C1=CC(=CC=C1N)S(=O)(=O)N | 1 | validation |
| 144208528 | C1=CC(=CC(=C1)Cl)[N+](=O)[O-] | 0 | test |
| 144208527 | C1=CC=NC(=C1)CCl.Cl | 0 | train |
| 144208526 | CCOP(=O)(Cl)Cl | 0 | train |
| 144208525 | CCCCC(CC)COC(=O)C=CC1=CC=C(C=C1)OC | 0 | validation |
| 144208524 | [O-]S(=O)(=O)OOS(=O)(=O)[O-].[Na+].[Na+] | 0 | test |
| 144208523 | C1=CC=C2C(=C1)C(=O)OC(=O)N2 | 0 | train |
| 144208522 | C1CC(OC1)CO | 0 | train |
| 144208521 | C1CN2CCN1CC2 | 0 | validation |
| 144208520 | C1=CC=C(C=C1)CCO | 0 | test |
| 144208519 | CC1=CC=CC2=NNN=C12 | 0 | train |
| 144208518 | CCOCCOCCOCCO | 0 | train |
| 144208517 | CC1=C(C=CC(=C1)Cl)OCCCC(=O)O | 0 | validation |
| 144208516 | CC(C)C1=CC(=CC=C1)C(C)C | 0 | test |
| 144208515 | CCCCOCCOCCSC#N | 0 | train |
| 144208514 | CC1=NC=CN1 | 0 | train |
| 144208513 | CCOC(=O)C1C(O1)(C)C2=CC=CC=C2 | 1 | validation |
| 144208512 | CC(=O)NC1=CC=C(C=C1)C(=O)CCl | 1 | test |
| 144208511 | C1=CC=C(C=C1)C(Cl)Cl | 0 | train |
| 144208510 | C1=CC(=C(C=C1[N+](=O)[O-])N)C(=O)O | 0 | train |
| 144208509 | C1=CC(=C(C=C1Cl)Cl)Cl | 0 | validation |
| 144208508 | C1=CC(=C(C=C1N)Cl)N.OS(=O)(=O)O | 1 | test |
| 144208507 | C1C(O1)CO | 0 | train |
| 144208506 | CC1=CC=CC=C1NC(=O)CC(=O)C | 0 | train |
| 144208505 | CC(C)CC(=O)OCC=C | 0 | validation |
| 144208504 | CC(=CCCC(C)(C=C)OC(=O)C)C | 0 | test |
| 144208503 | C(=O)/C(=C(¥C(=O)O)/Cl)/Cl | 0 | train |
| 144208502 | CCCCCCCCCCCC#N | 0 | train |
| 144208501 | CCCCCCCCCCN | 0 | validation |
| 144208500 | CC(C)(CO)COC(=O)C(C)(C)CO | 0 | test |
| 144208499 | C(CCCCCC(=O)O)CCCCCC(=O)O | 0 | train |
| 144208498 | CCOC(=O)C(C)OC(=O)C1=C(C=CC(=C1)OC2=C(C=C(C=C2)C(F)(F)F)Cl)[N+](=O)[O-] | 1 | train |
| 144208497 | C=C(CC(=O)O)C(=O)O | 0 | validation |
| 144208496 | C1(=C(C(=NC(=C1Cl)Cl)C(=O)O)Cl)N | 1 | test |
| 144208495 | CC(C)(C1=CC=CC=C1)C2=CC=C(C=C2)O | 1 | train |
| 144208494 | C1=CC(=CC=C1C(C2=CC=C(C=C2)Cl)C(Cl)Cl)Cl | 1 | train |
| 144208493 | CC1=CC(=CC=C1)NC(=O)OC2=CC=CC(=C2)NC(=O)OC | 1 | validation |
| 144208492 | C1CNC(=S)N1 | 1 | test |
| 144208491 | CC1=C(C=CC(=C1)O)Cl | 0 | train |
| 144208490 | CC1=C(C=CC(=C1)C2=CC(=C(C=C2)N)C)N | 1 | train |
| 144208489 | C[C@]12CC[C@H]3[C@H]([C@@H]1CC[C@]2(C#C)O)CCC4=C3C=CC(=C4)O | 0 | validation |
| 144208488 | C1=CC=C(C=C1)C(Cl)(Cl)Cl | 0 | test |
| 144208487 | C1CCC(CC1)N.Cl | 0 | train |
| 144208486 | C1=CC(=CC=C1Br)Br | 0 | train |
| 144208485 | C(COCCO)N | 0 | validation |
| 144208484 | CCC/C=C/C=O | 0 | test |
| 144208483 | C1=CC=C(C=C1)C(=O)C2=C(C=C(C=C2)O)O | 1 | train |
| 144208482 | C(C#CCO)O | 0 | train |
| 144208481 | CC1=C(C=CC(=C1)Cl)N.Cl | 0 | validation |
| 144208480 | CN(C)C(=O)C(C1=CC=CC=C1)C2=CC=CC=C2 | 1 | test |
| 144208479 | CN(C(=O)NC1=CC(=C(C=C1)Cl)Cl)OC | 1 | train |
| 144208478 | CC(=O)CC(=O)NC1=CC=CC=C1OC | 0 | train |
| 144208477 | CCCCCCCC(=O)O | 0 | validation |
| 144208476 | CN(C)CCC=C1C2=CC=CC=C2CCC3=CC=CC=C31.Cl | 1 | test |
| 144208475 | CC1=CC=CC=C1N.Cl | 0 | train |
| 144208474 | C1C=CCC2C1C(=O)N(C2=O)SC(Cl)(Cl)Cl | 0 | train |
| 144208473 | CC1=CC(=C(C=C1)C)O | 0 | validation |
| 144208472 | CC1=C(C(=CC=C1)C)O | 0 | test |
| 144208471 | C1=C(C=C(C(=C1Br)O)Br)Br | 0 | train |
| 144208470 | CC(C)NCC(C1=CC(=C(C=C1)O)O)O.Cl | 0 | train |
| 144208469 | C(C(C(CC(=O)O)C(=O)O)C(=O)O)C(=O)O | 0 | validation |

| | | | |
|---|---|---|---|
| 144208468 | CC(=O)OCCOCCOCCOC(=O)C | 0 | test |
| 144208467 | C1=CC=C(C=C1)OC2=CC=C(C=C2)Br | 0 | train |
| 144208466 | CCCCC(CC)C(=O)Cl | 0 | train |
| 144208465 | CCN(CC)C(=S)[S-].O.O.O.[Na+] | 1 | validation |
| 144208464 | CC(=O)CC(C)(C)O | 0 | test |
| 144208463 | CC1=CC(=CC=C1)N | 0 | train |
| 144208462 | C1=CC=C(C(=C1)C(F)(F)F)[N+](=O)[O-] | 0 | train |
| 144208461 | C1=CC(=CC=C1OS(=O)(=O)C2=CC=C(C=C2)Cl)Cl | 1 | validation |
| 144208460 | C[C@]12CC[C@H]3[C@H]([C@@H]1CC[C@H]2O)CCC4=C3C=CC(=C4)O | 1 | test |
| 144208459 | CNC[C@@H](C1=CC(=CC=C1)O)O.Cl | 0 | train |
| 144208458 | C1=CC(=CC=C1C(=C(Cl)Cl)C2=CC=C(C=C2)Cl)Cl | 1 | train |
| 144208457 | CN(C)C=O | 0 | validation |
| 144208456 | C1=CC(=CC=C1C(=O)O)[N+](=O)[O-] | 0 | test |
| 144208455 | CC(C)CCOC(=O)C | 0 | train |
| 144208454 | CC1=CC=CC=C1N | 0 | train |
| 144208453 | CCN(CC)N=O | 0 | validation |
| 144208452 | C(CCl)OP(=O)(OCCCl)OCCCl | 0 | test |
| 144208451 | CC1CC(=O)O1 | 0 | train |
| 144208450 | C([C@@H]1[C@@H]2[C@@H]([C@H]([C@H](O1)O[C@@H]3[C@H](O[C@@H]([C@@H]([C@H]3O)O)O[C@@H]4[C@H](O[C@@H]([C@@H]([C@H]4O)O)O[C@@H]5[C@H](O[C@@H]([C@@H]([C@H]5O)O)O[C@@H]6[C@H](O[C@@H]([C@@H]([C@H]6O)O)O[C@@H]7[C@H](O[C@H](O2)[C@@H]([C@H]7O)O)CO)CO)CO)CO)CO)O)O)O | 0 | train |
| 144208449 | CC1=CC=CC=C1Cl | 0 | validation |
| 144208448 | COCCCN | 0 | test |
| 144208447 | C[Sn](C)(Cl)Cl | 0 | train |
| 144208446 | C1=CC(=C(C=C1N)N)O.Cl.Cl | 0 | train |
| 144208445 | CCCCCCCO | 0 | validation |
| 144208444 | C1=CC=C2C(=C1)C(=CN2)C[C@@H](C(=O)O)N | 0 | test |
| 144208443 | C1=CC2=C(C=CC(=C2)S(=O)(=O)[O-])C=C1O.[K+] | 0 | train |
| 144208442 | CC1=C(C(=O)N(C(=O)N1)C(C)(C)C)Cl | 0 | train |
| 144208441 | CC/C(=C(¥C1=CC=CC=C1)/C2=CC=C(C=C2)OCCN(C)C)/C3=CC=CC=C3 | 1 | validation |
| 144208440 | CCC(C)C=O | 0 | test |
| 144208439 | CCCCCCCCCC1=CC=C(C=C1)O | 1 | train |
| 144208438 | CC(=O)CCC1=CC=CC=C1 | 0 | train |
| 144208437 | C1=CC=NC(=C1)NS(=O)(=O)C2=CC=C(C=C2)N/N=C¥3/C=CC(=O)C(=C3)C(=O)O | 0 | validation |
| 144208436 | C1=CC(=CC(=C1)[N+](=O)[O-])C(=O)O | 0 | test |
| 144208435 | CCCCOC(=O)COC1=C(C=C(C=C1)Cl)Cl | 0 | train |
| 144208434 | CN1CN(C(=S)SC1)C | 1 | train |
| 144208433 | COC(=O)CCCC(=O)OC | 0 | validation |
| 144208432 | CCCCNC(=O)N1C2=CC=CC=C2N=C1NC(=O)OC | 1 | test |
| 144208431 | C1=CC=C2C(=C1)C=CC=C2N | 1 | train |
| 144208430 | CC(COC(=O)C(=C)C)O | 0 | train |
| 144208429 | C1=NN(C(=O)N=C1N)[C@H]2[C@@H]([C@@H]([C@H](O2)CO)O)O | 0 | validation |
| 144208428 | COC1=CC(=C(C=C1)N)OC.Cl | 0 | test |
| 144208427 | C1=CC=C(C=C1)C(=O)C2=CC=C(C=C2)O | 1 | train |
| 144208426 | CC1=C(C=CC=C1[N+](=O)[O-])C(=O)O | 0 | train |
| 144208425 | CC(C)(CCl)C(=O)Cl | 0 | validation |
| 144208424 | CC(=O)OC1=CC=CC=C1C(=O)NC2=NC=C(S2)[N+](=O)[O-] | 1 | test |
| 144208423 | CCSC(=O)Cl | 0 | train |
| 144208422 | CC1=CC=CC=C1 | 0 | train |
| 144208421 | C1=CC=C(C=C1)Cl | 1 | validation |
| 144208420 | CC(=O)C(Cl)(Cl)Cl | 1 | test |
| 144208419 | CC1=CC(=C(C=C1)OC)[N+](=O)[O-] | 0 | train |
| 144208418 | C1=CC=C(C=C1)C(=O)O[C@H]([C@H](C(=O)O)OC(=O)C2=CC=CC=C2)C(=O)O | 0 | train |
| 144208417 | CC(=O)C1=CC=C(C=C1)C(=O)C | 0 | validation |
| 144208416 | CC(=O)C(=O)C | 0 | test |
| 144208415 | C1=CC(=C(C=C1Cl)Cl)C=O | 0 | train |
| 144208414 | CC(C)CC(C)CC(C)/C=C(¥C)/C1CC(=O)OC1=O | 0 | train |
| 144208413 | C(C(=O)C(=O)O)C(=O)O | 0 | validation |
| 144208412 | CC(C)CO | 0 | test |
| 144208411 | CCCCCCC | 0 | train |
| 144208410 | C[C@]12CC[C@H]3[C@H]([C@@H]1CC[C@@H]2O)CCC4=CC(=O)CC[C@H]34 | 1 | train |

| | | | |
|---|---|---|---|
| 144208409 | C1OC2=C(O1)C=C(C=C2)O | 0 | validation |
| 144208408 | CC1=CC(=CC(=C1O)C)C(C)(C)C2=CC(=C(C(=C2)C)O)C | 1 | test |
| 144208407 | C1(C(=C(C(=O)O1)Cl)C(Cl)Cl)O | 0 | train |
| 144208406 | CN(C)CCCCl.Cl | 0 | train |
| 144208405 | CC(C)C(=O)O | 0 | validation |
| 144208404 | CC/C=C/1¥C2=CC=CC=C2C(=O)O1 | 0 | test |
| 144208403 | C[C@@]12[C@H]3C[C@@H]([C@@H]1C(=O)OC2=O)C=C3 | 0 | train |
| 144208402 | CC(C)(C(=O)NC1=CC(=C(C=C1)[N+](=O)[O-])C(F)(F)F)O | 1 | train |
| 144208401 | C[C@H]1CCC[C@@H](CCCCCC2=CC(=CC(=C2C(=O)O1)O)O)O | 0 | validation |
| 144208400 | C(CN)N | 0 | test |
| 144208399 | CC(CCl)OC(C)CCl | 0 | train |
| 144208398 | CC1=NC(=NC(=N1)OC)NC(=O)NS(=O)(=O)C2=CC=CC=C2OCCCl | 0 | train |
| 144208397 | CN(CCO)CCO | 0 | validation |
| 144208396 | C1=CC(=CC=C1CCN)O.Cl | 0 | test |
| 144208395 | CCCCCCCCCCCCCCC(=O)O | 0 | train |
| 144208394 | C(=C(Cl)Cl)(Cl)Cl | 0 | train |
| 144208393 | CC1=C(C=C(C(=C1[N+](=O)[O-])OC)C(C)(C)C)[N+](=O)[O-] | 1 | validation |
| 144208392 | CNNC.Cl.Cl | 0 | test |
| 144208391 | CC1=CC(=O)CC(C1=O)(C)C | 0 | train |
| 144208390 | CCC(=O)/C=C/C1C(=CCCC1(C)C)C | 0 | train |
| 144208389 | C1=CC=C(C=C1)CN=C=S | 0 | validation |
| 144208388 | C(F)(Cl)(Cl)Cl | 0 | test |
| 144208387 | C1=CC(=C(C(=C1)Cl)Cl)C(=O)O | 0 | train |
| 144208386 | CCC(C)OC(C)CC | 0 | train |
| 144208385 | C(C(Br)(Br)Br)Br | 0 | validation |
| 144208384 | C1C2=CC(=C(C=C2[C@@H]3[C@]1(COC4=C3C=CC(=C4O)O)O)O)O | 0 | test |
| 144208383 | C(CCl)C(=O)O | 0 | train |
| 144208382 | CC(C)(C)OC | 0 | train |
| 144208381 | CC(COC(=O)C1=CC=CC=C1)OC(=O)C2=CC=CC=C2 | 0 | validation |
| 144208380 | CC(C)(C1=CC(=C(C(=C1)Br)O)Br)C2=CC(=C(C(=C2)Br)O)Br | 0 | test |
| 144208379 | C1=CC(=CC=C1C(=O)NCC(=O)O)[N+](=O)[O-] | 0 | train |
| 144208378 | CC(C)C(CCCN(C)CCC1=CC(=C(C=C1)OC)OC)(C#N)C2=CC(=C(C=C2)OC)OC.Cl | 0 | train |
| 144208377 | C1CC(=O)OC1=O | 0 | validation |
| 144208376 | CC([N+](=O)[O-])(Cl)Cl | 0 | test |
| 144208375 | CC(COC(=O)C=C)O | 0 | train |
| 144208374 | CN(C)CCCNCCCN | 0 | train |
| 144208373 | C1CCNCC1 | 1 | validation |
| 144208372 | CCC(C(C)(C)C)O | 0 | test |
| 144208371 | CCCCOC(=O)C1=CC=CC=C1N | 1 | train |
| 144208370 | CCCCN(/C(=N/[N+](=O)[O-])/N)N=O | 0 | train |
| 144208369 | C(CCC(=O)O)CCC(=O)O | 0 | validation |
| 144208368 | CC(C)S | 0 | test |
| 144208367 | C1=CC=C(C(=C1)C(=O)C2=CC=CC=C2O)O | 0 | train |
| 144208366 | C(=S)=S | 0 | train |
| 144208365 | C1=CC=C(C=C1)N=[N+](C2=CC=CC=C2)[O-] | 1 | validation |
| 144208364 | CC1=NC(=NC(=N1)N)N | 0 | test |
| 144208363 | C1=CC(=CC=C1C2=C(C(=O)C3=C(C=C(C=C3O2)O)O)O)O | 1 | train |
| 144208362 | C([C@H]([C@H]([C@@H]([C@@H](CBr)O)O)O)O)Br | 0 | train |
| 144208361 | C1=CC(=CC=C1CC2=CC=C(C=C2)O)O | 1 | validation |
| 144208360 | COC1=CC=C(C=C1)N=[N+](C2=CC=C(C=C2)OC)[O-] | 1 | test |
| 144208359 | [NH4+].[NH4+].[O-]S(=O)(=O)OOS(=O)(=O)[O-] | 0 | train |
| 144208358 | CC1=C(N=CN1)CSCCNC(=NC)NC#N | 0 | train |
| 144208357 | C[C@H]1[C@H]([C@H](C[C@@H](O1)O[C@H]2C[C@@](CC3=C(C4=C(C(=C23)O)C(=O)C5=C(<br>C4=O)C=CC=C5OC)O)(C(=O)C)O)N)O.Cl | 1 | validation |
| 144208356 | C[C@H]1C[C@@H](C(=O)[C@@H](C1)[C@@H](CC2CC(=O)NC(=O)C2)O)C | 0 | test |
| 144208355 | CC(C)CC(C)N | 0 | train |
| 144208354 | CC(C)C(C1=CC=C(C=C1)OC(F)(F)F)(C2=CN=CN=C2)O | 0 | train |
| 144208353 | C/C=C/CC=C | 0 | validation |
| 144208352 | CCC(C)(C)OC | 0 | test |
| 144208351 | C[C@H]1CCCC(=O)CCC/C=C/C2=CC(=CC(=C2C(=O)O1)O)O | 0 | train |
| 144208350 | C1CCC2C(C1)O2 | 1 | train |
| 144208349 | C(=C/C#N)¥C#N | 1 | validation |

| | | | |
|---|---|---|---|
| 144208348 | C1=CC(=C(C=C1C2=C(C(=O)C3=C(C=C(C=C3O2)O)O)O)O)O.O.O | 1 | test |
| 144208347 | CC1=CC(=NO1)N | 0 | train |
| 144208346 | CC1([C@@H](N2[C@H](S1)[C@@H](C2=O)NC(=O)[C@@H](C3=CC=CC=C3)N)C(=O)O)C.O.O.O | 0 | train |
| 144208345 | C1=CC=C(C=C1)COC2=CC=C(C=C2)O | 1 | validation |
| 144208344 | CC1=CC2=C(C=CC=N2)C=C1 | 0 | test |
| 144208343 | CC(C)OC(=O)CC1=CC=CC=C1 | 0 | train |
| 144208342 | C=CC1CC2CC1C=C2 | 0 | train |
| 144208341 | C1=CC(=C(C=C1Br)Br)OC2=CC(=C(C=C2Br)Br)Br | 1 | validation |
| 144208340 | C1=CC2=C(C=C1S(=O)(=O)O)C(=C(C=C2[N+](=O)[O-])[N+](=O)[O-])O | 0 | test |
| 144208339 | C(C(Cl)(Cl)Cl)(Cl)Cl | 0 | train |
| 144208338 | C(CCl)CCl | 0 | train |
| 144208337 | CC1CC(CCC1N)CC2CCC(C(C2)C)N | 0 | validation |
| 144208336 | CCOC(=O)C(C)C(=O)C | 0 | test |
| 144208335 | CC1=CC(=O)CC(C1(/C=C/C(=C¥C(=O)O)/C)O)(C)C | 0 | train |
| 144208334 | COC1=CC=CC=C1O | 0 | train |
| 144208333 | CCC#N | 0 | validation |
| 144208332 | CN(C)CCN(CC1=CC=CS1)C2=CC=CC=N2.Cl | 0 | test |
| 144208331 | C(O)S(=O)[O-].[Na+] | 0 | train |
| 144208330 | CC1(CCC(CC1)C(C)(C)N)N | 0 | train |
| 144208329 | CC(=O)NC1=CC=C(C=C1)OC | 0 | validation |
| 144208328 | COC(=O)CS | 0 | test |
| 144208327 | C=CCOC(=O)C1=CC=CC=C1N | 1 | train |
| 144208326 | C1=CC=C(C=C1)N=C=S | 1 | train |
| 144208325 | CC(C)CC(C)(C)C | 1 | validation |
| 144208324 | CN(/C(=N/[N+](=O)[O-])/N)N=O | 0 | test |
| 144208323 | CC(CO)(CO)CO | 0 | train |
| 144208322 | C=CCCC#N | 1 | train |
| 144208321 | CCCCCCC1=CC=C(C=C1)C2=CC=C(C=C2)C#N | 1 | validation |
| 144208320 | C1=CC2=C(C=CC(=C2)C(=O)O)C=C1C(=O)O | 0 | test |
| 144208319 | C1C[C@@H](O[C@@H]1CO)N2C=NC3=C2N=CN=C3N | 0 | train |
| 144208318 | C1=CC2=C(C=CC(=C2C(=C1)O)O)O | 1 | train |
| 144208317 | CCCC(=O)CC | 0 | validation |
| 144208316 | CC1=CC(=C(C=C1C)N)C | 1 | test |
| 144208315 | CC(C)(C)OC(=O)C=C | 0 | train |
| 144208314 | COC1=C(C=CC(=C1)/C=C/C(=O)CC(=O)/C=C/C2=CC(=C(C=C2)O)OC)O | 1 | train |
| 144208313 | CC1=CC=C(C=C1)O | 0 | validation |
| 144208312 | C(C(=O)O)Cl | 0 | test |
| 144208311 | CC1=CC=C(C=C1)C | 0 | train |
| 144208310 | CC(C(=O)[O-])(Cl)Cl.[Na+] | 0 | train |
| 144208309 | C([C@H]([C@@H]1C(=C(C(=O)O1)O)O)O)O | 0 | validation |
| 144208308 | COC1=CC2=C(C=CN=C2C=C1)[C@@H]([C@H]3CC4CCN3C[C@@H]4C=C)O | 0 | test |
| 144208307 | C(C(Cl)(Cl)Cl)Cl | 0 | train |
| 144208306 | CC(C)CC(=O)C | 0 | train |
| 144208305 | CCCCCO | 1 | validation |
| 144208304 | C([C@@H]1[C@H]([C@@H]([C@H]([C@H](O1)O[C@@H]2[C@@H]([C@H]([C@@H]([C@H](O2)CO)O)O)O)O)O)O)O.O.O | 0 | test |
| 144208303 | CCCCN1C=C[N+](=C1)C.OS(=O)(=O)[O-] | 1 | train |
| 144208302 | C1=C(NC(=O)NC1=O)C(=O)O | 0 | train |
| 144208301 | CCN1CCOCC1 | 0 | validation |
| 144208300 | CCC(=C)C(=O)C1=C(C(=C(C=C1)OCC(=O)O)Cl)Cl | 1 | test |
| 144208299 | C(C(=C(CO)Br)Br)O | 1 | train |
| 144208298 | CN1C=C[N+](=C1)C.COP(=O)([O-])OC | 0 | train |
| 144208297 | CC1=CC(=CC(=C1)OP(=O)(OC2=CC(=CC(=C2)C)C)OC3=CC(=CC(=C3)C)C)C | 1 | validation |
| 144208296 | C1=CC2=C(C(=C1)O)C(=O)C3=C(C=C(C=C3C2=O)C(=O)O)O | 0 | test |
| 144208295 | CC(=CC(=O)C=C(C)C)C | 0 | train |
| 144208294 | CCCCNCC | 0 | train |
| 144208293 | C(O)S(=O)(=O)[O-].[Na+] | 0 | validation |
| 144208292 | C1=CC(=CC(=C1)[N+](=O)[O-])C(=O)Cl | 0 | test |
| 144208291 | CC1=CC(=C(C(=C1)C(C)(C)C)O)C(C)(C)C | 1 | train |
| 144208290 | CCC(C)O | 0 | train |
| 144208289 | CC1=CC=C(C=C1)C(=O)O | 0 | validation |

| | | | |
|---|---|---|---|
| 144208288 | CC(=O)C(Cl)Cl | 0 | test |
| 144208287 | C1=CC=C(C(=C1)CCl)Cl | 0 | train |
| 144208286 | C=CC(=O)OCCOCCOC(=O)C=C | 1 | train |
| 144208285 | C1CCCCC1 | 0 | validation |
| 144208284 | CCCC(=O)OC(=O)CCC | 0 | test |
| 144208283 | C1=CC(=CC(=C1)I)[N+](=O)[O-] | 0 | train |
| 144208282 | C1=CC=C(C(=C1)C(=O)O)O.C(CO)N(CCO)CCO | 0 | train |
| 144208281 | CCCC[N+]1(CCCC1)C.C(F)(F)(F)S(=O)(=O)[N-]S(=O)(=O)C(F)(F)F | 0 | validation |
| 144208280 | C1=C(OC(=C1)C(=O)O)CO | 0 | test |
| 144208279 | CC1=CC=C(C=C1)OC | 0 | train |
| 144208278 | CC(=O)OC(C1=CC=CC=C1)C(=O)C2=CC=CC=C2 | 1 | train |
| 144208277 | CC1=CC2=C(C=C1C)SC(=N2)N | 1 | validation |
| 144208276 | CC(=O)N[C@@H](CS)C(=O)O | 0 | test |
| 144208275 | CC1=CC(NC2=CC=CC=C12)(C)C | 0 | train |
| 144208274 | CC1=C(C(CCC1)(C)C)/C=C/C(=C/C=C/C(=C¥C=O)/C)/C | 1 | train |
| 144208273 | C1COC(=O)O1 | 0 | validation |
| 144208272 | C[NH+](C)CCC(C1=CC=CC=C1)C2=CC=CC=[NH+]2.C(=C¥C(=O)[O-])¥C(=O)[O-] | 0 | test |
| 144208271 | CS(=O)(=O)O | 0 | train |
| 144208270 | C1=CC=C(C=C1)COC(=O)Cl | 0 | train |
| 144208269 | CCC=O | 0 | validation |
| 144208268 | C(=O)(C(Cl)(Cl)Cl)Cl | 0 | test |
| 144208267 | CC1=C(C=C(C=C1)C(=O)O)[N+](=O)[O-] | 0 | train |
| 144208266 | CCCCCCCC(=O)OC1=C(C=C(C=C1Br)C#N)Br | 0 | train |
| 144208265 | CCCCCCCCS(=O)(=O)[O-].[Na+] | 0 | validation |
| 144208264 | C(CNCCNCCN)N | 0 | test |
| 144208263 | CC(C)(C)N(CCO)CCO | 0 | train |
| 144208262 | CCC1=CC=CC=C1 | 0 | train |
| 144208261 | C1=C(C=C(C(=C1Cl)N)Cl)Cl | 0 | validation |
| 144208260 | CCC(C)C1=CC(=C(C(=C1)C(C)(C)C)O)C(C)(C)C | 1 | test |
| 144208259 | CCN1C=C[N+](=C1)C.C(F)(F)(F)S(=O)(=O)[N-]S(=O)(=O)C(F)(F)F | 0 | train |
| 144208258 | CN=C=O | 0 | train |
| 144208257 | C1CC(CC=C1)C=O | 0 | validation |
| 144208256 | C(#N)C(Cl)(Cl)Cl | 0 | test |
| 144208255 | C1C2=CC=CC3=C2C4=C(C=CC=C41)C=C3 | 1 | train |
| 144208254 | C([C@H](C([C@H](CO)O)O)O)O | 0 | train |
| 144208253 | C1=CC(=C(C=C1/C=C/C(=O)O[C@@H](C(=O)O)[C@@H](OC(=O)/C=C/C2=CC(=C(C=C2)O)O)C(=O)O)O)O | 0 | validation |
| 144208252 | C1=CC=C(C=C1)F | 0 | test |
| 144208251 | CCOC(=O)CCl | 0 | train |
| 144208250 | CCCCOC(=O)C | 0 | train |
| 144208249 | CCOC(=O)CC(C(=O)OCC)[O-].[Na+] | 0 | validation |
| 144208248 | C1=CC=C(C=C1)COP(=O)(O)OCC2=CC=CC=C2 | 0 | test |
| 144208247 | C1=CC(=CC=C1NC(=O)N)[As](=O)(O)O | 0 | train |
| 144208246 | CC1=CN=CN1 | 0 | train |
| 144208245 | C1=CC(=C(C=C1Cl)N)O | 1 | validation |
| 144208244 | C1(=C(C1(Cl)Cl)Cl)Cl | 0 | test |
| 144208243 | [O-]S(=O)(=O)[O-].[Na+].[Na+] | 0 | train |
| 144208242 | C1=CC(=C(C=C1S(=O)(=O)O)N)O | 0 | train |
| 144208241 | C[C@H]1CC[C@@H]([C@H](C1)O)C(C)C | 0 | validation |
| 144208240 | CCCCCCCCCCCCCCC1CO1 | 0 | test |
| 144208239 | CCOC1=CC(=C(C=C1C(=O)C)OCC)OCC | 1 | train |
| 144208238 | CCCCCCCCCOC(=O)C1=CC=CC=C1C(=O)OCCCCCCCCC | 1 | train |
| 144208237 | CCN1C=C[N+](=C1)C.C(F)(F)(F)S(=O)(=O)[O-] | 1 | validation |
| 144208236 | C1=CC=C(C=C1)SCCSC2=CC=CC=C2 | 1 | test |
| 144208235 | CCCCOCCCC | 0 | train |
| 144208234 | C(CBr)O | 0 | train |
| 144208233 | CC1=NN=C(S1)NS(=O)(=O)C2=CC=C(C=C2)N | 0 | validation |
| 144208232 | CCOC(=O)Cl | 0 | test |
| 144208231 | COC1=CC(=O)OC(C1)CCC2=CC=CC=C2 | 1 | train |
| 144208230 | C1=CC2=C(C=C1O)C(=CN2)C[C@@H](C(=O)O)N | 0 | train |
| 144208229 | CCCCCCCCCCC(=O)O | 0 | validation |
| 144208228 | C1=CC(=CC=C1N)O | 1 | test |

| | | | |
|---|---|---|---|
| 144208227 | CCC(=O)OC(=O)CC | 0 | train |
| 144208226 | CCCCCCCC[N+](CCCCCCCC)(CCCCCCCC)CCCCCCCC.[Br-] | 1 | train |
| 144208225 | C1=CC(=CC(=C1)S(=O)(=O)O)N | 0 | validation |
| 144208224 | C1CCOC1 | 0 | test |
| 144208223 | CC1=CC(=C(C=C1)C)N | 0 | train |
| 144208222 | CCN1C=C[N+](=C1)C.C(=[N-])=NC#N | 0 | train |
| 144208221 | C1=CNC(=O)NC1=O | 0 | validation |
| 144208220 | CC1=CC(=C(C=C1SC2=CC(=C(C=C2C)O)C(C)(C)C)C(C)(C)C)O | 1 | test |
| 144208219 | CC(C)(C#CC(C)(C)O)O | 0 | train |
| 144208218 | C1=CC=C(C(=C1)C(=O)O)[N+](=O)[O-] | 0 | train |
| 144208217 | CCCCCC(=O)N | 0 | validation |
| 144208216 | C1=CC(=C(C=C1CCN)O)O.Cl | 0 | test |
| 144208215 | C1CCC(CC1)(C2=CC=C(C=C2)O)C3=CC=C(C=C3)O | 1 | train |
| 144208214 | C1=C(C=C(C(=C1S(=O)(=O)O)O)C(=O)O)N | 0 | train |
| 144208213 | C1/C=C¥CC/C=C¥C1 | 0 | validation |
| 144208212 | C1=CC(=C(C=C1[N+](=O)[O-])OCCO)NCCO | 0 | test |
| 144208211 | CC/C=C/C/C=C/C/C=C/CCCCCCCC(=O)OCC | 0 | train |
| 144208210 | COC(=O)C1=CC(=CC=C1)Cl | 0 | train |
| 144208209 | CC[C@@H]1[C@@]([C@@H]([C@H](N(C[C@@H](C[C@@]([C@@H]([C@H]([C@@H]([C@H](C(=O)O1)C)O[C@H]2C[C@@]([C@H]([C@@H](O2)C)O)(C)OC)C)O[C@H]3[C@@H]([C@H](C[C@H](O3)C)N(C)C)O)(C)O)C)C)C)O)(C)O | 0 | validation |
| 144208208 | CN(C)NN=C1C(=NC=N1)C(=O)N | 0 | test |
| 144208207 | C1=CC=C(C=C1)CC2=CC=C(C=C2)O | 1 | train |
| 144208206 | CCNC1=C(C=CC(=C1)O)C | 1 | train |
| 144208205 | C1=CC=C2C(=C1)C(=O)[N-]C2=O.[K+] | 0 | validation |
| 144208204 | C([C@@H]1[C@@H]2[C@@H]([C@H]([C@H](O1)O[C@@H]3[C@H](O[C@@H]([C@@H]([C@H]3O)O)O[C@@H]4[C@H](O[C@@H]([C@@H]([C@H]4O)O)O[C@@H]5[C@H](O[C@@H]([C@@H]([C@H]5O)O)O[C@@H]6[C@H](O[C@@H]([C@@H]([C@H]6O)O)O[C@@H]7[C@H](O[C@@H]([C@@H]([C@H]7O)O)O[C@@H]8[C@H](O[C@H](O2)[C@@H]([C@H]8O)O)CO)CO)CO)CO)CO)CO)O)O)O | 0 | test |
| 144208203 | CC1=C(C(CCC1)(C)C)/C=C/C(=C/C=C/C(=C/C=O)/C)/C | 1 | train |
| 144208202 | CCCCCCCN | 0 | train |
| 144208201 | CCOC1=CC=CC=C1N | 1 | validation |
| 144208200 | CC(C)C(=O)OC(=O)C(C)C | 0 | test |
| 144208199 | CC(=O)[O-].[Tl+] | 0 | train |
| 144208198 | COC(=O)C=C | 0 | train |
| 144208197 | C1=CC=C(C(=C1)C#N)C#N | 0 | validation |
| 144208196 | CC(CCl)O | 0 | test |
| 144208195 | CCOC1=CC=CC(=C1)N | 0 | train |
| 144208194 | CNCCS(=O)(=O)[O-].[Na+] | 0 | train |
| 144208193 | C1=CC=C(C=C1)C2=NC3=C(N2)C=C(C=C3)S(=O)(=O)O | 0 | validation |
| 144208192 | C1=NC2C(N1)C(=S)NC=N2.O | 1 | test |
| 144208191 | COC1=CC(=C(C=C1)C(=O)C2=CC=CC=C2O)O | 1 | train |
| 144208190 | C1=CC(=C(C=C1N)N)[N+](=O)[O-] | 0 | train |
| 144208189 | C(#N)/C(=C(¥C#N)/N)/N | 0 | validation |
| 144208188 | COC(=O)C1=CC=C(C=C1)C(=O)O | 0 | test |
| 144208187 | CC1=C(C(CCC1)(C)C)/C=C/C(=C/C=C/C(=C/C(=O)NC2=CC=C(C=C2)O)/C)/C | 1 | train |
| 144208186 | C1=CC=C(C=C1)C(=O)OCC(COC(=O)C2=CC=CC=C2)(COC(=O)C3=CC=CC=C3)COC(=O)C4=CC=CC=C4 | 0 | train |
| 144208185 | C[C@]12CCC(=O)C[C@@H]1CC[C@@H]3[C@@H]2CC[C@]4([C@H]3CC[C@@H]4O)C | 0 | validation |
| 144208184 | C1=CC2=C(C(=C1)O)C(=O)C3=C(C2=O)C=CC=C3O | 1 | test |
| 144208183 | C1=CC(=CC=C1N)OC2=CC=C(C=C2)Cl | 1 | train |
| 144208182 | C=CC=O | 0 | train |
| 144208181 | CC(C)CCON=O | 0 | validation |
| 144208180 | CN(C)CCCCCCN(C)C | 0 | test |
| 144208179 | C1=CC=C2C=C(C=CC2=C1)Cl | 0 | train |
| 144208178 | C1=CC=C(C(=C1)N)N.Cl.Cl | 1 | train |
| 144208177 | C1=CC(=C(C(=C1)C(=O)O)C(=O)O)C(=O)O | 0 | validation |
| 144208176 | CN(C)CCN(CC1=CC=C(C=C1)OC)C2=CC=CC=N2 | 0 | test |
| 144208175 | CCOP(=S)(OCC)Cl | 0 | train |
| 144208174 | C#CCO | 1 | train |
| 144208173 | C1=CC=C(C(=C1)CC#N)[N+](=O)[O-] | 0 | validation |

| | | | |
|---|---|---|---|
| 144208172 | C1=CC(=CC=C1C(=O)C2=CC=C(C=C2)Cl)O | 1 | test |
| 144208171 | C1=CC(=CC=C1O)SC2=CC=C(C=C2)O | 1 | train |
| 144208170 | C1=C2C(=CC(=C1Cl)S(=O)(=O)N)S(=O)(=O)N=CN2 | 0 | train |
| 144208169 | C1COCCO1 | 0 | validation |
| 144208168 | C1CC(C(CC1C(CBr)Br)Br)Br | 1 | test |
| 144208167 | CC1=CC=C(C=C1)Br | 0 | train |
| 144208166 | CN1C(=C(C(=O)N(C1=O)C)N=O)N | 1 | train |
| 144208165 | C1=NC(=NN1[C@H]2[C@@H]([C@@H]([C@H](O2)CO)O)O)C(=O)N | 0 | validation |
| 144208164 | C1=C(C=C(C(=C1[N+](=O)[O-])Cl)[N+](=O)[O-])C(F)(F)F | 0 | test |
| 144208163 | C/C(=C/CCl)/Cl | 0 | train |
| 144208162 | CCCCOCCOCCOCCCC | 1 | train |
| 144208161 | CC(=O)O[C@H]1CC[C@@]2([C@H]3CC[C@]4([C@H]([C@@H]3CC=C2C1)CCC4=O)C)C | 0 | validation |
| 144208160 | CNCC(C1=CC=C(C(=C1)O)O)O | 0 | test |
| 144208159 | C1=CC(=C(C=C1Cl)SC2=C(C=CC(=C2)Cl)O)O | 0 | train |
| 144208158 | C1=CC=NC=C1 | 0 | train |
| 144208157 | C1=CC=C(C=C1)C2=CC=CC=C2N | 0 | validation |
| 144208156 | CC1=CC(=CC=C1)OC2=CC=CC=C2 | 0 | test |
| 144208155 | C(C#N)Cl | 0 | train |
| 144208154 | C1=CC=C(C(=C1)N)S(=O)(=O)O | 0 | train |
| 144208153 | CC(OC)(OC)OC | 0 | validation |
| 144208152 | C1=CC=C(C=C1)C(F)(F)F | 0 | test |
| 144208151 | C1=CC2=C(C=CC=C2[N+](=O)[O-])C(=C1)[N+](=O)[O-] | 1 | train |
| 144208150 | COC1=C(C=CC(=C1)Cl)Cl | 0 | train |
| 144208149 | C(CSSCCC(=O)O)C(=O)O | 0 | validation |
| 144208148 | C(=O)(N)NN.Cl | 0 | test |
| 144208147 | CC(C)(CCl)C1=CC=CC=C1 | 0 | train |
| 144208146 | C1=CC(=CC(=C1)Br)C=O | 0 | train |
| 144208145 | CC1=CC=C(C=C1)C#N | 0 | validation |
| 144208144 | C1=CC(=C(C=C1Cl)Cl)C(F)(F)F | 1 | test |
| 144208143 | C[C@H]1[C@@H]([C@H]([C@H]([C@@H](O1)O[C@@H]2[C@H]([C@@H](O[C@@H]([C@H]2OC(=O)/C=C/C3=CC(=C(C=C3)O)O)CO[C@H]4[C@@H]([C@H]([C@@H]([C@H](O4)CO)O)O)O)OCCC5=CC(=C(C=C5)O)O)O)O)O)O | 0 | train |
| 144208142 | C1=C(OC(=C1)C(=O)NCC(=O)O)CO | 0 | train |
| 144208141 | CC/C(=C(¥C1=CC=C(C=C1)O)/C2=CC=C(C=C2)OCCN(C)C)/C3=CC=CC=C3 | 1 | validation |
| 144208140 | C1OCOCO1 | 0 | test |
| 144208139 | CC(C)CCCCOC(=O)C1=CC=CC=C1C(=O)OCCCCC(C)C | 1 | train |
| 144208138 | C1=CC2=C(C(=C1)N)C(=O)NNC2=O | 0 | train |
| 144208137 | C1=CC(=C(C=C1Cl)Cl)C(Cl)(Cl)Cl | 0 | validation |
| 144208136 | COC(=O)CC#N | 0 | test |
| 144208135 | C1=C(C(=CC(=C1Cl)Cl)Cl)[N+](=O)[O-] | 0 | train |
| 144208134 | C([C@H]([C@H]([C@@H]([C@H]([C@H](C(=O)[O-])O)O)O)O)O)O.[Na+] | 0 | train |
| 144208133 | CCCC(C)C(=O)O | 0 | validation |
| 144208131 | CC1=CC(=C(C=C1)O)[N+](=O)[O-] | 1 | train |
| 144208130 | C1=CC=C(C(=C1)N)O | 1 | train |
| 144208129 | C1=C(C=C(C=C1Cl)Cl)O | 0 | validation |
| 144208128 | CC1=CC=C(C=C1)C(=O)C2=C(C=C(C=C2)OC)O | 1 | test |
| 144208127 | C[C@@H]1CC2(C3C(O3)(C(O2)O)C)OC4C1[C@]5(CC[C@@]67C[C@@]68CCC(C([C@@H]8CC=C7[C@@]5(C4)C)(C)C)O[C@H]9[C@@H]([C@H]([C@@H](CO9)O)O)O)C | 0 | train |
| 144208126 | C(O)[P+](CO)(CO)CO.C(O)[P+](CO)(CO)CO.[O-]S(=O)(=O)[O-] | 1 | train |
| 144208125 | CCSCC | 1 | validation |
| 144208124 | CCCCC(CC)COC(=O)COC1=C(C=C(C=C1)Cl)Cl | 0 | test |
| 144208123 | CC(=O)NC1=CC=CC=C1 | 0 | train |
| 144208122 | C1C(=O)NC(=O)NC1=O | 1 | train |
| 144208121 | C(=O)(C(Cl)(Cl)Br)O | 0 | validation |
| 144208120 | C1=C2C(=NC=NC2=O)NN1 | 0 | test |
| 144208119 | CC1=C(C=CC=C1O)C(=O)N[C@@H](CSC2=CC=CC=C2)[C@@H](CN3C[C@H]4CCCC[C@H]4C[C@H]3C(=O)NC(C)(C)C)O.CS(=O)(=O)O | 1 | train |
| 144208118 | C1=COC=C1 | 0 | train |
| 144208117 | CCN(CC)C=O | 1 | validation |
| 144208116 | C(CC(=O)O)C(=O)C(=O)O | 0 | test |
| 144208115 | CC1=CC=CC=C1N=O | 0 | train |
| 144208114 | CC(CC(C)O)O | 0 | train |

| | | | |
|---|---|---|---|
| 144208113 | C1=CC=C(C(=C1)C(=O)O)C(=O)O | 0 | validation |
| 144208112 | C([C@@H]1[C@H]([C@@H]([C@H]([C@H](O1)OC[C@H]([C@H]([C@@H]([C@@H](CO)O)O)O)O)O)O)O)O | 0 | test |
| 144208111 | C(=O)(C(Br)(Br)Br)O | 0 | train |
| 144208110 | C=C1C(C2(C(C(C1(C2(Cl)Cl)Cl)Cl)Cl)Cl)(CCl)CCl | 1 | train |
| 144208109 | CCSCCCl | 0 | validation |
| 144208108 | C1=C(C(=C(C(=C1Cl)Cl)O)Cl)Cl | 0 | test |
| 144208107 | C1=CC(=C(C(=C1)Cl)Cl)O | 0 | train |
| 144208106 | CCCCCCCCOC(=O)C1=CC=C(C=C1)O | 1 | train |
| 144208105 | COC1=CC(=O)O[C@H](C1)/C=C/C2=CC=CC=C2 | 1 | validation |
| 144208104 | CNC1=CC=C(C=C1)O.CNC1=CC=C(C=C1)O.OS(=O)(=O)O | 1 | test |
| 144208103 | C(C(F)(F)F)O | 0 | train |
| 144208102 | CCCCCCCCOC1=CC=C(C=C1)C(=O)O | 1 | train |
| 144208101 | C1=CC=C(C=C1)COCC2=CC=CC=C2 | 0 | validation |
| 144208100 | C1=CC(=CC(=C1)S(=O)(=O)[O-])[N+](=O)[O-].[Na+] | 0 | test |
| 144208099 | COC1=CC(=O)O[C@H](C1)/C=C/C2=CC3=C(C=C2)OCO3 | 1 | train |
| 144208098 | C1=C(N=C(S1)N)CC(=O)O | 0 | train |
| 144208097 | C([N+](=O)[O-])(Br)Br | 0 | validation |
| 144208096 | C=CCNC(=S)N | 0 | test |
| 144208095 | C1=CC=C(C=C1)C2=CSC(=N2)NN | 1 | train |
| 144208094 | C[C@H](CCC(=O)[O-])[C@H]1CC[C@@H]2[C@@]1([C@H](C[C@H]3[C@H]2CC[C@H]4[C@@]3(CC[C@H](C4)O)C)O)C.[Na+] | 0 | train |
| 144208093 | CC(C)(C)NSC1=NC2=CC=CC=C2S1 | 1 | validation |
| 144208092 | C(C(=O)O)(Cl)Br | 0 | test |
| 144208091 | C1=C(C(=C(C(=C1Cl)Cl)Cl)Cl)[N+](=O)[O-] | 0 | train |
| 144208090 | C1=CC(=CC=C1C(=O)C2=C(C(=C(C=C2)O)O)O)O | 1 | train |
| 144208089 | CC(C)CC=O | 0 | validation |
| 144208088 | CC(C)CC(C)NC1=CC=C(C=C1)NC2=CC=CC=C2 | 1 | test |
| 144208087 | CCOC(=O)C(C)O | 0 | train |
| 144208086 | CCN(CC)C1=CC=C(C=C1)N | 1 | train |
| 144208085 | CC1=CC(=CC=C1)NC(=O)C | 0 | validation |
| 144208084 | C(=N[N+](=O)[O-])(N)N | 0 | test |
| 144208083 | CC1=CC=CC=C1OCC2CO2 | 0 | train |
| 144208082 | CON=C(C1=CC=CO1)C(=O)N[C@H]2[C@@H]3N(C2=O)C(=C(CS3)COC(=O)N)C(=O)O | 0 | train |
| 144208081 | CCC(C)COC(=O)C | 0 | validation |
| 144208080 | CC1=CC(=O)OC2=CC(=CC(=C12)O)O | 1 | test |
| 144208079 | CC(=O)NC1=CC=CC(=C1)N | 0 | train |
| 144208078 | C1=CC=NC(=C1)N | 0 | train |
| 144208077 | COC(=O)NN | 0 | validation |
| 144208076 | C1=CC=C(C=C1)C(=O)NN | 1 | test |
| 144208075 | CC1CCCNC1 | 0 | train |
| 144208074 | CC1=CC(=C(C=C1)O)N2N=C3C=CC=CC3=N2 | 1 | train |
| 144208073 | CC1=C(C=C(C=C1)[N+](=O)[O-])Cl | 0 | validation |
| 144208072 | CC(C)(C)S | 0 | test |
| 144208071 | CCN(CC)CC | 0 | train |
| 144208070 | C1CCC(CC1)C2=CC(=CC(=C2O)[N+](=O)[O-])[N+](=O)[O-] | 0 | train |
| 144208069 | C=CC1CO1 | 0 | validation |
| 144208068 | C1=CC(=CC=C1C(CN)O)O.Cl | 0 | test |
| 144208067 | CCCCC(=O)N(C)C | 0 | train |
| 144208066 | C[C@@H]1[C@H]2C[C@]2(CC1=O)C(C)C | 0 | train |
| 144208065 | CN1[C@@H]2CC(C[C@H]1[C@H]3[C@@H]2O3)OC(=O)[C@H](CO)C4=CC=CC=C4.O.O.O.Br | 0 | validation |
| 144208064 | CN(C)C1=CC=C(C=C1)C2(C3=C(C=C(C=C3)N(C)C)C(=O)O2)C4=CC=C(C=C4)N(C)C | 0 | test |
| 144208063 | CCCCCl | 0 | train |
| 144208062 | CC(=O)[O-].CC(=O)[O-].O.O.[Zn+2] | 0 | train |
| 144208061 | C(CS(=O)(=O)[O-])S.[Na+] | 0 | validation |
| 144208060 | CCC1(C(=O)NC(=NC1=O)[O-])CC.[Na+] | 0 | test |
| 144208059 | CCCCCC[C@H](C/C=C¥CCCCCCCC(=O)OC)O | 0 | train |
| 144208058 | CCCCCCCCCC#N | 0 | train |
| 144208057 | C1=CC=C2C(=C1)C(=O)N(C2=O)CO | 0 | validation |
| 144208056 | CCCCCCCCCCCCCCCCCC(=O)[O-].C(CO)[NH+](CCO)CCO | 1 | test |
| 144208055 | C(=N)(N)[Se] | 0 | train |

| | | | |
|---|---|---|---|
| 144208054 | CC1=NC(=NC(=N1)OC)NC(=O)NS(=O)(=O)C2=CC=CC=C2C(=O)OC | 0 | train |
| 144208053 | CC(C)OS(=O)(=O)C | 0 | validation |
| 144208052 | C[C@]12CCC(=O)C=C1CC[C@@H]3[C@@]2([C@H](C[C@]4([C@H]3CC[C@]4(C)O)C)O)F | 0 | test |
| 144208051 | CC1=CC=CC(=C1)C#N | 0 | train |
| 144208050 | CC1=CC=CC(=C1[N+](=O)[O-])C(=O)O | 0 | train |
| 144208049 | C1=CC(=C(C(=C1)Cl)Cl)[N+](=O)[O-] | 0 | validation |
| 144208048 | C(C(F)(F)F)(Cl)Br | 0 | test |
| 144208047 | C1=CC=C(C=C1)Br | 0 | train |
| 144208046 | CC1=CC=C(C=C1)/C(=C¥CN2CCCC2)/C3=CC=CC=N3.O.Cl | 0 | train |
| 144208045 | CC(C1=CC=CC(=C1)C(=O)C2=CC=CC=C2)C(=O)O | 0 | validation |
| 144208044 | C1=CC(=CC=C1C2=CSC(=N2)N)[N+](=O)[O-] | 1 | test |
| 144208043 | CCC(CO)(CO)N | 0 | train |
| 144208042 | C=C(CCC#N)C#N | 0 | train |
| 144208041 | CCOCCOCCOC(=O)C | 0 | validation |
| 144208040 | CCCCCCCCN1C=C[N+](=C1)C.C(F)(F)(F)S(=O)(=O)[N-]S(=O)(=O)C(F)(F)F | 0 | test |
| 144208039 | COC(=O)Cl | 0 | train |
| 144208038 | C(OCCl)Cl | 0 | train |
| 144208037 | CC1=CC2=CC=CC=C2C=C1 | 0 | validation |
| 144208036 | CC(C)(C)OO | 0 | test |
| 144208035 | C1=CC(=CC(=C1)[N+](=O)[O-])CCl | 0 | train |
| 144208034 | CCCCC(CC)COCCCN | 0 | train |
| 144208033 | CC1=C(C=CC(=C1)C(=O)O)[N+](=O)[O-] | 0 | validation |
| 144208032 | C/C=C/C=C/C=O | 0 | test |
| 144208031 | C1=CC(=CC(=C1)Cl)O | 0 | train |
| 144208030 | CC1=CC=C(C=C1)NC(=O)N | 0 | train |
| 144208029 | C(C(F)(F)F)(OC(F)F)Cl | 0 | validation |
| 144208028 | CC(=O)NC1=CC2=C(C=C1)C3=CC=CC=C3C2 | 1 | test |
| 144208027 | C1=CC=C(C=C1)C(=O)OC(=O)C2=CC=CC=C2 | 0 | train |
| 144208026 | CCC1CCC(C1)CCC(=O)O | 0 | train |
| 144208025 | C(CSCCO)O | 0 | validation |
| 144208024 | CC(=C)C(=O)O | 0 | test |
| 144208023 | CC[S+](C)CC.C(F)(F)(F)S(=O)(=O)[N-]S(=O)(=O)C(F)(F)F | 0 | train |
| 144208022 | C=CCCl | 0 | train |
| 144208021 | C1=CC=C2C(=C1)C=CC=C2Cl | 0 | validation |
| 144208020 | C[N+](=O)[O-] | 0 | test |
| 144208019 | C1=CC=C(C(=C1)CCl)[N+](=O)[O-] | 0 | train |
| 144208018 | COC1=CC=CC(=C1)N | 0 | train |
| 144208017 | CCCOCC(C)OCC(C)O | 0 | validation |
| 144208016 | C1=CC=C2C(=C1)N=C(S2)N | 1 | test |
| 144208015 | CC(=O)OC=C | 0 | train |
| 144208014 | CS(=O)(=O)C1=CC=C(C=C1)[C@H]([C@@H](CO)NC(=O)C(Cl)Cl)O | 0 | train |
| 144208013 | C[C@]12CCC(=O)C=C1CC[C@@H]3[C@@H]2[C@H](C[C@]4([C@H]3CC[C@@]4(C(=O)CO)O)C)O | 0 | validation |
| 144208012 | O=P(Cl)(Cl)Cl | 0 | test |
| 144208011 | OP(=O)([O-])OP(=O)(O)[O-].[Na+].[Na+] | 0 | train |
| 144208010 | CCCCCCCCCCCCl | 0 | train |
| 144208009 | CCCCS | 0 | validation |
| 144208008 | CCOC(=O)C(=C)C | 0 | test |
| 144208007 | CCCCCCCCCCCCCC[P+](CCCCCC)(CCCCCC)CCCCCC.[Br-] | 1 | train |
| 144208006 | C=CC1=CC=CC=C1 | 0 | train |
| 144208005 | C1=CC(=C(C=C1Cl)[N+](=O)[O-])N | 1 | validation |
| 144208004 | C(Cl)(Cl)Br | 0 | test |
| 144208003 | C1=CC=C(C(=C1)C(=O)N)[N+](=O)[O-] | 0 | train |
| 144208002 | C1=CC=C(C=C1)C(C#N)O | 0 | train |
| 144208001 | CC1=NC2=C(N1)C=C(C=C2)Cl | 1 | validation |
| 144208000 | C1=CC=C(C(=C1)N)Cl | 0 | test |
| 144207999 | C=CCBr | 0 | train |
| 144207998 | CC(C)(C)NCC(C1=CC(=C(C=C1)O)CO)O | 0 | train |
| 144207997 | C[C@]12CC[C@](C[C@H]1C3=CC(=O)[C@@H]4[C@]5(CC[C@@H](C([C@@H]5CC[C@]4([C@@]3(CC2)C)C)(C)C)O)C)(C)C(=O)O | 0 | validation |
| 144207996 | [O-]S(=O)(=O)OOS(=O)(=O)[O-].[K+].[K+] | 0 | test |

| | | | |
|---|---|---|---|
| 144207995 | CC1=CC(=C(C(=C1CN2C(=O)N(C(=O)N(C2=O)CC3=C(C(=C(C=C3C)C(C)(C)C)O)C)CC4=C(C(=C(C=C4C)C(C)(C)C)O)C)C)O)C(C)(C)C | 0 | train |
| 144207994 | C(/C=C¥CCl)Cl | 0 | train |
| 144207993 | CC(=CC(C)(C)C)C | 0 | validation |
| 144207992 | CNC(=O)NC | 0 | test |
| 144207991 | C1=CN=CC=C1N | 0 | train |
| 144207990 | CC(C)N(C)C | 0 | train |
| 144207989 | C1=CC=C(C(=C1)[N+](=O)[O-])Cl | 0 | validation |
| 144207988 | C(Cl)Br | 0 | test |
| 144207987 | C1=CC2=C(C=CC(=C2)SSC3=CC4=C(C=C3)C=C(C=C4)O)C=C1O | 1 | train |
| 144207986 | C1=CC=C2C(=C1)C(=C3C=CC=CC3=N2)N.O.Cl | 1 | train |
| 144207985 | CCCCCCCCCCCCOCC1CO1 | 0 | validation |
| 144207984 | CNC(=O)N(C1=CC=CC=C1)C2=CC=CC=C2 | 1 | test |
| 144207983 | CCCCC(CC)CNCC(CC)CCCC | 0 | train |
| 144207982 | O=[Cr]O[Cr]=O | 0 | train |
| 144207981 | C1CNC(=O)N1 | 0 | validation |
| 144207980 | CC(CO)OC1=CC=CC=C1 | 0 | test |
| 144207979 | CCCCOCC(C)OCC(C)O | 0 | train |
| 144207978 | CO[Si](OC)(OC)OC | 0 | train |
| 144207977 | CCCCOC(=O)/C=C¥C(=O)OCCCC | 0 | validation |
| 144207976 | C(C(=O)Cl)(Cl)Cl | 0 | test |
| 144207975 | CCCCCC | 0 | train |
| 144207974 | CC1=CC(=CC(=C1)[N+](=O)[O-])C | 0 | train |
| 144207973 | C1=CC(=C(C(=C1)Cl)O)Cl | 0 | validation |
| 144207972 | C(=CCl)Cl | 0 | test |
| 144207971 | CC1=C(C=CC=C1[N+](=O)[O-])N | 0 | train |
| 144207970 | C1=CC=C(C=C1)/C=C/[N+](=O)[O-] | 1 | train |
| 144207969 | C1=CC(=CC=C1COC(CN2C=CN=C2)C3=C(C=C(C=C3)Cl)Cl)Cl.[N+](=O)(O)[O-] | 1 | validation |
| 144207968 | CC1=C2C(=NC=C1)N(C3=C(C=CC=N3)C(=O)N2)C4CC4 | 0 | test |
| 144207967 | CCOC(=O)OCC | 0 | train |
| 144207966 | CCCCCCCCN1C=C[N+](=C1)C.F[P-](F)(F)(F)(F)F | 0 | train |
| 144207965 | C(=S)(C(=S)N)N | 0 | validation |
| 144207964 | C1=CC(=CC(=C1)CN)CN | 0 | test |
| 144207963 | CCCC[P+](CCCC)(CCCC)CCCC.[Cl-] | 0 | train |
| 144207962 | CCOC(=O)/C=C/C(=O)OCC | 0 | train |
| 144207961 | CCN(CC)CCOC(=O)C(=C)C | 0 | validation |
| 144207960 | C([C@@H]1[C@H]([C@@H]([C@](O1)(CO)O)O)O)O | 0 | test |
| 144207959 | CC(C(=O)O)OC1=CC(=C(C=C1Cl)Cl)Cl | 0 | train |
| 144207958 | C1=CC(=CC(=C1)[N+](=O)[O-])C(F)(F)F | 0 | train |
| 144207957 | C1=CC=C(C=C1)C2=CC=CC=C2[N+](=O)[O-] | 0 | validation |
| 144207956 | C[C@H]1/C=C/C=C(¥C(=O)NC2=C3C(=NC4(N3)CCN(CC4)CC(C)C)C5=C6C(=C(C(=C5C2=O)O)C)O[C@@](C6=O)(O/C=C/[C@@H]([C@H]([C@H]([C@@H]([C@@H]([C@@H]([C@H]1O)C)O)C)OC(=O)C)C)OC)C)/C | 0 | test |
| 144207955 | CN(C)C1=CC=C(C=C1)N=NC2=CC=CC=C2 | 1 | train |
| 144207954 | C1=CC(=C(C=C1Cl)Cl)C(=O)O | 0 | train |
| 144207953 | COC1=C(C=CC(=C1)[C@@H]2[C@H](OC3=C(O2)C=C(C=C3)[C@@H]4[C@H](C(=O)C5=C(C=C(C=C5O4)O)O)O)CO)O | 0 | validation |
| 144207952 | CC(=O)NC1=CC=C(C=C1)N | 0 | test |
| 144207951 | CCCC(C)CO | 0 | train |
| 144207950 | CCCCCCN1C=C[N+](=C1)C.[I-] | 0 | train |
| 144207949 | C1=CC=C(C(=C1)C(F)(F)F)Cl | 0 | validation |
| 144207948 | CCCCCCCCCCCCCC[P+](CCCCCC)(CCCCCC)CCCCCC.F[P-](F)(F)(F)(F)F | 1 | test |
| 144207947 | CCCC(C1=CC(=C(C=C1C)O)C(C)(C)C)C2=CC(=C(C=C2C)O)C(C)(C)C | 1 | train |
| 144207946 | COCCOCCOCCOCCO | 0 | train |
| 144207945 | CC1=CC(=C(C(=C1)[N+](=O)[O-])O)[N+](=O)[O-] | 0 | validation |
| 144207944 | C1=CC(=CC=C1OCCO)OCCO | 0 | test |
| 144207943 | CC(C)CCCCCCOC(=O)C1=CC=CC=C1C(=O)OCCCCCCC(C)C | 0 | train |
| 144207942 | CCN(C)N=O | 0 | train |
| 144207941 | COC1=C(C=C(C=C1)C(=O)NC2=CC=CC=C2)[N+](=O)[O-] | 1 | validation |
| 144207940 | C1=CC(=C(C(=C1)Cl)C=O)Cl | 0 | test |
| 144207939 | CCCCO | 0 | train |
| 144207938 | CCC(C)(C)C(=O)O | 0 | train |

| | | | |
|---|---|---|---|
| 144207937 | C1COC1 | 0 | validation |
| 144207936 | CC(C)NC(=O)C=C | 0 | test |
| 144207935 | CC(=O)C1=CC(=CC=C1)[N+](=O)[O-] | 0 | train |
| 144207934 | CCCCC(CC)CN | 0 | train |
| 144207933 | CCCCCCN1C=C[N+](=C1)C.F[P-](F)(F)(F)(F)F | 0 | validation |
| 144207932 | C1=CC(=CC(=C1)N)N.Cl.Cl | 1 | test |
| 144207931 | CCN(CC)C(=O)C | 0 | train |
| 144207930 | C1=CC2=C(C=CC(=C2)O)C=C1C(=O)O | 0 | train |
| 144207929 | C1=CC=C2C(=C1)C(=O)N(C2=O)CCl | 0 | validation |
| 144207928 | CCC(=O)OC | 0 | test |
| 144207927 | CCOC1=CC=C(C=C1)[N+](=O)[O-] | 0 | train |
| 144207926 | C1=CC=C(C=C1)SSC2=CC=CC=C2 | 0 | train |
| 144207925 | CC(=O)OC(C1=CC=C(O1)[N+](=O)[O-])OC(=O)C | 1 | validation |
| 144207924 | C1=CSC(=N1)N | 0 | test |
| 144207923 | C1=CC(=C2C(=C1[N+](=O)[O-])C(=O)C3=C(C=CC(=C3C2=O)O)[N+](=O)[O-])O | 1 | train |
| 144207922 | C(C(Cl)(Cl)Cl)(Cl)(Cl)Cl | 0 | train |
| 144207921 | CCCCC=C | 0 | validation |
| 144207920 | CC1CCC2=C(C1)OC=C2C | 0 | test |
| 144207919 | C1=CC=C2C=C3C4=CC=CC5=C4C(=CC=C5)C3=CC2=C1 | 1 | train |
| 144207918 | C1=CC=C(C=C1)NC2=CC=CC=C2[N+](=O)[O-] | 1 | train |
| 144207917 | CN(C)CC1=CC=CC=C1 | 0 | validation |
| 144207916 | CCCCCCN1C=C[N+](=C1)C.[Cl-] | 0 | test |
| 144207915 | CCCN1C=C[N+](=C1)C.C(F)(F)(F)S(=O)(=O)[N-]S(=O)(=O)C(F)(F)F | 0 | train |
| 144207914 | C1=CC(=CC=C1C(=O)NC2=CC=C(C=C2)N)N | 0 | train |
| 144207913 | C1=C(C=C(C=C1C(=O)O)S(=O)(=O)[O-])C(=O)O.[Na+] | 0 | validation |
| 144207912 | CCCCCCCCS(=O)(=O)Cl | 0 | test |
| 144207911 | CC(OC)OC | 0 | train |
| 144207910 | CC1=CC=CC=C1NC(=NC2=CC=CC=C2C)N | 0 | train |
| 144207909 | CCCCCCOC(=O)C | 0 | validation |
| 144207908 | C1=CC=C2C3=C4C(=CC2=C1)C=CC5=C4C(=CC=C5)C=C3 | 1 | test |
| 144207907 | C(C(CBr)Br)O | 0 | train |
| 144207906 | CCCCCCCCN1C=C[N+](=C1)C.C(F)(F)(F)S(=O)(=O)[O-] | 0 | train |
| 144207905 | CCCCCC[N+](=O)[O-] | 0 | validation |
| 144207904 | C1=CC(=CC(=C1)O)N | 0 | test |
| 144207903 | C1=CC=C2C(=C1)C(=O)C(C2=O)(O)O | 0 | train |
| 144207902 | C1=C(C=C(C(=C1Cl)Cl)Cl)[N+](=O)[O-] | 0 | train |
| 144207901 | C1=CC=C(C(=C1)C(=O)O)N | 0 | validation |
| 144207900 | CCCCN(CCCC)CCO | 0 | test |
| 144207899 | CCCC[P+](CCCC)(CCCC)CCCC.[Br-] | 0 | train |
| 144207898 | C1=CC=C(C=C1)C2=NC(C(=O)NC3=C2C=C(C=C3)Cl)O | 0 | train |
| 144207897 | C([C@@H](C(=O)O)N)S.Cl | 0 | validation |
| 144207896 | C1CN(C(=O)N1)CCO | 0 | test |
| 144207895 | CCCCCCCCCCCCCC[P+](CCCCCC)(CCCCCC)CCCCCC.[Cl-] | 1 | train |
| 144207894 | CNC.Cl | 0 | train |
| 144207893 | [B-](F)(F)(F)F.CCCCCCCCN1C=C[N+](=C1)C | 0 | validation |
| 144207892 | CC(COC(=O)C(=C)C)O | 0 | test |
| 144207891 | CN1C2=C(C3=C(C=C2)N=CC=C3)N=C1N | 1 | train |
| 144207890 | CC1=CC(=C(C=C1)O)N | 1 | train |
| 144207888 | CCCCC(=O)Cl | 0 | test |
| 144207887 | C[C@]12CC[C@H]3[C@H]([C@@H]1CC[C@@H]2O)CCC4=CC(=O)CC[C@]34C | 0 | train |
| 144207886 | C1=CC(=C(C=C1F)F)[N+](=O)[O-] | 0 | train |
| 144207885 | C1=C(C=C(C(=C1Cl)[N+](=O)[O-])Cl)Cl | 0 | validation |
| 144207884 | CN1CCC2=CC3=C(C=C2[C@@H]1[C@@H]4C5=C(C(=C(C=C5)OC)OC)C(=O)O4)OCO3 | 0 | test |
| 144207883 | C1=CC=C(C=C1)CC(=O)OCC2=CC=CC=C2 | 0 | train |
| 144207882 | CC(=C)C1=CC(=CC=C1)C(=C)C | 0 | train |
| 144207881 | C1=CC2=C(C(=C1)[N+](=O)[O-])N=CC=C2 | 1 | validation |
| 144207880 | C1=CC=C(C=C1)C=CCOC(=O)C2=CC=CC=C2N | 1 | test |
| 144207879 | CN(C)CCN(C)CCN(C)C | 0 | train |
| 144207878 | CCCCOCC(C)O | 0 | train |
| 144207877 | C1=CC(=CC(=C1)N=C=O)C(F)(F)F | 0 | validation |
| 144207876 | CC1=CC(=C(C=C1Cl)S(=O)(=O)O)N | 0 | test |
| 144207875 | CCC(=O)CC | 0 | train |

| | | | |
|---|---|---|---|
| 144207874 | C1(C(C(C(C(C1Cl)Cl)Cl)Cl)Cl)Cl | 0 | train |
| 144207873 | C1=CC(=C(C=C1O)Cl)Cl | 0 | validation |
| 144207872 | CC(C)C(C)C | 0 | test |
| 144207871 | C1=C(C=C(C(=C1Cl)Cl)Cl)Cl | 0 | train |
| 144207870 | CC1=CC2=C(C=C1)C(=CC=C2)C | 0 | train |
| 144207869 | C1=CC(=C(C=C1Cl)Cl)C(=O)CCl | 0 | validation |
| 144207868 | C1=CC(=C(C(=C1[N+](=O)[O-])Cl)Cl)Cl | 0 | test |
| 144207867 | C(CNCCNCCNCCN)N | 0 | train |
| 144207866 | C1C(O1)COC2=CC(=CC=C2)OCC3CO3 | 0 | train |
| 144207865 | CC(C(C)Cl)Cl | 0 | validation |
| 144207864 | C1=CC(=CN=C1)C(=O)NN | 1 | test |
| 144207863 | CC(=O)[C@]1(CC[C@@H]2[C@@]1(CC[C@H]3[C@H]2C=C(C4=CC(=O)CC[C@]34C)Cl)C)OC(=O)C | 0 | train |
| 144207862 | CCCCCCCCCCCCCCOC(=O)C(=C)C | 0 | train |
| 144207861 | CC(=O)O[Si](C)(OC(=O)C)OC(=O)C | 0 | validation |
| 144207860 | C[Si](C1=CC=CC=C1)(C2=CC=CC=C2)Cl | 1 | test |
| 144207859 | C1CCC2C(C1)C(=O)OC2=O | 0 | train |
| 144207858 | C[N+](C)(C)C.[Cl-] | 0 | train |
| 144207857 | CC1(C(=C)[C@@]2(C([C@]1(C(C2(Cl)Cl)(Cl)Cl)Cl)Cl)Cl)C | 1 | validation |
| 144207856 | CC1=CC=CC=C1C | 0 | test |
| 144207855 | C(C(=O)O)Br | 0 | train |
| 144207854 | CCOCCOCC | 0 | train |
| 144207853 | CC(COC(C)(C)C)O | 0 | validation |
| 144207852 | CC(C)OC(=O)/C=C(¥C)/C=C/CC(C)CCCC(C)(C)OC | 1 | test |
| 144207851 | CCOC1=C(C=C(C=C1)NC(=O)C)N | 1 | train |
| 144207850 | CCCCCCC=C | 0 | train |
| 144207849 | C1=CC(=C(C=C1CC2=CC(=C(C=C2)N)Cl)Cl)N | 1 | validation |
| 144207848 | CC(C(=O)O)Cl | 0 | test |
| 144207847 | CC1=CC(=CC(=C1N(C)C)C)OC(=O)NC | 1 | train |
| 144207846 | C1=CC(=C(C=C1C=CC(=O)O)O)O | 0 | train |
| 144207845 | C(=N)(N)S(=O)O | 0 | validation |
| 144207844 | CC(C)(C)C(=O)Cl | 0 | test |
| 144207843 | CCOC(=O)CC(=O)C | 0 | train |
| 144207842 | C(C(=O)Cl)Cl | 0 | train |
| 144207841 | CS(=O)(=O)Cl | 0 | validation |
| 144207840 | CC(C)N(C(C)C)C(=O)SCC(=CCl)Cl | 0 | test |
| 144207839 | CCOC1=CC2=C(C=C1)N=C(S2)N | 1 | train |
| 144207838 | C=C(CCl)Cl | 0 | train |
| 144207837 | C(=C/C(=O)Cl)¥C(=O)Cl | 0 | validation |
| 144207836 | CN(CCCl)CCCl.Cl | 1 | test |
| 144207835 | C1=CC=C(C(=C1)C(=O)Cl)F | 0 | train |
| 144207834 | CCC(=O)C=C | 0 | train |
| 144207833 | C(CC#N)C#N | 0 | validation |
| 144207832 | C1=CC=C(C=C1)C=O | 0 | test |
| 144207831 | CCC(C)Br | 0 | train |
| 144207830 | CC1=CC(=C(C=C1)O)O | 1 | train |
| 144207829 | CC(C)(C)C1=CC(=CC(=C1O)C(C)(C)C)CO | 1 | validation |
| 144207828 | CCC[N+]1(CCCC1)C.C(F)(F)(F)S(=O)(=O)[N-]S(=O)(=O)C(F)(F)F | 0 | test |
| 144207827 | C1=C(C=C(C=C1Cl)Cl)C(=O)Cl | 0 | train |
| 144207826 | C1CCS(=O)(=O)C1 | 0 | train |
| 144207825 | CC(C)(OC)OC | 0 | validation |
| 144207824 | CC[N+]1(CCCC1)C.[I-] | 0 | test |
| 144207823 | CC[N+]1(CCCC1)C.C(F)(F)(F)S(=O)(=O)[N-]S(=O)(=O)C(F)(F)F | 0 | train |
| 144207822 | C1=C(C=C(C(=C1Cl)Cl)O)Cl | 0 | train |
| 144207821 | CCCC(C)(COC(=O)N)COC(=O)NC(C)C | 0 | validation |
| 144207820 | C(CO)[N+](=O)[O-] | 0 | test |
| 144207819 | C1=CC=C(C=C1)C(=O)N | 0 | train |
| 144207818 | C1=CC(=C(C=C1F)F)N | 0 | train |
| 144207817 | CCCBr | 0 | validation |
| 144207816 | CC(=O)CCC(=O)C | 0 | test |
| 144207815 | CN(C)C1=CC=C(C=C1)N(C)C | 1 | train |
| 144207814 | CCCC[N+]1(CCCC1)C.C(=[N-])=NC#N | 0 | train |

| | | | |
|---|---|---|---|
| 144207813 | CCCC[N+]1(CCCC1)C.[I-] | 0 | validation |
| 144207812 | CCCC[N+]1(CCCC1)C.F[P-](F)(F)(F)(F)F | 0 | test |
| 144207811 | CCCCCC[N+]1=CC=CC=C1.F[P-](F)(F)(F)(F)F | 0 | train |
| 144207810 | CCCCCC[N+]1=CC=CC=C1.[Cl-] | 0 | train |
| 144207809 | CCCCCC[N+]1=CC=CC=C1.[Br-] | 0 | validation |
| 144207808 | CCCC[N+]1=CC=CC=C1.[Br-] | 0 | test |
| 144207807 | CCCC[N+]1=CC=CC=C1.F[P-](F)(F)(F)(F)F | 0 | train |
| 144207806 | CCCCN1C=C[N+](=C1C)C.[Br-] | 0 | train |
| 144207805 | CCCCN1C=C[N+](=C1C)C.C(F)(F)(F)S(=O)(=O)[N-]S(=O)(=O)C(F)(F)F | 0 | validation |
| 144207804 | C[N+]1=CN(C=C1)CC2=CC=CC=C2.[Cl-] | 0 | test |
| 144207803 | C[N+]1=CN(C=C1)CC=C.[Cl-] | 0 | train |
| 144207802 | CCCN.[N+](=O)(O)[O-] | 0 | train |
| 144207801 | CCCCCCCC[N+](C)(CCCCCCCC)CCCCCCCC.C(F)(F)(F)S(=O)(=O)[N-]S(=O)(=O)C(F)(F)F | 1 | validation |
| 144207800 | CCCCCCN1C=C[N+](=C1)C.[Br-] | 0 | test |
| 144207799 | CN.[N+](=O)(O)[O-] | 0 | train |
| 144207798 | CCCCCCCCCCCCCCCCN1C=C[N+](=C1)C.[Cl-] | 1 | train |
| 144207797 | C[N+](C)(C)CCO.C(F)(F)(F)S(=O)(=O)[N-]S(=O)(=O)C(F)(F)F | 0 | validation |
| 144207796 | CCN1C=C[N+](=C1)C.F[P-](F)(F)(F)(F)F | 0 | test |
| 144207795 | CCCC[N+](C)(C)C.C(F)(F)(F)S(=O)(=O)[N-]S(=O)(=O)C(F)(F)F | 0 | train |
| 144207794 | CCCCCCCCCCCCN1C=C[N+](=C1)C.F[P-](F)(F)(F)(F)F | 1 | train |
| 144207793 | CCCCCCCCCCCCN1C=C[N+](=C1)C.[Cl-] | 1 | validation |
| 144207792 | CCCCCCCCCCCCN1C=C[N+](=C1)C.C(F)(F)(F)S(=O)(=O)[N-]S(=O)(=O)C(F)(F)F | 1 | test |
| 144207791 | CCCCCCCCCCN1C=C[N+](=C1)C.C(F)(F)(F)S(=O)(=O)[O-] | 0 | train |
| 144207790 | [B-](F)(F)(F)F.CCCCCCCCCCN1C=C[N+](=C1)C | 0 | train |
| 144207789 | CCCCN1C=C[N+](=C1)C.C(#N)[S-] | 0 | validation |
| 144207788 | CCCCCCCCCCN1C=C[N+](=C1)C.C(F)(F)(F)S(=O)(=O)[N-]S(=O)(=O)C(F)(F)F | 0 | test |
| 144207787 | CCCCN1C=C[N+](=C1)C.CS(=O)(=O)[O-] | 0 | train |
| 144207786 | CCCCN1C=C[N+](=C1)C.F[P-](F)(F)(F)(F)F | 0 | train |
| 144207785 | CCCCN1C=C[N+](=C1)C.C(=[N-])=NC#N | 0 | validation |
| 144207784 | [B-](F)(F)(F)F.CCCCN1C=C[N+](=C1C)C | 0 | test |
| 144207783 | CCCCN1C=C[N+](=C1C)C.[I-] | 0 | train |
| 144207782 | CCCCN1C=C[N+](=C1C)C.F[P-](F)(F)(F)(F)F | 0 | train |
| 144207781 | CCCCN1C=C[N+](=C1C)C.[Cl-] | 0 | validation |
| 144207780 | CCCC[N+](C)(CCCC)CCCC.C(F)(F)(F)S(=O)(=O)[N-]S(=O)(=O)C(F)(F)F | 0 | test |
| 144207779 | CCCCCCCC[N+](C)(CCCCCCCC)CCCCCCCC.C(F)(F)(F)S(=O)(=O)[O-] | 1 | train |
| 144207778 | CNC.[N+](=O)(O)[O-] | 0 | train |
| 144207777 | C(CO)N.C(=O)O | 0 | validation |
| 144207776 | CCN.[N+](=O)(O)[O-] | 0 | test |
| 144207775 | CCN1C=C[N+](=C1)C.[Cl-] | 0 | train |
| 144207774 | CCCCCCCC[P+](CCCCCCCC)(CCCCCCCC)CCCCCCCC.[Br-] | 1 | train |
| 144207773 | CC1=CC=C(C=C1)S(=O)(=O)[O-].CC(C)C[P+](C)(CC(C)C)CC(C)C | 0 | validation |
| 144207772 | C1=CC=NC(=C1)C(=O)[O-].C1=CC=NC(=C1)C(=O)[O-].C1=CC=NC(=C1)C(=O)[O-].[Cr+3] | 0 | test |
| 144207771 | CC(C)(C#N)C(C)(C)C#N | 0 | train |
| 144207770 | C1=CC(=CC=C1CCl)[N+](=O)[O-] | 0 | train |
| 144207769 | C(C(Br)(Br)Br)(Br)Br | 0 | validation |
| 144207768 | CC(=CCCC(C)(C=C)OC(=O)C1=CC=CC=C1N)C | 0 | test |
| 144207767 | CC(=O)C=CC1=CC=CO1 | 0 | train |
| 144207766 | CC1=C2C(=C(C(=C1C)OC(=O)CCC(=O)O)C)CC[C@@](O2)(C)CCC[C@H](C)CCC[C@H](C)CCCC(C)C | 0 | train |
| 144207765 | CC1=CC2=C(C=C1)C3=CC=CC=C3C=C2 | 1 | validation |
| 144207764 | C(C(F)(Cl)Cl)(F)(Cl)Cl | 0 | test |
| 144207763 | C[C@]12CCC[C@@]([C@H]1CC[C@@]34[C@H]2C[C@H]([C@H](C3)C(=C)C4)O[C@H]5[C@@H]([C@H]([C@@H]([C@H](O5)CO)O)O[C@H]6[C@@H]([C@H]([C@@H]([C@H](O6)CO)O)O)O)OC7[C@@H]([C@H]([C@@H]([C@H](O7)CO)O)O)O)(C)C(=O)O[C@H]8[C@@H]([C@H]([C@@H]([C@H](O8)CO)O)O)O | 0 | train |
| 144207762 | C([C@H]([C@H](CO)O)O)O | 0 | train |
| 144207761 | C1[C@H]([C@@H]([C@@H]([C@@](O1)(CO)O)O)O)O | 0 | validation |
| 144207760 | CC1=CC=C(C=C1)C=C2C3CCC(C2=O)C3(C)C | 1 | test |
| 144207759 | CC1CCC(C(C1)OC(=O)C2=CC=CC=C2N)C(C)C | 1 | train |
| 144207758 | C1=CC=C(C=C1)C(=O)C2=C(C=CC(=C2)Cl)O | 1 | train |
| 144207757 | C1CC2C(C1)C3CC2CC3(C4=CC=C(C=C4)O)C5=CC=C(C=C5)O | 0 | validation |

| | | | |
|---|---|---|---|
| 144207756 | C1=CC(=CC=C1C(=C(Cl)Cl)C2=CC=C(C=C2)O)O | 1 | test |
| 144207755 | CC(C)(C1=CC(=C(C(=C1)Cl)O)Cl)C2=CC(=C(C(=C2)Cl)O)Cl | 1 | train |
| 144207754 | CC(C)(C1=CC=CC=C1)C2=CC=CC=C2 | 1 | train |
| 144207753 | CC(C1=CC=C(C=C1)O)C2=CC=C(C=C2)O | 1 | validation |
| 144207752 | CC1=C(C=CC(=C1)C(C)(C)C2=CC(=C(C=C2)O)C)O | 1 | test |
| 144207751 | CCN1C=C[N+](=C1)C.CCOP(=O)([O-])OCC | 0 | train |
| 144207750 | CC[S+](CC)CC.C(F)(F)(F)S(=O)(=O)[N-]S(=O)(=O)C(F)(F)F | 0 | train |
| 144207749 | CCCCCOC(=O)C1=CC=C(C=C1)O | 1 | validation |
| 144207748 | C1=CC(=CC=C1C(=O)C2=CC=C(C=C2)O)O | 1 | test |
| 144207747 | COC1=CC(=C(C=C1)C(=O)C2=CC(=CC=C2)O)OC | 0 | train |
| 144207746 | CCCCCCCCCCOC(=O)C1=CC=C(C=C1)O | 0 | train |
| 144207745 | CCN1C=C[N+](=C1)C.C(#N)[S-] | 0 | validation |
| 144207744 | CCCCCCCCCCCCOC(=O)C1=CC=C(C=C1)O | 0 | test |
| 144207743 | C1=CC=C(C=C1)OC(=O)C2=CC=C(C=C2)O | 0 | train |
| 144207742 | CC1=CC(=C(C=C1)O)C(=O)C2=CC=CC=C2 | 0 | train |
| 144207741 | COC1=CC(=C(C=C1)C(=O)C2=C(C=C(C=C2)OC)O)O | 1 | validation |
| 144207740 | C1=CC=C(C=C1)C(=O)C2=CC(=CC=C2)O | 1 | test |
| 144207739 | C1=CC=C(C=C1)C(=O)C2=CC=CC=C2O | 0 | train |
| 144207738 | CCCCCCCOC(=O)C1=CC=C(C=C1)O | 1 | train |
| 144207737 | C[C@]12C=CC3=C4CCC(=O)C=C4CC[C@H]3[C@@H]1CC[C@@H]2O | 1 | validation |
| 144207736 | CCCCCCCCCCC=O | 0 | test |
| 144207735 | CCCCCCCCCCCCC(=O)O | 0 | train |
| 144207734 | C(=O)(C(Cl)(Cl)Cl)N | 0 | train |
| 144207733 | C(=O)C(Br)(Br)Br | 1 | validation |
| 144207732 | C(C(=O)O)C(=O)O | 1 | test |
| 144207731 | C1=CC=C(C=C1)CC(=O)O | 1 | train |
| 144207730 | CCCC(=O)C(=O)O | 1 | train |
| 144207729 | C(CCCC(=O)O)CCC(=O)O | 0 | validation |
| 144207728 | CCC(C)C(=O)C | 0 | test |
| 144207727 | CC(CCC(=O)O)C(=O)O | 0 | train |
| 144207726 | CC(C)CC#N | 1 | train |
| 144207725 | C1=CC(=CC(=C1)O)C(=O)O | 0 | validation |
| 144207724 | CCCCCCC#N | 0 | test |
| 144207723 | CC(C)(CCC(=O)O)C(=O)O | 0 | train |
| 144207722 | CC(C)(CC(=O)O)C(=O)O | 0 | train |
| 144207721 | C1=C(C(=CC(=C1O)Cl)Cl)O | 0 | validation |
| 144207720 | C1CCC(=O)C(C1)Cl | 0 | test |
| 144207719 | CC(C)(C)C1=CC(=O)C=C(C1=O)C(C)(C)C | 0 | train |
| 144207718 | [B-](F)(F)(F)F.[Na+] | 0 | train |
| 144207717 | CC1=CC(=C(C=C1)N)C.Cl | 0 | validation |
| 144207716 | CC1=CC(=C(C=C1)C)C | 0 | test |
| 144207715 | CC1=CC=C(C=C1)N.Cl | 1 | train |
| 144207714 | C[C@@]1([C@H]2C[C@H]3[C@@H](C(=O)C(=C([C@]3(C(=O)C2=C(C4=C1C=CC=C4O)O)O)O)O<br>)C(=O)N)N(C)C)O.Cl | 0 | train |
| 144207713 | C1=CC=C2C(=C1)C(=O)[N-]S2(=O)=O.[Na+] | 0 | validation |
| 144207712 | CN(C)CCN(CC1=CC=C(C=C1)OC)C2=CC=CC=N2.C(=C¥C(=O)O)¥C(=O)O | 0 | test |
| 144207711 | C1=CC(=CC2=NC3=C(C=CC(=C3)N)C=C21)N.Cl | 0 | train |
| 144207710 | C1=CC=C(C(=C1)C(=O)N)C(=O)N | 0 | train |
| 144207709 | C1=CC=C(C=C1)N/N=C/2¥C(=O)C=CC3=CC=CC=C32 | 1 | validation |
| 144207708 | CCC1(C(=O)NC(=O)NC1=O)C2=CC=CC=C2 | 0 | test |
| 144207707 | CC1=CN=C(C(=C1OC)C)CS(=O)C2=NC3=C(N2)C=C(C=C3)OC | 1 | train |
| 144207706 | C1=CC2=C(C=CC(=C2)[N+](=O)[O-])N=C1 | 0 | train |
| 144207705 | C1=CC=C2C(=C1)C=CC(=C2S(=O)(=O)O)N | 1 | validation |
| 144207704 | CC1=C(C=CC(=C1)O)O | 1 | test |
| 144207703 | CC(C1=CC=CC=C1)O | 0 | train |
| 144207702 | COC1=CC=CC(=C1O)O | 1 | train |
| 144207701 | C1(=NC(=NC(=N1)N)N)N | 0 | validation |
| 144207700 | C1=CN=CC=C1C(=O)O | 0 | test |
| 144207699 | CC(C)COC(=O)C1=CC=C(C=C1)O | 1 | train |
| 144207698 | C1=CC(=CC=C1C(=O)O)NN.Cl | 1 | train |
| 144207697 | C1COS(=O)O1 | 0 | validation |
| 144207696 | C1COS(=O)(=O)O1 | 0 | test |

| | | | |
|---|---|---|---|
| 144207695 | CC1=C(C(=O)N(N1C)C2=CC=CC=C2)N(C)CS(=O)(=O)[O-].[Na+] | 0 | train |
| 144207694 | CC1(C(=O)NC(=O)O1)C | 0 | train |
| 144207693 | C1C2C3CC4C(C3C1C5C2O5)O4 | 0 | validation |
| 144207692 | COC1=C(C=C(C=C1)N)N.OS(=O)(=O)O | 1 | test |
| 144207691 | C1CCC(=NO)C1 | 1 | train |
| 144207690 | C[N+](C)(C)CCO.[Cl-] | 0 | train |
| 144207689 | C[C@H](CCC(=O)O)[C@H]1CC[C@@H]2[C@@]1(CC[C@H]3[C@H]2[C@@H](C[C@H]4[C@@]3(CC[C@H](C4)O)C)O)C | 0 | validation |
| 144207688 | C1CC(=O)OC1 | 0 | test |
| 144207687 | C1=CC=C(C=C1)CNN.Cl.Cl | 1 | train |
| 144207686 | O.O.[Cl-].[Cl-].[Ba+2] | 0 | train |
| 144207685 | CN1[C@@H]2CC[C@H]1CC(C2)OC(=O)C(CO)C3=CC=CC=C3 | 0 | validation |
| 144207684 | CCN1C2=C(C=C(C=C2)N)C3=CC=CC=C31.Cl | 1 | test |
| 144207683 | CCCC(CC)CCCCCCCN | 0 | train |
| 144207682 | O=S(=O)(Cl)Cl | 0 | train |
| 144207681 | OS(=O)(=O)O | 0 | validation |
| 144207680 | CCCCCCCC[Sn](CCCCCCCC)(Cl)Cl | 0 | test |
| 144207679 | C(#N)[S-].[K+] | 0 | train |
| 144207678 | CC1=C(C=CC(=C1)OC)Br | 0 | train |
| 144207677 | C[N+]1(CCOCC1)[O-] | 0 | validation |
| 144207676 | CC[C@H](CO)N | 0 | test |
| 144207675 | COC(=O)C1CCC(CC1)C(=O)OC | 0 | train |
| 144207674 | CC(C)(C1=CC=C(C=C1)C(C)(C)O)O | 0 | train |
| 144207673 | C1=CC2=C(C=C1C(=O)C3=CC4=C(C=C3)C(=O)OC4=O)C(=O)OC2=O | 0 | validation |
| 144207672 | CC1=CN=C(C=C1)N | 0 | test |
| 144207671 | CC1=CC=CC=[N+]1[O-] | 0 | train |
| 144207670 | CCC1=CC=C(C=C1)C | 0 | train |
| 144207669 | C1CN(CCN1CCO)CCO | 0 | validation |
| 144207668 | CCCC(C)C | 0 | test |
| 144207667 | C1=CC(=CC=C1C2=CC=C(C=C2)O)O | 1 | train |
| 144207666 | CC(C)(CC(=O)OC)C=C | 0 | train |
| 144207665 | CC(C)(C1=CC=C(C=C1)OC2=CC3=C(C=C2)C(=O)OC3=O)C4=CC=C(C=C4)OC5=CC6=C(C=C5)C(=O)OC6=O | 0 | validation |
| 144207664 | CC(C)CCCCCOC(=O)C1=CC=CC=C1C(=O)OCCCCCC(C)C | 1 | test |
| 144207663 | CCC(C)(C)C1=CC(=C(C(=C1)N2N=C3C=CC=CC3=N2)O)C(C)(C)CC | 0 | train |
| 144207662 | CC/C(=N/O[Si](O/N=C(/CC)¥C)(O/N=C(/CC)¥C)C)/C | 0 | train |
| 144207661 | C=CC(=O)[O-].C=CC(=O)[O-].[Zn+2] | 0 | validation |
| 144207660 | CC(C)OC(=O)CCCCC(=O)OC(C)C | 0 | test |
| 144207659 | CCCCC(CC)COC(=O)C1=CC=C(C=C1)C(=O)OCC(CC)CCCC | 0 | train |
| 144207658 | CC(CO)(CO)C(=O)O | 0 | train |
| 144207657 | COC(=O)C1=CC(=CC(=C1)S(=O)(=O)[O-])C(=O)OC.[Na+] | 0 | validation |
| 144207656 | C1C=CCC2C1CC=C2 | 0 | test |
| 144207655 | CC1=C(C(=CC=C1)N)N | 1 | train |
| 144207654 | CCCCCCCCCCCCCCCCl | 0 | train |
| 144207653 | C1=CC=C(C=C1)[P+](=O)O | 0 | validation |
| 144207652 | C1CCC(CC1)S | 0 | test |
| 144207651 | CO[Si](C)(OC)OC | 0 | train |
| 144207650 | CC(C)(C)C1=CC=C(C=C1)C=O | 0 | train |
| 144207649 | C(C=CCCl)Cl | 0 | validation |
| 144207648 | C(CCCO)CCO | 0 | test |
| 144207647 | CC(C)(C1=CC=CC=C1)O | 0 | train |
| 144207646 | CC(C)(C)C(=O)OC | 0 | train |
| 144207645 | CC(C(=O)OC)O | 0 | validation |
| 144207644 | C1CC2=CC=CC=C2C(=O)C1 | 0 | test |
| 144207643 | C1CCC(=O)OCC1 | 0 | train |
| 144207642 | CCCCCCCC/C=C¥CCCCCCCC(=O)N | 0 | train |
| 144207641 | CC(C)C(C(C)(C)CO)O | 0 | validation |
| 144207640 | CC(C)(C)C1=C(C(=CC=C1)C(C)(C)C)O | 0 | test |
| 144207639 | C(C(CO)(CO)[N+](=O)[O-])O | 0 | train |
| 144207638 | COP(OC)OC | 0 | train |
| 144207637 | C12C(C(=O)OC1=O)C3(C(=C(C2(C3(Cl)Cl)Cl)Cl)Cl)Cl | 0 | validation |
| 144207636 | CCCCCCCCCCCCCCCCCC[N+](C)(C)C.[Cl-] | 0 | test |

| | | | |
|---|---|---|---|
| 144207635 | CCCCN=C=O | 0 | train |
| 144207634 | CC(C)CC(C)O | 0 | train |
| 144207633 | CC(=C)CC(C)(C)C | 0 | validation |
| 144207632 | CCOC(=O)CC#N | 0 | test |
| 144207631 | C1CC(CCC1CO)CO | 0 | train |
| 144207630 | C1=CC(=CC=C1[N+](=O)[O-])OC2=CC=C(C=C2)[N+](=O)[O-] | 0 | train |
| 144207629 | C1=CC=C(C=C1)C(=O)Cl | 0 | validation |
| 144207628 | CO[C@@H]1[C@@H]([C@H]([C@@H]([C@H](O1)CO)O)O)O | 0 | test |
| 144207627 | C([C@@H]1[C@H]([C@@H]([C@H](C(=O)O1)O)O)O)O | 0 | train |
| 144207626 | CC1=CC(=C(C=C1Cl)N)S(=O)(=O)O | 0 | train |
| 144207625 | CCCCCCCCCCOC(=O)C1=CC=CC=C1C(=O)OCCCCCCCCCC | 0 | validation |
| 144207624 | CCO[Si](OCC)(OCC)OCC | 0 | test |
| 144207623 | CC(C)(C)C(=O)O | 0 | train |
| 144207622 | CC(=O)[O-].CC(=O)[O-].[Co+2] | 0 | train |
| 144207621 | CCCCN(CCCC)CCCC | 0 | validation |
| 144207620 | C(SCl)(Cl)(Cl)Cl | 0 | test |
| 144207619 | CC(C)CCCCCCCOC(=O)C(=C)C | 0 | train |
| 144207618 | COCCOC | 0 | train |
| 144207617 | CCCCCCCCCCCCCCCC[N+](C)(C)CC1=CC=CC=C1.[Cl-] | 1 | validation |
| 144207616 | CCCCNCCCC | 0 | test |
| 144207615 | CC(O)(P(=O)(O)O)P(=O)(O)O | 0 | train |
| 144207614 | CCC(C)C1=CC=C(C=C1)O | 0 | train |
| 144207613 | CC(=C)C(=O)OCCO | 0 | validation |
| 144207612 | CC(=O)CC(=O)C | 0 | test |
| 144207611 | CCOCCOCCO | 0 | train |
| 144207610 | C1=CC=C(C(=C1)C(=O)N)N | 0 | train |
| 144207609 | C1=CC=C(C(=C1)C(=O)[O-])O.[Na+] | 0 | validation |
| 144207608 | C(Br)Br | 0 | test |
| 144207607 | C1=CC(=CC(=C1)C(=O)O)C(=O)O | 0 | train |
| 144207606 | CC(C(Cl)Cl)Cl | 0 | train |
| 144207605 | O[Se](=O)O | 1 | validation |
| 144207604 | C1=CC(=O)OC1=O | 0 | test |
| 144207603 | C1CSCCS1 | 0 | train |
| 144207602 | CCC(C)Cl | 0 | train |
| 144207601 | CCN(CC)C(=S)[S-].[Na+] | 1 | validation |
| 144207600 | CC1=CC=C(C=C1)O | 0 | test |
| 144207599 | C(C(F)(Cl)Cl)(F)(F)Cl | 0 | train |
| 144207598 | CC1=CC[C@@H](CC1)C(=C)C | 0 | train |
| 144207597 | C1=CC(=CC=C1Cl)Cl | 0 | validation |
| 144207596 | CC(COC1=CC=C(C=C1)C(C)(C)C)OS(=O)OCCCl | 0 | test |
| 144207595 | CC[C@H]1CN2CCC3=CC(=C(C=C3[C@@H]2C[C@@H]1C[C@@H]4C5=CC(=C(C=C5CCN4)OC)OC)OC)OC.Cl.Cl | 1 | train |
| 144207594 | CN1CCC2=CC3=C(C=C2C1O)OCO3.Cl | 0 | train |
| 144207593 | CCCCCCCCS(=O)C(C)CC1=CC2=C(C=C1)OCO2 | 1 | validation |
| 144207592 | CC(C)(CCCC1=CCC(CC1)C=O)O | 0 | test |
| 144207591 | COC1=CC(=NC(=N1)NC(=O)NS(=O)(=O)CC2=CC=CC=C2C(=O)OC)OC | 0 | train |
| 144207590 | C(Cl)(Cl)(Br)Br | 0 | train |
| 144207589 | C1=CC(=C(C=C1C(F)(F)F)Cl)OC2=CC(=C(C=C2)[N+](=O)[O-])C(=O)[O-].[Na+] | 0 | validation |
| 144207588 | CCOC(=O)[C@H](CCC1=CC=CC=C1)N[C@@H](C)C(=O)N2CC3=CC=CC=C3C[C@H]2C(=O)O.Cl | 0 | test |
| 144207587 | C=CCOCC1CO1 | 0 | train |
| 144207586 | C=CC1CCC=CC1 | 0 | train |
| 144207585 | CC1=C(C=CC(=C1)[N+](=O)[O-])N | 0 | validation |
| 144207584 | C1=CC(=CC(=C1)[N+](=O)[O-])N | 0 | test |
| 144207583 | C1=CC(=CC(=C1)Cl)C(F)(F)F | 0 | train |
| 144207582 | C1=C(C=C(C(=C1Cl)O)SC2=CC(=CC(=C2O)Cl)Cl)Cl | 0 | train |
| 144207581 | CC1CCCO1 | 0 | validation |
| 144207580 | C(C(CBr)Br)Cl | 0 | test |
| 144207579 | C1=CC(=C(C=C1Cl)N)Cl | 0 | train |
| 144207578 | CC1=CC(=C(C=C1)N)C | 0 | train |
| 144207577 | CC1=CC=CC=C1Br | 0 | validation |
| 144207576 | CCCCOC(=O)C1=CC=C(C=C1)N | 1 | test |

| | | | |
|---|---|---|---|
| 144207575 | C1CCC(CC1)(C#N)O | 0 | train |
| 144207574 | C1=CC=C(C=C1)C2=CC=C(C=C2)N | 1 | train |
| 144207573 | CCN(CC1=CC=CC=C1)C2=CC=CC=C2 | 1 | validation |
| 144207572 | C1CNCC2=CC=CC=C21 | 0 | test |
| 144207571 | C1=CC=C(C(=C1)C=O)Cl | 0 | train |
| 144207570 | CC1=CC(=C(C=C1)C)[N+](=O)[O-] | 0 | train |
| 144207569 | CCC(C)NC1=CC=C(C=C1)NC(C)CC | 1 | validation |
| 144207568 | CC1=C(C=CC=C1Cl)N | 0 | test |
| 144207567 | CCN(C1=CC=CC=C1)C(=O)N(CC)C2=CC=CC=C2 | 1 | train |
| 144207566 | CO[Si](CCCNCCC[Si](OC)(OC)OC)(OC)OC | 0 | train |
| 144207565 | CC(=C)C(=O)OC | 0 | validation |
| 144207564 | CCNS(=O)(=O)C1=CC=C(C=C1)C | 0 | test |
| 144207563 | CC(C)[N+](=O)[O-] | 0 | train |
| 144207562 | C(C(Br)Br)(Br)Br | 0 | train |
| 144207561 | CC(=O)C=C | 0 | validation |
| 144207560 | CC(CCl)Cl | 0 | test |
| 144207559 | CCOP(=O)(CC)OCC | 0 | train |
| 144207558 | CC(C)NC1=CC=CC=C1 | 0 | train |
| 144207557 | CC(C)(C)OC=O | 0 | validation |
| 144207556 | CCC(C)(C)C | 0 | test |
| 144207555 | C=C(Cl)Cl | 0 | train |
| 144207554 | CC(C)Br | 0 | train |
| 144207553 | CCBr | 0 | validation |
| 144207552 | C1=CC=C(C=C1)NC2=CC=C(C=C2)NC3=CC=CC=C3 | 1 | test |
| 144207551 | CC1=CC(=NO1)NS(=O)(=O)C2=CC=C(C=C2)N | 0 | train |
| 144207550 | C1=CC(=C(C=C1[N+](=O)[O-])[N+](=O)[O-])F | 1 | train |
| 144207549 | CC(C)CCOCC(=O)OCC=C | 0 | validation |
| 144207548 | C1=CC=C(C=C1)/C=C¥C2=CC=CC=C2 | 0 | test |
| 144207547 | CCOC(C)(C)C | 0 | train |
| 144207546 | COC1=C(C2=C[N+]3=C(C=C2C=C1)C4=CC5=C(C=C4CC3)OCO5)OC.[Cl-] | 1 | train |
| 144207545 | C(CCC(=O)N)CC(=O)N | 0 | validation |
| 144207544 | CCCC[N+](=O)[O-] | 0 | test |
| 144207543 | C1=CC=C(C=C1)N | 0 | train |
| 144207542 | CC1=C(C=CC(=C1)NC2=CC=C(C=C2)O)N | 1 | train |
| 144207541 | C1=CC(=CC=C1C(=O)N)[N+](=O)[O-] | 0 | validation |
| 144207540 | C(C(CCl)Cl)O | 0 | test |
| 144207539 | CC1=CC=CC2=C1N=CC=C2 | 0 | train |
| 144207538 | C1=CC=C(C(=C1)C(=O)Cl)[N+](=O)[O-] | 0 | train |
| 144207537 | C1(=C(C(=C(C(=C1Br)Br)Br)Br)Br)O | 0 | validation |
| 144207536 | CC(=O)OC1=CC=C(C=C1)C(C2=CC=C(C=C2)OC(=O)C)C3=CC=CC=N3 | 1 | test |
| 144207535 | CCC([N+](=O)[O-])Cl | 0 | train |
| 144207534 | CC(C)([N+](=O)[O-])Cl | 0 | train |
| 144207533 | CC(=O)OCC=C | 0 | validation |
| 144207532 | CC1=CC(=CC=C1)Br | 0 | test |
| 144207531 | CC1=CC(=C(C=C1)[N+](=O)[O-])N | 0 | train |
| 144207530 | CCCC(C)(COC(=O)N)COC(=O)N | 0 | train |
| 144207529 | CC=C(Cl)Cl | 0 | validation |
| 144207528 | CC(=O)NC1=CC=C(C=C1)C(=O)O | 0 | test |
| 144207527 | C1=CC=C2C(=C1)C=CC=C2N=C=S | 1 | train |
| 144207526 | C1=CC2=C(C=C1[N+](=O)[O-])C(=O)OC2=O | 0 | train |
| 144207525 | C1=CC(=C(C=C1Br)Br)OC2=C(C=C(C=C2)Br)Br | 1 | validation |
| 144207524 | C1=CC=C(C=C1)CSCC2=CC=CC=C2 | 1 | test |
| 144207523 | C1=CC(=CC(=C1)Cl)C(=O)O | 0 | train |
| 144207522 | CC1=CC=CC=C1C#N | 0 | train |
| 144207521 | CN1C2=C(C(=O)N(C1=O)C)NC(=N2)Cl.CN(C)CCOC(C1=CC=CC=C1)C2=CC=CC=C2 | 0 | validation |
| 144207520 | C1=CC(=C(C=C1C(=O)O)Cl)Cl | 0 | test |
| 144207519 | C1=CC(=C(C=C1Cl)C(=O)O)Cl | 0 | train |
| 144207518 | C1=CC(=C(C(=C1)Cl)C(=O)O)Cl | 0 | train |
| 144207517 | CC(C)COC(=O)COC1=CC(=C(C=C1Cl)Cl)Cl | 0 | validation |
| 144207516 | C1=CC(=C(C(=C1)Cl)C(=O)Cl)Cl | 0 | test |
| 144207515 | C/C=C(/C)¥C#N | 0 | train |
| 144207514 | CC=CC=O | 0 | train |

| | | | |
|---|---|---|---|
| 144207513 | CC(C)/C=C/CCCCC(=O)NCC1=CC(=C(C=C1)O)OC | 1 | validation |
| 144207512 | C/C=C¥1/C(=O)C[C@@H]2[C@@]1(CC[C@H]3[C@H]2CCC4=CC(=O)CC[C@]34C)C | 1 | test |
| 144207511 | C[C@@H]1CC[C@H](C2=C(CC[C@H]12)C)/C=C(¥C)/C(=O)O | 0 | train |
| 144207510 | C1=C(C(=C(C(=C1Br)Br)Br)Br)OC2=CC(=C(C(=C2Br)Br)Br)Br | 0 | train |
| 144207509 | CCCCCCCCCCOC(=O)C(=C)C | 0 | validation |
| 144207508 | CC(C)(C)C1=CC=C(C=C1)OCC2CO2 | 1 | test |
| 144207507 | COC(=O)C(C1CCCCN1)C2=CC=CC=C2.Cl | 0 | train |
| 144207506 | C1=CC=C(C=C1)NC2=CC(=CC=C2)O | 1 | train |
| 144207505 | CN(C)CCOC(=O)C=C | 0 | validation |
| 144207504 | CCCCCCCCC1CO1 | 0 | test |
| 144207503 | C1=CC(=CC=C1N/N=C/2¥C=CC(=O)C(=C2)C(=O)O)[N+](=O)[O-] | 1 | train |
| 144207502 | CC1=C(C(C(=C(N1)C)C(=O)OC)C2=CC=CC=C2[N+](=O)[O-])C(=O)OC | 1 | train |
| 144207501 | C[C@H]1[C@@H]([C@H]([C@H]([C@@H](O1)O[C@@H]2[C@H]([C@@H]([C@H](O[C@H]2OC3<br>=CC(=C(C(=C3)O)C(=O)CCC4=CC(=C(C=C4)OC)O)O)CO)O)O)O)O)O | 0 | validation |
| 144207500 | CNC.C1=CC(=C(C=C1Cl)Cl)OCC(=O)O | 0 | test |
| 144207499 | CC(C)CC1=NC=CS1 | 0 | train |
| 144207498 | C1CC(CCC1CC2CCC(CC2)N)N | 0 | train |
| 144207497 | C1CCC2=NC3=CC=CC=C3C(=C2C1)N.Cl | 0 | validation |
| 144207496 | CNC1=CC=CC=C1 | 0 | test |
| 144207495 | C1=CC(=C(C(=C1O)Cl)Cl)Cl | 0 | train |
| 144207494 | COC1=CC(=O)OC(=C1)/C=C/C2=CC=CC=C2 | 1 | train |
| 144207493 | C1=CC=C(C(=C1)CCO)[N+](=O)[O-] | 0 | validation |
| 144207492 | CCCC(=O)Cl | 0 | test |
| 144207491 | CC(=O)C1=C(C(=C(C=C1)Cl)Cl)Cl | 0 | train |
| 144207490 | CC1=CC(=CC=C1)C | 0 | train |
| 144207489 | C1=CC2=C(C(=C(C=C2Cl)I)O)N=C1 | 1 | validation |
| 144207488 | C1C2C(C(C1Cl)Cl)C3(C(=C(C2(C3(Cl)Cl)Cl)Cl)Cl)Cl | 1 | test |
| 144207487 | C1C(O1)COC2=CC=CC=C2 | 0 | train |
| 144207486 | CC1=CC=CC=C1NC(=O)C | 0 | train |
| 144207485 | C1=CC=C2C(=C1)C=NNC2=O | 0 | validation |
| 144207484 | C1=CC=C(C(=C1)C(=O)O)Cl | 0 | test |
| 144207483 | C1=CC(=CC=C1CCO)[N+](=O)[O-] | 0 | train |
| 144207482 | C(CCl)OCOCCCl | 0 | train |
| 144207481 | C(CO)N(CCO)N=O | 0 | validation |
| 144207480 | CCCCC#N | 0 | test |
| 144207479 | CC(C)(CCC(C)(C)O)O | 0 | train |
| 144207478 | CCCC=C | 0 | train |
| 144207477 | C1=C(C=C(C=C1Cl)Cl)Cl | 0 | validation |
| 144207476 | CC(=O)OC(=O)C | 0 | test |
| 144207475 | C1=CC=C(C=C1)C(=O)C=O | 0 | train |
| 144207474 | C1C(O1)CCl | 0 | train |
| 144207473 | CC1=CC(=C(O1)C)C(=O)C | 0 | validation |
| 144207472 | CCOC(C)OCC | 0 | test |
| 144207471 | C1=CC(=CC=C1NO)N=O | 1 | train |
| 144207470 | C1=CC=C(C=C1)C=CC=O | 0 | train |
| 144207469 | C1=CC=C(C=C1)NC=O | 0 | validation |
| 144207468 | C=CCN1C(=O)N(C(=O)N(C1=O)CC=C)CC=C | 0 | test |
| 144207467 | C1=CC=C(C=C1)NC(=S)NC2=CC=CC=C2 | 1 | train |
| 144207466 | C1=CC(=CC=C1CC2=CC=C(C=C2)N=C=O)N=C=O | 0 | train |
| 144207465 | C1CCN(CC1)N=O | 0 | validation |
| 144207464 | C1=CC=C(C=C1)CCl | 0 | test |
| 144207463 | CC(=O)C1=CC=C(C=C1)[N+](=O)[O-] | 0 | train |
| 144207462 | CCN(CC)O | 0 | train |
| 144207461 | CCN(CC)C(=S)[S-].CCN(CC)C(=S)[S-].[Zn+2] | 1 | validation |
| 144207460 | COC1=C(C=C2C(=C1)C3=CC=CC=C3O2)N | 1 | test |
| 144207459 | CC[N+]1(CCCC1)C.F[P-](F)(F)(F)(F)F | 0 | train |
| 144207458 | CC1=CC2=C(C=C1)C=C(C=C2)C | 0 | train |
| 144207457 | C1=C(C(=NC(=C1Cl)Cl)Cl)Cl | 0 | validation |
| 144207456 | CC(CN1CC(=O)NC(=O)C1)N2CC(=O)NC(=O)C2 | 0 | test |
| 144207455 | C1=CC(=CC=C1C(Cl)(Cl)Cl)C(Cl)(Cl)Cl | 0 | train |
| 144207454 | [N+](=O)([O-])[O-].[Tl+] | 0 | train |
| 144207453 | CC1=NC(=NC(=N1)OC)NC(=O)NS(=O)(=O)C2=CC=CC=C2Cl | 0 | validation |

| | | | |
|---|---|---|---|
| 144207452 | C1=CC=C(C=C1)C2=CC=C(C=C2)Br | 0 | test |
| 144207451 | CC(C)(C)OCC1CO1 | 0 | train |
| 144207450 | CC(=O)NC1=CC(=CC=C1)O | 0 | train |
| 144207449 | COC1=C2C(=CC=C1)SC(=N2)N | 1 | validation |
| 144207448 | CCCCCCCCCCCCC1CO1 | 0 | test |
| 144207447 | C(C(CBr)(CBr)CBr)O | 0 | train |
| 144207446 | CCCCC | 0 | train |
| 144207445 | C1=CC(=CC(=C1)O)C[C@@H](CO)[C@@H](CC2=CC(=CC=C2)O)CO | 0 | validation |
| 144207444 | O.O.O.O.O.O.[Mg+2].[Cl-].[Cl-] | 0 | test |
| 144207443 | CC(C)C(C(=O)OC(C#N)C1=CC(=CC=C1)OC2=CC=CC=C2)NC3=C(C=C(C=C3)C(F)(F)F)Cl | 1 | train |
| 144207442 | C1CCC(=O)C1 | 0 | train |
| 144207441 | CN(C)CCOCCO | 0 | validation |
| 144207440 | CCCCCCCC/C=C¥CCCCCCCCCCCC(=O)O | 0 | test |
| 144207439 | CN(C)CCN(C)C | 0 | train |
| 144207438 | [C-]#N.[C-]#N.[K+].[Ag+] | 1 | train |
| 144207437 | CCOP(=O)(OCC)SCCSCC | 1 | validation |
| 144207436 | CCN(CC)C1=CC(=CC=C1)O | 1 | test |
| 144207435 | CC1(C2CCC1(C(=O)C2)C)C | 0 | train |
| 144207434 | CCCCC(CC)COC(=O)C(=C(C1=CC=CC=C1)C2=CC=CC=C2)C#N | 1 | train |
| 144207433 | C(C=CCl)Cl | 0 | validation |
| 144207432 | C1CCC(CC1)N=C=O | 1 | test |
| 144207431 | CC1=C2C(=CC=C1)SC(=N2)N | 1 | train |
| 144207430 | CC1=CC(=CC(=C1)N)C | 0 | train |
| 144207429 | CC1(C(=O)N(C(=O)O1)C)C | 0 | validation |
| 144207428 | C1=CN=CC=C1C(=O)N | 0 | test |
| 144207427 | CCOC(C=C)OCC | 0 | train |
| 144207426 | CC(=O)C1=CC=C(C=C1)O | 0 | train |
| 144207425 | C(CS(=O)(=O)[O-])O.[Na+] | 0 | validation |
| 144207424 | C(CSCCC#N)C#N | 0 | test |
| 144207423 | CCCCCCCC/C=C¥CCCCCCCC(=O)OC | 0 | train |
| 144207422 | CCCCCCCC/C=C¥CCCCCCCC(=O)OCCOC(=O)CCCCCCC/C=C¥CCCCCCCC | 0 | train |
| 144207421 | CC[N+]1=CC=CC=C1.F[P-](F)(F)(F)(F)F | 0 | validation |
| 144207420 | C1CCC2CCCCC2C1 | 0 | test |
| 144207419 | C[Si](C)(C)Cl | 0 | train |
| 144207418 | CNC(=O)C1=CC=CC=C1 | 0 | train |
| 144207417 | CCCCCCCCCCCC[N+](C)(C)CCOC1=CC=CC=C1.[Br-] | 1 | validation |
| 144207416 | C(#N)C(Cl)Cl | 0 | test |
| 144207415 | C1=CC=C(C=C1)CC#N | 0 | train |
| 144207414 | CC(C)NC(C)C | 0 | train |
| 144207413 | CC1=CC(=CC=C1)N.Cl | 1 | validation |
| 144207412 | C(C(=O)N)I | 0 | test |
| 144207411 | CCCCN(CCCC)C1=CC(=C(C=C1)C(=O)C2=CC=CC=C2C(=O)O)O | 0 | train |
| 144207410 | CCC(OCCCOC)OC(=O)C | 0 | train |
| 144207409 | C1=CC=C(C=C1)COC(=O)N[C@@H](CC(=O)O)C(=O)O | 0 | validation |
| 144207408 | COCCC#N | 0 | test |
| 144207407 | C(CNCCO)N | 0 | train |
| 144207406 | CC(=O)O.C1CCNC(C1)CNC(=O)C2=C(C=CC(=C2)OCC(F)(F)F)OCC(F)(F)F | 0 | train |
| 144207405 | CCC1CO1 | 0 | validation |
| 144207404 | C[C@@H]1CCC(=C(C)C)C(=O)C1 | 0 | test |
| 144207403 | CC(Cl)Cl | 0 | train |
| 144207402 | C1=CC(=C(C=C1Cl)Cl)[N+](=O)[O-] | 0 | train |
| 144207401 | CC1=CC=CO1 | 0 | validation |
| 144207400 | C1CCCC1 | 0 | test |
| 144207399 | CC1=C(C(=CC=C1)[N+](=O)[O-])C(=O)O | 0 | train |
| 144207398 | C=CCO | 0 | train |
| 144207397 | COC1=C(C=CC(=C1)Cl)OC2=C(C=C(C=C2)Cl)Cl | 1 | validation |
| 144207396 | C1=CC(=CC=C1C(=O)O)NN | 1 | test |
| 144207395 | [Cl-].[Cl-].[Fe+2] | 0 | train |
| 144207394 | CN1C(=O)C2=C(C1=O)C=C(C=C2)[N+](=O)[O-] | 0 | train |
| 144207393 | CCCCCCCCC=C | 0 | validation |
| 144207392 | C(CS)C(=O)O | 0 | test |
| 144207391 | CCCCCCCCCC(CC)C1=CC=C(C=C1)S(=O)(=O)[O-].[Na+] | 0 | train |

| | | | |
|---|---|---|---|
| 144207390 | CC(C)(C)Cl | 0 | train |
| 144207389 | CN(C)C1=CC=C(C=C1)N | 1 | validation |
| 144207388 | C1=CC2=C(C=C1[N+](=O)[O-])C(=O)NC2=O | 0 | test |
| 144207387 | C(Br)(Br)Br | 0 | train |
| 144207386 | C1=C(C=C(C(=C1Cl)Cl)Cl)O | 1 | train |
| 144207385 | CC1=CC=CC=C1C(=O)N | 0 | validation |
| 144207384 | CC1=CC=CC(=C1)C=C | 0 | test |
| 144207383 | CCC=CC#N | 0 | train |
| 144207382 | CC(C)COC(=O)C=C | 0 | train |
| 144207381 | C1=CC(=C(C(=C1C2=C(C(=C(C=C2)Cl)Cl)Cl)Cl)Cl)Cl | 1 | validation |
| 144207380 | CNCCC(C1=CC=CC=C1)OC2=CC=C(C=C2)C(F)(F)F.Cl | 1 | test |
| 144207379 | Cl[Sn](Cl)(Cl)Cl | 0 | train |
| 144207378 | CC(=O)OC1CCC(CC1)C(C)(C)C | 0 | train |
| 144207377 | CCOCCC(=O)OCC | 0 | validation |
| 144207376 | COC(=O)CCCCCCCCC(=O)OC | 0 | test |
| 144207375 | CC(C)CNCC(C)C | 0 | train |
| 144207374 | CCCCCCCCCOCC1CO1 | 1 | train |
| 144207373 | CC1=CCC(CC1O)C(=C)C | 1 | validation |
| 144207372 | CC1=C(C2=CC=CC=C2C=C1)[N+](=O)[O-] | 0 | test |
| 144207371 | CCCCCCCC(=O)OCC=C | 0 | train |
| 144207370 | COC1=CC(=CC2=C1OCO2)CC=C | 0 | train |
| 144207369 | C1=CC=C(C=C1)C2=CSC(=N2)N.O.Br | 1 | validation |
| 144207368 | C1=CC=C2C(=C1)NC(=N2)NC(=O)N | 0 | test |
| 144207367 | C1=CC(=C(C=C1C(Cl)(Cl)Cl)Cl)Cl | 1 | train |
| 144207366 | C1=CC(=CC=C1OC2=C(C=C(C=N2)Cl)Cl)OC3=C(C=C(C=N3)Cl)Cl | 0 | train |
| 144207365 | C1=CC(=C(C=C1C(=O)O)O)O | 0 | validation |
| 144207364 | C(C(OC(F)F)(F)F)(F)Cl | 0 | test |
| 144207363 | CC1(CC(CC(N1)(C)C)O)C | 0 | train |
| 144207362 | CCCCCCCCCCCCC1=CC=CC=C1O | 1 | train |
| 144207361 | C1=CC=NC=C1.Cl | 0 | validation |
| 144207360 | CC(=O)CC(=O)OC | 0 | test |
| 144207359 | CCCCOC(=O)C(=C)C | 0 | train |
| 144207358 | CCC(COCCOC(=O)C=C)(COCCOC(=O)C=C)COCCOC(=O)C=C | 1 | train |
| 144207357 | CC(=C)C1=CC=CC=C1 | 0 | validation |
| 144207356 | CC1=C(C(=CC=C1)N)C | 0 | test |
| 144207355 | COC1=CC(=CC(=C1OC)OC)CC2=CN=C(N=C2N)N | 0 | train |
| 144207354 | C1=CC(=C(C=C1Br)Br)OC2=CC(=C(C=C2Br)Br)Br | 1 | train |
| 144207353 | C1=C(C=C(C=C1Cl)Cl)C(=O)O | 0 | validation |
| 144207352 | CC1=C2C=CC3=CC=CC4=C3C2=C(C=C1)C=C4 | 1 | test |
| 144207351 | C(Cl)(Br)Br | 0 | train |
| 144207350 | CCOC(=O)CBr | 0 | train |
| 144207349 | CN1C(=C(C2=CC=CC=C2S1(=O)=O)O)C(=O)NC3=CC=CC=N3 | 0 | validation |
| 144207348 | C1=CC=C(C=C1)OC(=O)OC2=CC=CC=C2 | 0 | test |
| 144207347 | CC(=CC=O)C | 0 | train |
| 144207346 | CC(C)C(C(C)(C)COC(=O)C(C)C)O | 0 | train |
| 144207345 | CCC(CCl)Cl | 0 | validation |
| 144207344 | CCC1=CC=C(C=C1)CC | 0 | test |
| 144207343 | C([C@@H]1[C@H]([C@@H]([C@H](C(O1)O)O)O)O)O | 0 | train |
| 144207342 | CC1=C(C=C(C=C1)N=C=O)N=C=O | 1 | train |
| 144207341 | C1=CC=C(C=C1)S(=O)(=O)Cl | 0 | validation |
| 144207340 | C1=CC=C2C(=C1)C(=O)OC2=O | 0 | test |
| 144207339 | C[C@H]1C[C@@H]2[C@H](CC[C@]3([C@H]2CC[C@@]3(C(=O)C)OC(=O)C)C)[C@@]4(C1=CC(=O)CC4)C | 0 | train |
| 144207338 | C(C(C(=O)O)Br)Br | 0 | train |
| 144207337 | CC(C)CCCC(C)CCCC(C)CCCC(C)(C=C)O | 0 | validation |
| 144207336 | C1=CC=C2C(=C1)N=C(C(=N2)Cl)Cl | 0 | test |
| 144207335 | C1=CC(=CC=C1C(=O)Cl)[N+](=O)[O-] | 0 | train |
| 144207334 | C1=CC(=CC=C1C=O)Cl | 0 | train |
| 144207333 | CCCCCCCCN1C=C[N+](=C1)C.[Cl-] | 0 | validation |
| 144207332 | C1=CC=C(C=C1)CCNN.OS(=O)(=O)O | 1 | test |
| 144207331 | C(=O)NNC=O | 0 | train |
| 144207330 | CC(CCCC(C)(C)O)C=C | 0 | train |

| | | | |
|---|---|---|---|
| 144207329 | CC(C)(C)C(=O)C(Cl)Cl | 0 | validation |
| 144207328 | CN(C)C.Cl | 0 | test |
| 144207327 | C1=CC=C(C=C1)CS | 0 | train |
| 144207326 | CCCCCCCC/C=C¥CCCCCCCCOCCO | 1 | train |
| 144207325 | CC(C)NCC(C1=CC=C(C=C1)NS(=O)(=O)C)O.Cl | 0 | validation |
| 144207324 | C1=CC(=C(C=C1[N+](=O)[O-])[N+](=O)[O-])N | 0 | test |
| 144207323 | CC1=C(C=CC=C1N)N | 1 | train |
| 144207322 | CC1=CSC=N1 | 0 | train |
| 144207321 | CC(C)(C(=O)O)O | 0 | validation |
| 144207320 | C[C@@H]1C[C@H]2[C@@H]3CCC4=CC(=O)C=C[C@@]4([C@]3([C@H](C[C@@]2([C@]1(C(=O)CO)O)C)O)F)C | 0 | test |
| 144207319 | C1=CC=C(C=C1)C2=CC(=CC=C2)Br | 0 | train |
| 144207318 | C1OC2=C(O1)C=C(C=C2)C=O | 0 | train |
| 144207317 | CC1=CC=C(C=C1)NC(=O)C | 0 | validation |
| 144207316 | C[N+]1=CN(C=C1)CCO.[Cl-] | 0 | test |
| 144207315 | C1=CC=C(C=C1)NC2=CC=C(C=C2)N.Cl | 1 | train |
| 144207314 | CCCC[Sn](CCCC)(OC(=O)C)OC(=O)C | 1 | train |
| 144207313 | CCCCCCCCCCCC1=CC=CC=C1 | 0 | validation |
| 144207312 | CCCCCCCCCC(=O)N(C)C | 1 | test |
| 144207311 | CC(OC(=O)C)OC(=O)C | 0 | train |
| 144207310 | CC1=CCC(CC1)C(C)(C)O | 0 | train |
| 144207309 | CCCCCCCC/C=C¥CCCCCCCCO | 0 | validation |
| 144207308 | C(C(Cl)(Cl)Cl)(O)O | 0 | test |
| 144207307 | C1CCC(C1)O | 0 | train |
| 144207306 | CC1=CCC2CC1C2(C)C | 0 | train |
| 144207305 | CC1([C@@H]([C@@H]1C(=O)O[C@H](C#N)C2=CC(=CC=C2)OC3=CC=CC=C3)/C=C(/C(F)(F)F)¥Cl)C | 0 | validation |
| 144207304 | C1[C@@H]([C@H](O[C@H]1N2C=C(C(=O)NC2=O)Br)CO)O | 1 | test |
| 144207303 | C1=C(C(=C(C(=C1Cl)Cl)Cl)Cl)O | 1 | train |
| 144207302 | C[C@H]1CC[C@@H]2[C@H]([C@H]3[C@@H](N2C1)C[C@@H]4[C@@]3(CC[C@H]5[C@H]4CC=C6[C@@]5(CCC(C6)O[C@H]7[C@@H]([C@H]([C@H]([C@H](O7)CO)O)O[C@H]8[C@@H]([C@H]([C@@H]([C@H](O8)CO)O)O)O)O[C@@H]9[C@@H]([C@@H]([C@H]([C@@H](O9)C)O)O)O)C)C)C | 1 | train |
| 144207301 | CC1=C(C=C(C=C1)N)[N+](=O)[O-] | 0 | validation |
| 144207300 | C1=CC=C(C=C1)CCBr | 1 | test |
| 144207299 | CCCCCCN1C=C[N+](=C1)C.C(F)(F)(F)S(=O)(=O)[O-] | 0 | train |
| 144207298 | C1=CC=C(C=C1)CCN=C=S | 1 | train |
| 144207297 | CC(=O)NNC(=O)C | 0 | validation |
| 144207296 | CCO[Si](CCCCl)(OCC)OCC | 0 | test |
| 144207295 | CCCOCCOCCO | 0 | train |
| 144207294 | C1CC(C2=CC=CC=C2C1)O | 0 | train |
| 144207293 | COC1=CC=C(C=C1)CCN2CCC(CC2)NC3=NC4=CC=CC=C4N3CC5=CC=C(C=C5)F | 1 | validation |
| 144207292 | CC(C)(C#C)O | 0 | test |
| 144207291 | CC1=C(C(CCC1)(C)C)/C=C/C(=C/C=C/C(=C¥C(=O)O)/C)/C | 1 | train |
| 144207290 | COP(=O)(OC)OC(=CCl)C1=CC(=C(C=C1Cl)Cl)Cl | 1 | train |
| 144207289 | CC1=CC=C(C=C1)S(=O)(=O)NC2CCCCC2 | 0 | validation |
| 144207288 | C1=CC(=CC(=C1)[N+](=O)[O-])C(=O)N | 0 | test |
| 144207287 | CC1=CC=C(C=C1)CO | 0 | train |
| 144207286 | CC=CCC#N | 1 | train |
| 144207285 | C1=CC2=C(C(=C1)Cl)N=C(S2)N | 1 | validation |
| 144207284 | CC1=CC=CC=C1C(=O)O | 0 | test |
| 144207283 | C1=CC(=C(C=C1N=C=O)Cl)Cl | 1 | train |
| 144207282 | CCCC[P+](C)(CCCC)CCCC.COS(=O)(=O)[O-] | 0 | train |
| 144207281 | C1=CC=C2C(=C1)C3=C(N2)C=NC=C3 | 0 | validation |
| 144207280 | CN1CC[C@]23[C@@H]4[C@H]1CC5=C2C(=C(C=C5)OC)O[C@H]3[C@H](C=C4)O | 0 | test |
| 144207279 | CC(CC(=O)O)CC(C)(C)C | 0 | train |
| 144207278 | CCN(CC)C1=C(C=C2C(=C1)OC3=CC(=[N+](CC)CC)C(=CC3=C2C4=C(C=C(C=C4)S(=O)(=O)[O-])S(=O)(=O)[O-])C)C.[Na+] | 0 | train |
| 144207277 | C(C(Cl)(Cl)Cl)(F)(F)F | 0 | validation |
| 144207276 | C1=CC(=CC2=C(C=C(C=C21)S(=O)(=O)O)O)N | 0 | test |
| 144207275 | CC(C)(C)CC(C)(C)N | 0 | train |
| 144207274 | C1CC1CN2C(=O)CN=C(C3=C2C=CC(=C3)Cl)C4=CC=CC=C4 | 1 | train |

| | | | |
|---|---|---|---|
| 144207273 | CC1=C(C=C(C=C1)Cl)N | 0 | validation |
| 144207272 | C(C(Cl)Cl)(Cl)Cl | 0 | test |
| 144207271 | C1=C(C(=CC(=C1Cl)[N+](=O)[O-])O)N | 1 | train |
| 144207270 | CC(=O)C1=CC=CC=C1[N+](=O)[O-] | 0 | train |
| 144207269 | C[C@]12CC[C@H]3[C@H]([C@@H]1CC[C@]2(C)O)CC[C@@H]4[C@@]3(C/C(=C/O)/C(=O)C4)C | 0 | validation |
| 144207268 | C1=C(C=C(C(=C1[N+](=O)[O-])N)Br)[N+](=O)[O-] | 1 | test |
| 144207267 | CC(=O)NNC1=CC=CC=C1 | 1 | train |
| 144207266 | C1=CC=C(C=C1)NC(=O)NC2=CC=CC=C2 | 1 | train |
| 144207265 | CC(=C)C=C | 0 | validation |
| 144207264 | C1=CC2=C3C(=C1)C=CC4=C(C=CC(=C43)C=C2)[N+](=O)[O-] | 0 | test |
| 144207263 | C[N+](C)(C)CCCl.[Cl-] | 0 | train |
| 144207262 | CCCCCCCCCCCC(=O)OC[C@H]([C@@H]1[C@@H]([C@H](CO1)OCCO)OCCO)O | 0 | train |
| 144207261 | COC1CCC=CO1 | 0 | validation |
| 144207260 | COC(OC)OC | 0 | test |
| 144207259 | CCCCC(CC)COC(=O)C1=CC=CC=C1C(=O)OCCCC | 1 | train |
| 144207258 | CC(=O)C(C)(C)C | 0 | train |
| 144207257 | COC1=C(C2=C[N+]3=C(C=C2C=C1)C4=CC(=C(C=C4CC3)OC)OC)OC.O.[Cl-] | 0 | validation |
| 144207256 | CC1=C(C=C(C=C1)N)C | 1 | test |
| 144207255 | CC(=O)OC | 0 | train |
| 144207254 | C12=C(C(=C(C(=C1Br)Br)Br)Br)C(=O)OC2=O | 0 | train |
| 144207253 | CC(=O)[O-].CC(=O)[O-].O.O.[Cd+2] | 1 | validation |
| 144207252 | C1=NC=N[N-]1.[Na+] | 0 | test |
| 144207251 | C1=CC=C(C=C1)CCl | 0 | train |
| 144207250 | CCCCCCCCN | 1 | train |
| 144207249 | CC(=CC1=CC=CC=C1)C=O | 0 | validation |
| 144207248 | C(C(=O)N)(Cl)Cl | 0 | test |
| 144207247 | C1=CC=C2C(=C1)C=CC=C2CC(=O)N | 0 | train |
| 144207246 | C1=CC=C(C=C1)NNC(=O)N | 0 | train |
| 144207245 | CC(CO)CO | 0 | validation |
| 144207244 | C(CCCNCCCCCCN)CCN | 0 | test |
| 144207243 | C1=CC2=C3C(=C1)C(=O)OC(=O)C3=CC=C2 | 0 | train |
| 144207242 | CN1C2=C(C=C(C=C2)Cl)C(=NC(C1=O)O)C3=CC=CC=C3 | 1 | train |
| 144207241 | C1(=C(C(=NC(=C1Cl)Cl)C(=O)O)Cl)N | 1 | validation |
| 144207240 | CCN(CC)C(=S)SCC(=C)Cl | 1 | test |
| 144207239 | CCC(=O)C | 0 | train |
| 144207238 | C1=CC(=C(C=C1C=O)Cl)Cl | 0 | train |
| 144207237 | C(Cl)(Cl)(Cl)Cl | 0 | validation |
| 144207236 | CC(C)OCC1CO1 | 0 | test |
| 144207235 | C=CCCCCCCC=C | 0 | train |
| 144207234 | CCCCCCN | 0 | train |
| 144207233 | C=CC1=CC=CC=N1 | 0 | validation |
| 144207232 | C(C(C(=O)O)Cl)C(=O)O | 0 | test |
| 144207231 | CNN.OS(=O)(=O)O | 0 | train |
| 144207230 | C1=CC2=C(C=C1O)OC3=C2C(=O)OC4=C3C=CC(=C4)O | 1 | train |
| 144207229 | CCC1=CC=C(C=C1)Br | 0 | validation |
| 144207228 | COC(=O)CCS | 0 | test |
| 144207227 | CC1(C2CCC(=C)C1C2)C | 0 | train |
| 144207226 | CCC(CO)(CO)CO | 0 | train |
| 144207225 | CC(=O)CCl | 0 | validation |
| 144207224 | COC1=CC=CC2=C3C(=C(C=C21)[N+](=O)[O-])C(=CC4=C3OCO4)C(=O)[O-].[Na+] | 0 | test |
| 144207223 | CCOC1=CC=CC=C1C(=O)N | 1 | train |
| 144207222 | CC(C)C=O | 0 | train |
| 144207221 | C1=C(C=C(C=C1Cl)Cl)N | 0 | validation |
| 144207220 | C1=CC(=CC(=C1)O)[N+](=O)[O-] | 0 | test |
| 144207219 | C1=CC2=C(C=C1[N+](=O)[O-])C(=O)NNC2=O | 0 | train |
| 144207218 | C[C@@H]1CC[C@H](C2=C(C[C@H]([C@@H]12)O)C)/C=C(¥C)/C(=O)O | 0 | train |
| 144207217 | C(/C=C/CCl)Cl | 0 | validation |
| 144207216 | C1=CC=C(C=C1)CBr | 1 | test |
| 144207215 | C1=C(C=C(C=C1C(=O)O)C(=O)O)C(=O)O | 0 | train |
| 144207214 | CNC1=NC=NC2=C1NC=N2 | 0 | train |
| 144207213 | C1=CC=C(C=C1)CSC#N | 0 | validation |

| | | | |
|---|---|---|---|
| 144207212 | CCN(CC)CCCl.Cl | 0 | test |
| 144207211 | CO[Si](CCCCl)(OC)OC | 0 | train |
| 144207210 | CCCCCCCCCCCCCCC(=O)OC | 0 | train |
| 144207209 | C[Si](C)(C)C | 0 | validation |
| 144207208 | CC1CCC(C(C1)O)C(C)C | 1 | test |
| 144207207 | CCOP(=S)(CC)SC1=CC=CC=C1 | 1 | train |
| 144207206 | C1CC=CC(=O)C1 | 0 | train |
| 144207205 | CC(C)CCOC(=O)C=CC1=CC=CC=C1 | 1 | validation |
| 144207204 | CCOS(=O)(=O)C | 0 | test |
| 144207203 | C1CCC(C(C1)C(=O)OCC2CO2)C(=O)OCC3CO3 | 0 | train |
| 144207202 | CSC1=CC=C(C=C1)C=O | 0 | train |
| 144207201 | C(=C¥Cl)¥Cl | 0 | validation |
| 144207200 | C1=CSC=C1 | 1 | test |
| 144207199 | CNC1=CC=C(C=C1)[N+](=O)[O-] | 1 | train |
| 144207198 | CC1=NC(=O)C2=C(N1)C=CC(=C2)CN(C)C3=CC=C(S3)C(=O)N[C@@H](CCC(=O)O)C(=O)O | 0 | train |
| 144207173 | C([C@@H]1[C@H]([C@@H]([C@H]([C@H](O1)O[C@]2([C@H]([C@@H]([C@H](O2)COS(=O)[O-])OS(=O)[O-])OS(=O)[O-])COS(=O)[O-])OS(=O)[O-])OS(=O)[O-])OS(=O)[O-])OS(=O)[O-].[Na+].[Na+].[Na+].[Na+].[Na+].[Na+].[Na+].[Na+] | 0 | validation |
| 144207171 | CC(C)(C)C(/C=C/C1=CC2=C(C=C1)OCO2)O | 1 | test |
| 144207162 | CC1(CC(=O)N(C1=O)C)C2=CC=CC=C2 | 0 | train |
| 144207154 | C[N+](C)(CCOC1=CC=CC=C1)CC2=CC=CS2.[I-] | 0 | train |
| 144207147 | CC1=C([C@H](C(=C(N1)C)C(=O)O[C@@H]2CCCN(C2)CC3=CC=CC=C3)C4=CC(=CC=C4)[N+](=O)[O-])C(=O)OC.Cl | 0 | validation |
| 144207139 | C[C@@H]1C/C=C/C=C/[C@@H]([C@@H](C[C@@H]([C@@H]([C@H]([C@@H](CC(=O)O1)OC(=O)C)OC)O[C@H]2[C@@H]([C@H]([C@@H]([C@H](O2)C)O[C@H]3C[C@@]([C@H]([C@@H](O3)C)O)(C)O)N(C)C)O)CC=O)C)O[C@@H]4CC[C@@H]([C@H](O4)C)N(C)C | 0 | test |
| 144206961 | C1=CC(=CC=C1N/N=C¥2/C(=NN(C2=O)C3=CC=C(C=C3)S(=O)(=O)[O-])C(=O)[O-])S(=O)(=O)[O-].[Na+].[Na+].[Na+] | 0 | train |
| 144206942 | C[C@@]12CCC[C@H]1[C@@H]3CC[C@H]4C[C@H](CC[C@@]4([C@H]3CC2)C)O | 0 | train |
| 144206912 | C=CC(=O)NC1=C(C=C2C(=C1)C(=NC=N2)NC3=CC(=C(C=C3)F)Cl)OCCCN4CCOCC4 | 1 | validation |
| 144206892 | C[C@H](CN1CCCC1)C(=O)C2=CC=C(C=C2)C(F)(F)F | 0 | test |
| 144206891 | C1CCN(CC1)C(=O)[C@H]2CCC(=O)N2 | 0 | train |
| 144206890 | CCCCC1C(=O)NC(=O)N(C1=O)C2CCCCC2 | 0 | train |
| 144206889 | CCN(CC)CCNC(=O)C1=C(C=C(C=C1)Cl)OCC=C | 1 | validation |
| 144206888 | CC1=NC2=C(CC3=C(O2)C=CC(=C3)C(C)C(=O)OCC(=O)N(C)C)C=C1 | 1 | test |
| 144206887 | CC1=NC2=C(CC3=C(O2)C=CC(=C3)C(C)C(=O)OCC(=O)N(C)C)C=C1 | 1 | train |
| 144206886 | CN(C)C(=O)CNC(=O)CC1=CC=CC=C1 | 0 | train |
| 144206885 | CCOC(=O)C1=CC=C(C=C1)NC(=O)CN2CCCCC2 | 1 | validation |
| 144206884 | CC(C(C1=CC=CC=C1)O)N(C)CCOC(C2=CC=CC=C2)C3=CC=CC=C3 | 0 | test |
| 144206883 | CC(=O)O[C@@H]1[C@H]2[C@H]([C@@H](C(=O)O2)OC(=O)C)OC1=O | 0 | train |
| 144206882 | C(CO[N+](=O)[O-])N(CCO[N+](=O)[O-])CCO[N+](=O)[O-] | 0 | train |
| 144206881 | C1CC12CN(C[C@@H]2N)C3=C(C=C4C(=C3Cl)N(C=C(C4=O)C(=O)O)C5C[C@H]5F)F | 0 | validation |
| 144206880 | C1CCN2C(C1)NC(=O)C23CCN(CC3)CCCN4C5=CC=CC=C5CCC6=C4C=C(C=C6)Cl | 0 | test |
| 144206879 | CO[C@]1([C@H]2N(C1=O)C(=C(CO2)CSC3=NN=NN3CCO)C(=O)O)NC(=O)CSC(F)F | 0 | train |
| 144206878 | CC1(CCCO1)CN(C)S(=O)(=O)C2=CC(=C(C=C2)Cl)S(=O)(=O)N | 0 | train |
| 144206877 | CCOP(=O)(CC)OC1=CC=C(C=C1)[N+](=O)[O-] | 0 | validation |
| 144206876 | CCCN(CCC)C(=O)C(CCC(=O)OCCCN1CCN(CC1)CCOC(=O)CC2=C(N(C3=C2C=C(C=C3)OC)C(=O)C4=CC=C(C=C4)Cl)C)NC(=O)C5=CC=CC=C5 | 0 | test |
| 144206875 | CCCCCC[C@@H](CCC)C(=O)O | 0 | train |
| 144206874 | CC(CCC1=CC2=C(C=C1)OCO2)NN.Cl | 0 | train |
| 144206873 | CC(C)(C)C1=NN=C(S1)NS(=O)(=O)C2=CC=CC=C2 | 0 | validation |
| 144206872 | CC(=O)OC1=CC=C(C=C1)C2(C(=O)NC3=CC=CC=C3O2)C4=CC=C(C=C4)OC(=O)C | 0 | test |
| 144206871 | CC(C)CCOCC(CN1CCOCC1)OC(=O)C2=CC(=C(C(=C2)OC)OC)OC | 0 | train |
| 144206870 | CC(COC1=CC=CC=C1)NN | 0 | train |
| 144206869 | C1C[C@H]2C[C@@H]1[C@H]3[C@@H]2C(=O)N(C3=O)CCCCN4CCN(CC4)C5=NC=CC=N5 | 0 | validation |
| 144206868 | CN1CCC(CC1)N(CC2=CC=CS2)C3=CC=CC=C3 | 0 | test |
| 144206867 | CN(CC(=O)O)C(=S)C1=CC=CC2=C1C=CC(=C2C(F)(F)F)OC | 0 | train |
| 144206866 | CN1C=C(C(=O)C2=C1C=C(C=C2)F)S(=O)C | 0 | train |
| 144206864 | C1CC2=CC=CC=C2C(C3=CC=CC=C31)NCCCCCCC(=O)O | 0 | validation |
| 144206863 | C1=CC=C(C(=C1)CC(=O)O)OC2=C(C=C(C=C2)Cl)Cl | 0 | test |
| 144206862 | CC(=O)C1=CC=C(C=C1)OCC(=O)N2CCCCC2 | 1 | train |

| | | | |
|---|---|---|---|
| 144206861 | C/C(=N¥O)/C1=CC=C(C=C1)OCC(=O)N2CCCCC2 | 1 | train |
| 144206860 | CC1=C(C2=C(N1CC(=O)O)C=C(C=C2)OC)C(=O)C3=CC=C(C=C3)Cl | 0 | validation |
| 144206859 | CC1=NC(=CN1C2=CC=C(C=C2)[N+](=O)[O-])[N+](=O)[O-] | 0 | test |
| 144206858 | CCCOC(=O)CC1=CC(=C(C=C1)OCC(=O)N(CC)CC)OC | 0 | train |
| 144206857 | CC1CN(CCN1)C2=C(C=C3C(=C2)N(C=C(C3=O)C(=O)O)C4=C(C=C(C=C4)F)F)F | 0 | train |
| 144206855 | CCC(=O)O[C@H]1[C@H](C[C@@H]2[C@@]1(CC[C@H]3[C@H]2CC[C@@H]4[C@@]3(C[C@@H]([C@H](C4)OC(=O)C)N5CCCCC5)C)C)[N+]6(CCCCC6)CC=C.[Br-] | 0 | validation |
| 144206854 | C1CCN(C1)NC(=O)NS(=O)(=O)C2=CC=C(C=C2)Cl | 0 | test |
| 144206853 | CC1=NN(N=N1)CC2=C(N3[C@@H]([C@@H](C3=O)NC(=O)/C(=N/OC)/C4=CSC(=N4)N)SC2)C(=O)O | 0 | train |
| 144206852 | C1[C@@H](C2=C(CN1)C(=C(C=C2)O)O)C3=CC(=C(C=C3)O)O.Cl | 0 | train |
| 144206851 | CC1C2=NN=C(N2C3=C(C=C(S3)CCC4=CC=C(C=C4)CC(C)C)C(=N1)C5=CC=CC=C5Cl)C | 1 | validation |
| 144206850 | CC(=O)OCC(=O)NC1=CC(=CC(=C1Cl)NC(=O)COC(=O)C)C#N | 0 | test |
| 144206849 | CC1=CC(=CC=C1)N(C)C(=S)OC2=CC3=C(CCC3)C=C2 | 1 | train |
| 144206848 | C1CC2=NC1=C(C3=CC=C(N3)C(=C4C=CC(=N4)C(=C5C=CC(=C2C6=CC(=CC=C6)O)N5)C7=CC(=CC=C7)O)C8=CC(=CC=C8)O)C9=CC(=CC=C9)O | 0 | train |
| 144206847 | CC1=NC(=CC(=N1)OC)NS(=O)(=O)C2=CC=C(C=C2)N | 0 | validation |
| 144206846 | CC[N+]1(CCC(=C(C2=CC=CC=C2)C3=CC=CC=C3)C1C)CC.[Br-] | 0 | test |
| 144206845 | C1=CN(C(=N1)[N+](=O)[O-])CO[C@@H](CO)[C@@H](CO)O | 0 | train |
| 144206843 | CCCCCC1NC2=C(C=C(C(=C2)C(F)(F)F)S(=O)(=O)N)S(=O)(=O)N1 | 0 | train |
| 144206842 | CC[C@H]1C[C@H]2[C@@H]3CCC4=CC(=O)CC[C@@H]4[C@H]3CC[C@@]2([C@H]1O)C | 1 | validation |
| 144206840 | CC1=C(C(C(=C(N1)COC(=O)N)C(=O)OC(C)C)C2=C(C(=CC=C2)Cl)Cl)C(=O)OC | 0 | test |
| 144206839 | C1CN(CCN1CCCC(=O)NC2C3=CC=CC=C3CSC4=CC=CC=C24)C5=CC=C(C=C5)F | 0 | train |
| 144206838 | CC(C)(C)NC[C@@H](C1=C(C=C(C=C1)O)Cl)O | 0 | train |
| 144206837 | CCCCC/C=C¥C/C=C¥CCCCCCCC(=O)NC(C)C1=CC=CC=C1 | 0 | validation |
| 144206836 | CC(C1=CC=CC=C1)NN.OS(=O)(=O)O | 0 | test |
| 144206835 | CC1=CC=CC=C1C(=O)N2CC/C(=N¥OS(=O)(=O)[O-])/C3=C2C=C(C=C3)Cl.[K+] | 0 | train |
| 144206834 | CCNC(=O)NCCCOC1=CC=CC(=C1)CN2CCCCC2 | 0 | train |
| 144206833 | CC1=CC=C(C=C1)C(=O)CC(CSC(=O)C)C(=O)O | 0 | validation |
| 144206832 | CCOC(=O)CN1C=CC=C1C2=NC(=C(S2)C3=CC=C(C=C3)OC)C4=CC=C(C=C4)OC | 1 | test |
| 144206831 | CN1[C@@H]2CC[C@H]1CC(C2)NC(=O)N3C4=CC=CC=C4NC3=O | 0 | train |
| 144206830 | C1CCN(CC1)CC2=CC(=NC=C2)OC/C=C¥CNC3=C(C(=O)C3=O)N | 0 | train |
| 144206829 | CC1=CC=C(C=C1)/C=N/N2C(=CSC2=S)C3=CC=CC=C3 | 1 | validation |
| 144206828 | CCN1C(C2=CC=CC=C2C1=O)NC3=CC=C(C=C3)OCCN4CCCCC4 | 0 | test |
| 144206827 | CCOC(=O)C1=CC=C(C=C1)OCCN2C[C@@H]([C@H]([C@@H]([C@H]2CO)O)O)O | 0 | train |
| 144206826 | CCCCCCCCCCCCCCCCCCNC(=O)OC1CCN(CC1)C(=O)OC[C@H](COC(=O)N(CC2=CC=CC=[N+]2CC)C(=O)C3=CC=CC=C3OC)OC.[Cl-] | 0 | train |
| 144206825 | CC[N+](CC)(CC)CCOC(=O)C(C1CCCC1)C2CCCC2.[Br-] | 0 | validation |
| 144206824 | CCCCCOC1=C(C=C(C=C1)C2C(=O)NC(=O)S2)OCC | 1 | test |
| 144206823 | CC(C1=CC=C(C=C1)/C=C/2¥CCCCC2=O)C(=O)O | 1 | train |
| 144206822 | CCOC(=O)NC(C)(C)CC1=CC=C(C=C1)Cl | 1 | train |
| 144206821 | CCOC(=O)OCC/C(=C(¥C)/N(CC1=CN=C(N=C1N)C)C=O)/SC(=O)OCC.Cl | 0 | validation |
| 144206820 | CC(=O)NC1=NC(=O)N(C=C1)C2COC(O2)COC(=O)C3=CC=CC=C3 | 0 | test |
| 144206819 | C1C(O[C@H](O1)CO)N2C=CC(=NC2=O)N | 0 | train |
| 144206818 | CCN1CCN(CC1)C2=NC3=C(CCCCC3)C(=C2)C4=CC=C(C=C4)F | 0 | train |
| 144206817 | CCC(C1=CC=CC=C1)C(=O)NC(=O)NC(=O)C | 0 | validation |
| 144206816 | C1=CC=C(C=C1)C(=O)SCC(=O)NCC(=O)NCC(=O)NCC(=O)O | 0 | test |
| 144206815 | CCC1=C2C=CC=C(C=C2C(=C1)S(=O)(=O)[O-])C(C)C.[Na+] | 0 | train |
| 144206814 | C1CN2C(=O)[C@@H](N=C(C3=C2C1=CC=C3)C4=CC=CC=C4F)NC(=O)C5=CC6=CC=CC=C6N5 | 1 | train |
| 144206813 | C1CN2C(=O)[C@H](N=C(C3=C2C1=CC=C3)C4=CC=CC=C4F)NC(=O)C5=CC6=CC=CC=C6N5 | 0 | validation |
| 144206811 | C1=CC(=CC(=C1)CC(=O)O)C2=C(C=CC(=C2)CCCCCCC3=CC(=C(C=C3)O[C@@H]4[C@H]([C@H]([C@@H]([C@H](O4)CO)O)O)O)C5=CC(=CC=C5)CC(=O)O)O[C@@H]6[C@H]([C@H]([C@@H]([C@H](O6)CO)O)O)O | 0 | test |
| 144206810 | CC/C=C(/C1=CSC(=N1)N)¥C(=O)N[C@H]2[C@@H]3N(C2=O)C(=C(CS3)COC(=O)N)C(=O)OCOC(=O)C(C)(C)C.Cl | 0 | train |
| 144206809 | C1=C(C(=C(C(=C1I)NC(=O)COCCOCCOCC(=O)NC2=C(C=C(C(=C2I)C(=O)O)I)I)I)C(=O)O)I | 0 | train |
| 144206808 | C[N+]1(CCCCC1)CC2COC(O2)(C3=CC=CC=C3)C4=CC=CC=C4.[I-] | 0 | validation |
| 144206807 | CC1=CC=CC=C1C(=O)NC2=CC(=C(C=C2)C(=O)N3CCCC(C4=C3C=CC(=C4)Cl)O)C | 0 | test |
| 144206805 | C1=CC=C(C=C1)OC(=O)CCCCCCCCC#Cl | 0 | train |
| 144206804 | C1[C@H](S/C(=C(¥C#N)/N2C=CN=C2)/S1)C3=C(C=C(C=C3)Cl)Cl | 1 | train |

| | | | |
|---|---|---|---|
| 144206803 | CCCCNC1=CC=C(C=C1)C(=O)OCC[NH+](CC)CC.[Cl-] | 1 | validation |
| 144206802 | C1CCNC2=C(C1)C=CC(=C2)C(=O)CCC3CCN(CC3)CC4=CC=CC=C4.C(=C/C(=O)O)¥C(=O)O | 1 | test |
| 144206800 | CCOC(=O)C1=C2CN(C(=O)C3=C(N2C=N1)C=CC=C3I)C | 0 | train |
| 144206758 | CC1=C2C(=CC(=C1)O)CC[C@@](O2)(C)CCC[C@H](C)CCC[C@H](C)CCCC(C)C | 0 | train |
| 144206753 | CC1CC(=O)NN=C1C2=CC=C(C=C2)NCC(C)(C)NCC(COC3=C(C=CC(=C3)Cl)C#N)O | 0 | validation |
| 144206640 | CCOC1=NN=C(C=C1)NS(=O)(=O)C2=CC=C(C=C2)N | 0 | test |
| 144206631 | COP(=S)(OC)OC1=CC=C(C=C1)S(=O)(=O)N | 0 | train |
| 144206602 | C1=CC(=CC=C1C(=N)N)OCCCOC2=CC=C(C=C2)C(=N)N | 0 | train |
| 144206517 | CC1=C2C(=C(C(=C1C)OC(=O)CCC(=O)OCCO)C)CC[C@@](O2)(C)CCC[C@H](C)CCC[C@H](C)CCCC(C)C | 0 | validation |
| 144206513 | CC(=CCC/C(=C/CC/C(=C/CCC(=CCCC(=O)C)C)/C)/C)C | 1 | test |
| 144206509 | CC1([C@@H](N2[C@H](S1)[C@@H](C2=O)NC(=O)[C@@H](C3=CC=CC=C3)S(=O)(=O)[O-])C(=O)[O-])C.[Na+].[Na+] | 0 | train |
| 144206505 | C1=CC=C(C=C1)CCCCOC2=CC=C(C=C2)C(=O)NC3=CC=CC4=C3OC(=CC4=O)C5=NNN=N5 | 0 | train |
| 144206504 | C(C(COP(=O)([O-])[O-])O)O.[K+].[K+] | 0 | validation |
| 144206494 | C1=CC=C(C(=C1)C(=O)[O-])NC2=C(C=CC(=C2)Cl)C(=O)[O-].[Na+].[Na+] | 0 | train |
| 144206491 | CCCC(=O)O[C@@]1(CC[C@@H]2[C@@]1(C[C@@H]([C@H]3[C@H]2CCC4=CC(=O)CC[C@]34C)O)C)C(=O)COC(=O)CC | 0 | train |
| 144206489 | C[C@H](CCC=C(C)C)[C@H]1CC[C@@]2([C@@]1(CC[C@]34[C@H]2CC[C@@H]5[C@]3(C4)CC[C@@H](C5(C)C)OC(=O)/C=C/C6=CC(=C(C=C6)O)OC)C)C | 0 | validation |
| 144206486 | CC(C1=CC(=C(C=C1)C2=CC=CC=C2)F)C(=O)OC(C)OC(=O)C | 0 | test |
| 144206484 | CCC1=CC2=C(S1)N3C(=NN=C3CN=C2C4=CC=CC=C4Cl)C | 0 | train |
| 144206480 | CCOC(=O)[C@H](CCC1=CC=CC=C1)N[C@@H](C)C(=O)N(CC(=O)O)C2CC3=CC=CC=C3C2.Cl | 0 | train |
| 144206474 | CO/N=C(¥C1=NSC(=N1)N)/C(=O)N[C@H]2[C@@H]3N(C2=O)C(=C(CS3)CN4C=C[N+]5=C4C=CC=N5)C(=O)[O-].Cl | 0 | validation |
| 144206473 | CN1C(=NN=N1)SCC2=C(N3[C@@H]([C@@](C3=O)(NC(=O)CSC[C@H](C(=O)[O-])N)OC)SC2)C(=O)O.[Na+] | 0 | test |
| 144206472 | CC1=C(N2[C@@H]([C@@H](C2=O)NC(=O)/C(=N/OC)/C3=CSC(=N3)N)SC1)C(=O)OCOC(=O)C(C)(C)C.Cl | 0 | train |
| 144206464 | CC/C=C(/C1=CSC(=N1)N)¥C(=O)N[C@H]2[C@@H]3N(C2=O)C(=C(CS3)COC(=O)N)C(=O)OCOC(=O)C(C)(C)C.Cl | 0 | train |
| 144206463 | CC(C)(C)NCC(=O)NC1=C(C2=C(C[C@H]3C[C@H]4[C@@H](C(=O)C(=C([C@]4(C(=O)C3=C2O)O)O)C(=O)N)N(C)C)C(=C1)N(C)C)O | 0 | validation |
| 144206461 | C1[C@@H]2C[C@@H]2N([C@@H]1C#N)C(=O)[C@H](C34CC5CC(C3)CC(C5)(C4)O)N | 0 | test |
| 144206457 | C[C@H](CSC(=O)C)C(=O)N1CCC[C@H]1C(=O)N[C@@H](CC2=CC=CC=C2)C(=O)O | 0 | train |
| 144206456 | C1=CC(=C(C=C1C(F)(F)F)Cl)OC2=CC(=C(C=C2)[N+](=O)[O-])C(=O)[O-].[Na+] | 0 | train |
| 144206453 | CCC(=O)N(C1CCN(CC1)C(C)CC2=CC=CC=C2)C3=CC=CC=C3.Cl | 0 | validation |
| 144206452 | C[C@]([C@H]1C[C@@]23CCC1([C@H]4[C@@]25CCN([C@@H]3CC6=C5C(=C(C=C6)O)O4)CC7CC7)OC)(C(C)(C)C)O.Cl | 1 | test |
| 144206451 | CN(C)CCC1=CNC2=C1C(=CC=C2)OP(=O)(O)O | 0 | train |
| 144206450 | CC[C@H]1C[C@@H]2C[C@@H]3[C@H]1N(C2)CCC4=C3NC5=C4C=C(C=C5)OC.Cl | 0 | train |
| 144206449 | CC(CC1=CC=CC=C1)NCCN2C=NC3=C2C(=O)N(C(=O)N3C)C.Cl | 0 | validation |
| 144206448 | CCC(=O)C(CC(C)N(C)C)(C1=CC=CC=C1)C2=CC=CC=C2.Cl | 0 | test |
| 144206447 | CN(C)CCC1=CNC2=C1C(=CC=C2)O | 0 | train |
| 144206446 | CCCN(CCC)CCC1=CNC2=CC=CC=C21.Cl | 0 | train |
| 144206445 | CN(C)CCC1=CNC2=CC=CC=C21.C(=C/C(=O)O)¥C(=O)O | 0 | validation |
| 144206444 | CCN(CC)CCC1=CNC2=CC=CC=C21.CCN(CC)CCC1=CNC2=CC=CC=C21.C(=C/C(=O)O)¥C(=O)O | 0 | test |
| 144206443 | CN(C)CCC1=CNC2=C1C=C(C=C2)O | 0 | train |
| 144206442 | CC(CC1=CC2=C(C=C1)OCO2)NC.Cl | 0 | train |
| 144206441 | CCNC(C)CC1=CC2=C(C=C1)OCO2.Cl | 0 | validation |
| 144206439 | CN1CC[C@]23[C@@H]4[C@H]1CC5=C2C(=C(C=C5)O)O[C@H]3[C@H](CC4)O | 0 | test |
| 144206438 | CN1CC[C@]23[C@@H]4[C@H]1CC5=C2C(=C(C=C5)OC)O[C@H]3[C@@H](CC4)O | 0 | train |
| 144206437 | CN1CC[C@]23[C@@H]4[C@H]1CC5=C2C(=C(C=C5)OC)O[C@H]3[C@H](C=C4)O.Cl | 1 | train |
| 144206435 | C1NCCN(C1)CC2=CC=CC=C2.C(=C/C(=O)O)¥C(=O)O.C(=C/C(=O)O)¥C(=O)O | 0 | validation |
| 144206434 | C1CCC(CC1)(C2=CC=CC=C2)N.Cl | 1 | test |
| 144206433 | CC(C)N(CCC1=CNC2=C1C=C(C=C2)OC)C(C)C.Cl | 0 | train |
| 144206432 | CCC(=O)N(C1=CC=CC=C1)C2(CCN(CC2)CCC3=CC=CS3)COC.C(C(=O)O)C(CC(=O)O)(C(=O)O)O | 1 | train |

| | | | |
|---|---|---|---|
| 144206431 | CCC(=O)N(C1=CC=CC=C1)C2(CCN(CC2)CCN3C(=O)N(N=N3)CC)COC.Cl | 0 | validation |
| 144206430 | C[C@H]1[C@H]2CC3=C([C@@]1(CCN2CC=C(C)C)C)C=C(C=C3)O.C(CC(=O)O)C(=O)O | 0 | test |
| 144206428 | COC1=CC(=C(C=C1CCN)OC)Br.Cl | 1 | train |
| 144206427 | C[C@H](CC1=CC(=C(C=C1OC)Br)OC)N.Cl | 0 | train |
| 144206425 | CCN(CC)C(=O)[C@H]1CN([C@@H]2CC3=CNC4=CC=CC(=C34)C2=C1)C | 0 | validation |
| 144206423 | CC1=NC2=CC=CC=C2C(=O)N1C3=CC=CC=C3Cl | 0 | test |
| 144206420 | CCC[C@@H](C)C1(C(=O)NC(=O)NC1=O)CC=C | 0 | train |
| 144206419 | CCC1(C(=O)NC(=O)NC1=O)CCC(C)C | 0 | train |
| 144206418 | CCN[C@H](C)CC1=CC(=CC=C1)C(F)(F)F.Cl | 0 | validation |
| 144206415 | C1CN[C@@H]2CC3=C4[C@@]15[C@H]2C=C[C@@H]([C@@H]5OC4=C(C=C3)O)O.Cl | 0 | test |
| 144206414 | CN1CC[C@]23[C@@H]4[C@H]1CC5=C2C(=C(C=C5)O)O[C@H]3[C@H](C=C4)O.CN1CC[C@]2<br>3[C@@H]4[C@H]1CC5=C2C(=C(C=C5)O)O[C@H]3[C@H](C=C4)O.O.O.O.O.O.OS(=O)(=O)O | 0 | train |
| 144206412 | CC(CC(C#N)(C1=CC=CC=C1)C2=CC=CC=C2)N(C)C | 1 | train |
| 144206411 | CCN[C@@H](C)CC1=CC=CC=C1.Cl | 0 | validation |
| 144206405 | CC(CC1=CC2=C(C=C1)OCO2)NO.Cl | 0 | test |
| 144206403 | CC(CC1=C(C=CC(=C1)OC)OC)N.Cl | 0 | train |
| 144206401 | CC1=CC(=C(C=C1OC)C[C@@H](C)N)OC.Cl | 0 | train |
| 144206400 | CCC(=O)N(C1CCN(CC1)CCC2=CC=CS2)C3=CC=CC=C3.Cl | 0 | validation |
| 144206399 | CCC(=O)N(C1CCN(CC1)CC2=CC=CS2)C3=CC=CC=C3.Cl | 0 | test |
| 144206398 | CCC(=O)N(C1CCN(CC1C)CCC2=CC=CS2)C3=CC=CC=C3.Cl | 1 | train |
| 144206397 | CCC(=O)N(C1CCN(CC1)C(C)CC2=CC=CS2)C3=CC=CC=C3.Cl | 0 | train |
| 144206396 | CCC(=O)N(C1CCN(CC1)CC(C2=CC=CC=C2)O)C3=CC=CC=C3.Cl | 0 | validation |
| 144206395 | CC(CC1=CC2=C(C(=C1)OC)OCO2)N.Cl | 0 | test |
| 144206393 | CCC1=CC(=C(C=C1OC)C[C@H](C)N)OC.Cl | 0 | train |
| 144206392 | CCC(=O)N(C1CCN(CC1)CC2=CC=CC=C2)C3=CC=CC=C3.Cl | 0 | train |
| 144206390 | CCC(=O)N([C@@H]1CCN(C[C@@H]1C)CCC2=CC=CC=C2)C3=CC=CC=C3.Cl | 0 | validation |
| 144206389 | CCC(=O)N(C1CCN(CC1)CCC2=CC=CC=C2)C3=CC=C(C=C3)F | 0 | test |
| 144206386 | CC(=O)OC1(CCN(CC1)CCC2=CC=CC=C2)C3=CC=CC=C3 | 0 | train |
| 144206385 | CCC(=O)OC1(CCN(CC1)C)C2=CC=CC=C2.Cl | 0 | train |
| 144206384 | CC[C@@H](C(CC(C)NC)(C1=CC=CC=C1)C2=CC=CC=C2)OC(=O)C.Cl | 1 | validation |
| 144206383 | CCN(CC)CCN1C2=C(C=C(C=C2)[N+](=O)[O-])N=C1CC3=CC=C(C=C3)OCC | 1 | test |
| 144206382 | C[C@H](CN1CCOCC1)C(C2=CC=CC=C2)(C3=CC=CC=C3)C(=O)N4CCCC4.[C@@H]([C@H](C(<br>=O)O)O)(C(=O)O)O | 0 | train |
| 144206380 | CC[C@@H](C(C[C@H](C)N(C)C)(C1=CC=CC=C1)C2=CC=CC=C2)O.Cl | 0 | train |
| 144206379 | C[C@H]1[C@@H]2CC3=C([C@]1(CCN2C)C)C=C(C=C3)O.C[C@H]1[C@@H]2CC3=C([C@]1(CC<br>N2C)C)C=C(C=C3)O.C(=C/C(=O)O)¥C(=O)O | 0 | validation |
| 144206376 | CC(C)CC1=CC(=C(C(=C1)OC)OC)OC.Cl | 0 | test |
| 144206375 | COC1=CC(=CC(=C1OC)OC)CCN.Cl | 0 | train |
| 144206371 | C[C@@H]1[C@@H](OC(=N1)N)C2=CC=CC=C2 | 0 | train |
| 144206369 | C[C@@H](C(=O)C1=CC=CC=C1)N.Cl | 0 | validation |
| 144206333 | C1=CC=C(C(=C1)C(=O)[O-])[O-].C1=CC=C(C(=C1)C(=O)[O-])[O-].O.O.O.O.[Mg+2] | 0 | test |
| 144206330 | CCCCOC(=O)CC(CC(=O)OCCCC)(C(=O)OCCCC)OC(=O)C | 1 | train |
| 144206329 | CCCC(=O)OC[C@H]([C@H]([C@H](CN1C2=C(C=C(C(=C2)C)C)N=C3C1=NC(=O)NC3=O)OC(<br>=O)CCC)OC(=O)CCC)OC(=O)CCC | 1 | train |
| 144206328 | CC1=CC(=C(C(=C1NC(=O)CN(CC(=O)O)CC(=O)O)C)Br)C | 0 | validation |
| 144206326 | CC1=NC=C(C(=N1)N)CN(C=O)C(=C(CCO)SSC(=C(C)N(CC2=CN=C(N=C2N)C)C=O)CCO)C | 0 | test |
| 144206325 | C[C@]12CC[C@H]3[C@H]([C@@H]1CC[C@]2(C)O)CC[C@@H]4[C@@]3(CC5=NON=C5C4)C | 0 | train |
| 144206117 | CO[C@H]1[C@@H](C[C@@H]2CN3CCC4=C([C@H]3C[C@@H]2[C@@H]1C(=O)OC)NC5=C4C=<br>CC(=C5)OC)OC(=O)COC6=CC=C(C=C6)OC | 0 | train |
| 144205501 | CN1C(=S)CN=C(C2=C1C=CC(=C2)Cl)C3=CC=CC=C3 | 1 | validation |

activity score 0:non-toxic, activity score 1:toxic
