# Supplementary material for: Optimization of a Deep-Learning Method Based on the Classification of Images Generated by Parameterized Deep Snap a Novel Molecular-Image-Input Technique for Quantitative Structure–Activity Relationship (QSAR) Analysis
Source: Front Bioeng Biotechnol. 2019 Mar 28;7:65. doi: 10.3389/fbioe.2019.00065 (PMC6447703; doi:10.3389/fbioe.2019.00065)
Supplement: Supplementary file 2 [file Data_Sheet_2.PDF]

Supplementary table 2. Numbers of pictures in train, validation, and test datasets used in optimization of parameter of Deep Snap.

No. of picture for one molecule	Activity score	Train	Validation	Test	Sum
3	0 : Non-toxic	10,953	5,574	5,634	22,161
3	1 : Toxic	3,330	1,569	1,509	6,408
	Sum	14,283	7,143	7,143	28,569
4	0 : Non-toxic	14,604	7,432	7,512	29,548
4	1 : Toxic	4,440	2,092	2,012	8,544
	Sum	19,044	9,524	9,524	38,092
8	0 : Non-toxic	29,208	14,864	15,024	59,096
8	1 : Toxic	8,880	4,184	4,024	17,088
	Sum	38,088	19,048	19,048	76,184
