# Supplementary figures and images for: Optimization of a Deep-Learning Method Based on the Classification of Images Generated by Parameterized Deep Snap a Novel Molecular-Image-Input Technique for Quantitative Structure–Activity Relationship (QSAR) Analysis
Source: Front Bioeng Biotechnol. 2019 Mar 28;7:65. doi: 10.3389/fbioe.2019.00065 (PMC6447703; doi:10.3389/fbioe.2019.00065)

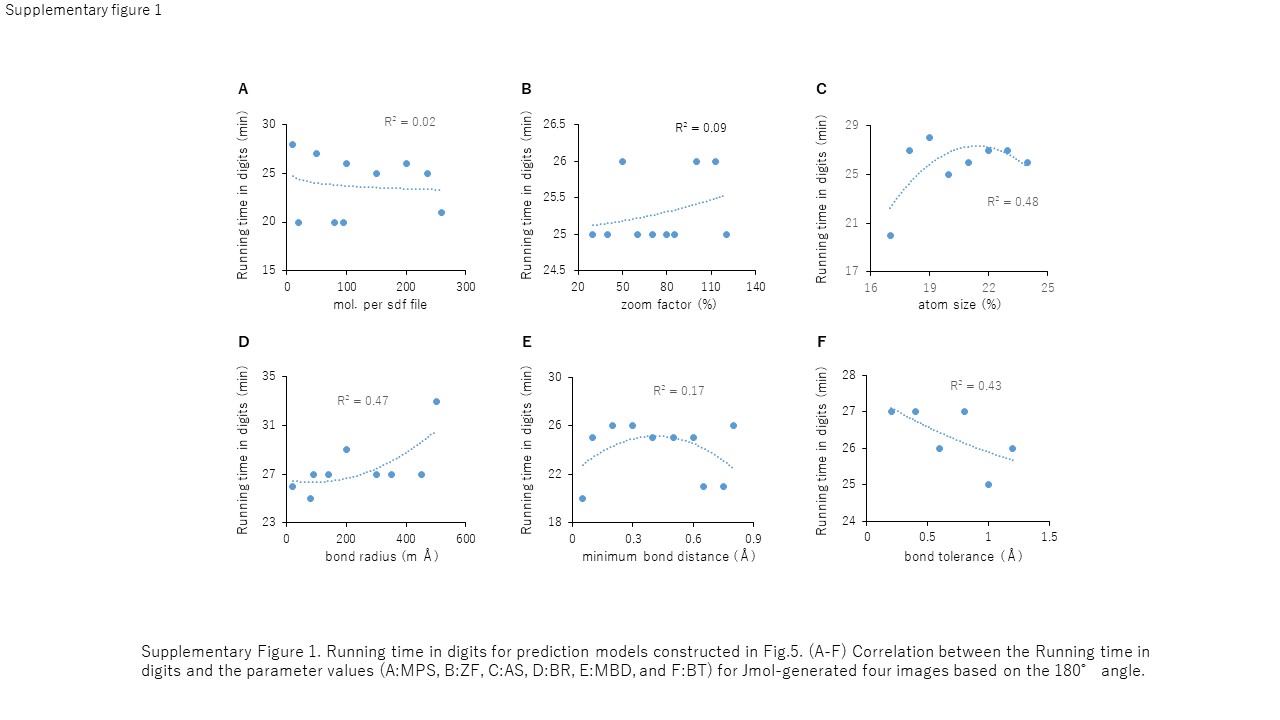

Supplement: Supplementary file 3 [file Image_1.JPEG]
